# Supplementary material for: Recent trend reversal for declining European seagrass meadows
Source: Nat Commun. 2019 Jul 26;10:3356. doi: 10.1038/s41467-019-11340-4 (PMC6659699; doi:10.1038/s41467-019-11340-4)
Supplement: Supplementary file 1 — Supplementary Information [file 41467_2019_11340_MOESM1_ESM.pdf]

Recent trend reversal for declining European seagrass meadows

de los Santos *et al.*, 2019

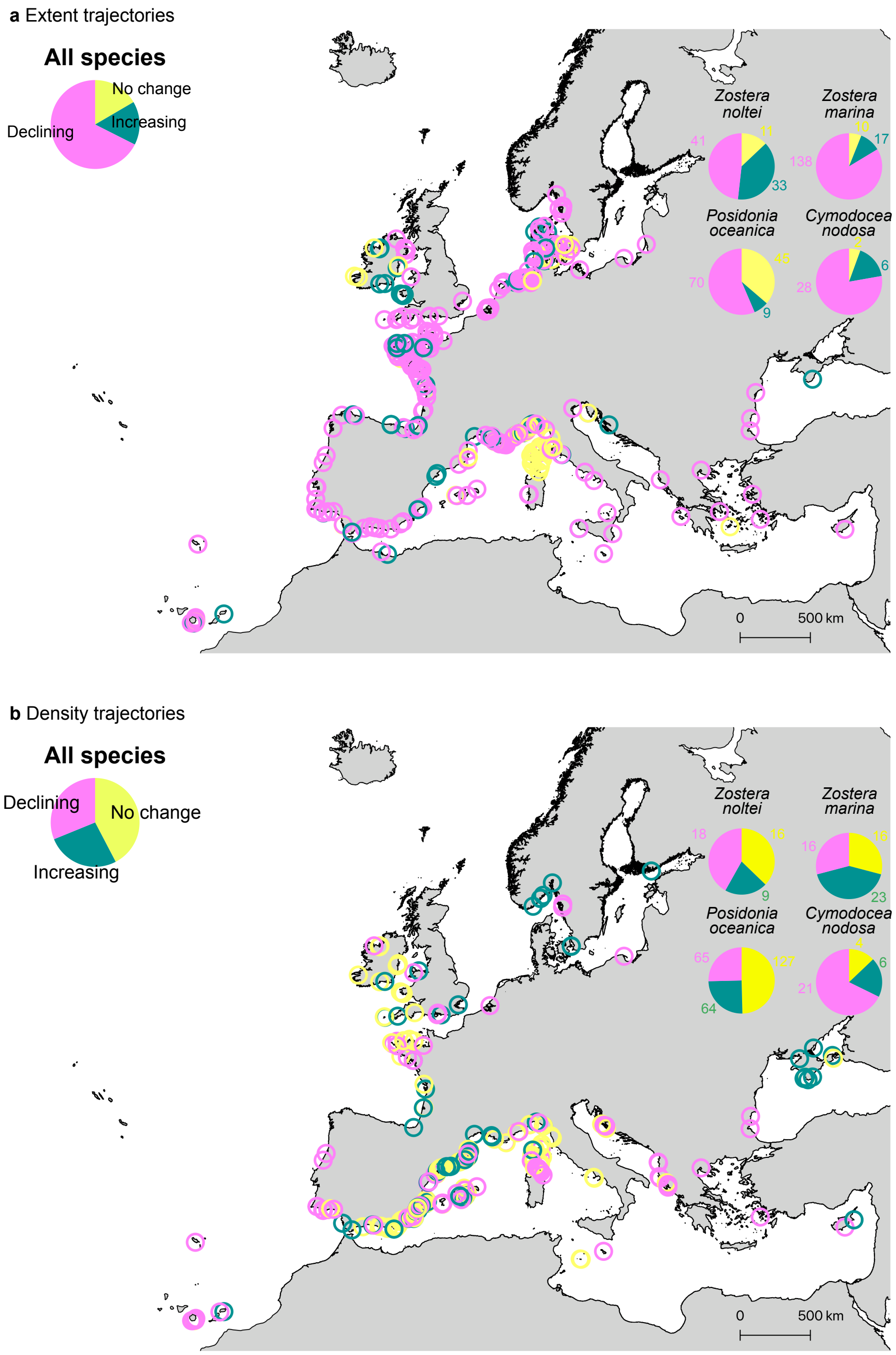

Supplementary Figure 1. Distribution of compiled seagrass sites in Europe and their trajectories. Seagrass sites in Europe showing no change (yellow circles, n = 68 for extent and n = 163 for density), increase (green circles, n = 65 for extent and n = 102 for density), and decline (magenta circles, n = 277 for extent and n = 120 for density) trajectories between 1869 and 2016 for (A) extent metrics (occurrence, area, depth limits) and (B) density metrics (cover, shoot density, biomass). Pie charts show the overall and species-specific frequencies of trajectories. Number of sites per trajectories for each species is given next to the pie charts. Trajectories are based on the available time-series reports and thus correspond to different time windows.

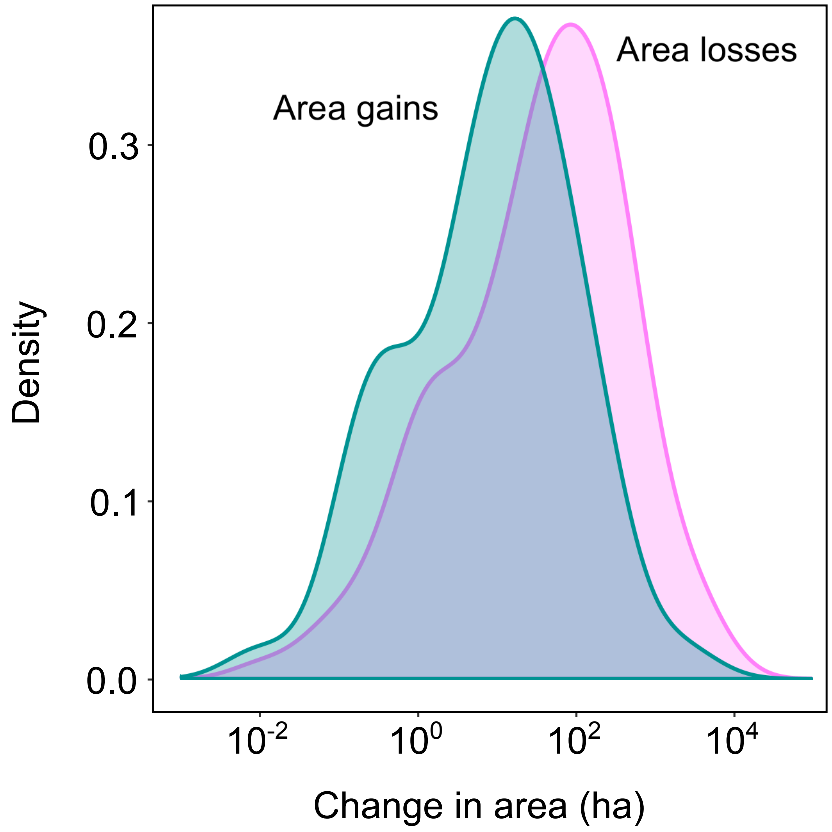

Supplementary Figure 2. Changes in seagrass area for the compiled sites in Europe. Density plots for the losses (magenta, n = 136) and gains (green, n = 56) in area of European seagrasses compiled between 1869 and 2016. Note the $\log_{10}$ x-scale.

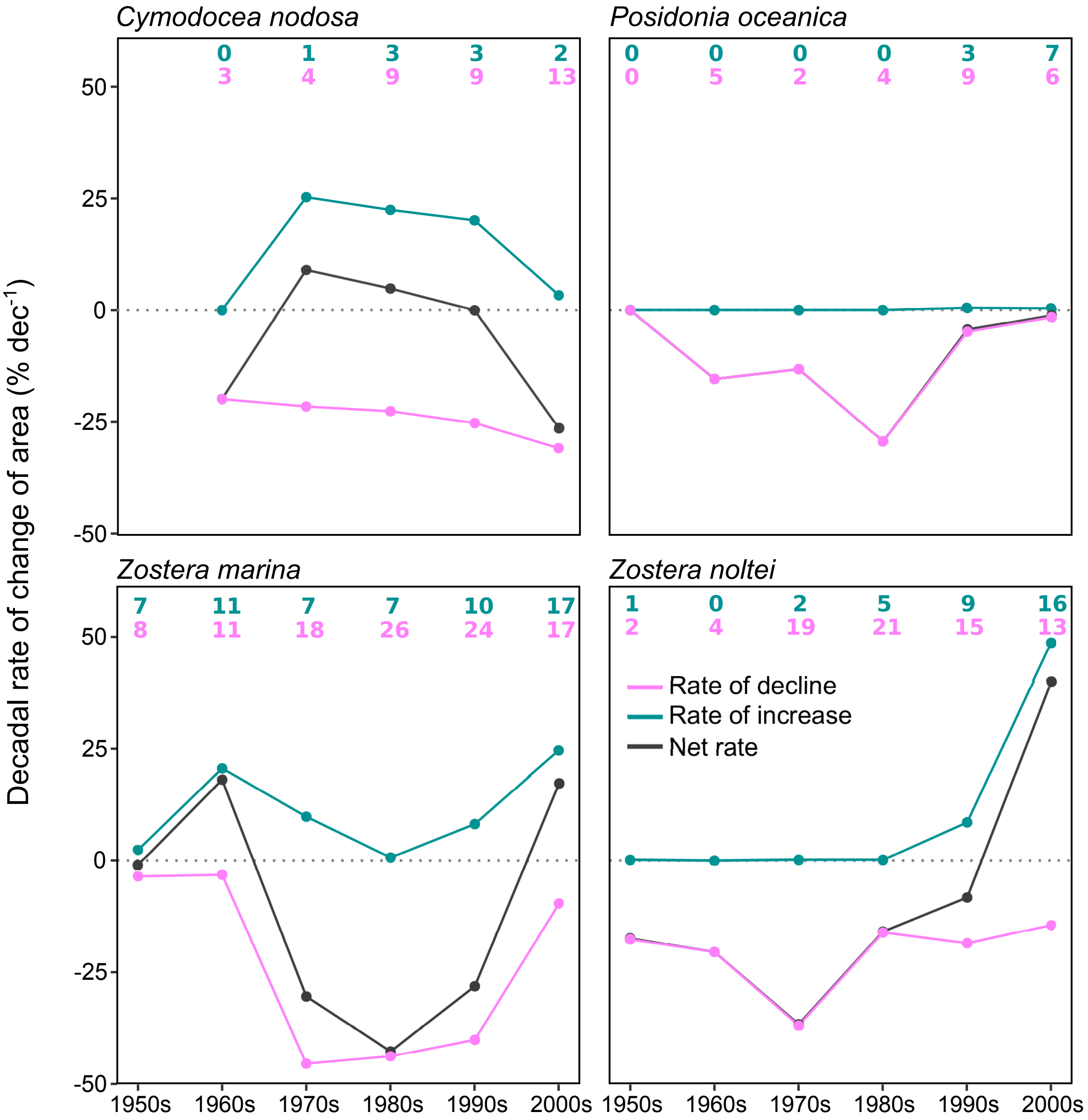

Supplementary Figure 3. Decadal rate of change of area of European seagrass species (1950s – 2000s). Decadal analysis includes time-series > 8 years. Number of sites per decade and trajectory are given at the top of the plot.

Supplementary Table 1. Nature of the combination of multiple causes in documented seagrass losses.

**Cause 1**	**Cause 2**	**Cause 3**	**N**
Coastal modification	Extreme events		1
Coastal modification	Mechanical damage		3
Extreme events	Mechanical damage		2
Extreme events	Overgrazing		2
Extreme events	Algae invasion		1
Mechanical damage	Algae invasion		1
Water quality degradation	Coastal modification		7
Water quality degradation	Extreme events		1
Water quality degradation	Mechanical damage		3
Water quality degradation	Overgrazing		1
Coastal modification	Extreme events	Mechanical damage	1
Water quality degradation	Coastal modification	Extreme events	2
Water quality degradation	Coastal modification	Mechanical damage	1
Water quality degradation	Coastal modification	Wasting disease	1

N: number of sites reported per each combination.
