# Supplementary material for: Recent trend reversal for declining European seagrass meadows
Source: Nat Commun. 2019 Jul 26;10:3356. doi: 10.1038/s41467-019-11340-4 (PMC6659699; doi:10.1038/s41467-019-11340-4)
Supplement: Supplementary file 3 — Reporting Summary [file 41467_2019_11340_MOESM3_ESM.pdf]

nature research

Corresponding author(s): Carmen B. de los Santos.

Reporting Summary

Nature Research wishes to improve the reproducibility of the work that we publish. This form provides structure for consistency and transparency in reporting. For further information on Nature Research policies, see Authors & Referees and the Editorial Policy Checklist.

Statistical parameters

When statistical analyses are reported, confirm that the following items are present in the relevant location (e.g. figure legend, table legend, main text, or Methods section).

n/a	Confirmed	
☐	☒	The exact sample size (n) for each experimental group/condition, given as a discrete number and unit of measurement
☒	☐	An indication of whether measurements were taken from distinct samples or whether the same sample was measured repeatedly
☐	☒	The statistical test(s) used AND whether they are one- or two-sided<br>*Only common tests should be described solely by name; describe more complex techniques in the Methods section.*
☒	☐	A description of all covariates tested
☐	☒	A description of any assumptions or corrections, such as tests of normality and adjustment for multiple comparisons
☐	☒	A full description of the statistics including central tendency (e.g. means) or other basic estimates (e.g. regression coefficient) AND variation (e.g. standard deviation) or associated estimates of uncertainty (e.g. confidence intervals)
☐	☒	For null hypothesis testing, the test statistic (e.g. F, t, r) with confidence intervals, effect sizes, degrees of freedom and P value noted<br>*Give P values as exact values whenever suitable.*
☒	☐	For Bayesian analysis, information on the choice of priors and Markov chain Monte Carlo settings
☒	☐	For hierarchical and complex designs, identification of the appropriate level for tests and full reporting of outcomes
☒	☐	Estimates of effect sizes (e.g. Cohen's d, Pearson's r), indicating how they were calculated
☐	☒	Clearly defined error bars<br>*State explicitly what error bars represent (e.g. SD, SE, CI)*

Our web collection on statistics for biologists may be useful.

Software and code

Policy information about availability of computer code

Data collection	Data was collected from sources of information (articles, thesis, etc...). When the data were not directly available from the source, data values were inferred from graphical representations using GraphicClick (©2008 Arizona Software) or ImageJ (US National Institutes of Health).
Data analysis	Data and statistical analysis were performed using R Statistical Package:<br>R Core Team (2018). R: A language and environment for statistical computing. R Foundation for Statistical Computing, Vienna, Austria. URL https://www.R-project.org/.

For manuscripts utilizing custom algorithms or software that are central to the research but not yet described in published literature, software must be made available to editors/reviewers upon request. We strongly encourage code deposition in a community repository (e.g. GitHub). See the Nature Research guidelines for submitting code & software for further information.

Data

Policy information about availability of data

All manuscripts must include a data availability statement. This statement should provide the following information, where applicable:

- Accession codes, unique identifiers, or web links for publicly available datasets
- A list of figures that have associated raw data
- A description of any restrictions on data availability

The dataset of compiled seagrass sites and time-series are available as Supplementary Data 1 and Data 2, respectively. Source data of tables and figures are provided as a Source Data file. All other relevant data is available on request.

Field-specific reporting

Please select the best fit for your research. If you are not sure, read the appropriate sections before making your selection.

☐ Life sciences ☐ Behavioural & social sciences ☒ Ecological, evolutionary & environmental sciences

For a reference copy of the document with all sections, see nature.com/authors/policies/ReportingSummary-flat.pdf

Ecological, evolutionary & environmental sciences study design

All studies must disclose on these points even when the disclosure is negative.

Study description	We collated existing assessments of changes in the extent and density of European seagrasses from 1869 to 2016, to assess the continental-scale trends over time and to identify the drivers of change, both for loss and recovery.
Research sample	The research sample is a seagrass site in Europe. Each site was classified by the seagrass species for monospecific meadows or by the dominant species for mixed meadows. When a site included separate assessments for depth (i.e. shallow meadow and deep meadow) or for co-occurring species, those observations were considered as independent sites.<br><br>The study includes time-series of the seagrass species Cymodocea nodosa, Posidonia oceanica, Zostera marina and Z. noltei, the four native seagrass species along European shores.
Sampling strategy	Sample size of number of sites was based on the available time-series.
Data collection	Published sources were gathered using the search browser GoogleScholar in April 2013 and then again in December 2017, by combining keywords related to seagrasses (seagrass, Posidonia, Cymodocea, Zostera, or eelgrass), with changes (loss, gain, decline, increase, stability, recovery, change, long-term, evolution, dynamic, impact, and diachronic), and names of European countries or regional seas. These searches, together with the authors' personal data collections, yielded 520 potential time-series sources, from which 241 were kept.<br><br>Sources were sorted in 166 journal articles, 33 technical reports, 12 book chapters, 13 conference proceedings, and 6 PhD or MSc theses. In addition, 11 verified databases were facilitated by participants of the COST Action ES0906 (Seagrass productivity: from genes to ecosystem management) in 2013. The thirty-four European sites included in the previous global review of seagrass trends (4) (16 % in their database) were added to our database, accounting for 4 % of our compilation, and 13 of them were updated based on new studies reporting recent observations. Several data verification steps were conducted, including independent checks by authors of the sources they provided, proof-reading the data twice and identifying and verifying outliers against original sources. When not directly available from the source, data values were inferred from graphical representations using GraphicClick (©2008 Arizona Software) or ImageJ (US National Institutes of Health). Dataset and references are available from Supplementary Data 1.
Timing and spatial scale	The overall dataset covered 147 years, from 1869 to 2016, with the observation effort increasing exponentially over time. The time series was highly variable among sites, from 1 to 121 years with a median of 9 years.<br><br>The geographical range of the sites was: 27.744 to 59.840 degrees for latitude, and -16.936 to 36.504 degrees for longitude. 56 % of the sites were in the Mediterranean Sea (including the Black Sea), 38 % in the European North Atlantic Ocean and 6 % in the Baltic Sea. Sites were distributed across 25 countries (EU-countries: Bulgaria, Croatia, Cyprus, Denmark, Finland, France, Germany, Greece, Ireland, Italy, Lithuania, Malta, Poland, Portugal, Romania, Slovenia, Spain, Sweden, The Netherlands, United Kingdom, and non-EU countries: Albania, Monaco, Norway, Turkey-in-Europe, Ukraine).
Data exclusions	The reasons for exclusion were: 1) inaccessible source, 2) source including a site already compiled or updated in another more recent source, 3) source being a review or compilation (in those cases, the source was consulted to find potential studies to assess); and, 4) sources in which data criteria were not met (e.g. type of metrics).
Reproducibility	This study does not include experimental trials. Reproducibility of the data analsyses is available through the R codes written for data handling, statistics, summaries and plots. R code is available from corresponding author upon request.
Randomization	Randomization is not relevant in this study because seagrass sites were sorted into species or region groups based on the information given in the sources.

Blinding	Blinding in the data analyses was accomplished by fixing the used criteria (e.g. definition of trajectory, minimum length of time-series, excluding sites with "no change" trajectory from the quantitative analsysis) before the exploratory and confirmatory analysis. In addition, definition of null hypotheses and selection of statistical tests were done prior to the test performance.

Did the study involve field work? ☐ Yes ☒ No

Reporting for specific materials, systems and methods

Materials & experimental systems

n/a	Involved in the study
☒	☐ Unique biological materials
☒	☐ Antibodies
☒	☐ Eukaryotic cell lines
☒	☐ Palaeontology
☒	☐ Animals and other organisms
☒	☐ Human research participants

Methods

n/a	Involved in the study
☒	☐ ChIP-seq
☒	☐ Flow cytometry
☒	☐ MRI-based neuroimaging
