# Supplementary material for: Recent trend reversal for declining European seagrass meadows
Source: Nat Commun. 2019 Jul 26;10:3356. doi: 10.1038/s41467-019-11340-4 (PMC6659699; doi:10.1038/s41467-019-11340-4)
Supplement: Supplementary file 4 — Description of Additional Supplementary Files [file 41467_2019_11340_MOESM4_ESM.pdf]

Description of Additional Supplementary Files

File Name: Supplementary Data 1

Description: Dataset of compiled studies reporting changes in extent and/or density of seagrass sites in Europe, with time-windows from 1869 to 2016. Metadata and list of sources are given within the file.

File Name: Supplementary Data 2

Description: Visualisation of the compiled time-series of extent and/or density of seagrass sites in Europe, with time-windows from 1869 to 2016. Elements shown in each figure are explained within the file.
