# Supplementary material for: Recent trend reversal for declining European seagrass meadows
Source: Nat Commun. 2019 Jul 26;10:3356. doi: 10.1038/s41467-019-11340-4 (PMC6659699; doi:10.1038/s41467-019-11340-4)

Supplementary Data 2. Visualisation of the compiled time-series of extent and/or density of seagrass sites in Europe, with time-windows from 1869 to 2016.

Elements in each figure are (see example below):

1. Code of the assessment (site ID_metric), as in Supplementary Data 1.
2. Code of the source, as in Supplementary Data 1.
3. Name of the site (country - region) - Species code (if known, depth in m).
4. Overall analysis: Net rate of change (units depend on the metric), specific rate of change (% yr-1), and percentage of change (%) for the overall time period (from first to last observation) > trajectory for the overall time period (decrease, increase, no change).
5. Decadal analysis: Whether the time-series is included or not in the decadal analysis (YES or NO). Time window is given between brackets (in years). Next lines shows: decade, trajectory for that decade using the observations at the decade boundaries, evolution of the trajectory (from previous decade), specific rate of change for that decade (% yr-1), and time window available for that decade.
6. Dark blue dots are observed values, light blue dots are interpolated values, purple circles show values used for the decadal calculations, red crosses indicate absence of seagrasses for that year (either based on observation or interpolations).
7. Grey dashed lines separate decades.

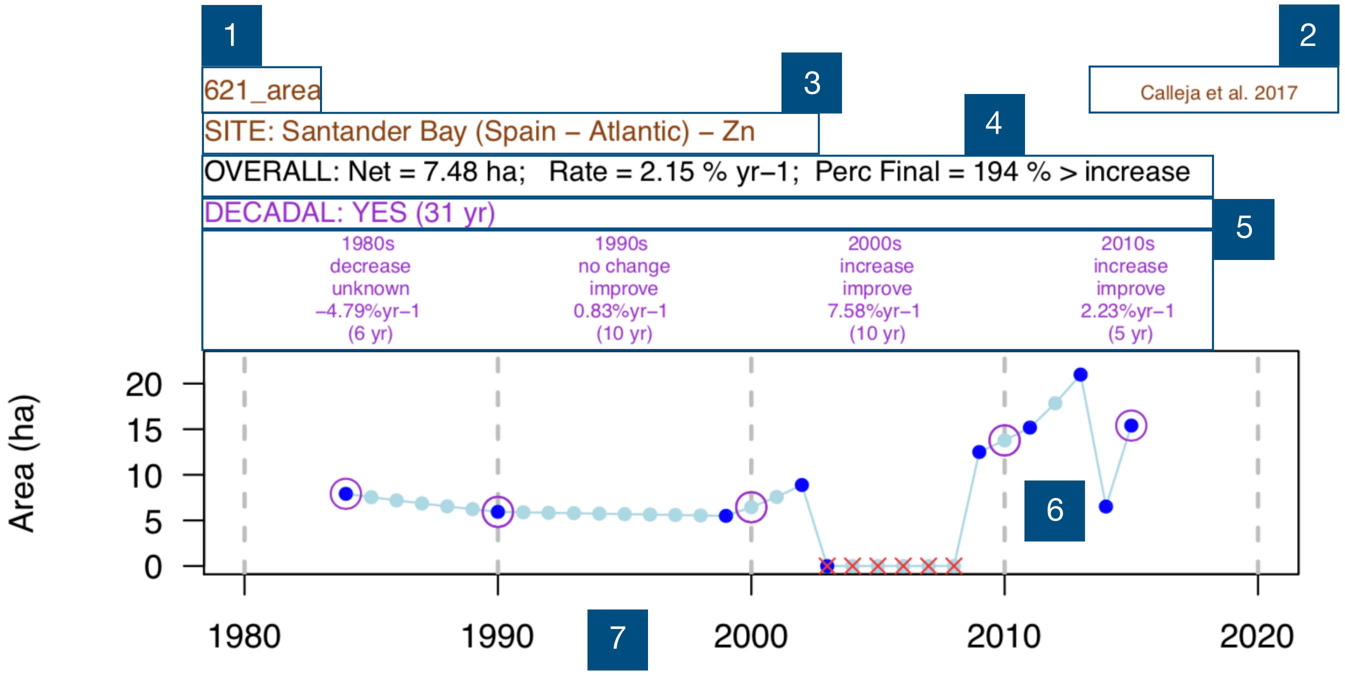

3_area

Cochón and Sánchez 2005

SITE: Ría de Pontevedra (Spain – Atlantic) – Zn (? m)

OVERALL: Net = –167.94 ha;   Rate = –2.09 % yr–1; Perc Final = 32 % > decrease

DECADAL: YES (54 yr)

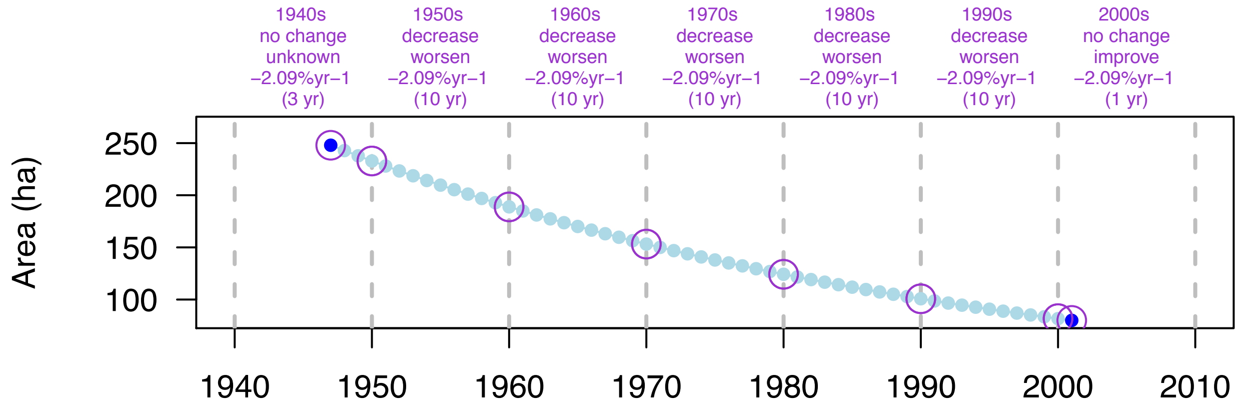

5_area

Castellanos et al. 2003

SITE: Ría de Foz (Spain – Atlantic) – Zn (? m)

OVERALL: Net = 24.5 ha;   Rate = 1.54 % yr–1; Perc Final = 132 % > increase

DECADAL: YES (18 yr)

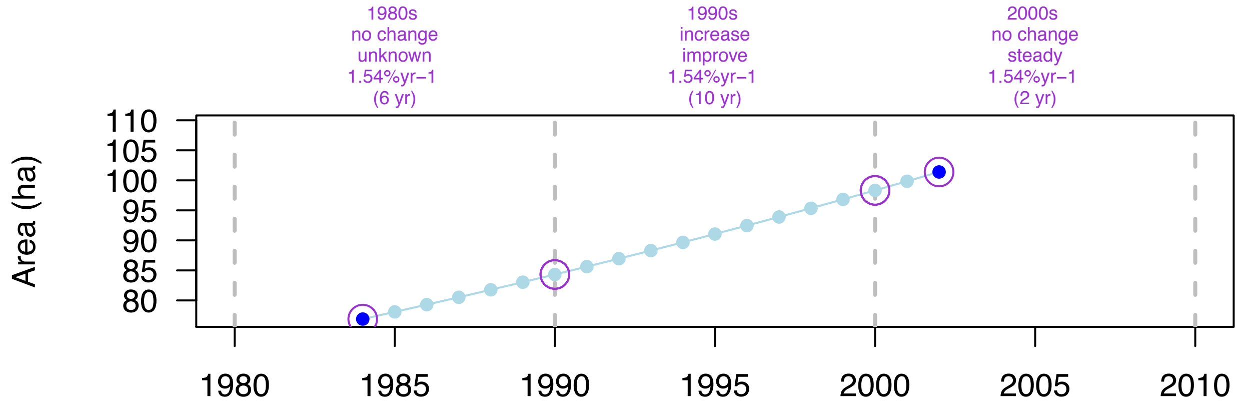

7_lowerlimit

Whelan 1986 (in Hily et al. 2003)

SITE: Ventry Bay (Ireland – Atlantic) – Zm (–13 m)

OVERALL: Net = –3 m;   Rate = –13.12 % yr–1; Perc Final = 77 % > decrease

DECADAL: NO (2 yr)

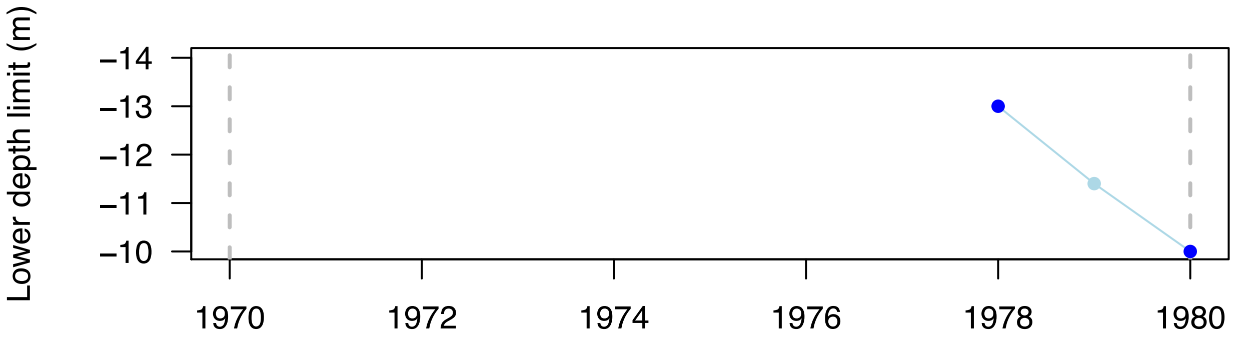

8_area

Tagliapietra et al. 1998, Rismonto and Mion 2008

SITE: Venice Lagoon (Italy – Mediterranean) – Zn (? m)

OVERALL: Net = –28 ha;   Rate = –54.93 % yr–1; Perc Final = 33 % > decrease

DECADAL: NO (2 yr)

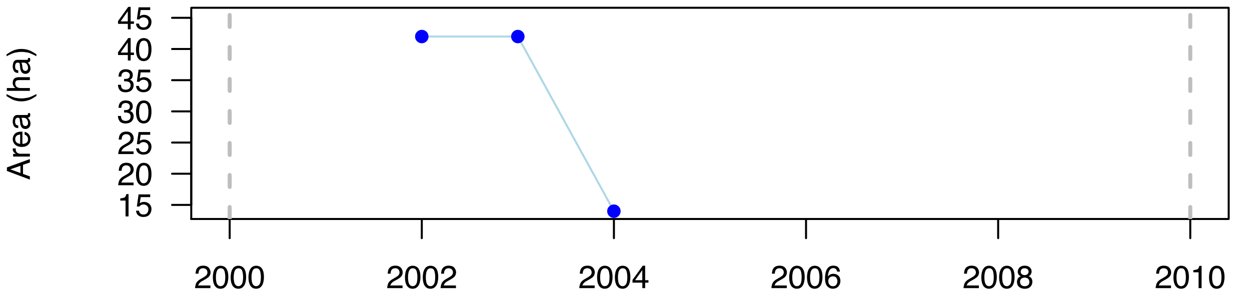

9_area

Arconada et al. 2013

SITE: Águilas (desalination plant) (Spain – Mediterranean) – Po (? m)

OVERALL: Net = −25.86 ha;   Rate = −3.77 % yr−1; Perc Final = 77 % > decrease

DECADAL: NO (7 yr)

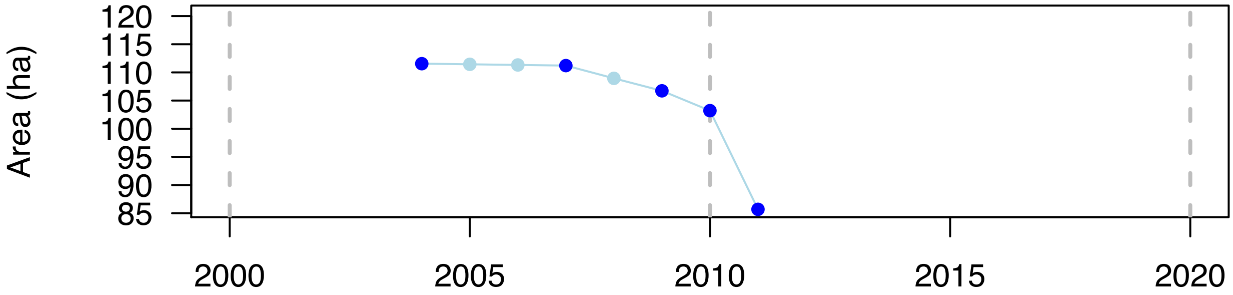

11_area

Ardizzone et al. 2006

SITE: Cape Circeo and Sperlonga (Italy – Mediterranean) – Po (? m)

OVERALL: Net = −4364.8 ha;   Rate = −1.98 % yr−1; Perc Final = 40 % > decrease

DECADAL: YES (46 yr)

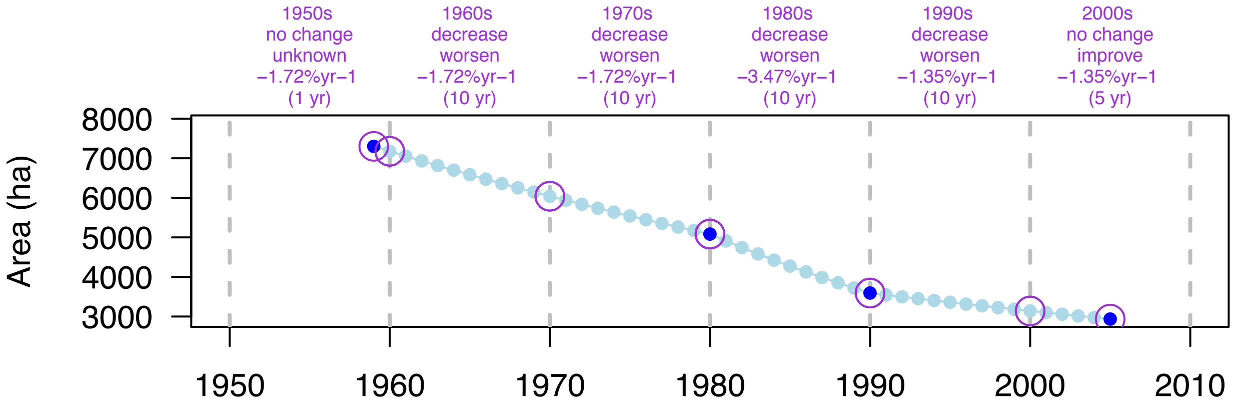

11_lowerlimit

Ardizzone et al. 2006

SITE: Cape Circeo and Sperlonga (Italy – Mediterranean) – Po (? m)

OVERALL: Net = –10.63 m;   Rate = –0.78 % yr–1; Perc Final = 70 % > decrease

DECADAL: YES (46 yr)

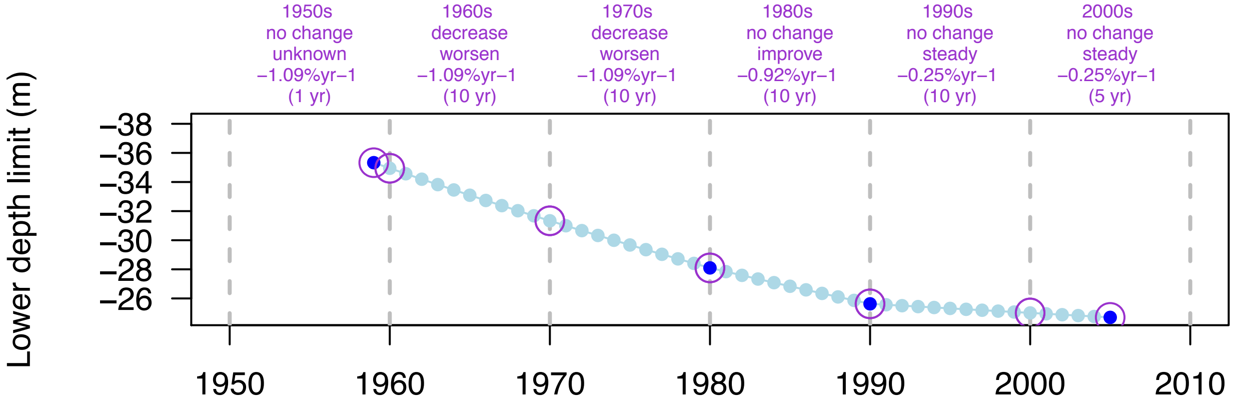

11_upperlimit

Ardizzone et al. 2006

SITE: Cape Circeo and Sperlonga (Italy – Mediterranean) – Po (? m)

OVERALL: Net = –2.33 m;   Rate = –0.33 % yr–1; Perc Final = 86 % > decrease

DECADAL: YES (46 yr)

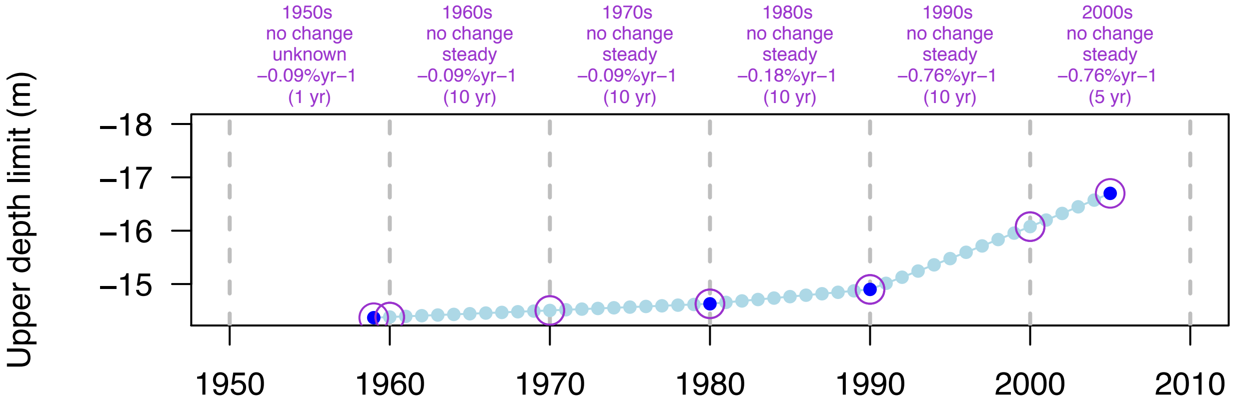

12_cover

Marbà et al. 2015

SITE: Agua Amarga (POS_08) (Spain – Mediterranean) – Po (−13 m)

OVERALL: Net = −10.5 %;   Rate = −12.2 % yr−1; Perc Final = 78 % > no change

DECADAL: NO (2 yr)

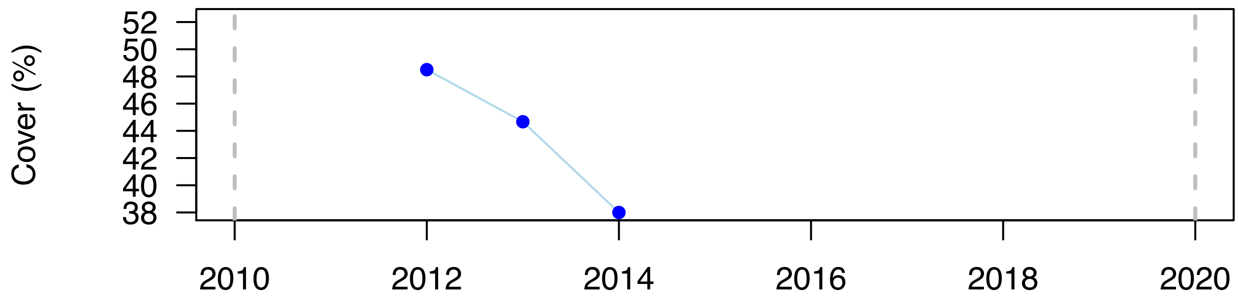

12_density

Marbà et al. 2015

SITE: Agua Amarga (POS_08) (Spain – Mediterranean) – Po (−13 m)

OVERALL: Net = −111.12 shoot m−2;   Rate = −14.93 % yr−1; Perc Final = 74 % > decrease

DECADAL: NO (2 yr)

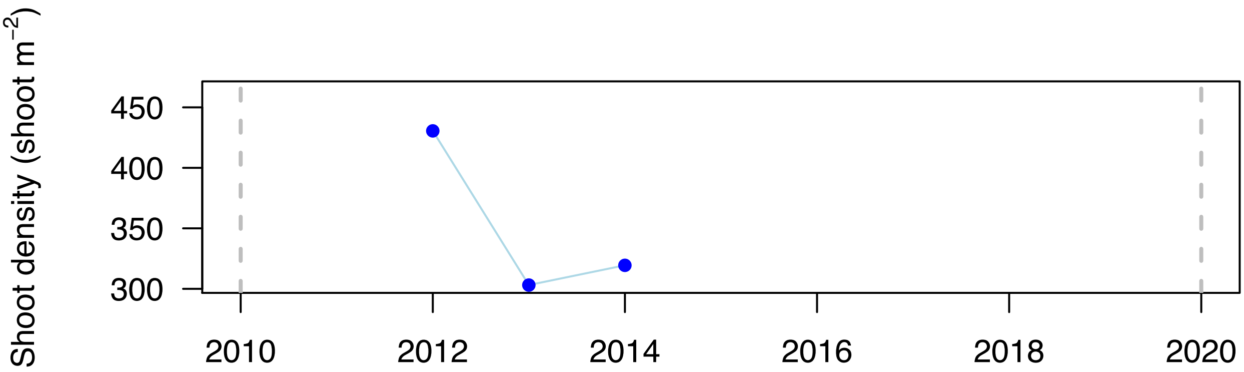

13_density

Marbà et al. 2015

SITE: Carnaje (POS_10) (Spain – Mediterranean) – Po (–13 m)

OVERALL: Net = 43.97 shoot m–2;   Rate = 4.21 % yr–1; Perc Final = 109 % > no change

DECADAL: NO (2 yr)

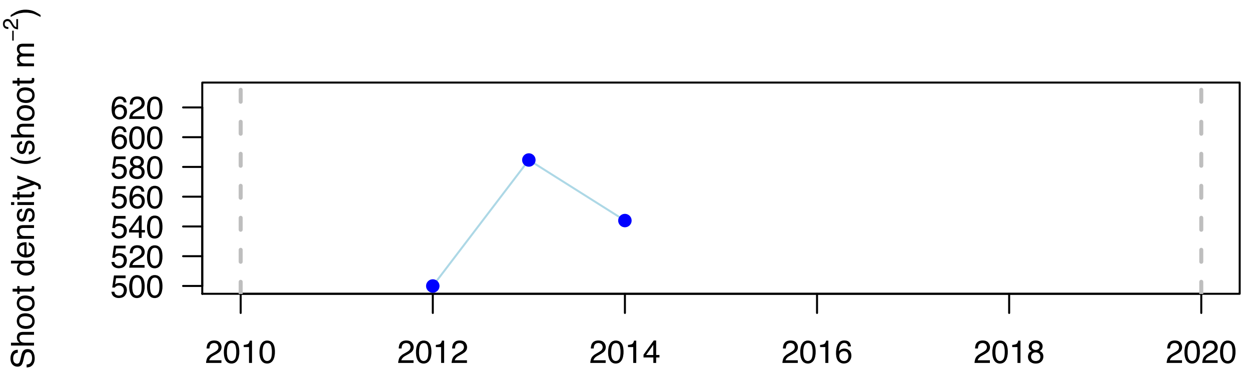

14_cover

Marbà et al. 2015

SITE: Las Negras (POS_09) (Spain – Mediterranean) – Po (–11 m)

OVERALL: Net = 22.53 %;   Rate = 17.69 % yr–1; Perc Final = 142 % > increase

DECADAL: NO (2 yr)

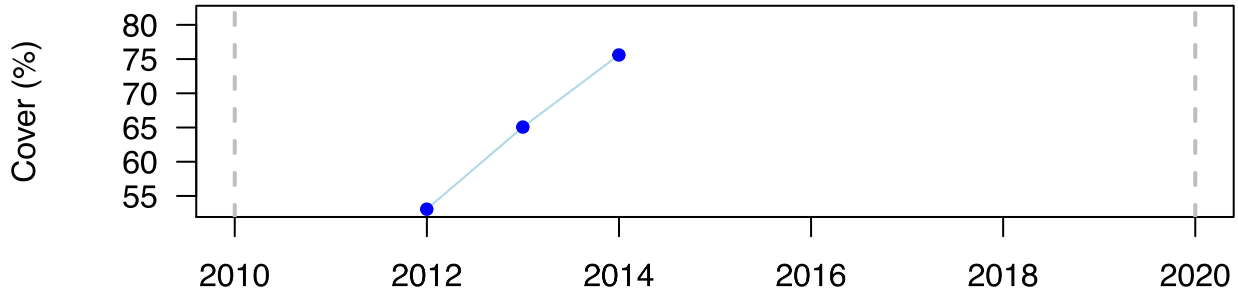

14_density Marbà et al. 2015

SITE: Las Negras (POS_09) (Spain – Mediterranean) – Po (–11 m)

OVERALL: Net = –33.92 shoot m–2; Rate = –2.49 % yr–1; Perc Final = 95 % > no change

DECADAL: NO (2 yr)

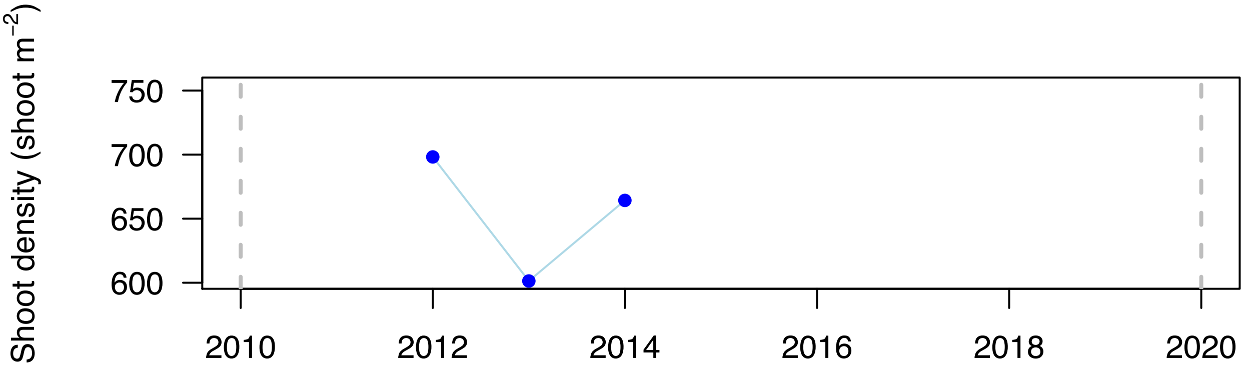

15_cover Marbà et al. 2015

SITE: Los Escullos (POS_11) (Spain – Mediterranean) – Po (–12 m)

OVERALL: Net = 0 %; Rate = 0 % yr–1; Perc Final = 100 % > no change

DECADAL: NO (2 yr)

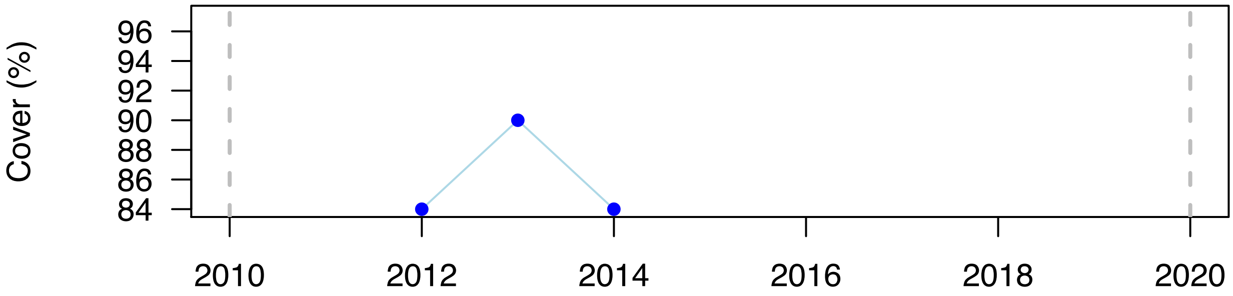

15_density

Marbà et al. 2015

SITE: Los Escullos (POS_11) (Spain – Mediterranean) – Po (–12 m)

OVERALL: Net = –166.48 shoot m–2;   Rate = –17.32 % yr–1; Perc Final = 71 % > decrease

DECADAL: NO (2 yr)

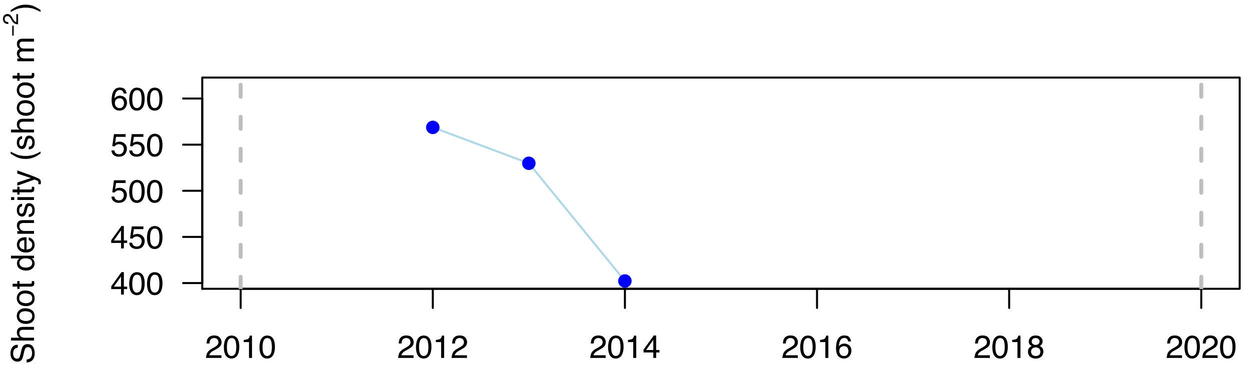

16_cover

Marbà et al. 2015

SITE: Bajos de Roquetas (POS_12) (Spain – Mediterranean) – Po (–11.5 m)

OVERALL: Net = 2.53 %;   Rate = 1.72 % yr–1; Perc Final = 103 % > no change

DECADAL: NO (2 yr)

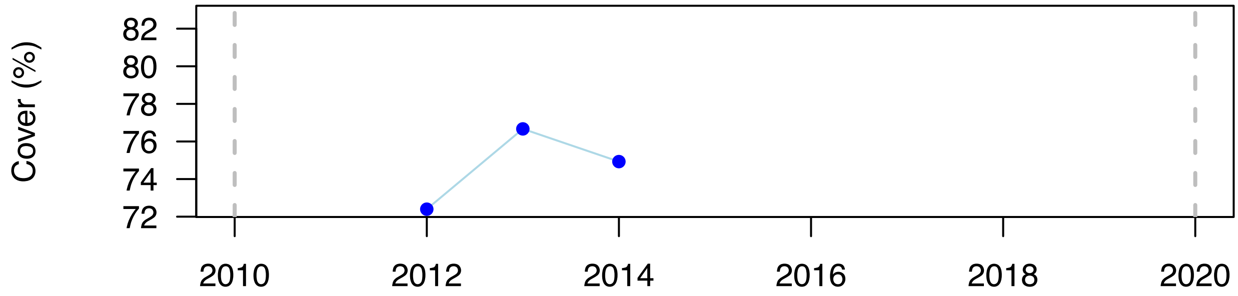

16_density

Marbà et al. 2015

SITE: Bajos de Roquetas (POS_12) (Spain – Mediterranean) – Po (–11.5 m)

OVERALL: Net = –60.56 shoot m–2; Rate = –7 % yr–1; Perc Final = 87 % > no change

DECADAL: NO (2 yr)

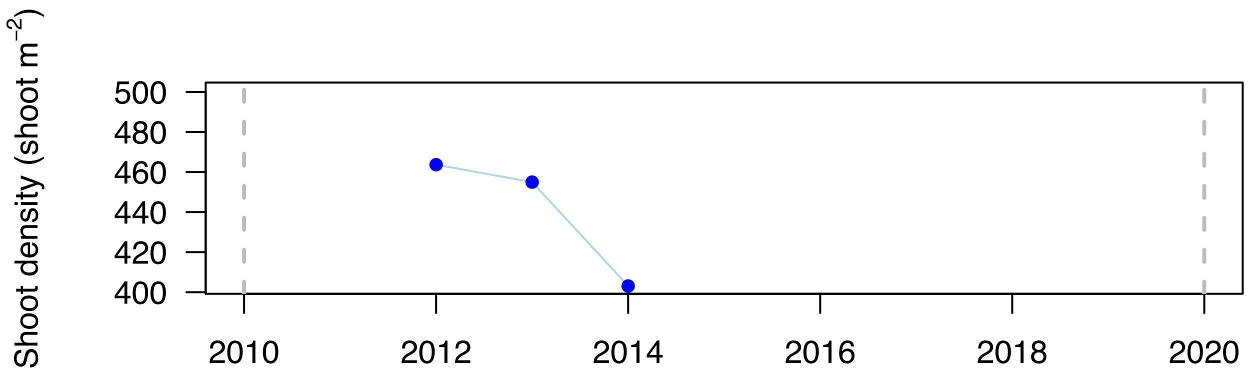

17_cover

Marbà et al. 2015

SITE: Punta Entinas (POS_14) (Spain – Mediterranean) – Po (–11 m)

OVERALL: Net = 1.33 %; Rate = 0.8 % yr–1; Perc Final = 102 % > no change

DECADAL: NO (2 yr)

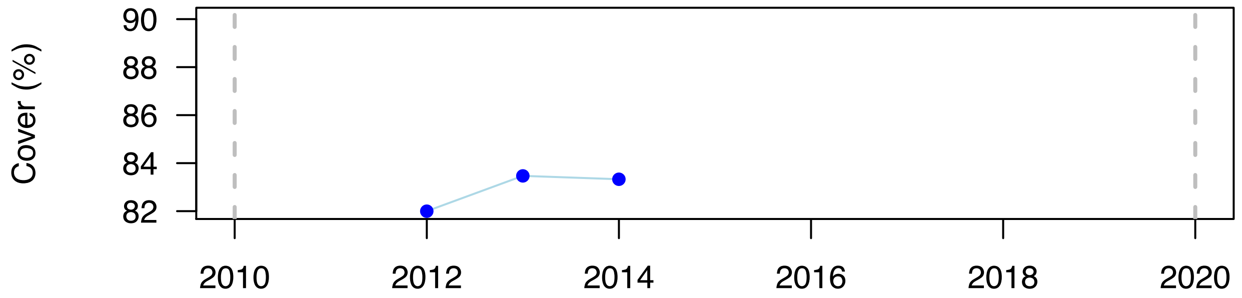

17_density

Marbà et al. 2015

SITE: Punta Entinas (POS_14) (Spain – Mediterranean) – Po (–11 m)

OVERALL: Net = 79.49 shoot m–2;   Rate = 9.87 % yr–1; Perc Final = 122 % > no change

DECADAL: NO (2 yr)

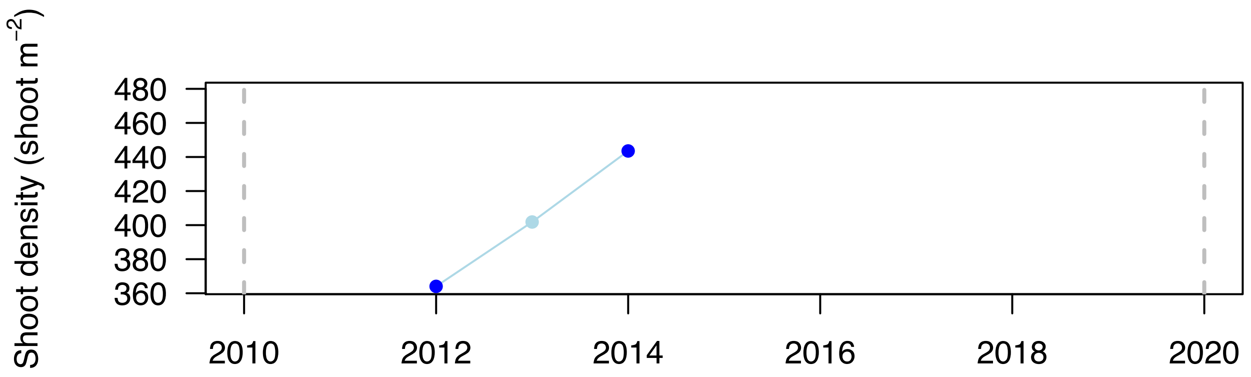

18_cover

Marbà et al. 2015

SITE: Cocedores (POS_01) (Spain – Mediterranean) – Po (–11 m)

OVERALL: Net = 12.07 %;   Rate = 7.42 % yr–1; Perc Final = 116 % > no change

DECADAL: NO (2 yr)

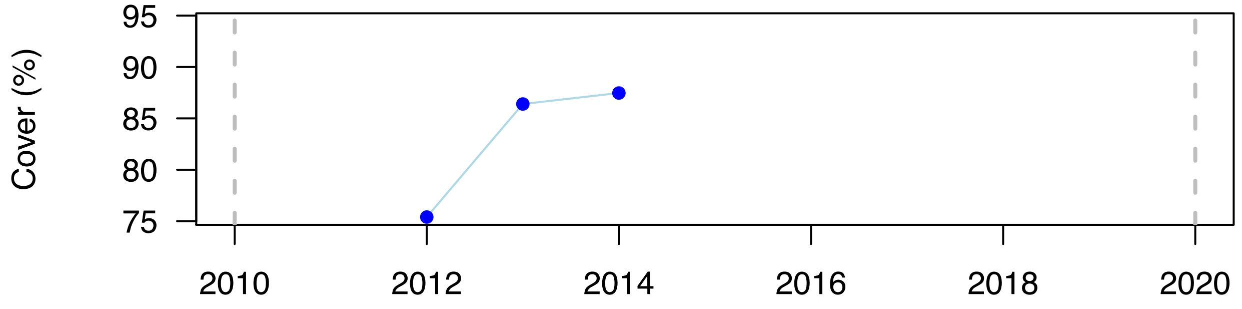

19_cover

Marbà et al. 2015

SITE: Isla de Terreros (POS_02) (Spain – Mediterranean) – Po (–11 m)

OVERALL: Net = 16 %; Rate = 12.57 % yr–1; Perc Final = 129 % > increase

DECADAL: NO (2 yr)

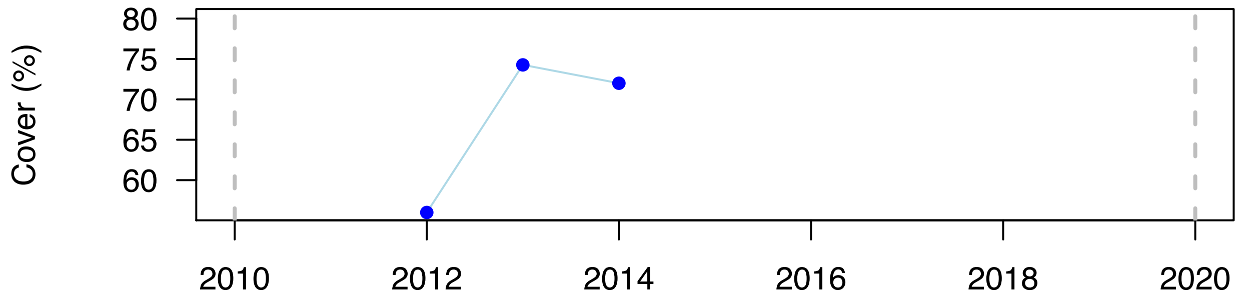

20_cover

Marbà et al. 2015

SITE: Pozo de Esparto (POS_03) (Spain – Mediterranean) – Po (–12.5 m)

OVERALL: Net = 7.47 %; Rate = 4.61 % yr–1; Perc Final = 110 % > no change

DECADAL: NO (2 yr)

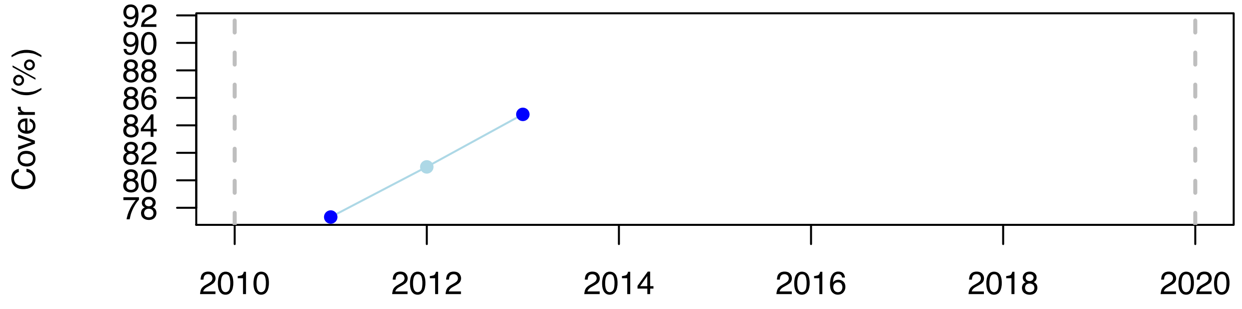

20_density

Marbà et al. 2015

SITE: Pozo de Esparto (POS_03) (Spain – Mediterranean) – Po (–12.5 m)

OVERALL: Net = –337.04 shoot m–2; Rate = –21.3 % yr–1; Perc Final = 53 % > decrease

DECADAL: NO (3 yr)

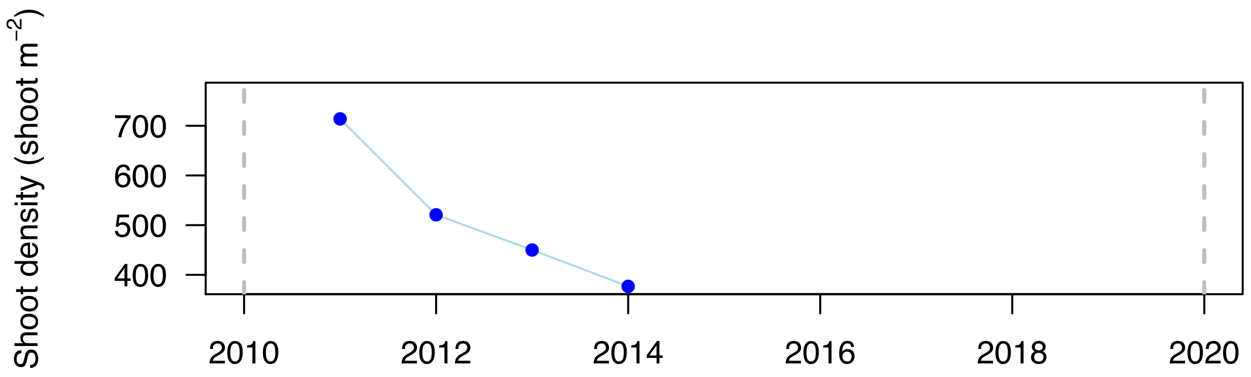

21_cover

Marbà et al. 2015

SITE: El Calón (POS_04) (Spain – Mediterranean) – Po (–12 m)

OVERALL: Net = –15.46 %; Rate = –6.66 % yr–1; Perc Final = 82 % > no change

DECADAL: NO (3 yr)

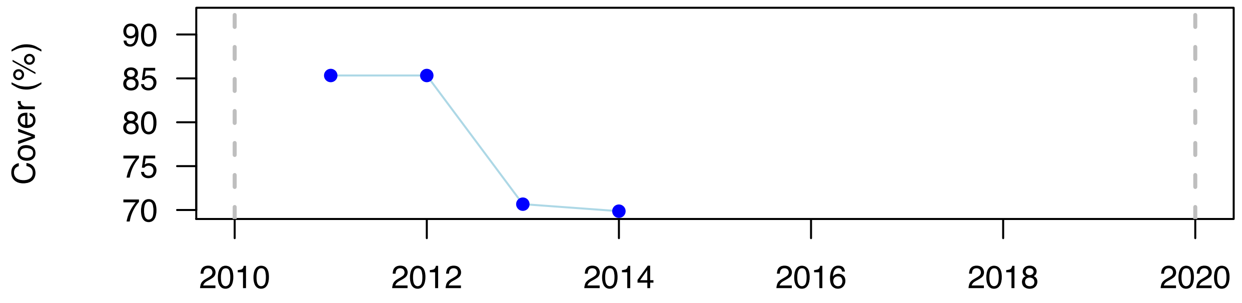

21_density
Marbà et al. 2015
SITE: El Calón (POS_04) (Spain – Mediterranean) – Po (–12 m)
OVERALL: Net = –59.37 shoot m–2;   Rate = –3.47 % yr–1; Perc Final = 90 % > no change
DECADAL: NO (3 yr)

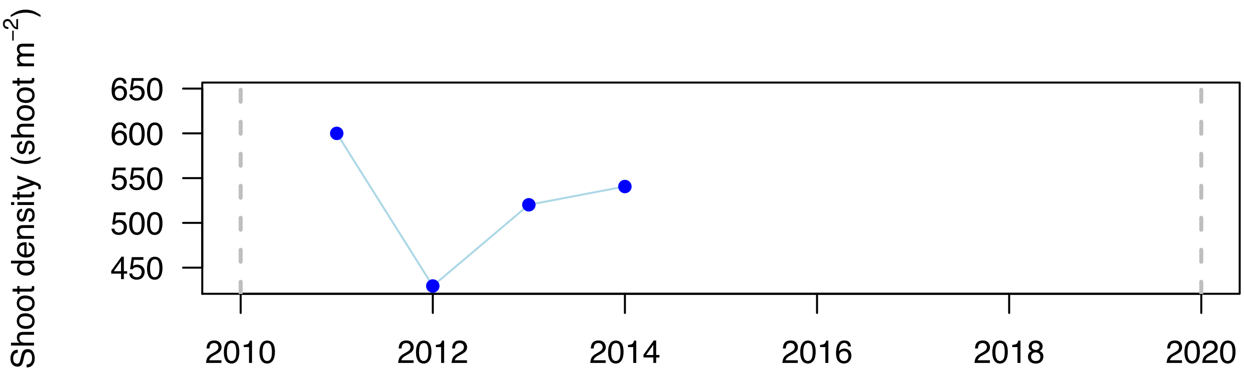

22_cover
Marbà et al. 2015
SITE: Loza del Payo (POS_05) (Spain – Mediterranean) – Po (–13 m)
OVERALL: Net = –11.07 %;   Rate = –4.79 % yr–1; Perc Final = 87 % > no change
DECADAL: NO (3 yr)

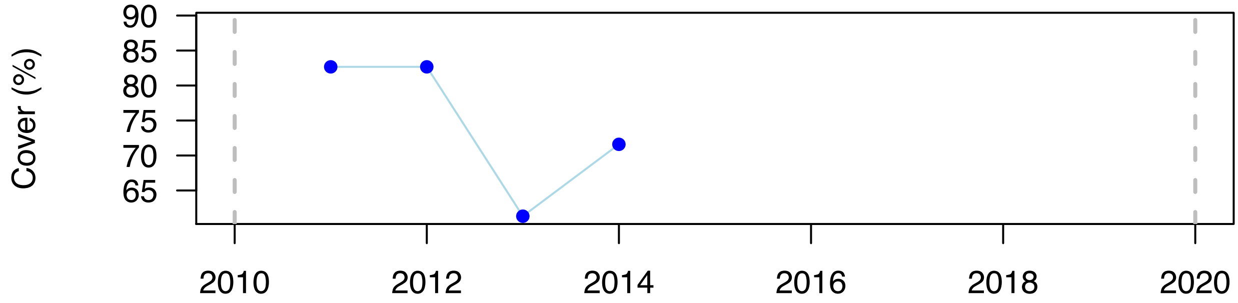

22_density
Marbà et al. 2015
SITE: Loza del Payo (POS_05) (Spain – Mediterranean) – Po (–13 m)
OVERALL: Net = 76.79 shoot m–2;   Rate = 4.76 % yr–1; Perc Final = 115 % > no change
DECADAL: NO (3 yr)

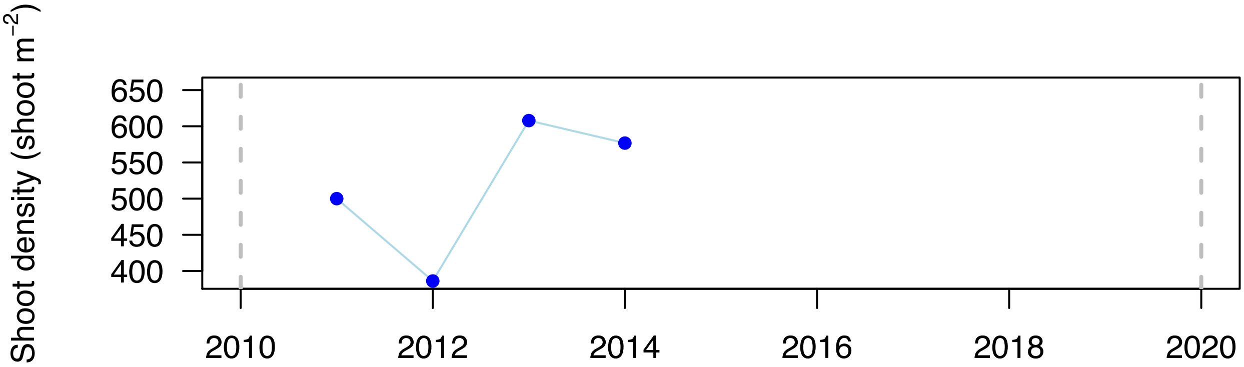

23_cover
Marbà et al. 2015
SITE: Deretil (POS_06) (Spain – Mediterranean) – Po (–12 m)
OVERALL: Net = 0.74 %;   Rate = 1.37 % yr–1; Perc Final = 103 % > no change
DECADAL: NO (2 yr)

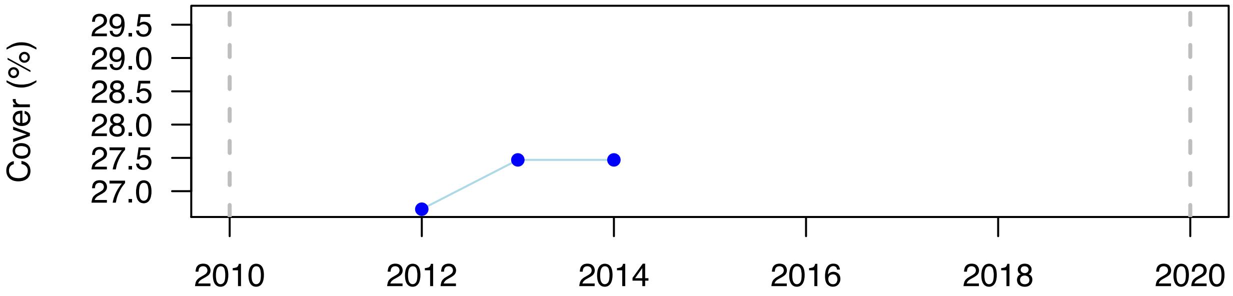

23_density

Marbà et al. 2015

SITE: Deretil (POS_06) (Spain – Mediterranean) – Po (–12 m)

OVERALL: Net = –95.59 shoot m–2;   Rate = –18.95 % yr–1; Perc Final = 68 % > decrease

DECADAL: NO (2 yr)

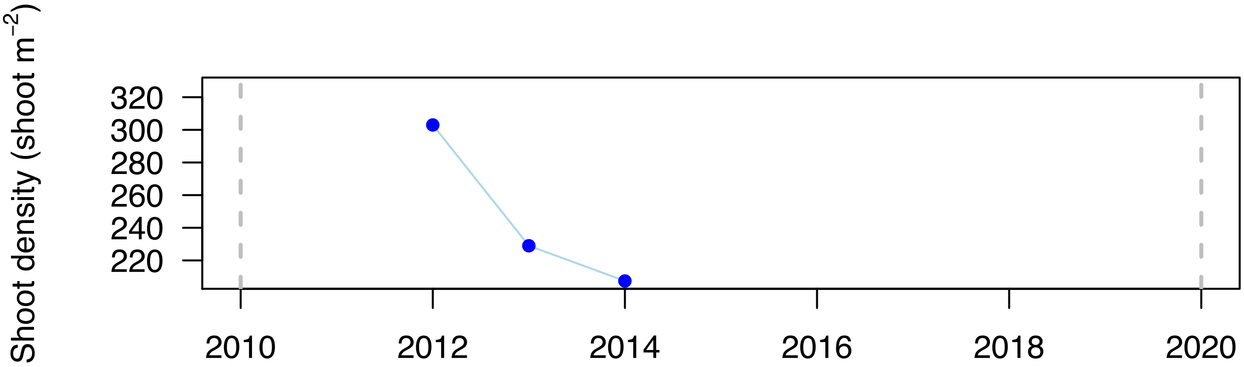

24_cover

Marbà et al. 2015

SITE: Isla de San Andrés (POS_07) (Spain – Mediterranean) – Po (–12 m)

OVERALL: Net = –5.33 %;   Rate = –5.06 % yr–1; Perc Final = 90 % > no change

DECADAL: NO (2 yr)

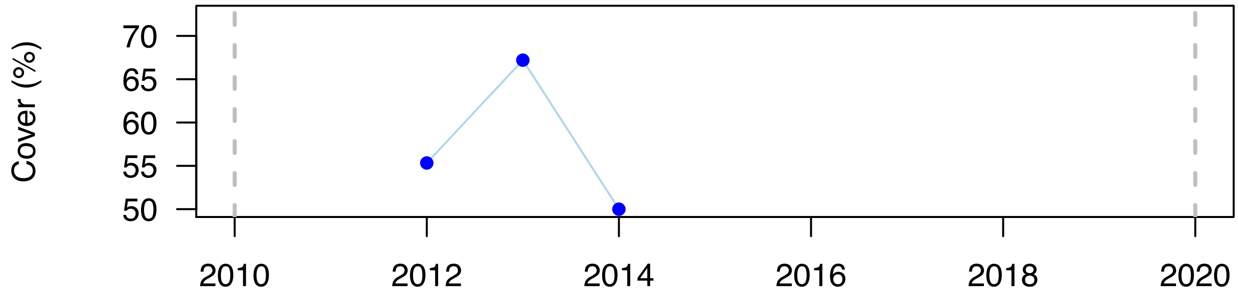

24_density

Marbà et al. 2015

SITE: Isla de San Andrés (POS_07) (Spain – Mediterranean) – Po (–12 m)

OVERALL: Net = –226.03 shoot m–2; Rate = –16.8 % yr–1; Perc Final = 71 % > decrease

DECADAL: NO (2 yr)

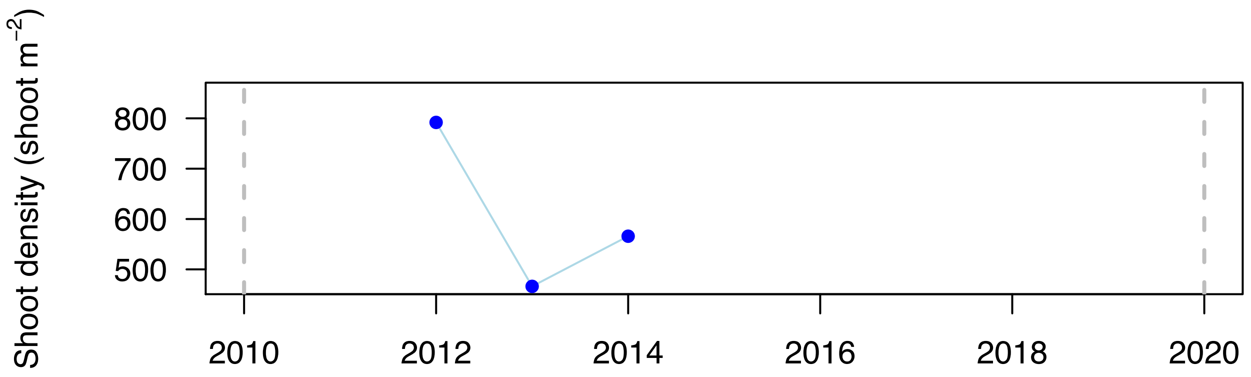

25_biomass

Boström et al. 2002

SITE: Hangö Peninsula (dense) (Finland – Baltic) – Zm (–4 m)

OVERALL: Net = 8.4 g dw m–2; Rate = 1.85 % yr–1; Perc Final = 159 % > increase

DECADAL: YES (25 yr)

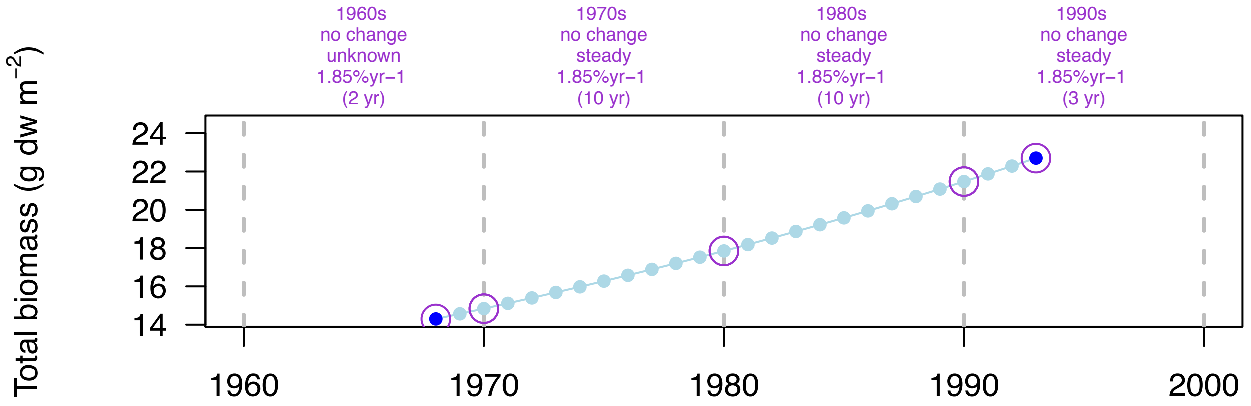

26_biomass

Boström et al. 2002

SITE: Hangö Peninsula (sparce) (Finland – Baltic) – Zm (–4 m)

OVERALL: Net = 2.8 g dw m–2;   Rate = 3.75 % yr–1; Perc Final = 256 % > increase

DECADAL: YES (25 yr)

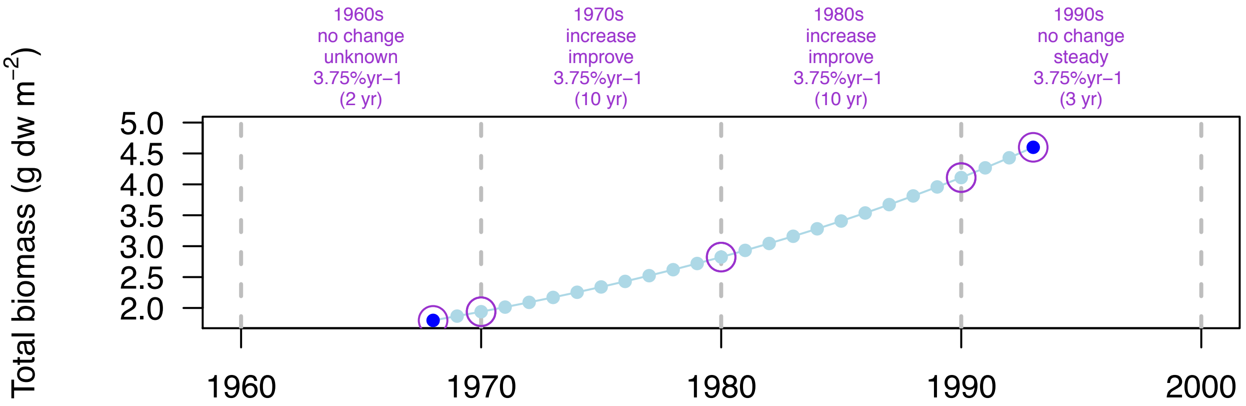

26_density

Boström et al. 2002

SITE: Hangö Peninsula (sparce) (Finland – Baltic) – Zm (–4 m)

OVERALL: Net = 11 shoot m–2;   Rate = 4.17 % yr–1; Perc Final = 283 % > increase

DECADAL: YES (25 yr)

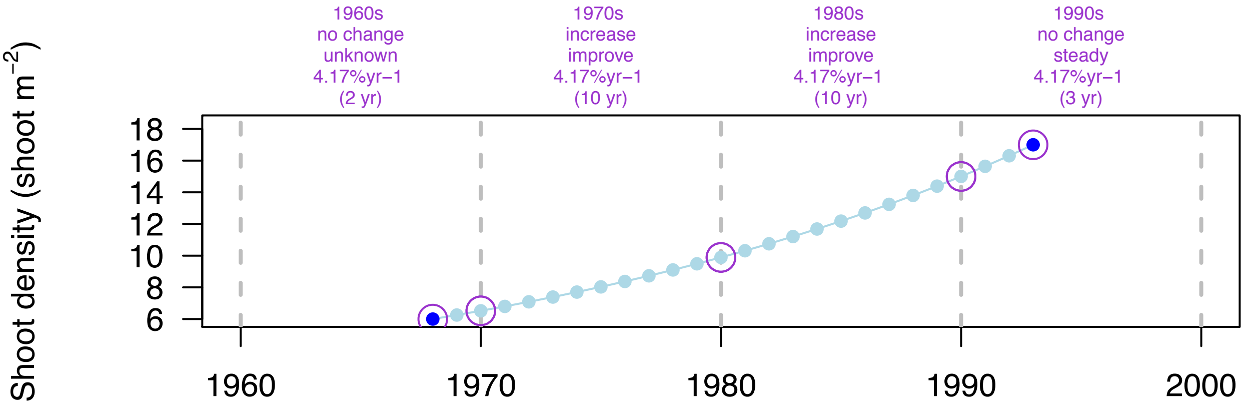

27_area

Bull et al. 2010

SITE: Gibraltar (United Kingdom – Mediterranean) – Zm (? m)

OVERALL: Net = –121.05 ha;   Rate = NA % yr–1; Perc Final = NA % > decrease

DECADAL: YES (15 yr)

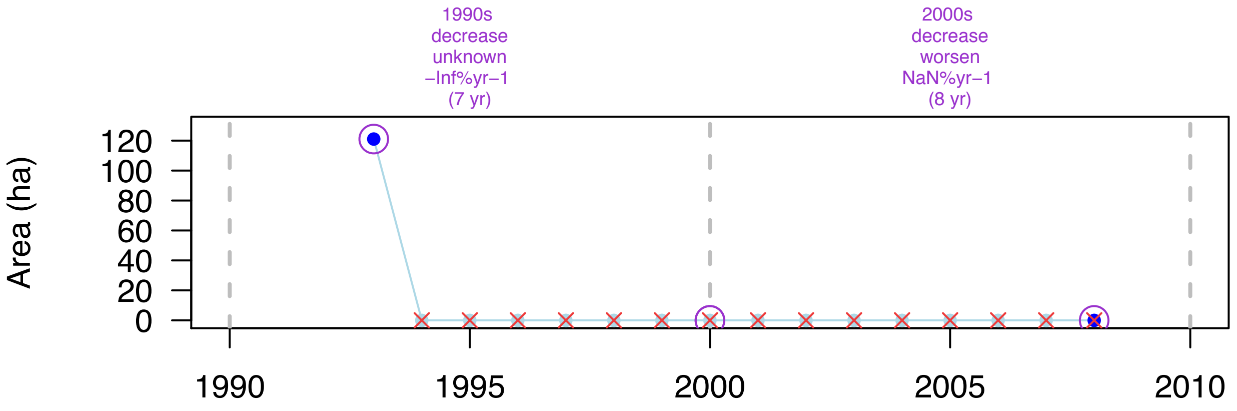

28_area

Bull et al. 2010

SITE: Gibraltar (United Kingdom – Mediterranean) – Po (? m)

OVERALL: Net = –152.41 ha;   Rate = NA % yr–1; Perc Final = NA % > decrease

DECADAL: YES (15 yr)

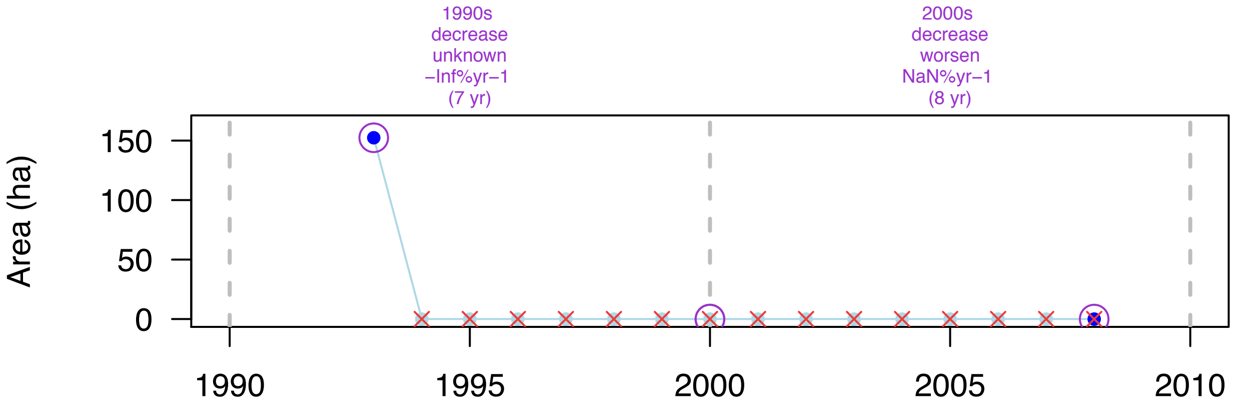

29_density

Bull et al. 2012

SITE: Broad Ledges (United Kingdom – Atlantic) – Zm (–0.2 m)

OVERALL: Net = –13.3 shoot m–2; Rate = –1.32 % yr–1; Perc Final = 84 % > no change

DECADAL: YES (13 yr)

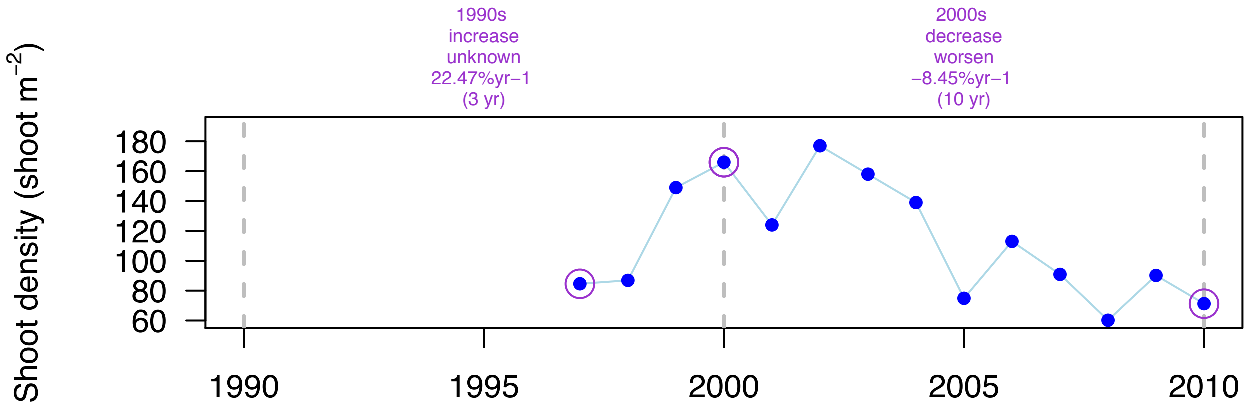

30_density

Bull et al. 2012

SITE: Higher Town Bay (United Kingdom – Atlantic) – Zm (0.5 m)

OVERALL: Net = –27 shoot m–2; Rate = –1.24 % yr–1; Perc Final = 85 % > no change

DECADAL: YES (13 yr)

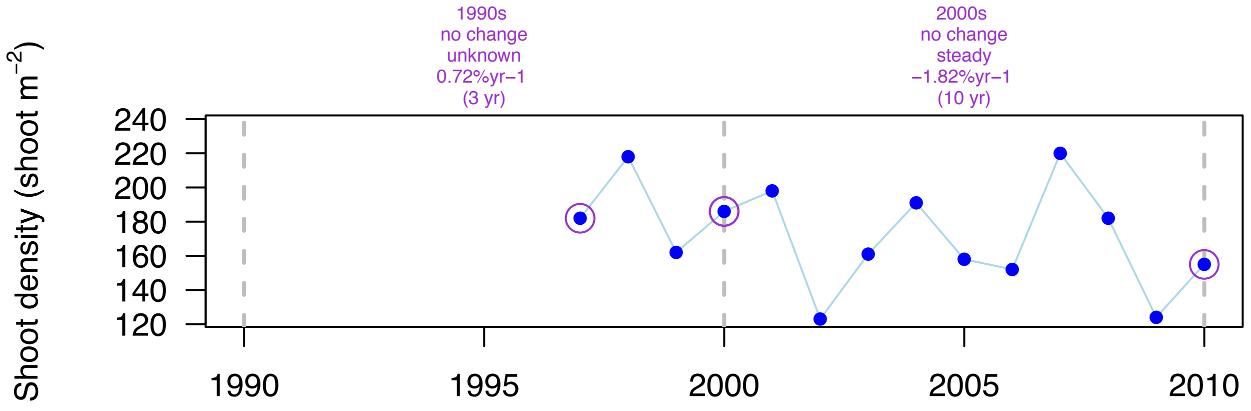

31_density

Bull et al. 2012

SITE: Little Arthur (United Kingdom – Atlantic) – Zm (–1 m)

OVERALL: Net = 33 shoot m–2;   Rate = 1.87 % yr–1; Perc Final = 125 % > increase

DECADAL: YES (12 yr)

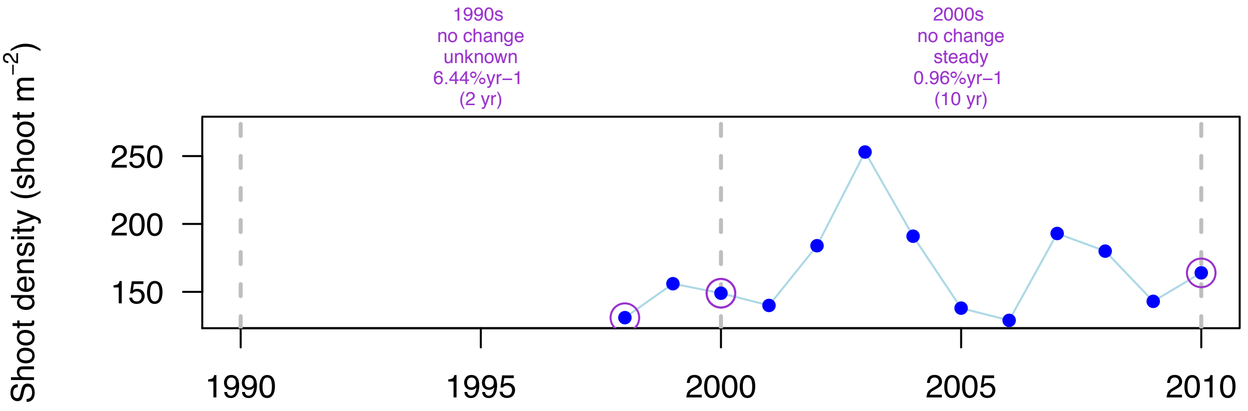

32_density

Bull et al. 2012

SITE: Old Grimsby Harbour (United Kingdom – Atlantic) – Zm (–0.6 m)

OVERALL: Net = –10 shoot m–2;   Rate = –0.68 % yr–1; Perc Final = 92 % > no change

DECADAL: YES (13 yr)

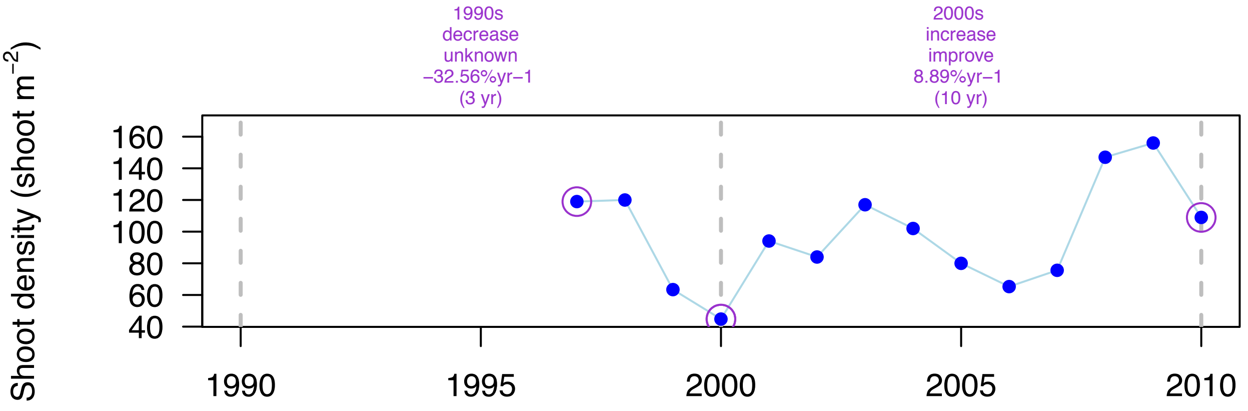

33_density

Bull et al. 2012

SITE: West Broad Ledges (United Kingdom – Atlantic) – Zm (–0.6 m)

OVERALL: Net = –15 shoot m–2;   Rate = –0.91 % yr–1; Perc Final = 89 % > no change

DECADAL: YES (13 yr)

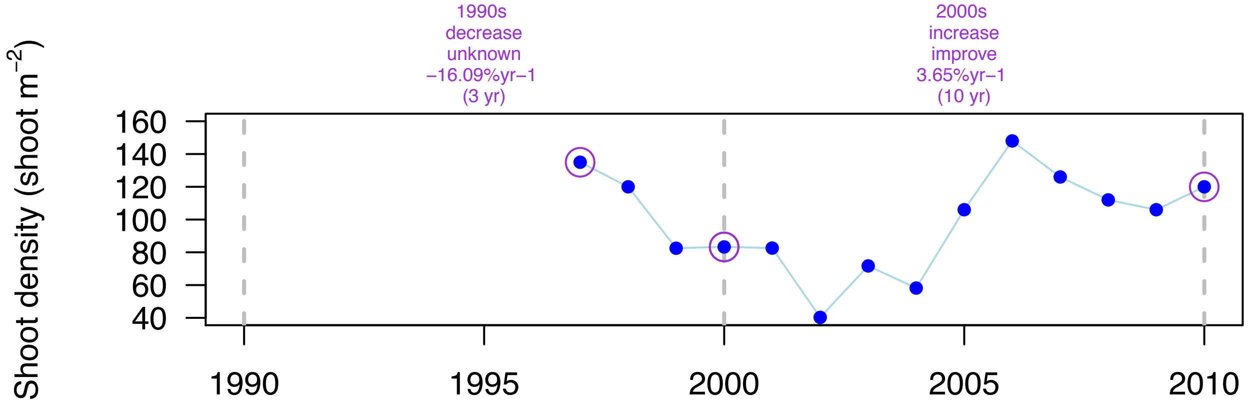

34_area

Burton et al. 2010

SITE: North Haven (Skomer) (United Kingdom – Atlantic) – Zm (? m)

OVERALL: Net = 0.25 ha;   Rate = 1.34 % yr–1; Perc Final = 145 % > increase

DECADAL: YES (28 yr)

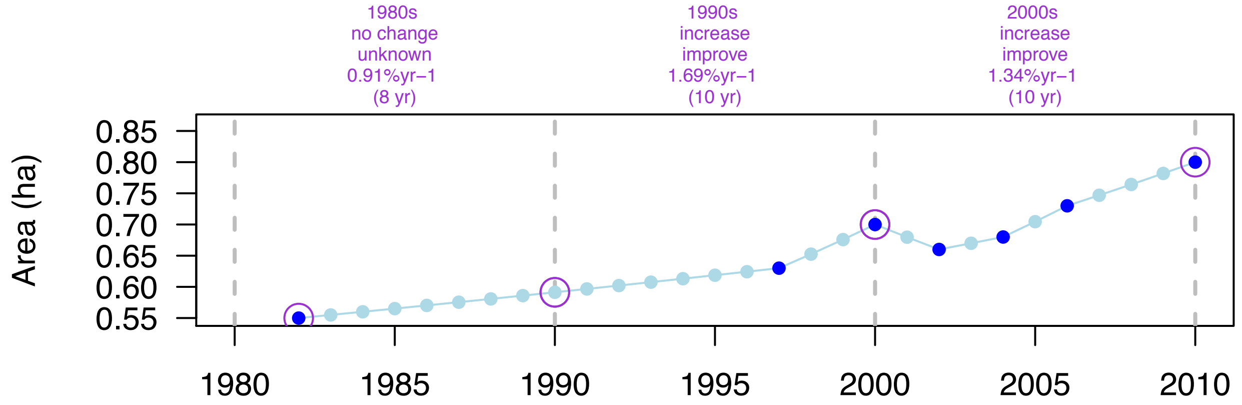

34_density

Burton et al. 2010

SITE: North Haven (Skomer) (United Kingdom – Atlantic) – Zm (? m)

OVERALL: Net = 4.2 shoot m–2;   Rate = 0.84 % yr–1; Perc Final = 112 % > no change

DECADAL: YES (13 yr)

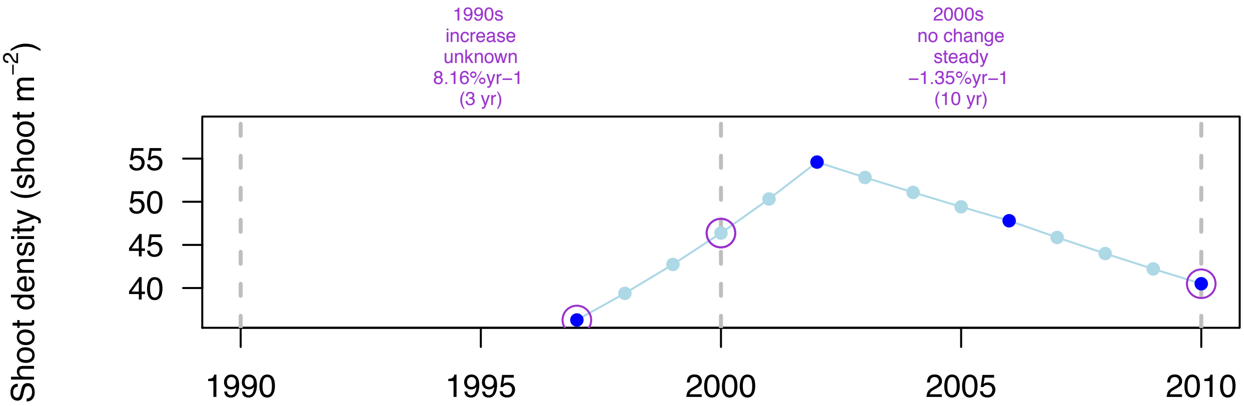

35_area

Rismondo and Mion 2008

SITE: Venice Lagoon (Italy – Mediterranean) – Zm (–1 m)

OVERALL: Net = –840 ha;   Rate = –37.43 % yr–1; Perc Final = 47 % > decrease

DECADAL: NO (2 yr)

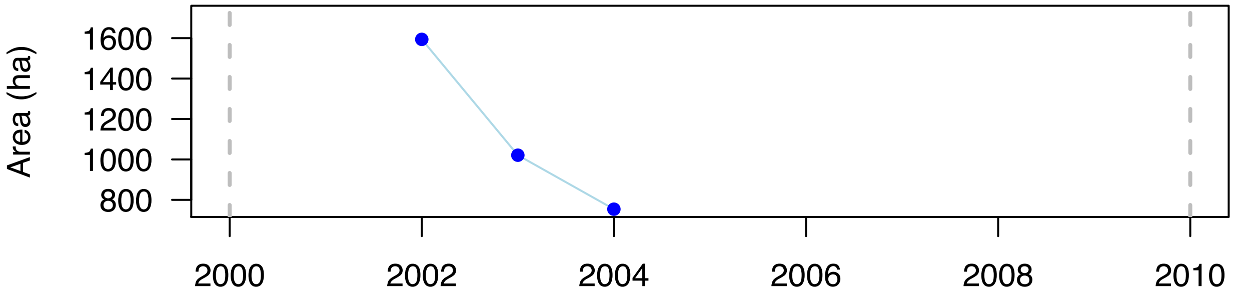

37_lowerlimit

Rask et al. 1999

SITE: South Funen Archipelago (Denmark – Baltic) – Zm (? m)

OVERALL: Net = 1.5 m;   Rate = 1.31 % yr–1; Perc Final = 125 % > increase

DECADAL: YES (17 yr)

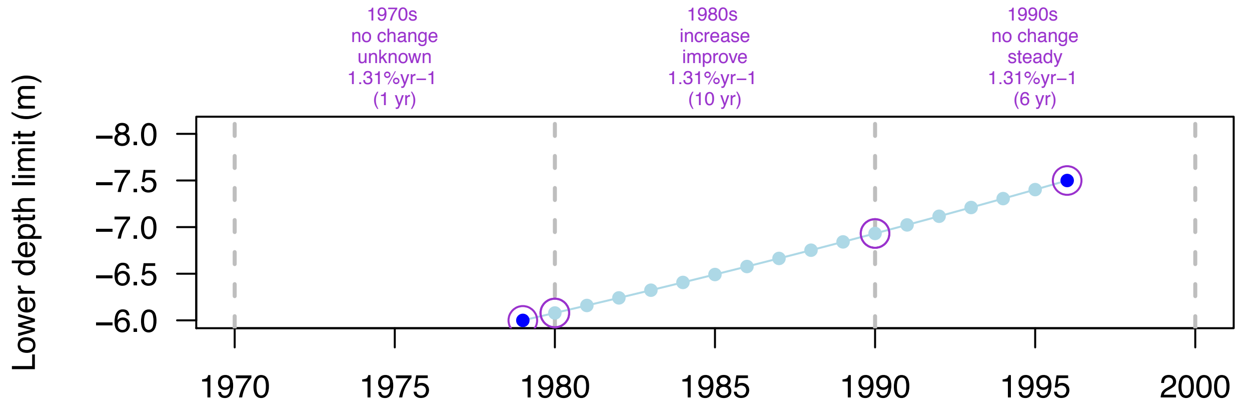

45_biomass

Plus et al. 2003

SITE: Thau Lagoon (France – Mediterranean) – Zm (–4.2 m)

OVERALL: Net = 29.8 g dw m–2;   Rate = NA % yr–1; Perc Final = NA % > increase

DECADAL: NO (2 yr)

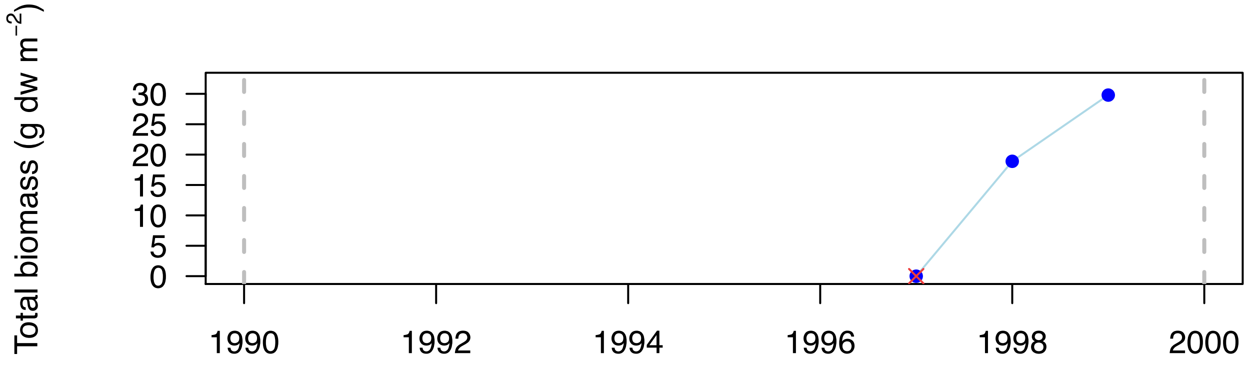

45_density

Plus et al. 2003

SITE: Thau Lagoon (France – Mediterranean) – Zm (–4.2 m)

OVERALL: Net = 107.41 shoot m–2; Rate = NA % yr–1; Perc Final = NA % > increase

DECADAL: NO (2 yr)

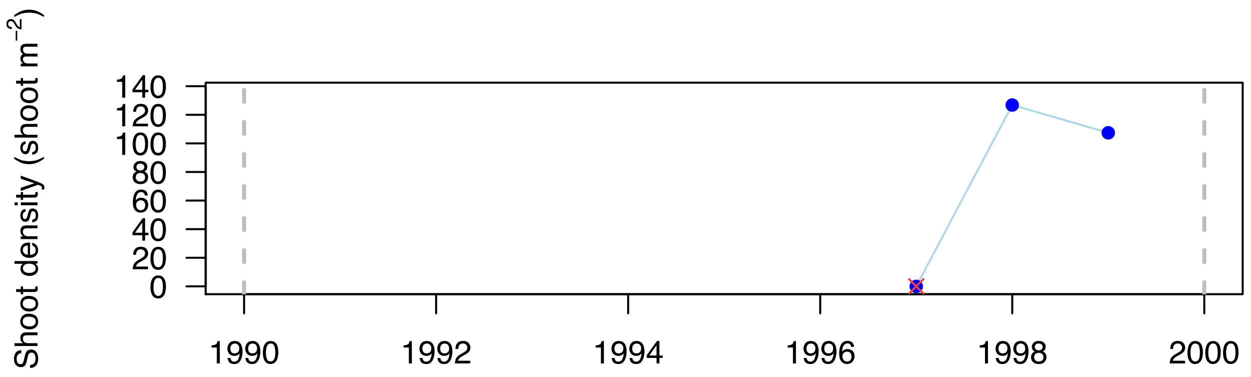

46_area

Ballesta et al. 2000

SITE: Pin Parasol Cove (France – Mediterranean) – Po (? m)

OVERALL: Net = –0.01 ha; Rate = –8.66 % yr–1; Perc Final = 50 % > decrease

DECADAL: YES (8 yr)

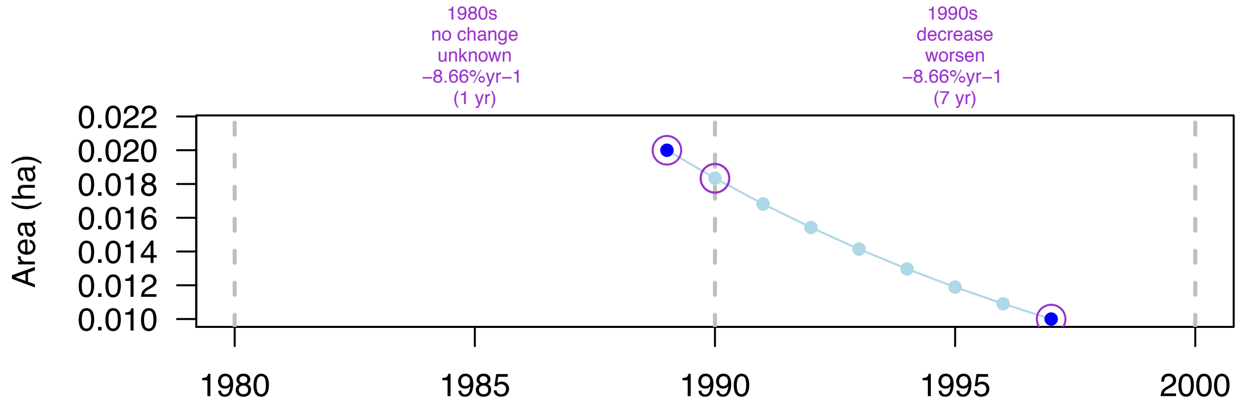

47_area

Rismondo and Mion 2008

SITE: Venice Lagoon (Italy – Mediterranean) – Cn (? m)

OVERALL: Net = –82 ha;   Rate = –41.68 % yr–1; Perc Final = 43 % > decrease

DECADAL: NO (2 yr)

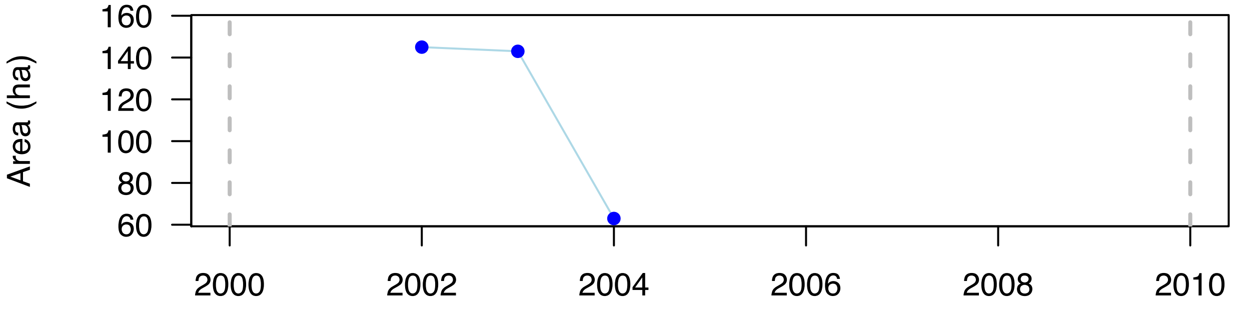

48_area

Charpentier et al. 2005

SITE: Vaccarès Lagoon (France – Mediterranean) – Zn (? m)

OVERALL: Net = –869.5 ha;   Rate = –2.02 % yr–1; Perc Final = 82 % > decrease

DECADAL: YES (10 yr)

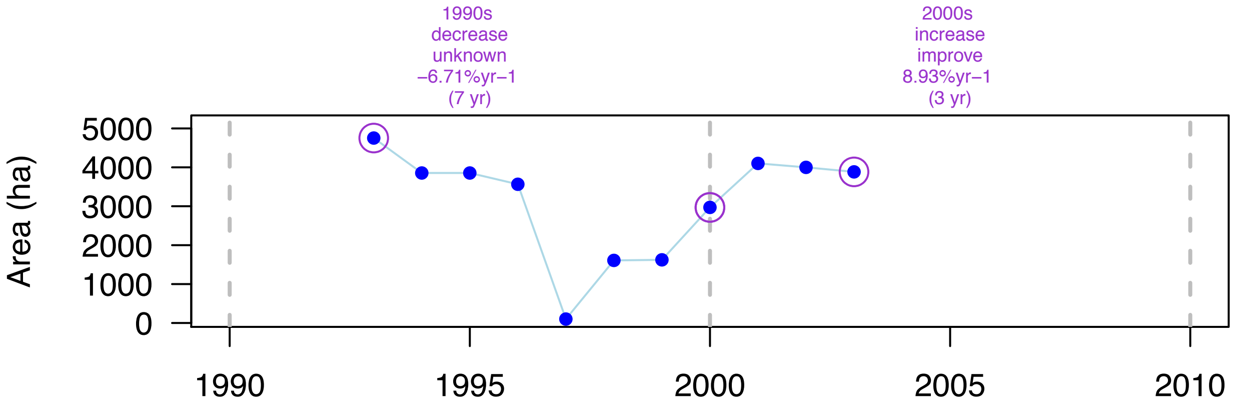

48_lowerlimit

Charpentier et al. 2005

SITE: Vaccarès Lagoon (France – Mediterranean) – Zn (? m)

OVERALL: Net = 0.14 m;   Rate = 1.17 % yr–1; Perc Final = 109 % > no change

DECADAL: NO (7 yr)

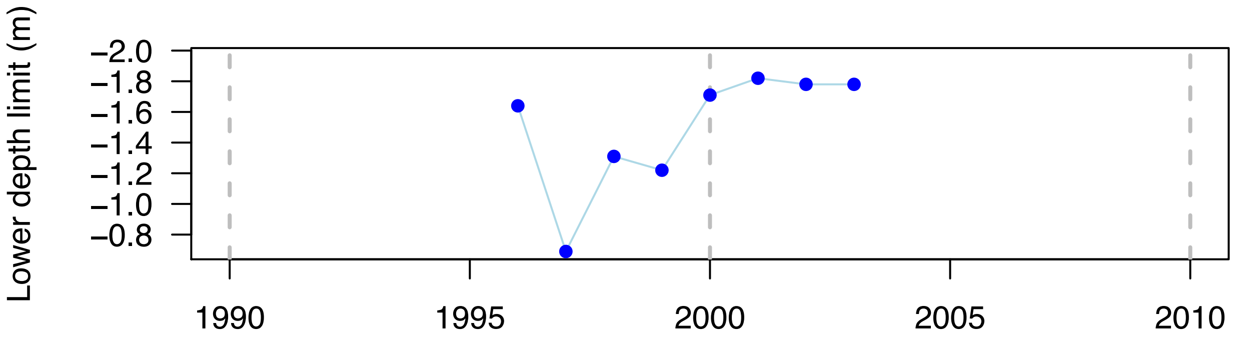

49_area

Nyqvist et al. 2009, Baden et al. 2003

SITE: Kungälv (Sweden – Baltic) – Zm (? m)

OVERALL: Net = –557.03 ha;   Rate = –8.08 % yr–1; Perc Final = 14 % > decrease

DECADAL: YES (24 yr)

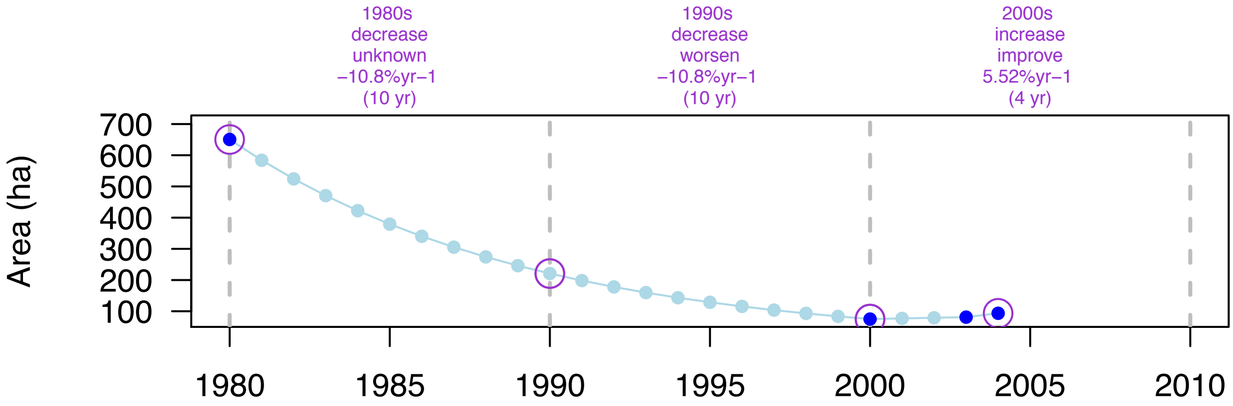

50_area

Nyqvist et al. 2009

SITE: Lysekil (Sweden – Baltic) – Zm (? m)

OVERALL: Net = –27.1 ha;   Rate = –0.86 % yr–1; Perc Final = 81 % > decrease

DECADAL: YES (24 yr)

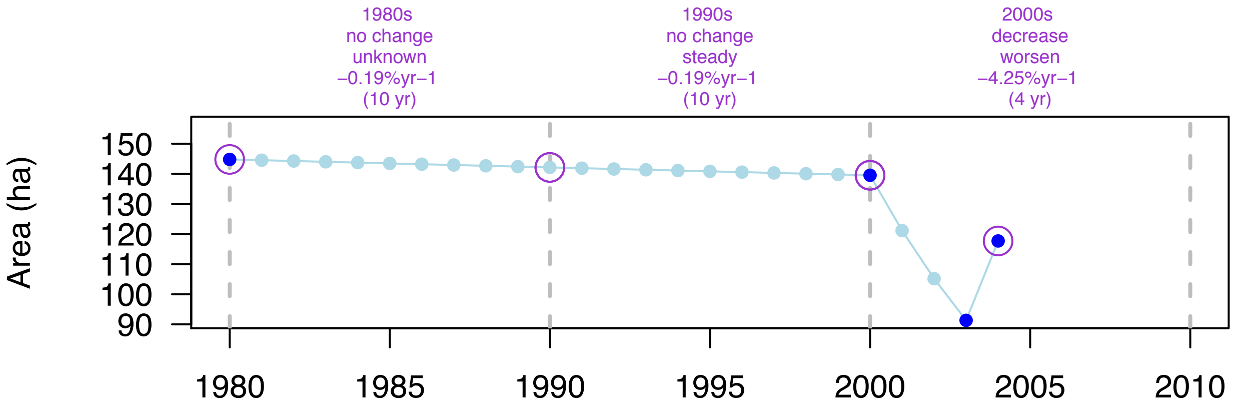

51_area

Nyqvist et al. 2009

SITE: Stenungsund (Sweden – Baltic) – Zm (? m)

OVERALL: Net = –50.71 ha;   Rate = –0.79 % yr–1; Perc Final = 83 % > decrease

DECADAL: YES (24 yr)

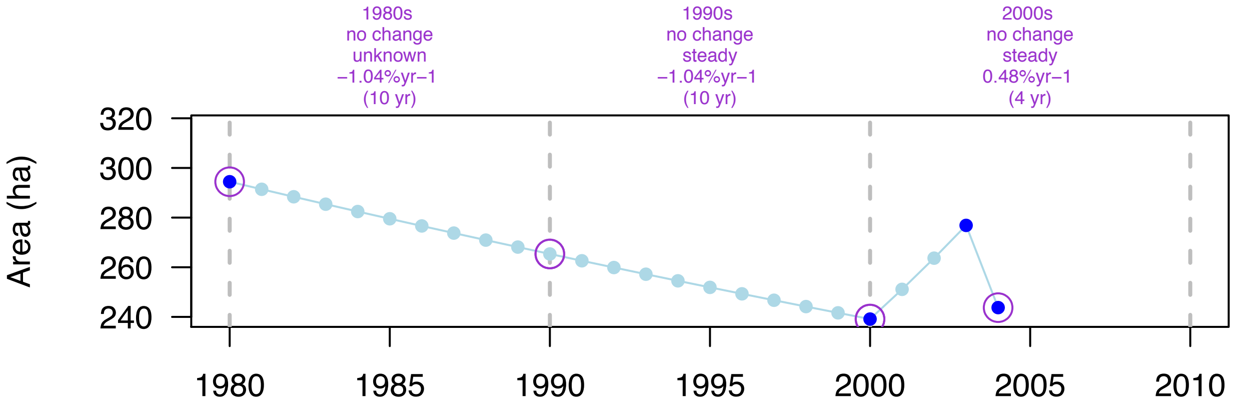

52_area

Nyqvist et al. 2009

SITE: Strömstad (Sweden – Baltic) – Zm (? m)

OVERALL: Net = –72.81 ha; Rate = –2.01 % yr–1; Perc Final = 62 % > decrease

DECADAL: YES (24 yr)

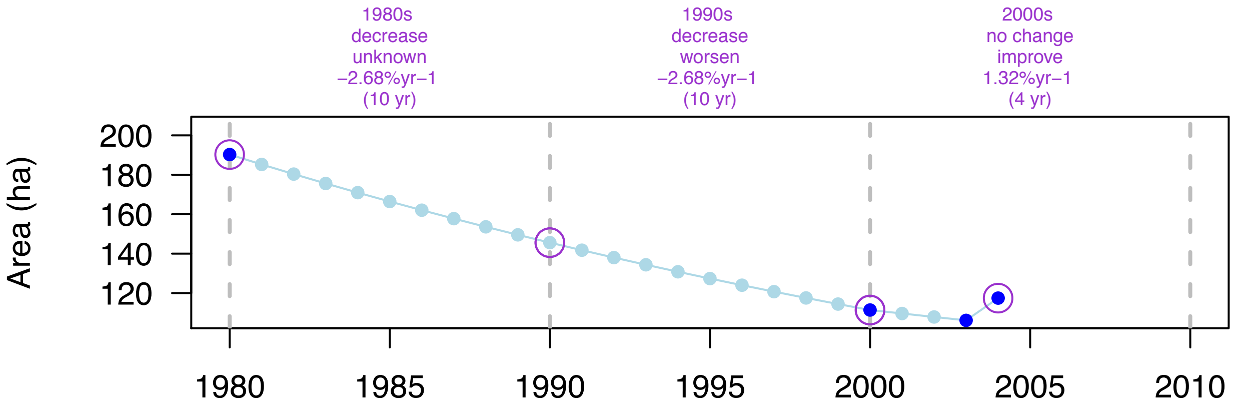

53_area

Nyqvist et al. 2009

SITE: Uddevalla (Sweden – Baltic) – Zm (? m)

OVERALL: Net = –255.06 ha; Rate = –4.48 % yr–1; Perc Final = 34 % > decrease

DECADAL: YES (24 yr)

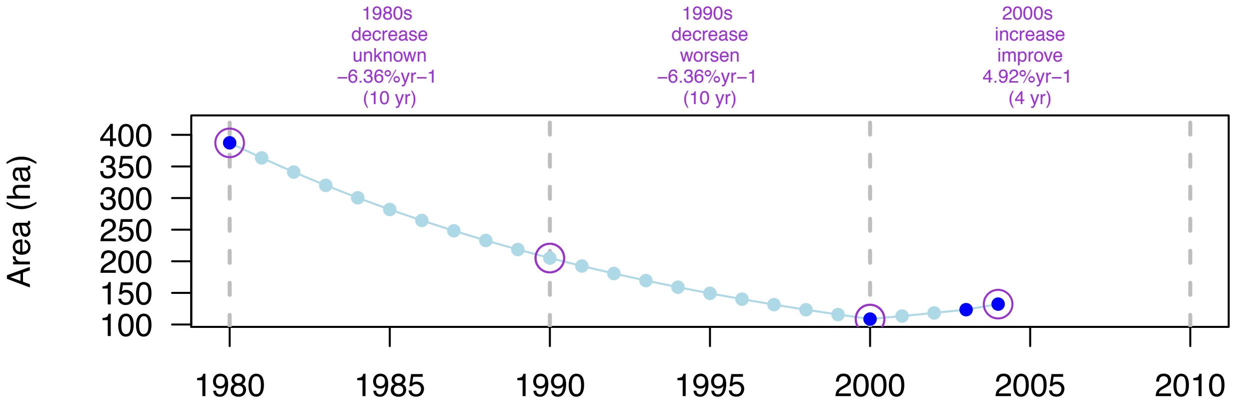

54_area

Garrido et al. 2013

SITE: Urbino Lagoon (France – Mediterranean) – Cn (? m)

OVERALL: Net = –22 ha; Rate = –0.27 % yr–1; Perc Final = 90 % > no change

DECADAL: YES (38 yr)

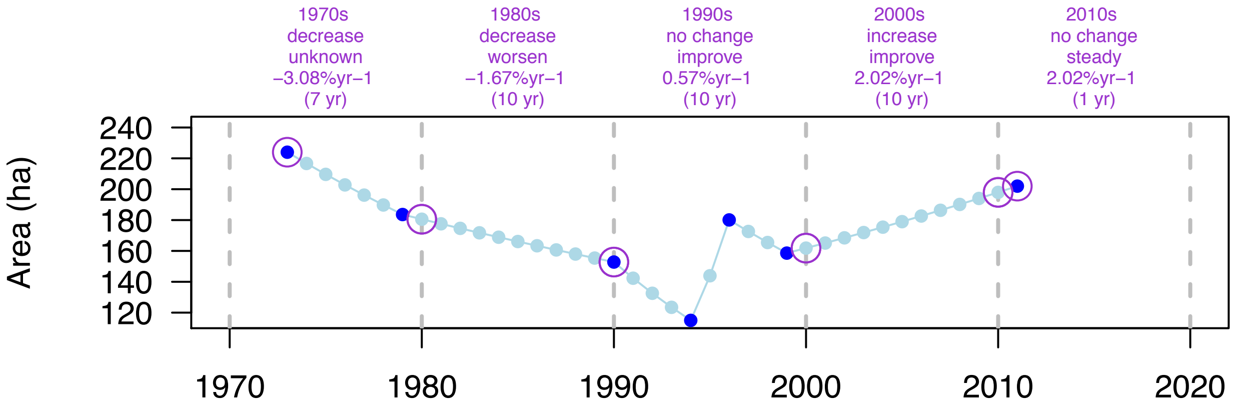

55_area

Martínez–Samper et al. 2011

SITE: Bahía de Confital (Spain – Atlantic) – Cn (? m)

OVERALL: Net = –10.45 ha; Rate = NA % yr–1; Perc Final = NA % > decrease

DECADAL: YES (22 yr)

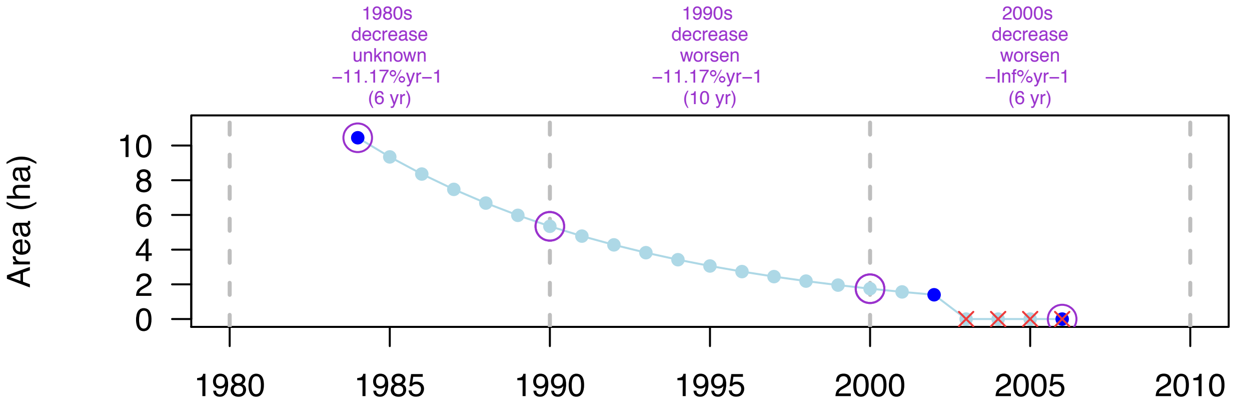

56_area

Martínez–Samper et al. 2011

SITE: Franja Las Palmas de Gran Canaria (Spain – Atlantic) – Cn (–16 m)

OVERALL: Net = –11.89 ha;   Rate = NA % yr–1; Perc Final = NA % > decrease

DECADAL: NO (5 yr)

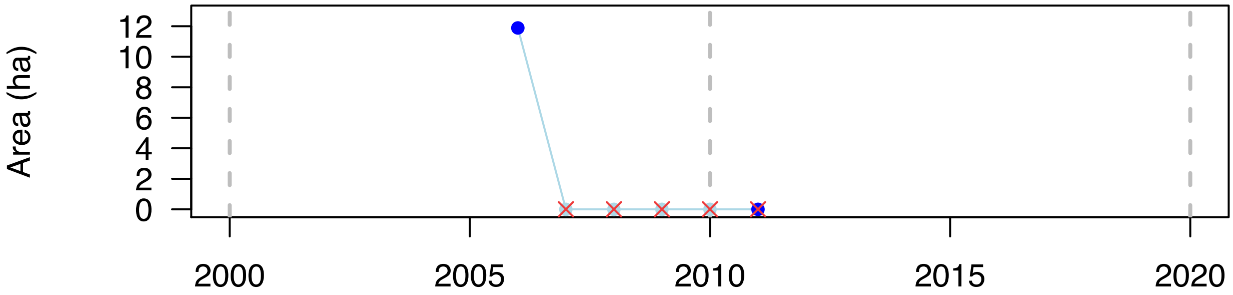

57_area

Martínez–Samper et al. 2011

SITE: Franja Marina del Telde (Spain – Atlantic) – Cn (–11.7 m)

OVERALL: Net = –25.25 ha;   Rate = –20.5 % yr–1; Perc Final = 0 % > decrease

DECADAL: YES (27 yr)

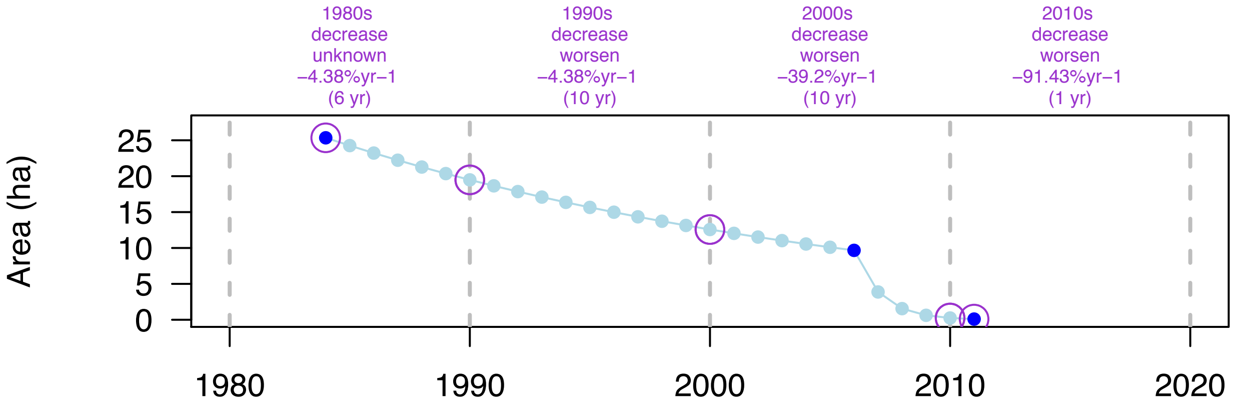

58_area

Martínez–Samper et al. 2011

SITE: Bahía de Gando (Spain – Atlantic) – Cn (–7.2 m)

OVERALL: Net = –66.1 ha;   Rate = NA % yr–1;   Perc Final = NA % > decrease

DECADAL: YES (27 yr)

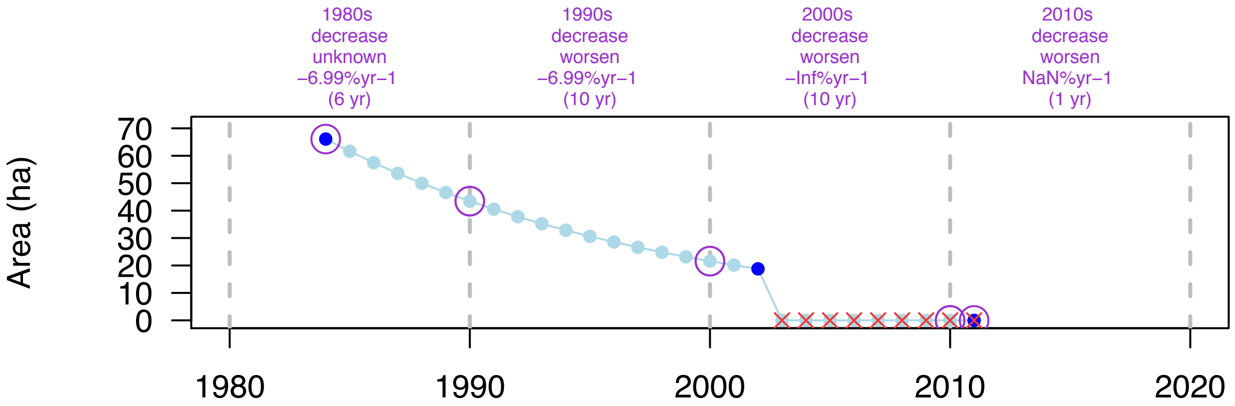

59_area

Martínez–Samper et al. 2011

SITE: Playa de Vargas (Spain – Atlantic) – Cn (–12.9 m)

OVERALL: Net = –24.73 ha;   Rate = –49.53 % yr–1;   Perc Final = 1 % > decrease

DECADAL: YES (9 yr)

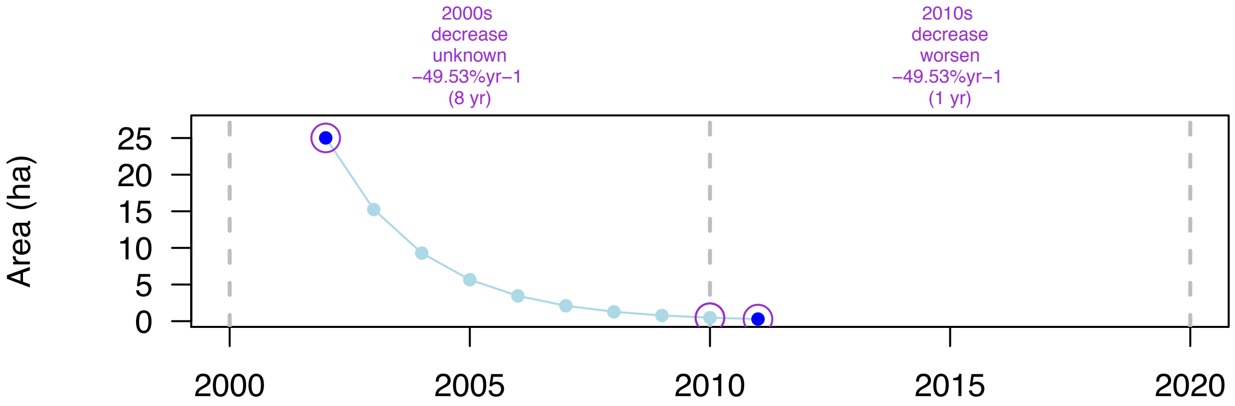

60_area Martínez–Samper et al. 2011

SITE: Playa del Cabrón (Spain – Atlantic) – Cn (–9 m)

OVERALL: Net = 0.19 ha; Rate = 0.08 % yr–1; Perc Final = 102 % > no change

DECADAL: YES (27 yr)

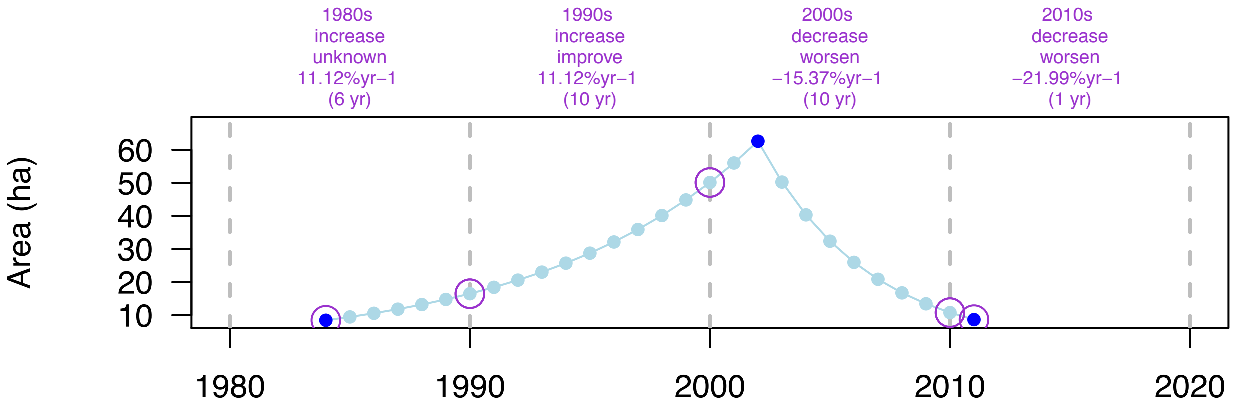

61_area Martínez–Samper et al. 2011

SITE: Arinaga (Spain – Atlantic) – Cn (–6.6 m)

OVERALL: Net = –66.4 ha; Rate = –20.4 % yr–1; Perc Final = 0 % > decrease

DECADAL: YES (27 yr)

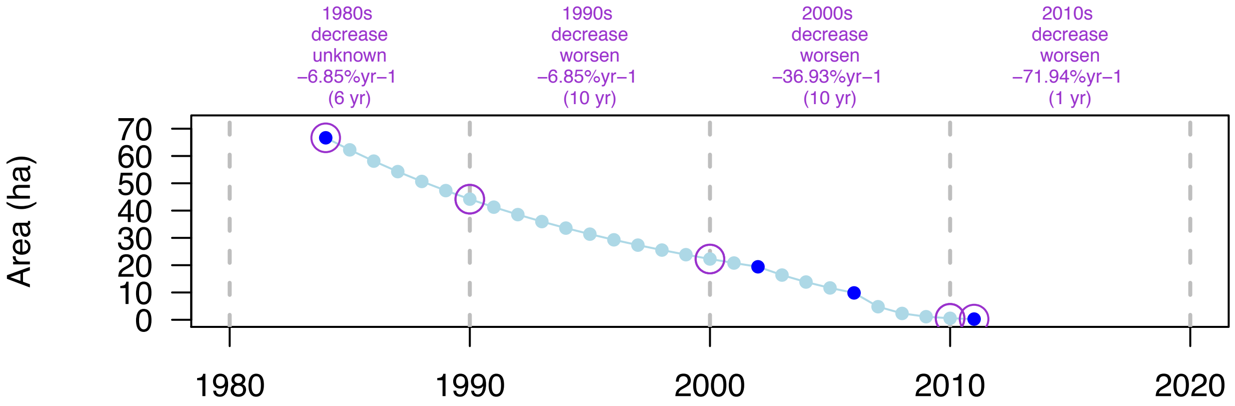

62_area

Martínez–Samper et al. 2011

SITE: Juan Grande (Spain – Atlantic) – Cn (–11.3 m)

OVERALL: Net = –6.85 ha;   Rate = –42 % yr–1; Perc Final = 2 % > decrease

DECADAL: YES (9 yr)

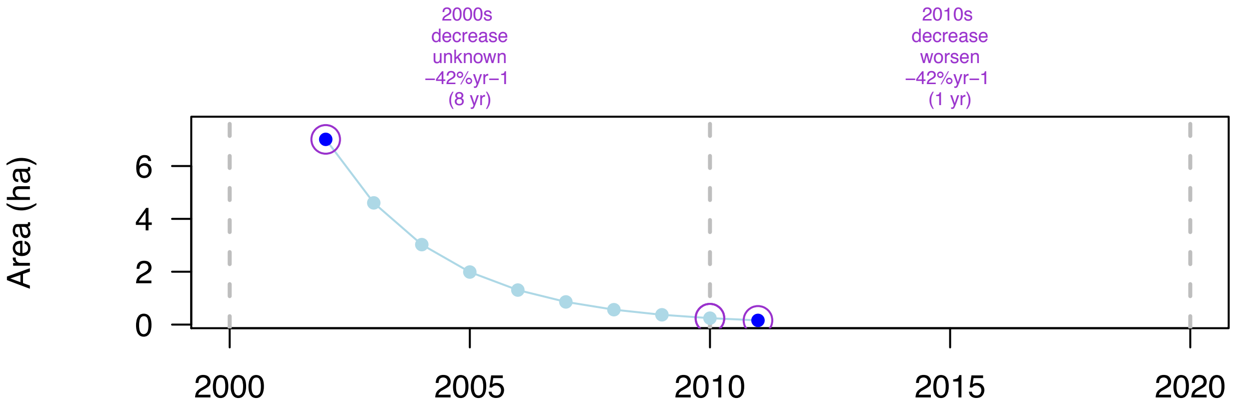

63_area

Martínez–Samper et al. 2011

SITE: Juncalillo del Sur (Spain – Atlantic) – Cn (–6.8 m)

OVERALL: Net = –23.11 ha;   Rate = –4.58 % yr–1; Perc Final = 66 % > decrease

DECADAL: YES (9 yr)

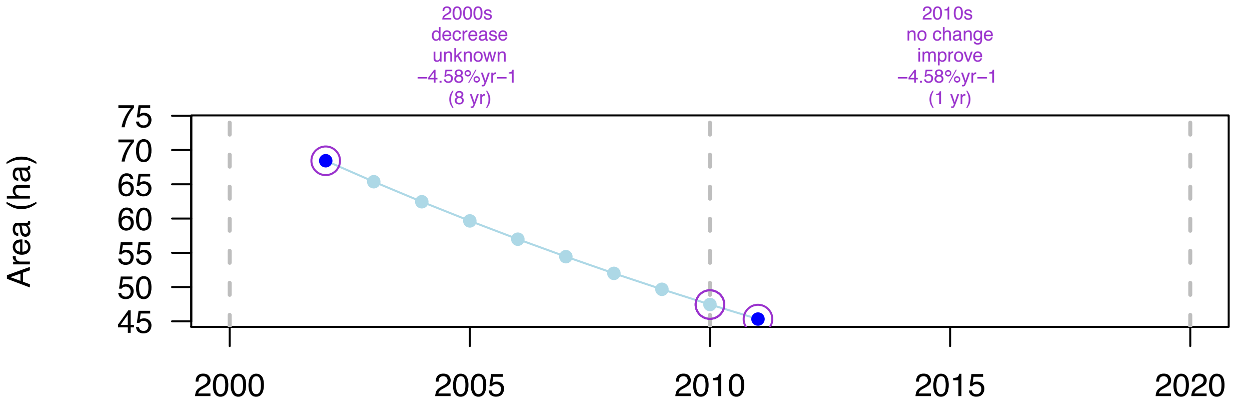

64_area

Martínez–Samper et al. 2011

SITE: Playa del Inglés (Spain – Atlantic) – Cn (–5.3 m)

OVERALL: Net = –43.57 ha;   Rate = –3.18 % yr–1; Perc Final = 42 % > decrease

DECADAL: YES (27 yr)

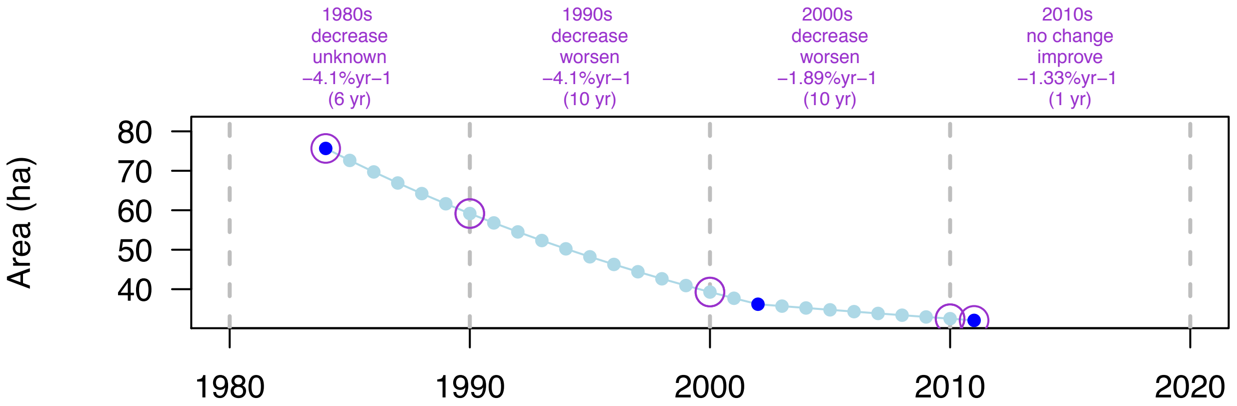

65_area

Martínez–Samper et al. 2011

SITE: Bahía Feliz – San Agustín (Spain – Atlantic) – Cn (–4.6 m)

OVERALL: Net = 4.53 ha;   Rate = 0.88 % yr–1; Perc Final = 127 % > increase

DECADAL: YES (27 yr)

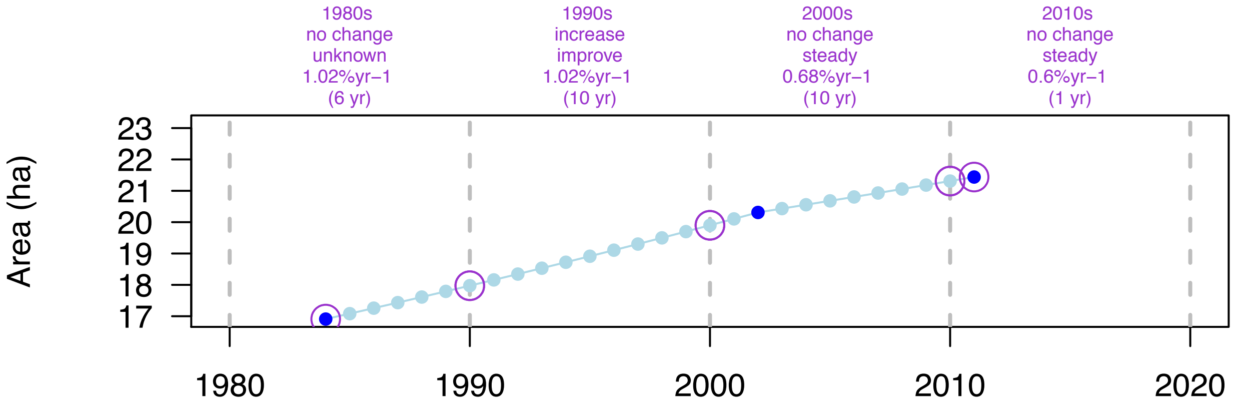

66_area

Martínez–Samper et al. 2011

SITE: Franja Marina de Mogán (Spain – Atlantic) – Cn (–5.5 m)

OVERALL: Net = –50.67 ha; Rate = –2.13 % yr–1; Perc Final = 56 % > decrease

DECADAL: YES (27 yr)

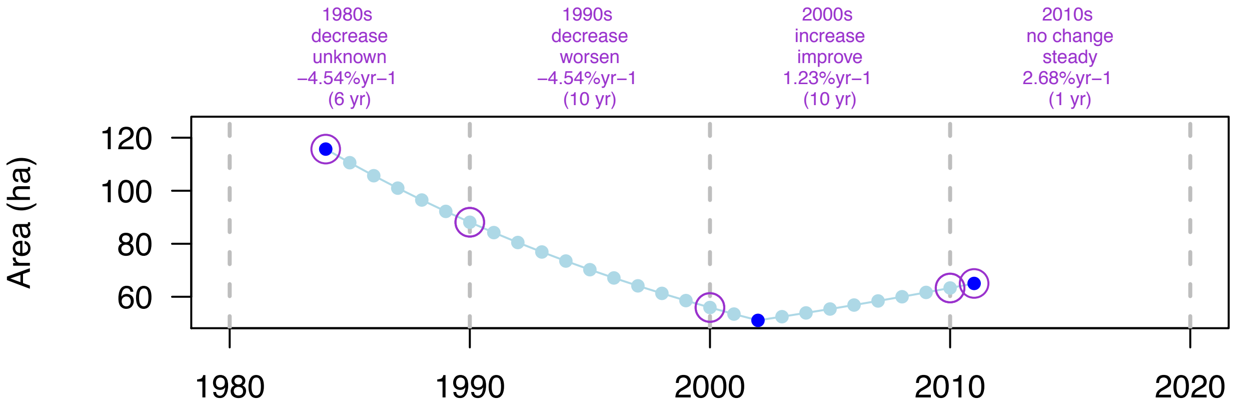

67_area

Boudouresque et al. 2006

SITE: Mourillion (France – Mediterranean) – Po (? m)

OVERALL: Net = –69 ha; Rate = –4.22 % yr–1; Perc Final = 53 % > decrease

DECADAL: YES (15 yr)

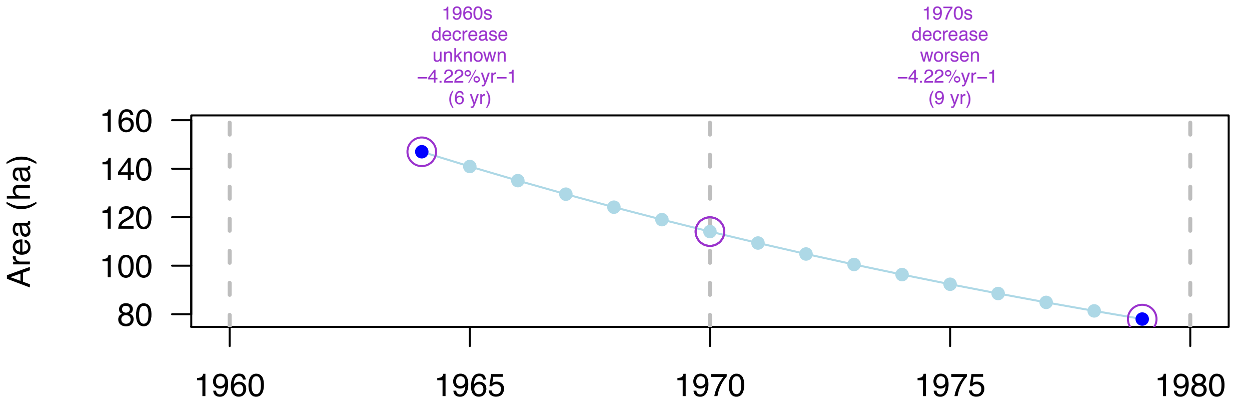

68_area

Philippart and Dijkema 1995

SITE: De Keeg – De Ans (The Netherlands – Atlantic) – Zn (? m)

OVERALL: Net = 8 ha; Rate = 2.67 % yr–1; Perc Final = 153 % > increase

DECADAL: YES (16 yr)

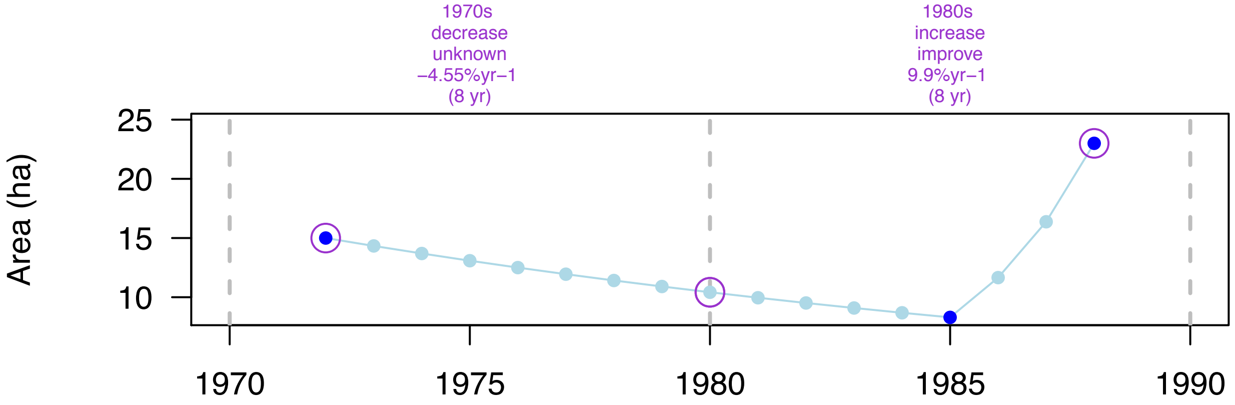

69_area

Philippart and Dijkema 1995

SITE: Emmapolder (The Netherlands – Atlantic) – Zn (? m)

OVERALL: Net = 77 ha; Rate = NA % yr–1; Perc Final = NA % > increase

DECADAL: YES (15 yr)

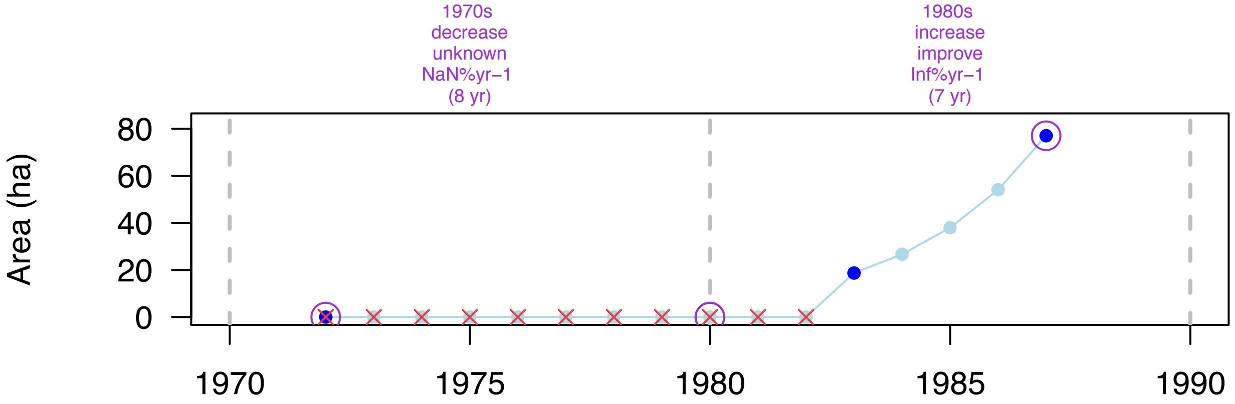

71_area

Philippart and Dijkema 1995

SITE: Linthorst Homanpolder (The Netherlands – Atlantic) – Zn (? m)

OVERALL: Net = 75 ha; Rate = NA % yr–1; Perc Final = NA % > increase

DECADAL: YES (16 yr)

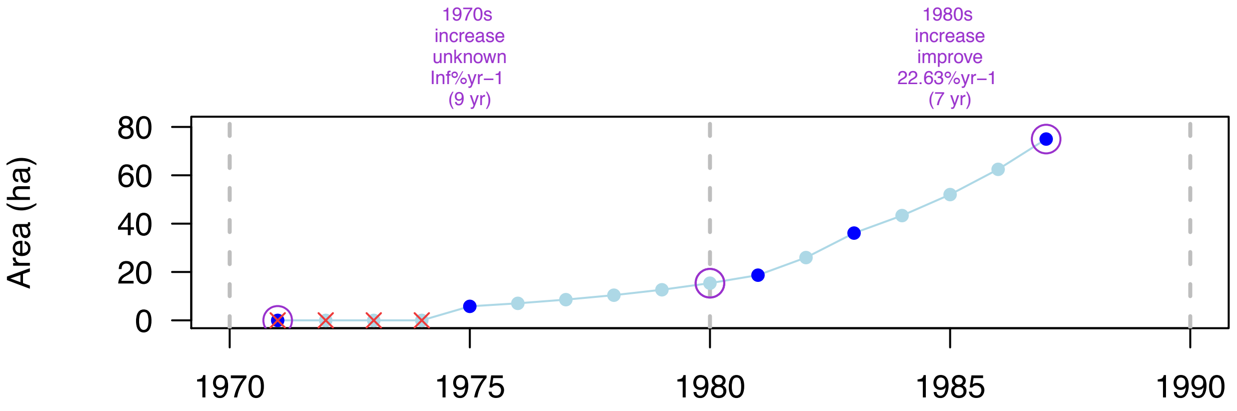

76_area

Portig 2006

SITE: Lough Foyle (United Kingdom – Atlantic) – Zm (? m)

OVERALL: Net = –189 ha; Rate = –5.33 % yr–1; Perc Final = 53 % > decrease

DECADAL: YES (12 yr)

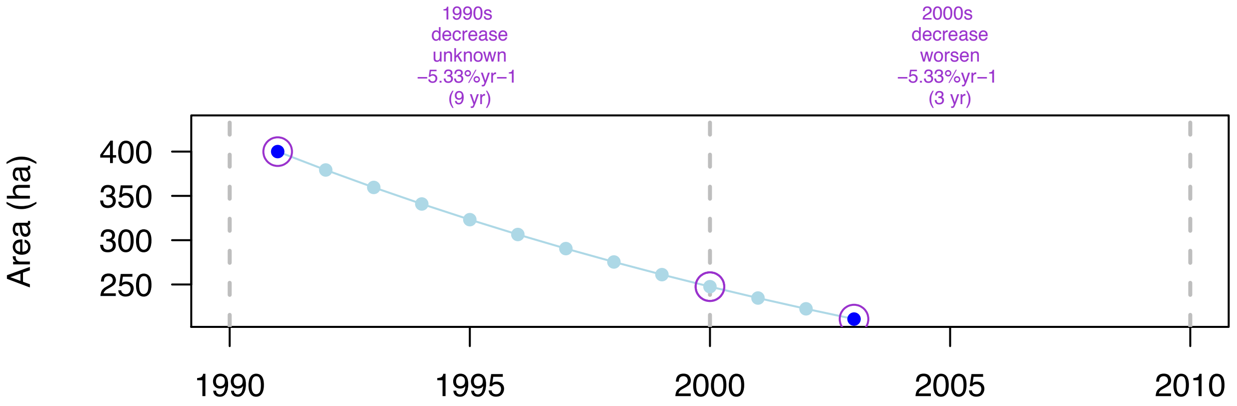

77_area

Portig 2006

SITE: Strangford Lough (United Kingdom – Atlantic) – Zm (? m)

OVERALL: Net = −646.8 ha; Rate = −1.61 % yr−1; Perc Final = 59 % > decrease

DECADAL: YES (33 yr)

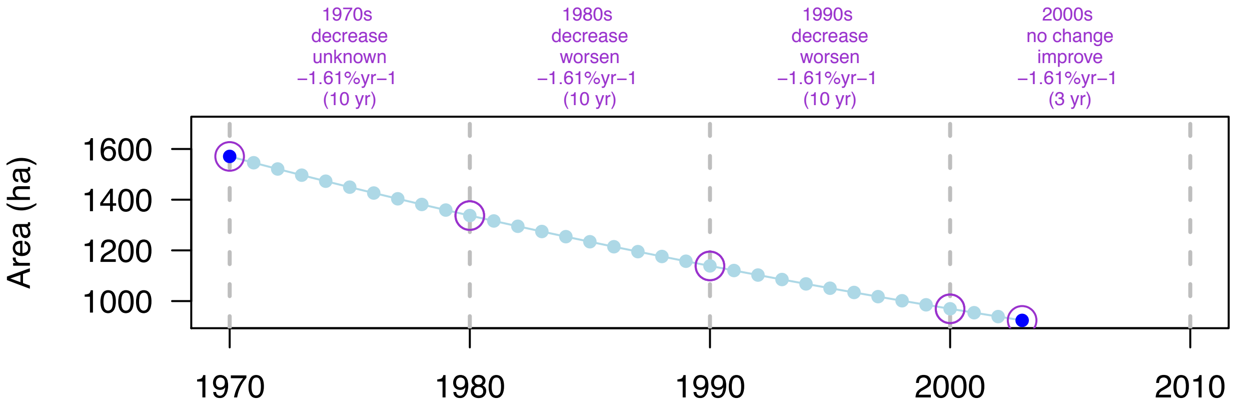

78_cover

Cook (unpublished)

SITE: Carrick Roads (United Kingdom – Atlantic) – Zn (? m)

OVERALL: Net = 14.26 %; Rate = 18.83 % yr−1; Perc Final = 146 % > increase

DECADAL: NO (2 yr)

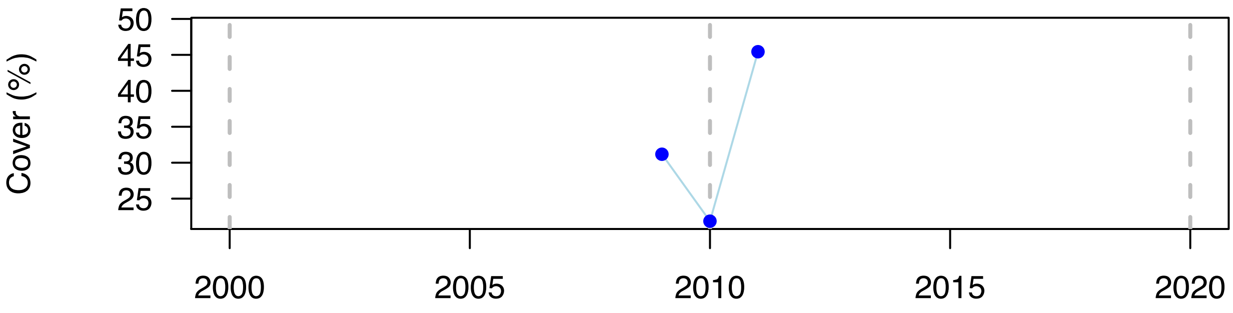

79_cover

Cook (unpublished)

SITE: Conwy Estuary (United Kingdom – Atlantic) – Zn (? m)

OVERALL: Net = 40 %;   Rate = 14.69 % yr−1; Perc Final = 180 % > increase

DECADAL: NO (4 yr)

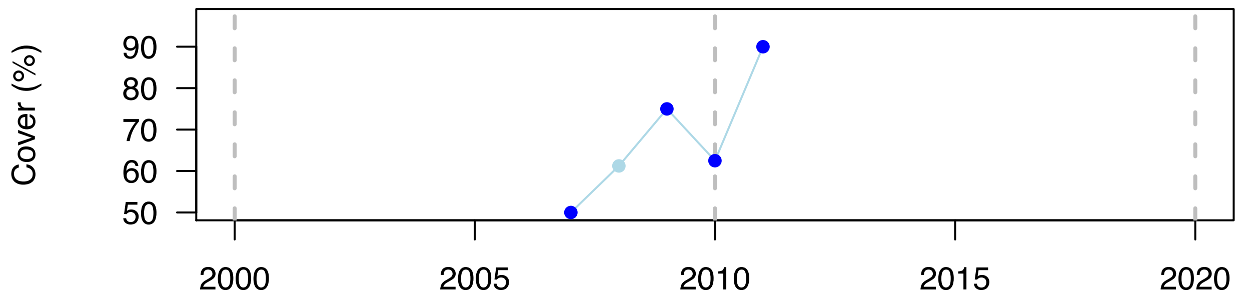

80_cover

Cook (unpublished)

SITE: Exe Estuary (United Kingdom – Atlantic) – Zn (? m)

OVERALL: Net = 15.46 %;   Rate = 7.26 % yr−1; Perc Final = 124 % > no change

DECADAL: NO (3 yr)

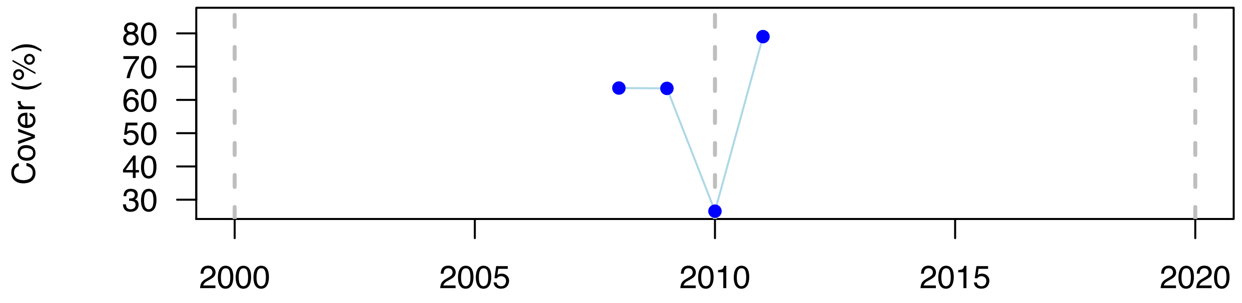

81_cover

Cook (unpublished)

SITE: Foryd bay (United Kingdom – Atlantic) – Zn (? m)

OVERALL: Net = −14.54 %; Rate = −11.55 % yr−1; Perc Final = 63 % > decrease

DECADAL: NO (4 yr)

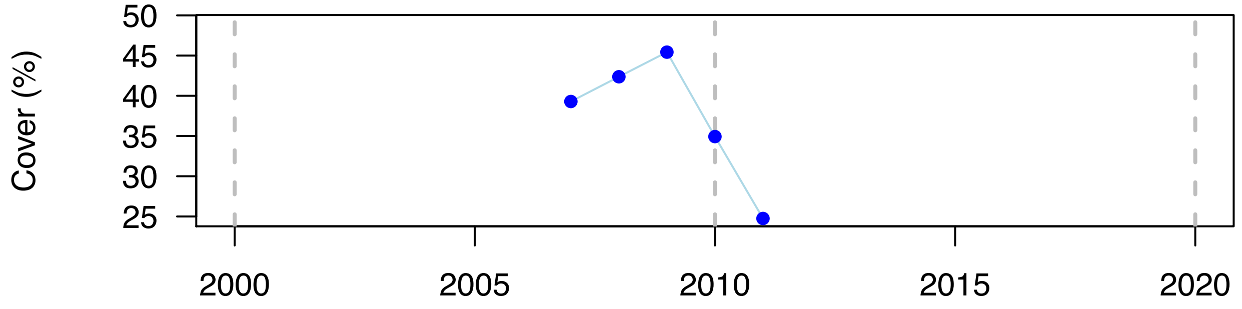

82_cover

Cook (unpublished)

SITE: Milford Haven (United Kingdom – Atlantic) – Zn (? m)

OVERALL: Net = 3.28 %; Rate = 1.81 % yr−1; Perc Final = 106 % > no change

DECADAL: NO (3 yr)

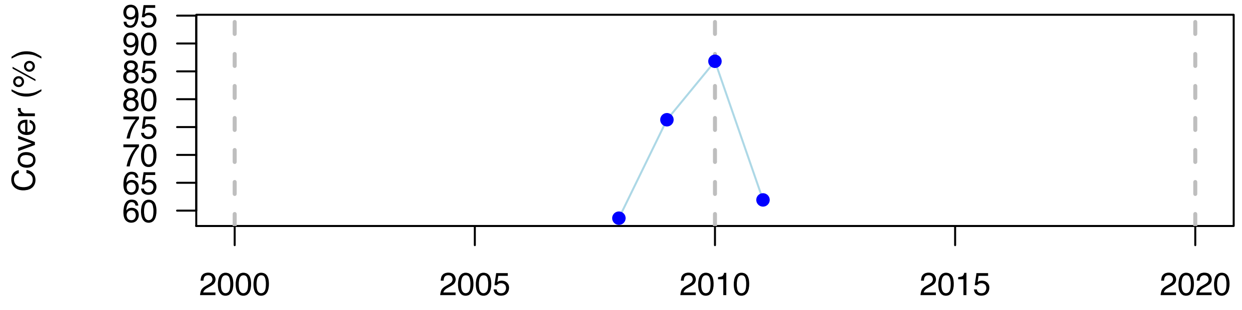

83_cover

Cook (unpublished)

SITE: Milford Haven (coastal) (United Kingdom – Atlantic) – Zn (? m)

OVERALL: Net = −13.18 %; Rate = −13.73 % yr−1; Perc Final = 76 % > no change

DECADAL: NO (2 yr)

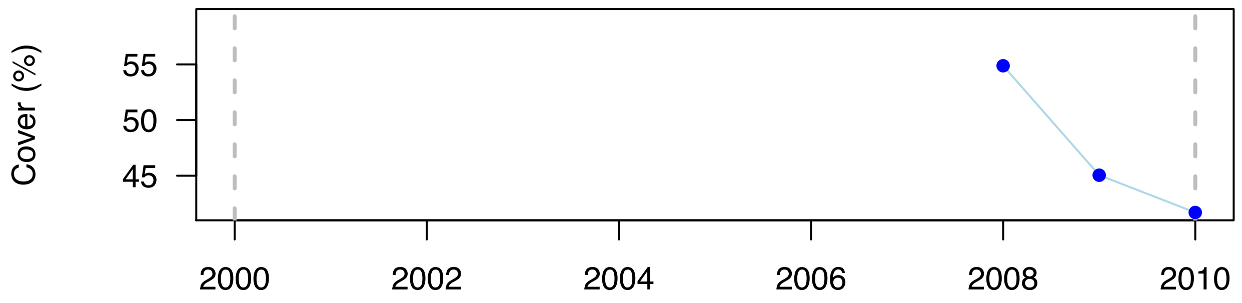

84_cover

Cook (unpublished)

SITE: Pagham Harbour (coastal) (United Kingdom – Atlantic) – Zm (? m)

OVERALL: Net = 28.02 %; Rate = 15.03 % yr−1; Perc Final = 157 % > increase

DECADAL: NO (3 yr)

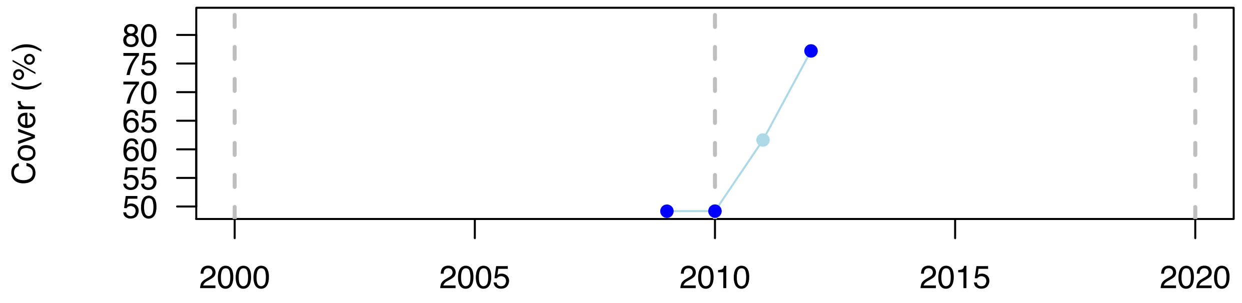

85_cover

Cook (unpublished)

SITE: Portsmouth Harbour (coastal) (United Kingdom – Atlantic) – Zn (? m)

OVERALL: Net = −20.6 %;   Rate = −23.7 % yr−1; Perc Final = 62 % > decrease

DECADAL: NO (2 yr)

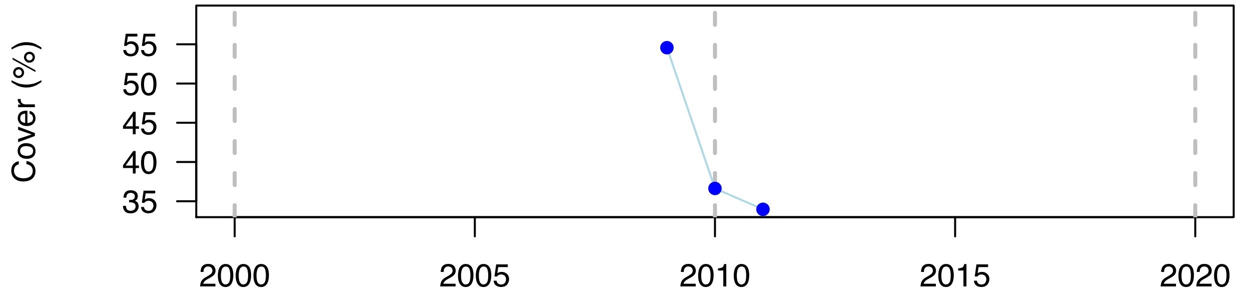

86_cover

Cook (unpublished)

SITE: Solent (United Kingdom – Atlantic) – Zn (? m)

OVERALL: Net = −15.17 %;   Rate = −11.79 % yr−1; Perc Final = 79 % > no change

DECADAL: NO (2 yr)

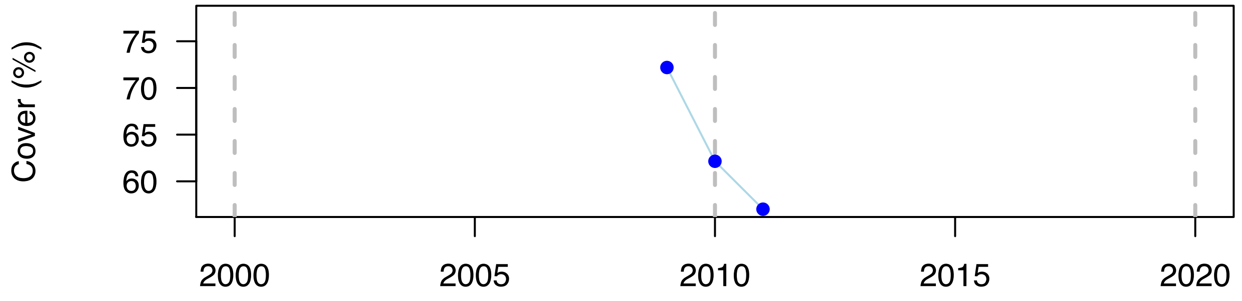

87_cover

Cook (unpublished)

SITE: Solent (United Kingdom – Atlantic) – Zm (? m)

OVERALL: Net = –9.62 %;   Rate = –57.18 % yr–1; Perc Final = 32 % > decrease

DECADAL: NO (2 yr)

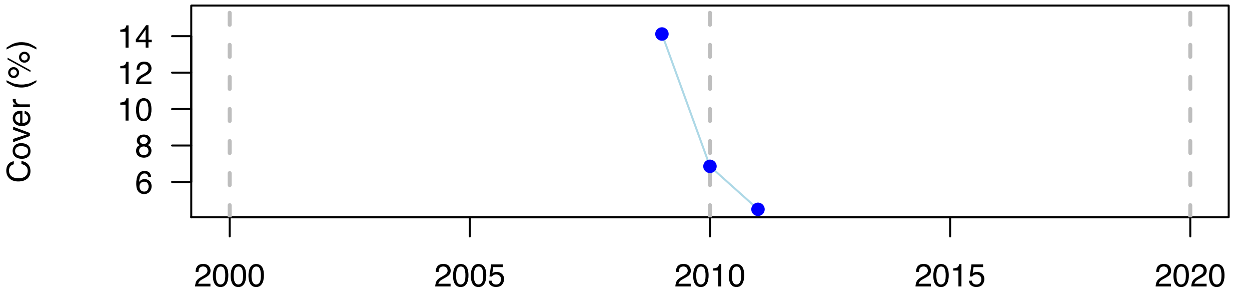

88_cover

Cook (unpublished)

SITE: Thames Estuary (United Kingdom – Atlantic) – Zn (? m)

OVERALL: Net = –41.45 %;   Rate = –13.65 % yr–1; Perc Final = 58 % > decrease

DECADAL: NO (4 yr)

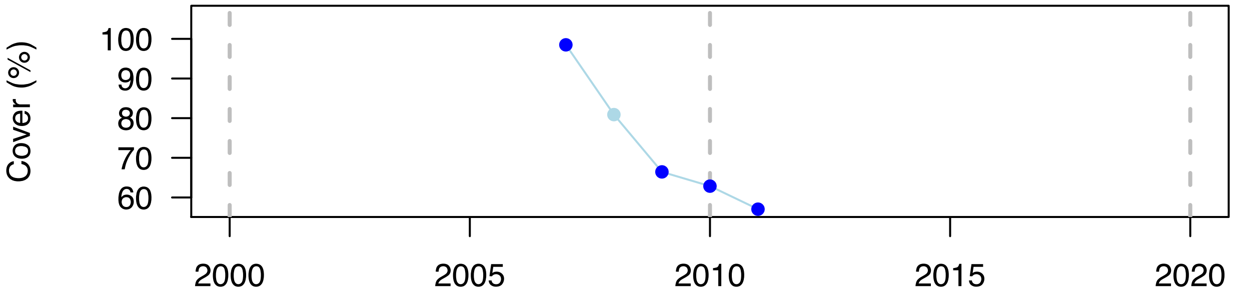

89_cover

Cook (unpublished)

SITE: Thames Estuary (United Kingdom – Atlantic) – Zm (? m)

OVERALL: Net = 9.42 %;   Rate = 26.96 % yr–1; Perc Final = 171 % > increase

DECADAL: NO (2 yr)

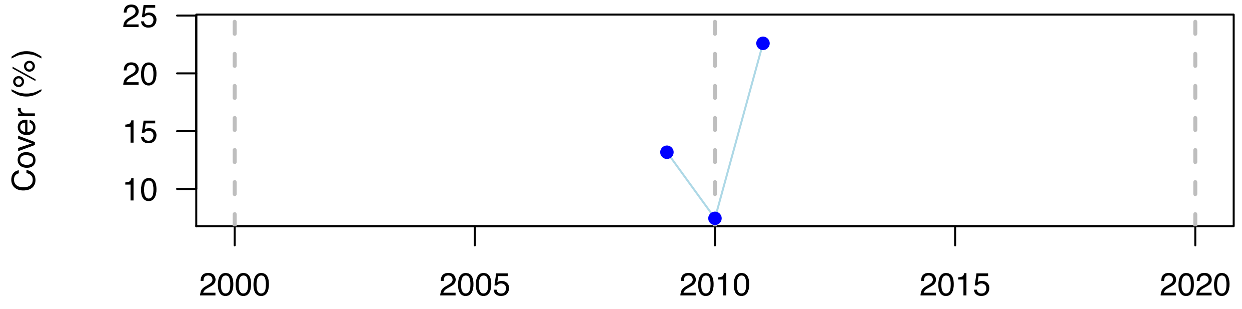

90_area

Den Hartog 1994

SITE: Langstone Harbour (United Kingdom – Atlantic) – Zm (? m)

OVERALL: Net = –10 ha;   Rate = NA % yr–1; Perc Final = NA % > decrease

DECADAL: NO (6 yr)

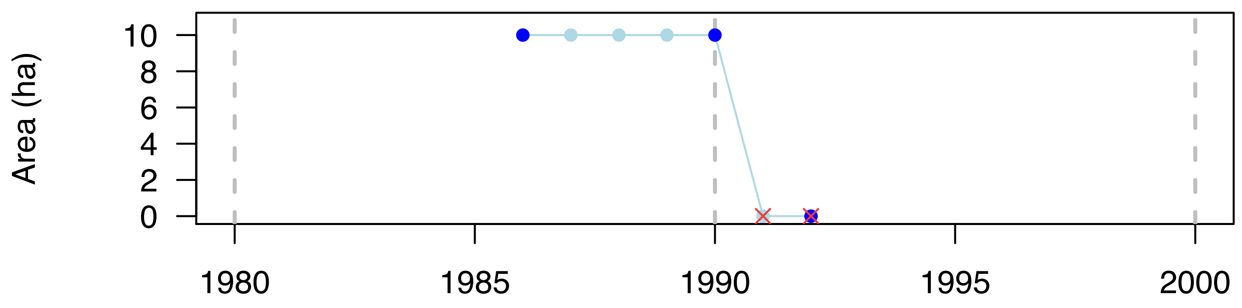

91_biomass

Milchakova and Phillips 2003

SITE: Holland Bay (Ukraine – Mediterranean) – Zm (–2 m)

OVERALL: Net = –513 g dw m–2;   Rate = –0.93 % yr–1; Perc Final = 85 % > no change

DECADAL: YES (18 yr)

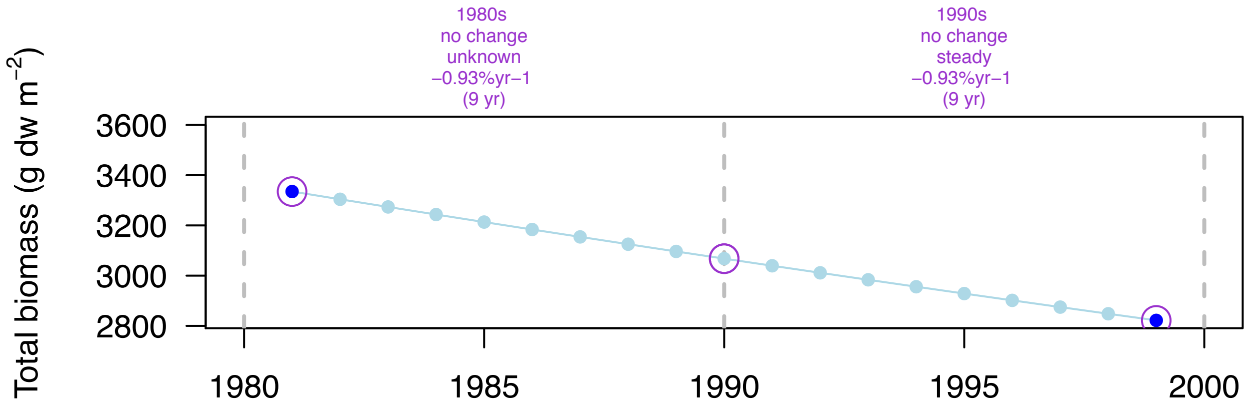

91_density

Milchakova and Phillips 2003

SITE: Holland Bay (Ukraine – Mediterranean) – Zm (–2 m)

OVERALL: Net = 216 shoot m–2;   Rate = 1.45 % yr–1; Perc Final = 130 % > increase

DECADAL: YES (18 yr)

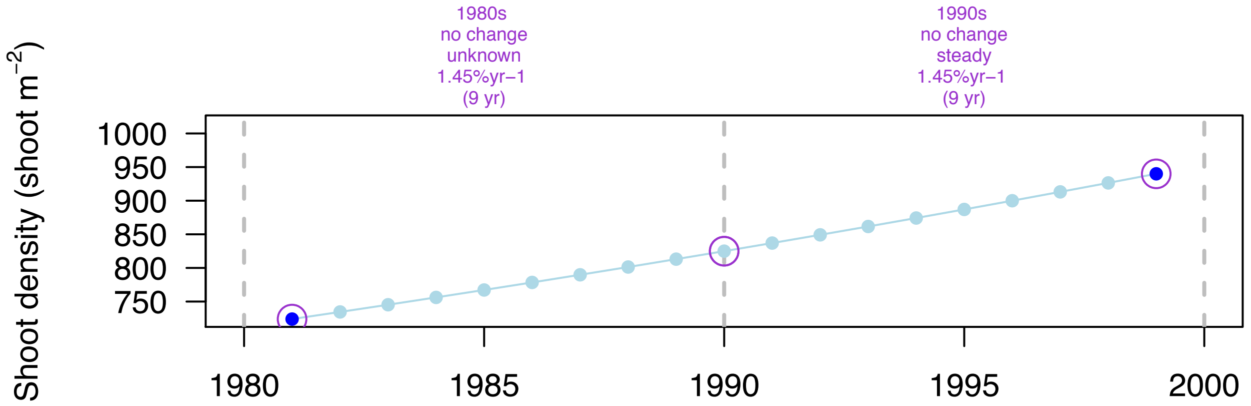

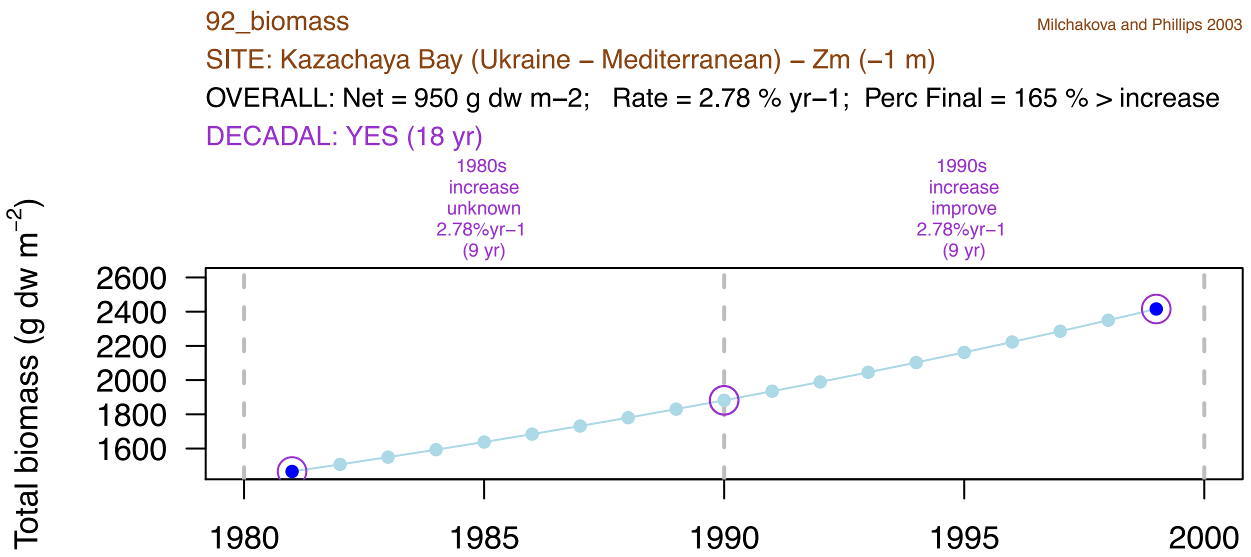
92_biomass
Milchakova and Phillips 2003
SITE: Kazachaya Bay (Ukraine – Mediterranean) – Zm (–1 m)
OVERALL: Net = 950 g dw m–2; Rate = 2.78 % yr–1; Perc Final = 165 % > increase
DECADAL: YES (18 yr)
1980s
increase
unknown
2.78%yr–1
(9 yr)
1990s
increase
improve
2.78%yr–1
(9 yr)
Total biomass (g dw m^{-2})
2600
2400
2200
2000
1800
1600
1980
1985
1990
1995
2000

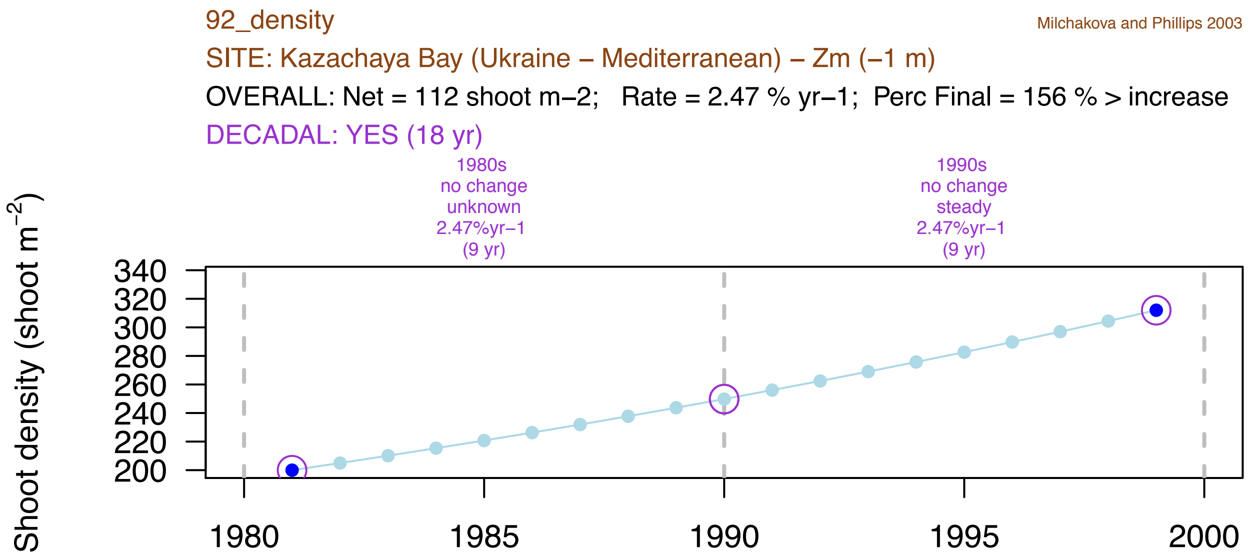
92_density
Milchakova and Phillips 2003
SITE: Kazachaya Bay (Ukraine – Mediterranean) – Zm (–1 m)
OVERALL: Net = 112 shoot m–2; Rate = 2.47 % yr–1; Perc Final = 156 % > increase
DECADAL: YES (18 yr)
1980s
no change
unknown
2.47%yr–1
(9 yr)
1990s
no change
steady
2.47%yr–1
(9 yr)
Shoot density (shoot m^{-2})
340
320
300
280
260
240
220
200
1980
1985
1990
1995
2000

93_biomass

Milchakova and Phillips 2003

SITE: Kazachaya Bay (Ukraine – Mediterranean) – Zm (–3 m)

OVERALL: Net = 629 g dw m–2; Rate = 1.88 % yr–1; Perc Final = 140 % > increase

DECADAL: YES (18 yr)

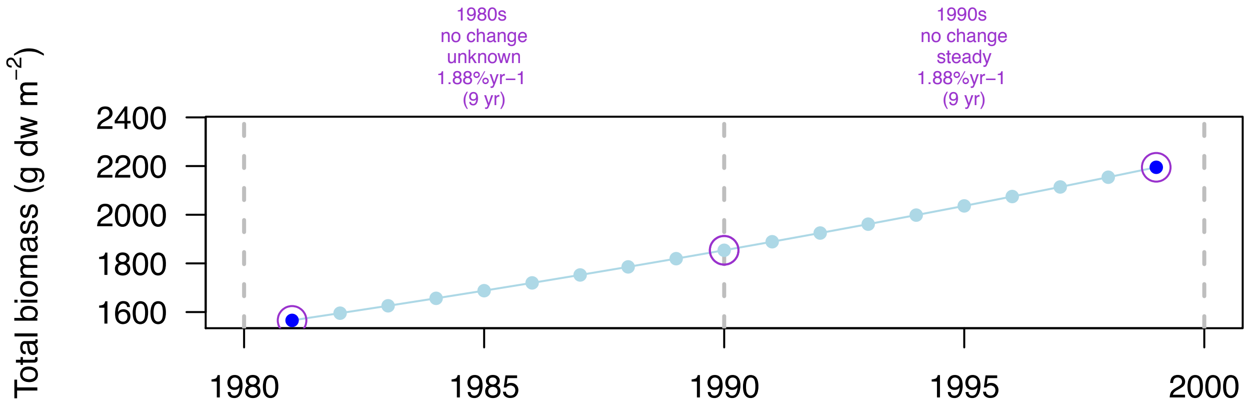

93_density

Milchakova and Phillips 2003

SITE: Kazachaya Bay (Ukraine – Mediterranean) – Zm (–3 m)

OVERALL: Net = 220 shoot m–2; Rate = 3.53 % yr–1; Perc Final = 189 % > increase

DECADAL: YES (18 yr)

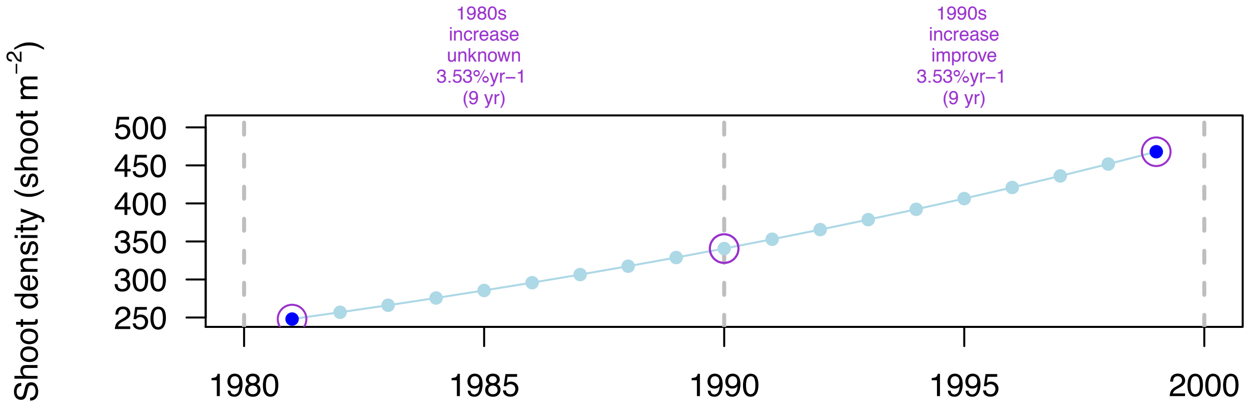

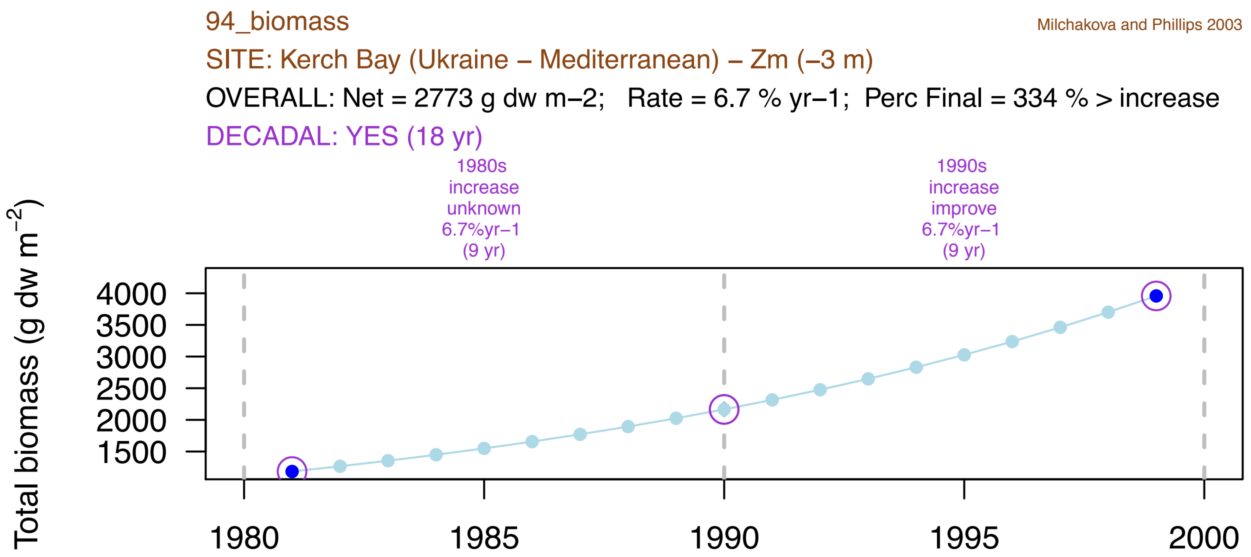
94_biomass
Milchakova and Phillips 2003
SITE: Kerch Bay (Ukraine – Mediterranean) – Zm (–3 m)
OVERALL: Net = 2773 g dw m–2; Rate = 6.7 % yr–1; Perc Final = 334 % > increase
DECADAL: YES (18 yr)
1980s
increase
unknown
6.7%yr–1
(9 yr)
1990s
increase
improve
6.7%yr–1
(9 yr)
Total biomass (g dw m−2)
4000
3500
3000
2500
2000
1500
1980
1985
1990
1995
2000

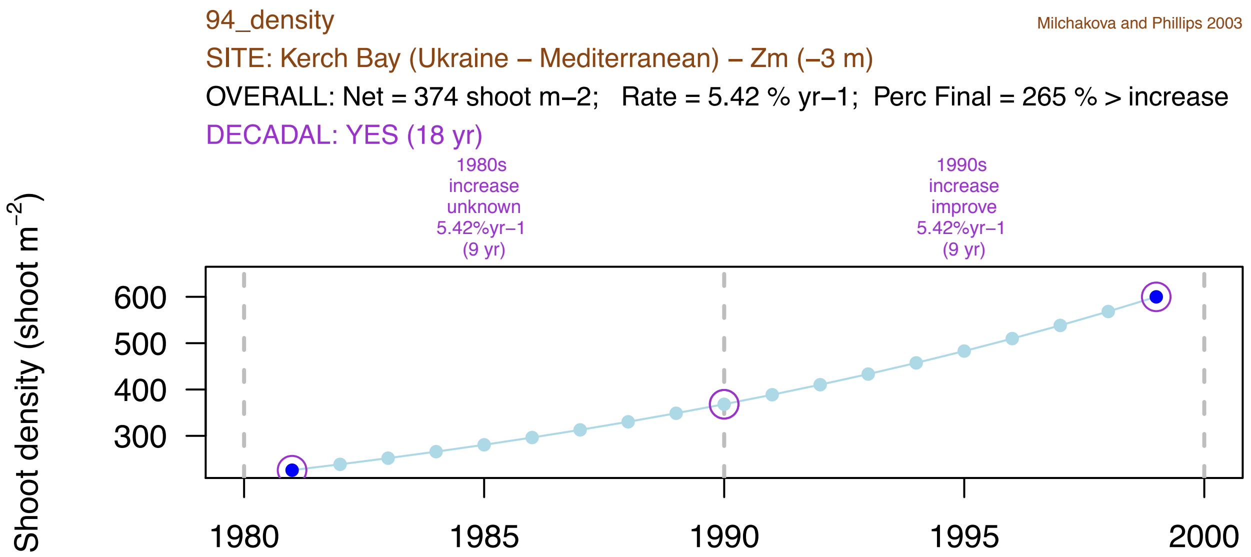
94_density
Milchakova and Phillips 2003
SITE: Kerch Bay (Ukraine – Mediterranean) – Zm (–3 m)
OVERALL: Net = 374 shoot m–2; Rate = 5.42 % yr–1; Perc Final = 265 % > increase
DECADAL: YES (18 yr)
1980s
increase
unknown
5.42%yr–1
(9 yr)
1990s
increase
improve
5.42%yr–1
(9 yr)
Shoot density (shoot m−2)
600
500
400
300
1980
1985
1990
1995
2000

95_biomass

Milchakova and Phillips 2003

SITE: Kerch Strait (Ukraine – Mediterranean) – Zm (–3 m)

OVERALL: Net = 2539 g dw m–2;   Rate = 3.28 % yr–1; Perc Final = 180 % > increase

DECADAL: YES (18 yr)

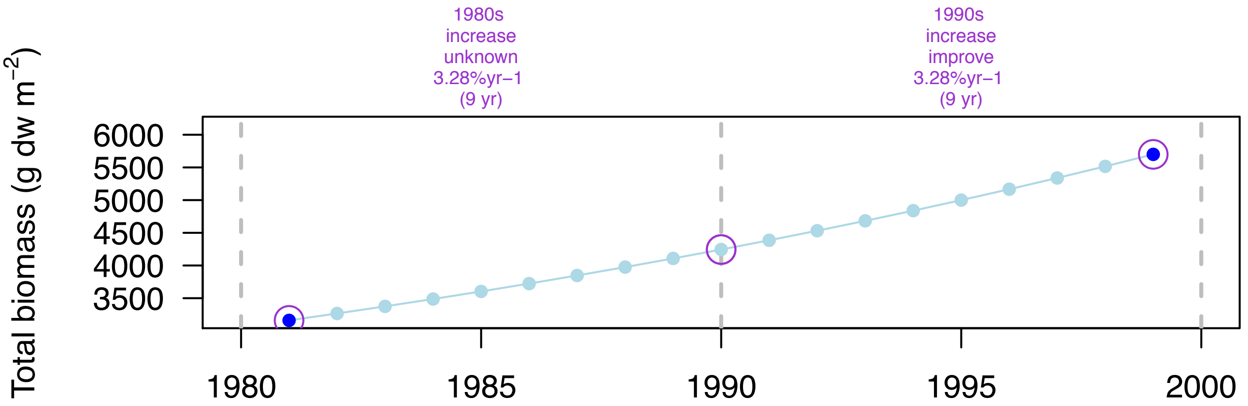

95_density

Milchakova and Phillips 2003

SITE: Kerch Strait (Ukraine – Mediterranean) – Zm (–3 m)

OVERALL: Net = –521 shoot m–2;   Rate = –4.16 % yr–1; Perc Final = 47 % > decrease

DECADAL: YES (18 yr)

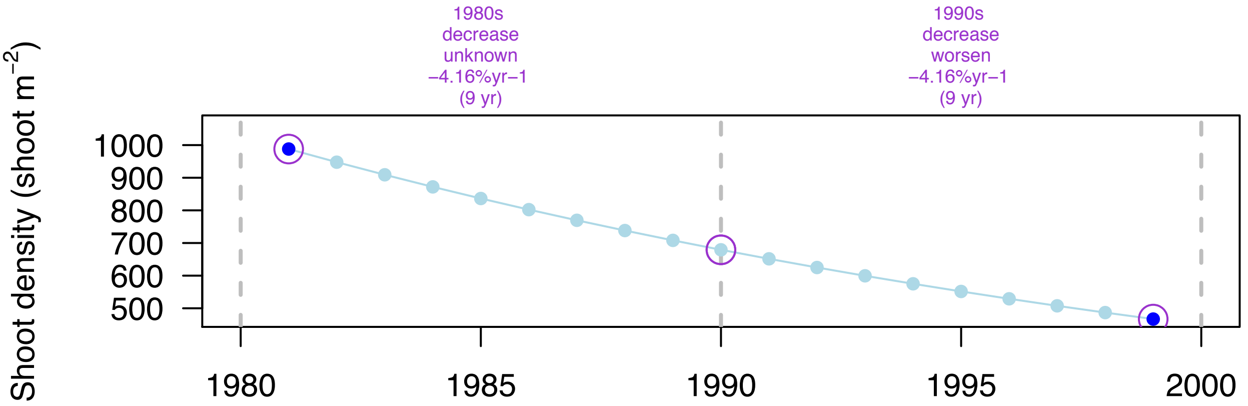

96_cover

Cook (unpublished)

SITE: Portsmouth Harbour (coastal) (United Kingdom – Atlantic) – Zm (? m)

OVERALL: Net = −15.88 %; Rate = −20.03 % yr−1; Perc Final = 67 % > decrease

DECADAL: NO (2 yr)

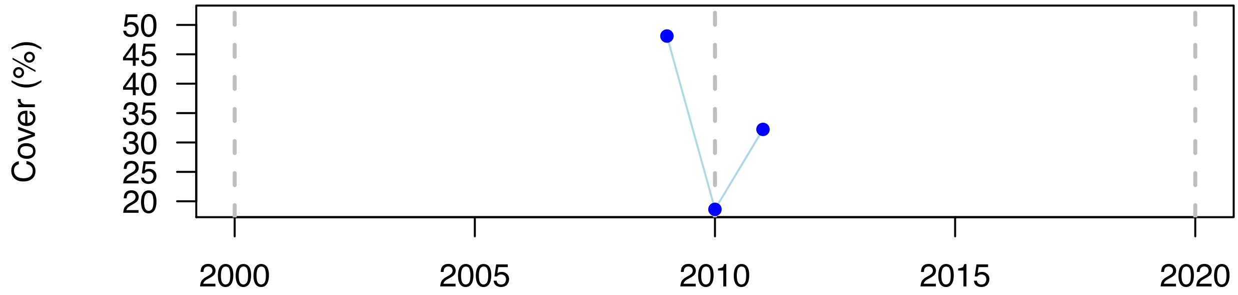

97_biomass

Milchakova and Phillips 2003

SITE: Laspi Bay (Ukraine – Mediterranean) – Zm (−5 m)

OVERALL: Net = 1309 g dw m−2; Rate = 5.26 % yr−1; Perc Final = 258 % > increase

DECADAL: YES (18 yr)

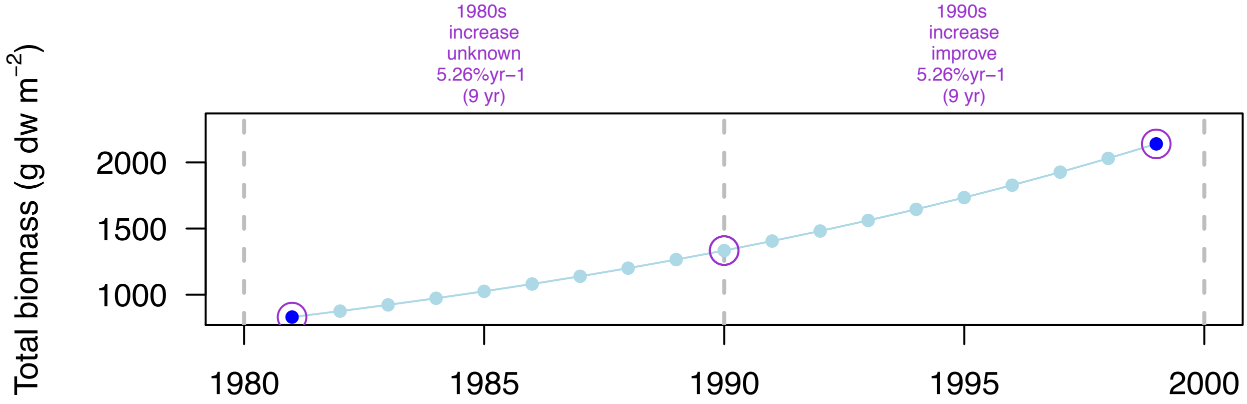

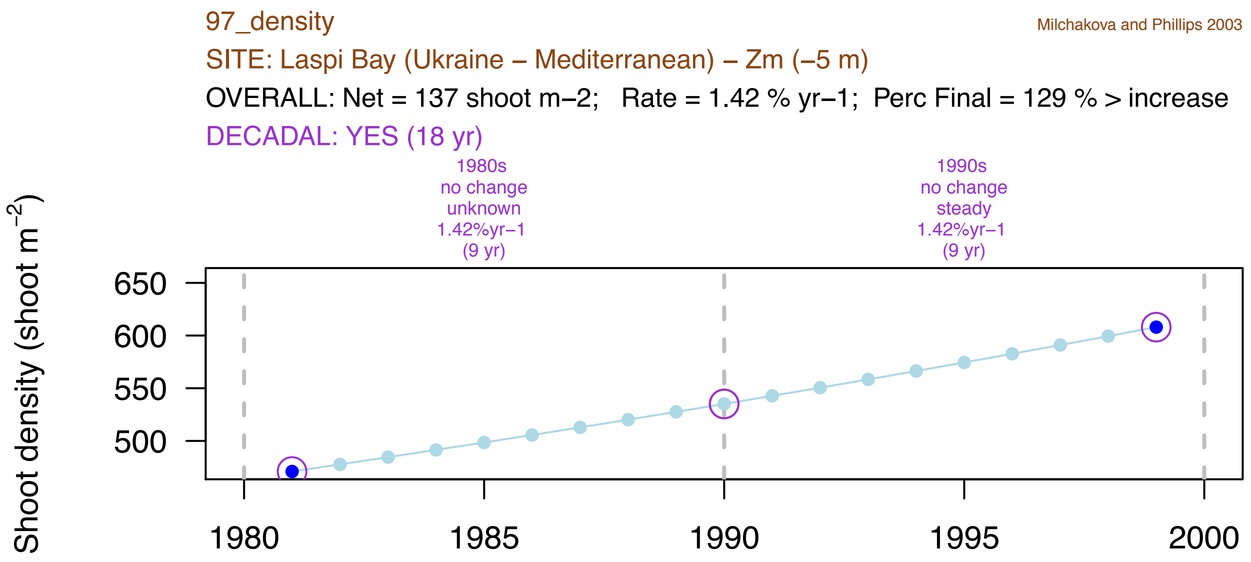
97_density
Milchakova and Phillips 2003
SITE: Laspi Bay (Ukraine – Mediterranean) – Zm (–5 m)
OVERALL: Net = 137 shoot m–2; Rate = 1.42 % yr–1; Perc Final = 129 % > increase
DECADAL: YES (18 yr)
1980s no change unknown 1.42%yr–1 (9 yr)
1990s no change steady 1.42%yr–1 (9 yr)
Shoot density (shoot m^{-2})
650
600
550
500
1980
1985
1990
1995
2000

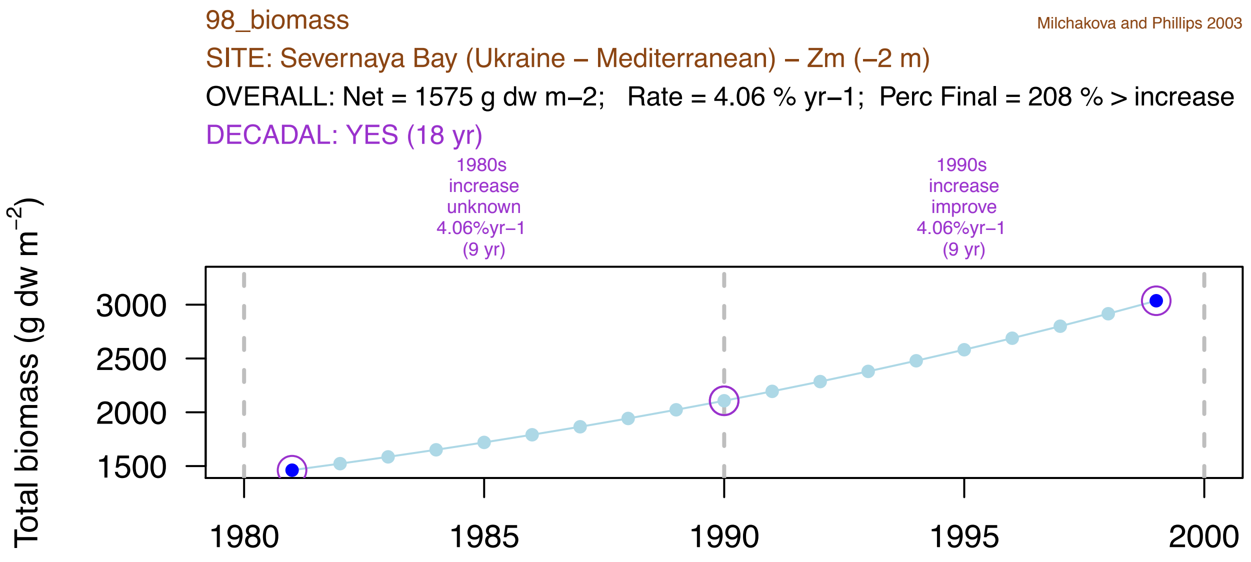
98_biomass
Milchakova and Phillips 2003
SITE: Severnaya Bay (Ukraine – Mediterranean) – Zm (–2 m)
OVERALL: Net = 1575 g dw m–2; Rate = 4.06 % yr–1; Perc Final = 208 % > increase
DECADAL: YES (18 yr)
1980s increase unknown 4.06%yr–1 (9 yr)
1990s increase improve 4.06%yr–1 (9 yr)
Total biomass (g dw m^{-2})
3000
2500
2000
1500
1980
1985
1990
1995
2000

98_density

Milchakova and Phillips 2003

SITE: Severnaya Bay (Ukraine – Mediterranean) – Zm (–2 m)

OVERALL: Net = 368 shoot m–2; Rate = 3.68 % yr–1; Perc Final = 194 % > increase

DECADAL: YES (18 yr)

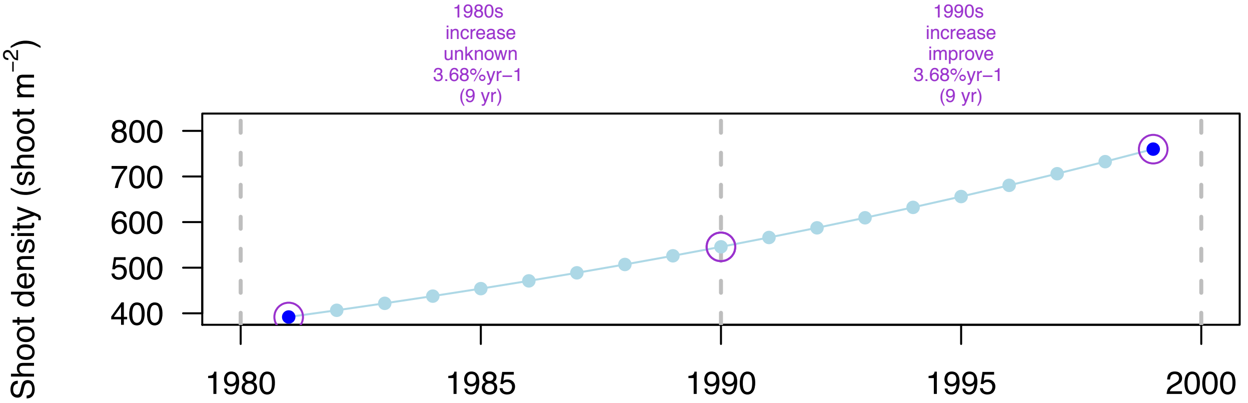

99_biomass

Milchakova and Phillips 2003

SITE: Streletskaya Bay (Ukraine – Mediterranean) – Zm (–2 m)

OVERALL: Net = 1654 g dw m–2; Rate = 4 % yr–1; Perc Final = 205 % > increase

DECADAL: YES (18 yr)

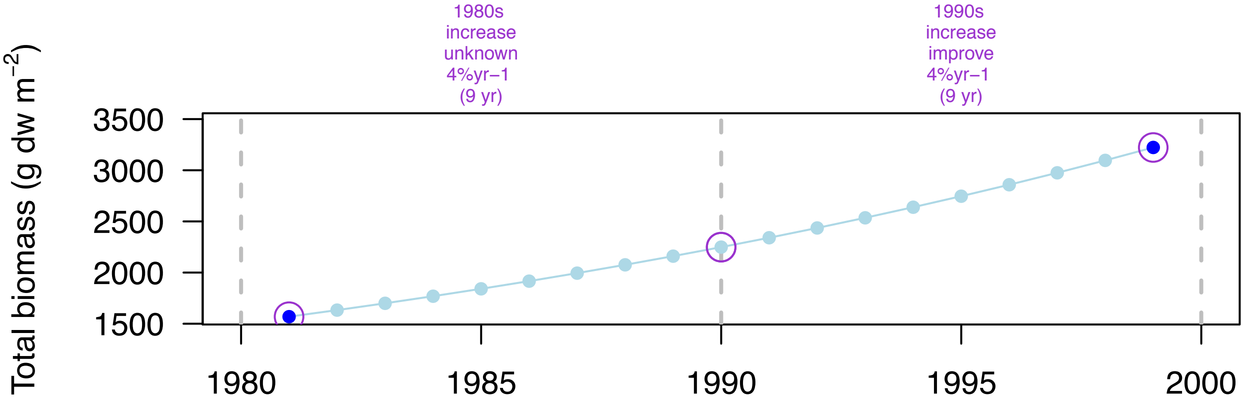

99_density

Milchakova and Phillips 2003

SITE: Streletskaya Bay (Ukraine – Mediterranean) – Zm (–2 m)

OVERALL: Net = 684 shoot m–2;   Rate = 7.29 % yr–1; Perc Final = 371 % > increase

DECADAL: YES (18 yr)

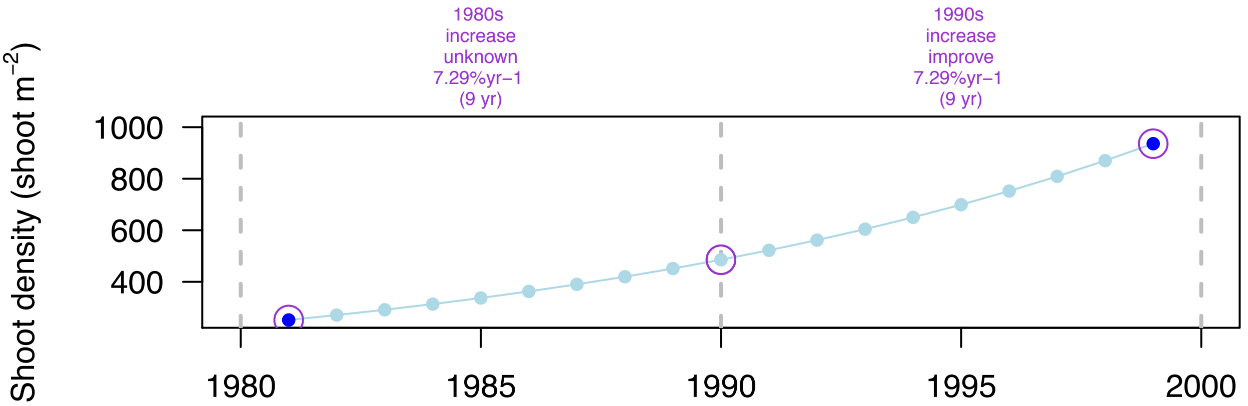

100_lowerlimit

Dural et al. 2012

SITE: Hekim Island (Turkey – Mediterranean) – Po (? m)

OVERALL: Net = –8 m;   Rate = –3.06 % yr–1; Perc Final = 71 % > decrease

DECADAL: YES (11 yr)

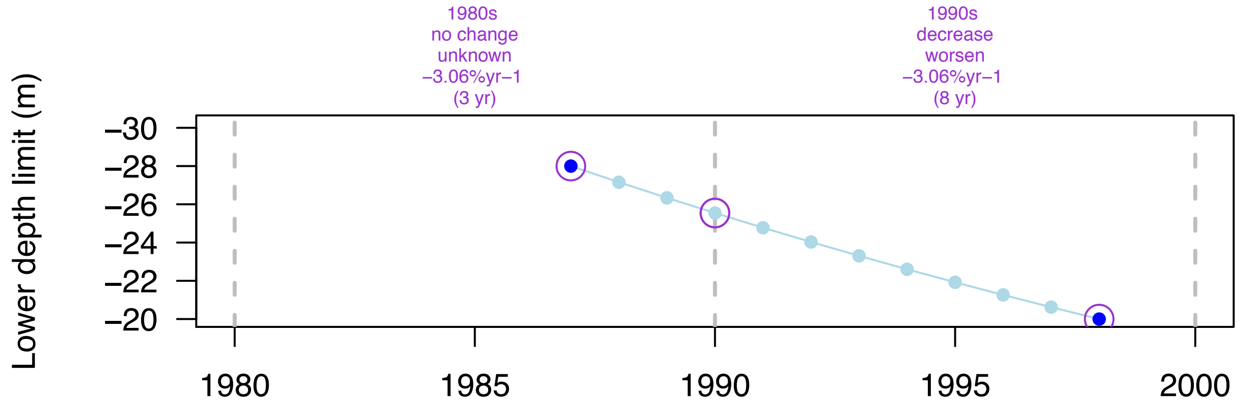

101_area

de Jong (unpublished)

SITE: Eastern Scheldt (The Netherlands – Atlantic) – Zn (0.4 m)

OVERALL: Net = –924.3 ha; Rate = –11.94 % yr–1; Perc Final = 2 % > decrease

DECADAL: YES (32 yr)

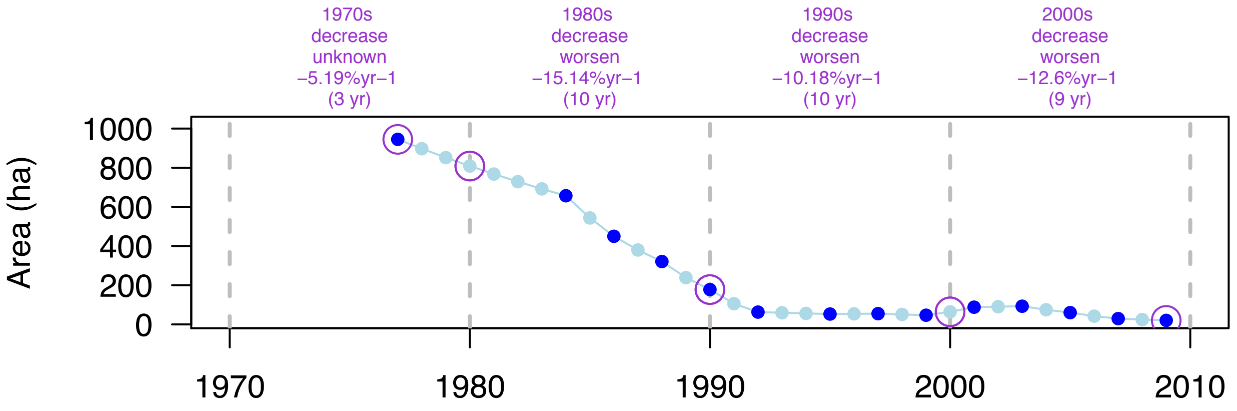

101_lowerlimit

de Jong (unpublished)

SITE: Eastern Scheldt (The Netherlands – Atlantic) – Zn (0.4 m)

OVERALL: Net = 0 m; Rate = 0 % yr–1; Perc Final = 100 % > no change

DECADAL: YES (32 yr)

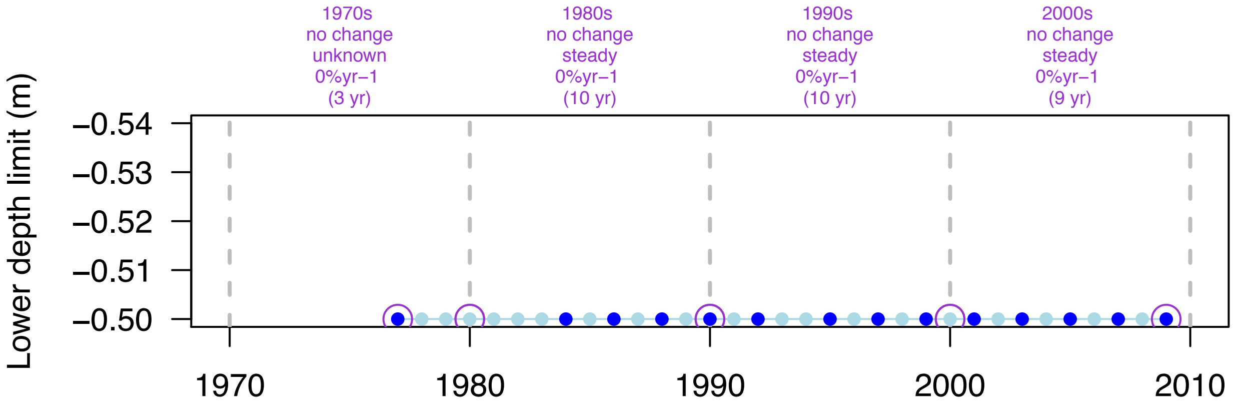

101_upperlimit
de Jong (unpublished)
SITE: Eastern Scheldt (The Netherlands – Atlantic) – Zn (0.4 m)
OVERALL: Net = 0 m;   Rate = 0 % yr–1; Perc Final = 100 % > no change
DECADAL: YES (32 yr)

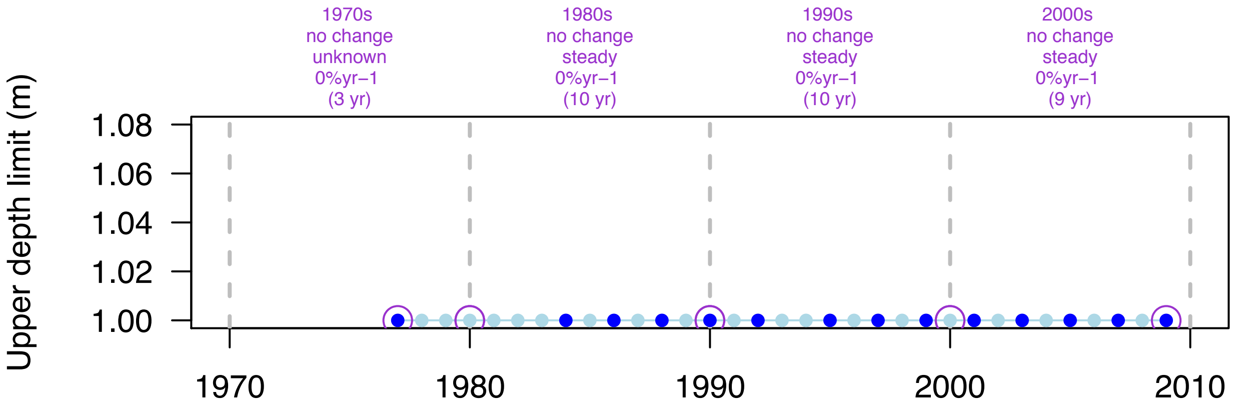

102_area
de Jong (unpublished)
SITE: Eastern Scheldt Galgeplaat (The Netherlands – Atlantic) – Zm (–0.2 m)
OVERALL: Net = –1 ha;   Rate = NA % yr–1; Perc Final = NA % > decrease
DECADAL: YES (8 yr)

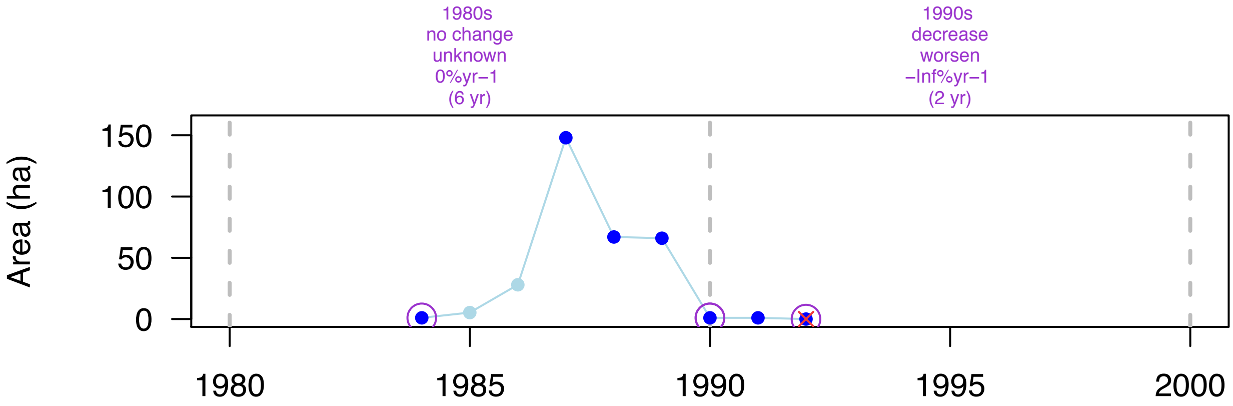

103_area

de Jong (unpublished)

SITE: Eastern Scheldt Roggenplaat (The Netherlands – Atlantic) – Zm (–0.1 m)

OVERALL: Net = –1 ha;   Rate = NA % yr–1; Perc Final = NA % > decrease

DECADAL: YES (22 yr)

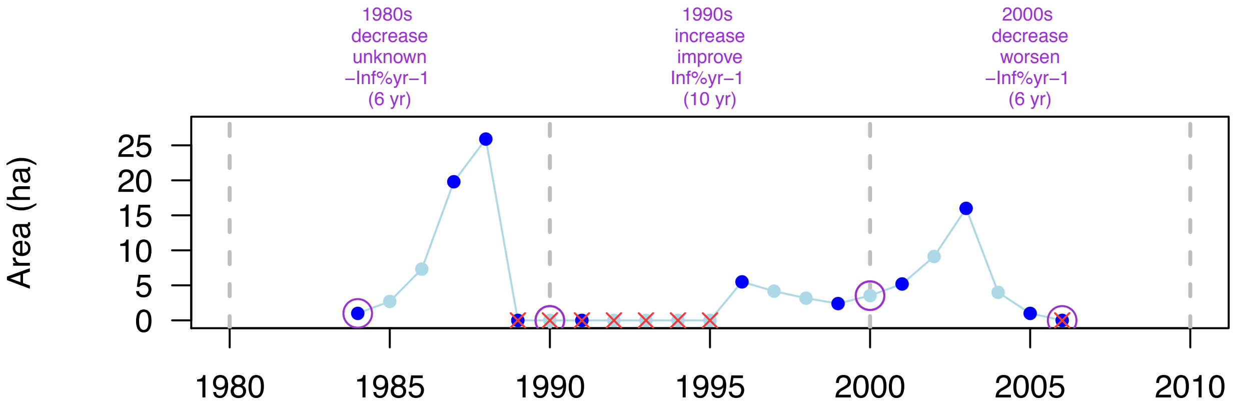

103_lowerlimit

de Jong (unpublished)

SITE: Eastern Scheldt Roggenplaat (The Netherlands – Atlantic) – Zm (–0.1 m)

OVERALL: Net = 0 m;   Rate = 0 % yr–1; Perc Final = 100 % > no change

DECADAL: YES (19 yr)

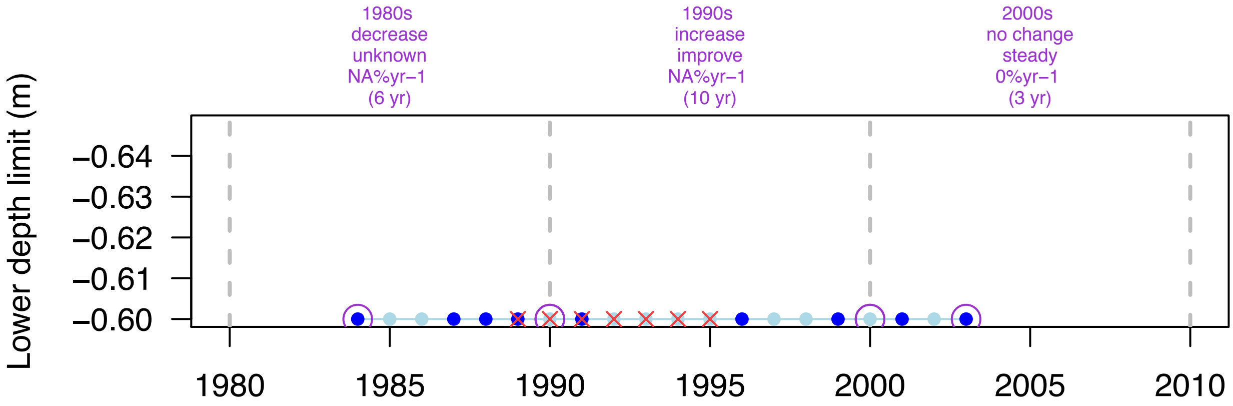

103_upperlimit

de Jong (unpublished)

SITE: Eastern Scheldt Roggenplaat (The Netherlands – Atlantic) – Zm (–0.1 m)

OVERALL: Net = 0 m;   Rate = 0 % yr–1; Perc Final = 100 % > no change

DECADAL: YES (19 yr)

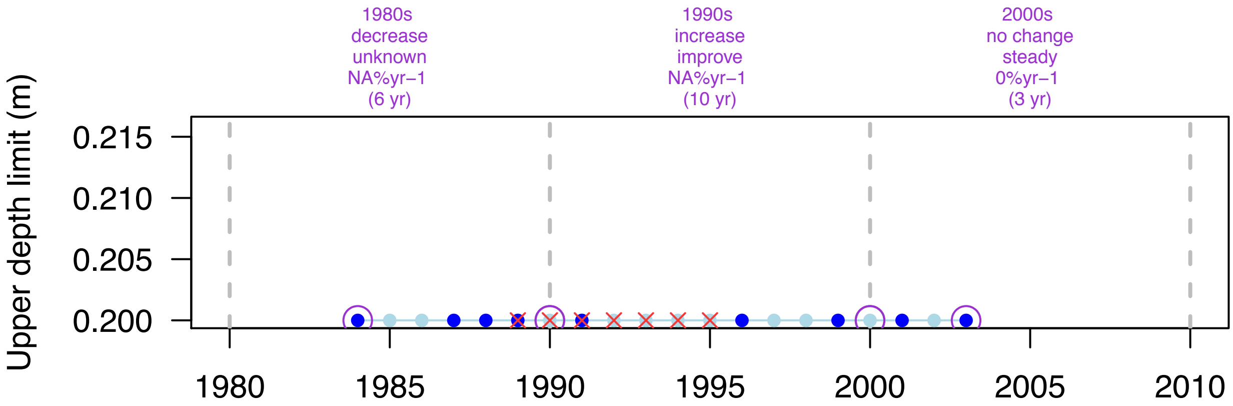

104_area

de Jong (unpublished)

SITE: Groningen kwelderwerken (The Netherlands – Atlantic) – Zn (0 m)

OVERALL: Net = 91.5 ha;   Rate = 8.6 % yr–1; Perc Final = 722 % > increase

DECADAL: YES (23 yr)

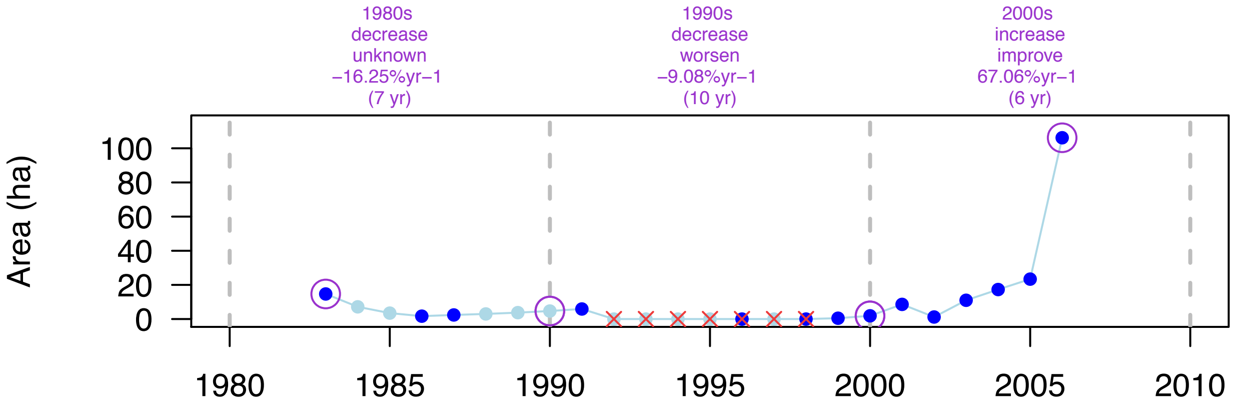

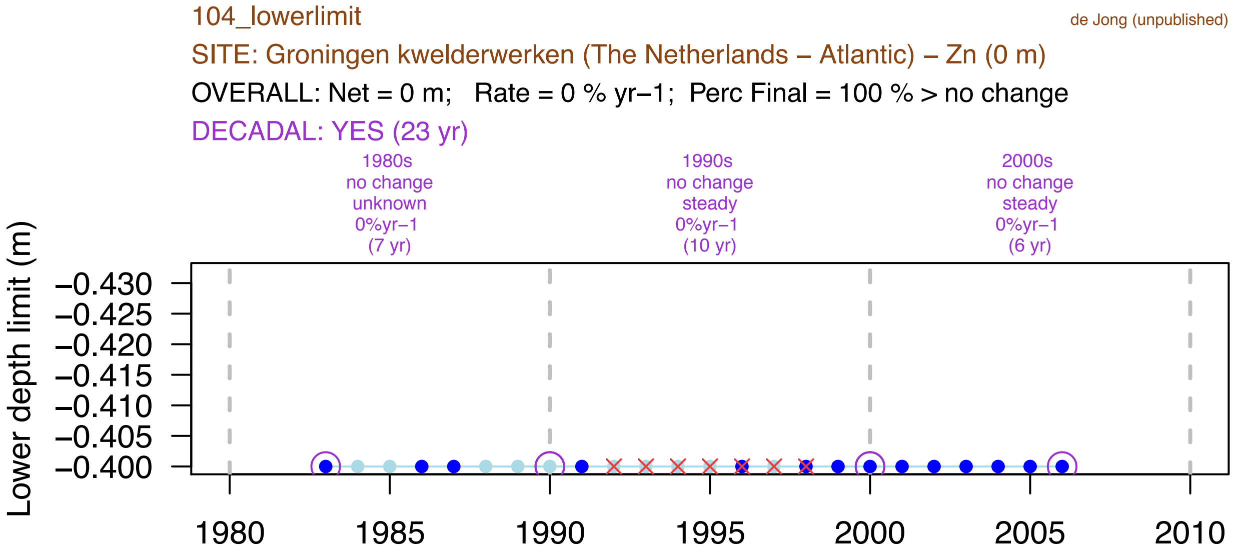

104_lowerlimit
de Jong (unpublished)
SITE: Groningen kwelderwerken (The Netherlands – Atlantic) – Zn (0 m)
OVERALL: Net = 0 m; Rate = 0 % yr–1; Perc Final = 100 % > no change
DECADAL: YES (23 yr)
1980s
no change
unknown
0%yr–1
(7 yr)
1990s
no change
steady
0%yr–1
(10 yr)
2000s
no change
steady
0%yr–1
(6 yr)
Lower depth limit (m)
–0.430
–0.425
–0.420
–0.415
–0.410
–0.405
–0.400
1980
1985
1990
1995
2000
2005
2010

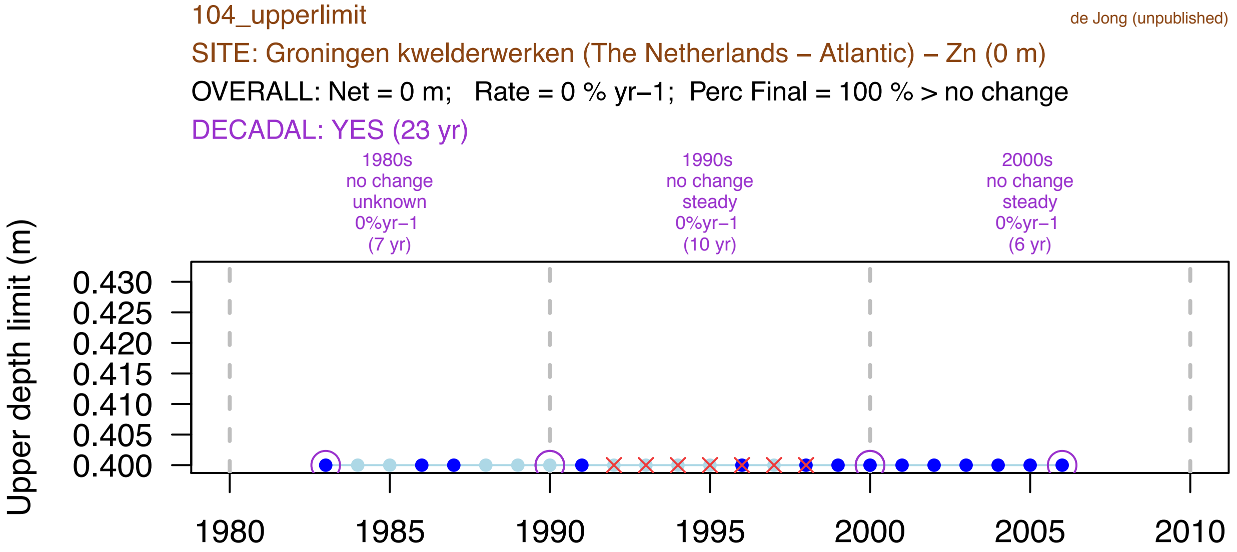

104_upperlimit
de Jong (unpublished)
SITE: Groningen kwelderwerken (The Netherlands – Atlantic) – Zn (0 m)
OVERALL: Net = 0 m; Rate = 0 % yr–1; Perc Final = 100 % > no change
DECADAL: YES (23 yr)
1980s
no change
unknown
0%yr–1
(7 yr)
1990s
no change
steady
0%yr–1
(10 yr)
2000s
no change
steady
0%yr–1
(6 yr)
Upper depth limit (m)
0.430
0.425
0.420
0.415
0.410
0.405
0.400
1980
1985
1990
1995
2000
2005
2010

105_area

de Jong (unpublished)

SITE: Groningen kwelderwerken (The Netherlands – Atlantic) – Zm (0 m)

OVERALL: Net = −0.7 ha;   Rate = −2.13 % yr−1; Perc Final = 77 % > decrease

DECADAL: YES (12 yr)

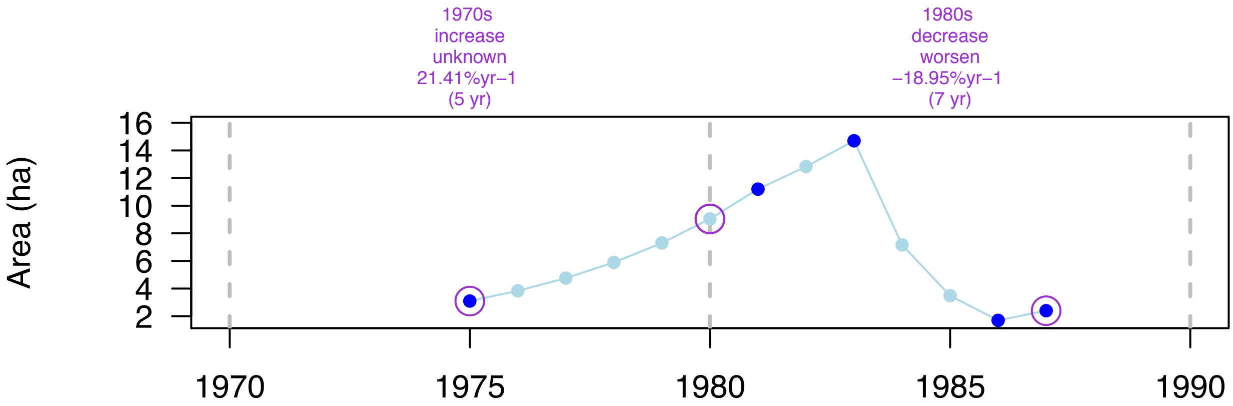

105_lowerlimit

de Jong (unpublished)

SITE: Groningen kwelderwerken (The Netherlands – Atlantic) – Zm (0 m)

OVERALL: Net = 0 m;   Rate = 0 % yr−1; Perc Final = 100 % > no change

DECADAL: YES (12 yr)

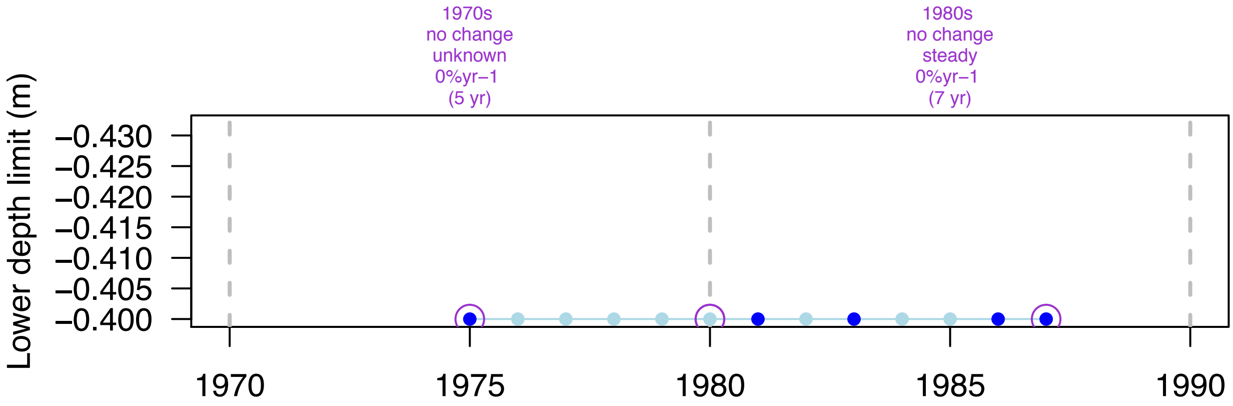

105_upperlimit

de Jong (unpublished)

SITE: Groningen kwelderwerken (The Netherlands – Atlantic) – Zm (0 m)

OVERALL: Net = 0 m; Rate = 0 % yr–1; Perc Final = 100 % > no change

DECADAL: YES (12 yr)

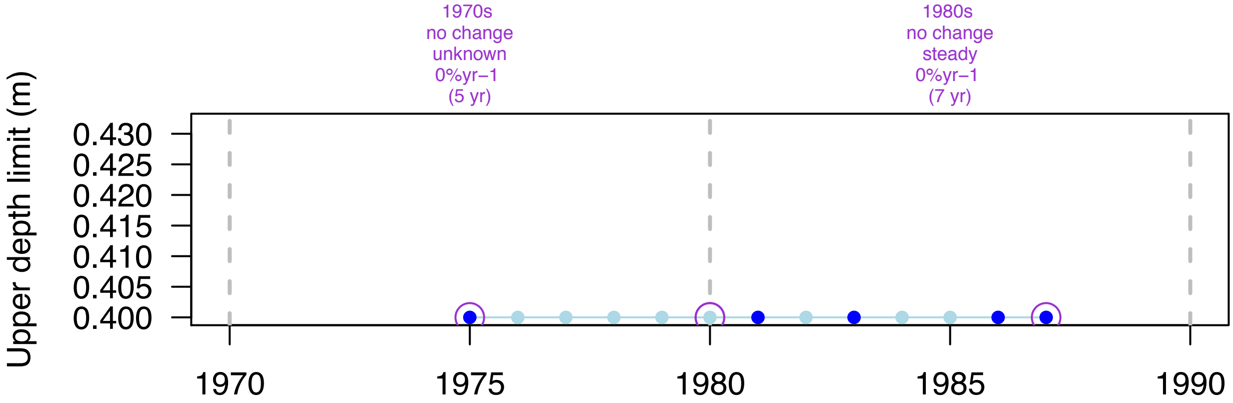

106_area

de Jong (unpublished)

SITE: Lake Veere (The Netherlands – Atlantic) – Zm (–2 m)

OVERALL: Net = –64.7 ha; Rate = NA % yr–1; Perc Final = NA % > decrease

DECADAL: YES (18 yr)

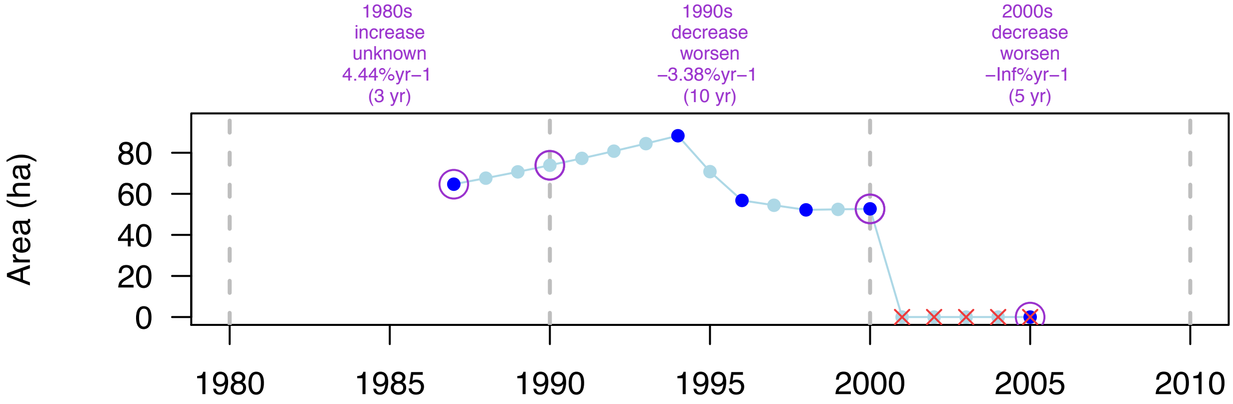

106_lowerlimit

de Jong (unpublished)

SITE: Lake Veere (The Netherlands – Atlantic) – Zm (–2 m)

OVERALL: Net = NA m;   Rate = NA % yr–1; Perc Final = NA % > decrease

DECADAL: YES (18 yr)

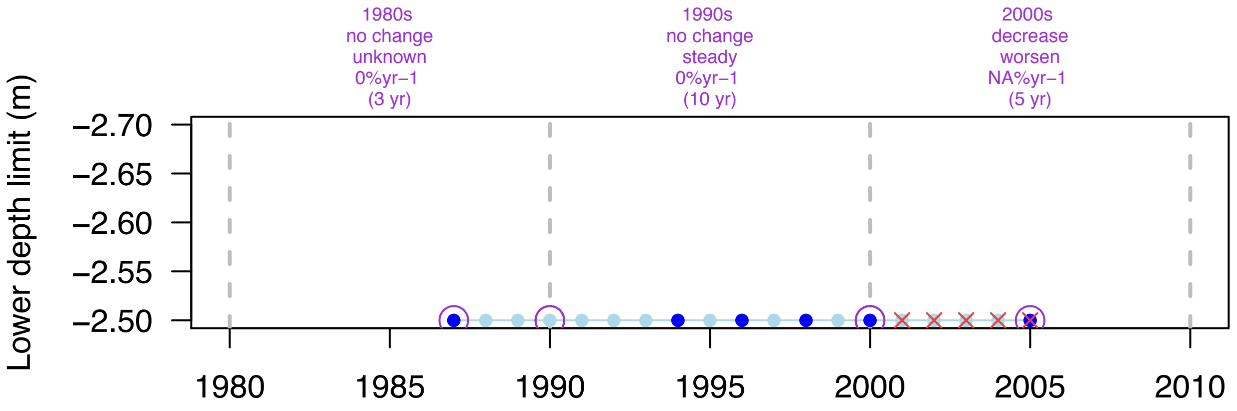

106_upperlimit

de Jong (unpublished)

SITE: Lake Veere (The Netherlands – Atlantic) – Zm (–2 m)

OVERALL: Net = NA m;   Rate = NA % yr–1; Perc Final = NA % > decrease

DECADAL: YES (18 yr)

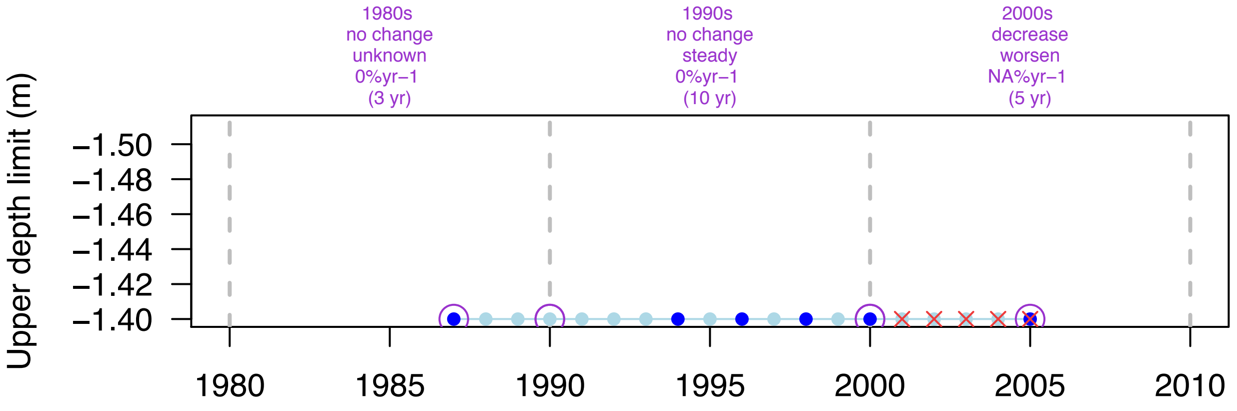

107_area

de Jong (unpublished)

SITE: Westerschelde Sloehaven (The Netherlands – Atlantic) – Zn (0.8 m)

OVERALL: Net = −3.8 ha;   Rate = −6.2 % yr−1; Perc Final = 24 % > decrease

DECADAL: YES (23 yr)

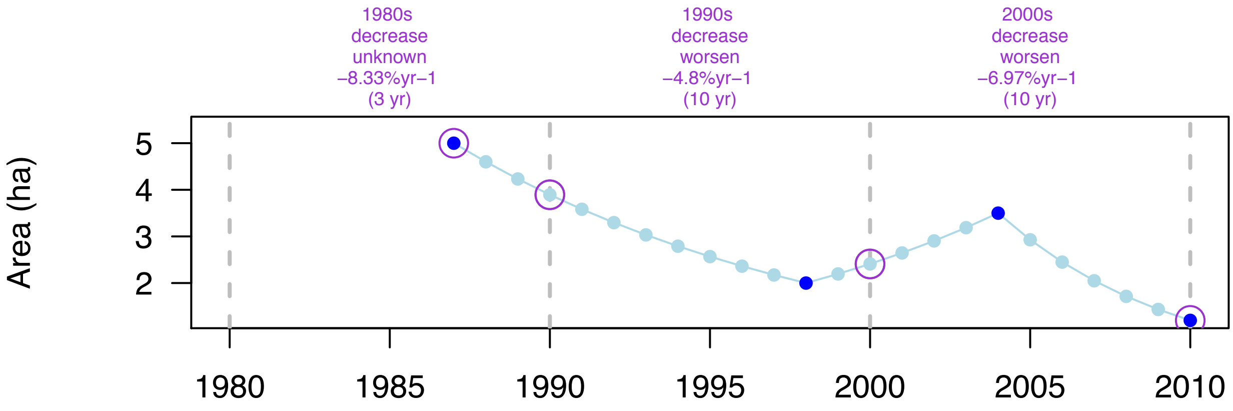

107_lowerlimit

de Jong (unpublished)

SITE: Westerschelde Sloehaven (The Netherlands – Atlantic) – Zn (0.8 m)

OVERALL: Net = 0 m;   Rate = 0 % yr−1; Perc Final = 100 % > no change

DECADAL: YES (23 yr)

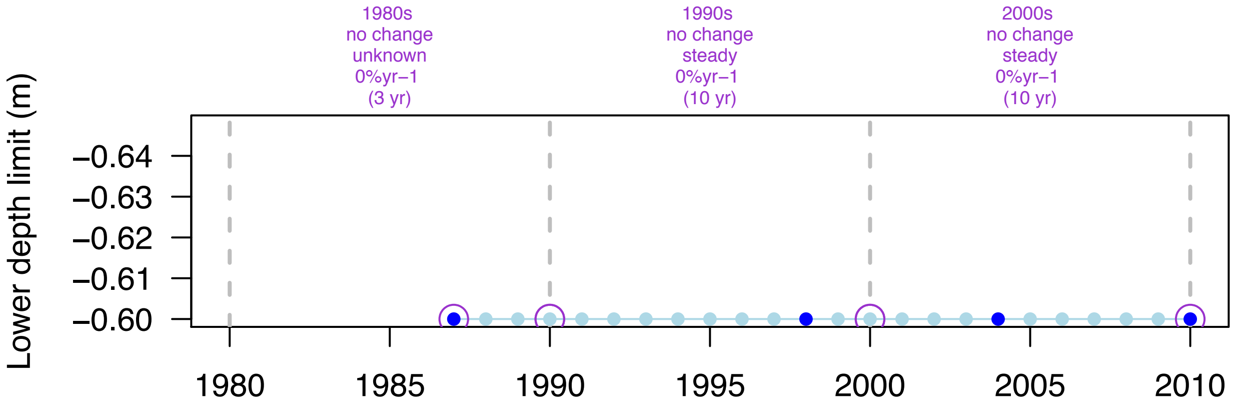

107_upperlimit

de Jong (unpublished)

SITE: Westerschelde Sloehaven (The Netherlands – Atlantic) – Zn (0.8 m)

OVERALL: Net = 0 m;   Rate = 0 % yr–1; Perc Final = 100 % > no change

DECADAL: YES (23 yr)

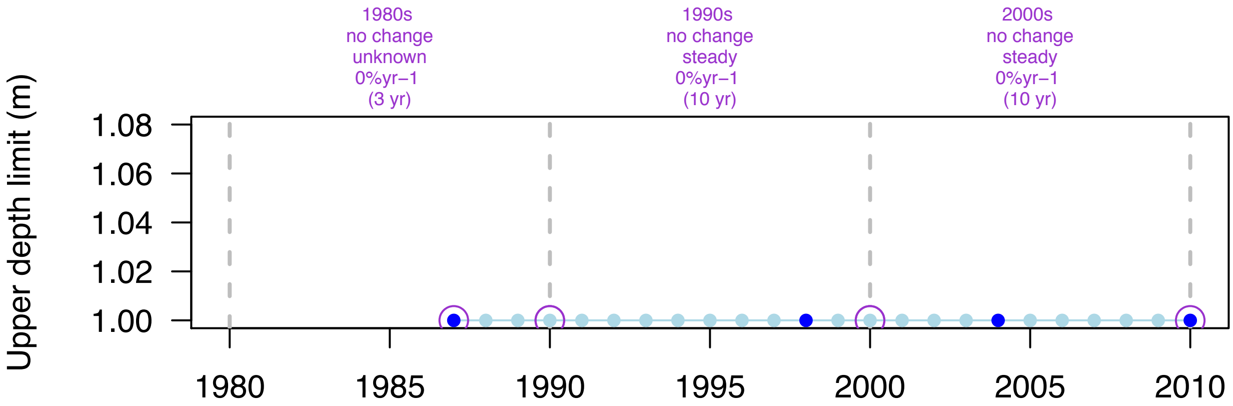

108_area

de Jong (unpublished)

SITE: Voolhok (The Netherlands – Atlantic) – Zm (0 m)

OVERALL: Net = 4.1 ha;   Rate = 36.09 % yr–1; Perc Final = 295 % > increase

DECADAL: NO (3 yr)

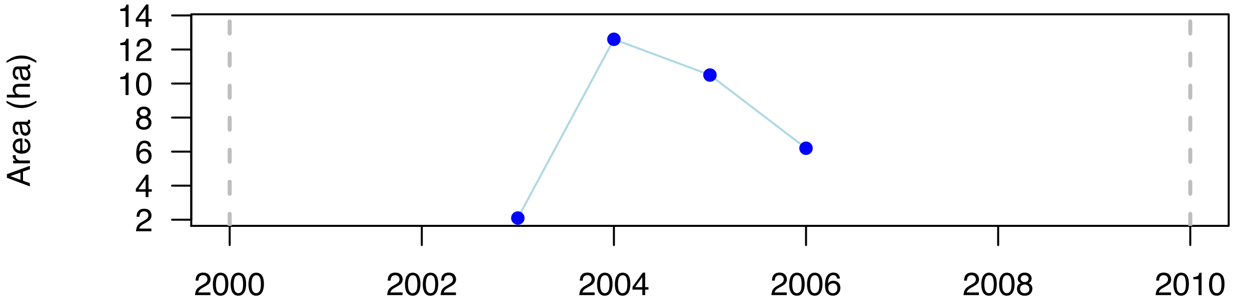

108_lowerlimit

de Jong (unpublished)

SITE: Voolhok (The Netherlands – Atlantic) – Zm (0 m)

OVERALL: Net = 0 m;   Rate = 0 % yr–1; Perc Final = 100 % > no change

DECADAL: NO (3 yr)

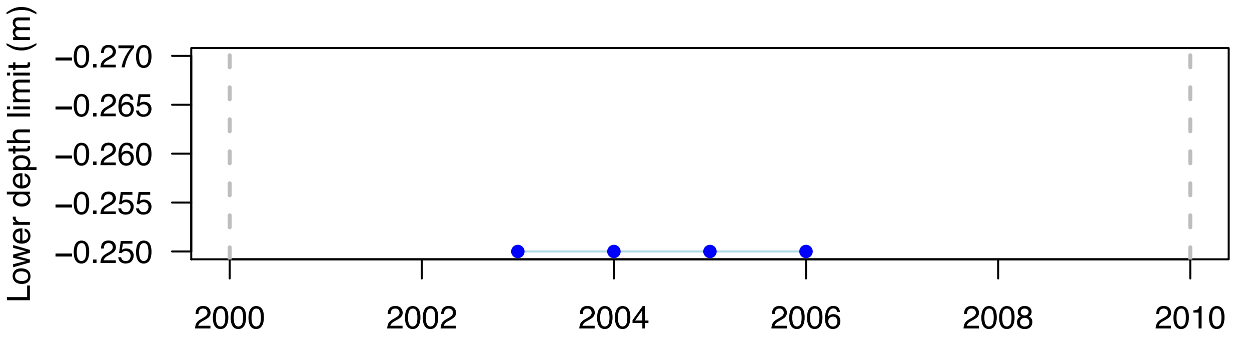

108_upperlimit

de Jong (unpublished)

SITE: Voolhok (The Netherlands – Atlantic) – Zm (0 m)

OVERALL: Net = 0 m;   Rate = 0 % yr–1; Perc Final = 100 % > no change

DECADAL: NO (3 yr)

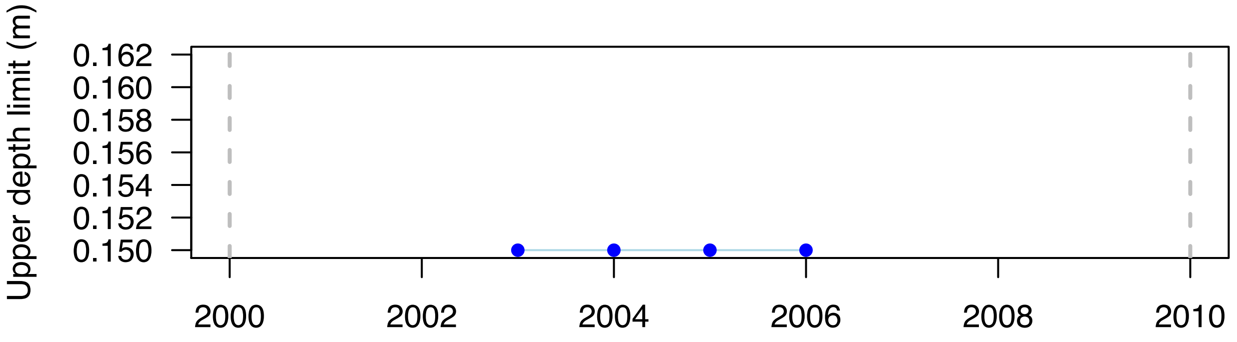

109_abiomass

Nienhuis et al. 1996, Herman et al. 1996, de Jong (unpublished)

SITE: Lake Grevelingen (The Netherlands – Atlantic) – Zm (–2 m)

OVERALL: Net = –23.97 g dw m–2;   Rate = –9.04 % yr–1; Perc Final = 16 % > decrease

DECADAL: YES (20 yr)

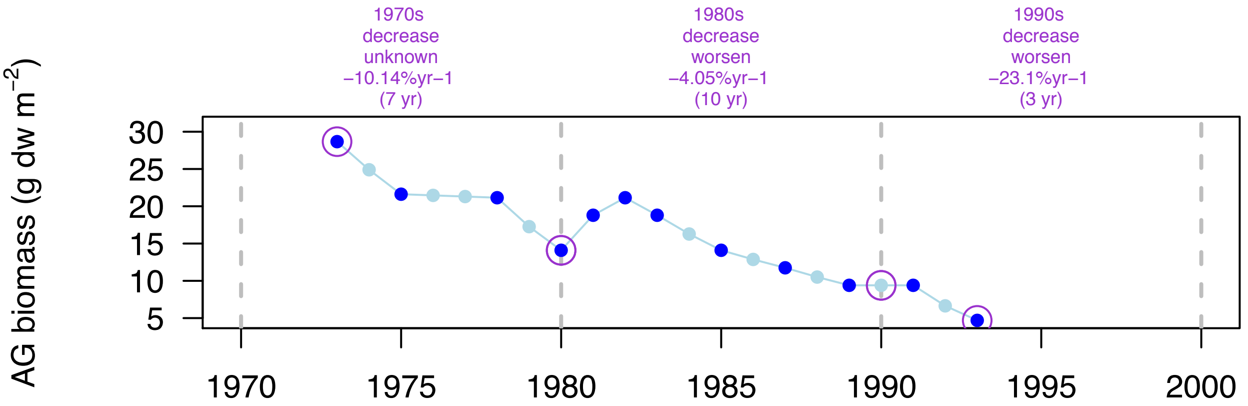

109_area

Nienhuis et al. 1996, Herman et al. 1996, de Jong (unpublished)

SITE: Lake Grevelingen (The Netherlands – Atlantic) – Zm (–2 m)

OVERALL: Net = –1357.9 ha;   Rate = NA % yr–1; Perc Final = NA % > decrease

DECADAL: YES (31 yr)

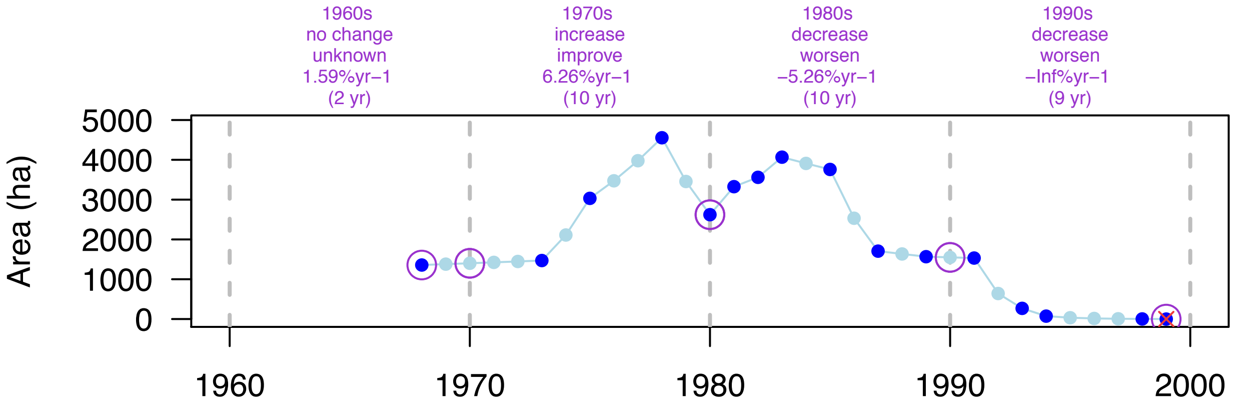

109_lowerlimit

Nienhuis et al. 1996, Herman et al. 1996, de Jong (unpublished)

SITE: Lake Grevelingen (The Netherlands – Atlantic) – Zm (–2 m)

OVERALL: Net = NA m; Rate = NA % yr–1; Perc Final = NA % > decrease

DECADAL: YES (26 yr)

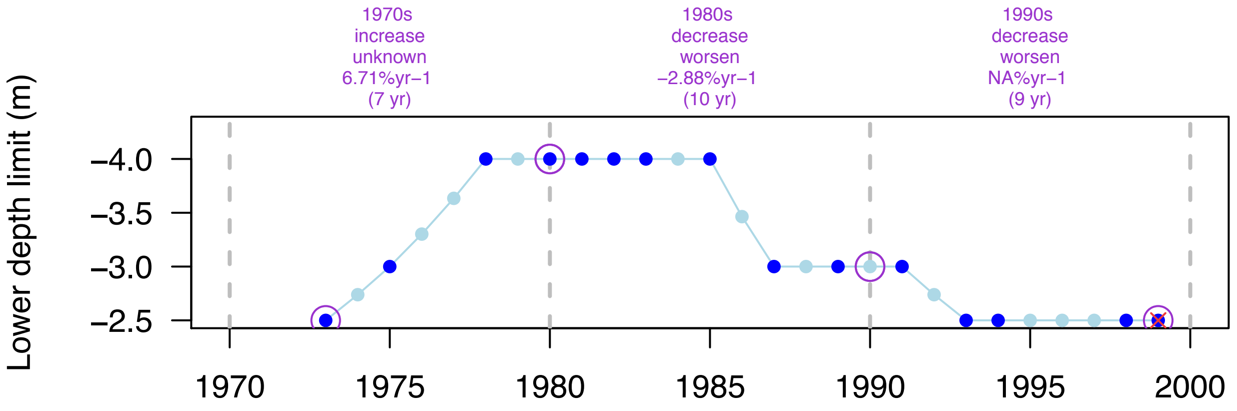

109_upperlimit

Nienhuis et al. 1996, Herman et al. 1996, de Jong (unpublished)

SITE: Lake Grevelingen (The Netherlands – Atlantic) – Zm (–2 m)

OVERALL: Net = NA m; Rate = NA % yr–1; Perc Final = NA % > decrease

DECADAL: YES (26 yr)

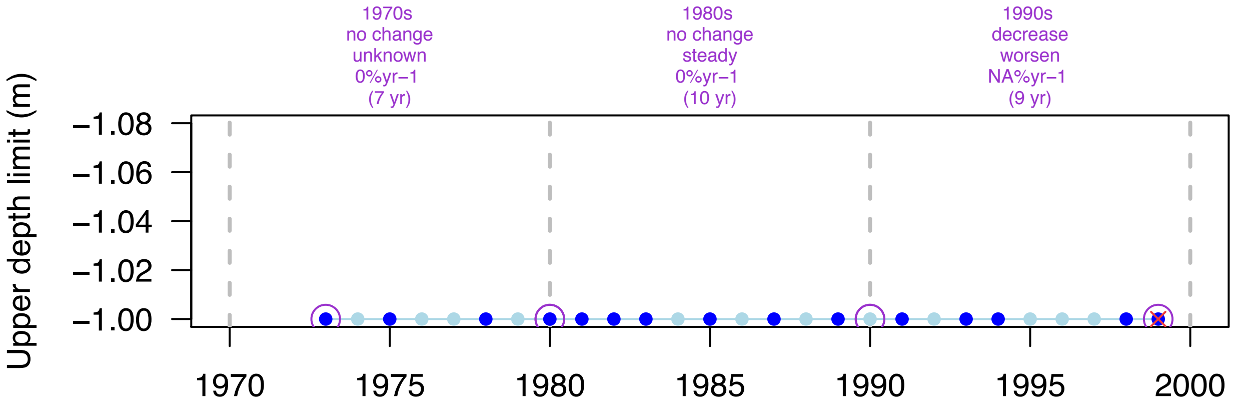

110_area

Valle et al. 2013

SITE: Hond Paap (The Netherlands – Atlantic) – Zm (0 m)

OVERALL: Net = −13.1 ha;   Rate = NA % yr−1; Perc Final = NA % > decrease

DECADAL: YES (20 yr)

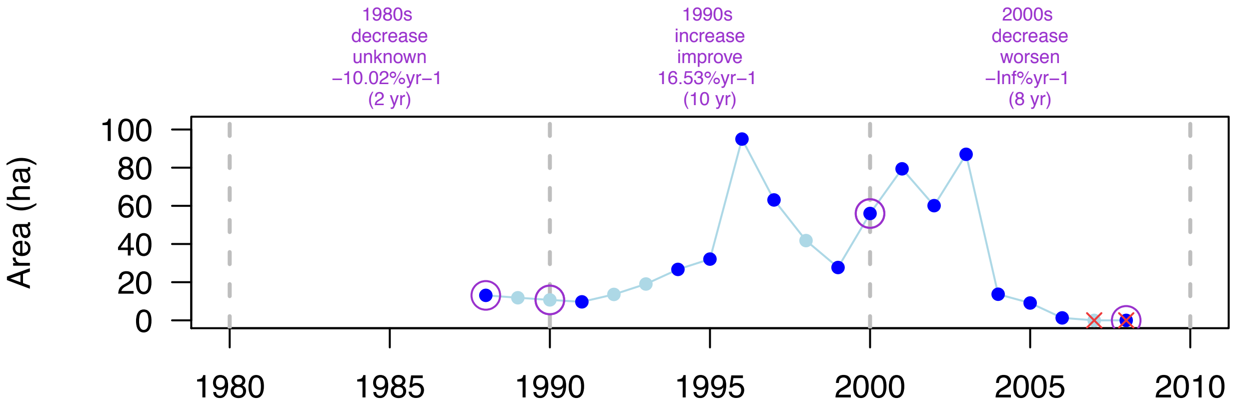

110_lowerlimit

Valle et al. 2013

SITE: Hond Paap (The Netherlands – Atlantic) – Zm (0 m)

OVERALL: Net = NA m;   Rate = NA % yr−1; Perc Final = NA % > decrease

DECADAL: YES (20 yr)

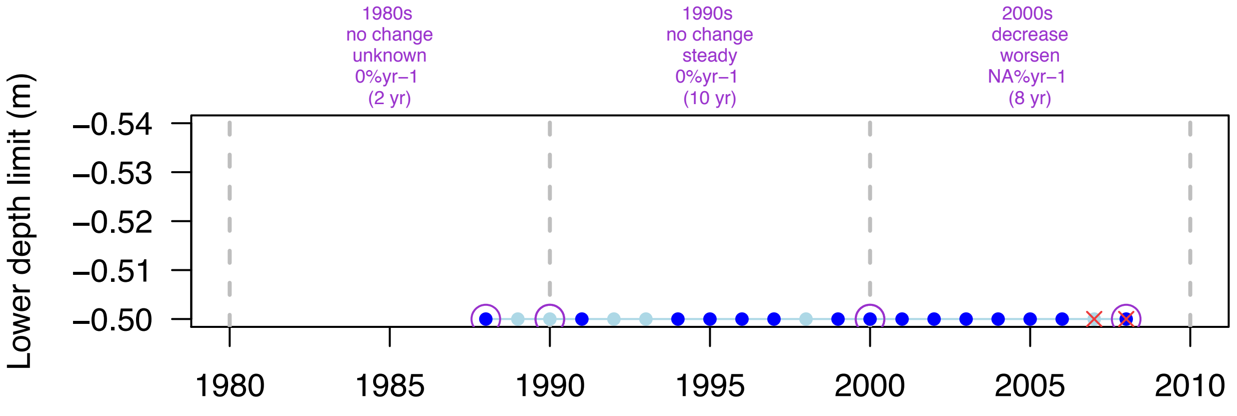

110_upperlimit

Valle et al. 2013

SITE: Hond Paap (The Netherlands – Atlantic) – Zm (0 m)

OVERALL: Net = NA m;   Rate = NA % yr–1; Perc Final = NA % > decrease

DECADAL: YES (20 yr)

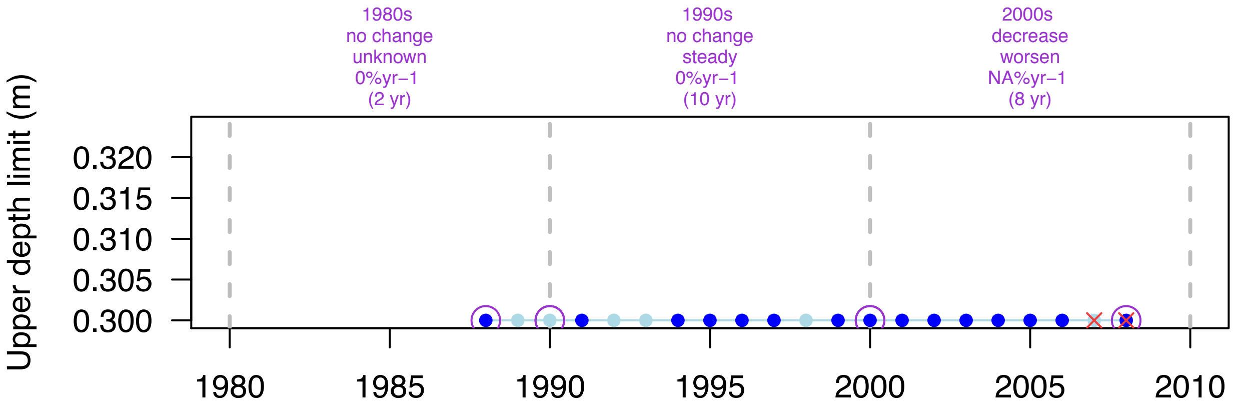

111_area

van Katwijk et al. 2010

SITE: Terschelling Haven (The Netherlands – Atlantic) – Zm (–0.3 m)

OVERALL: Net = –8.2 ha;   Rate = NA % yr–1; Perc Final = NA % > decrease

DECADAL: YES (13 yr)

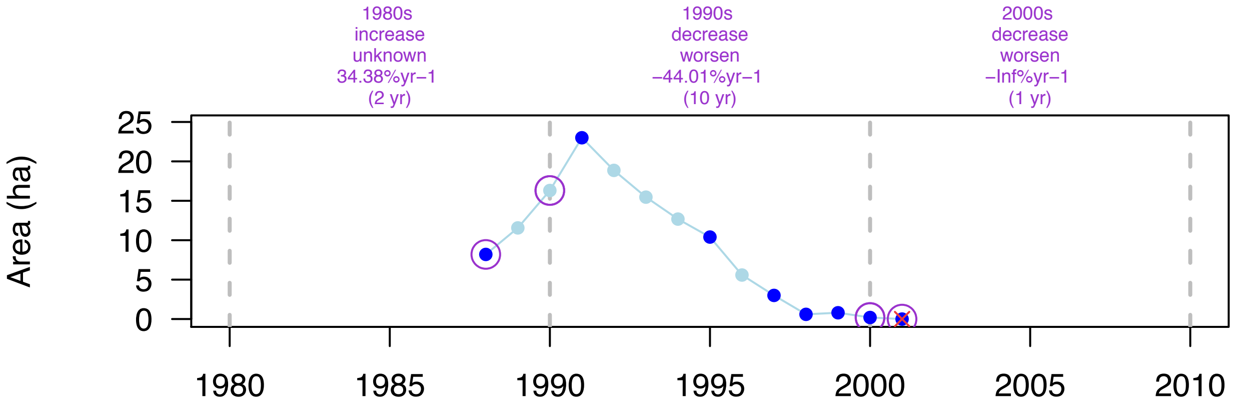

111_lowerlimit

van Katwijk et al. 2010

SITE: Terschelling Haven (The Netherlands – Atlantic) – Zm (–0.3 m)

OVERALL: Net = NA m;   Rate = NA % yr–1; Perc Final = NA % > decrease

DECADAL: YES (13 yr)

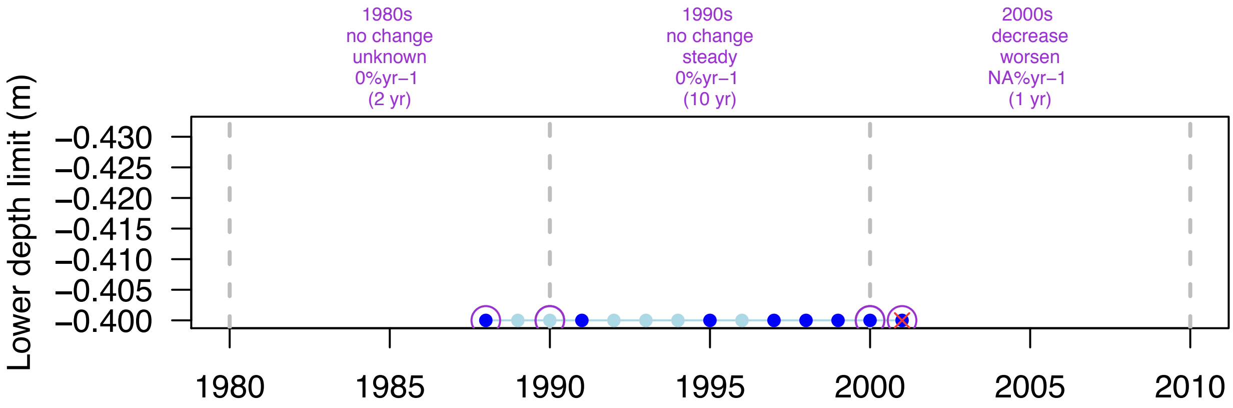

111_upperlimit

van Katwijk et al. 2010

SITE: Terschelling Haven (The Netherlands – Atlantic) – Zm (–0.3 m)

OVERALL: Net = NA m;   Rate = NA % yr–1; Perc Final = NA % > decrease

DECADAL: YES (13 yr)

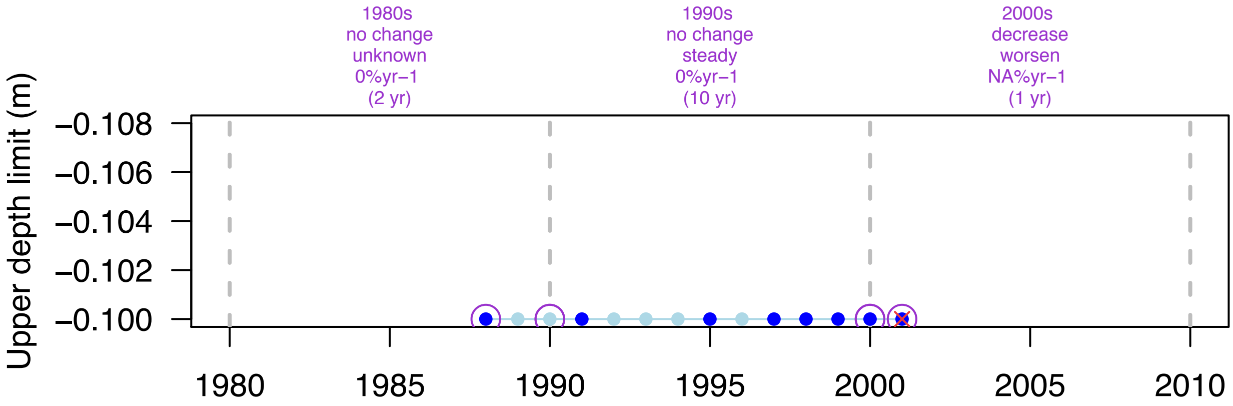

112_area

Polderman and den Hartog 1975, Braster and Carrière 1976, de Jong (unpublished)

SITE: Terschelling Hoorn (The Netherlands – Atlantic) – Zn (0.1 m)

OVERALL: Net = −11.2 ha;   Rate = −3.38 % yr−1; Perc Final = 32 % > decrease

DECADAL: YES (34 yr)

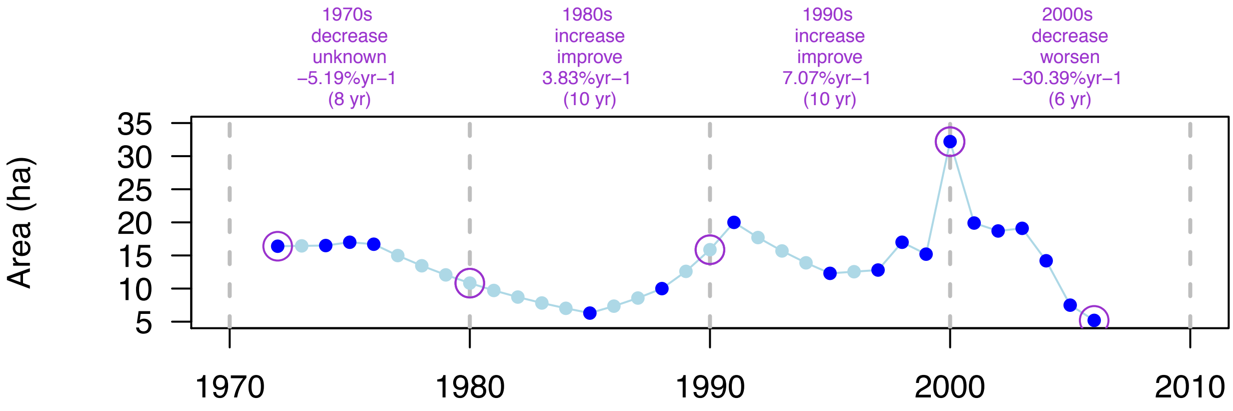

112_lowerlimit

Polderman and den Hartog 1975, Braster and Carrière 1976, de Jong (unpublished)

SITE: Terschelling Hoorn (The Netherlands – Atlantic) – Zn (0.1 m)

OVERALL: Net = 0 m;   Rate = 0 % yr−1; Perc Final = 100 % > no change

DECADAL: YES (34 yr)

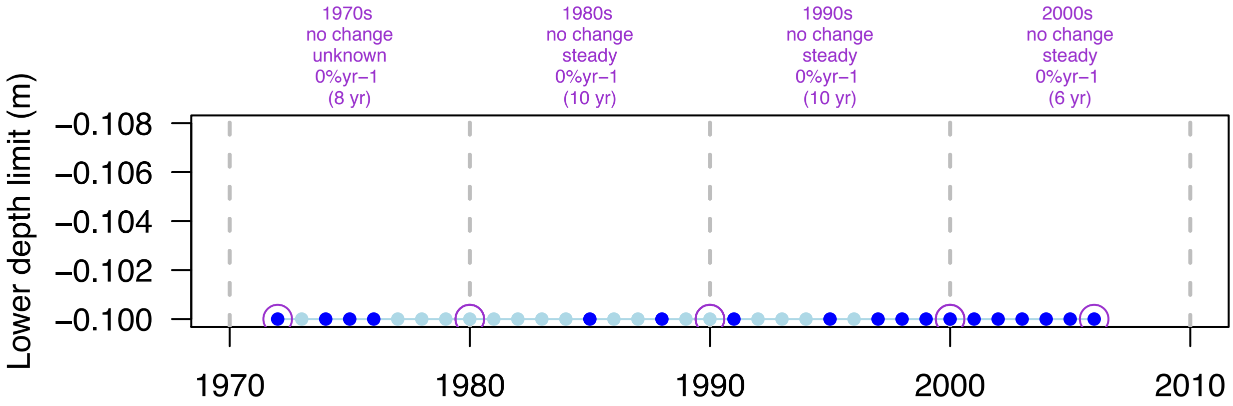

112_upperlimit

Polderman and den Hartog 1975, Braster and Carrière 1976, de Jong (unpublished)

SITE: Terschelling Hoorn (The Netherlands – Atlantic) – Zn (0.1 m)

OVERALL: Net = 0 m; Rate = 0 % yr–1; Perc Final = 100 % > no change

DECADAL: YES (34 yr)

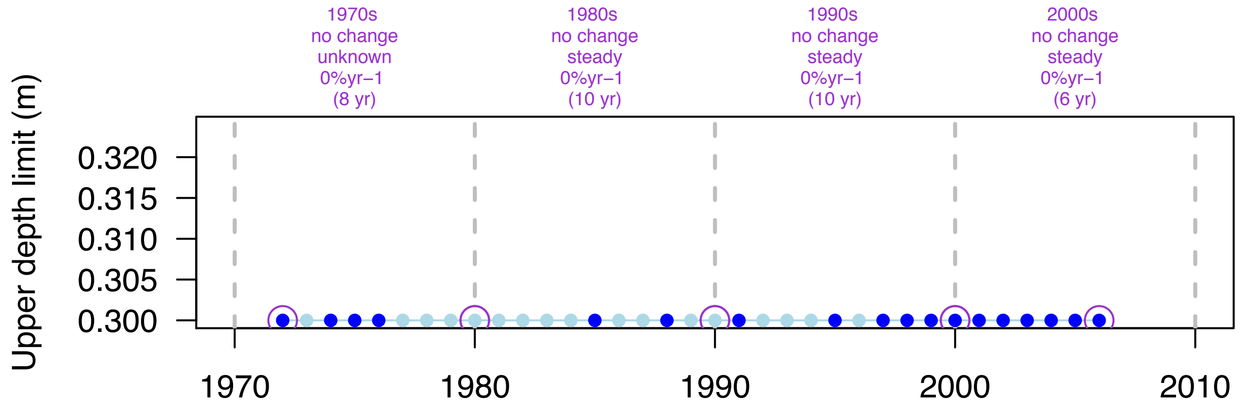

113_density

Marbà and Duarte 2010, Duarte and Marbà (unpublished)

SITE: El Castell (Spain – Mediterranean) – Po (–15 m)

OVERALL: Net = –262.5 shoot m–2; Rate = –8.33 % yr–1; Perc Final = 40 % > decrease

DECADAL: YES (11 yr)

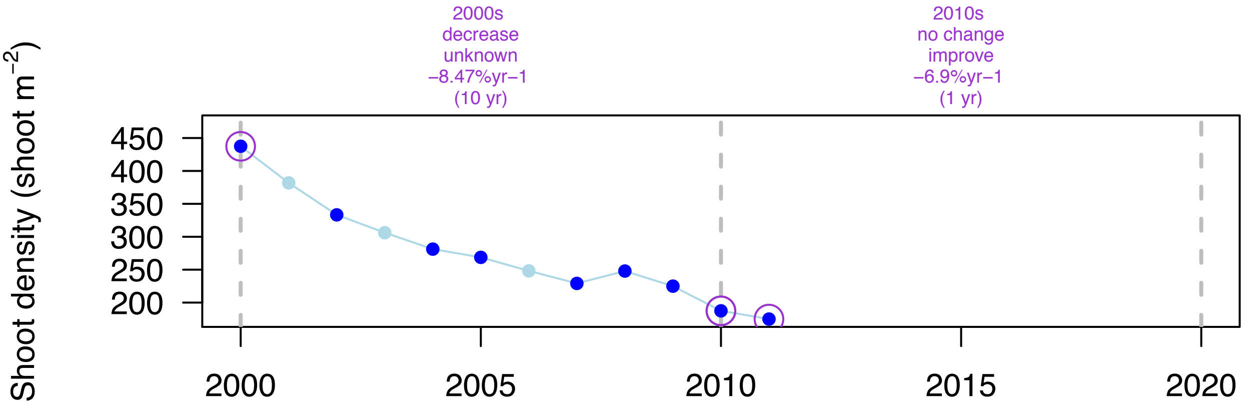

114_area

de Jong (unpublished)

SITE: Terschelling Keeg Ans (The Netherlands – Atlantic) – Zn (0.1 m)

OVERALL: Net = −2.7 ha;   Rate = NA % yr−1; Perc Final = NA % > decrease

DECADAL: NO (5 yr)

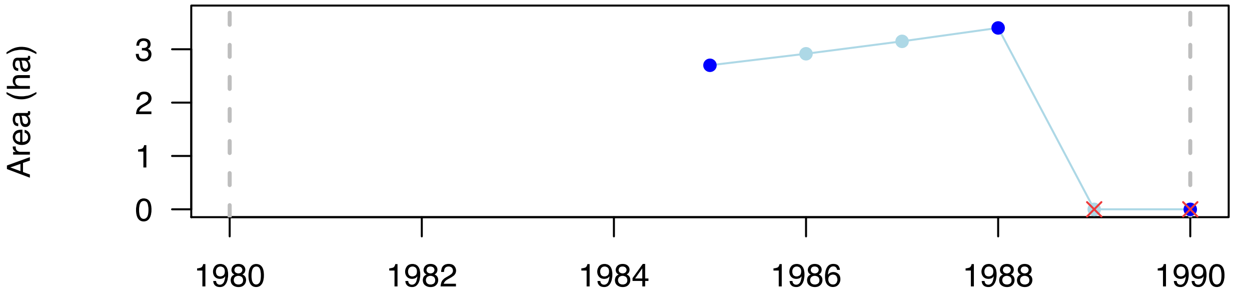

114_lowerlimit

de Jong (unpublished)

SITE: Terschelling Keeg Ans (The Netherlands – Atlantic) – Zn (0.1 m)

OVERALL: Net = NA m;   Rate = NA % yr−1; Perc Final = NA % > decrease

DECADAL: NO (5 yr)

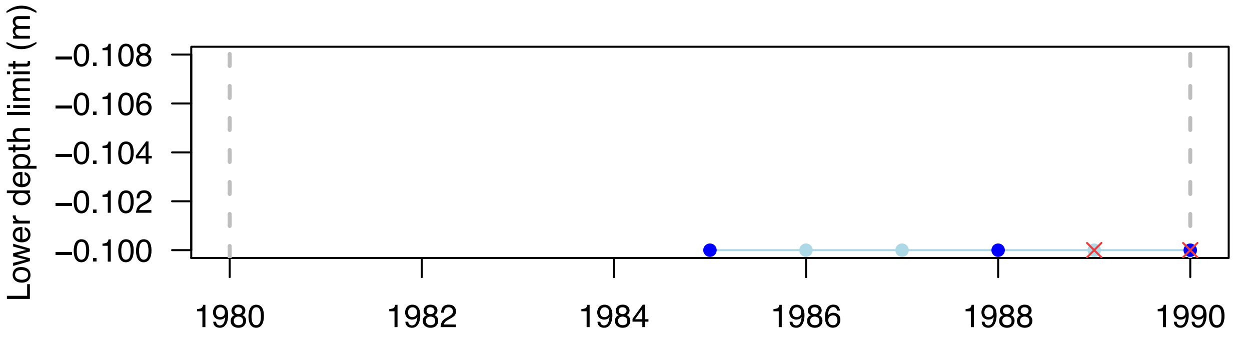

114_upperlimit

de Jong (unpublished)

SITE: Terschelling Keeg Ans (The Netherlands – Atlantic) – Zn (0.1 m)

OVERALL: Net = NA m;   Rate = NA % yr–1; Perc Final = NA % > decrease

DECADAL: NO (5 yr)

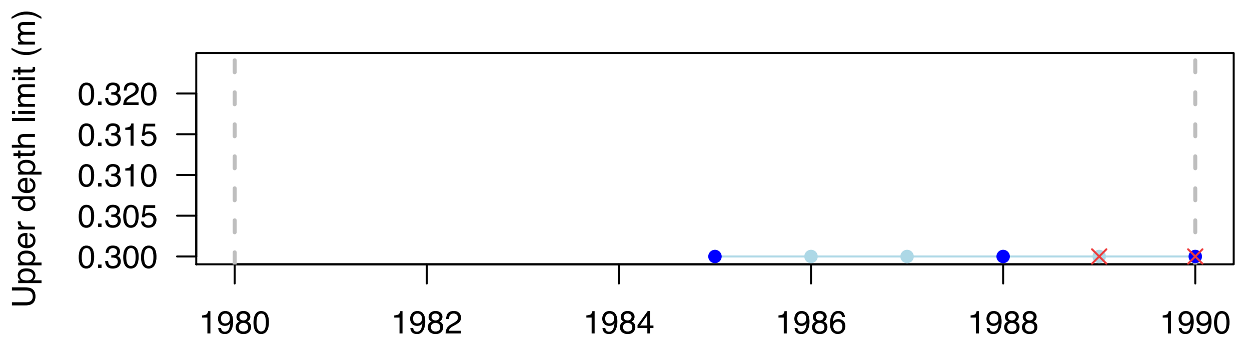

115_density

Marbà and Duarte 2010, Duarte and Marbà (unpublished)

SITE: El Castell (Spain – Mediterranean) – Po (–10 m)

OVERALL: Net = –362.5 shoot m–2;   Rate = –10.27 % yr–1; Perc Final = 32 % > decrease

DECADAL: YES (11 yr)

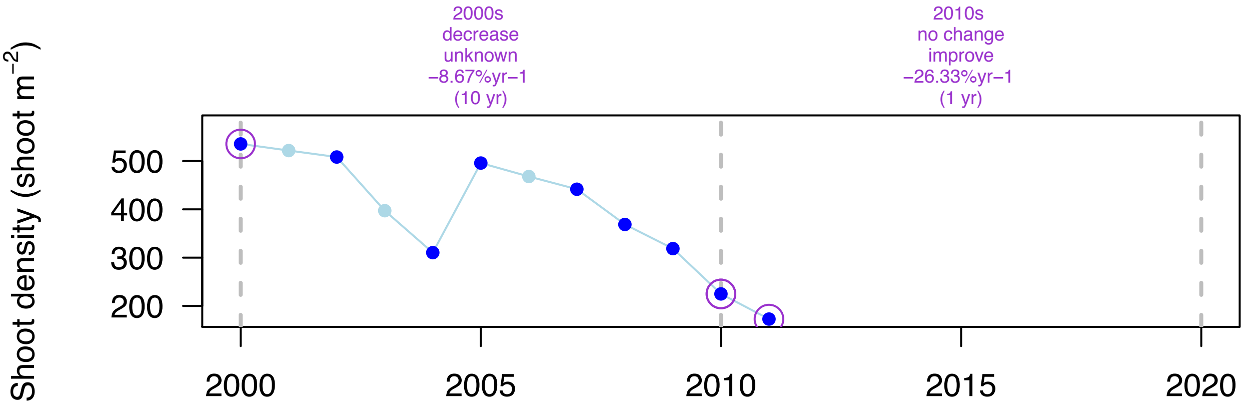

116_biomass

Peralta et al. (unpublished)

SITE: Santibañez (Spain – Atlantic) – Cn (–0.1 m)

OVERALL: Net = 0.73 g dw m–2;   Rate = 0.03 % yr–1; Perc Final = 100 % > no change

DECADAL: NO (7 yr)

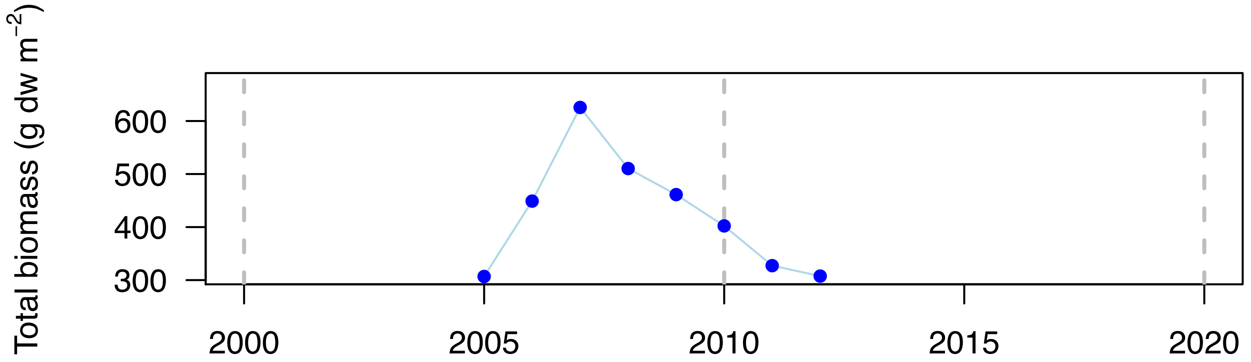

116_density

Peralta et al. (unpublished)

SITE: Santibañez (Spain – Atlantic) – Cn (–0.1 m)

OVERALL: Net = 537.5 shoot m–2;   Rate = 9.76 % yr–1; Perc Final = 180 % > increase

DECADAL: NO (6 yr)

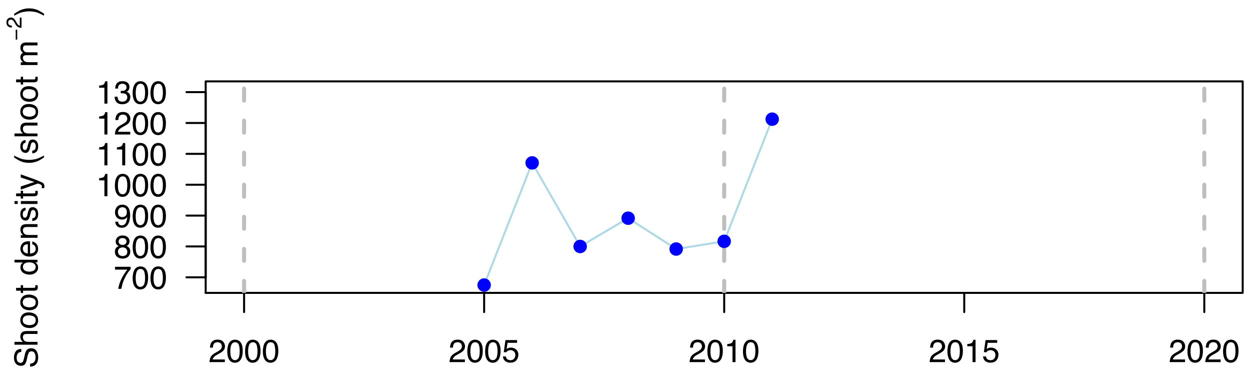

117_biomass

Peralta et al. (unpublished)

SITE: Santibañez (Spain – Atlantic) – Cn (–0.5 m)

OVERALL: Net = –90.12 g dw m–2;   Rate = –3.88 % yr–1; Perc Final = 76 % > no change

DECADAL: NO (7 yr)

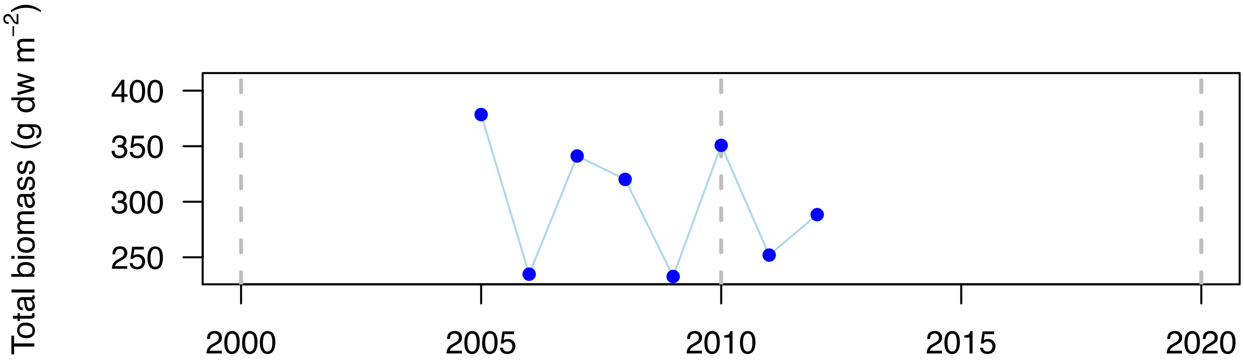

117_density

Peralta et al. (unpublished)

SITE: Santibañez (Spain – Atlantic) – Cn (–0.5 m)

OVERALL: Net = –168.75 shoot m–2;   Rate = –6.19 % yr–1; Perc Final = 69 % > decrease

DECADAL: NO (6 yr)

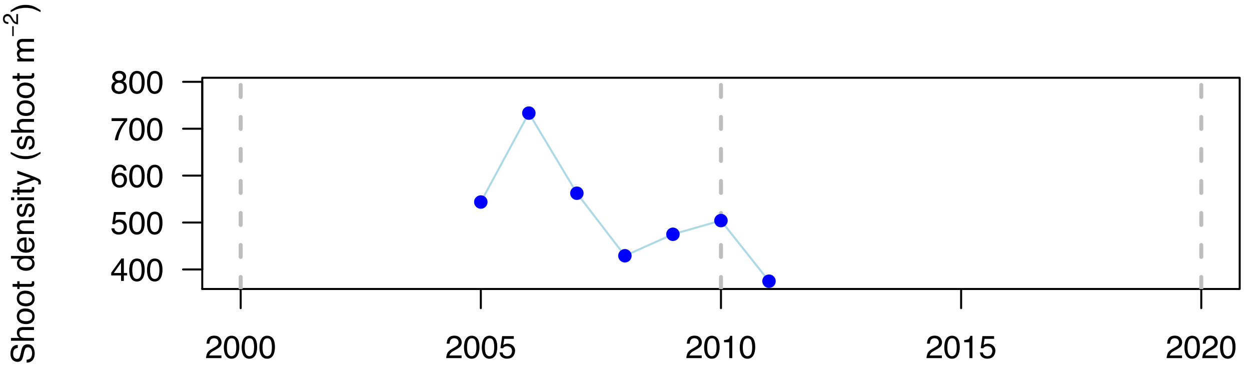

118_biomass

Peralta et al. (unpublished)

SITE: Santibañez (Spain – Atlantic) – Cn (0.4 m)

OVERALL: Net = −77.88 g dw m−2; Rate = −3.2 % yr−1; Perc Final = 80 % > no change

DECADAL: NO (7 yr)

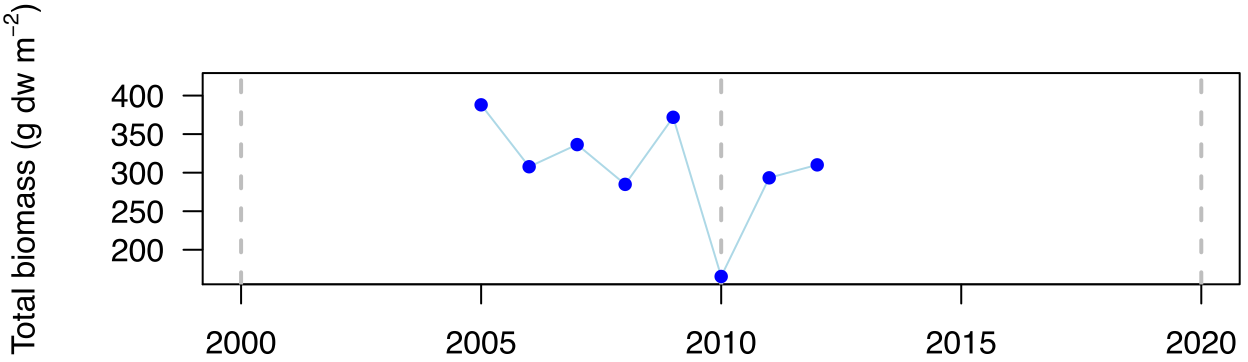

118_density

Peralta et al. (unpublished)

SITE: Santibañez (Spain – Atlantic) – Cn (0.4 m)

OVERALL: Net = 1289.58 shoot m−2; Rate = 13.75 % yr−1; Perc Final = 228 % > increase

DECADAL: NO (6 yr)

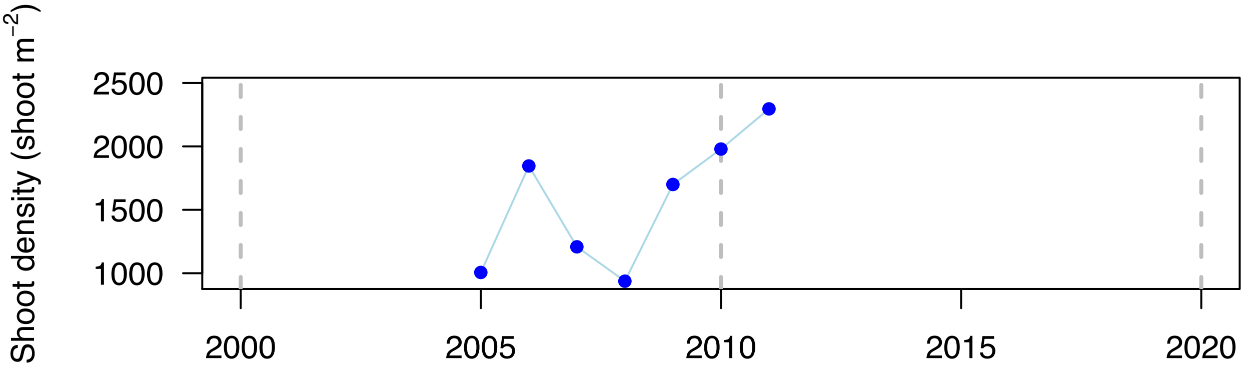

119_area

del Rei 2009

SITE: Inner Cádiz Bay (Spain – Atlantic) – Zn (? m)

OVERALL: Net = 38.1 ha;   Rate = 3.58 % yr–1; Perc Final = 107 % > no change

DECADAL: NO (2 yr)

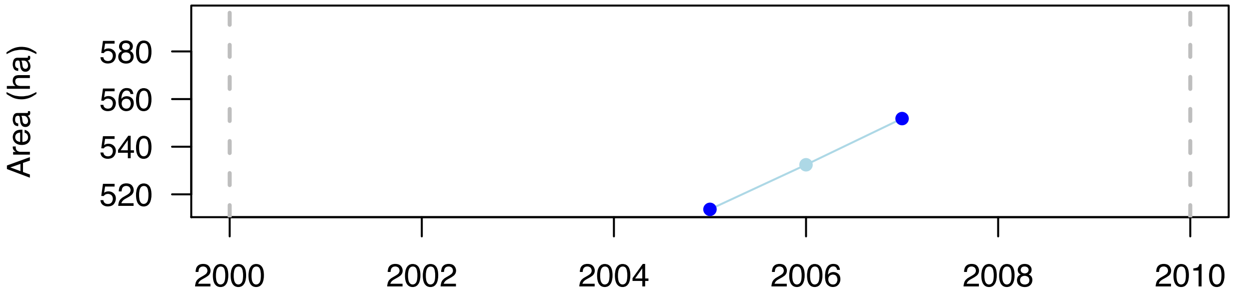

120_area

Muñoz–Ramos and Seglar (unpublished)

SITE: Mataró (Spain – Mediterranean) – Po (–16.2 m)

OVERALL: Net = –188.4 ha;   Rate = –4.72 % yr–1; Perc Final = 69 % > decrease

DECADAL: YES (8 yr)

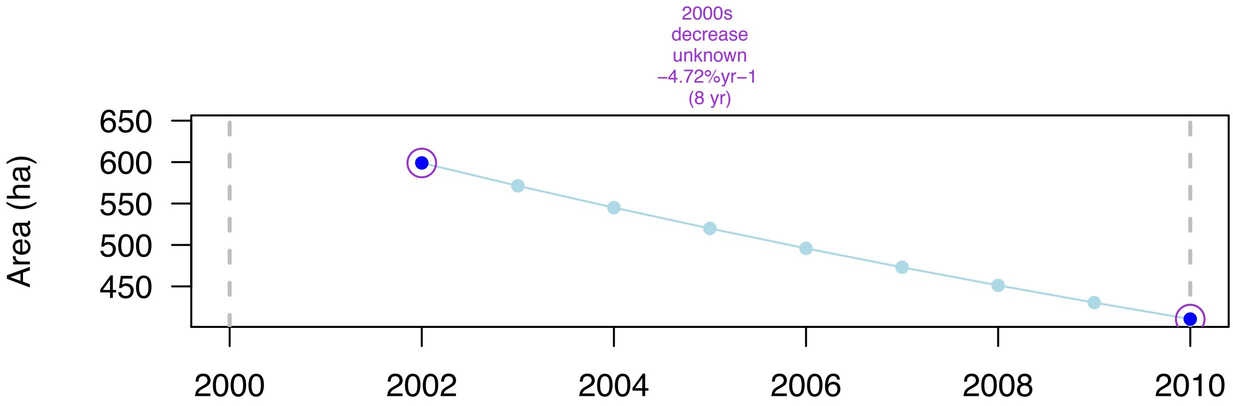

121_area

Muñoz–Ramos and Seglar (unpublished)

SITE: Mataró (Spain – Mediterranean) – Cn (–16.2 m)

OVERALL: Net = –118 ha; Rate = –27.28 % yr–1; Perc Final = 11 % > decrease

DECADAL: YES (8 yr)

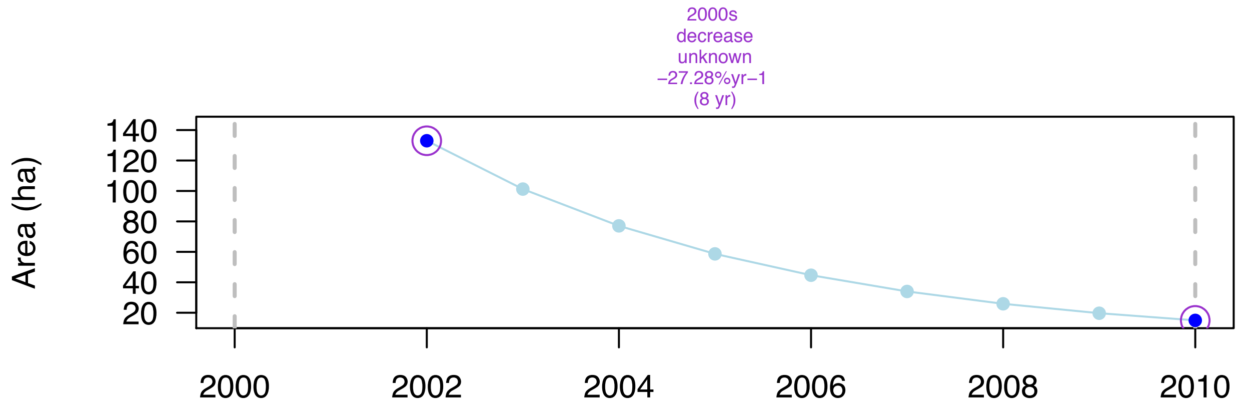

122_cover

Muñoz–Ramos and Seglar 2015

SITE: Estació Mataró I (Spain – Mediterranean) – Po (–12.5 m)

OVERALL: Net = –8.2 %; Rate = –1.33 % yr–1; Perc Final = 79 % > no change

DECADAL: YES (18 yr)

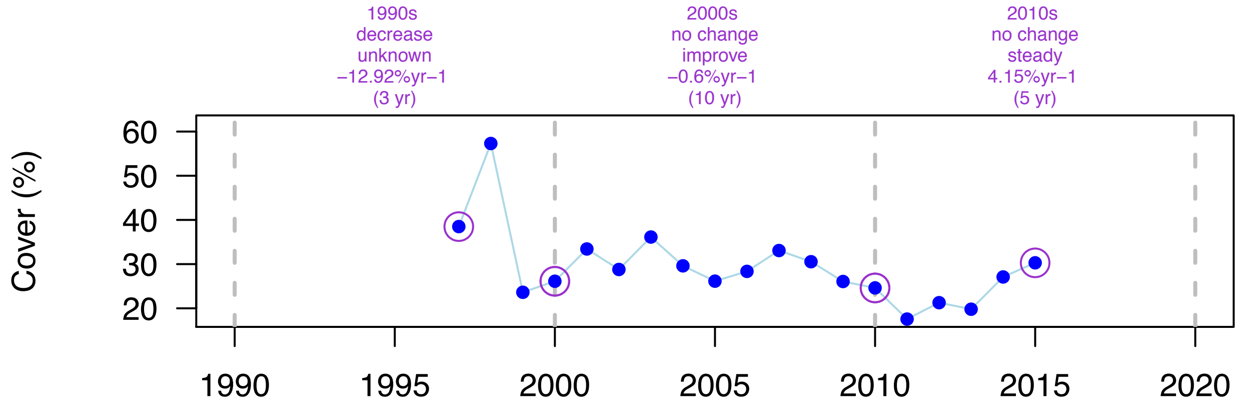

122_density

Muñoz–Ramos and Seglar 2015

SITE: Estació Mataró I (Spain – Mediterranean) – Po (–12.5 m)

OVERALL: Net = 116.65 shoot m–2;   Rate = 1.64 % yr–1; Perc Final = 134 % > increase

DECADAL: YES (18 yr)

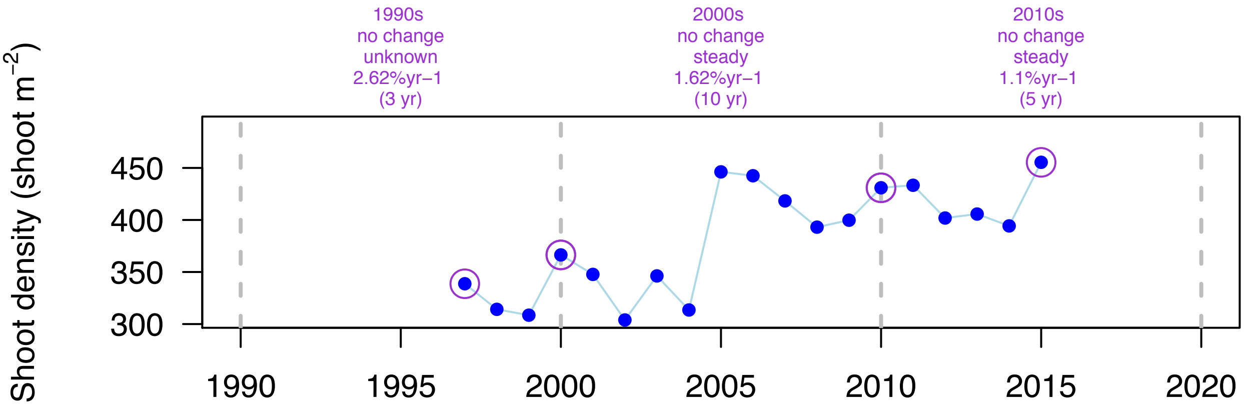

123_cover

Muñoz–Ramos and Seglar 2015

SITE: Estació Mataró III (Spain – Mediterranean) – Po (–17 m)

OVERALL: Net = 13.01 %;   Rate = 8.44 % yr–1; Perc Final = 152 % > increase

DECADAL: NO (5 yr)

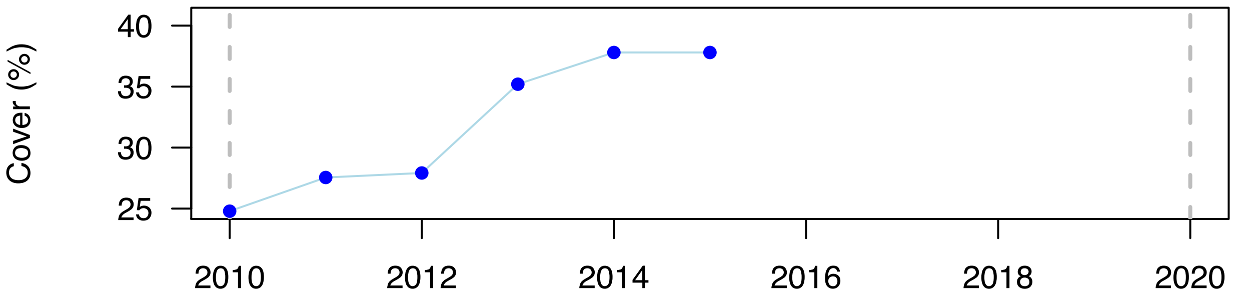

123_density

Muñoz–Ramos and Seglar 2015

SITE: Estació Mataró III (Spain – Mediterranean) – Po (–17 m)

OVERALL: Net = 10.79 shoot m–2;   Rate = 0.77 % yr–1; Perc Final = 104 % > no change

DECADAL: NO (5 yr)

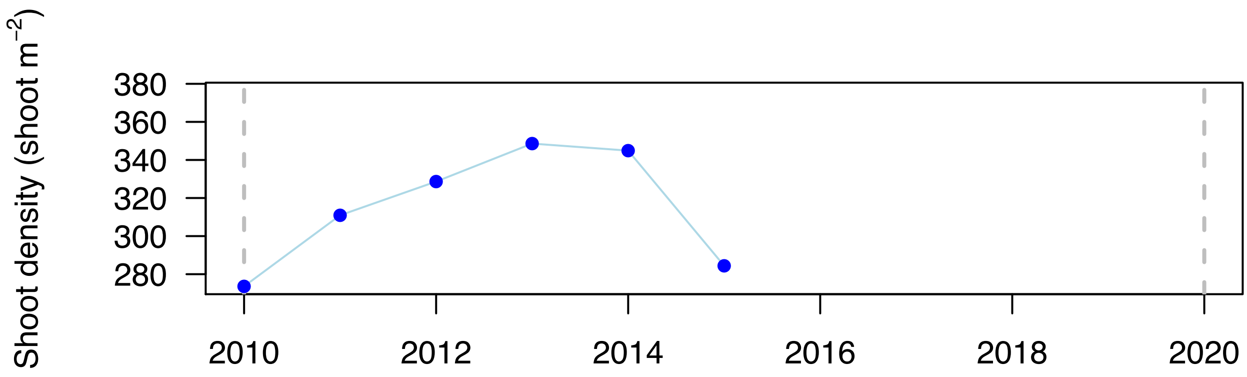

124_cover

Muñoz–Ramos and Seglar 2015

SITE: Estació Mataró III (Spain – Mediterranean) – Po (–19 m)

OVERALL: Net = –2.82 %;   Rate = –0.46 % yr–1; Perc Final = 92 % > no change

DECADAL: YES (17 yr)

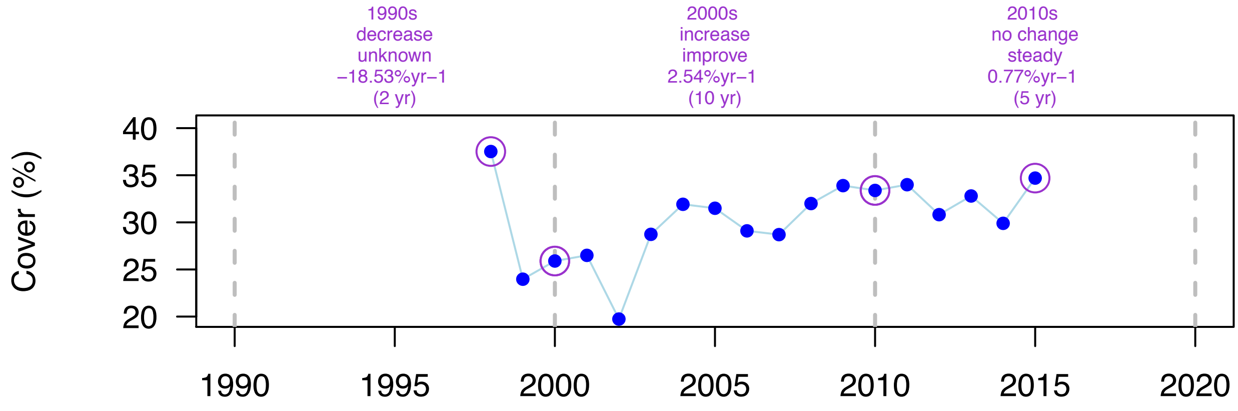

124_density

Muñoz–Ramos and Seglar 2015

SITE: Estació Mataró III (Spain – Mediterranean) – Po (–19 m)

OVERALL: Net = –51.5 shoot m–2;   Rate = –1.4 % yr–1; Perc Final = 80 % > no change

DECADAL: YES (16 yr)

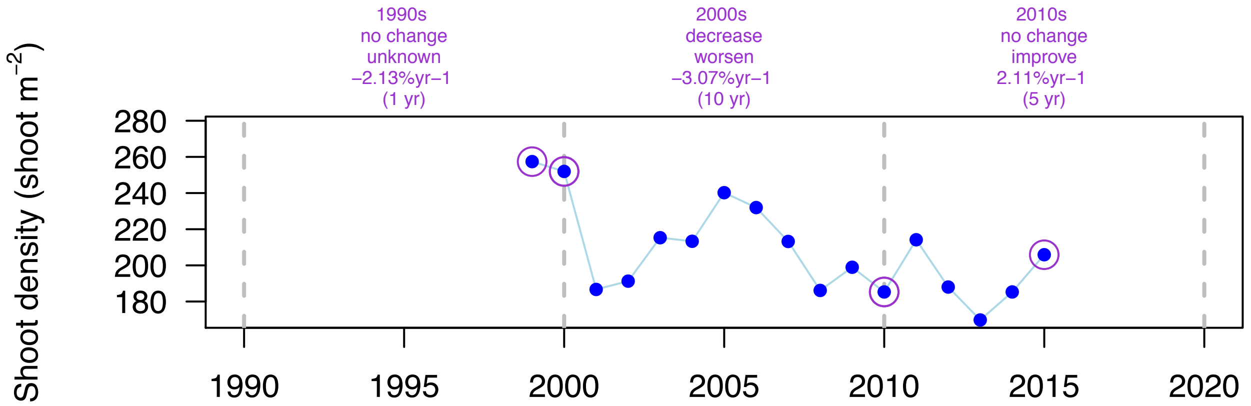

125_density

Roca et al. 2014

SITE: Marimurtra (control) (Spain – Mediterranean) – Po (–13 m)

OVERALL: Net = 10.42 shoot m–2;   Rate = 1.64 % yr–1; Perc Final = 103 % > no change

DECADAL: NO (2 yr)

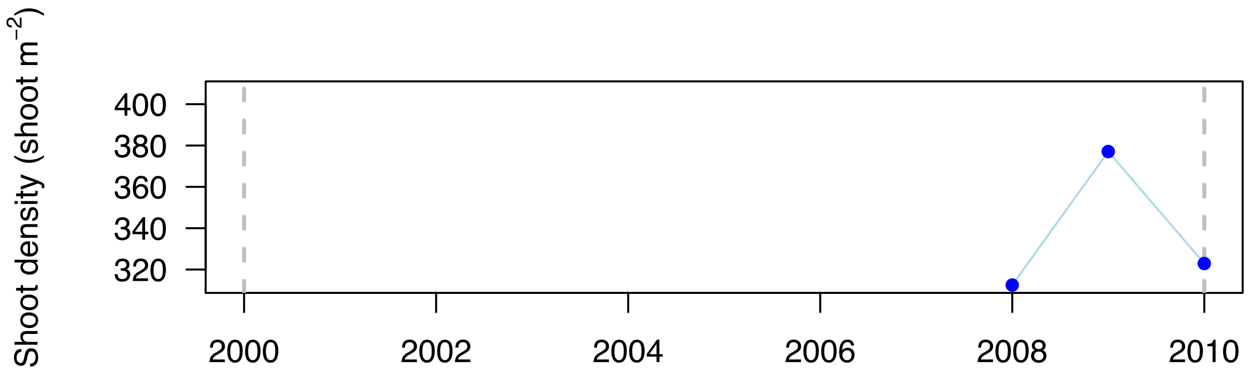

126_density

Roca et al. 2014

SITE: Port Blanes (impacted) (Spain – Mediterranean) – Po (–14 m)

OVERALL: Net = –152.09 shoot m–2;   Rate = –26.79 % yr–1; Perc Final = 59 % > decrease

DECADAL: NO (2 yr)

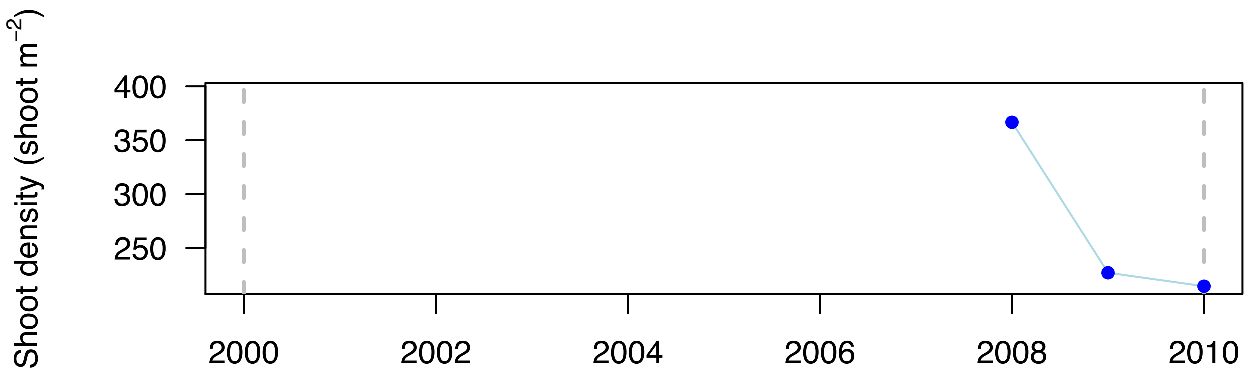

127_area

Mascaró 2011

SITE: Alfacs Bay (Spain – Mediterranean) – Cn (–1 m)

OVERALL: Net = 304 ha;   Rate = NA % yr–1; Perc Final = NA % > increase

DECADAL: YES (39 yr)

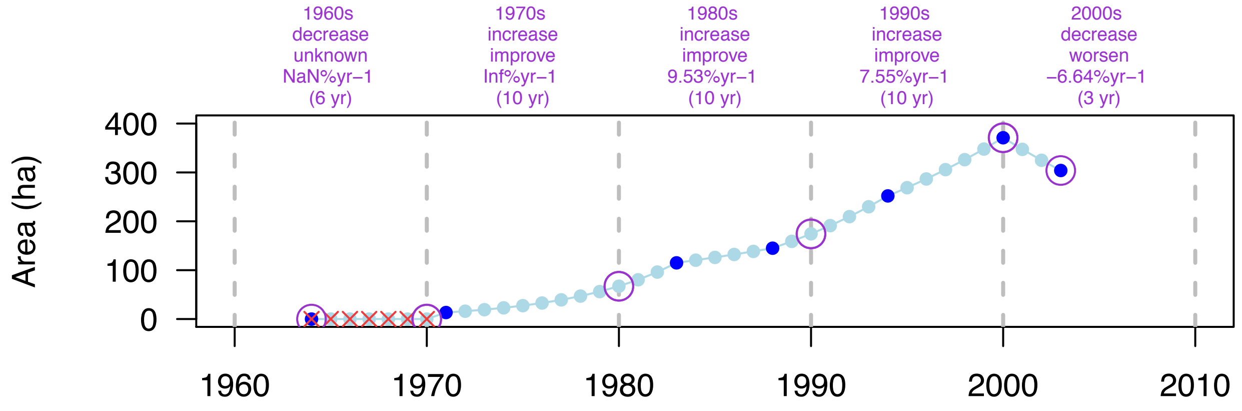

128_area

Mascaró 2011

SITE: Fangar Bay (Spain – Mediterranean) – Cn (–1 m)

OVERALL: Net = 49 ha;   Rate = NA % yr–1; Perc Final = NA % > increase

DECADAL: YES (36 yr)

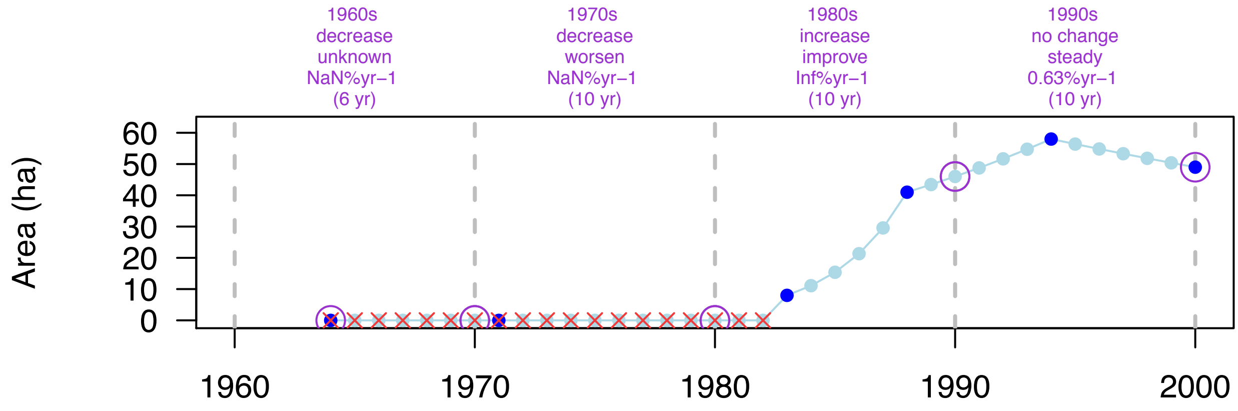

129_biomass

Romero et al. 2010 (b)

SITE: Alfacs (eutrophic) (Spain – Mediterranean) – Cn (–1 m)

OVERALL: Net = 20.5 g dw m–2;   Rate = 2.1 % yr–1; Perc Final = 111 % > no change

DECADAL: NO (5 yr)

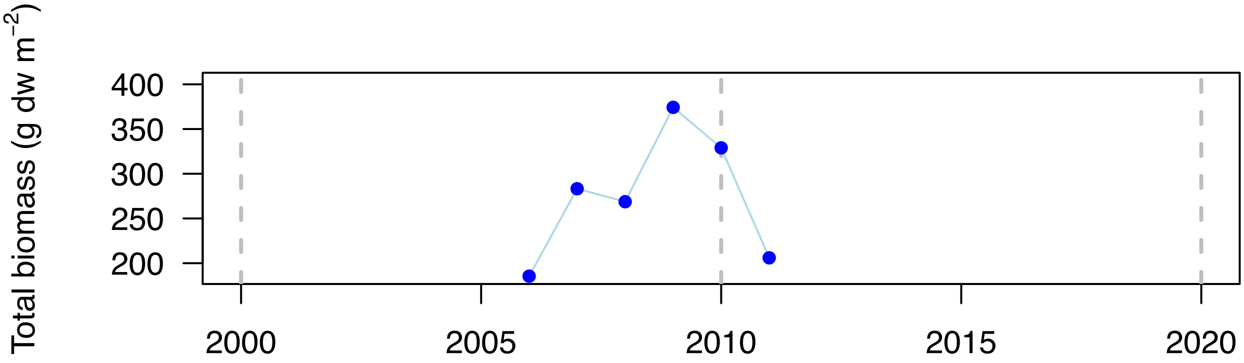

129_density
Romero et al. 2010 (b)
SITE: Alfacs (eutrophic) (Spain – Mediterranean) – Cn (–1 m)
OVERALL: Net = –1227.75 shoot m–2;   Rate = –13.61 % yr–1; Perc Final = 51 % > decrease
DECADAL: NO (5 yr)

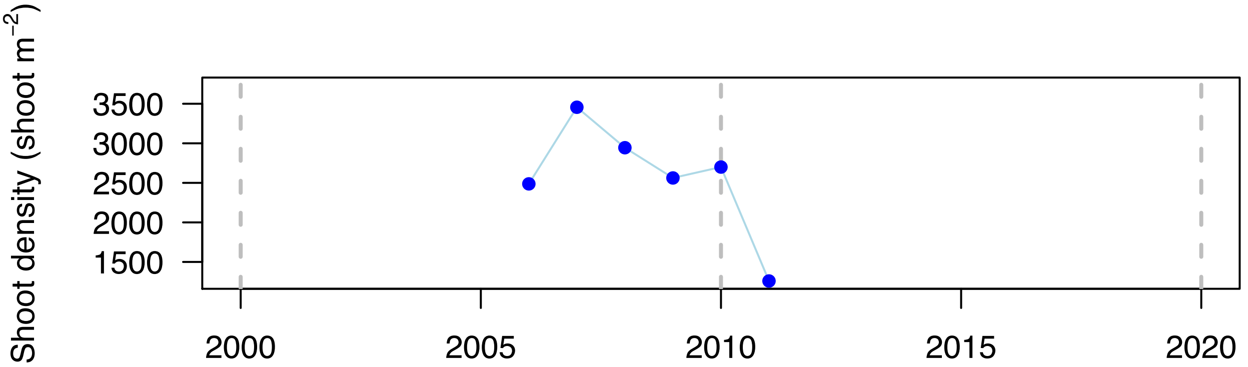

130_biomass
Romero et al. 2010 (b)
SITE: Alfacs (marine) (Spain – Mediterranean) – Cn (–1 m)
OVERALL: Net = –26 g dw m–2;   Rate = –2.8 % yr–1; Perc Final = 87 % > no change
DECADAL: NO (5 yr)

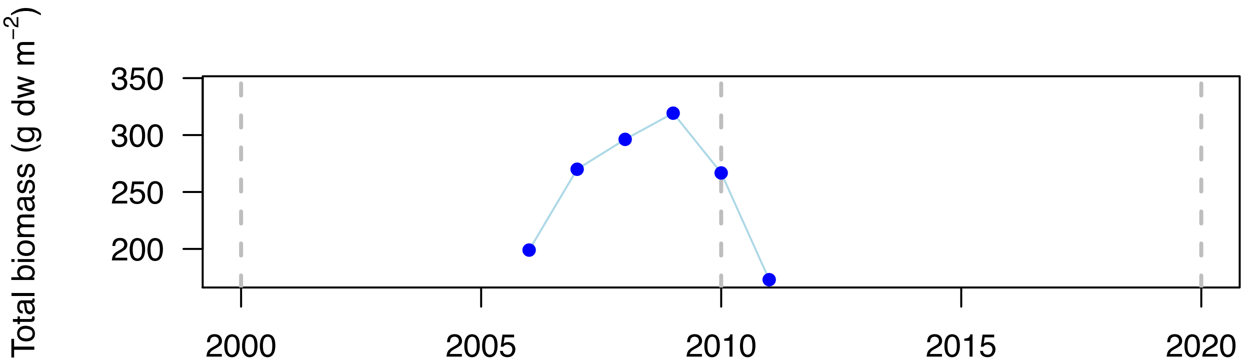

130_density

Romero et al. 2010 (b)

SITE: Alfacs (marine) (Spain – Mediterranean) – Cn (–1 m)

OVERALL: Net = –1763.32 shoot m–2;   Rate = –14.18 % yr–1; Perc Final = 49 % > decrease

DECADAL: NO (5 yr)

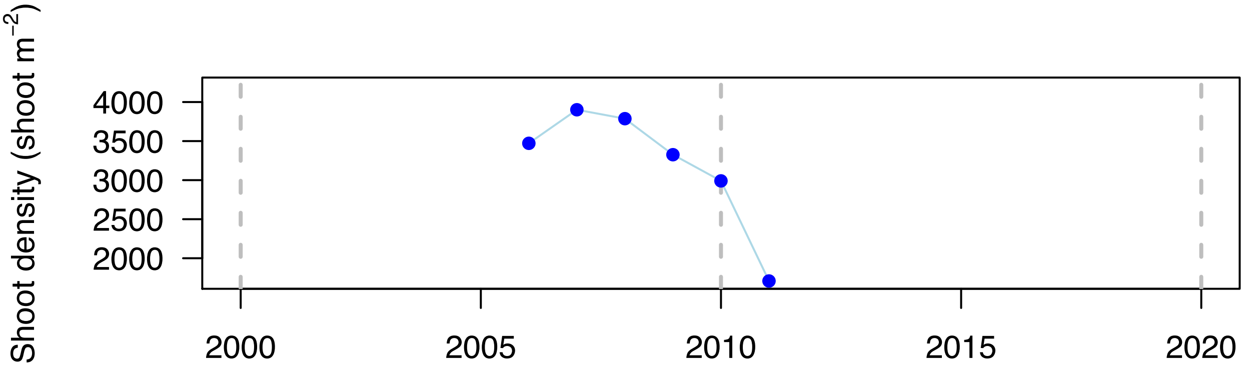

131_biomass

Romero et al. 2010 (b)

SITE: Fangar (eutrophic) (Spain – Mediterranean) – Cn (–1 m)

OVERALL: Net = 112.17 g dw m–2;   Rate = 9.34 % yr–1; Perc Final = 160 % > increase

DECADAL: NO (5 yr)

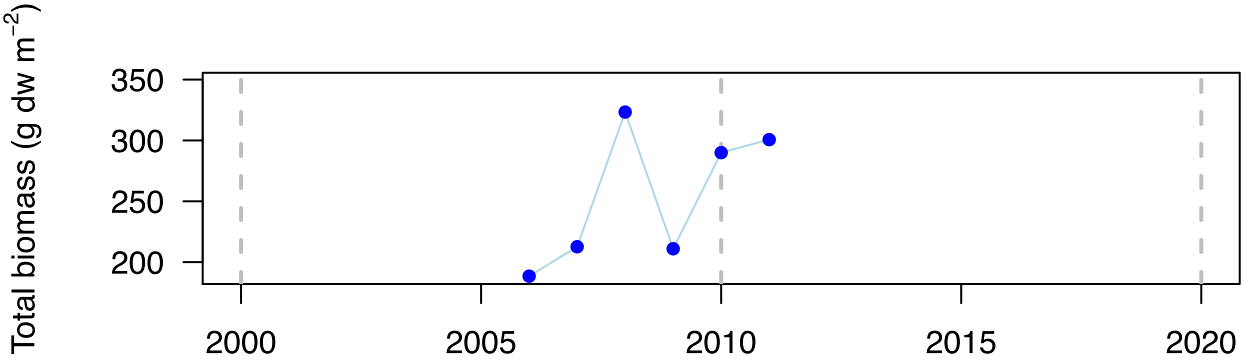

131_density
Romero et al. 2010 (b)
SITE: Fangar (eutrophic) (Spain – Mediterranean) – Cn (–1 m)
OVERALL: Net = 1914.35 shoot m–2;   Rate = 18.59 % yr–1; Perc Final = 253 % > increase
DECADAL: NO (5 yr)

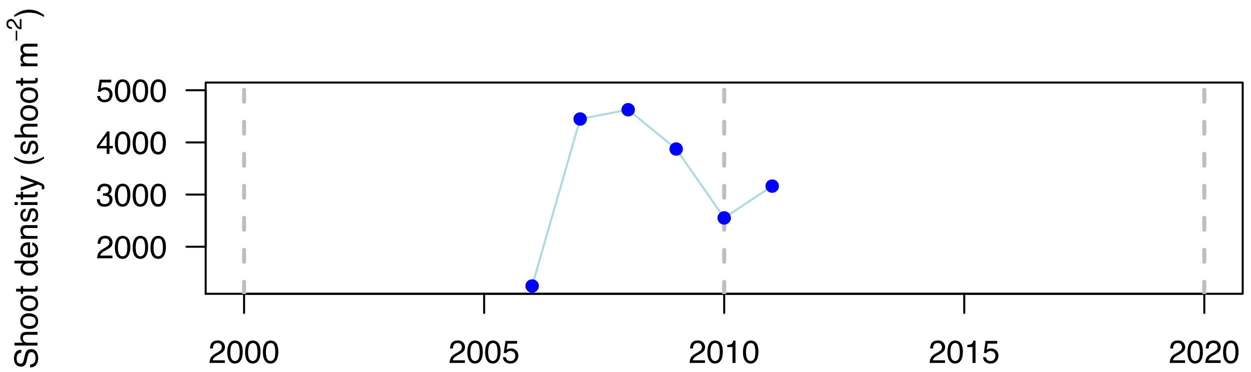

132_biomass
Romero et al. 2010 (b)
SITE: Fangar (marine) (Spain – Mediterranean) – Cn (–1 m)
OVERALL: Net = 159.5 g dw m–2;   Rate = 11.86 % yr–1; Perc Final = 181 % > increase
DECADAL: NO (5 yr)

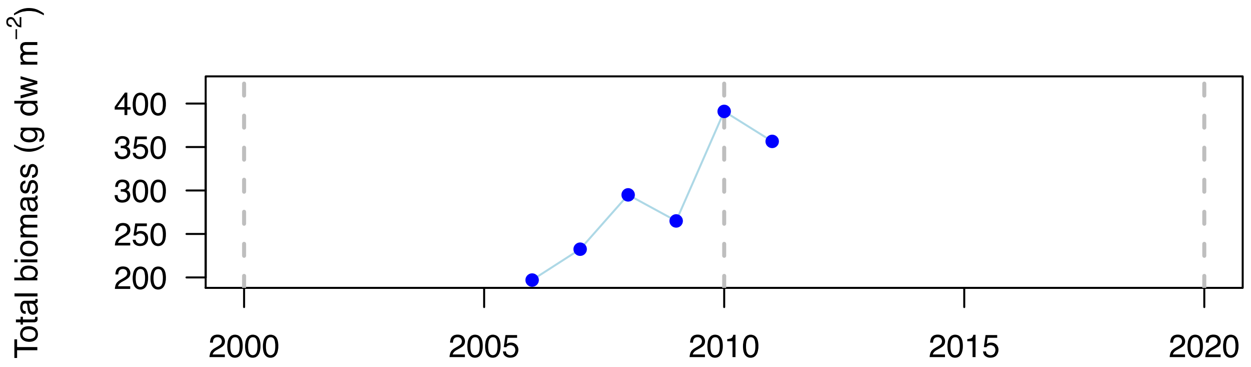

132_density

Romero et al. 2010 (b)

SITE: Fangar (marine) (Spain – Mediterranean) – Cn (–1 m)

OVERALL: Net = –833.24 shoot m–2;   Rate = –4.97 % yr–1; Perc Final = 78 % > no change

DECADAL: NO (5 yr)

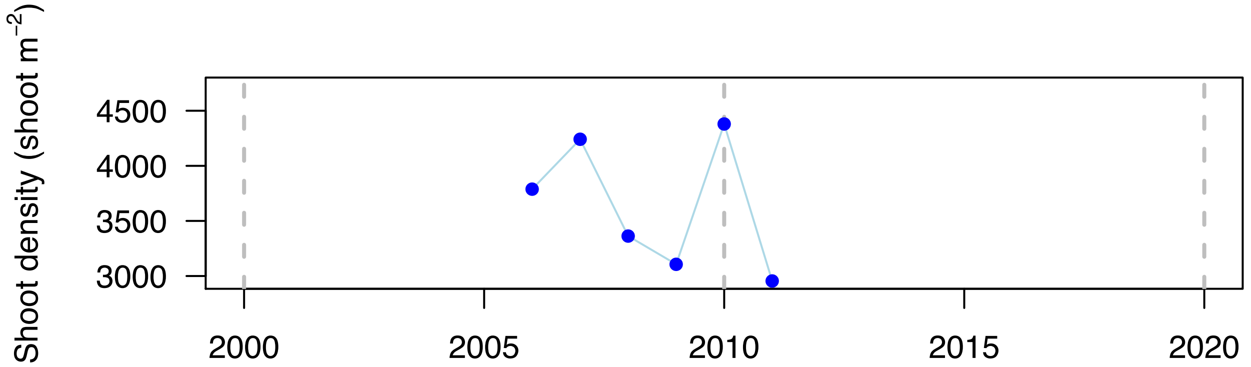

133_cover

Romero et al. 2012

SITE: Islas Medas (station) (Spain – Mediterranean) – Po (–14 m)

OVERALL: Net = –14 %;   Rate = –1.59 % yr–1; Perc Final = 64 % > decrease

DECADAL: YES (28 yr)

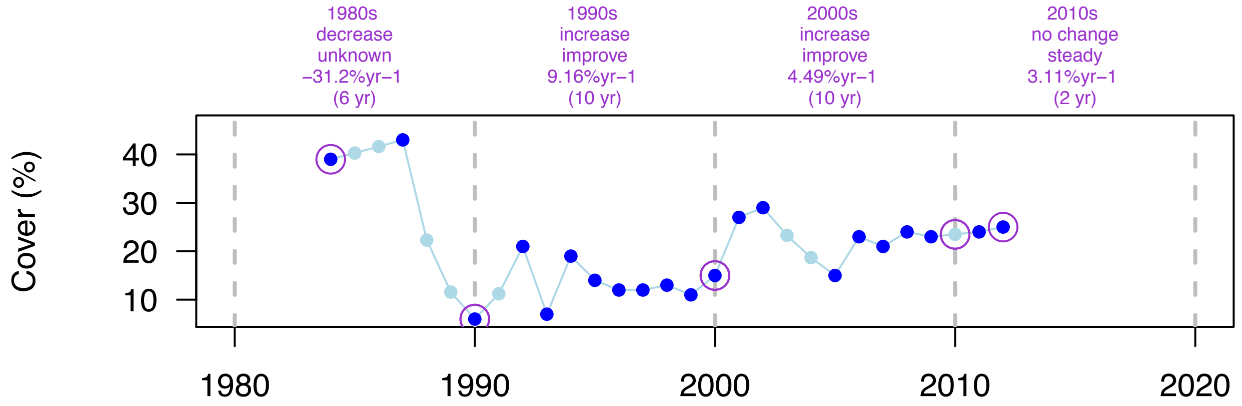

133_density

Romero et al. 2012

SITE: Islas Medas (station) (Spain – Mediterranean) – Po (–14 m)

OVERALL: Net = –94 shoot m–2; Rate = –1.16 % yr–1; Perc Final = 72 % > decrease

DECADAL: YES (28 yr)

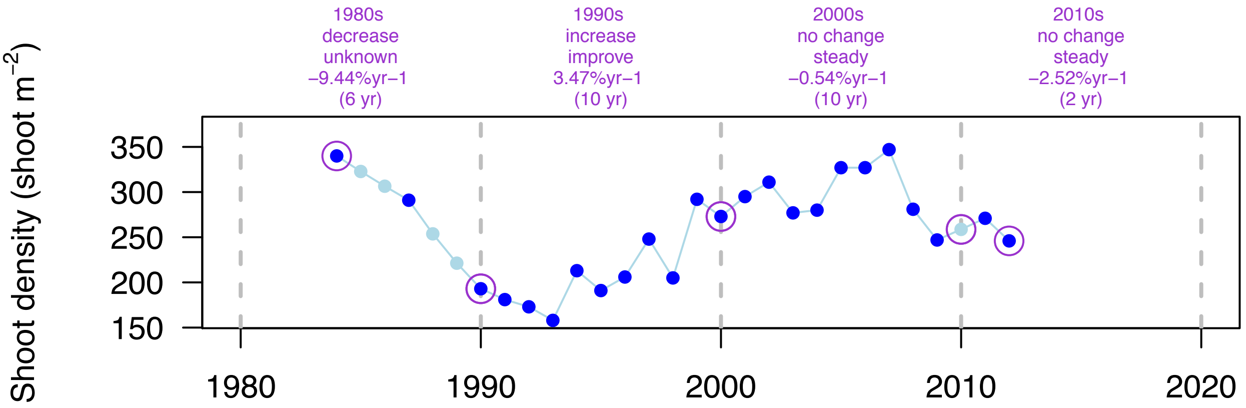

134_cover

Romero et al. 2012

SITE: Islas Medas (station) (Spain – Mediterranean) – Po (–5 m)

OVERALL: Net = –19 %; Rate = –1.03 % yr–1; Perc Final = 75 % > decrease

DECADAL: YES (28 yr)

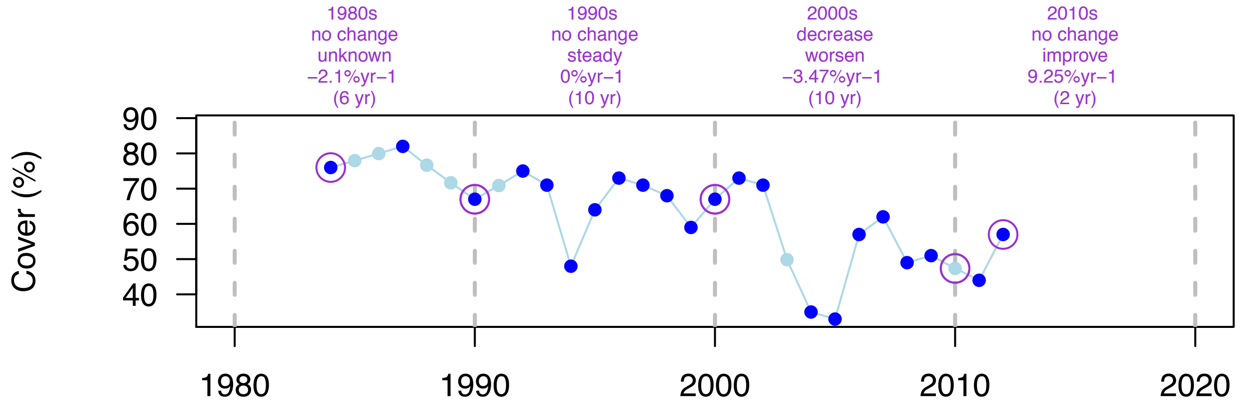

134_density

Romero et al. 2012

SITE: Islas Medas (station) (Spain – Mediterranean) – Po (–5 m)

OVERALL: Net = –44 shoot m–2;   Rate = –0.26 % yr–1; Perc Final = 93 % > no change

DECADAL: YES (28 yr)

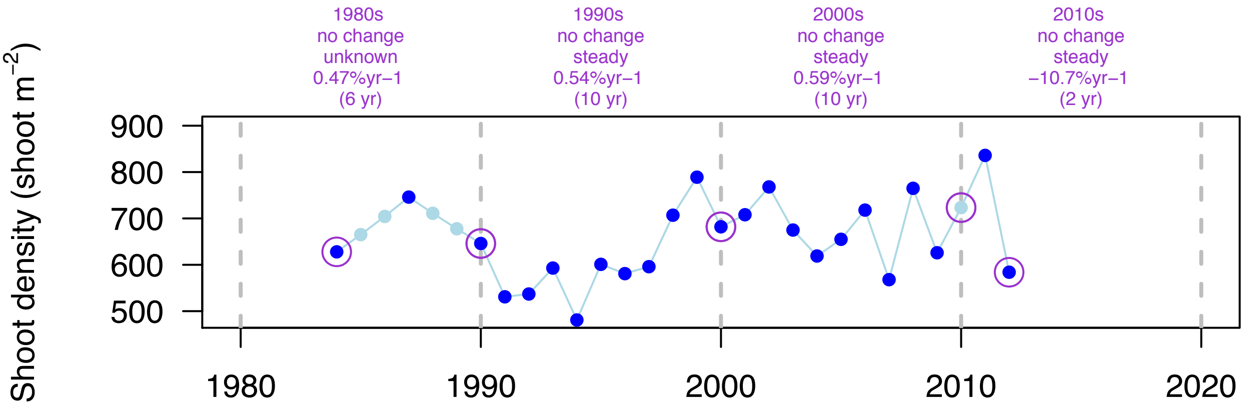

135_cover

Romero et al. 2012

SITE: Islas Medas (station) (Spain – Mediterranean) – Po (–6.5 m)

OVERALL: Net = 13 %;   Rate = 0.84 % yr–1; Perc Final = 127 % > increase

DECADAL: YES (28 yr)

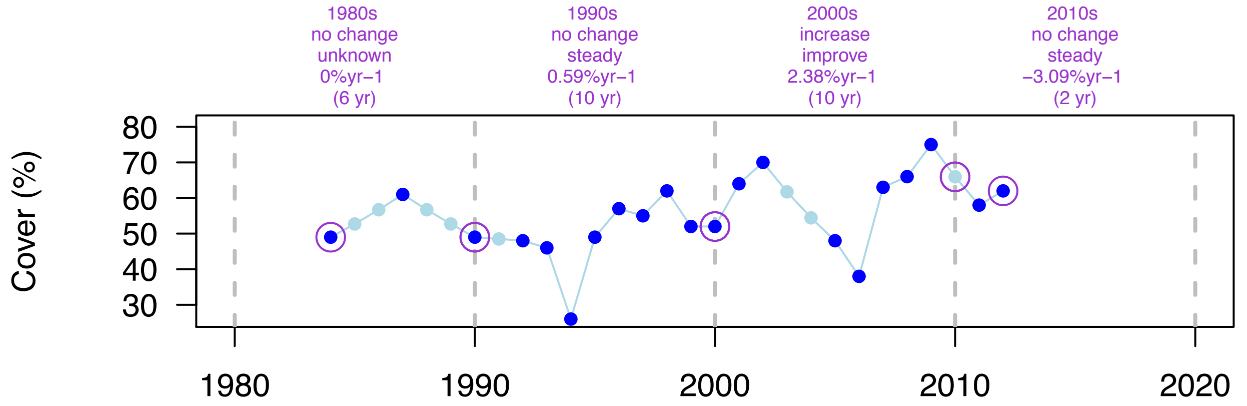

135_density

Romero et al. 2012

SITE: Islas Medas (station) (Spain – Mediterranean) – Po (–6.5 m)

OVERALL: Net = –110 shoot m–2; Rate = –0.81 % yr–1; Perc Final = 80 % > no change

DECADAL: YES (28 yr)

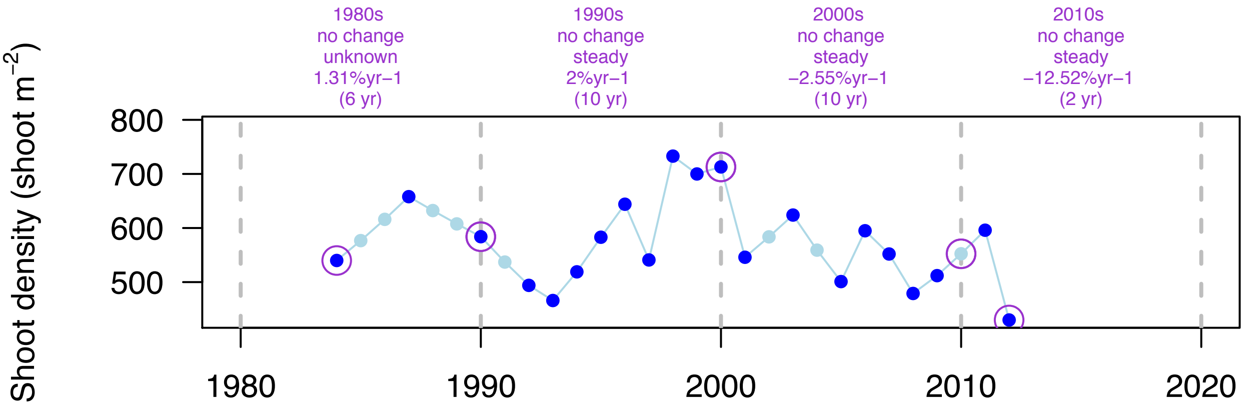

136_cover

Romero et al. 2012

SITE: Islas Medas (station) (Spain – Mediterranean) – Po (–8.5 m)

OVERALL: Net = 6 %; Rate = 0.45 % yr–1; Perc Final = 113 % > no change

DECADAL: YES (28 yr)

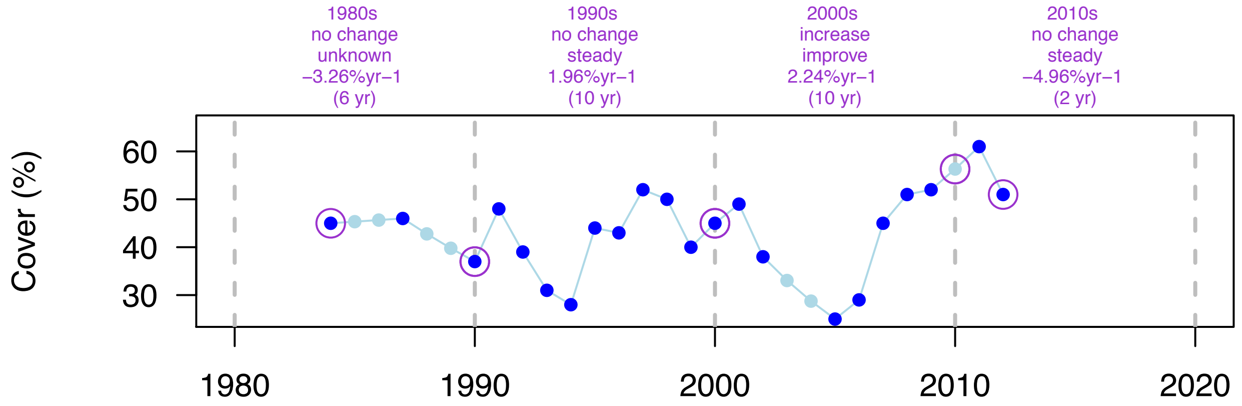

136_density

Romero et al. 2012

SITE: Islas Medas (station) (Spain – Mediterranean) – Po (–8.5 m)

OVERALL: Net = –102 shoot m–2; Rate = –0.91 % yr–1; Perc Final = 78 % > no change

DECADAL: YES (28 yr)

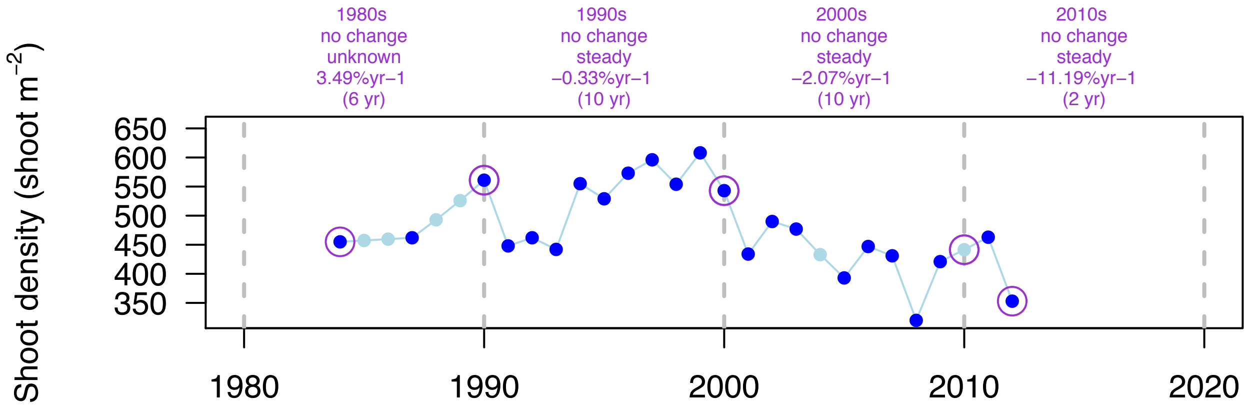

137_cover

Romero et al. 2010 (a)

SITE: Balís (Spain – Mediterranean) – Po (–15 m)

OVERALL: Net = –7.22 %; Rate = –3.18 % yr–1; Perc Final = 80 % > no change

DECADAL: NO (7 yr)

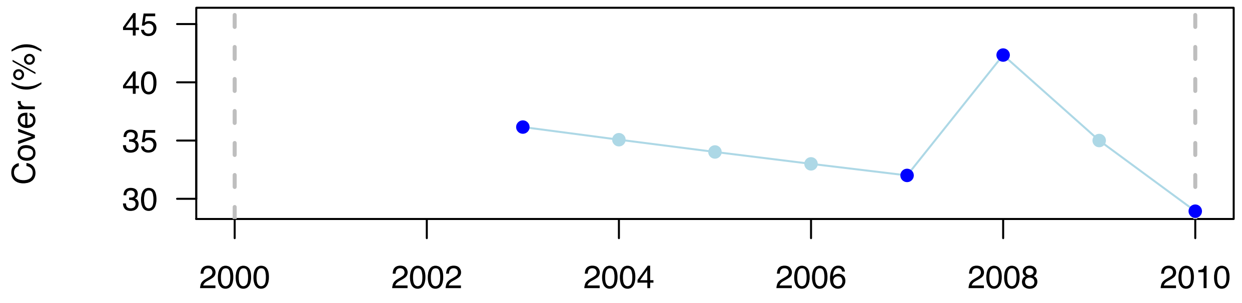

137_density

Romero et al. 2010 (a)

SITE: Balís (Spain – Mediterranean) – Po (–15 m)

OVERALL: Net = 10.94 shoot m–2;   Rate = 0.69 % yr–1; Perc Final = 105 % > no change

DECADAL: NO (7 yr)

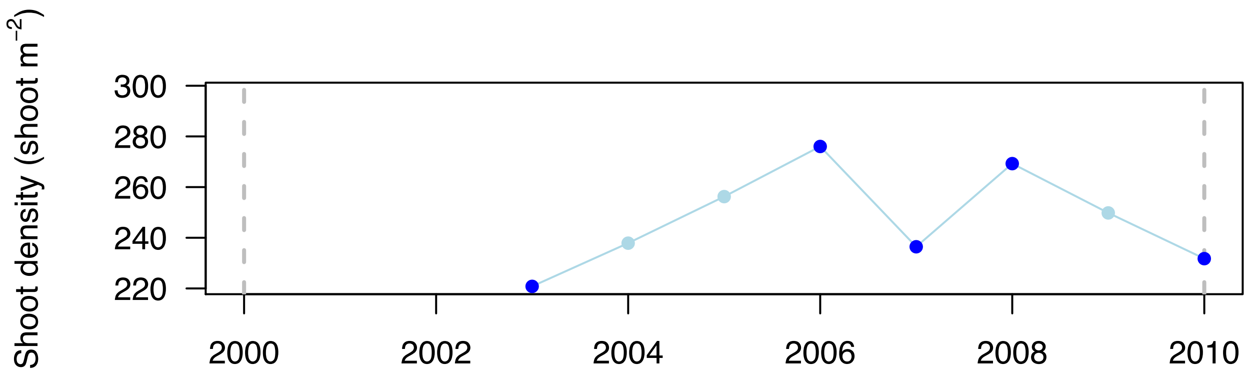

138_cover

Romero et al. 2010 (a)

SITE: Calafat (Spain – Mediterranean) – Po (–15 m)

OVERALL: Net = –2.41 %;   Rate = –1.33 % yr–1; Perc Final = 91 % > no change

DECADAL: NO (7 yr)

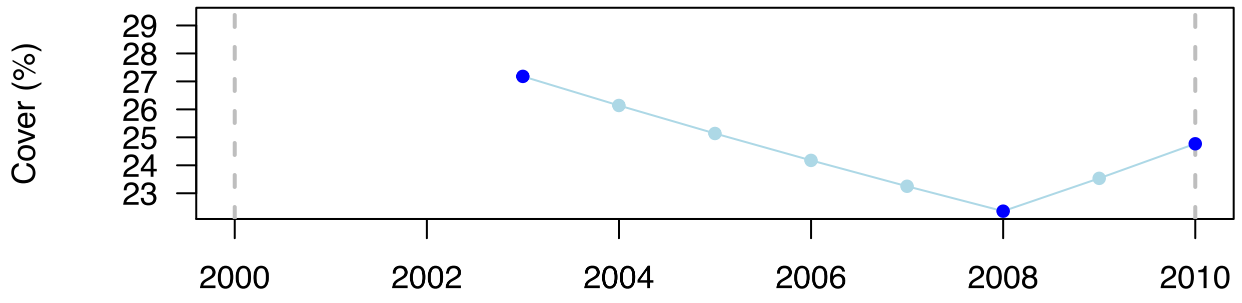

138_density

Romero et al. 2010 (a)

SITE: Calafat (Spain – Mediterranean) – Po (–15 m)

OVERALL: Net = –144.27 shoot m–2;   Rate = –9.1 % yr–1; Perc Final = 53 % > decrease

DECADAL: NO (7 yr)

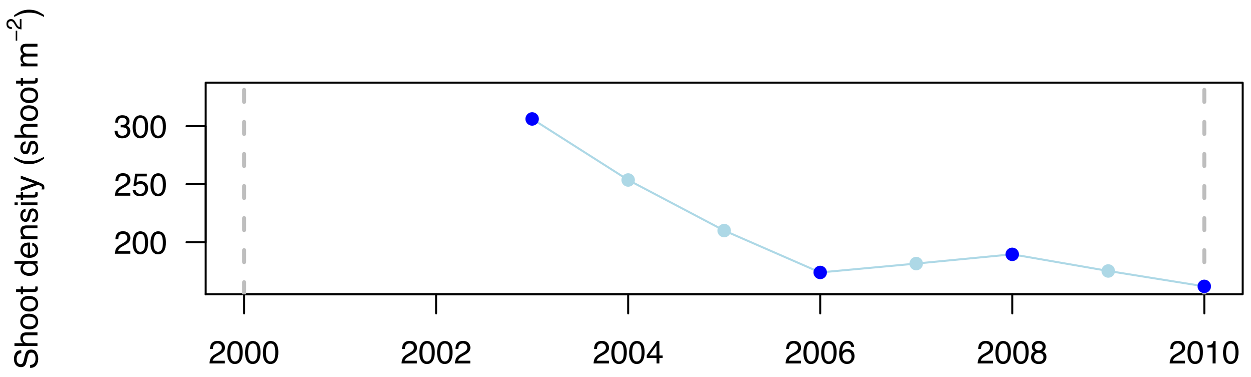

139_cover

Romero et al. 2010 (a), Gera et al. 2014

SITE: Canyelles (Spain – Mediterranean) – Po (–15 m)

OVERALL: Net = –5.79 %;   Rate = –2.46 % yr–1; Perc Final = 86 % > no change

DECADAL: NO (6 yr)

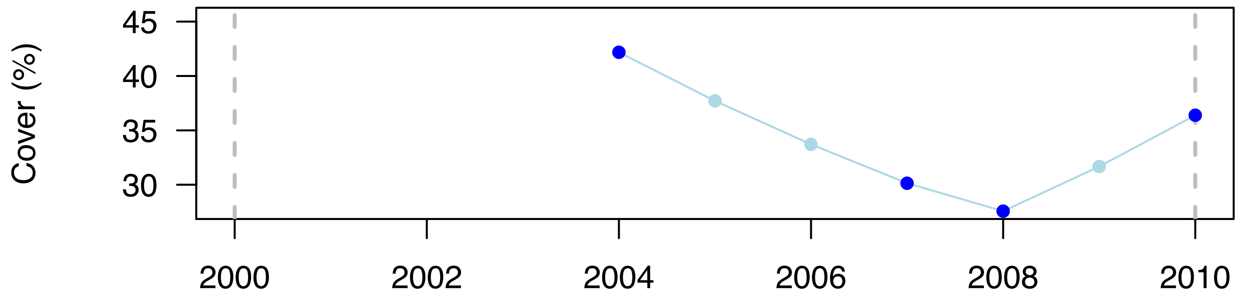

139_density

Romero et al. 2010 (a), Gera et al. 2014

SITE: Canyelles (Spain – Mediterranean) – Po (–15 m)

OVERALL: Net = 127.6 shoot m–2;   Rate = 9.95 % yr–1; Perc Final = 182 % > increase

DECADAL: NO (6 yr)

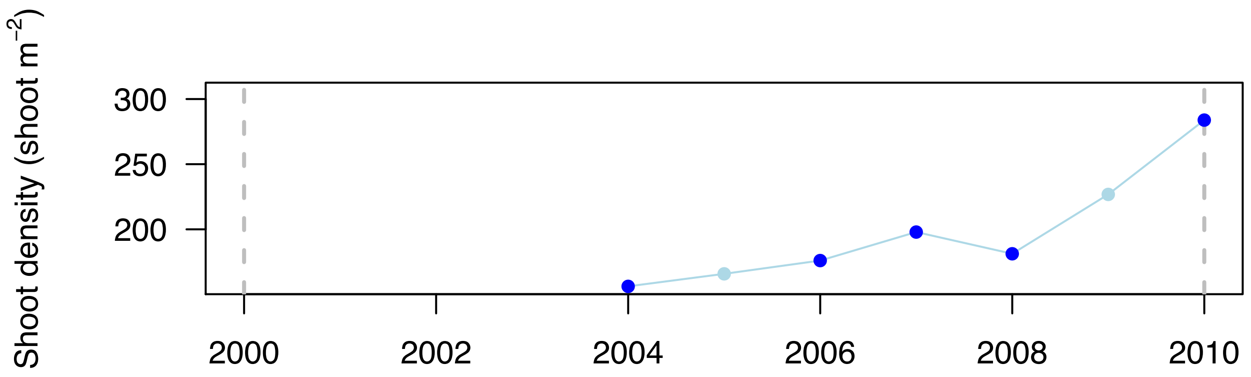

140_cover

Romero et al. 2010 (a)

SITE: Cap Roig (Spain – Mediterranean) – Po (–9 m)

OVERALL: Net = 9.01 %;   Rate = 9.62 % yr–1; Perc Final = 178 % > increase

DECADAL: NO (6 yr)

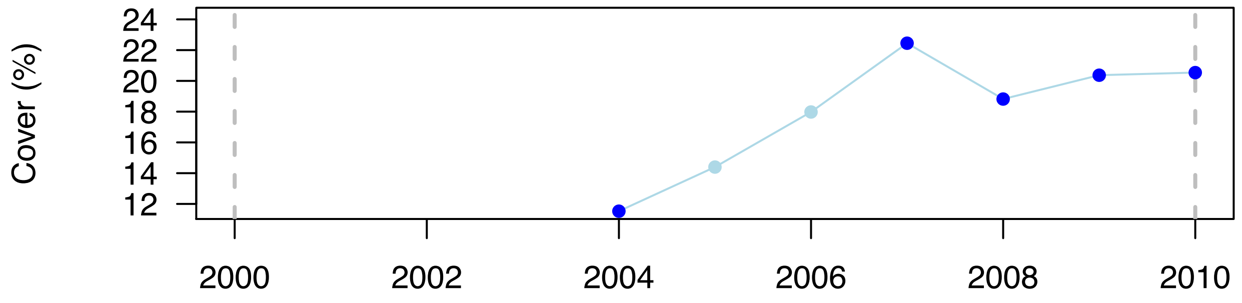

140_density

Romero et al. 2010 (a)

SITE: Cap Roig (Spain – Mediterranean) – Po (–9 m)

OVERALL: Net = 1.57 shoot m–2;   Rate = 0.11 % yr–1; Perc Final = 101 % > no change

DECADAL: NO (6 yr)

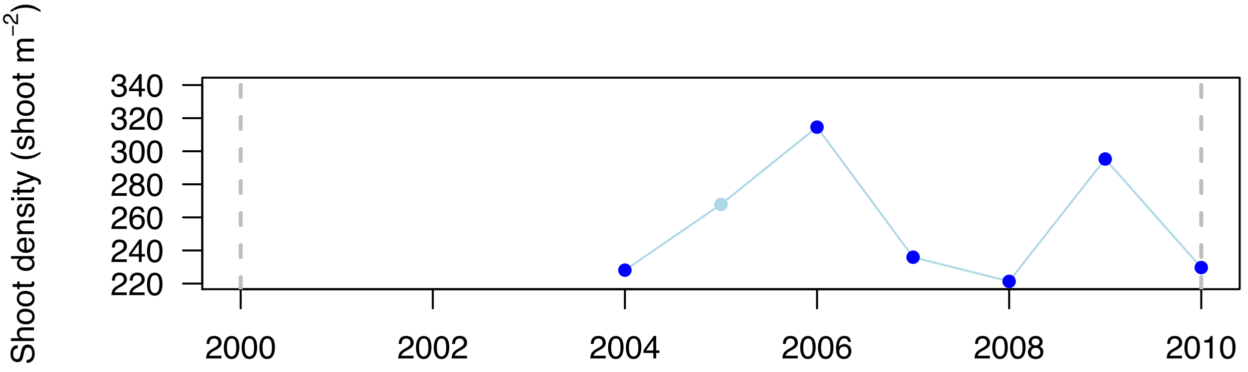

141_cover

Romero et al. 2010 (a)

SITE: Coma–ruga (Spain – Mediterranean) – Po (–15 m)

OVERALL: Net = 10.04 %;   Rate = 9.92 % yr–1; Perc Final = 221 % > increase

DECADAL: YES (8 yr)

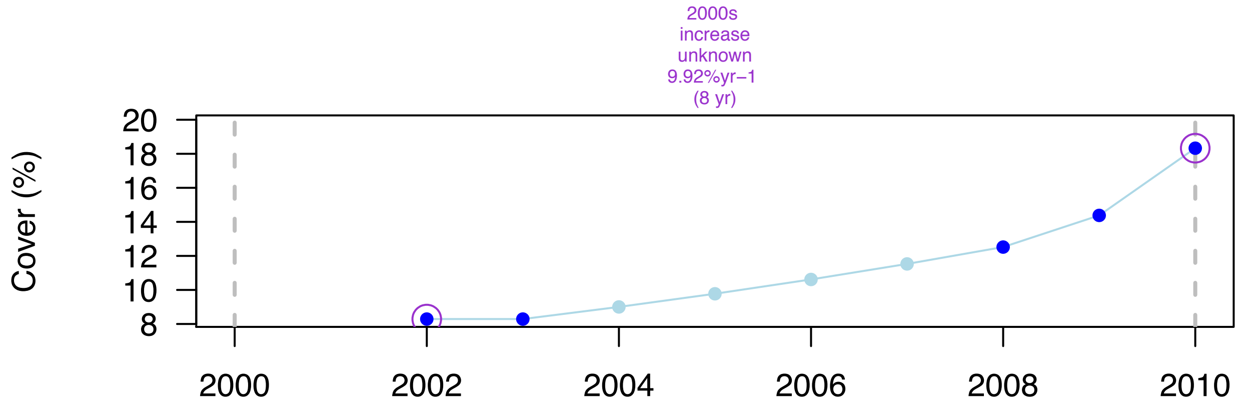

141_density

Romero et al. 2010 (a)

SITE: Coma–ruga (Spain – Mediterranean) – Po (–15 m)

OVERALL: Net = 110.07 shoot m–2;   Rate = 10.07 % yr–1; Perc Final = 224 % > increase

DECADAL: YES (8 yr)

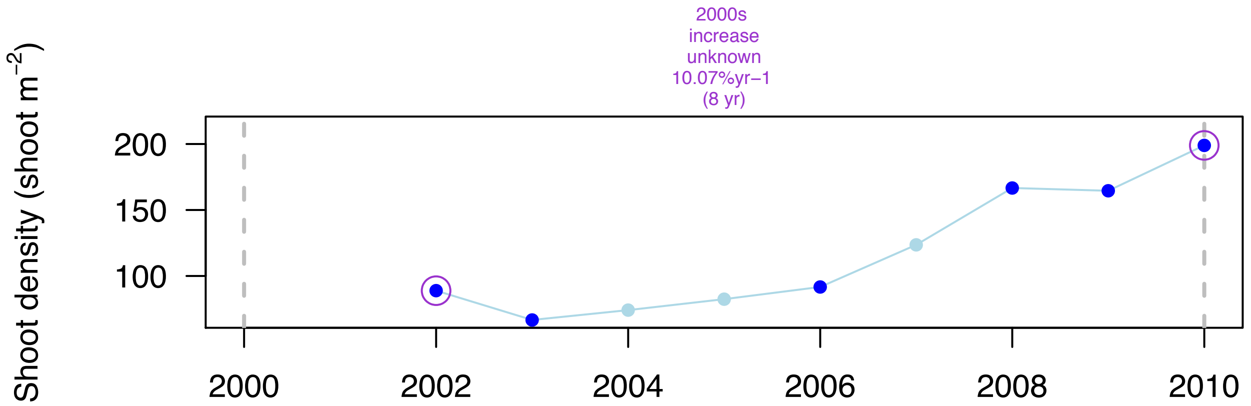

142_cover

Romero et al. 2010 (a)

SITE: Culip (Spain – Mediterranean) – Po (–15 m)

OVERALL: Net = –0.56 %;   Rate = –0.36 % yr–1; Perc Final = 98 % > no change

DECADAL: NO (7 yr)

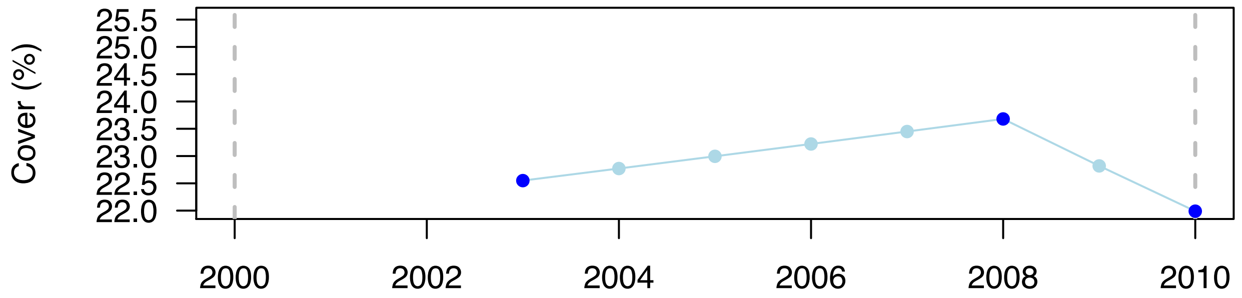

142_density
Romero et al. 2010 (a)
SITE: Culip (Spain – Mediterranean) – Po (–15 m)
OVERALL: Net = –3.13 shoot m–2;   Rate = –0.24 % yr–1; Perc Final = 98 % > no change
DECADAL: NO (7 yr)

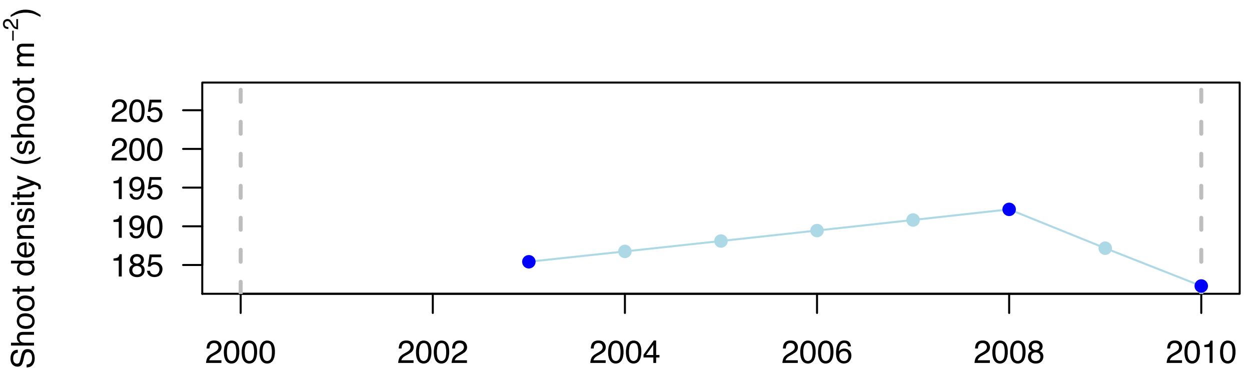

143_cover
Romero et al. 2010 (a), Gera et al. 2014
SITE: Fenals (Spain – Mediterranean) – Po (–16.2 m)
OVERALL: Net = 4.21 %;   Rate = 1.36 % yr–1; Perc Final = 112 % > no change
DECADAL: YES (8 yr)

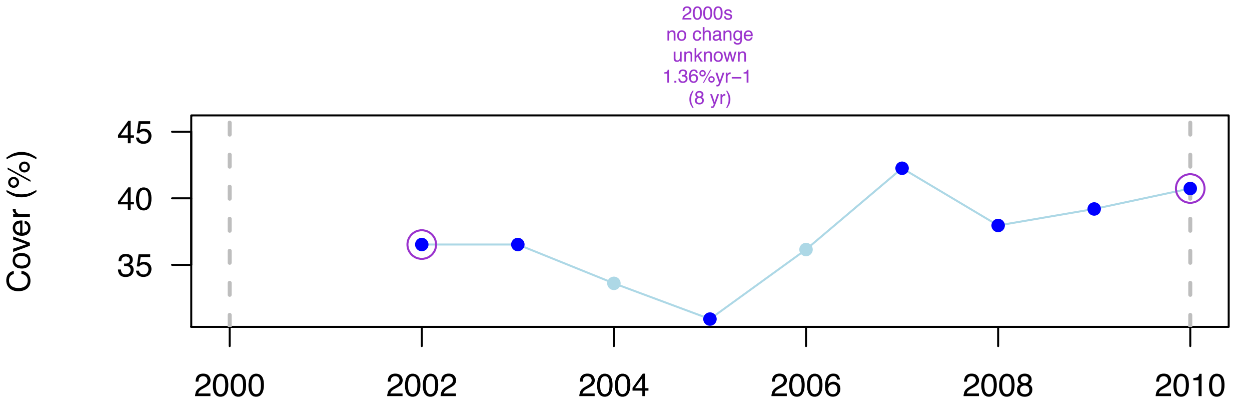

143_density

Romero et al. 2010 (a), Gera et al. 2014

SITE: Fenals (Spain – Mediterranean) – Po (–16.2 m)

OVERALL: Net = 39.58 shoot m–2;   Rate = 2.2 % yr–1; Perc Final = 119 % > no change

DECADAL: YES (8 yr)

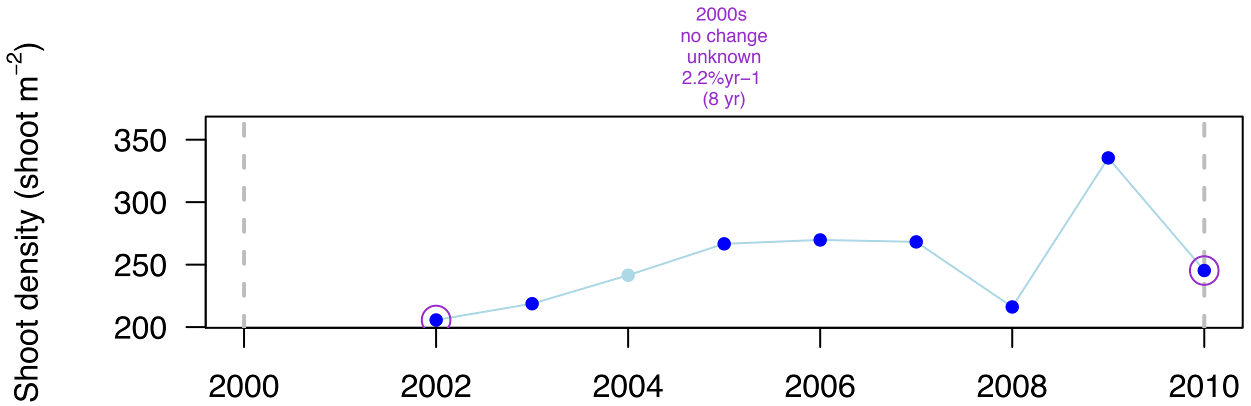

144_cover

Romero et al. 2010 (a)

SITE: Jugadora (Spain – Mediterranean) – Po (–16 m)

OVERALL: Net = –1.52 %;   Rate = –0.91 % yr–1; Perc Final = 93 % > no change

DECADAL: YES (8 yr)

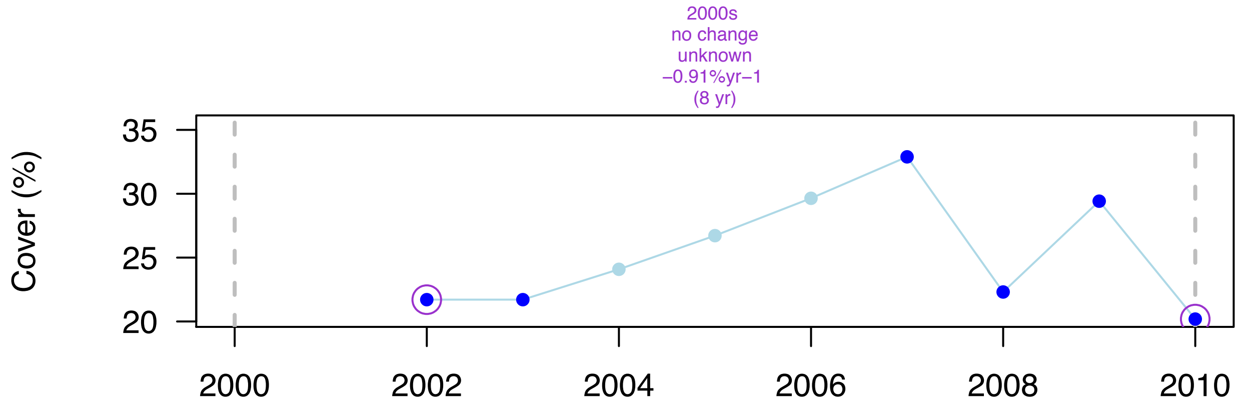

144_density

Romero et al. 2010 (a)

SITE: Jugadora (Spain – Mediterranean) – Po (–16 m)

OVERALL: Net = 23.96 shoot m–2; Rate = 2.1 % yr–1; Perc Final = 118 % > no change

DECADAL: YES (8 yr)

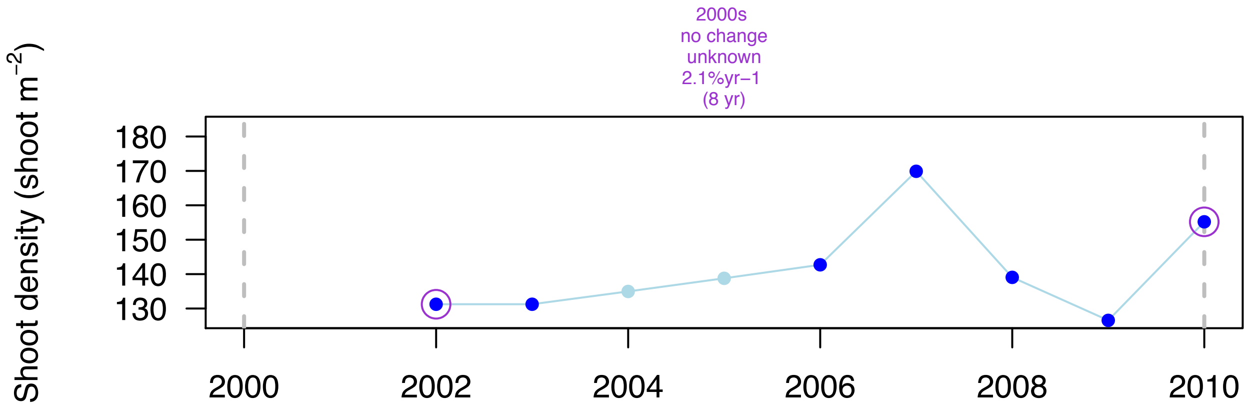

145_cover

Romero et al. 2010 (a)

SITE: L'Ametlla de Mar (Spain – Mediterranean) – Po (–14.8 m)

OVERALL: Net = –1.25 %; Rate = –0.77 % yr–1; Perc Final = 95 % > no change

DECADAL: NO (7 yr)

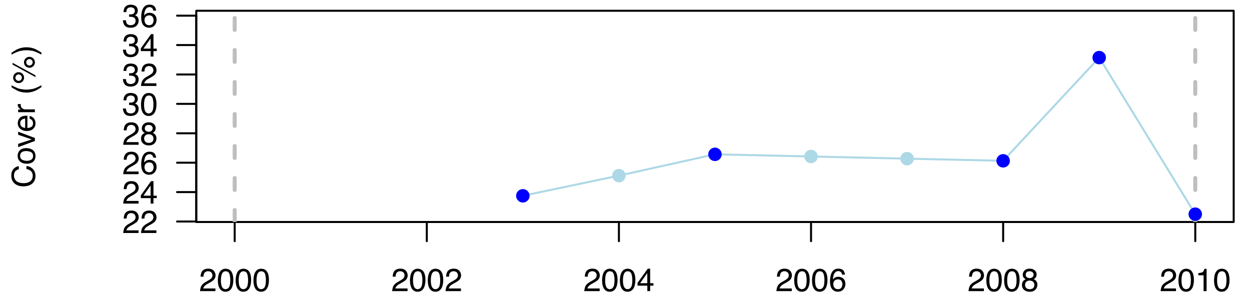

145_density Romero et al. 2010 (a)

SITE: L'Ametlla de Mar (Spain – Mediterranean) – Po (–14.8 m)

OVERALL: Net = –109.89 shoot m–2;   Rate = –6.14 % yr–1; Perc Final = 65 % > decrease

DECADAL: NO (7 yr)

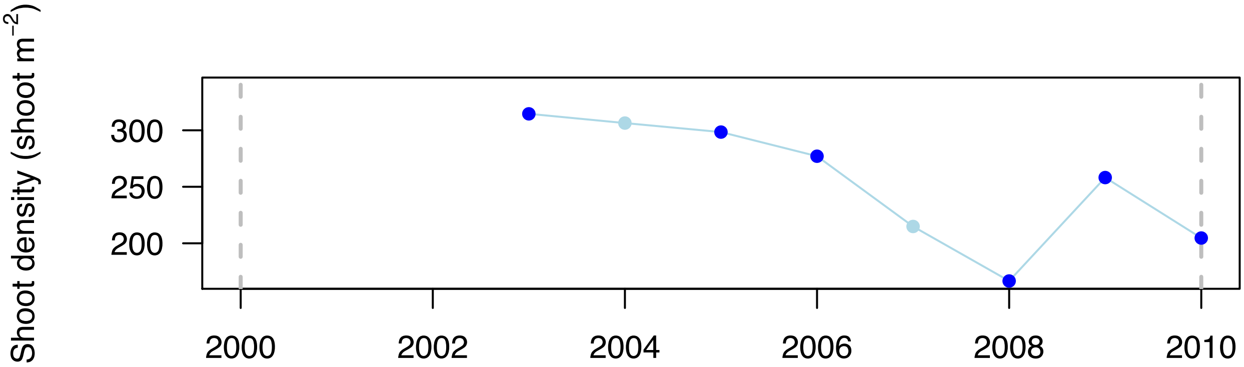

146_cover Romero et al. 2010 (a)

SITE: L'Hospitalet (Spain – Mediterranean) – Po (–15 m)

OVERALL: Net = 2.67 %;   Rate = 2.88 % yr–1; Perc Final = 122 % > no change

DECADAL: NO (7 yr)

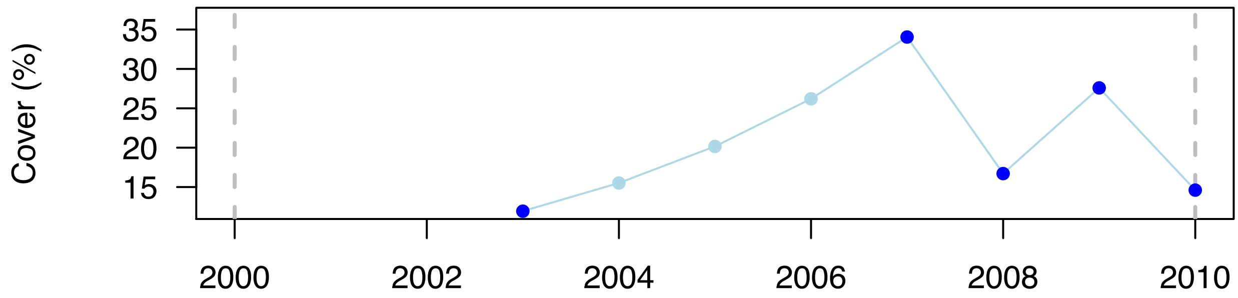

146_density

Romero et al. 2010 (a)

SITE: L'Hospitalet (Spain – Mediterranean) – Po (–15 m)

OVERALL: Net = –61.98 shoot m–2; Rate = –4.94 % yr–1; Perc Final = 71 % > decrease

DECADAL: NO (7 yr)

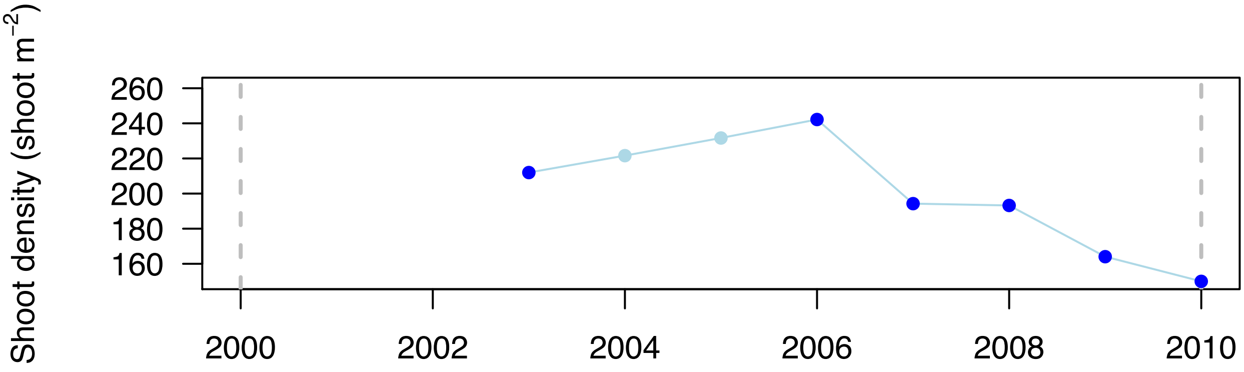

147_cover

Romero et al. 2010 (a)

SITE: Llafranc (Spain – Mediterranean) – Po (–15.5 m)

OVERALL: Net = 6.6 %; Rate = 3.46 % yr–1; Perc Final = 127 % > increase

DECADAL: NO (7 yr)

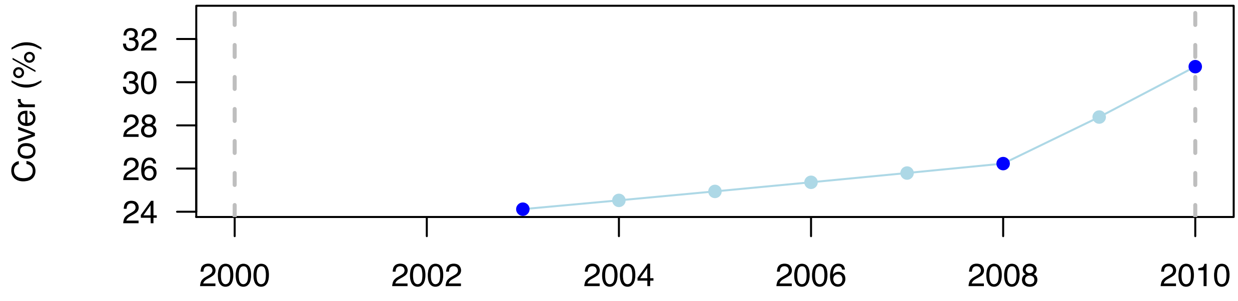

147_density

Romero et al. 2010 (a)

SITE: Llafranc (Spain – Mediterranean) – Po (–15.5 m)

OVERALL: Net = –76.04 shoot m–2;   Rate = –4.7 % yr–1; Perc Final = 72 % > decrease

DECADAL: NO (7 yr)

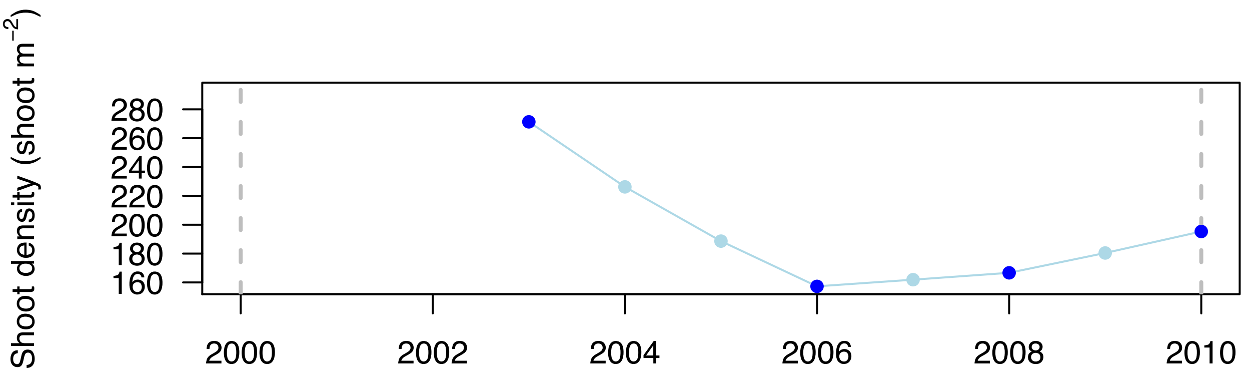

148_abiomass

Milchakova 2003

SITE: Utlyuk Salt Lake (Ukraine – Mediterranean) – Zn (? m)

OVERALL: Net = 407 g dw m–2;   Rate = 2.85 % yr–1; Perc Final = 257 % > increase

DECADAL: YES (33 yr)

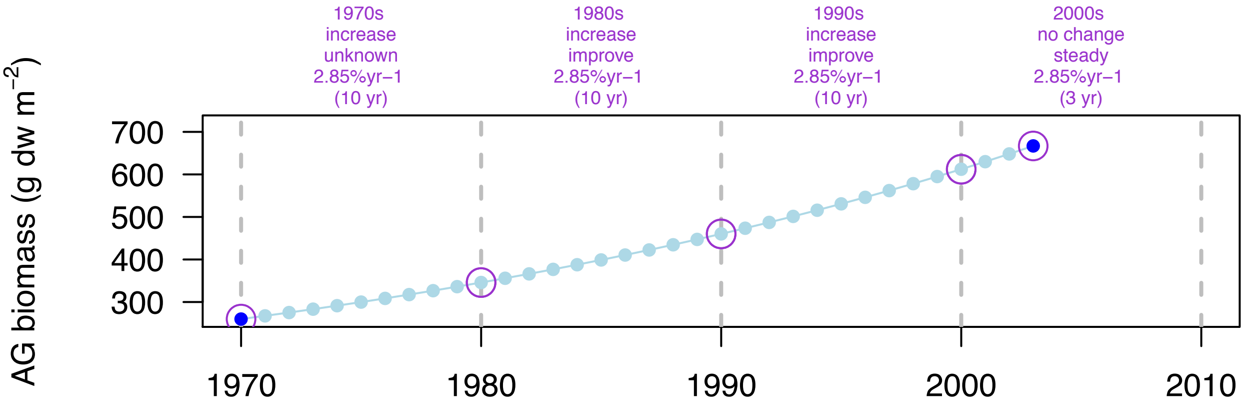

149_cover

Romero et al. 2010 (a)

SITE: Montgó (Spain – Mediterranean) – Po (–12.5 m)

OVERALL: Net = –8.86 %;   Rate = –4.49 % yr–1; Perc Final = 70 % > decrease

DECADAL: YES (8 yr)

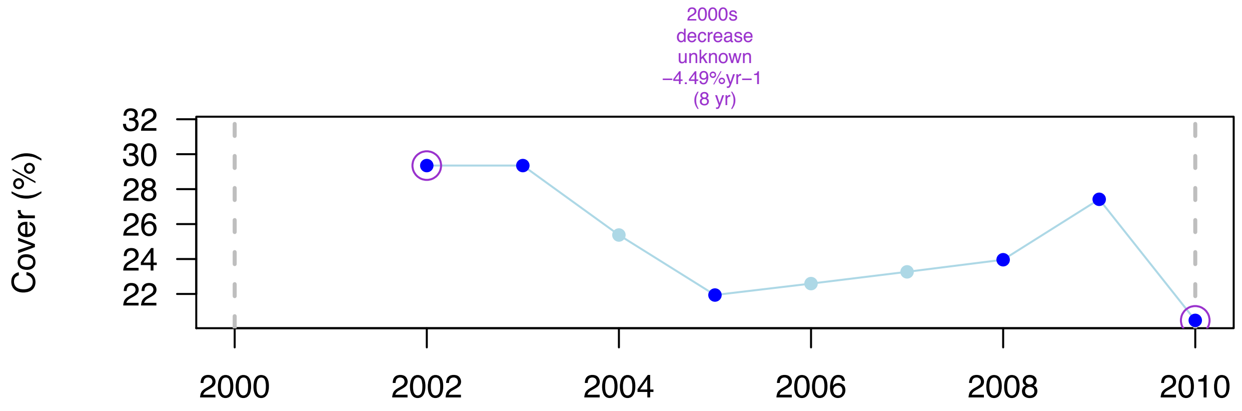

149_density

Romero et al. 2010 (a)

SITE: Montgó (Spain – Mediterranean) – Po (–12.5 m)

OVERALL: Net = –33.78 shoot m–2;   Rate = –1.7 % yr–1; Perc Final = 87 % > no change

DECADAL: YES (8 yr)

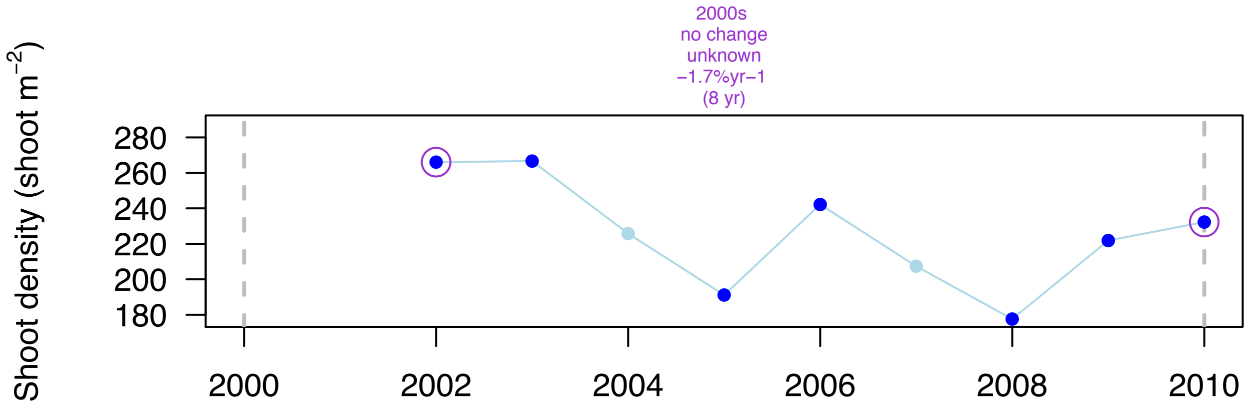

150_cover

Romero et al. 2010 (a)

SITE: Montjoi (Spain – Mediterranean) – Po (–14 m)

OVERALL: Net = –15.52 %;   Rate = –6.62 % yr–1; Perc Final = 59 % > decrease

DECADAL: YES (8 yr)

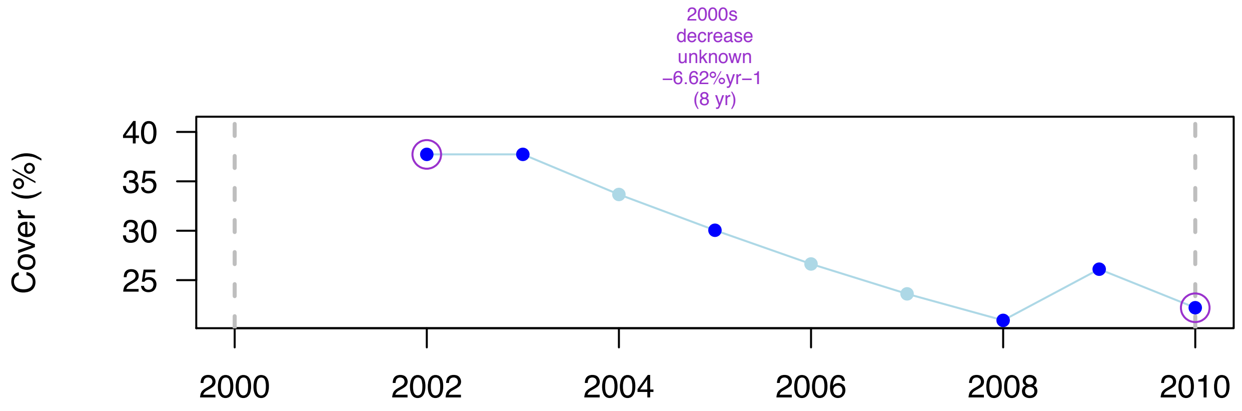

150_density

Romero et al. 2010 (a)

SITE: Montjoi (Spain – Mediterranean) – Po (–14 m)

OVERALL: Net = –90.93 shoot m–2;   Rate = –6.18 % yr–1; Perc Final = 61 % > decrease

DECADAL: YES (8 yr)

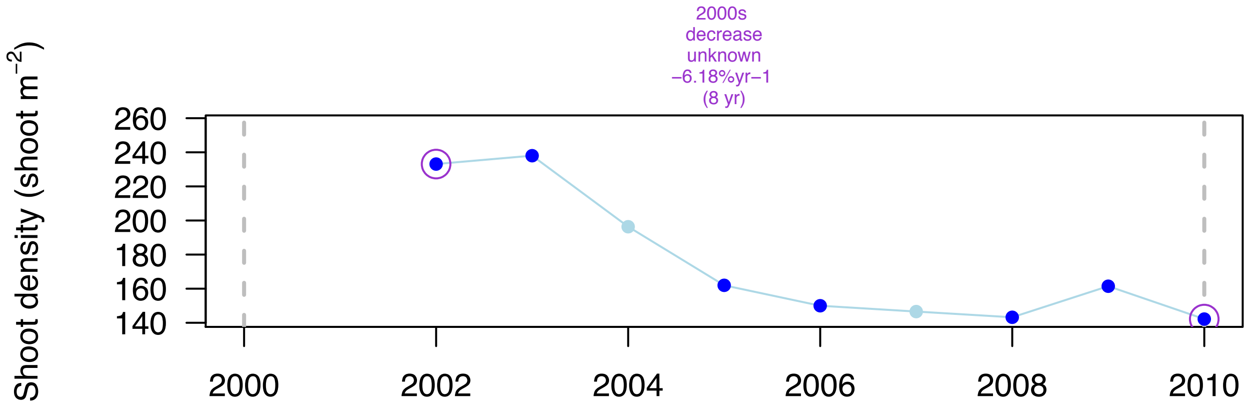

151_cover

Romero et al. 2010 (a)

SITE: Montroig del Camp (Spain – Mediterranean) – Po (–15 m)

OVERALL: Net = –0.51 %;   Rate = –0.32 % yr–1; Perc Final = 97 % > no change

DECADAL: YES (8 yr)

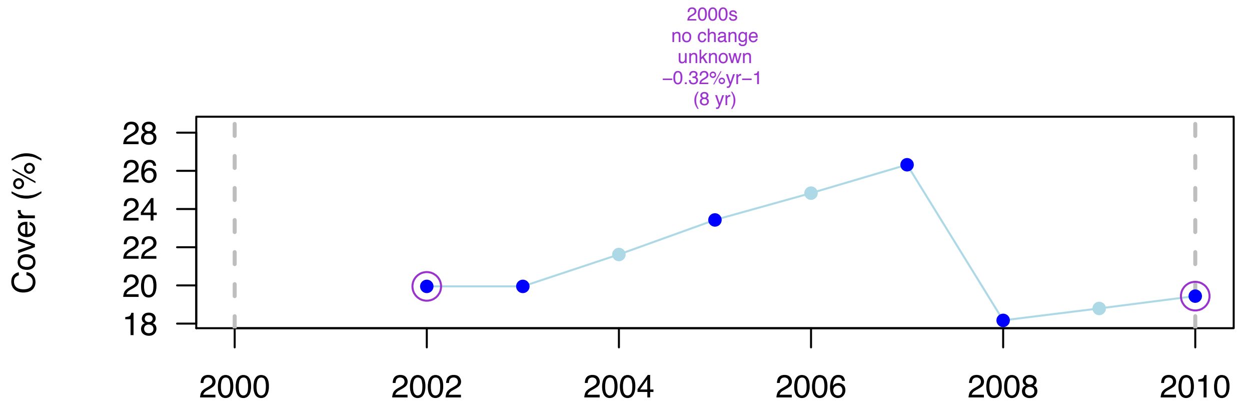

151_density

Romero et al. 2010 (a)

SITE: Montroig del Camp (Spain – Mediterranean) – Po (–15 m)

OVERALL: Net = –15.62 shoot m–2;   Rate = –1.2 % yr–1; Perc Final = 91 % > no change

DECADAL: YES (8 yr)

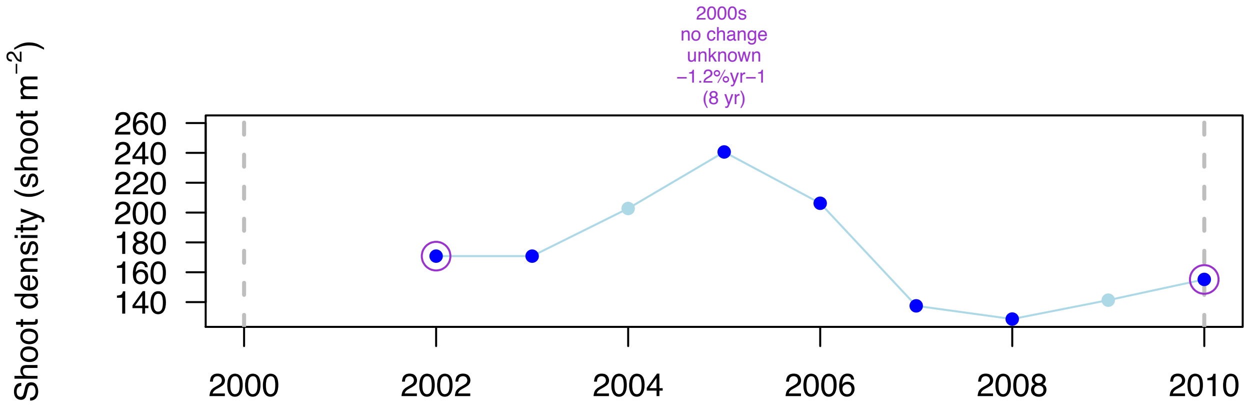

152_cover

Romero et al. 2010 (a), Gera et al. 2014

SITE: Palamós (Spain – Mediterranean) – Po (–14.2 m)

OVERALL: Net = –10.14 %;   Rate = –5.49 % yr–1; Perc Final = 68 % > decrease

DECADAL: NO (7 yr)

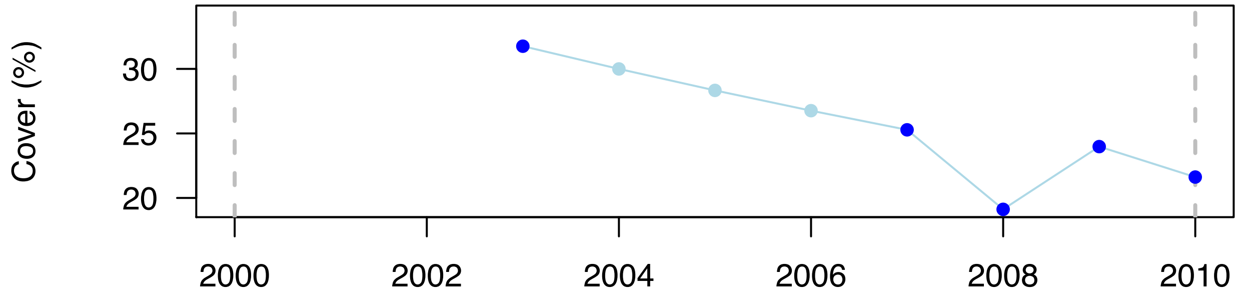

152_density

Romero et al. 2010 (a), Gera et al. 2014

SITE: Palamós (Spain – Mediterranean) – Po (–14.2 m)

OVERALL: Net = –45.83 shoot m–2;   Rate = –3.81 % yr–1; Perc Final = 77 % > no change

DECADAL: NO (7 yr)

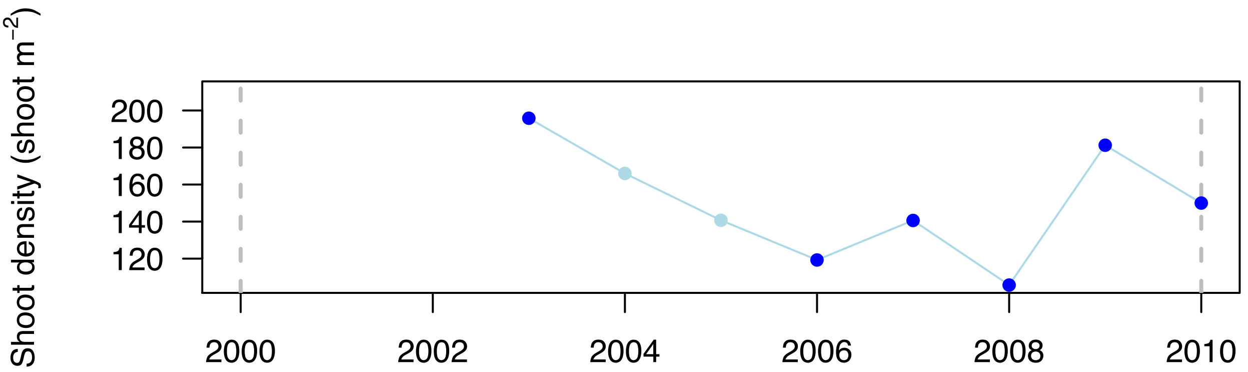

153_cover

Romero et al. 2010 (a)

SITE: Port de la Selva (Spain – Mediterranean) – Po (–14 m)

OVERALL: Net = –16.62 %;   Rate = –15.61 % yr–1; Perc Final = 63 % > decrease

DECADAL: NO (3 yr)

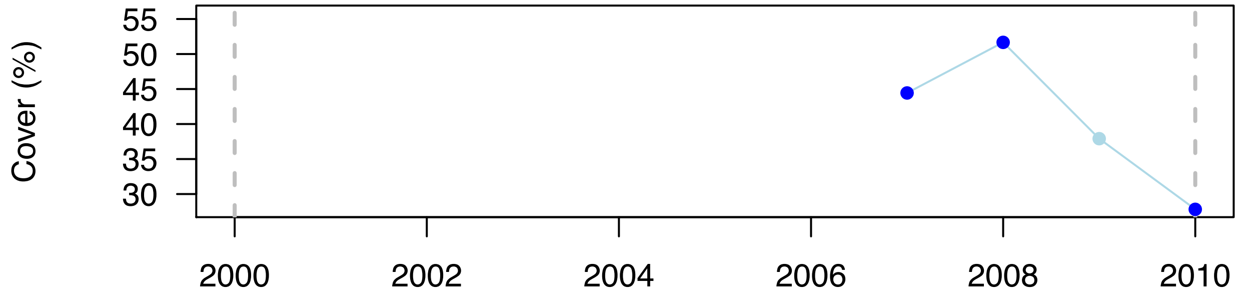

153_density

Romero et al. 2010 (a)

SITE: Port de la Selva (Spain – Mediterranean) – Po (–14 m)

OVERALL: Net = 16.67 shoot m–2;   Rate = 1.73 % yr–1; Perc Final = 107 % > no change

DECADAL: NO (4 yr)

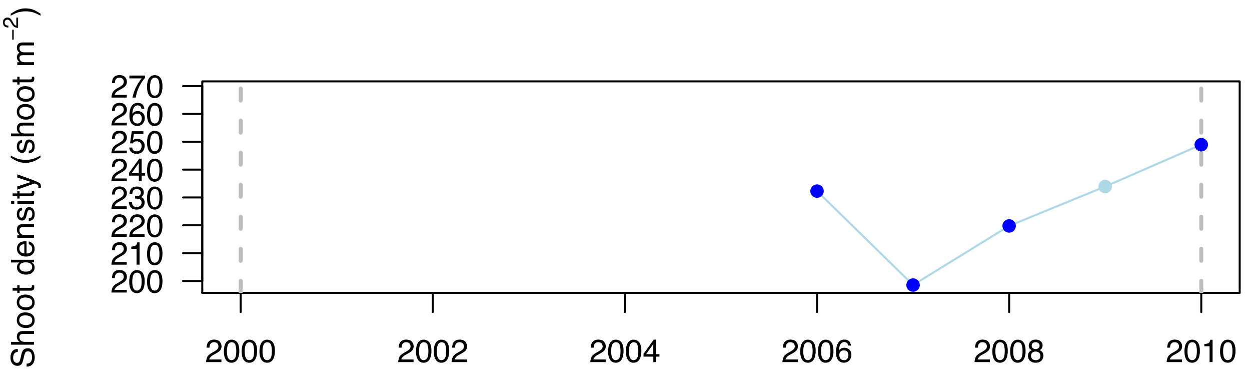

154_cover

Romero et al. 2010 (a)

SITE: Roses (near port) (Spain – Mediterranean) – Po (–13 m)

OVERALL: Net = 10.72 %;   Rate = 11.55 % yr–1; Perc Final = 225 % > increase

DECADAL: NO (7 yr)

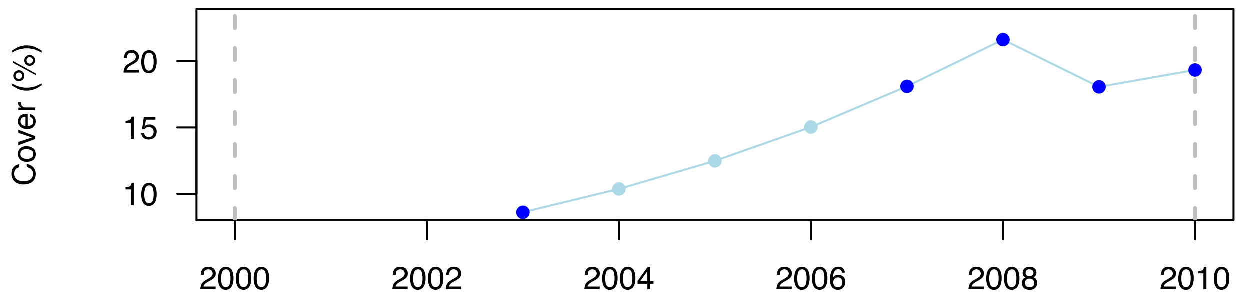

154_density

Romero et al. 2010 (a)

SITE: Roses (near port) (Spain – Mediterranean) – Po (–13 m)

OVERALL: Net = –3.65 shoot m–2;   Rate = –0.37 % yr–1; Perc Final = 97 % > no change

DECADAL: NO (7 yr)

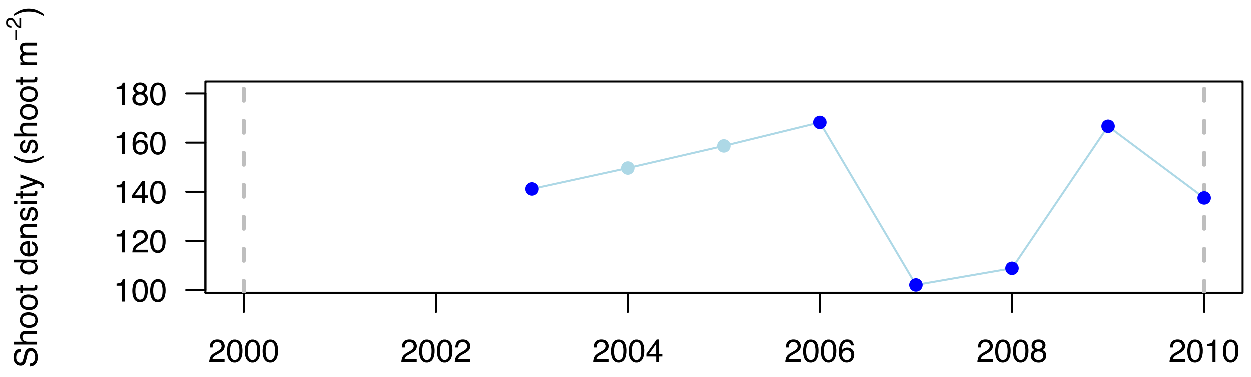

155_cover

Romero et al. 2010 (a)

SITE: Roses (Playa Almadraba) (Spain – Mediterranean) – Po (–13 m)

OVERALL: Net = –12.8 %;   Rate = –13.69 % yr–1; Perc Final = 66 % > decrease

DECADAL: NO (3 yr)

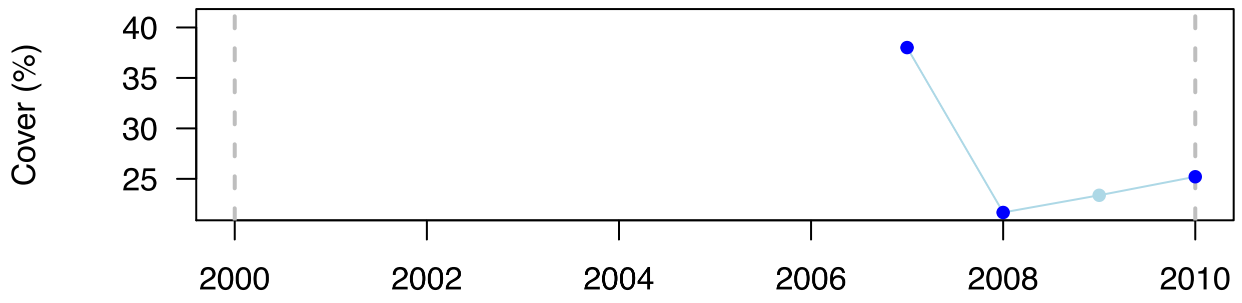

155_density

Romero et al. 2010 (a)

SITE: Roses (Playa Almadraba) (Spain – Mediterranean) – Po (–13 m)

OVERALL: Net = 9.38 shoot m–2;   Rate = 1.79 % yr–1; Perc Final = 106 % > no change

DECADAL: NO (3 yr)

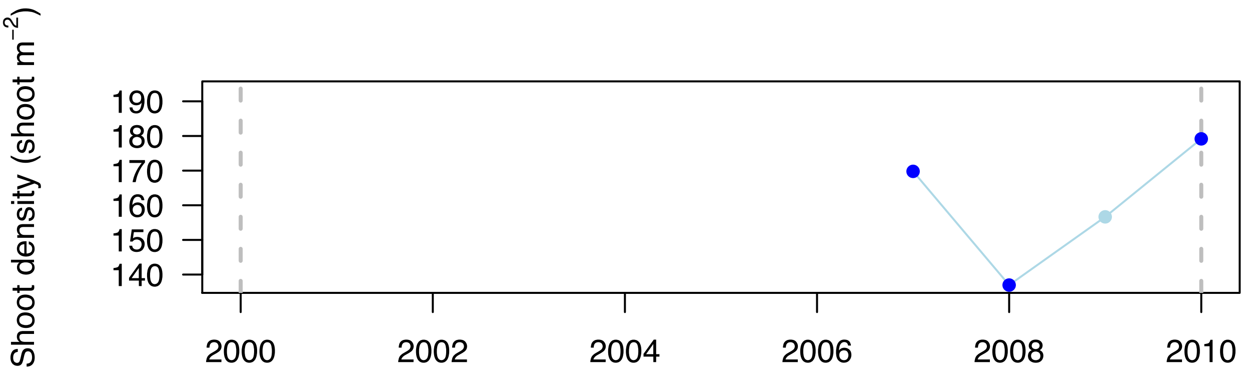

156_cover

Romero et al. 2010 (a)

SITE: Rovellada (Spain – Mediterranean) – Po (–14 m)

OVERALL: Net = –21.39 %; Rate = –10.61 % yr–1; Perc Final = 53 % > decrease

DECADAL: NO (6 yr)

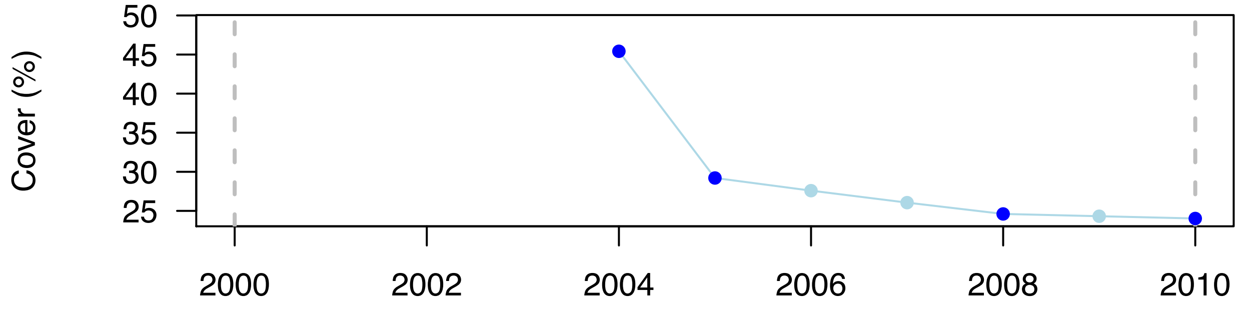

156_density

Romero et al. 2010 (a)

SITE: Rovellada (Spain – Mediterranean) – Po (–14 m)

OVERALL: Net = 32.3 shoot m–2; Rate = 2.33 % yr–1; Perc Final = 115 % > no change

DECADAL: NO (6 yr)

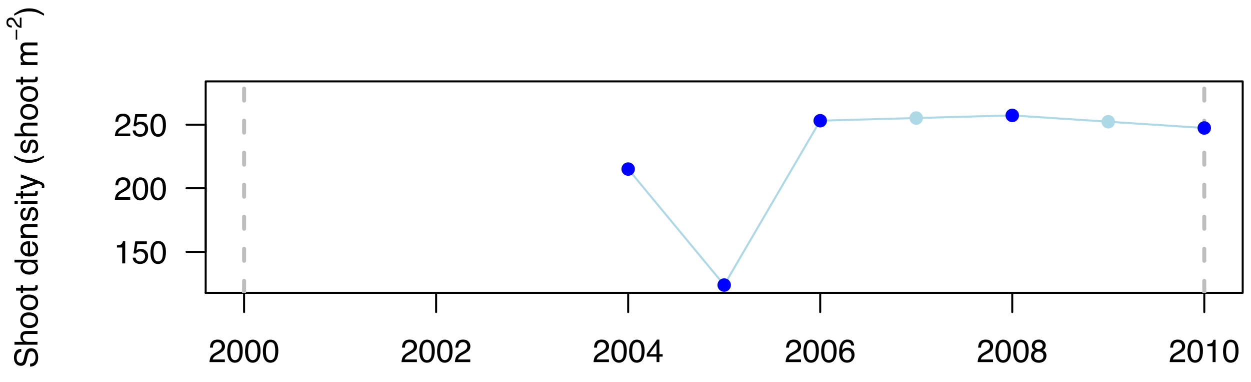

157_cover

Romero et al. 2010 (a)

SITE: Sa Tuna (Spain – Mediterranean) – Po (–14 m)

OVERALL: Net = 22.66 %;   Rate = 13.56 % yr–1; Perc Final = 258 % > increase

DECADAL: NO (7 yr)

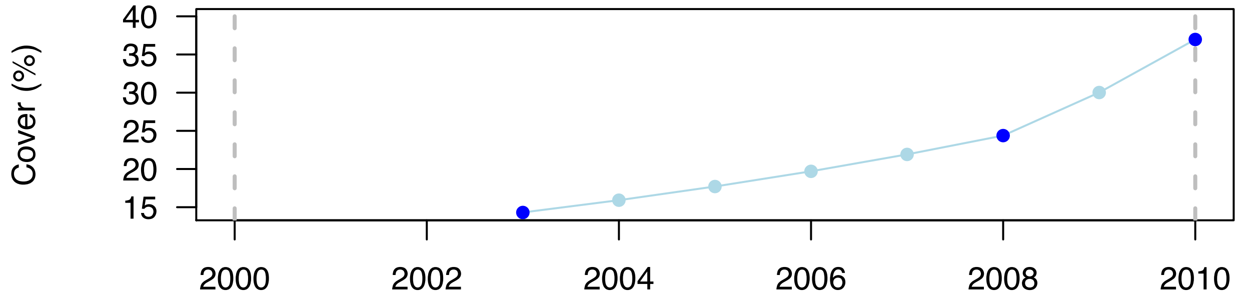

157_density

Romero et al. 2010 (a)

SITE: Sa Tuna (Spain – Mediterranean) – Po (–14 m)

OVERALL: Net = 15.1 shoot m–2;   Rate = 0.76 % yr–1; Perc Final = 105 % > no change

DECADAL: NO (7 yr)

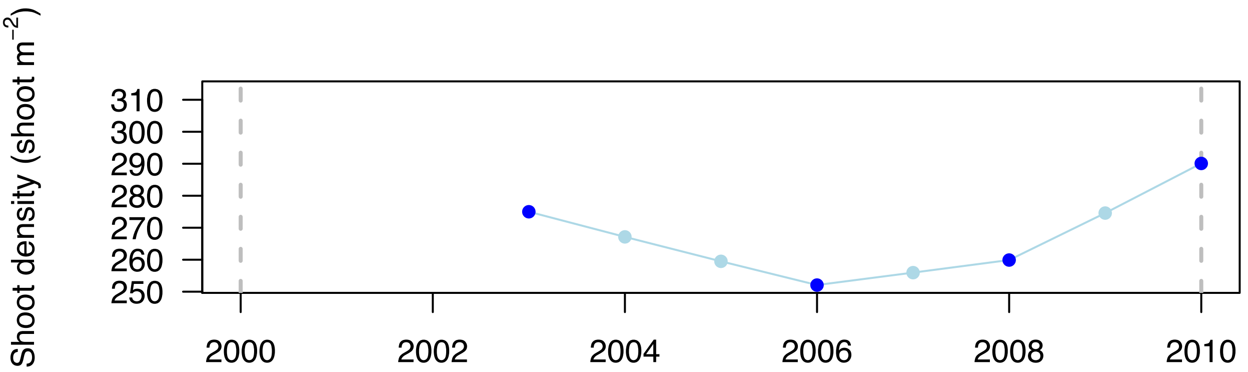

158_cover

Romero et al. 2010 (a)

SITE: Salou (Spain – Mediterranean) – Po (–14.5 m)

OVERALL: Net = 3.22 %;   Rate = 6.85 % yr–1; Perc Final = 162 % > increase

DECADAL: NO (7 yr)

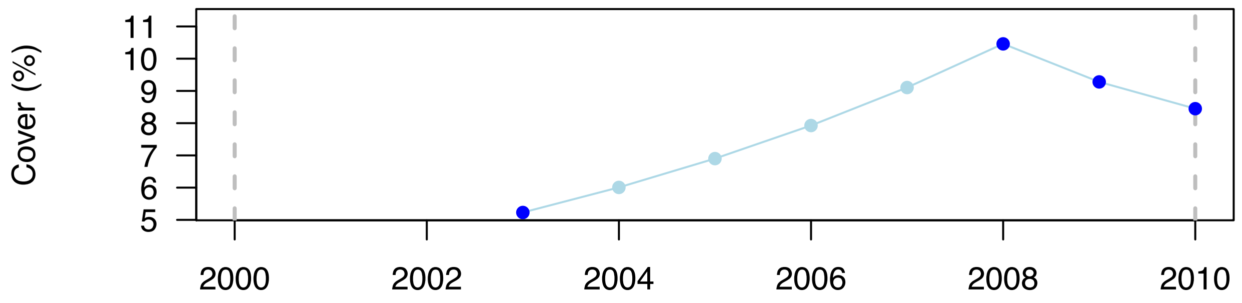

158_density

Romero et al. 2010 (a)

SITE: Salou (Spain – Mediterranean) – Po (–14.5 m)

OVERALL: Net = –76.04 shoot m–2;   Rate = –10.64 % yr–1; Perc Final = 47 % > decrease

DECADAL: NO (7 yr)

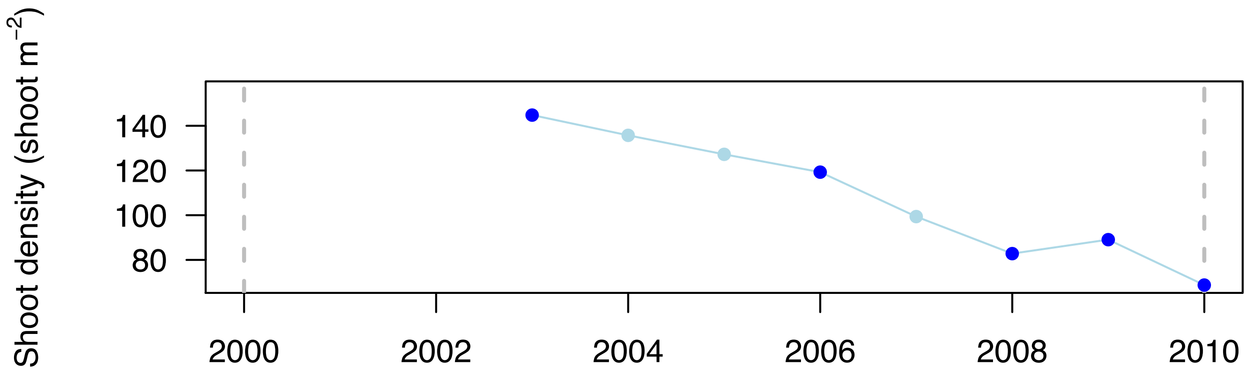

159_cover

Romero et al. 2010 (a)

SITE: Sant Feliu (Spain – Mediterranean) – Po (–14.5 m)

OVERALL: Net = 1.14 %; Rate = 0.44 % yr–1; Perc Final = 103 % > no change

DECADAL: NO (7 yr)

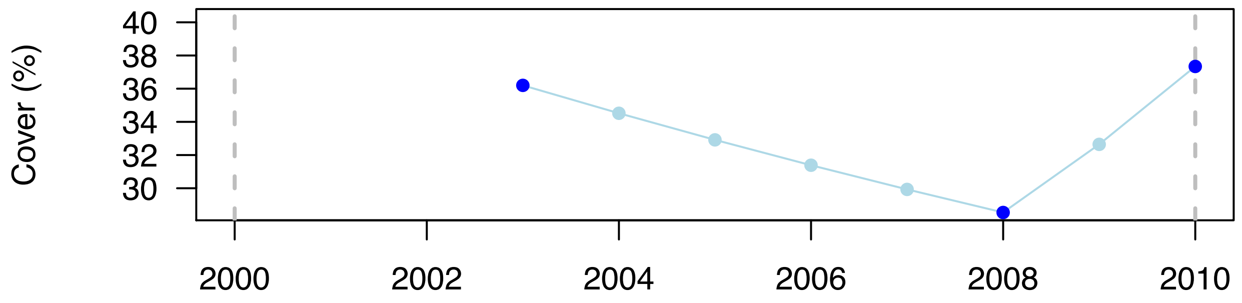

159_density

Romero et al. 2010 (a)

SITE: Sant Feliu (Spain – Mediterranean) – Po (–14.5 m)

OVERALL: Net = –28.65 shoot m–2; Rate = –1.54 % yr–1; Perc Final = 90 % > no change

DECADAL: NO (7 yr)

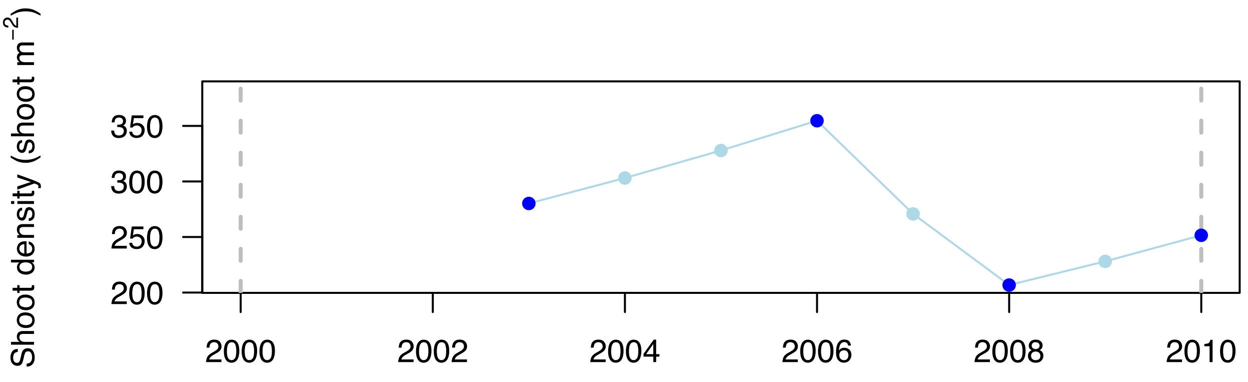

160_cover

SITE: Sitges (Spain – Mediterranean) – Po (–16.5 m)

OVERALL: Net = 12.52 %;   Rate = 32.79 % yr–1; Perc Final = 1378 % > increase

DECADAL: YES (8 yr)

Romero et al. 2010 (a)

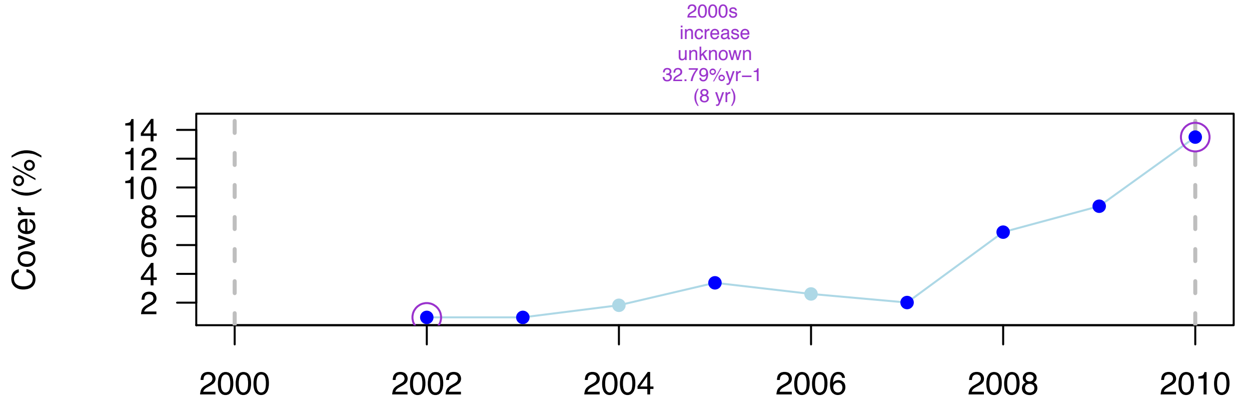

160_density

SITE: Sitges (Spain – Mediterranean) – Po (–16.5 m)

OVERALL: Net = 139.07 shoot m–2;   Rate = 15.24 % yr–1; Perc Final = 338 % > increase

DECADAL: YES (8 yr)

Romero et al. 2010 (a)

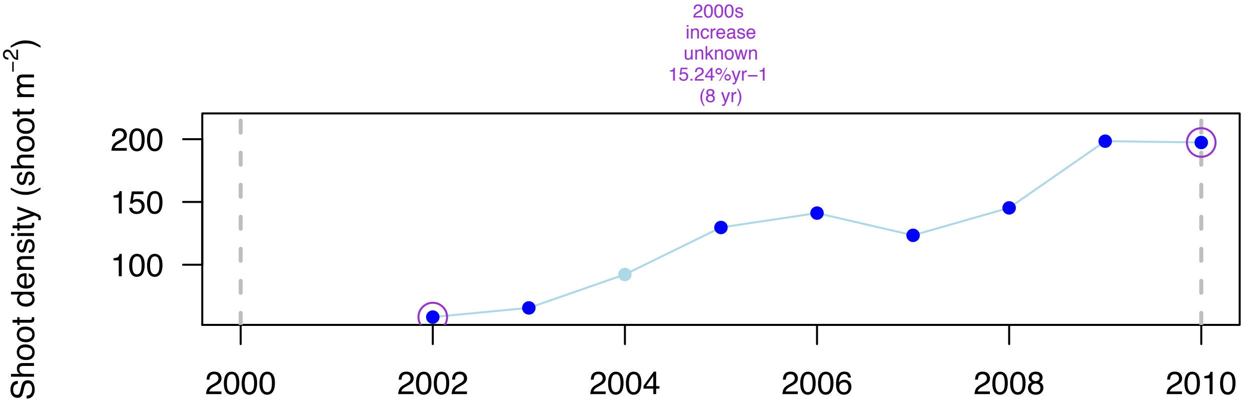

161_cover

Romero et al. 2010 (a)

SITE: Tamariua (Spain – Mediterranean) – Po (–15 m)

OVERALL: Net = 0.55 %;   Rate = 0.27 % yr–1; Perc Final = 102 % > no change

DECADAL: NO (6 yr)

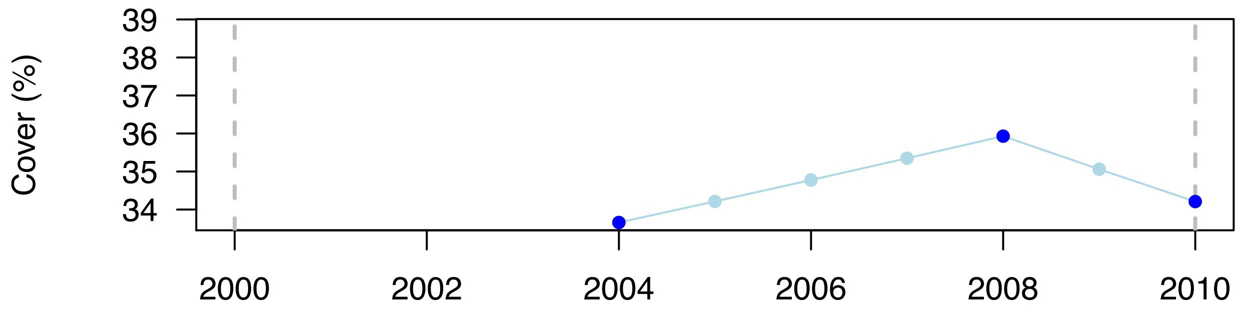

161_density

Romero et al. 2010 (a)

SITE: Tamariua (Spain – Mediterranean) – Po (–15 m)

OVERALL: Net = –37.5 shoot m–2;   Rate = –2.65 % yr–1; Perc Final = 85 % > no change

DECADAL: NO (6 yr)

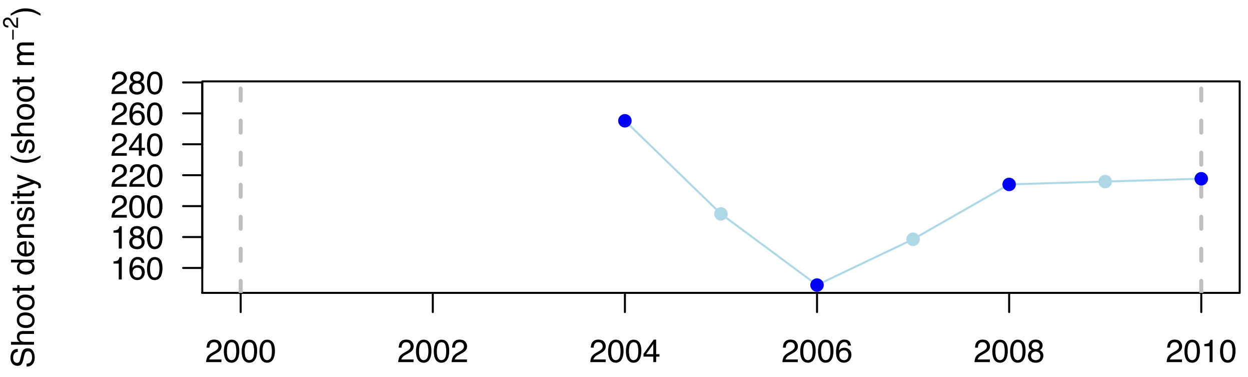

162_cover

Romero et al. 2010 (a)

SITE: Torredembarra (Spain – Mediterranean) – Po (–15 m)

OVERALL: Net = 19.63 %;   Rate = 20.71 % yr–1; Perc Final = 426 % > increase

DECADAL: NO (7 yr)

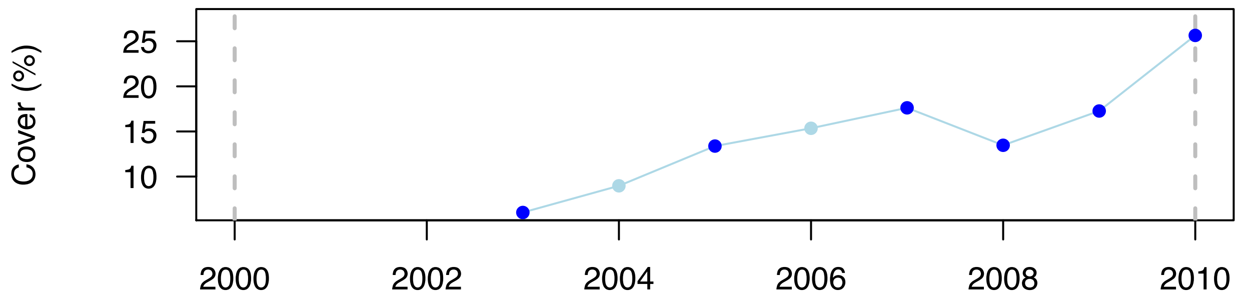

162_density

Romero et al. 2010 (a)

SITE: Torredembarra (Spain – Mediterranean) – Po (–15 m)

OVERALL: Net = 7.81 shoot m–2;   Rate = 0.69 % yr–1; Perc Final = 105 % > no change

DECADAL: NO (7 yr)

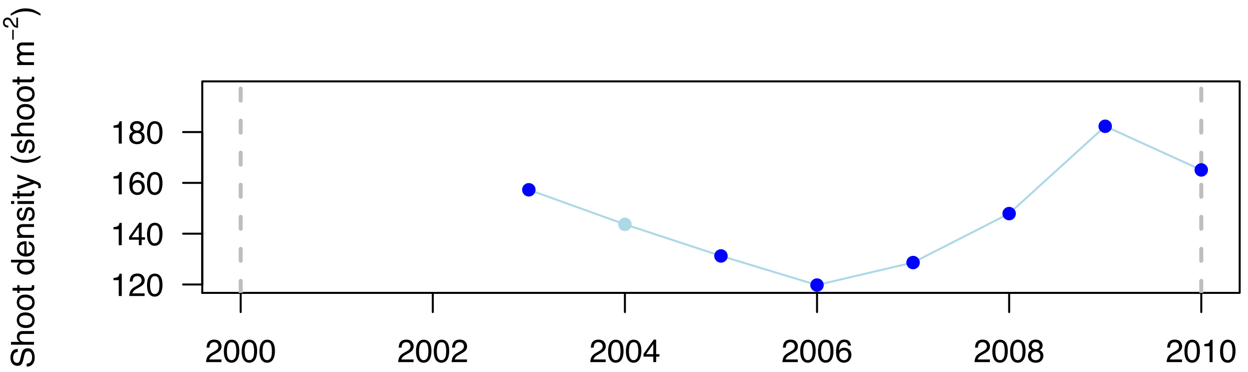

163_cover

Romero et al. 2010 (a)

SITE: Tossa de Mar (Spain – Mediterranean) – Po (–14 m)

OVERALL: Net = 19.65 %;   Rate = 9.71 % yr–1; Perc Final = 197 % > increase

DECADAL: NO (7 yr)

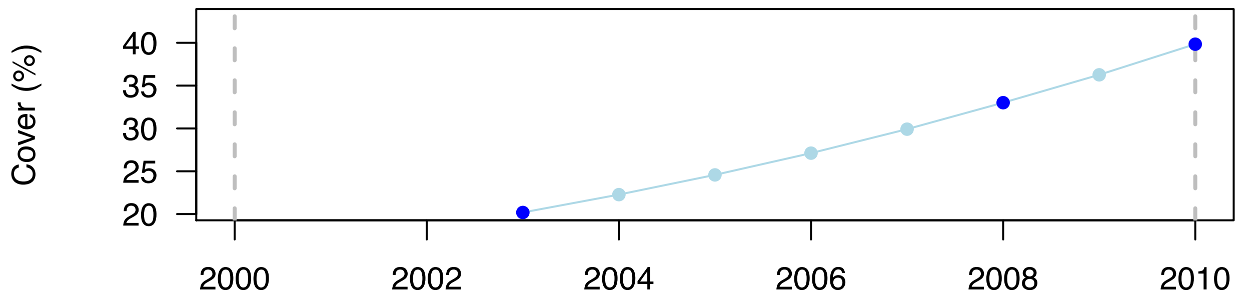

163_density

Romero et al. 2010 (a)

SITE: Tossa de Mar (Spain – Mediterranean) – Po (–14 m)

OVERALL: Net = –94.79 shoot m–2;   Rate = –5.45 % yr–1; Perc Final = 68 % > decrease

DECADAL: NO (7 yr)

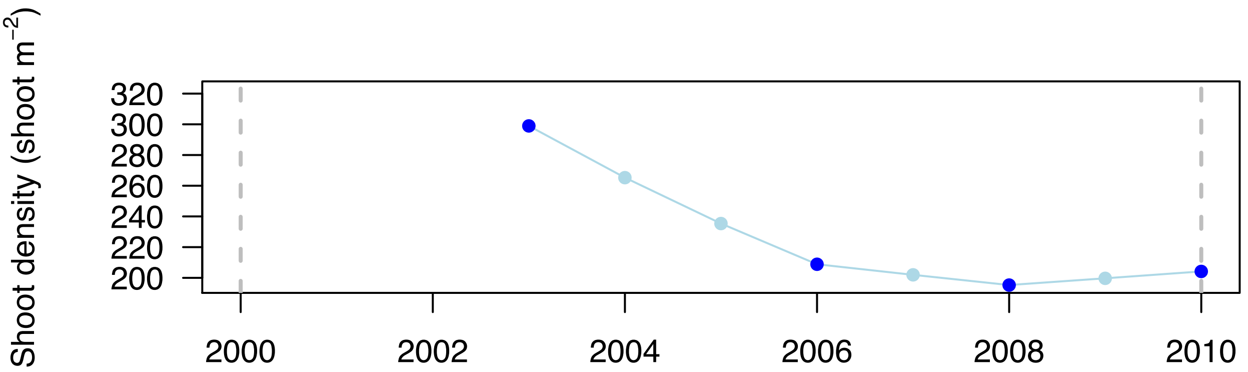

164_cover

Romero et al. 2010 (a)

SITE: Vilanova (Spain – Mediterranean) – Po (–17.5 m)

OVERALL: Net = 8.96 %;   Rate = 5.88 % yr–1; Perc Final = 142 % > increase

DECADAL: NO (6 yr)

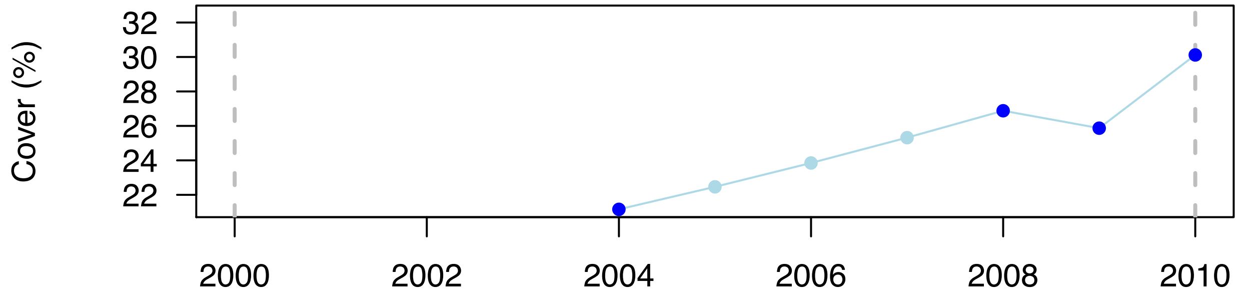

164_density

Romero et al. 2010 (a)

SITE: Vilanova (Spain – Mediterranean) – Po (–17.5 m)

OVERALL: Net = 76.04 shoot m–2;   Rate = 7.01 % yr–1; Perc Final = 152 % > increase

DECADAL: NO (6 yr)

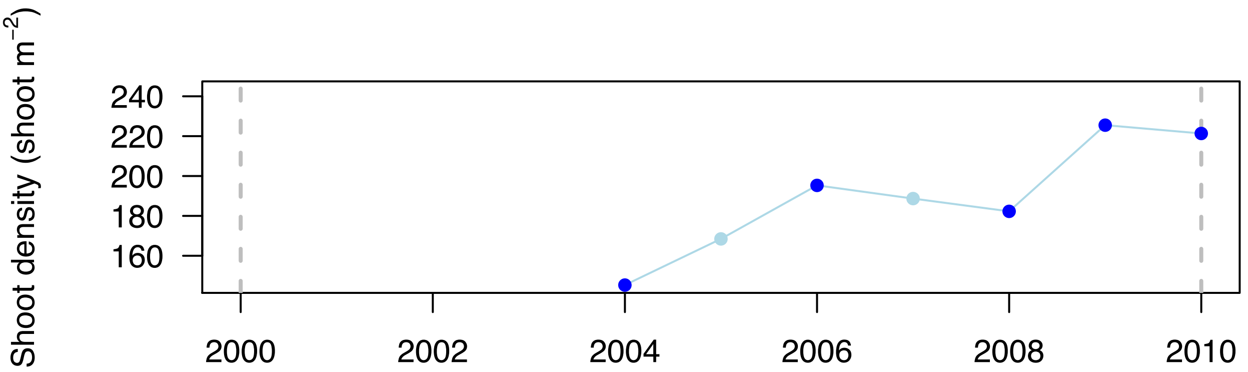

165_cover

Romero et al. 2010 (a)

SITE: Cadaqués (Spain – Mediterranean) – Po (–13.8 m)

OVERALL: Net = 0.71 %;   Rate = 1.4 % yr–1; Perc Final = 103 % > no change

DECADAL: NO (2 yr)

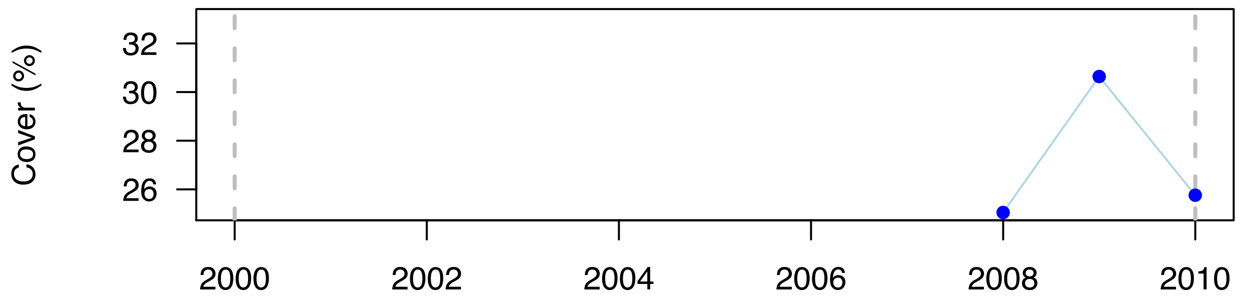

165_density

Romero et al. 2010 (a)

SITE: Cadaqués (Spain – Mediterranean) – Po (–13.8 m)

OVERALL: Net = 93.23 shoot m–2;   Rate = 34.11 % yr–1; Perc Final = 198 % > increase

DECADAL: NO (2 yr)

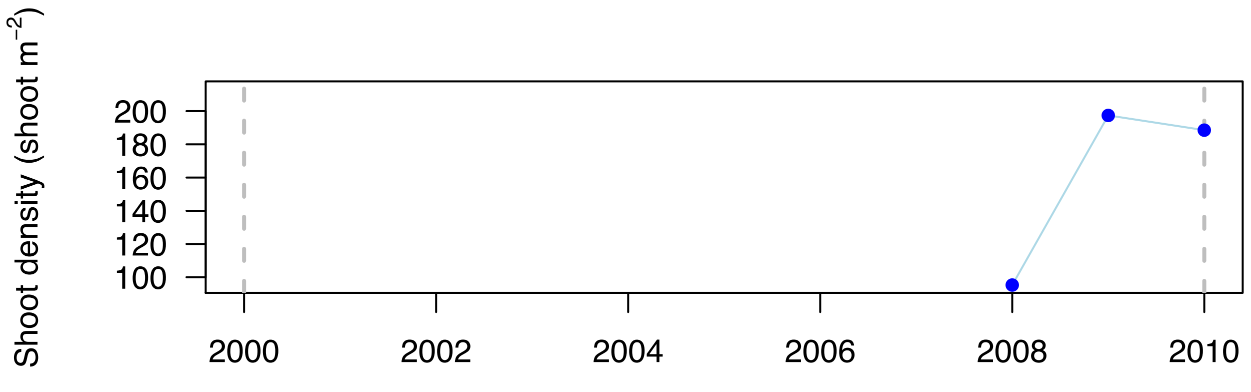

166_area

den Hartog and Polderman 1975

SITE: Western Dutch Wadden Sea (The Netherlands – Atlantic) – Zm (–2 m)

OVERALL: Net = –7190 ha; Rate = NA % yr–1; Perc Final = NA % > decrease

DECADAL: YES (103 yr)

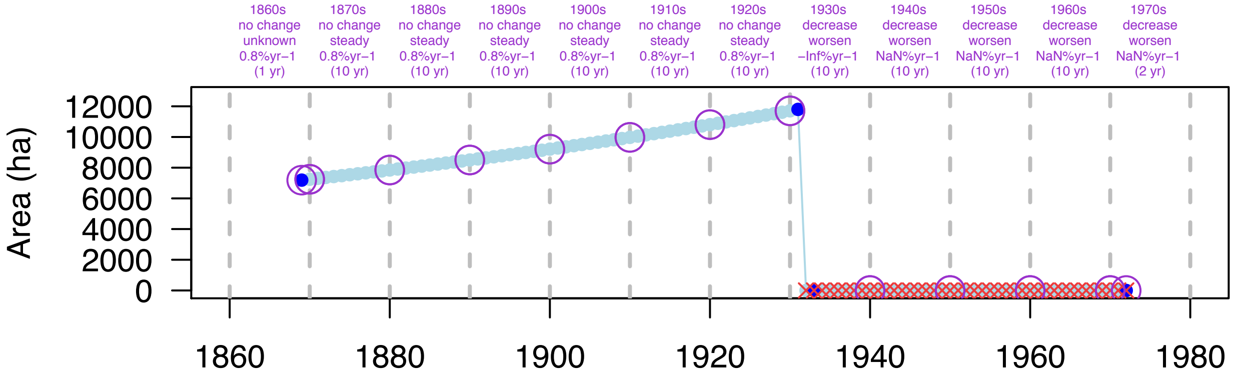

167_cover

Ruiz et al. 2013

SITE: Cala Túnez (Spain – Mediterranean) – Po (–7 m)

OVERALL: Net = 1.06 %; Rate = 0.27 % yr–1; Perc Final = 102 % > no change

DECADAL: YES (9 yr)

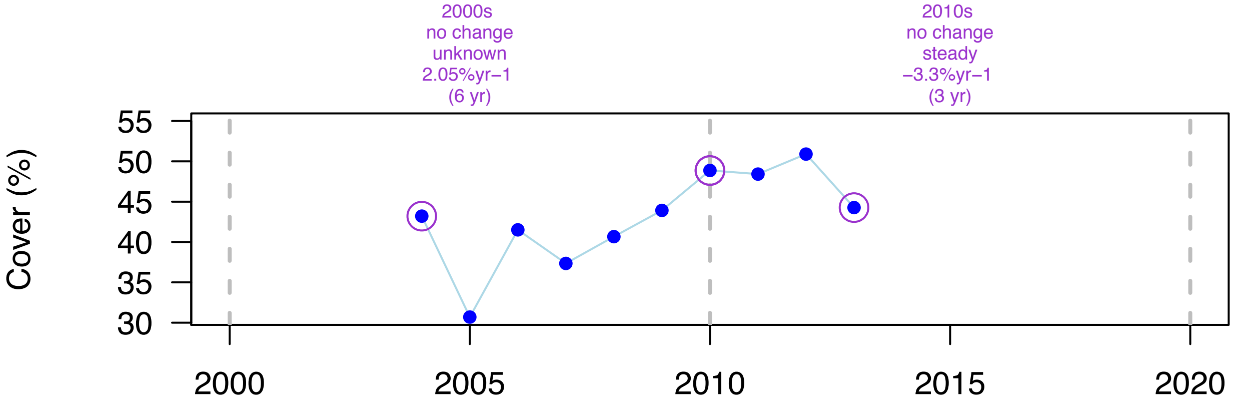

167_density

Ruiz et al. 2013

SITE: Cala Túnez (Spain – Mediterranean) – Po (–7 m)

OVERALL: Net = 16.38 shoot m–2; Rate = 5.49 % yr–1; Perc Final = 164 % > increase

DECADAL: YES (9 yr)

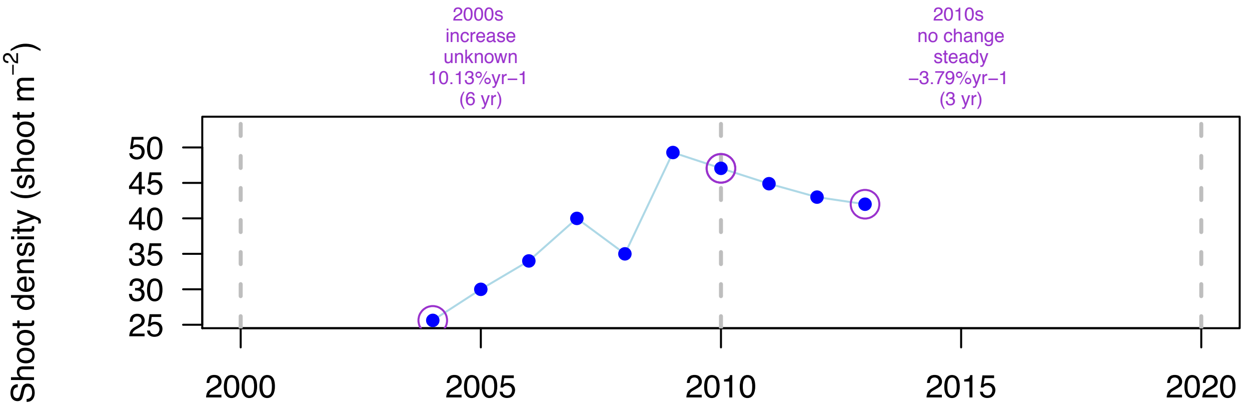

168_cover

Ruiz et al. 2013

SITE: Cala Escalera (Spain – Mediterranean) – Po (–17 m)

OVERALL: Net = –8.44 %; Rate = –3.4 % yr–1; Perc Final = 76 % > no change

DECADAL: YES (8 yr)

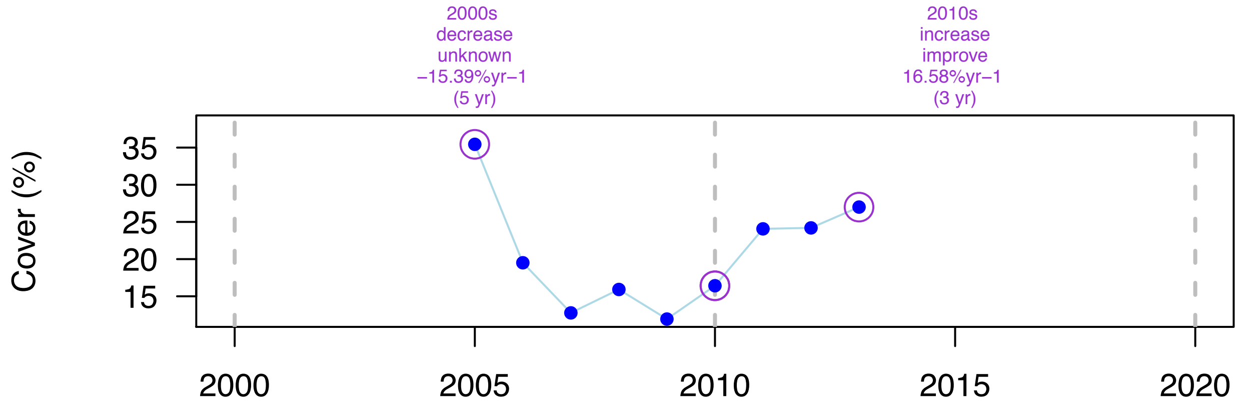

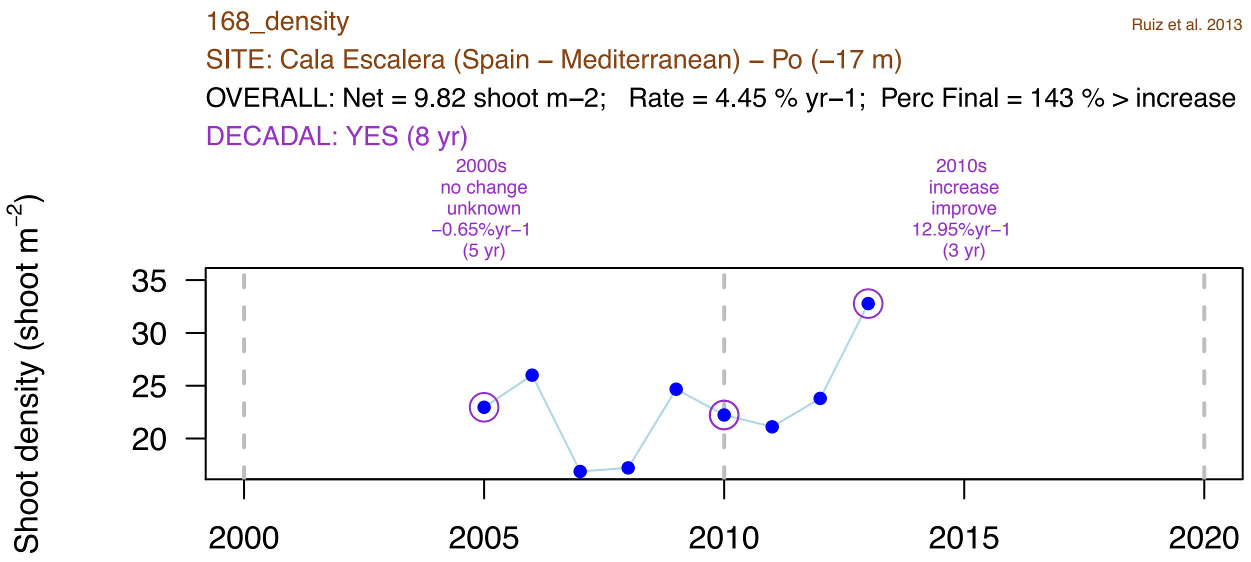
168_density
Ruiz et al. 2013
SITE: Cala Escalera (Spain – Mediterranean) – Po (–17 m)
OVERALL: Net = 9.82 shoot m–2; Rate = 4.45 % yr–1; Perc Final = 143 % > increase
DECADAL: YES (8 yr)
2000s
no change
unknown
–0.65%yr–1
(5 yr)
2010s
increase
improve
12.95%yr–1
(3 yr)
Shoot density (shoot m-2)
35
30
25
20
2000
2005
2010
2015
2020

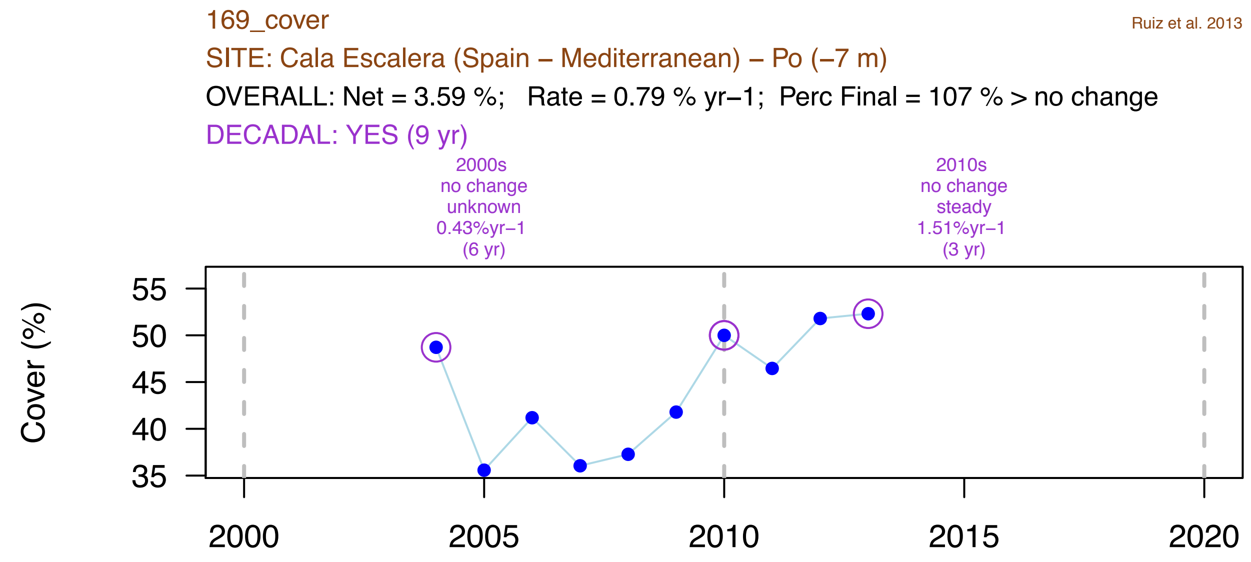
169_cover
Ruiz et al. 2013
SITE: Cala Escalera (Spain – Mediterranean) – Po (–7 m)
OVERALL: Net = 3.59 %; Rate = 0.79 % yr–1; Perc Final = 107 % > no change
DECADAL: YES (9 yr)
2000s
no change
unknown
0.43%yr–1
(6 yr)
2010s
no change
steady
1.51%yr–1
(3 yr)
Cover (%)
55
50
45
40
35
2000
2005
2010
2015
2020

169_density

Ruiz et al. 2013

SITE: Cala Escalera (Spain – Mediterranean) – Po (–7 m)

OVERALL: Net = 21.07 shoot m–2;   Rate = 5.39 % yr–1; Perc Final = 163 % > increase

DECADAL: YES (9 yr)

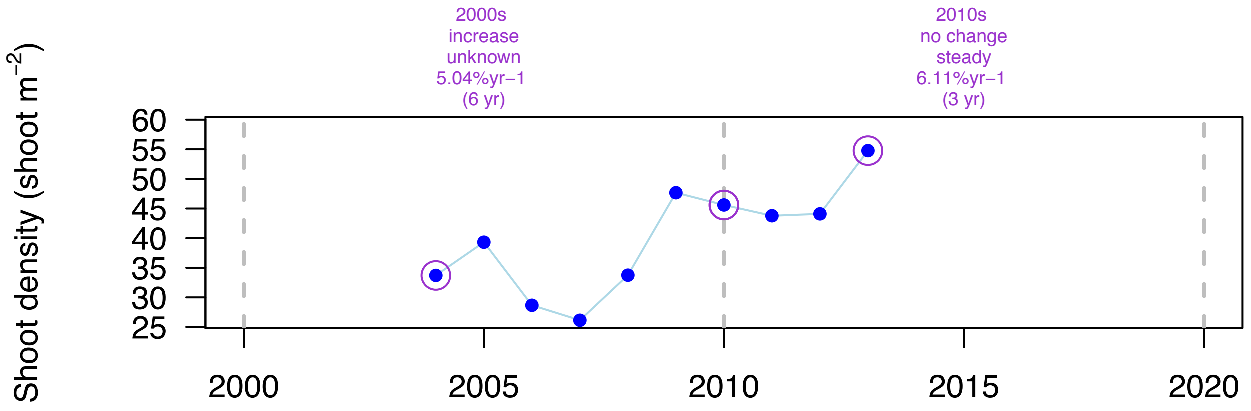

170_cover

Ruiz et al. 2013

SITE: Cala Cerrada (Spain – Mediterranean) – Po (–22 m)

OVERALL: Net = –7.25 %;   Rate = –5.07 % yr–1; Perc Final = 63 % > decrease

DECADAL: YES (9 yr)

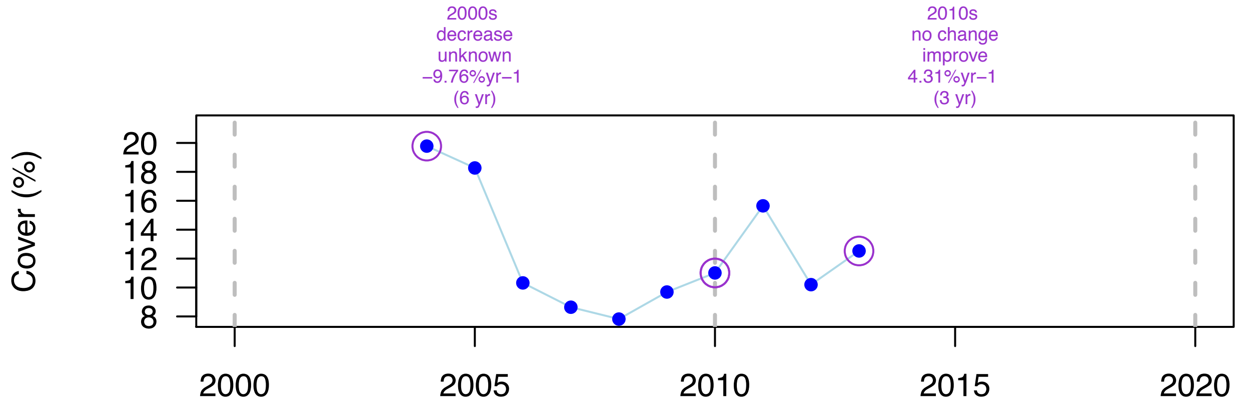

170_density

Ruiz et al. 2013

SITE: Cala Cerrada (Spain – Mediterranean) – Po (–22 m)

OVERALL: Net = –3.74 shoot m–2;   Rate = –3.43 % yr–1; Perc Final = 73 % > decrease

DECADAL: YES (9 yr)

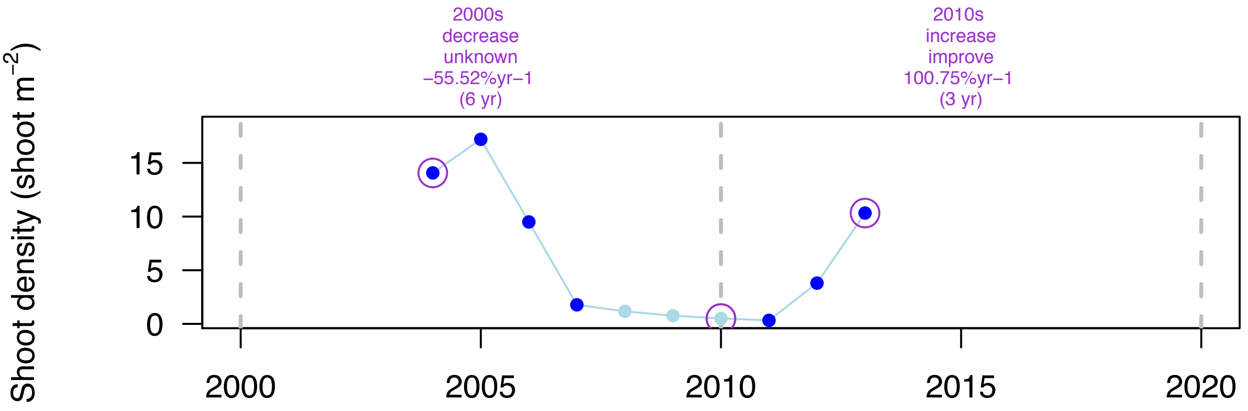

171_cover

Ruiz et al. 2013

SITE: Cala Cerrada (Spain – Mediterranean) – Po (–12 m)

OVERALL: Net = –3.08 %;   Rate = –1.39 % yr–1; Perc Final = 88 % > no change

DECADAL: YES (9 yr)

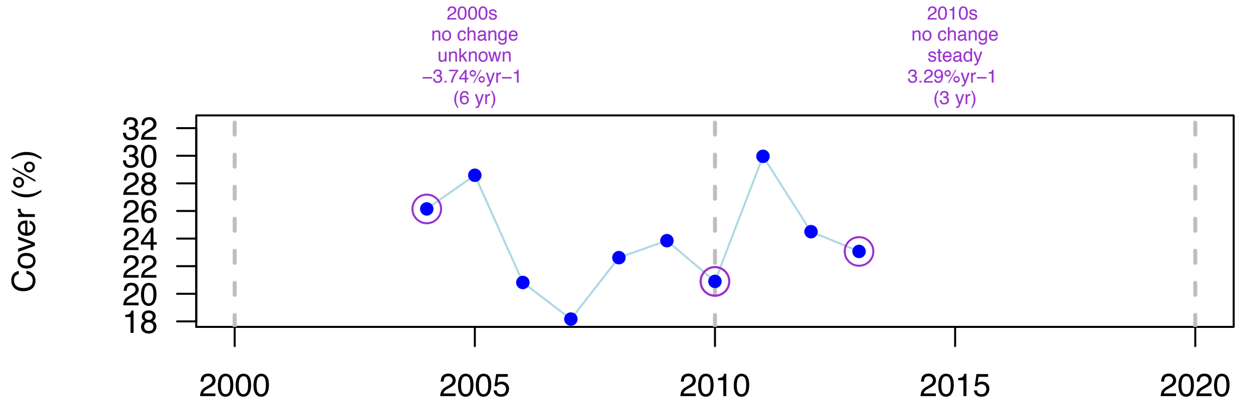

171_density

Ruiz et al. 2013

SITE: Cala Cerrada (Spain – Mediterranean) – Po (–12 m)

OVERALL: Net = –0.88 shoot m–2;   Rate = –0.4 % yr–1; Perc Final = 96 % > no change

DECADAL: YES (9 yr)

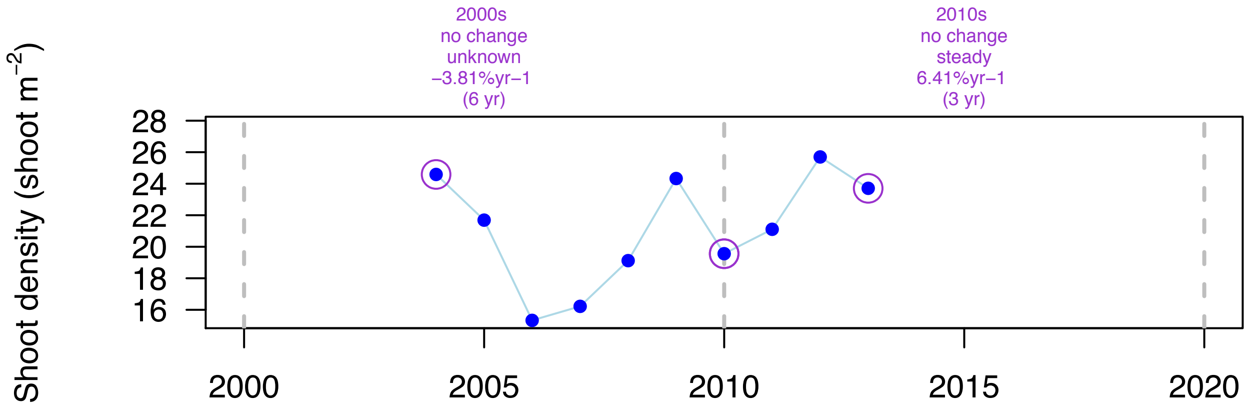

172_cover

Ruiz et al. 2013

SITE: La Azohía (Spain – Mediterranean) – Po (–17 m)

OVERALL: Net = 0.39 %;   Rate = 0.16 % yr–1; Perc Final = 101 % > no change

DECADAL: YES (9 yr)

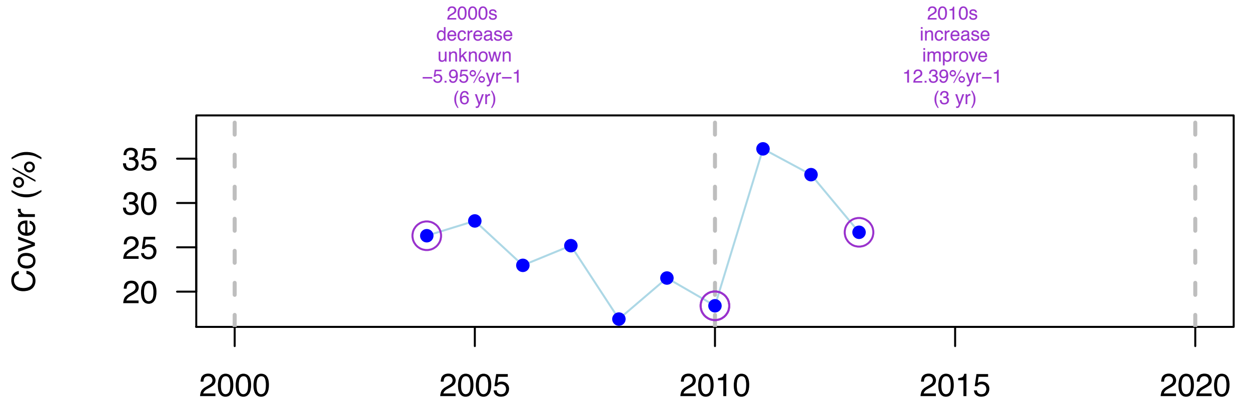

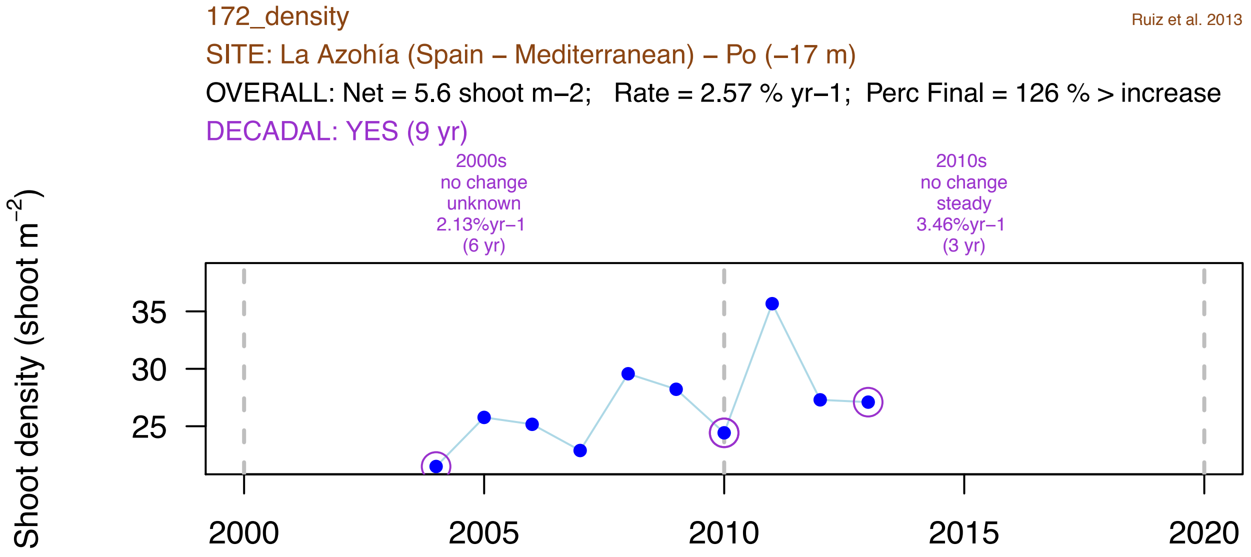
172_density
Ruiz et al. 2013
SITE: La Azohía (Spain – Mediterranean) – Po (–17 m)
OVERALL: Net = 5.6 shoot m–2; Rate = 2.57 % yr–1; Perc Final = 126 % > increase
DECADAL: YES (9 yr)
2000s
no change
unknown
2.13%yr–1
(6 yr)
2010s
no change
steady
3.46%yr–1
(3 yr)
Shoot density (shoot m⁻²)
35
30
25
2000
2005
2010
2015
2020

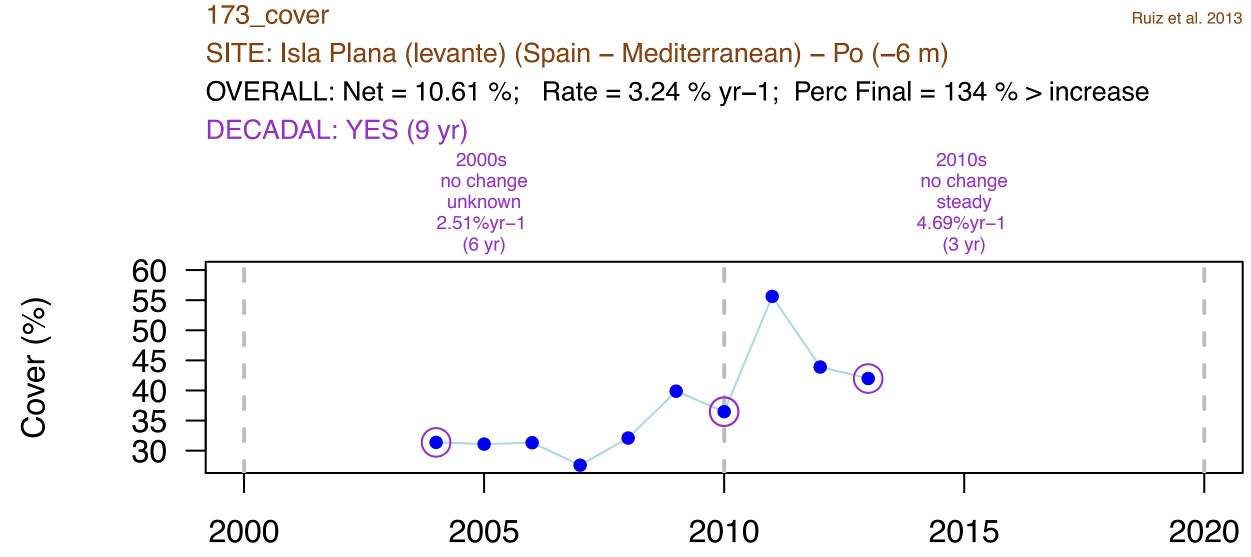
173_cover
Ruiz et al. 2013
SITE: Isla Plana (levante) (Spain – Mediterranean) – Po (–6 m)
OVERALL: Net = 10.61 %; Rate = 3.24 % yr–1; Perc Final = 134 % > increase
DECADAL: YES (9 yr)
2000s
no change
unknown
2.51%yr–1
(6 yr)
2010s
no change
steady
4.69%yr–1
(3 yr)
Cover (%)
60
55
50
45
40
35
30
2000
2005
2010
2015
2020

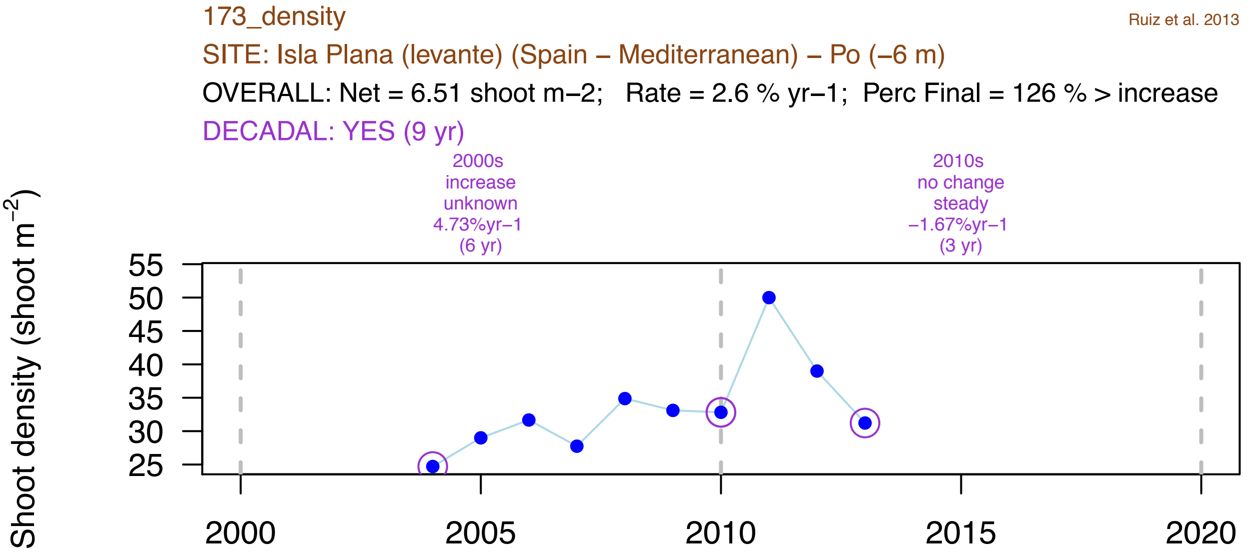
173_density
Ruiz et al. 2013
SITE: Isla Plana (levante) (Spain – Mediterranean) – Po (–6 m)
OVERALL: Net = 6.51 shoot m–2; Rate = 2.6 % yr–1; Perc Final = 126 % > increase
DECADAL: YES (9 yr)
2000s
increase
unknown
4.73%yr–1
(6 yr)
2010s
no change
steady
–1.67%yr–1
(3 yr)
Shoot density (shoot m-2)
55
50
45
40
35
30
25
2000
2005
2010
2015
2020

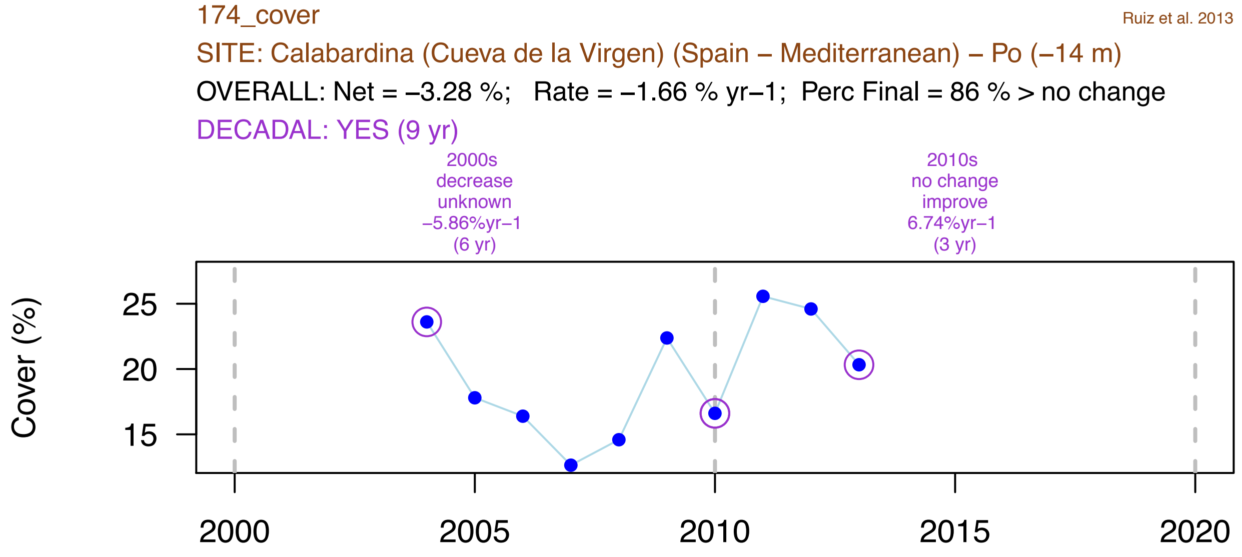
174_cover
Ruiz et al. 2013
SITE: Calabardina (Cueva de la Virgen) (Spain – Mediterranean) – Po (–14 m)
OVERALL: Net = –3.28 %; Rate = –1.66 % yr–1; Perc Final = 86 % > no change
DECADAL: YES (9 yr)
2000s
decrease
unknown
–5.86%yr–1
(6 yr)
2010s
no change
improve
6.74%yr–1
(3 yr)
Cover (%)
25
20
15
2000
2005
2010
2015
2020

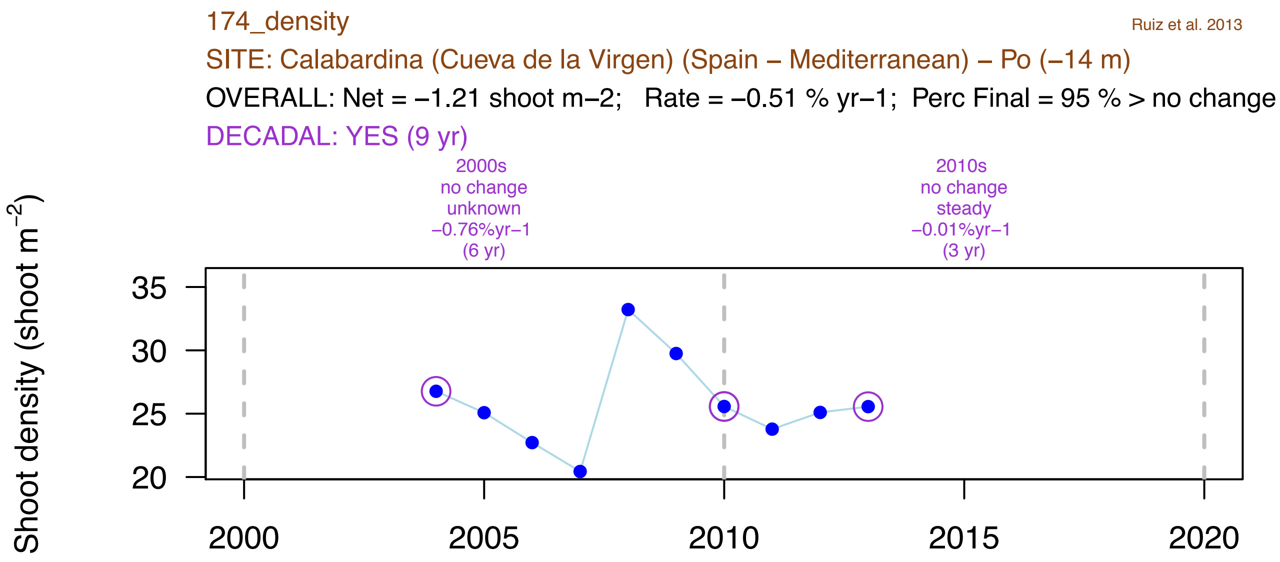

174_density
Ruiz et al. 2013
SITE: Calabardina (Cueva de la Virgen) (Spain – Mediterranean) – Po (–14 m)
OVERALL: Net = –1.21 shoot m–2; Rate = –0.51 % yr–1; Perc Final = 95 % > no change
DECADAL: YES (9 yr)
2000s
no change
unknown
–0.76%yr–1
(6 yr)
2010s
no change
steady
–0.01%yr–1
(3 yr)
Shoot density (shoot m^{-2})
35
30
25
20
2000
2005
2010
2015
2020

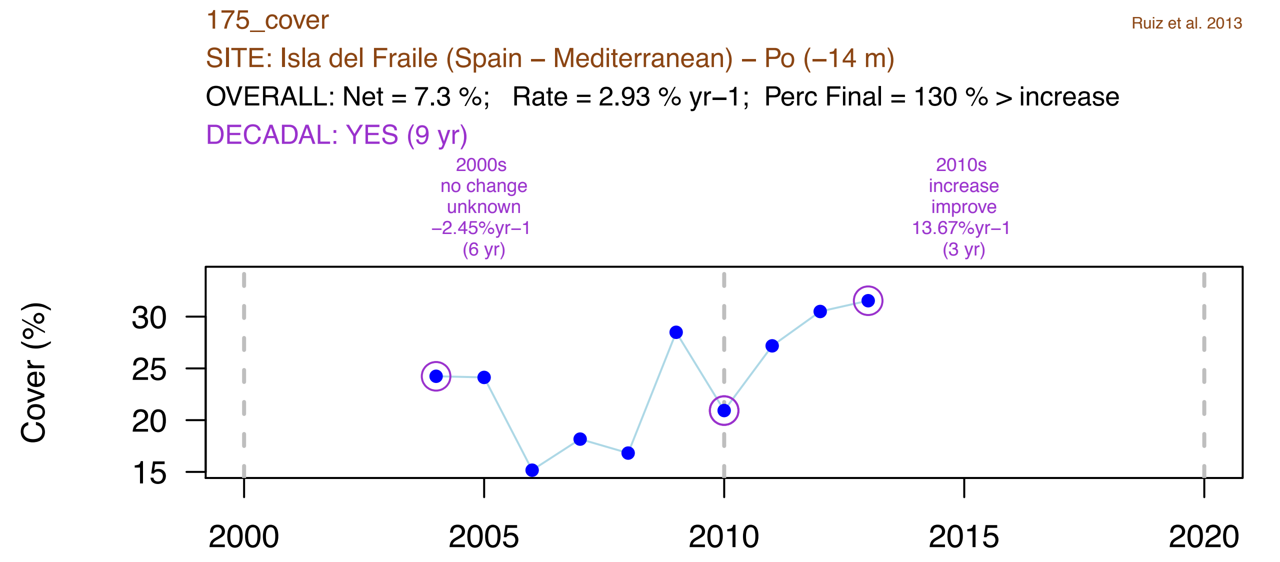

175_cover
Ruiz et al. 2013
SITE: Isla del Fraile (Spain – Mediterranean) – Po (–14 m)
OVERALL: Net = 7.3 %; Rate = 2.93 % yr–1; Perc Final = 130 % > increase
DECADAL: YES (9 yr)
2000s
no change
unknown
–2.45%yr–1
(6 yr)
2010s
increase
improve
13.67%yr–1
(3 yr)
Cover (%)
30
25
20
15
2000
2005
2010
2015
2020

175_density

Ruiz et al. 2013

SITE: Isla del Fraile (Spain – Mediterranean) – Po (–14 m)

OVERALL: Net = –8.08 shoot m–2;   Rate = –3.52 % yr–1; Perc Final = 73 % > decrease

DECADAL: YES (9 yr)

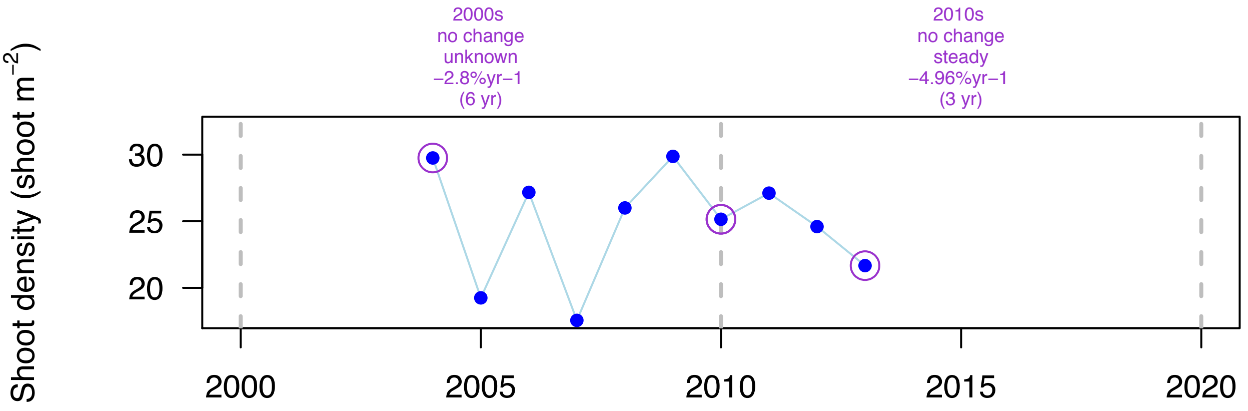

176_cover

Ruiz et al. 2013

SITE: Isla Plana (poniente) (Spain – Mediterranean) – Po (–2 m)

OVERALL: Net = 13.27 %;   Rate = 2.44 % yr–1; Perc Final = 125 % > no change

DECADAL: YES (9 yr)

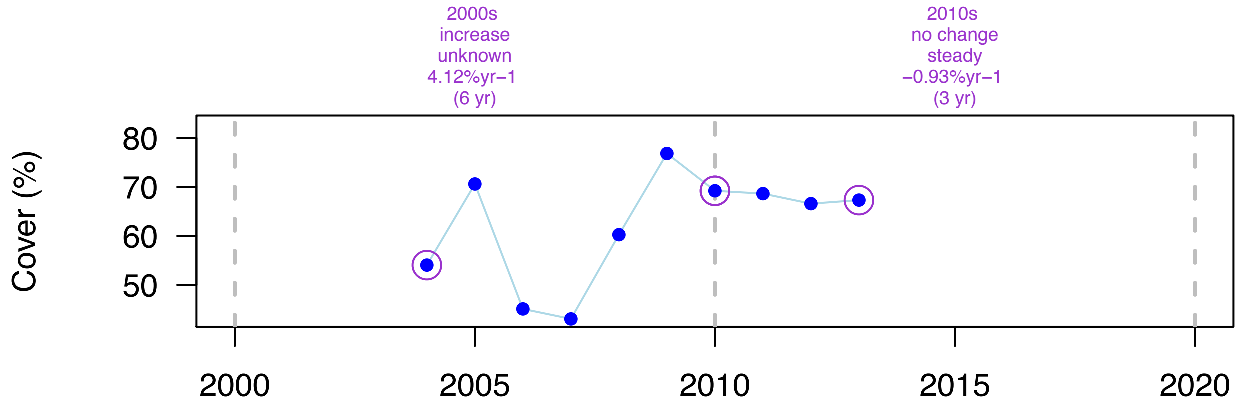

176_density

Ruiz et al. 2013

SITE: Isla Plana (poniente) (Spain – Mediterranean) – Po (–2 m)

OVERALL: Net = 8.34 shoot m–2;   Rate = 1.66 % yr–1; Perc Final = 116 % > no change

DECADAL: YES (9 yr)

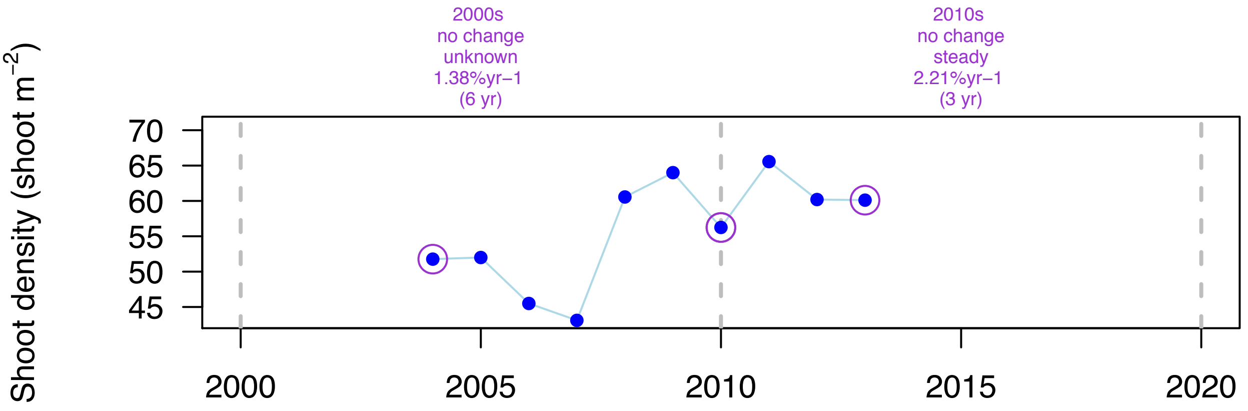

177_cover

Ruiz et al. 2013

SITE: Isla Grosa (Spain – Mediterranean) – Po (–4 m)

OVERALL: Net = 17.78 %;   Rate = 4.95 % yr–1; Perc Final = 149 % > increase

DECADAL: YES (8 yr)

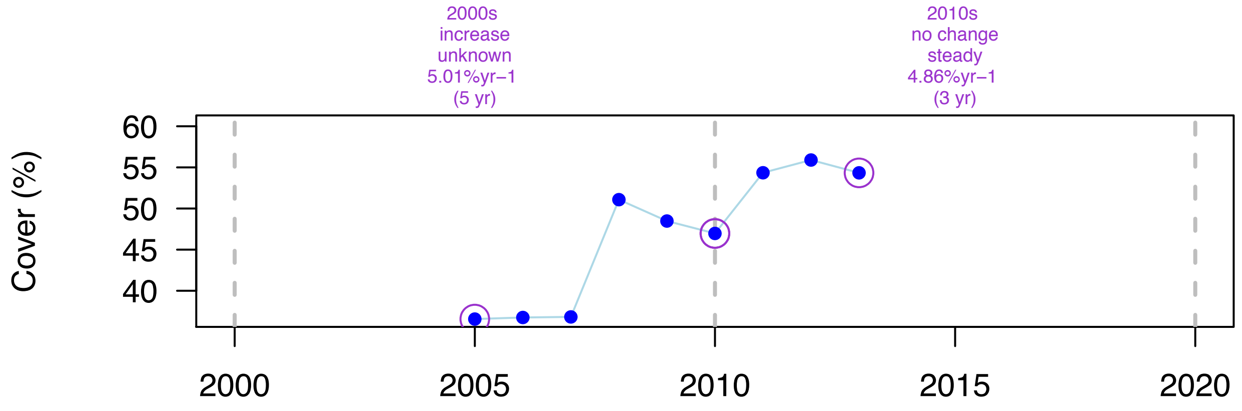

177_density
Ruiz et al. 2013

SITE: Isla Grosa (Spain – Mediterranean) – Po (–4 m)

OVERALL: Net = 6.74 shoot m–2;   Rate = 2.44 % yr–1; Perc Final = 119 % > no change

DECADAL: NO (7 yr)

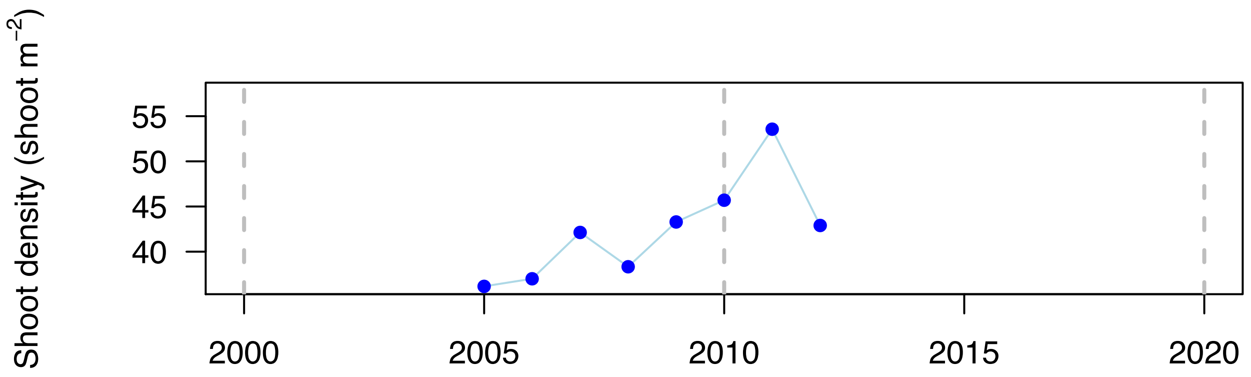

178_cover
Ruiz et al. 2013

SITE: Isla Grosa (Spain – Mediterranean) – Po (–12 m)

OVERALL: Net = 9.08 %;   Rate = 4.57 % yr–1; Perc Final = 132 % > increase

DECADAL: NO (6 yr)

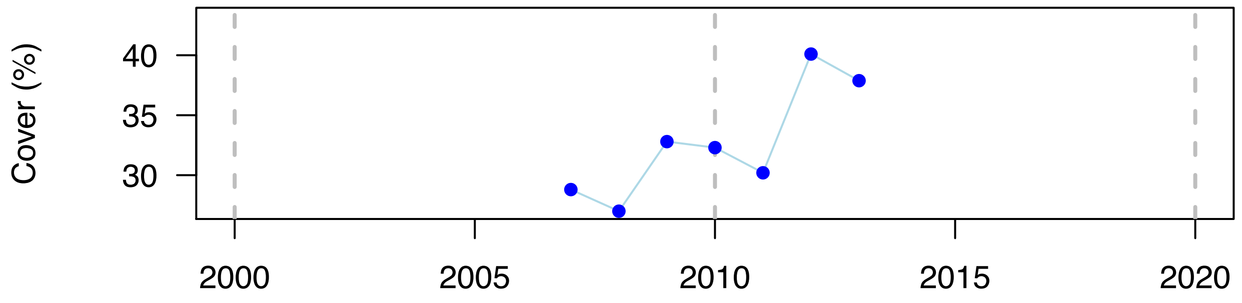

178_density

Ruiz et al. 2013

SITE: Isla Grosa (Spain – Mediterranean) – Po (–12 m)

OVERALL: Net = –1.67 shoot m–2; Rate = –1.06 % yr–1; Perc Final = 94 % > no change

DECADAL: NO (6 yr)

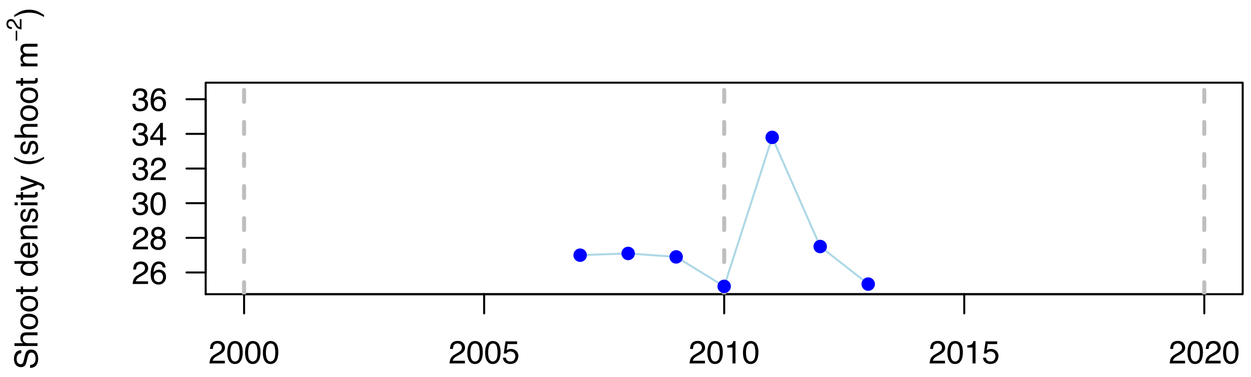

179_cover

Ruiz et al. 2013

SITE: Puerto de Tomás Maestre (Spain – Mediterranean) – Po (–4 m)

OVERALL: Net = –1.82 %; Rate = –0.47 % yr–1; Perc Final = 96 % > no change

DECADAL: YES (8 yr)

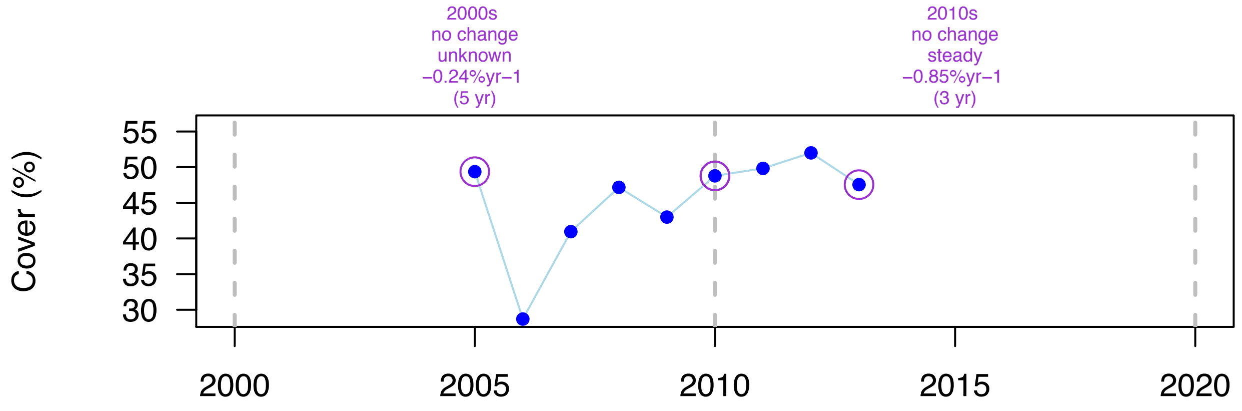

179_density Ruiz et al. 2013

SITE: Puerto de Tomás Maestre (Spain – Mediterranean) – Po (–4 m)

OVERALL: Net = 8.68 shoot m–2; Rate = 2.73 % yr–1; Perc Final = 124 % > no change

DECADAL: YES (8 yr)

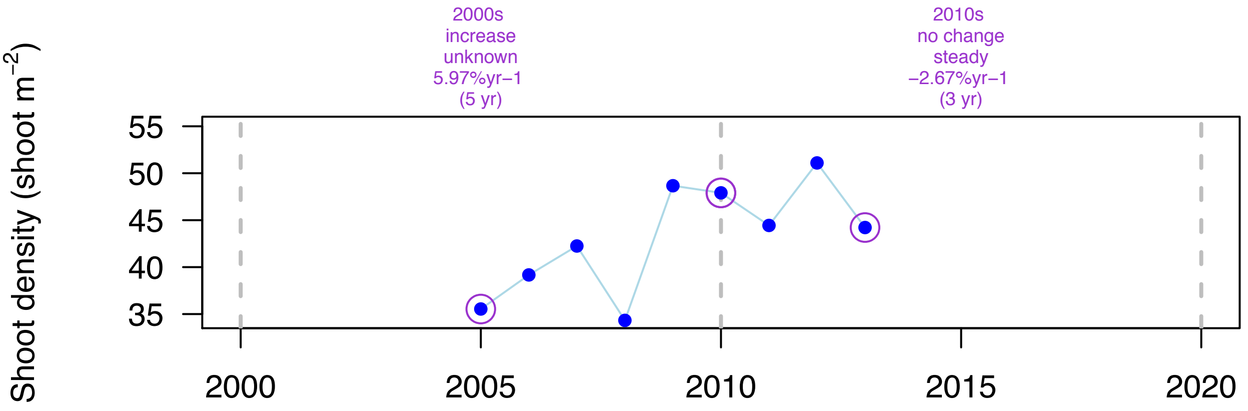

180_cover Ruiz et al. 2013

SITE: Isla de las Palomas (Spain – Mediterranean) – Po (–17 m)

OVERALL: Net = 0.33 %; Rate = 0.25 % yr–1; Perc Final = 102 % > no change

DECADAL: NO (7 yr)

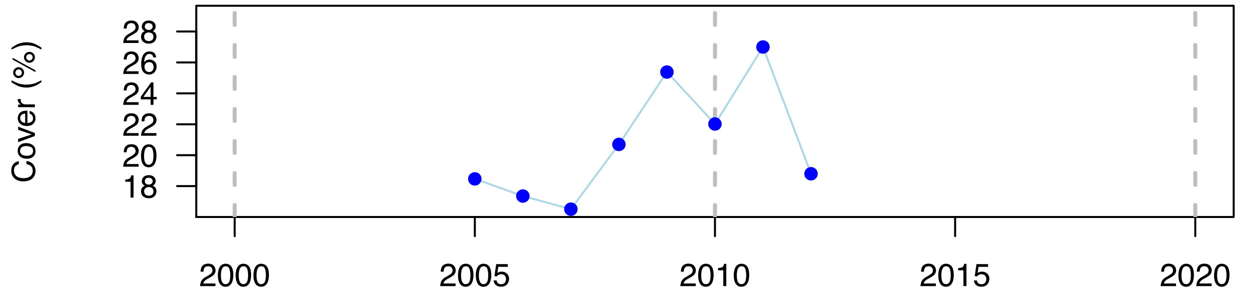

180_density

Ruiz et al. 2013

SITE: Isla de las Palomas (Spain – Mediterranean) – Po (–17 m)

OVERALL: Net = −2.53 shoot m−2;   Rate = −1.8 % yr−1; Perc Final = 88 % > no change

DECADAL: NO (7 yr)

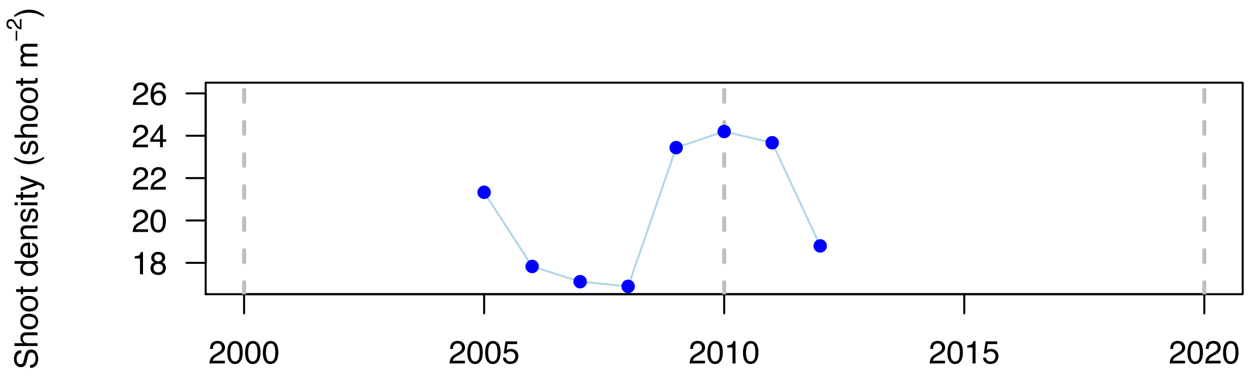

181_cover

Ruiz et al. 2013

SITE: Cala Reona (Spain – Mediterranean) – Po (–6 m)

OVERALL: Net = 0.09 %;   Rate = 0.04 % yr−1; Perc Final = 100 % > no change

DECADAL: NO (7 yr)

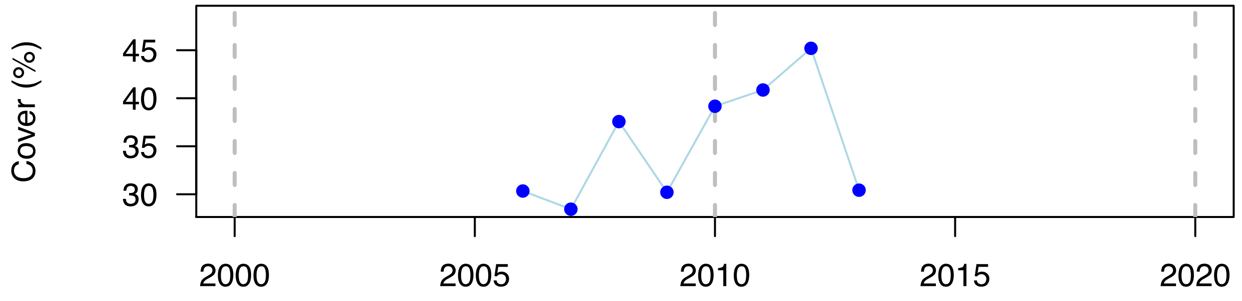

181_density

Ruiz et al. 2013

SITE: Cala Reona (Spain – Mediterranean) – Po (–6 m)

OVERALL: Net = 0.87 shoot m–2;   Rate = 0.29 % yr–1; Perc Final = 102 % > no change

DECADAL: NO (7 yr)

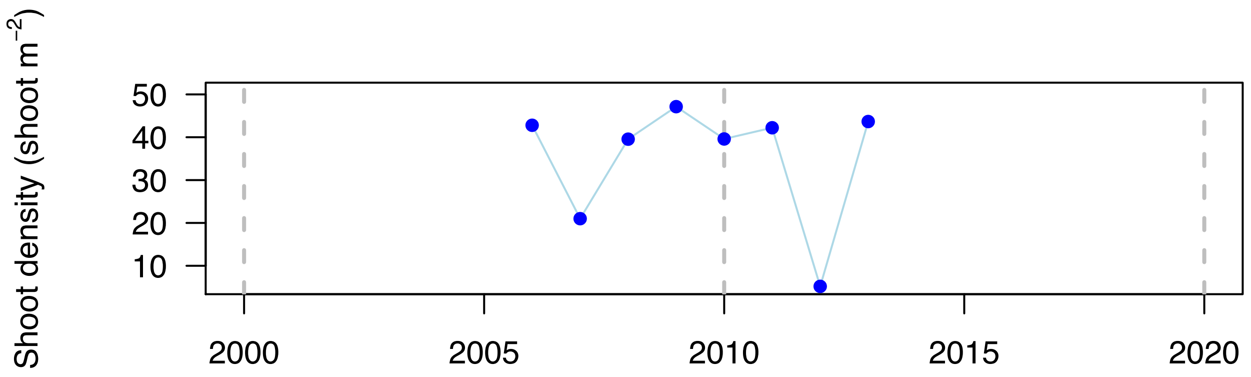

182_cover

Ruiz et al. 2013

SITE: Calblanque (Spain – Mediterranean) – Po (–26 m)

OVERALL: Net = 7.34 %;   Rate = 11.48 % yr–1; Perc Final = 199 % > increase

DECADAL: NO (6 yr)

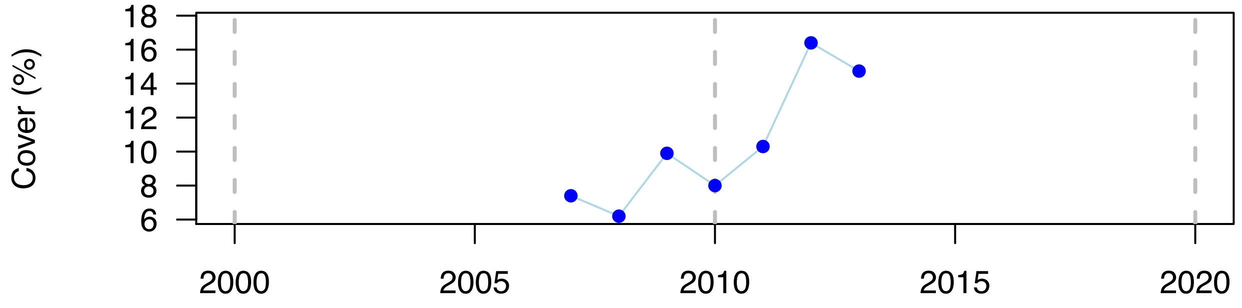

182_density

Ruiz et al. 2013

SITE: Calblanque (Spain – Mediterranean) – Po (–26 m)

OVERALL: Net = 9.35 shoot m–2;   Rate = 8.82 % yr–1; Perc Final = 170 % > increase

DECADAL: NO (6 yr)

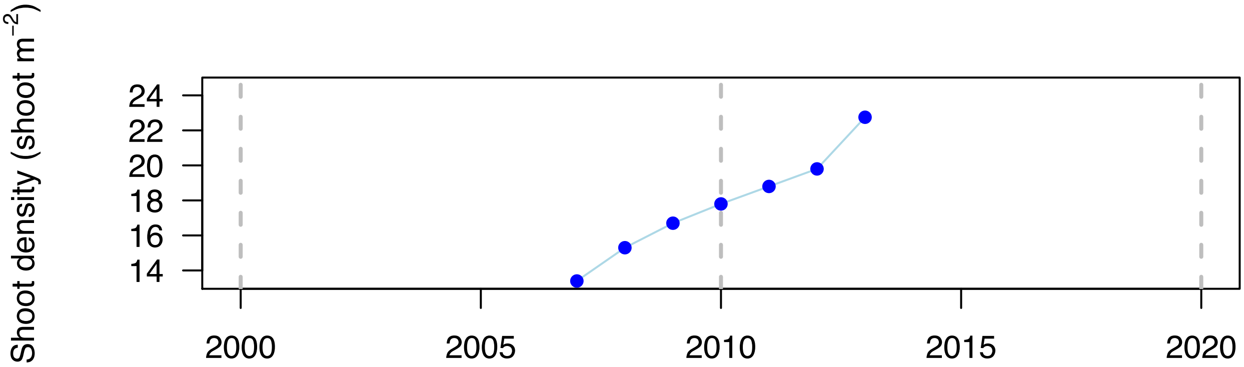

183_cover

Ruiz et al. 2013

SITE: El Muellecico (Cabo Tiñoso) (Spain – Mediterranean) – Po (–22 m)

OVERALL: Net = 8.08 %;   Rate = 10.34 % yr–1; Perc Final = 186 % > increase

DECADAL: NO (6 yr)

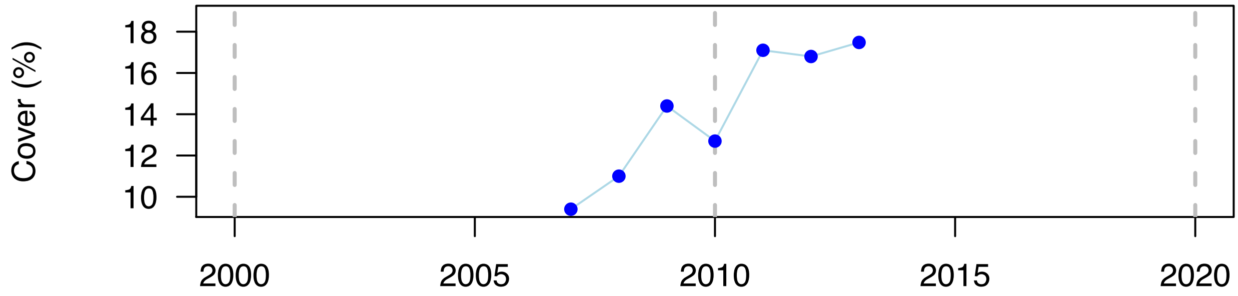

183_density Ruiz et al. 2013

SITE: El Muellecico (Cabo Tiñoso) (Spain – Mediterranean) – Po (–22 m)

OVERALL: Net = 4.57 shoot m–2; Rate = 4.28 % yr–1; Perc Final = 129 % > increase

DECADAL: NO (6 yr)

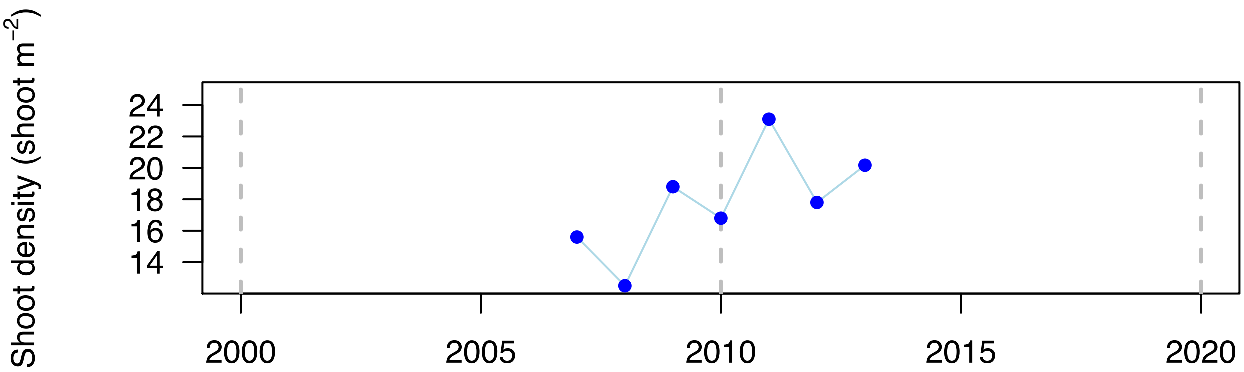

184_density Marbà and Duarte 2010, Duarte and Marbà (unpublished)

SITE: El Castell (Spain – Mediterranean) – Po (–5 m)

OVERALL: Net = –456 shoot m–2; Rate = –9.06 % yr–1; Perc Final = 37 % > decrease

DECADAL: YES (11 yr)

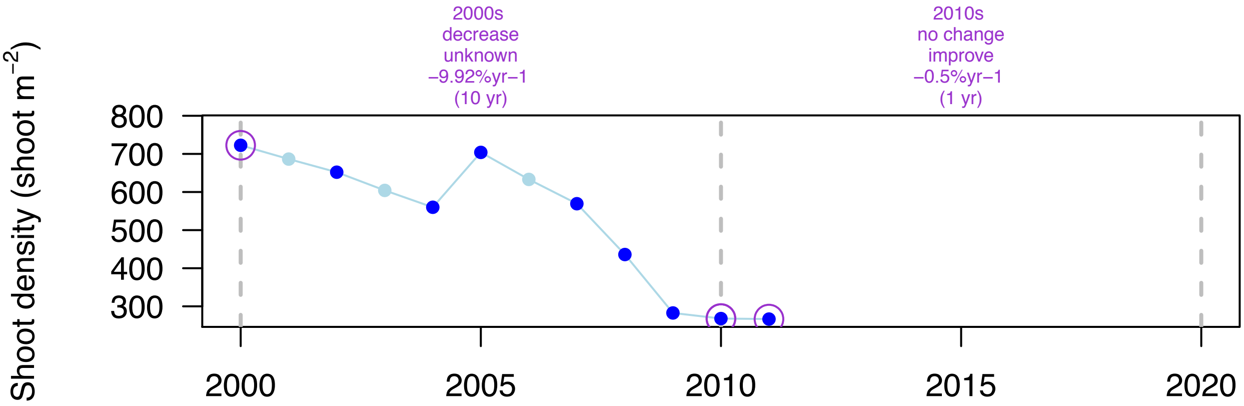

185_density

Marbà and Duarte 2010, Duarte and Marbà (unpublished)

SITE: Cala Santa María (Spain – Mediterranean) – Po (–13.1 m)

OVERALL: Net = –525.47 shoot m–2;   Rate = –10.64 % yr–1; Perc Final = 31 % > decrease

DECADAL: YES (11 yr)

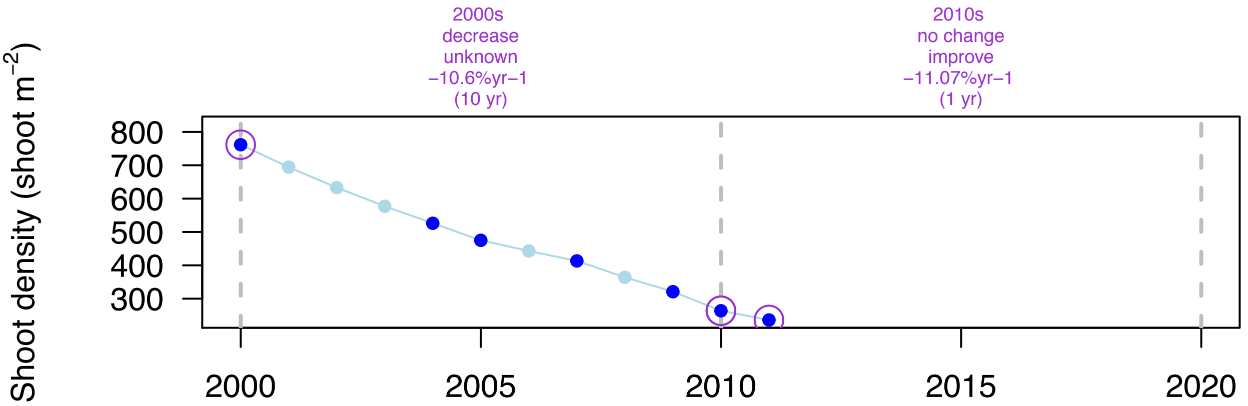

186_density

Duarte and Marbà (unpublished)

SITE: Mooring 22 (Spain – Mediterranean) – Po (–17 m)

OVERALL: Net = –142.73 shoot m–2;   Rate = –5.37 % yr–1; Perc Final = 55 % > decrease

DECADAL: YES (11 yr)

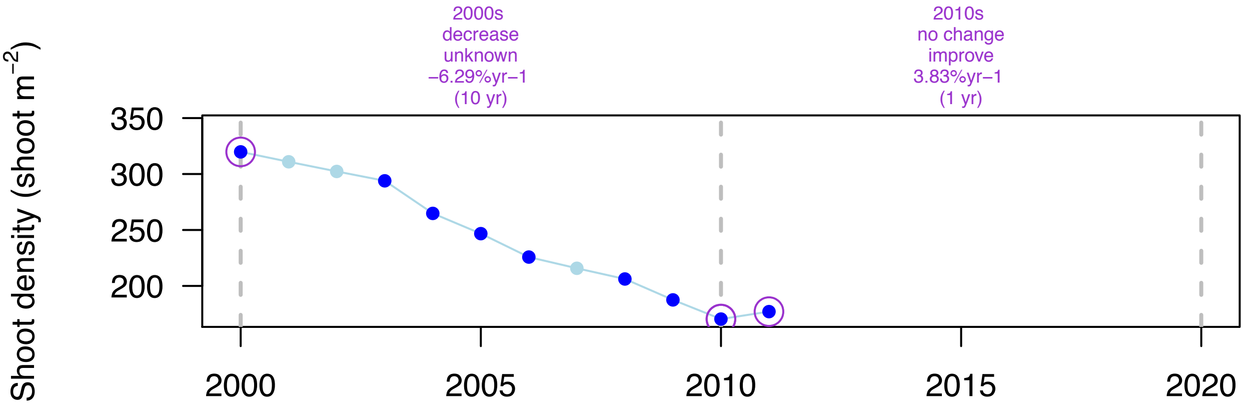

187_density

Duarte and Marbà (unpublished)

SITE: Porto Colom (Spain – Mediterranean) – Po (–6.4 m)

OVERALL: Net = 140 shoot m–2; Rate = 2.91 % yr–1; Perc Final = 134 % > increase

DECADAL: YES (10 yr)

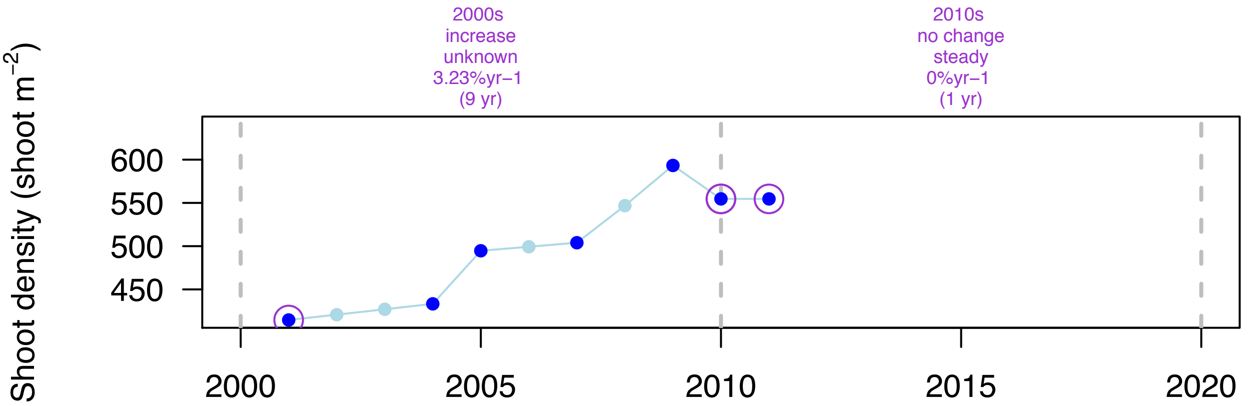

188_density

Duarte and Marbà (unpublished)

SITE: Pollença (Spain – Mediterranean) – Po (–4.1 m)

OVERALL: Net = –21.77 shoot m–2; Rate = –0.28 % yr–1; Perc Final = 97 % > no change

DECADAL: YES (10 yr)

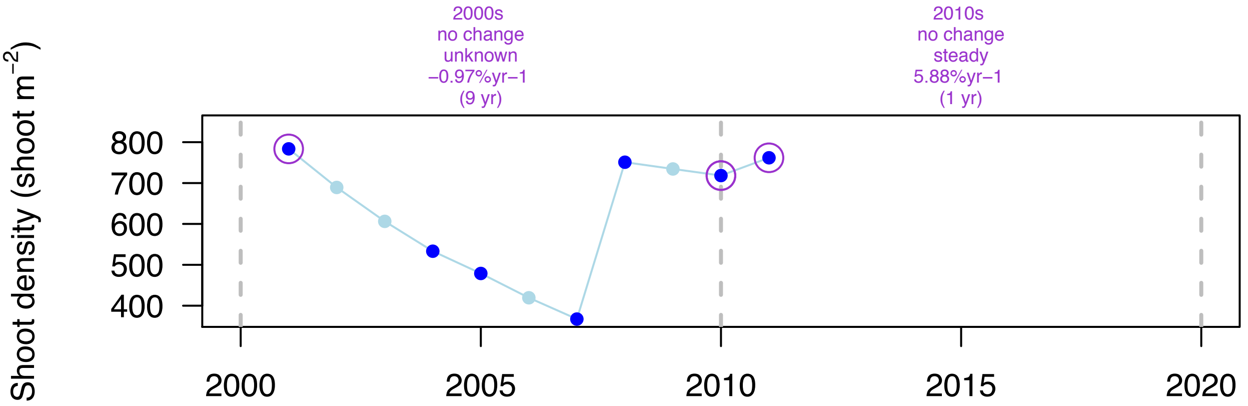

189_density

Duarte and Marbà (unpublished)

SITE: Cap Salines (Spain – Mediterranean) – Po (–25.5 m)

OVERALL: Net = –41.67 shoot m–2;   Rate = –1.14 % yr–1; Perc Final = 89 % > no change

DECADAL: YES (10 yr)

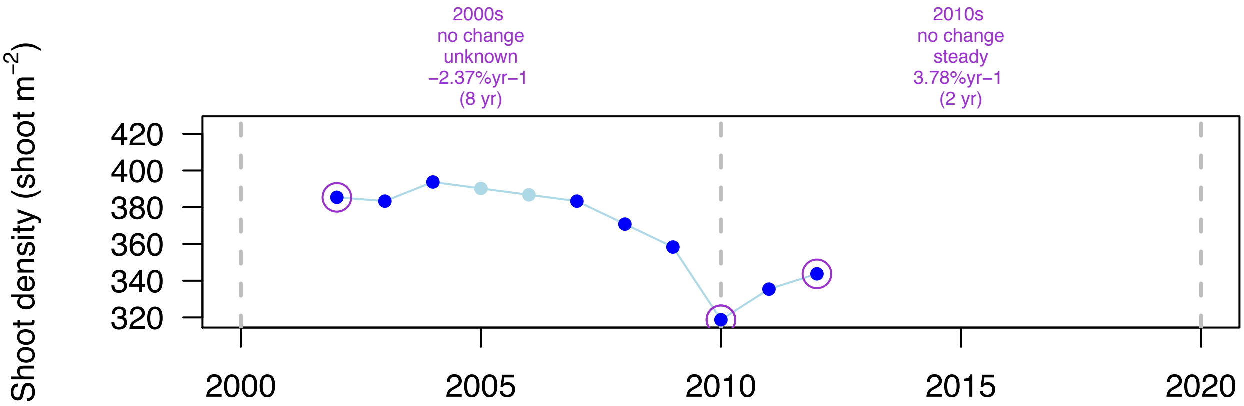

190_density

Duarte and Marbà (unpublished)

SITE: Cala Millor (Spain – Mediterranean) – Po (–6.5 m)

OVERALL: Net = 83.34 shoot m–2;   Rate = 1.4 % yr–1; Perc Final = 113 % > no change

DECADAL: YES (9 yr)

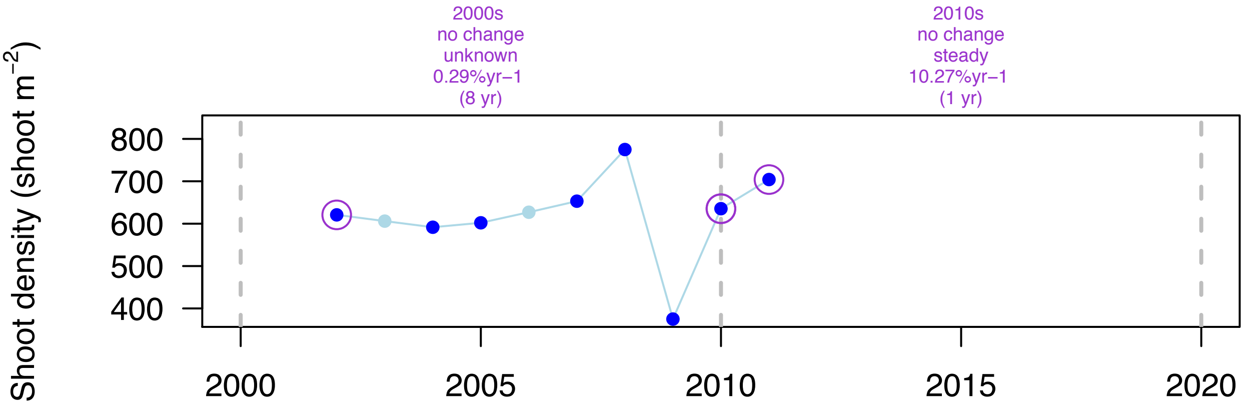

191_density

Duarte and Marbà (unpublished)

SITE: Can Picafort (Spain – Mediterranean) – Po (–10 m)

OVERALL: Net = –197.33 shoot m–2; Rate = –4.32 % yr–1; Perc Final = 68 % > decrease

DECADAL: YES (9 yr)

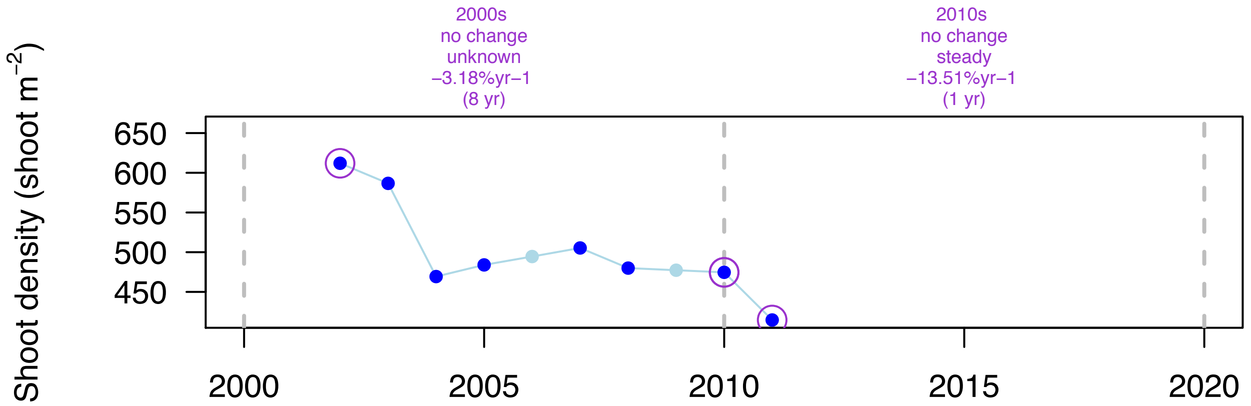

192_density

Duarte and Marbà (unpublished)

SITE: Es Cargol (Spain – Mediterranean) – Po (–6 m)

OVERALL: Net = 49.71 shoot m–2; Rate = 0.8 % yr–1; Perc Final = 105 % > no change

DECADAL: NO (6 yr)

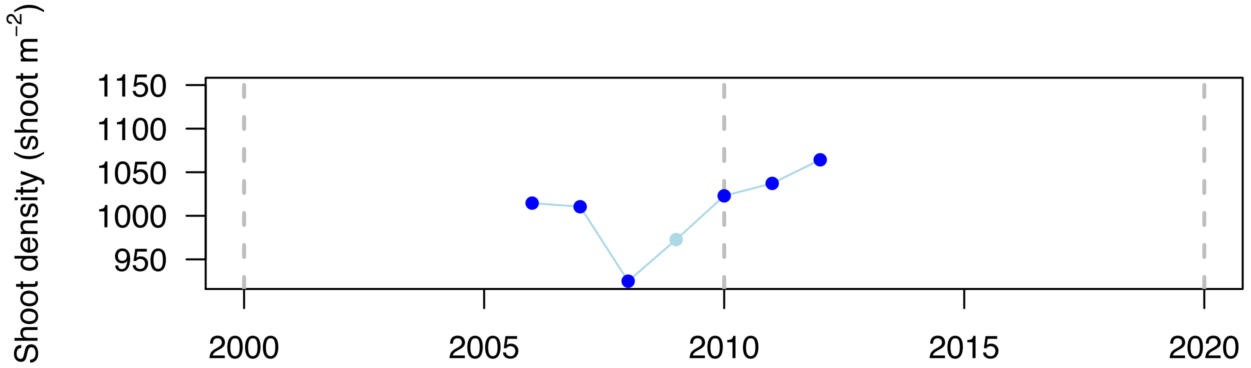

193_density

Duarte and Marbà (unpublished)

SITE: Fornells (Spain – Mediterranean) – Po (–6.8 m)

OVERALL: Net = –443.75 shoot m–2;   Rate = –8.04 % yr–1; Perc Final = 53 % > decrease

DECADAL: YES (8 yr)

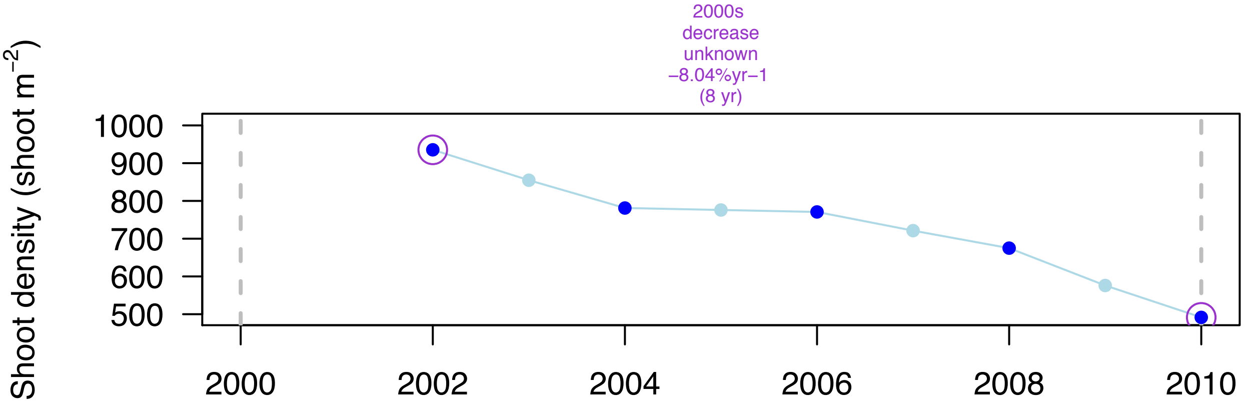

194_density

Duarte and Marbà (unpublished)

SITE: Cala d’Or (Spain – Mediterranean) – Po (–7 m)

OVERALL: Net = 15.34 shoot m–2;   Rate = 0.54 % yr–1; Perc Final = 104 % > no change

DECADAL: YES (8 yr)

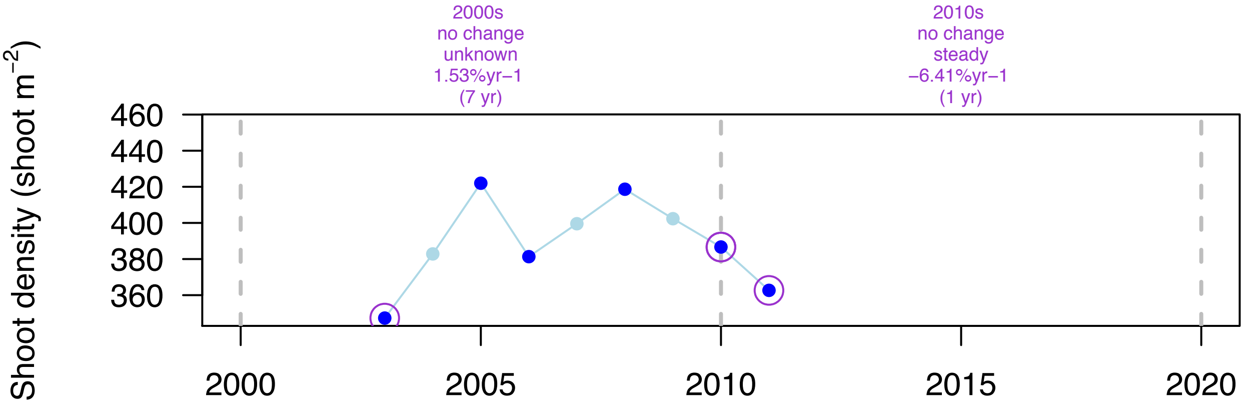

195_density

Duarte and Marbà (unpublished)

SITE: Cala Torreta (Spain – Mediterranean) – Po (–7.6 m)

OVERALL: Net = –180.24 shoot m–2;   Rate = –3.81 % yr–1; Perc Final = 68 % > decrease

DECADAL: YES (10 yr)

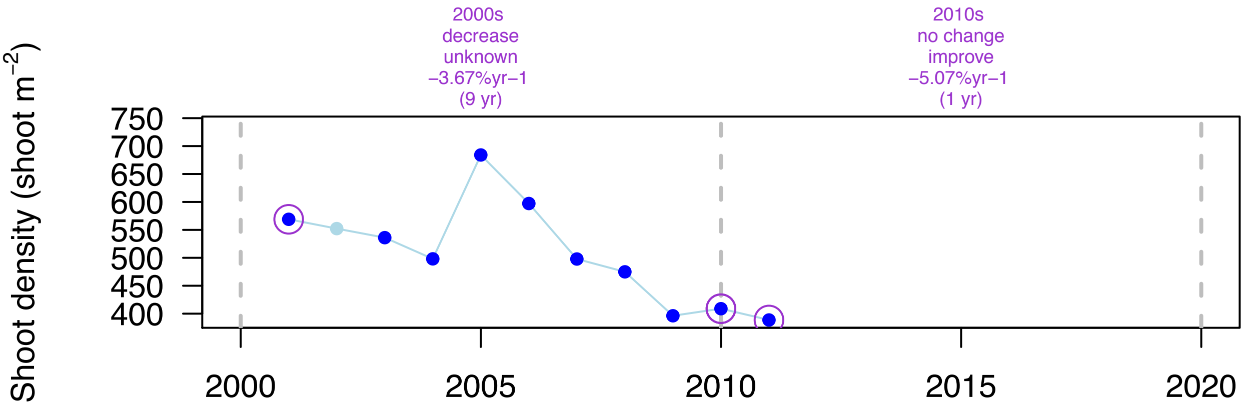

196_density

Duarte and Marbà (unpublished)

SITE: Illetes (Spain – Mediterranean) – Po (–5 m)

OVERALL: Net = 45.83 shoot m–2;   Rate = 0.66 % yr–1; Perc Final = 107 % > no change

DECADAL: YES (10 yr)

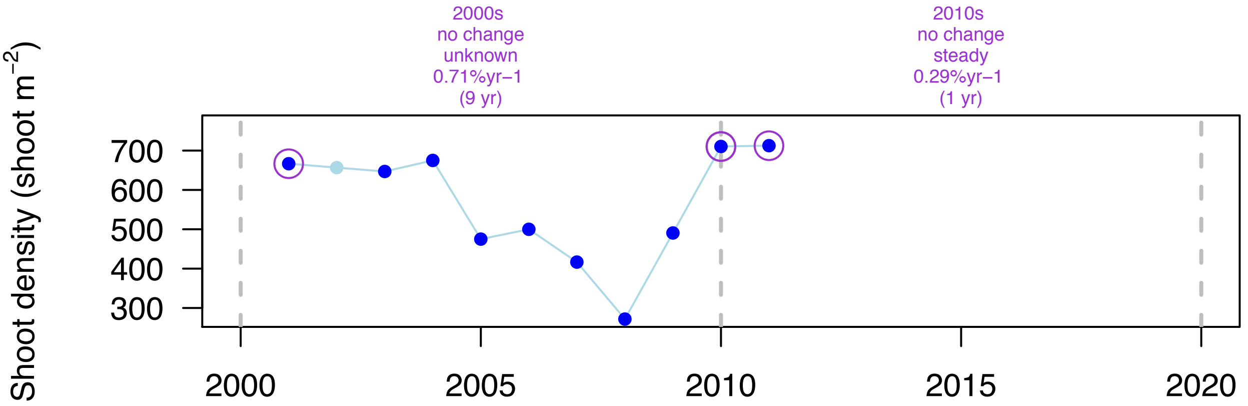

197_density

Duarte and Marbà (unpublished)

SITE: Els Pujols (Spain – Mediterranean) – Po (–4 m)

OVERALL: Net = 68.75 shoot m–2; Rate = 0.88 % yr–1; Perc Final = 109 % > no change

DECADAL: YES (10 yr)

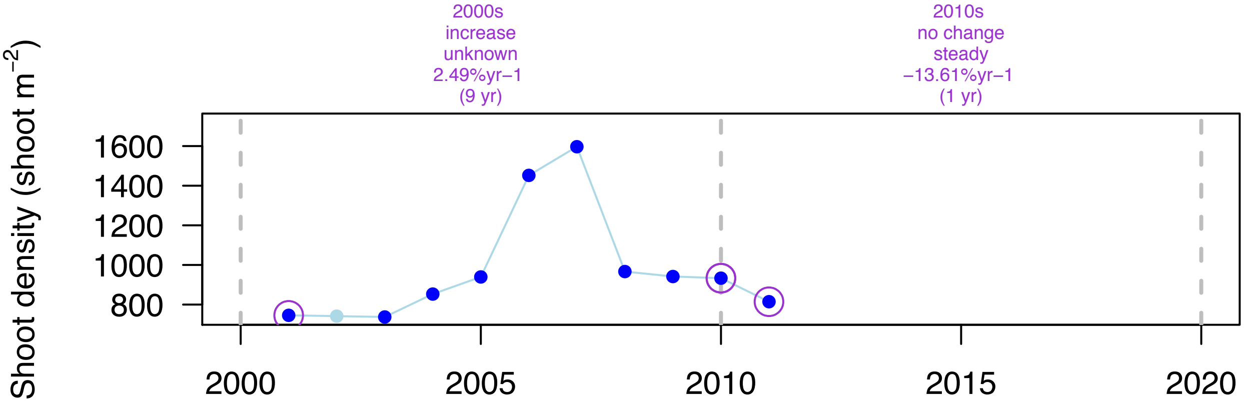

198_cover

González–Correa et al. 2015

SITE: Isla de Tabarca (Spain – Mediterranean) – Po (–4 m)

OVERALL: Net = –34 %; Rate = –2.65 % yr–1; Perc Final = 56 % > decrease

DECADAL: YES (22 yr)

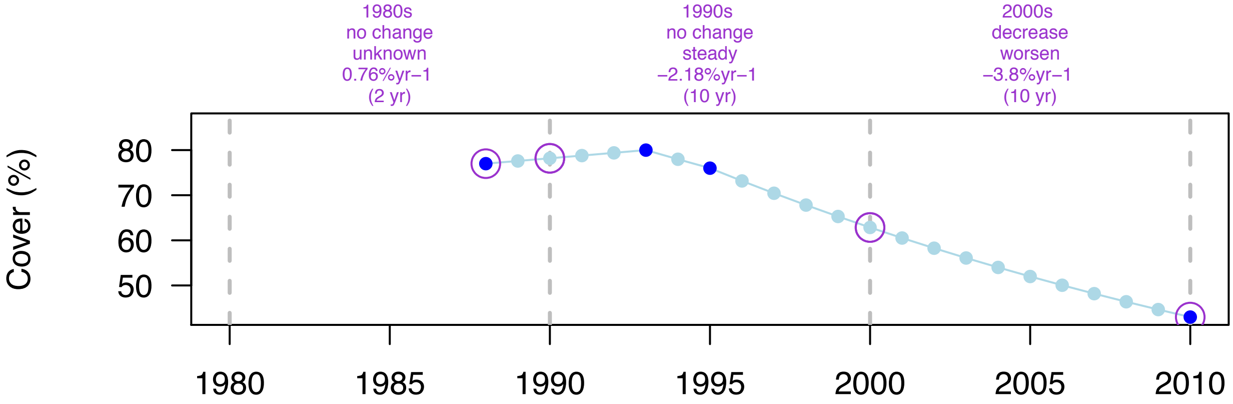

198_density

González–Correa et al. 2015

SITE: Isla de Tabarca (Spain – Mediterranean) – Po (–4 m)

OVERALL: Net = –238 shoot m–2;   Rate = –1.15 % yr–1; Perc Final = 78 % > no change

DECADAL: YES (22 yr)

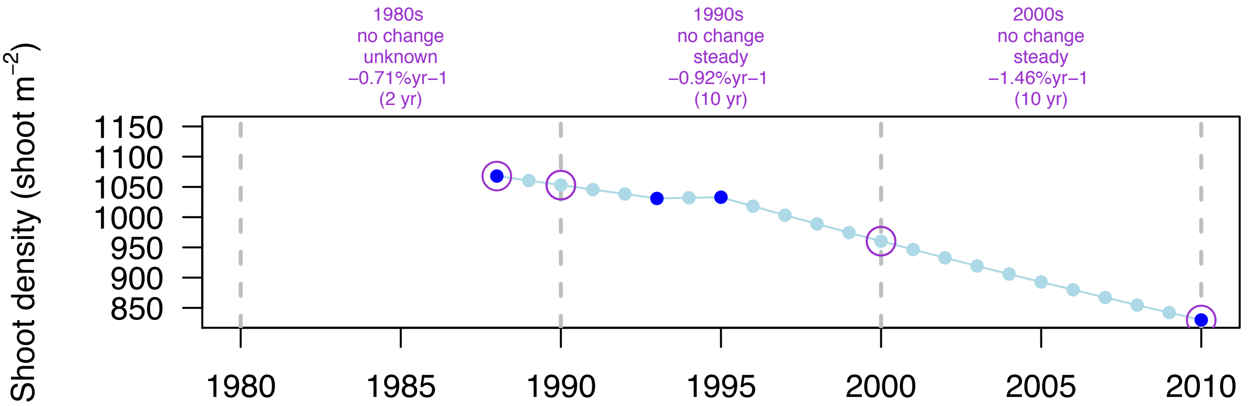

199_cover

González–Correa et al. 2015

SITE: Isla de Tabarca (Spain – Mediterranean) – Po (–12 m)

OVERALL: Net = 13 %;   Rate = 1.05 % yr–1; Perc Final = 126 % > increase

DECADAL: YES (22 yr)

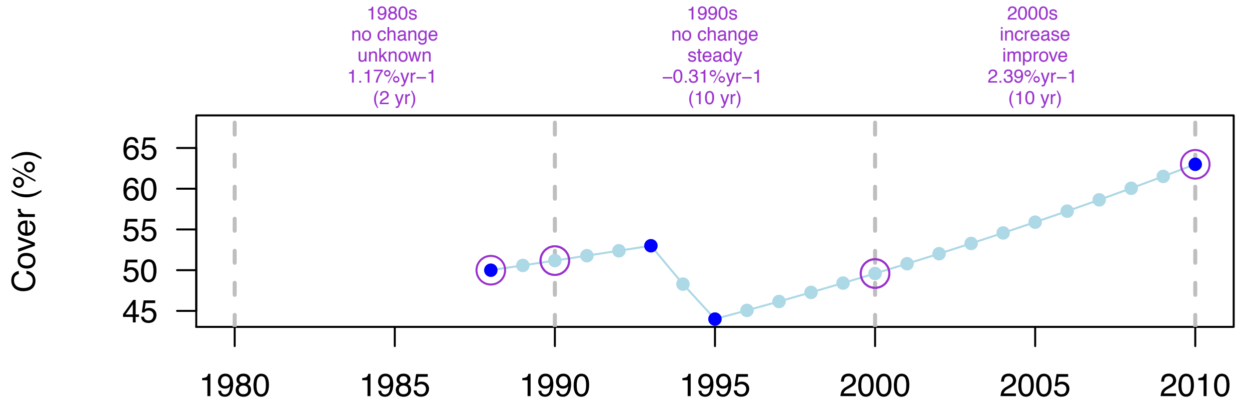

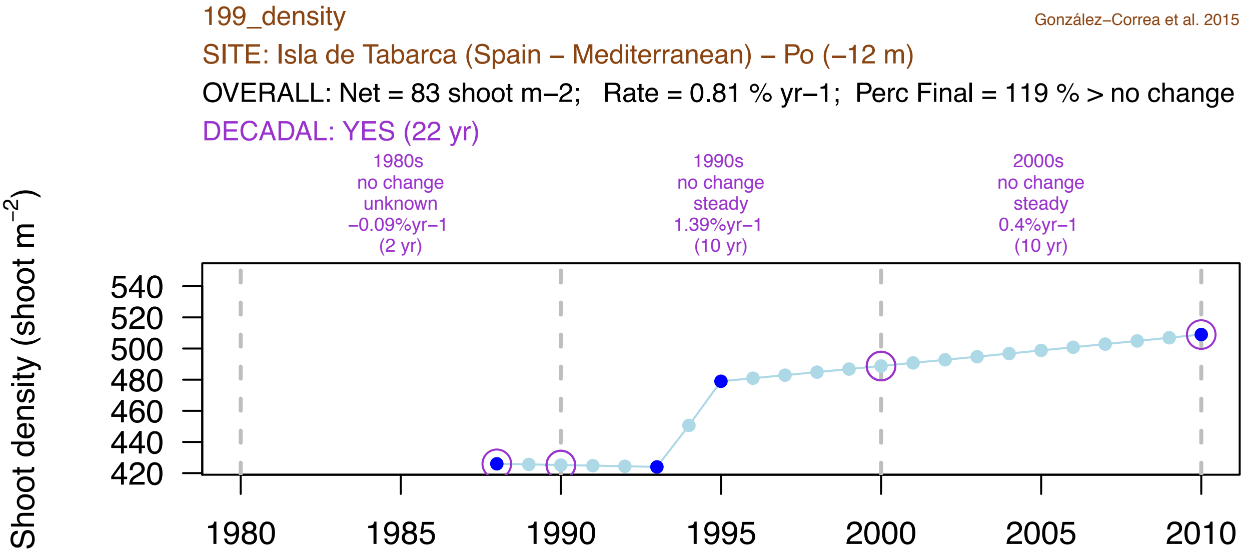
199_density
González−Correa et al. 2015
SITE: Isla de Tabarca (Spain − Mediterranean) − Po (−12 m)
OVERALL: Net = 83 shoot m−2;   Rate = 0.81 % yr−1; Perc Final = 119 % > no change
DECADAL: YES (22 yr)
1980s
no change
unknown
−0.09%yr−1
(2 yr)
1990s
no change
steady
1.39%yr−1
(10 yr)
2000s
no change
steady
0.4%yr−1
(10 yr)
Shoot density (shoot m−2)
540
520
500
480
460
440
420
1980
1985
1990
1995
2000
2005
2010

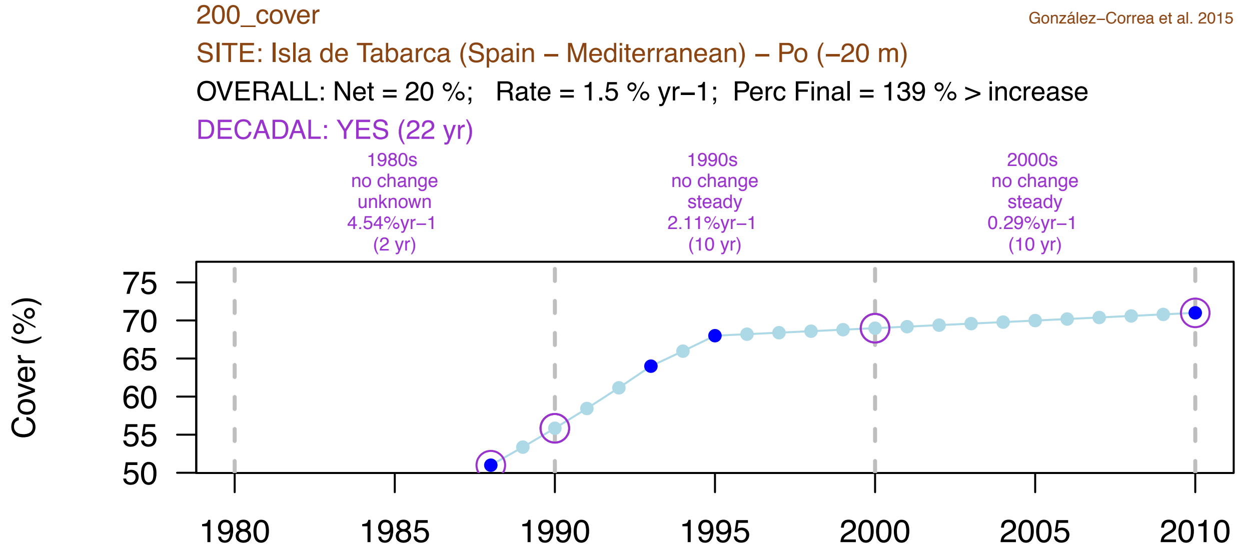
200_cover
González−Correa et al. 2015
SITE: Isla de Tabarca (Spain − Mediterranean) − Po (−20 m)
OVERALL: Net = 20 %;   Rate = 1.5 % yr−1; Perc Final = 139 % > increase
DECADAL: YES (22 yr)
1980s
no change
unknown
4.54%yr−1
(2 yr)
1990s
no change
steady
2.11%yr−1
(10 yr)
2000s
no change
steady
0.29%yr−1
(10 yr)
Cover (%)
75
70
65
60
55
50
1980
1985
1990
1995
2000
2005
2010

200_density

González–Correa et al. 2015

SITE: Isla de Tabarca (Spain – Mediterranean) – Po (–20 m)

OVERALL: Net = –52 shoot m–2; Rate = –0.65 % yr–1; Perc Final = 87 % > no change

DECADAL: YES (22 yr)

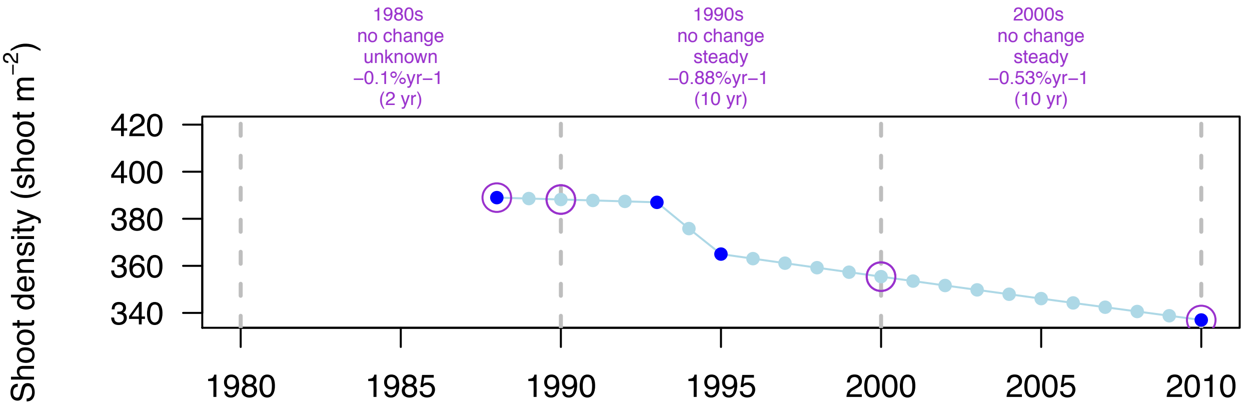

201_cover

Guillén et al. 2013

SITE: Altea (Spain – Mediterranean) – Po (–15 m)

OVERALL: Net = 23.81 %; Rate = 3.81 % yr–1; Perc Final = 141 % > increase

DECADAL: YES (9 yr)

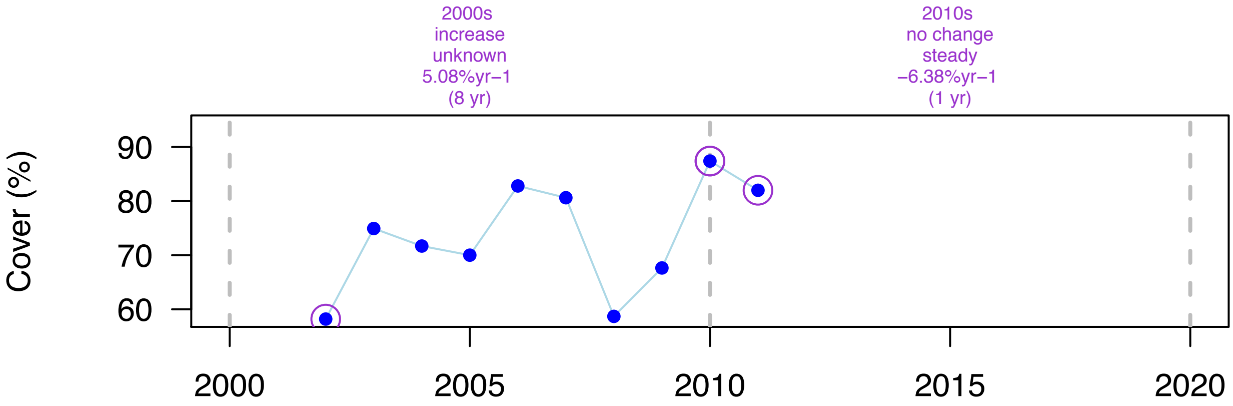

201_density

Guillén et al. 2013

SITE: Altea (Spain – Mediterranean) – Po (–15 m)

OVERALL: Net = 22.71 shoot m–2;   Rate = 1.6 % yr–1; Perc Final = 116 % > no change

DECADAL: YES (9 yr)

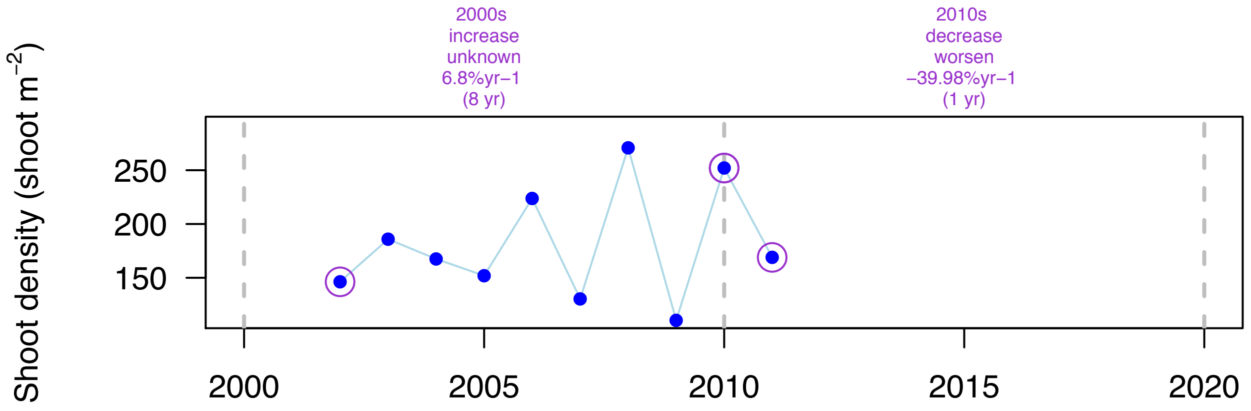

202_cover

Guillén et al. 2013

SITE: Altea (Spain – Mediterranean) – Po (–7 m)

OVERALL: Net = 5.8 %;   Rate = 0.81 % yr–1; Perc Final = 108 % > no change

DECADAL: YES (9 yr)

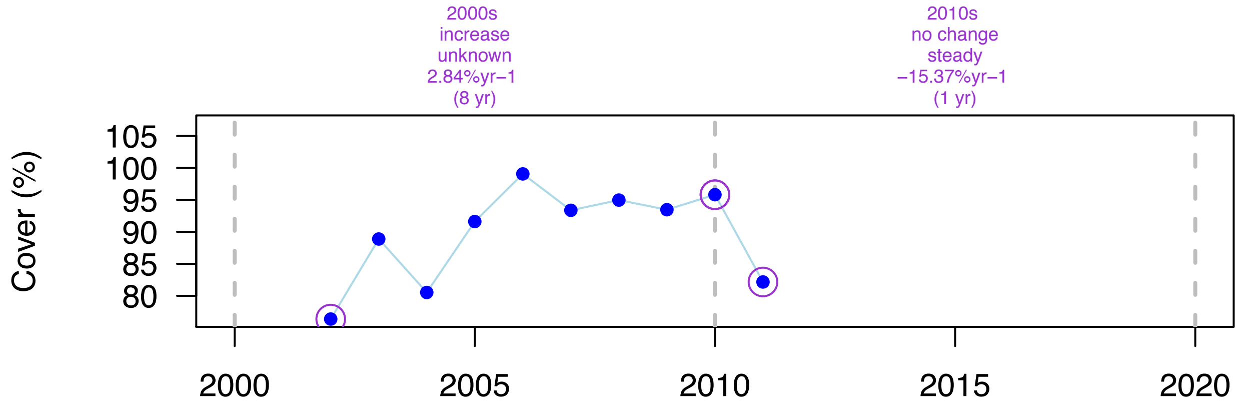

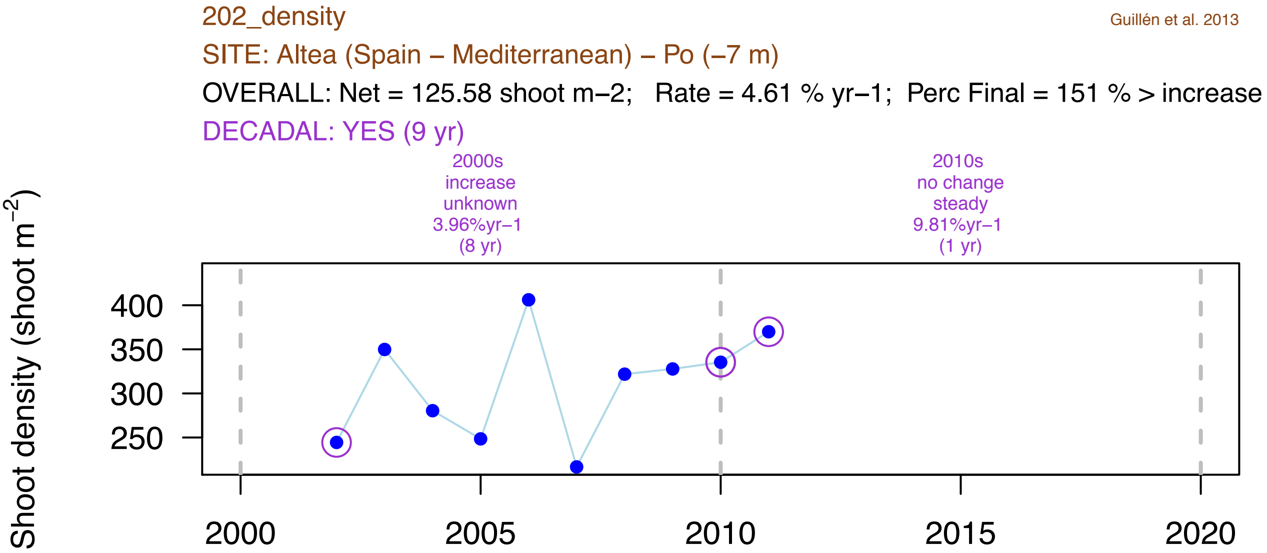
202_density
Guillén et al. 2013
SITE: Altea (Spain – Mediterranean) – Po (–7 m)
OVERALL: Net = 125.58 shoot m–2; Rate = 4.61 % yr–1; Perc Final = 151 % > increase
DECADAL: YES (9 yr)
2000s
increase
unknown
3.96%yr–1
(8 yr)
2010s
no change
steady
9.81%yr–1
(1 yr)
Shoot density (shoot m^{-2})
400
350
300
250
2000
2005
2010
2015
2020

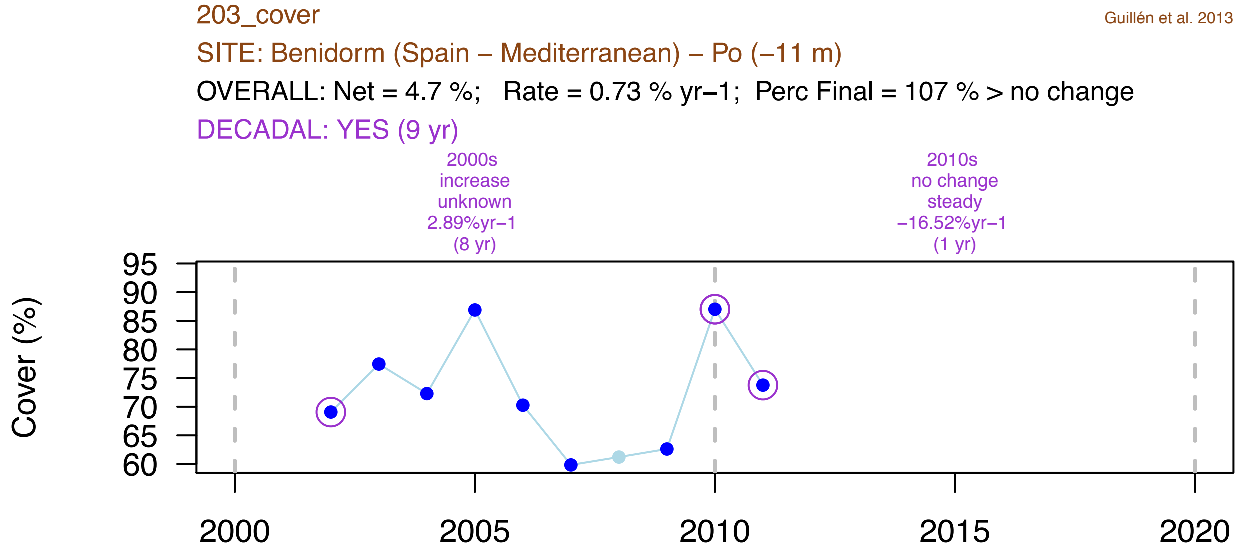
203_cover
Guillén et al. 2013
SITE: Benidorm (Spain – Mediterranean) – Po (–11 m)
OVERALL: Net = 4.7 %; Rate = 0.73 % yr–1; Perc Final = 107 % > no change
DECADAL: YES (9 yr)
2000s
increase
unknown
2.89%yr–1
(8 yr)
2010s
no change
steady
–16.52%yr–1
(1 yr)
Cover (%)
95
90
85
80
75
70
65
60
2000
2005
2010
2015
2020

203_density Guillén et al. 2013

SITE: Benidorm (Spain – Mediterranean) – Po (–11 m)

OVERALL: Net = 269.83 shoot m–2; Rate = 9.3 % yr–1; Perc Final = 231 % > increase

DECADAL: YES (9 yr)

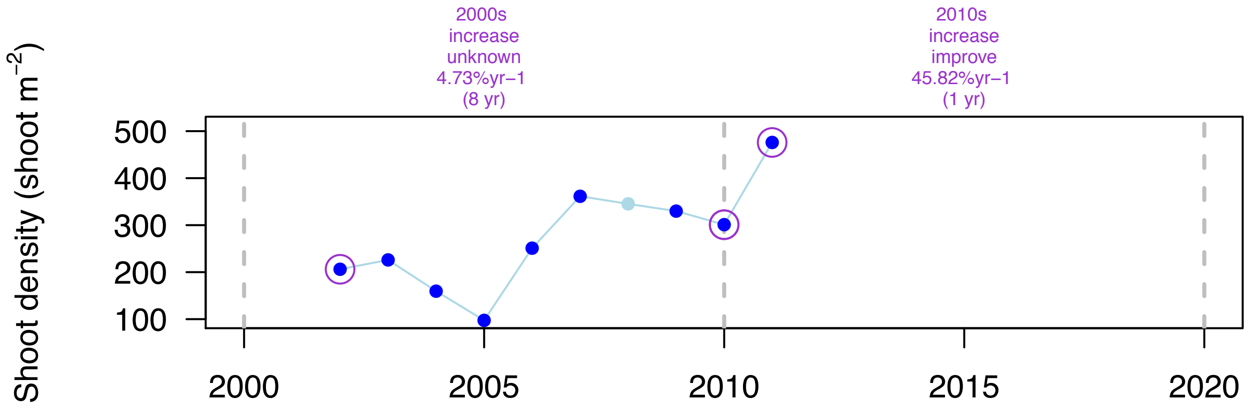

204_cover Guillén et al. 2013

SITE: Benidorm (Spain – Mediterranean) – Po (–5 m)

OVERALL: Net = 11.04 %; Rate = 1.63 % yr–1; Perc Final = 116 % > no change

DECADAL: YES (9 yr)

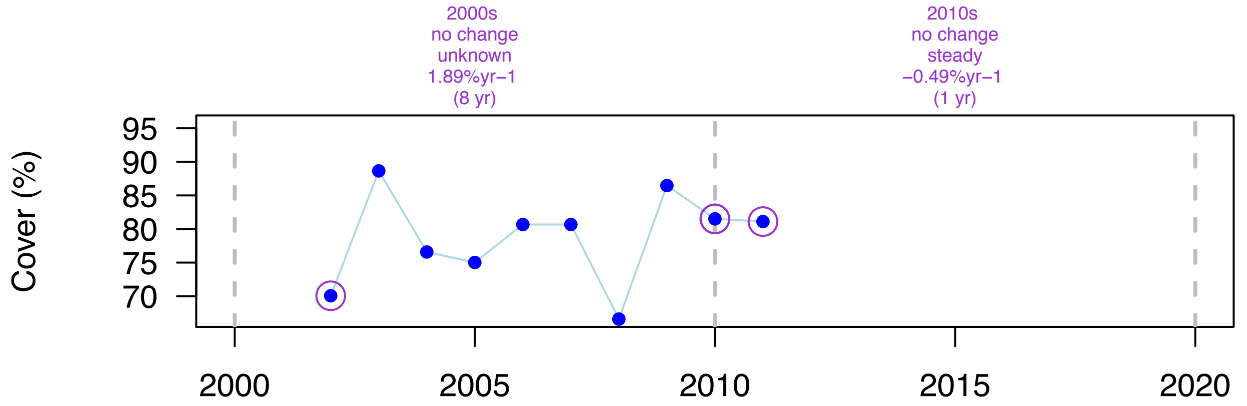

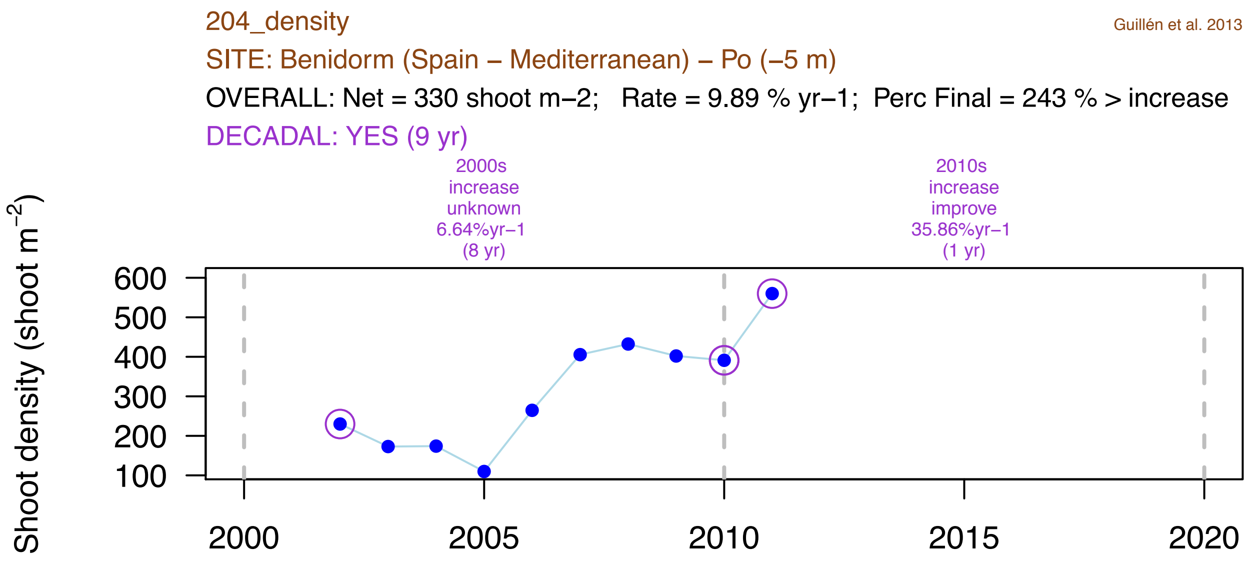
204_density
Guillén et al. 2013
SITE: Benidorm (Spain – Mediterranean) – Po (−5 m)
OVERALL: Net = 330 shoot m−2; Rate = 9.89 % yr−1; Perc Final = 243 % > increase
DECADAL: YES (9 yr)
2000s
increase
unknown
6.64%yr−1
(8 yr)
2010s
increase
improve
35.86%yr−1
(1 yr)
Shoot density (shoot m−2)
600
500
400
300
200
100
2000
2005
2010
2015
2020

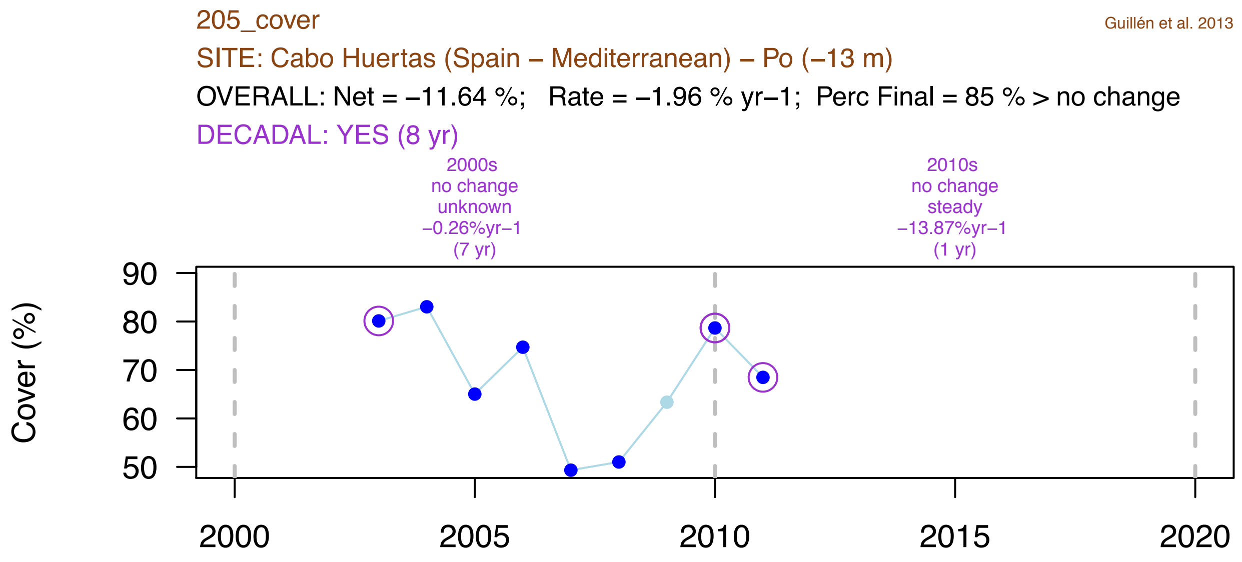
205_cover
Guillén et al. 2013
SITE: Cabo Huertas (Spain – Mediterranean) – Po (−13 m)
OVERALL: Net = −11.64 %; Rate = −1.96 % yr−1; Perc Final = 85 % > no change
DECADAL: YES (8 yr)
2000s
no change
unknown
−0.26%yr−1
(7 yr)
2010s
no change
steady
−13.87%yr−1
(1 yr)
Cover (%)
90
80
70
60
50
2000
2005
2010
2015
2020

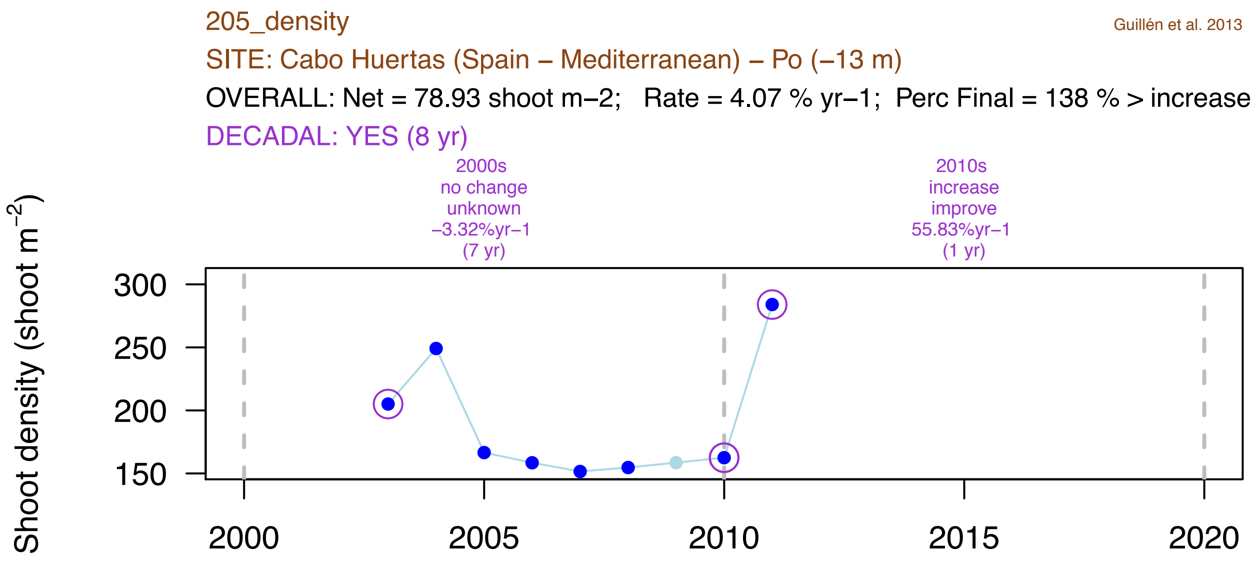
205_density
Guillén et al. 2013
SITE: Cabo Huertas (Spain – Mediterranean) – Po (–13 m)
OVERALL: Net = 78.93 shoot m–2; Rate = 4.07 % yr–1; Perc Final = 138 % > increase
DECADAL: YES (8 yr)
2000s
no change
unknown
–3.32%yr–1
(7 yr)
2010s
increase
improve
55.83%yr–1
(1 yr)
Shoot density (shoot m–2)
300
250
200
150
2000
2005
2010
2015
2020

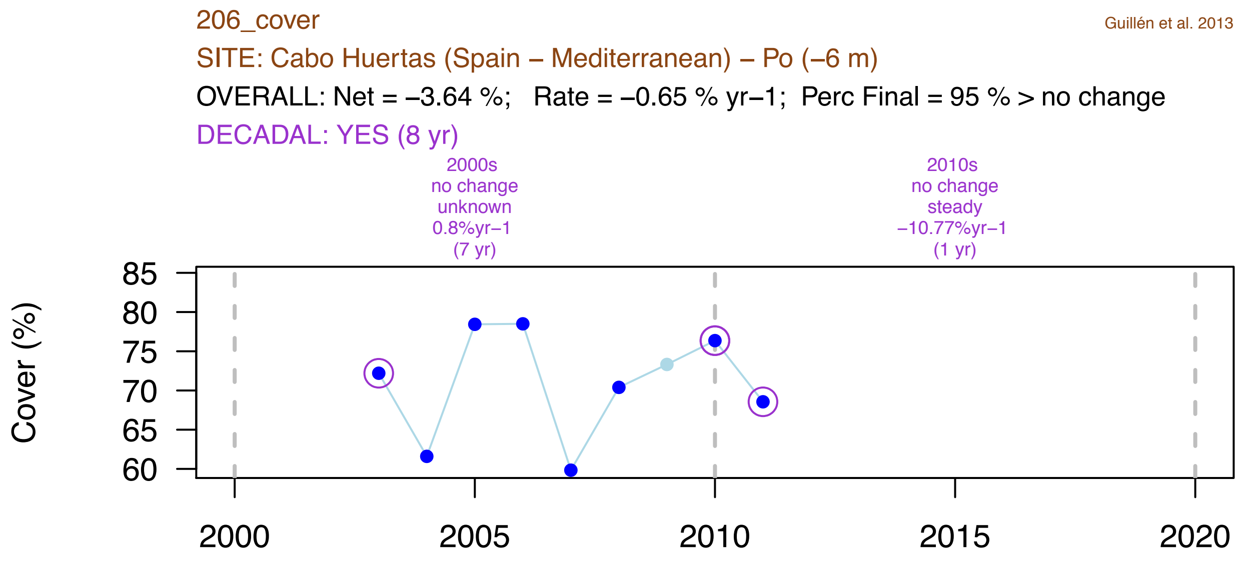
206_cover
Guillén et al. 2013
SITE: Cabo Huertas (Spain – Mediterranean) – Po (–6 m)
OVERALL: Net = –3.64 %; Rate = –0.65 % yr–1; Perc Final = 95 % > no change
DECADAL: YES (8 yr)
2000s
no change
unknown
0.8%yr–1
(7 yr)
2010s
no change
steady
–10.77%yr–1
(1 yr)
Cover (%)
85
80
75
70
65
60
2000
2005
2010
2015
2020

206_density
Guillén et al. 2013
SITE: Cabo Huertas (Spain – Mediterranean) – Po (–6 m)
OVERALL: Net = 316.43 shoot m–2;   Rate = 11.95 % yr–1; Perc Final = 260 % > increase
DECADAL: YES (8 yr)

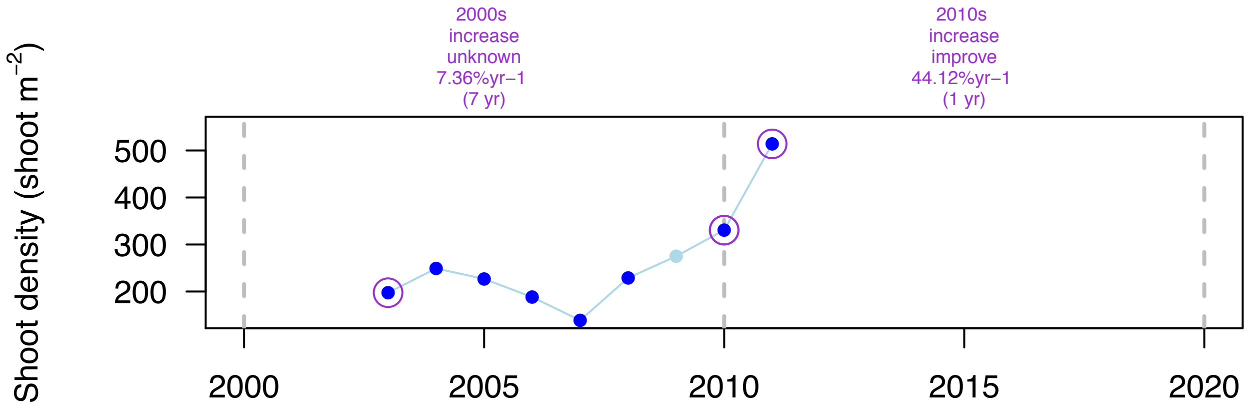

207_cover
Guillén et al. 2013
SITE: Cala Mina (Spain – Mediterranean) – Po (–10 m)
OVERALL: Net = 24.98 %;   Rate = 4.87 % yr–1; Perc Final = 141 % > increase
DECADAL: NO (7 yr)

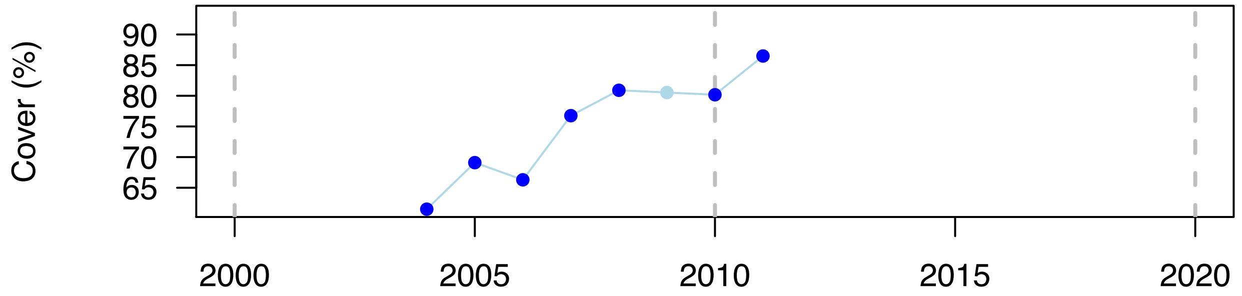

207_density

Guillén et al. 2013

SITE: Cala Mina (Spain – Mediterranean) – Po (–10 m)

OVERALL: Net = 47.29 shoot m–2; Rate = 2.98 % yr–1; Perc Final = 123 % > no change

DECADAL: NO (7 yr)

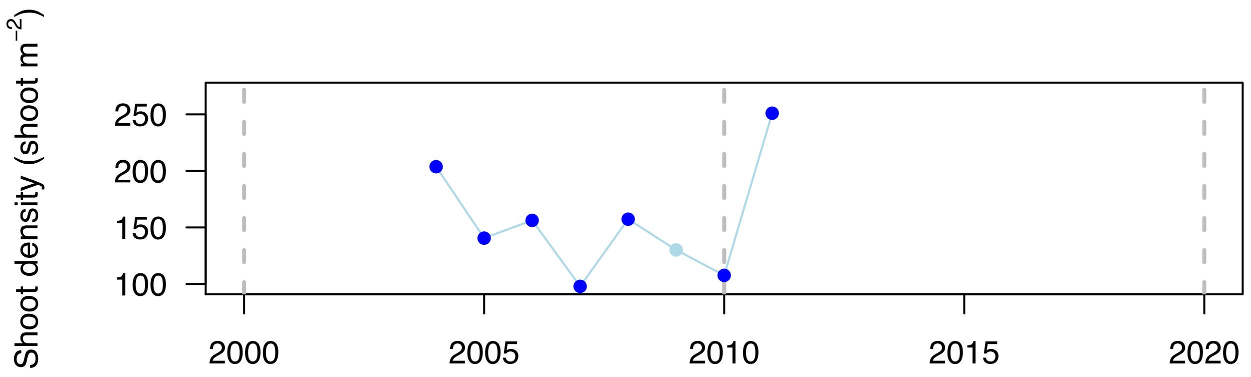

208_cover

Guillén et al. 2013

SITE: Cala Mina (Spain – Mediterranean) – Po (–5 m)

OVERALL: Net = –1.67 %; Rate = –0.35 % yr–1; Perc Final = 98 % > no change

DECADAL: NO (7 yr)

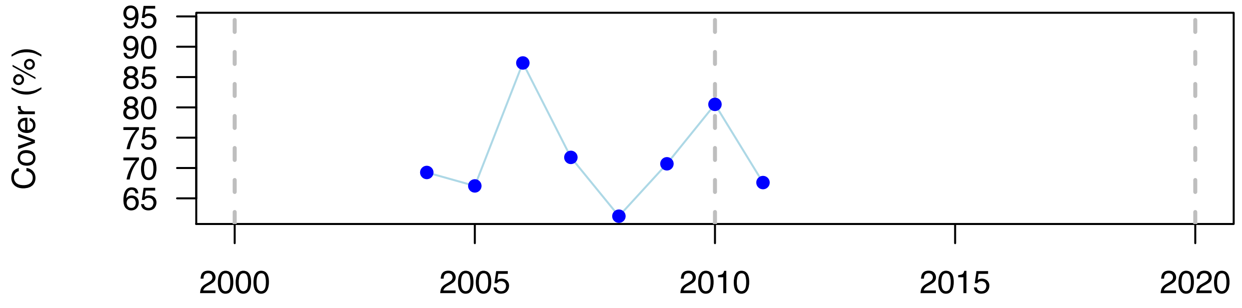

208_density Guillén et al. 2013

SITE: Cala Mina (Spain – Mediterranean) – Po (–5 m)

OVERALL: Net = 20 shoot m–2;   Rate = 1.22 % yr–1; Perc Final = 109 % > no change

DECADAL: NO (7 yr)

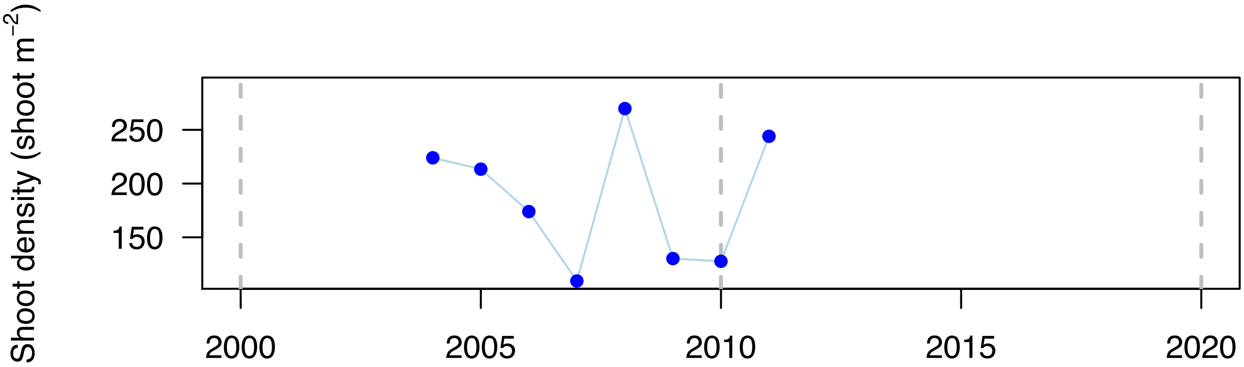

209_cover Guillén et al. 2013

SITE: Calpe (Spain – Mediterranean) – Po (–12 m)

OVERALL: Net = 17.65 %;   Rate = 2.42 % yr–1; Perc Final = 124 % > no change

DECADAL: YES (9 yr)

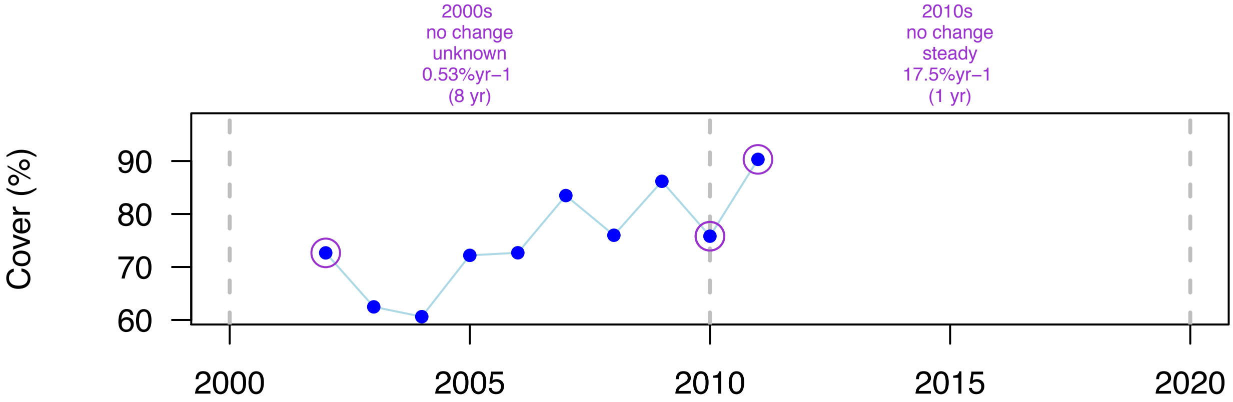

209_density

Guillén et al. 2013

SITE: Calpe (Spain – Mediterranean) – Po (–12 m)

OVERALL: Net = 75.5 shoot m–2;   Rate = 3.64 % yr–1; Perc Final = 139 % > increase

DECADAL: YES (9 yr)

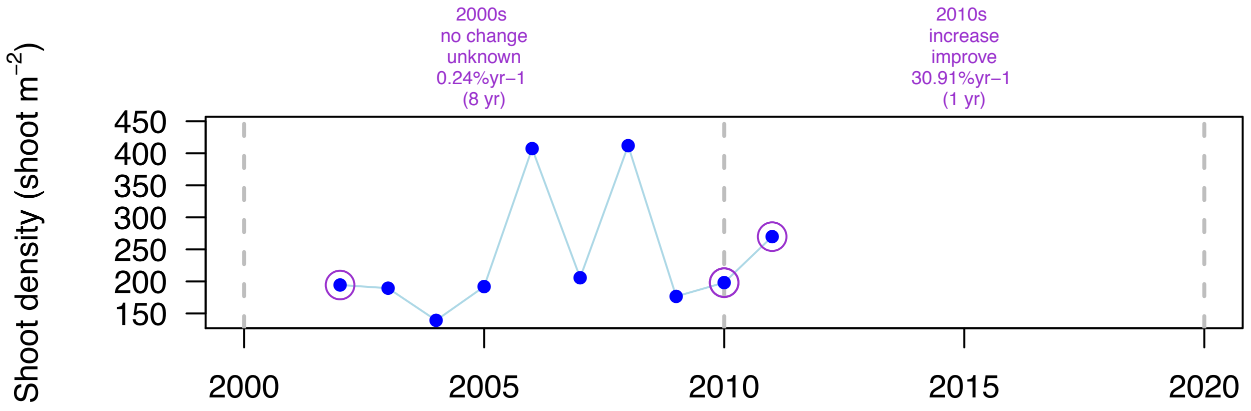

210_cover

Guillén et al. 2013

SITE: Calpe (Spain – Mediterranean) – Po (–6 m)

OVERALL: Net = 18.71 %;   Rate = 3.23 % yr–1; Perc Final = 134 % > increase

DECADAL: YES (9 yr)

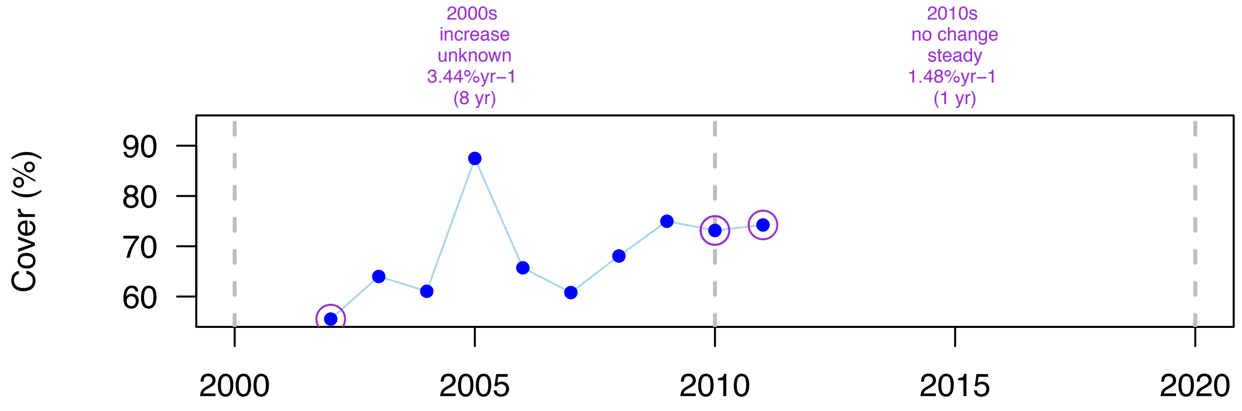

210_density

Guillén et al. 2013

SITE: Calpe (Spain – Mediterranean) – Po (–6 m)

OVERALL: Net = 205.4 shoot m–2;   Rate = 4.62 % yr–1; Perc Final = 152 % > increase

DECADAL: YES (9 yr)

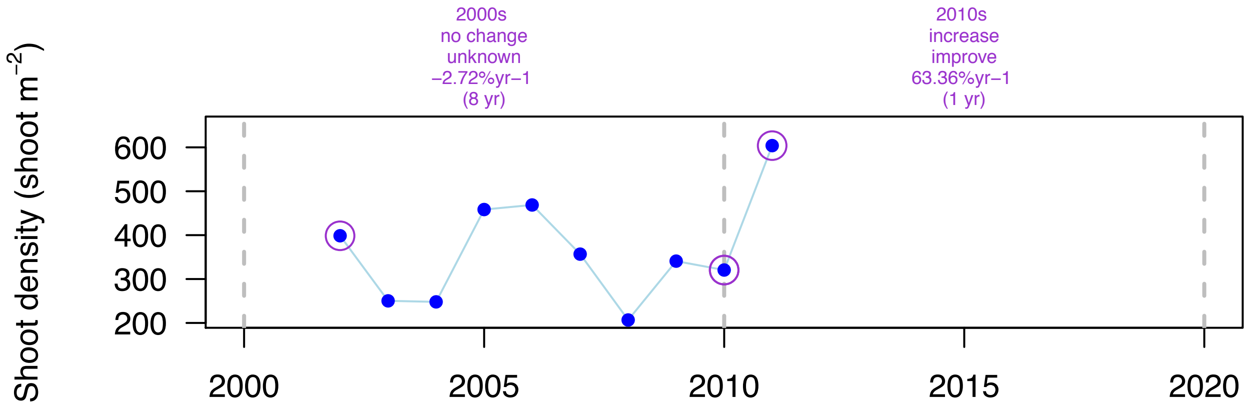

211_cover

Guillén et al. 2013

SITE: El Campello (Spain – Mediterranean) – Po (–13 m)

OVERALL: Net = –9.79 %;   Rate = –1.21 % yr–1; Perc Final = 90 % > no change

DECADAL: YES (9 yr)

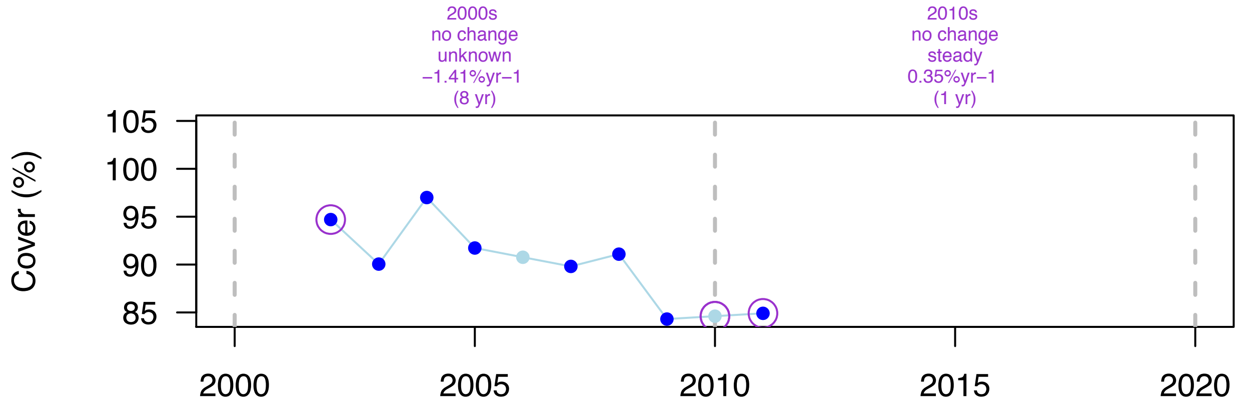

211_density

Guillén et al. 2013

SITE: El Campello (Spain – Mediterranean) – Po (–13 m)

OVERALL: Net = 98.9 shoot m–2;   Rate = 4.27 % yr–1; Perc Final = 147 % > increase

DECADAL: YES (9 yr)

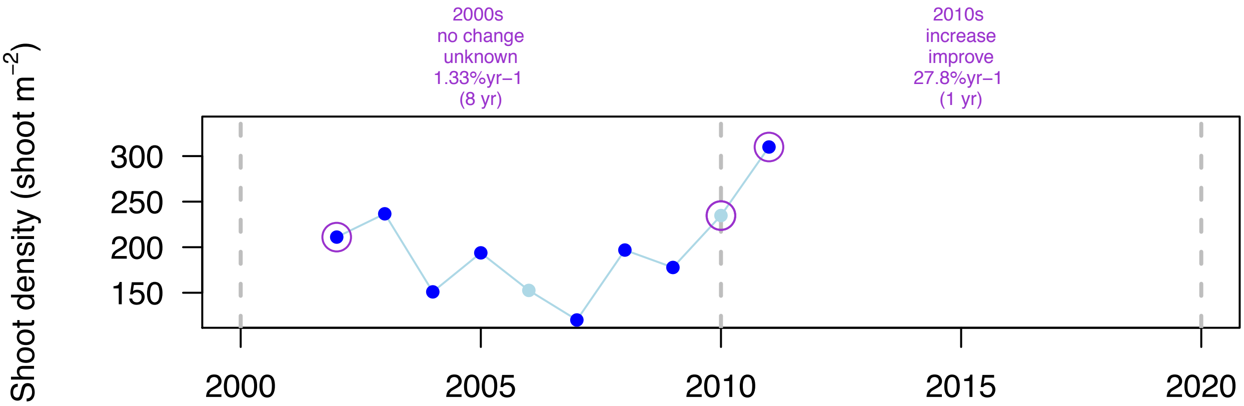

212_cover

Guillén et al. 2013

SITE: El Campello (Spain – Mediterranean) – Po (–7 m)

OVERALL: Net = 7.35 %;   Rate = 1.19 % yr–1; Perc Final = 111 % > no change

DECADAL: YES (9 yr)

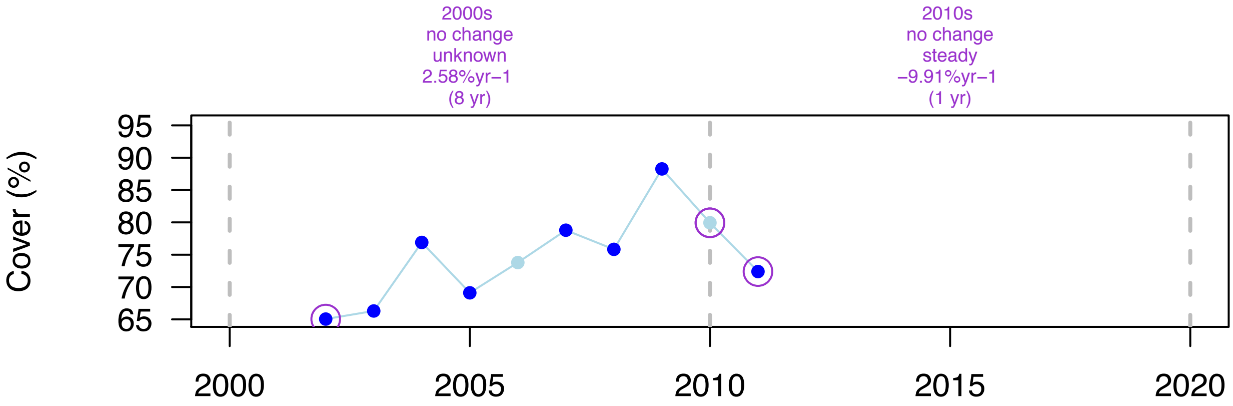

212_density

Guillén et al. 2013

SITE: El Campello (Spain – Mediterranean) – Po (–7 m)

OVERALL: Net = 134 shoot m–2;   Rate = 4.9 % yr–1; Perc Final = 155 % > increase

DECADAL: YES (9 yr)

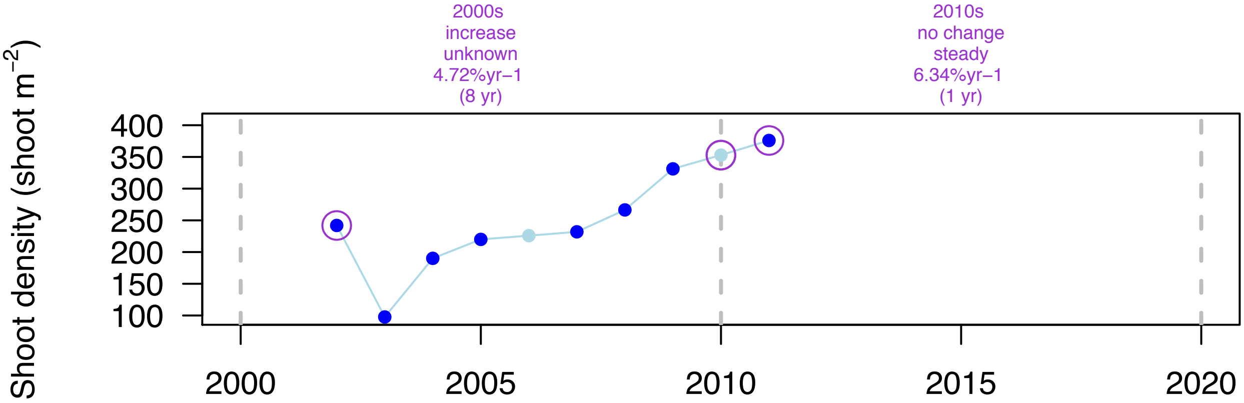

213_cover

Guillén et al. 2013

SITE: Carabassí (Spain – Mediterranean) – Po (–12 m)

OVERALL: Net = 32.33 %;   Rate = 4.65 % yr–1; Perc Final = 152 % > increase

DECADAL: YES (9 yr)

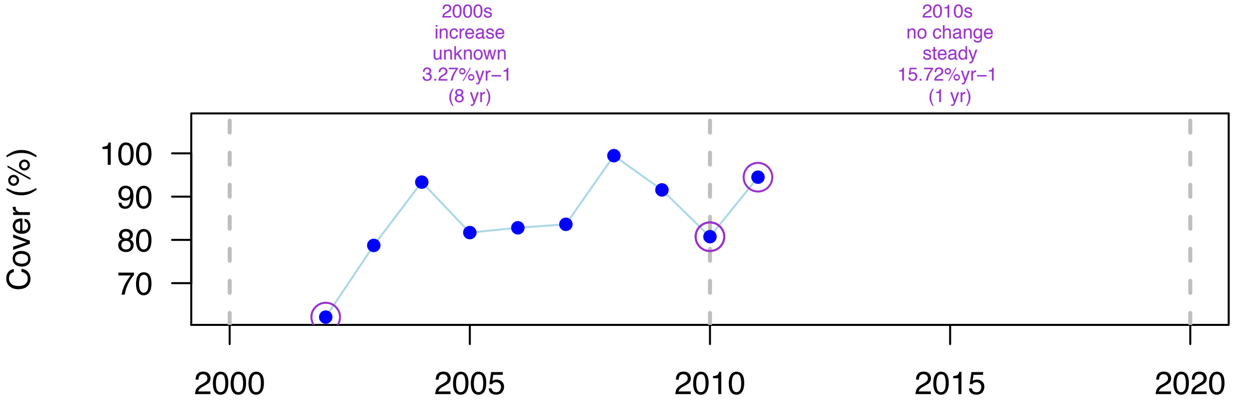

213_density

Guillén et al. 2013

SITE: Carabassí (Spain – Mediterranean) – Po (–12 m)

OVERALL: Net = 106.42 shoot m–2;   Rate = 6.62 % yr–1; Perc Final = 181 % > increase

DECADAL: YES (9 yr)

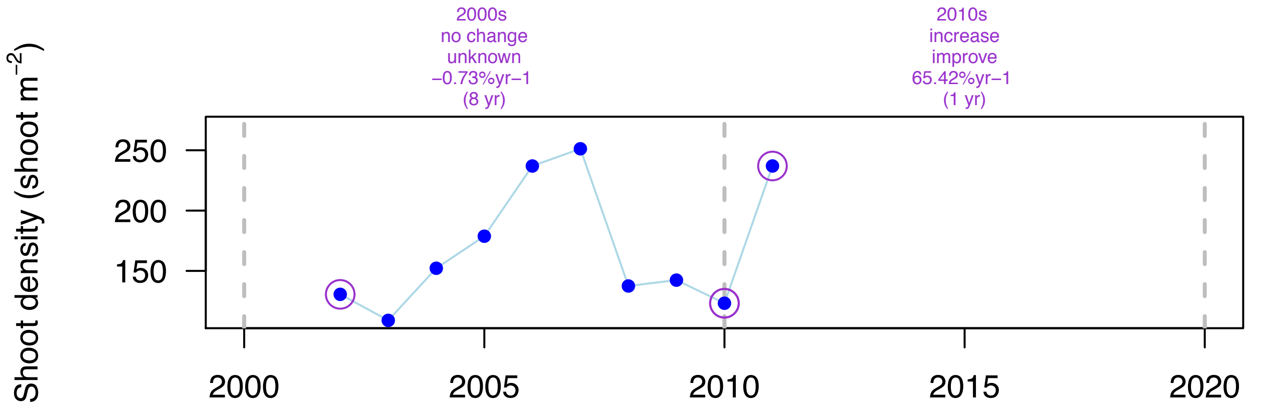

214_cover

Guillén et al. 2013

SITE: Carabassí (Spain – Mediterranean) – Po (–6 m)

OVERALL: Net = 0.44 %;   Rate = 0.07 % yr–1; Perc Final = 101 % > no change

DECADAL: YES (9 yr)

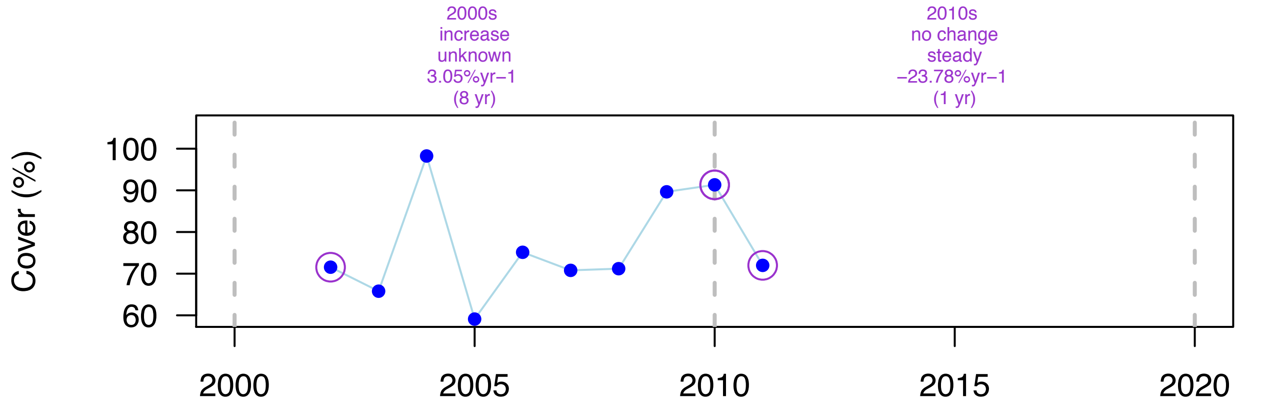

214_density

Guillén et al. 2013

SITE: Carabassí (Spain – Mediterranean) – Po (–6 m)

OVERALL: Net = 86 shoot m–2; Rate = 4.11 % yr–1; Perc Final = 145 % > increase

DECADAL: YES (9 yr)

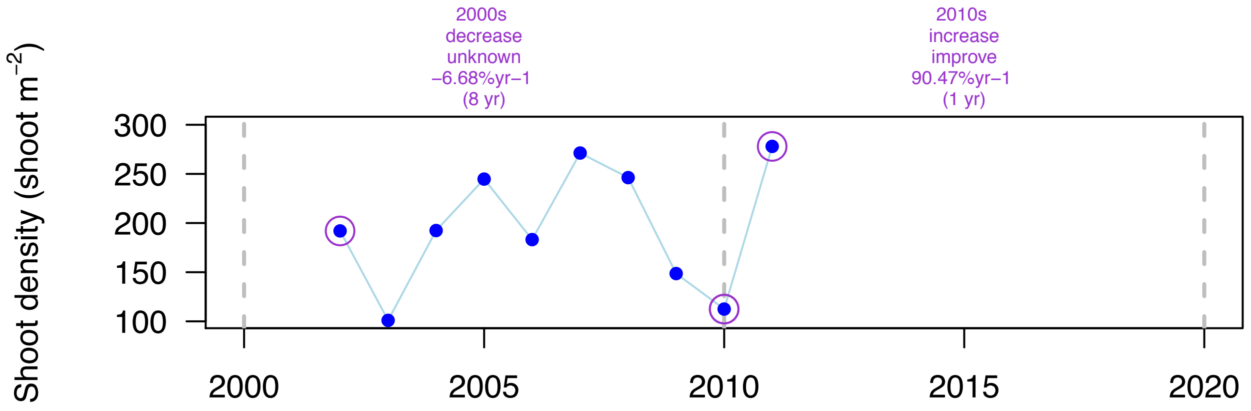

215_cover

Guillén et al. 2013

SITE: Denia (Spain – Mediterranean) – Po (–13 m)

OVERALL: Net = 15.9 %; Rate = 5.07 % yr–1; Perc Final = 143 % > increase

DECADAL: NO (7 yr)

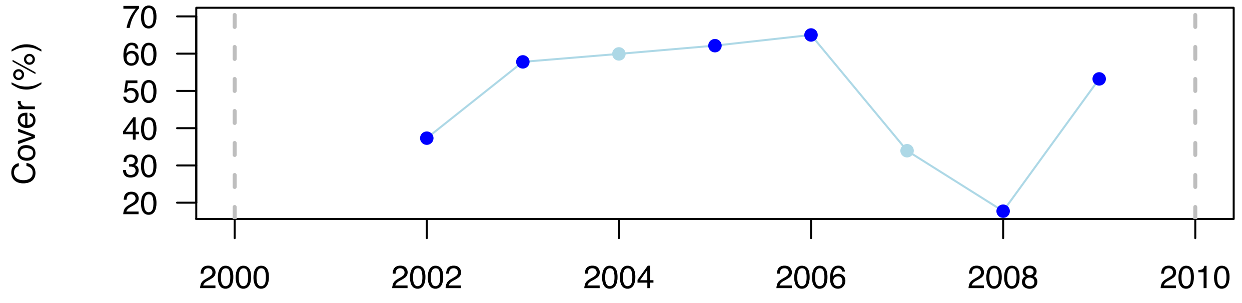

215_density Guillén et al. 2013

SITE: Denia (Spain – Mediterranean) – Po (–13 m)

OVERALL: Net = 33.46 shoot m–2;   Rate = 1.84 % yr–1; Perc Final = 114 % > no change

DECADAL: NO (7 yr)

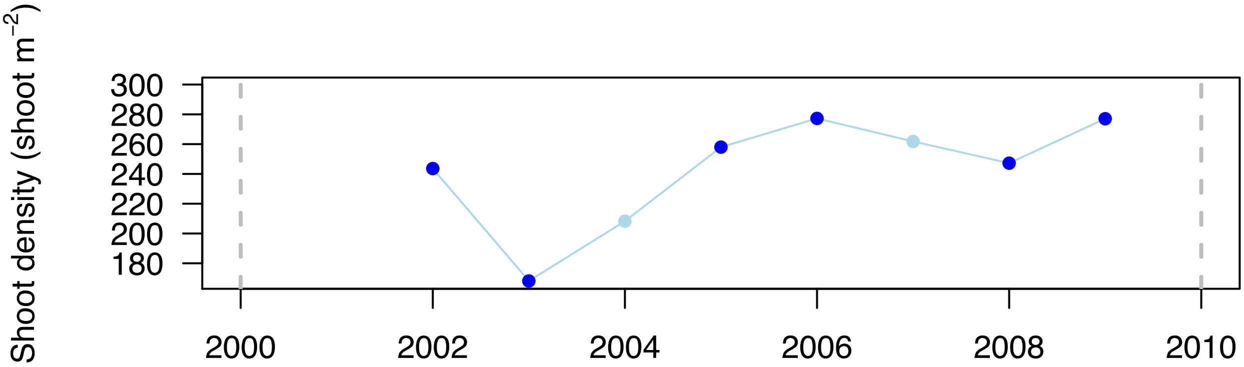

216_cover Guillén et al. 2013

SITE: Denia (Spain – Mediterranean) – Po (–5 m)

OVERALL: Net = 10.19 %;   Rate = 2.27 % yr–1; Perc Final = 117 % > no change

DECADAL: NO (7 yr)

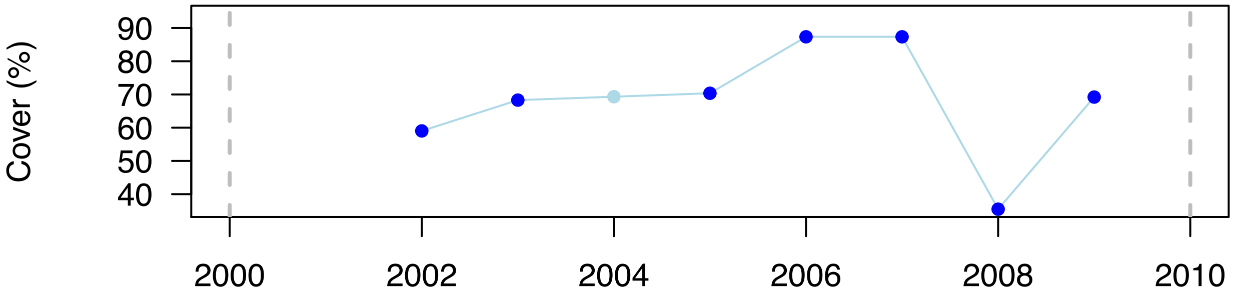

216_density

Guillén et al. 2013

SITE: Denia (Spain – Mediterranean) – Po (–5 m)

OVERALL: Net = 78.84 shoot m–2;   Rate = 4.01 % yr–1; Perc Final = 132 % > increase

DECADAL: NO (7 yr)

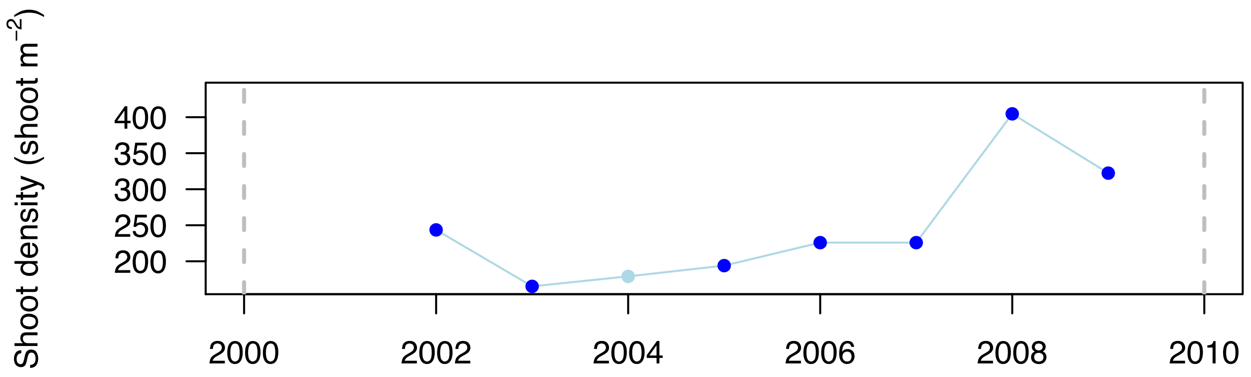

217_cover

Guillén et al. 2013

SITE: Moraira (Spain – Mediterranean) – Po (–14 m)

OVERALL: Net = 9.76 %;   Rate = 1.9 % yr–1; Perc Final = 112 % > no change

DECADAL: NO (6 yr)

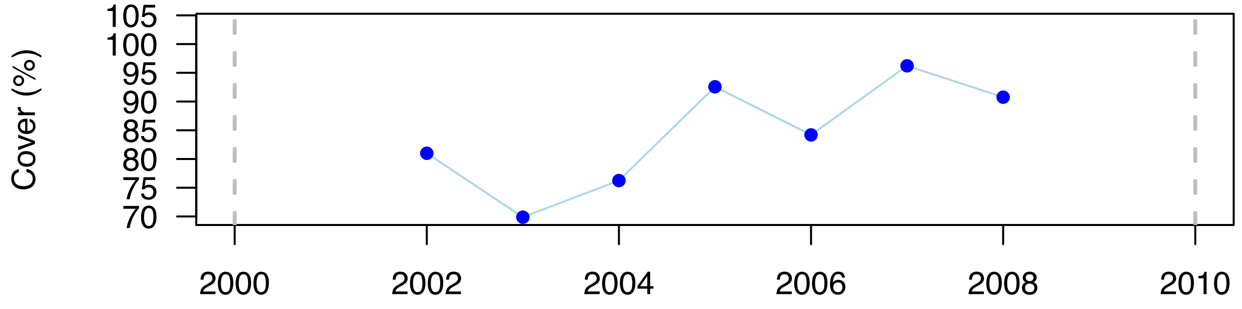

217_density
Guillén et al. 2013
SITE: Moraira (Spain – Mediterranean) – Po (–14 m)
OVERALL: Net = 4.3 shoot m–2;   Rate = 0.72 % yr–1; Perc Final = 104 % > no change
DECADAL: NO (6 yr)

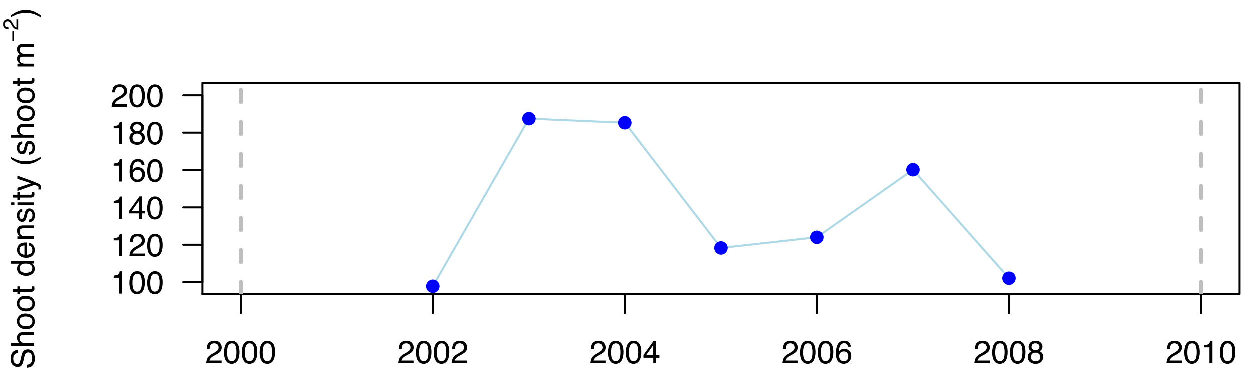

218_cover
Guillén et al. 2013
SITE: Moraira (Spain – Mediterranean) – Po (–7 m)
OVERALL: Net = 15.07 %;   Rate = 2.64 % yr–1; Perc Final = 120 % > no change
DECADAL: NO (7 yr)

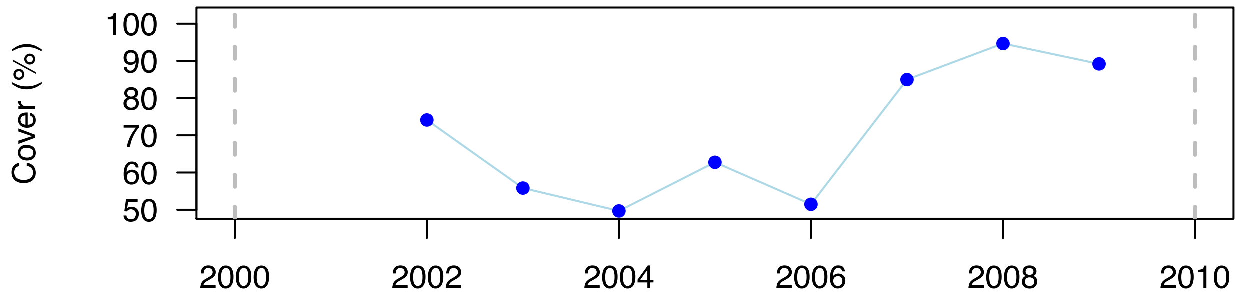

218_density

Guillén et al. 2013

SITE: Moraira (Spain – Mediterranean) – Po (–7 m)

OVERALL: Net = 33.1 shoot m–2;   Rate = 1.84 % yr–1; Perc Final = 114 % > no change

DECADAL: NO (7 yr)

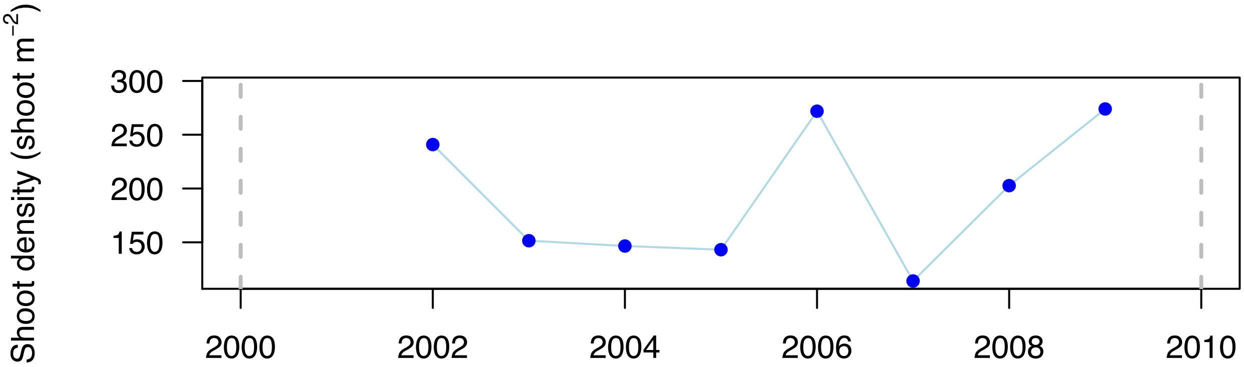

219_cover

Guillén et al. 2013

SITE: Racó Conill (Spain – Mediterranean) – Po (–10 m)

OVERALL: Net = 6.27 %;   Rate = 1.62 % yr–1; Perc Final = 112 % > no change

DECADAL: NO (7 yr)

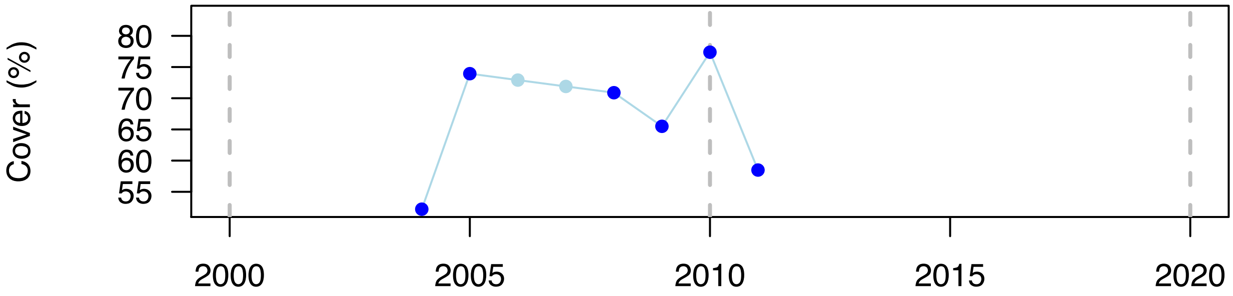

219_density Guillén et al. 2013

SITE: Racó Conill (Spain – Mediterranean) – Po (–10 m)

OVERALL: Net = –10.78 shoot m–2; Rate = –0.51 % yr–1; Perc Final = 96 % > no change

DECADAL: NO (7 yr)

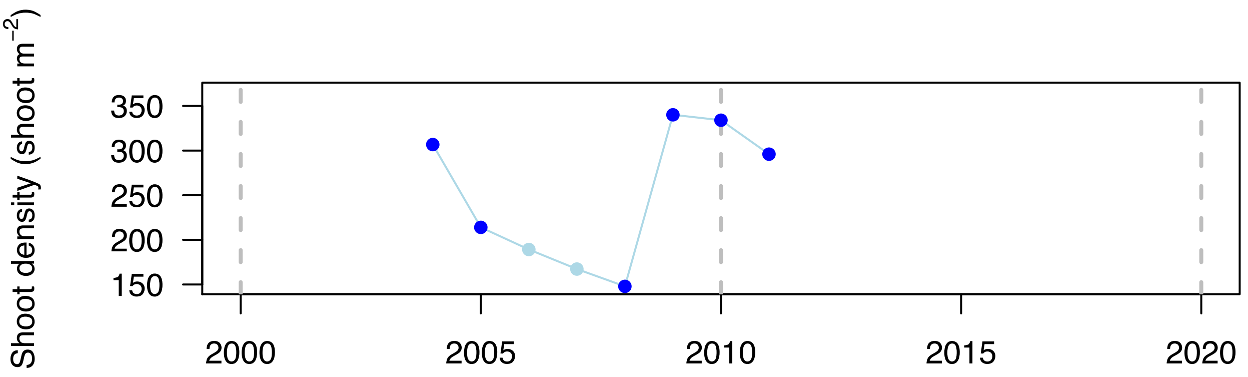

220_cover Guillén et al. 2013

SITE: Racó Conill (Spain – Mediterranean) – Po (–5 m)

OVERALL: Net = 3.62 %; Rate = 0.69 % yr–1; Perc Final = 105 % > no change

DECADAL: NO (7 yr)

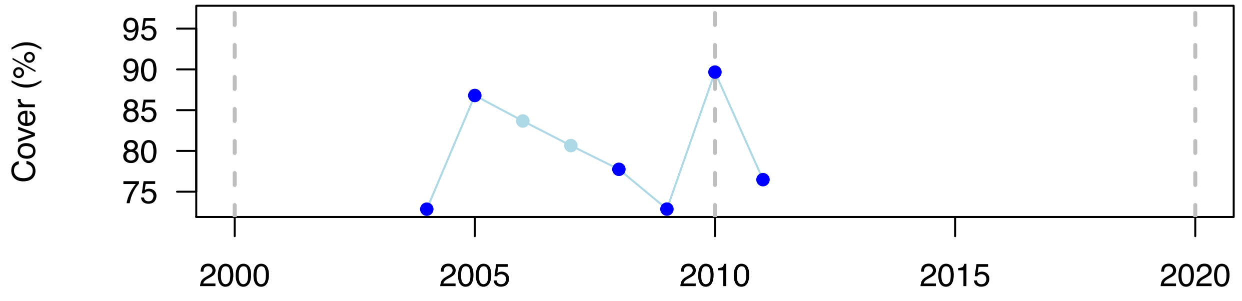

220_density Guillén et al. 2013

SITE: Racó Conill (Spain – Mediterranean) – Po (–5 m)

OVERALL: Net = 101 shoot m–2; Rate = 4.58 % yr–1; Perc Final = 138 % > increase

DECADAL: NO (7 yr)

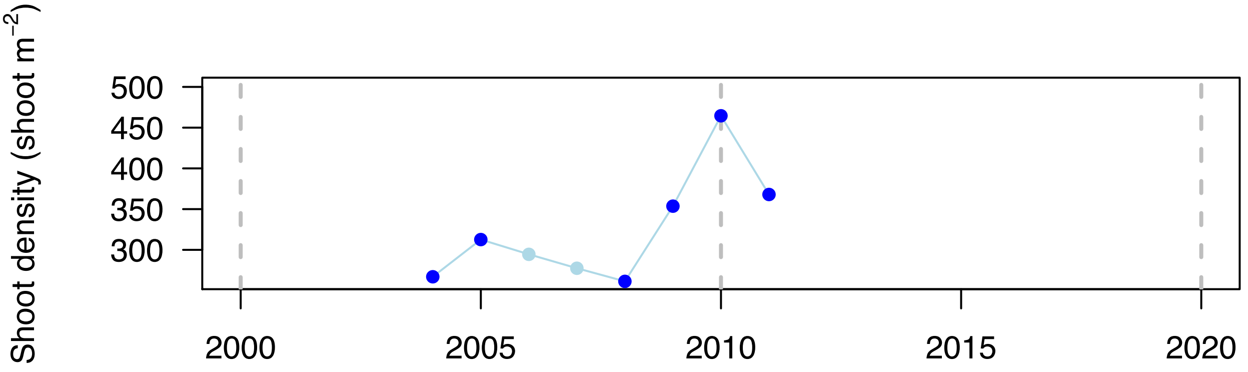

221_cover Guillén et al. 2013

SITE: Tabarca Escull Negre (Spain – Mediterranean) – Po (–12 m)

OVERALL: Net = –7 %; Rate = –0.97 % yr–1; Perc Final = 92 % > no change

DECADAL: YES (9 yr)

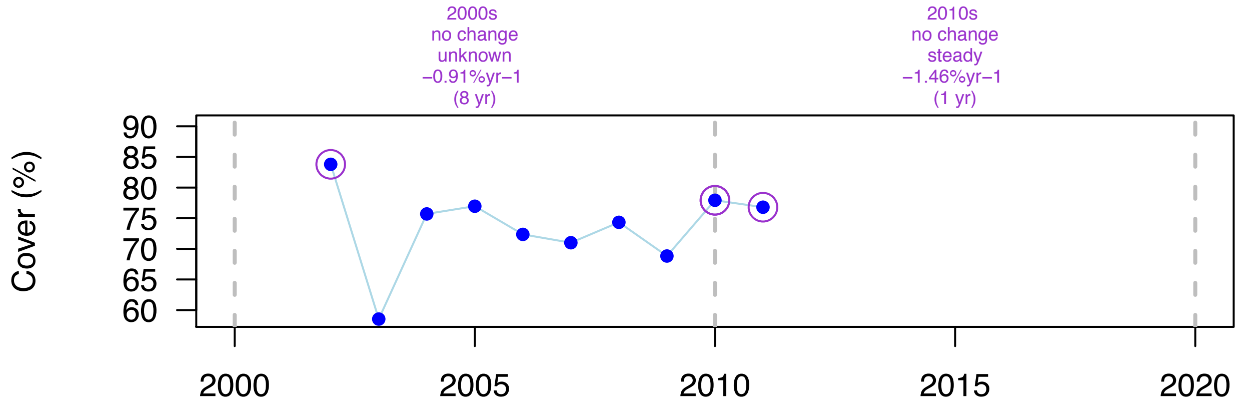

221_density

Guillén et al. 2013

SITE: Tabarca Escull Negre (Spain – Mediterranean) – Po (–12 m)

OVERALL: Net = 168 shoot m–2;   Rate = 5.84 % yr–1; Perc Final = 169 % > increase

DECADAL: YES (9 yr)

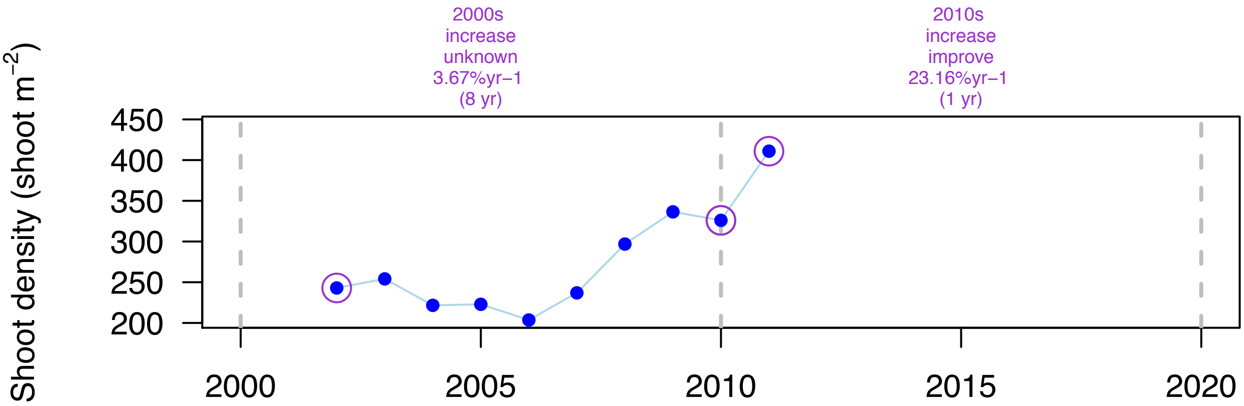

222_area

Plus et al. 2010

SITE: Arguin Bank (France – Atlantic) – Zn (0 m)

OVERALL: Net = 0 ha;   Rate = 0 % yr–1; Perc Final = 100 % > no change

DECADAL: NO (2 yr)

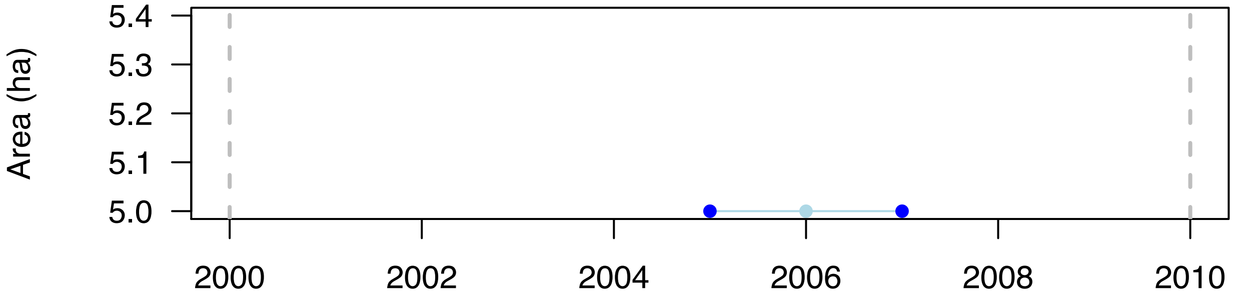

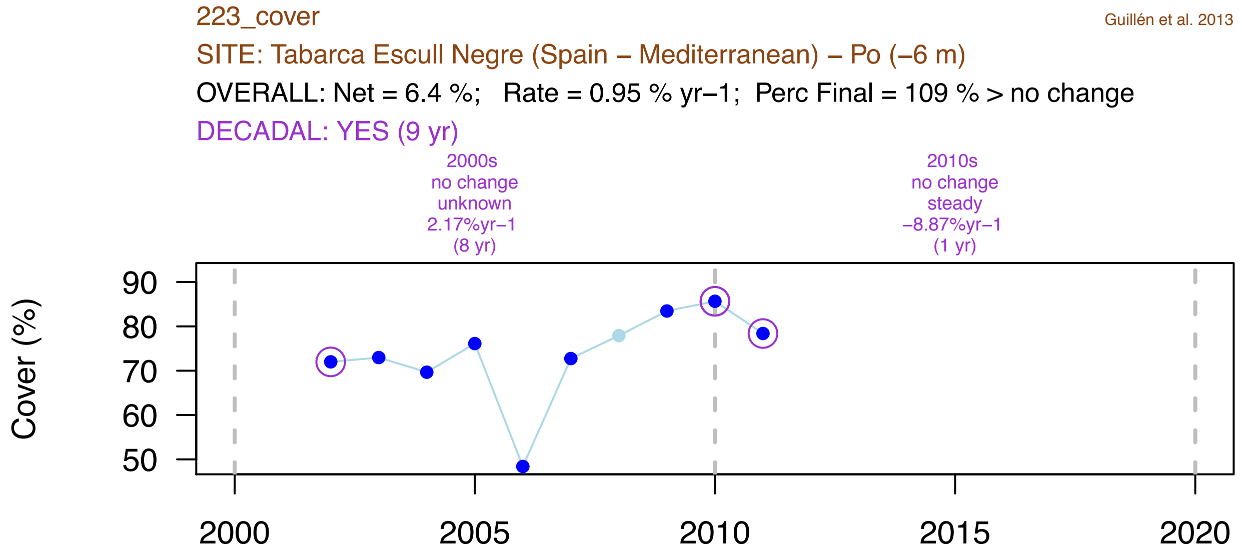
223_cover
Guillén et al. 2013
SITE: Tabarca Escull Negre (Spain – Mediterranean) – Po (–6 m)
OVERALL: Net = 6.4 %; Rate = 0.95 % yr–1; Perc Final = 109 % > no change
DECADAL: YES (9 yr)
2000s
no change
unknown
2.17%yr–1
(8 yr)
2010s
no change
steady
–8.87%yr–1
(1 yr)
Cover (%)
90
80
70
60
50
2000
2005
2010
2015
2020

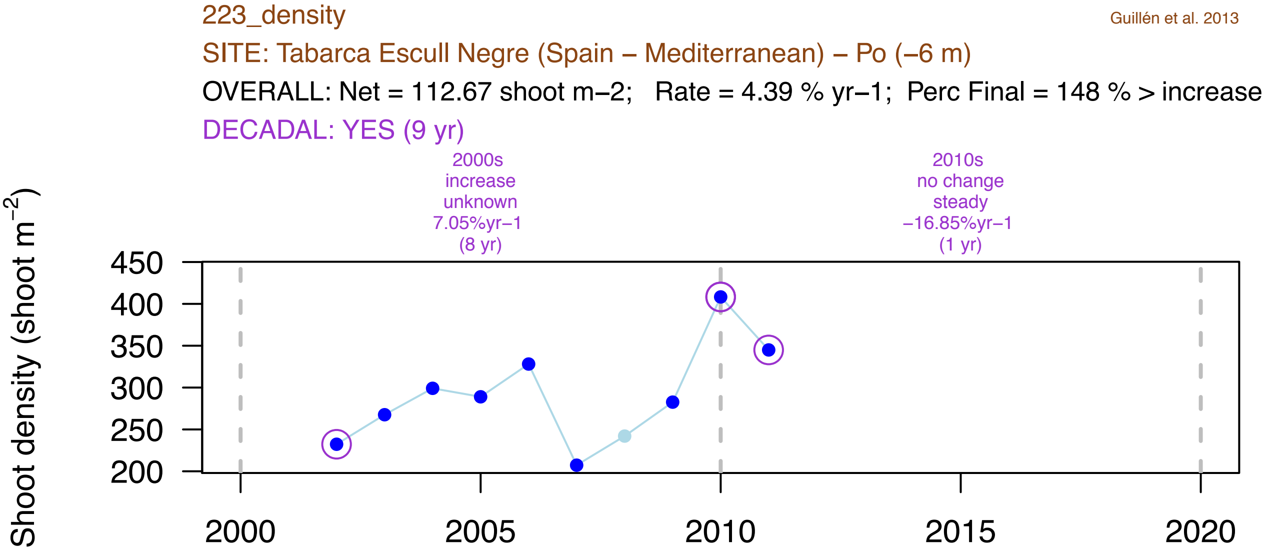
223_density
Guillén et al. 2013
SITE: Tabarca Escull Negre (Spain – Mediterranean) – Po (–6 m)
OVERALL: Net = 112.67 shoot m–2; Rate = 4.39 % yr–1; Perc Final = 148 % > increase
DECADAL: YES (9 yr)
2000s
increase
unknown
7.05%yr–1
(8 yr)
2010s
no change
steady
–16.85%yr–1
(1 yr)
Shoot density (shoot m–2)
450
400
350
300
250
200
2000
2005
2010
2015
2020

224_cover

Guillén et al. 2013

SITE: Tabarca La Nao (Spain – Mediterranean) – Po (–12 m)

OVERALL: Net = –21.83 %;   Rate = –2.74 % yr–1; Perc Final = 78 % > no change

DECADAL: YES (9 yr)

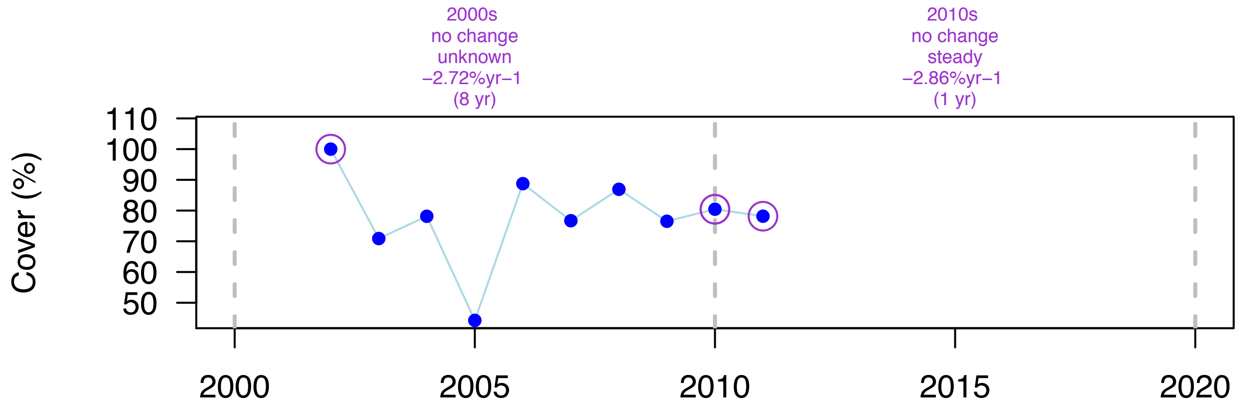

224_density

Guillén et al. 2013

SITE: Tabarca La Nao (Spain – Mediterranean) – Po (–12 m)

OVERALL: Net = 50.12 shoot m–2;   Rate = 2.06 % yr–1; Perc Final = 120 % > no change

DECADAL: YES (9 yr)

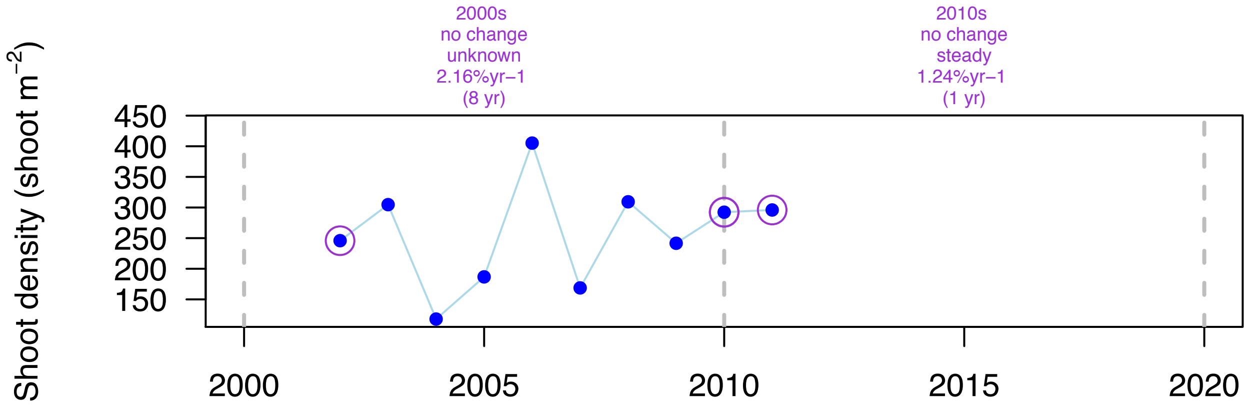

225_cover Guillén et al. 2013

SITE: Tabarca La Nao (Spain – Mediterranean) – Po (–6 m)

OVERALL: Net = –3.86 %; Rate = –0.49 % yr–1; Perc Final = 96 % > no change

DECADAL: YES (9 yr)

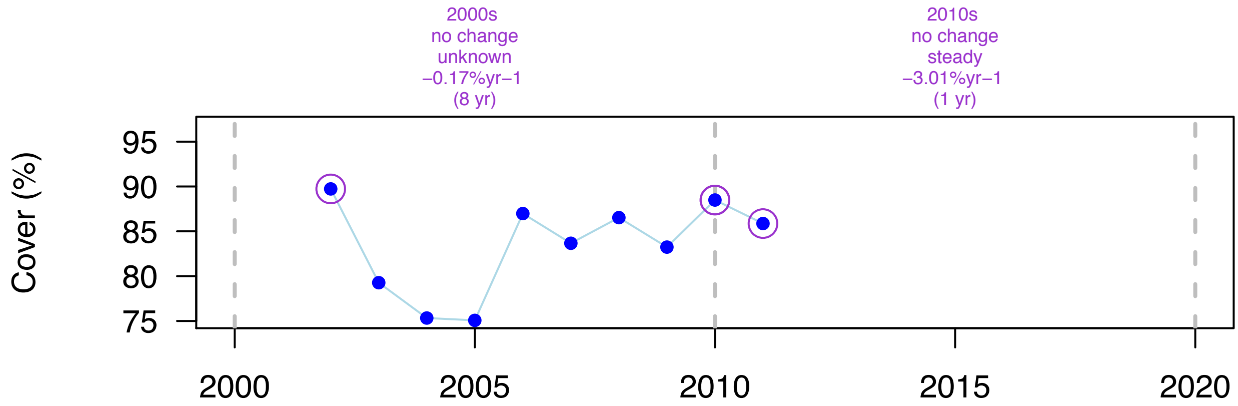

225_density Guillén et al. 2013

SITE: Tabarca La Nao (Spain – Mediterranean) – Po (–6 m)

OVERALL: Net = 144.5 shoot m–2; Rate = 5.19 % yr–1; Perc Final = 160 % > increase

DECADAL: YES (9 yr)

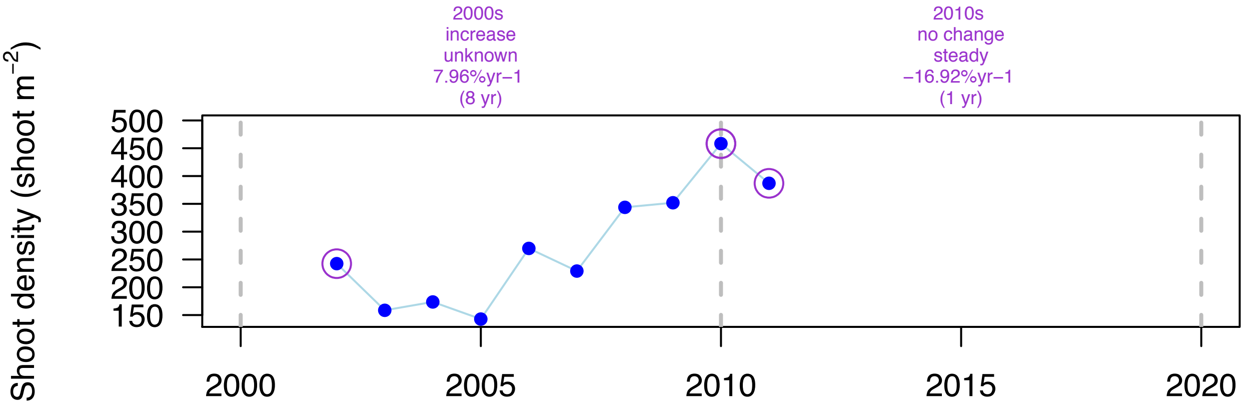

226_cover

Guillén et al. 2013

SITE: Torrevieja (Spain – Mediterranean) – Po (–11 m)

OVERALL: Net = 26.4 %;   Rate = 5.39 % yr–1; Perc Final = 146 % > increase

DECADAL: NO (7 yr)

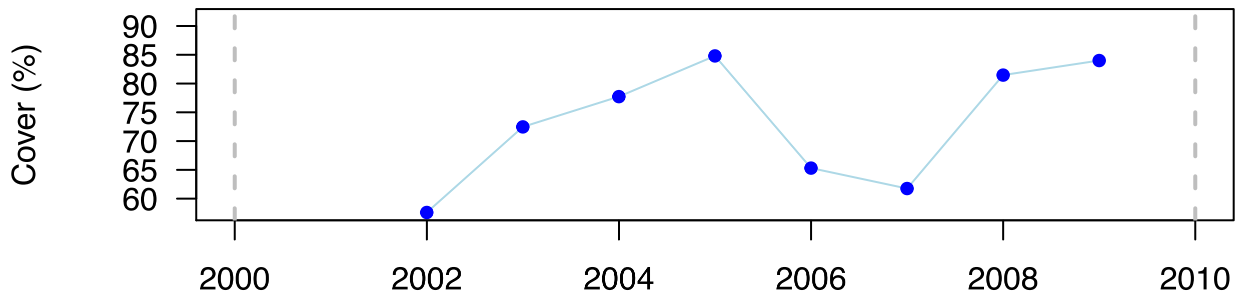

226_density

Guillén et al. 2013

SITE: Torrevieja (Spain – Mediterranean) – Po (–11 m)

OVERALL: Net = 2.94 shoot m–2;   Rate = 0.2 % yr–1; Perc Final = 101 % > no change

DECADAL: NO (7 yr)

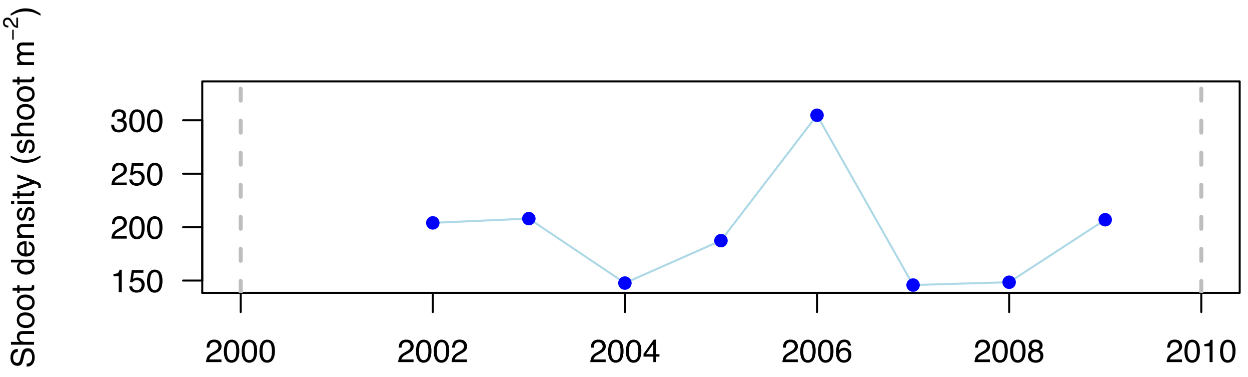

227_cover

Guillén et al. 2013

SITE: Torrevieja (Spain – Mediterranean) – Po (–6 m)

OVERALL: Net = 37.15 %;   Rate = 8.01 % yr–1; Perc Final = 162 % > increase

DECADAL: NO (6 yr)

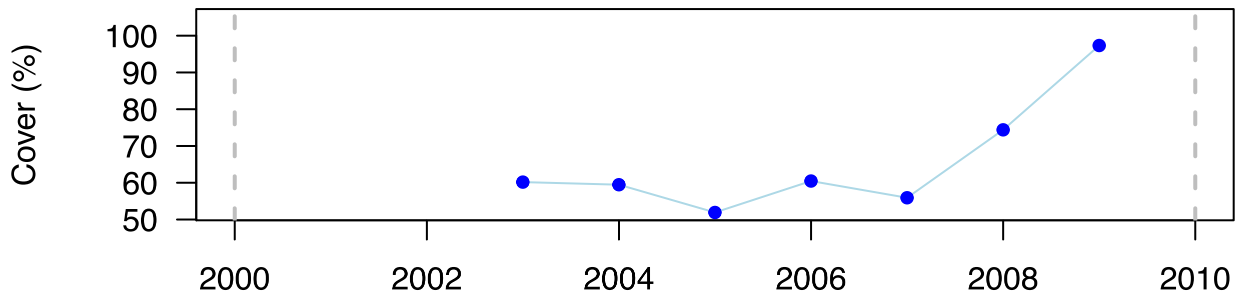

227_density

Guillén et al. 2013

SITE: Torrevieja (Spain – Mediterranean) – Po (–6 m)

OVERALL: Net = 98.13 shoot m–2;   Rate = 4.82 % yr–1; Perc Final = 134 % > increase

DECADAL: NO (6 yr)

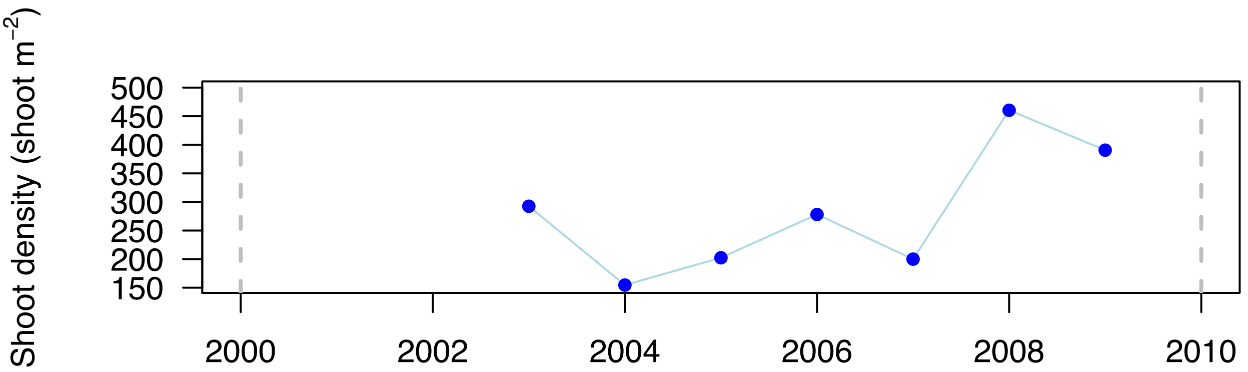

228_cover

Ramos–Esplá et al. 2006, 2007

SITE: Pilar de la Horadada (Spain – Mediterranean) – Po (–15.5 m)

OVERALL: Net = –5.89 %;   Rate = –3.16 % yr–1; Perc Final = 94 % > no change

DECADAL: NO (2 yr)

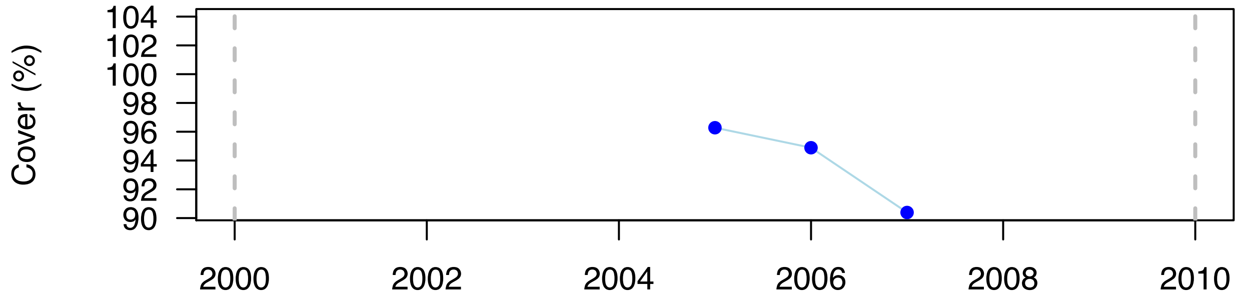

228_density

Ramos–Esplá et al. 2006, 2007

SITE: Pilar de la Horadada (Spain – Mediterranean) – Po (–15.5 m)

OVERALL: Net = 33.57 shoot m–2;   Rate = 5.91 % yr–1; Perc Final = 113 % > no change

DECADAL: NO (2 yr)

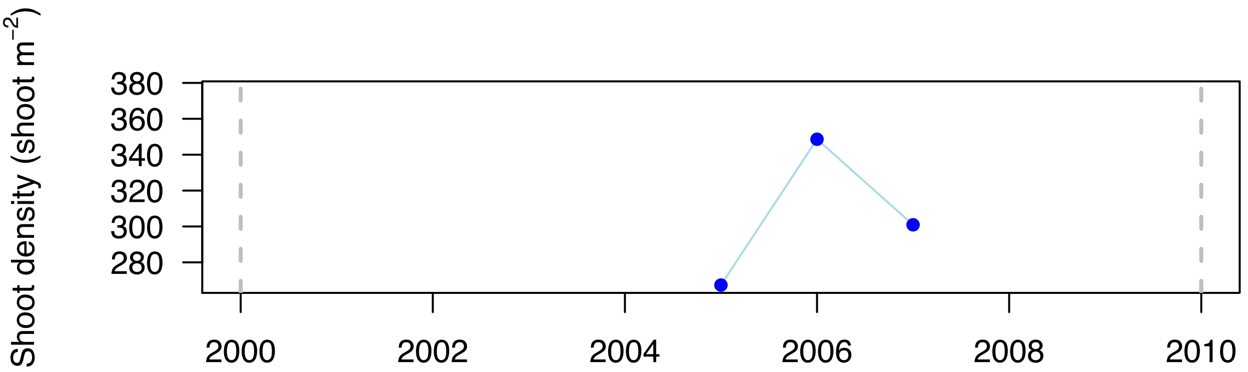

229_cover

Ramos–Esplá et al. 2006, 2007

SITE: Cabo Roig (Spain – Mediterranean) – Po (–13.8 m)

OVERALL: Net = –3.44 %;   Rate = –1.97 % yr–1; Perc Final = 96 % > no change

DECADAL: NO (2 yr)

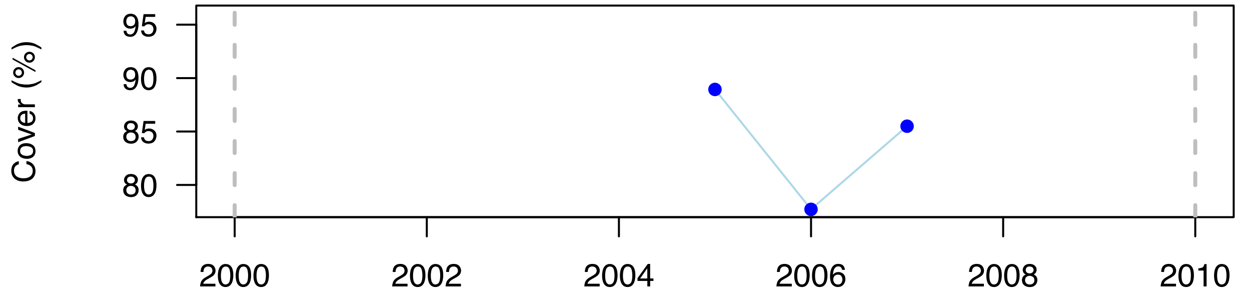

229_density

Ramos–Esplá et al. 2006, 2007

SITE: Cabo Roig (Spain – Mediterranean) – Po (–13.8 m)

OVERALL: Net = 41.2 shoot m–2;   Rate = 6.91 % yr–1; Perc Final = 115 % > no change

DECADAL: NO (2 yr)

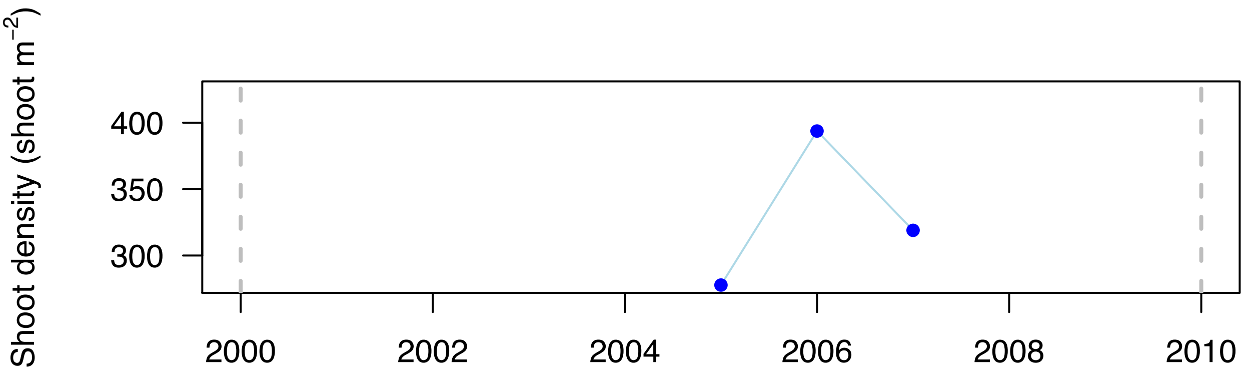

230_area

Boudouresque et al. 2006

SITE: Marseilles (entire) (France – Mediterranean) – Po (? m)

OVERALL: Net = –208 ha;   Rate = –0.62 % yr–1; Perc Final = 56 % > decrease

DECADAL: YES (94 yr)

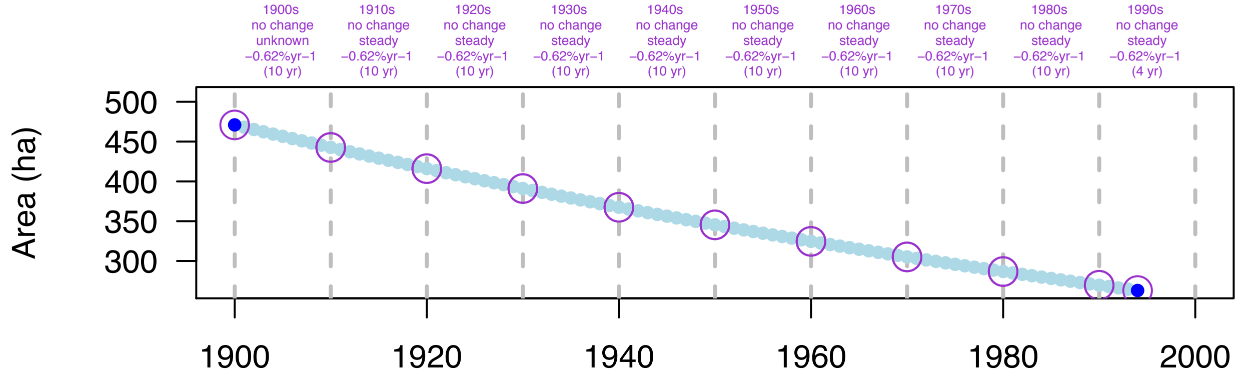

231_cover

Ramos–Esplá et al. 2006, 2007, 2008, 2009, 2010

SITE: Cabo de Santa Pola (Spain – Mediterranean) – Po (–14.5 m)

OVERALL: Net = 5.41 %;   Rate = 1.19 % yr–1; Perc Final = 106 % > no change

DECADAL: NO (5 yr)

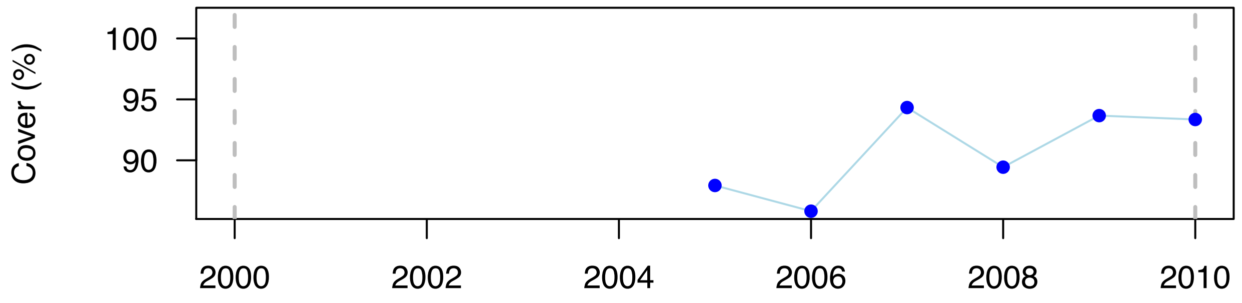

231_density
Ramos–Esplá et al. 2006, 2007, 2008, 2009, 2010
SITE: Cabo de Santa Pola (Spain – Mediterranean) – Po (–14.5 m)
OVERALL: Net = –84.03 shoot m–2; Rate = –5.65 % yr–1; Perc Final = 75 % > no change
DECADAL: NO (5 yr)

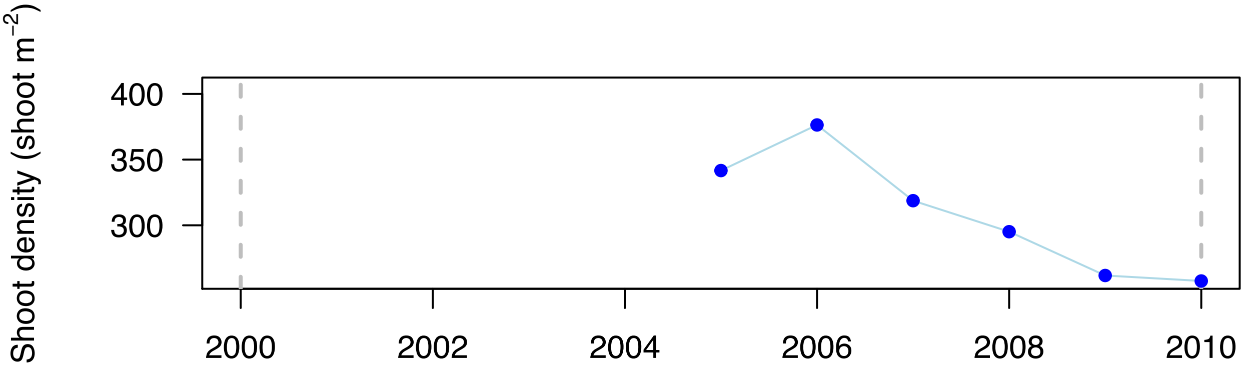

232_cover
Ramos–Esplá et al. 2006, 2007, 2008, 2009, 2010, 2011
SITE: Alicante (Spain – Mediterranean) – Po (–16.8 m)
OVERALL: Net = –17.11 %; Rate = –9.39 % yr–1; Perc Final = 57 % > decrease
DECADAL: NO (6 yr)

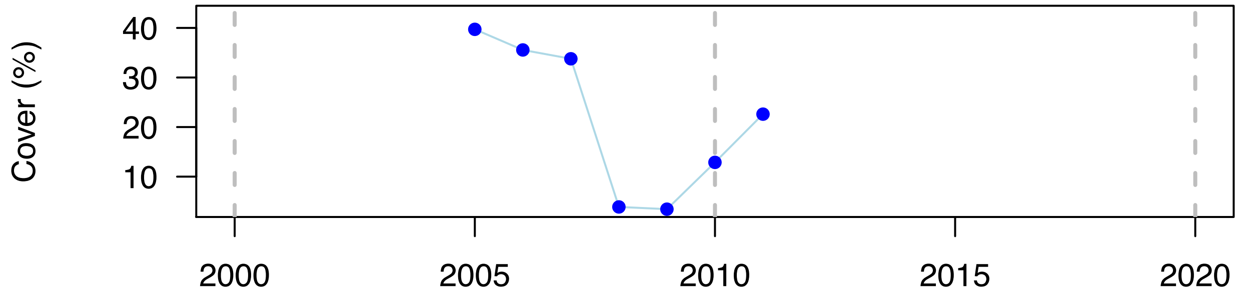

232_density

Ramos–Esplá et al. 2006, 2007, 2008, 2009, 2010, 2011

SITE: Alicante (Spain – Mediterranean) – Po (–16.8 m)

OVERALL: Net = –97.92 shoot m–2;   Rate = –18.39 % yr–1; Perc Final = 33 % > decrease

DECADAL: NO (6 yr)

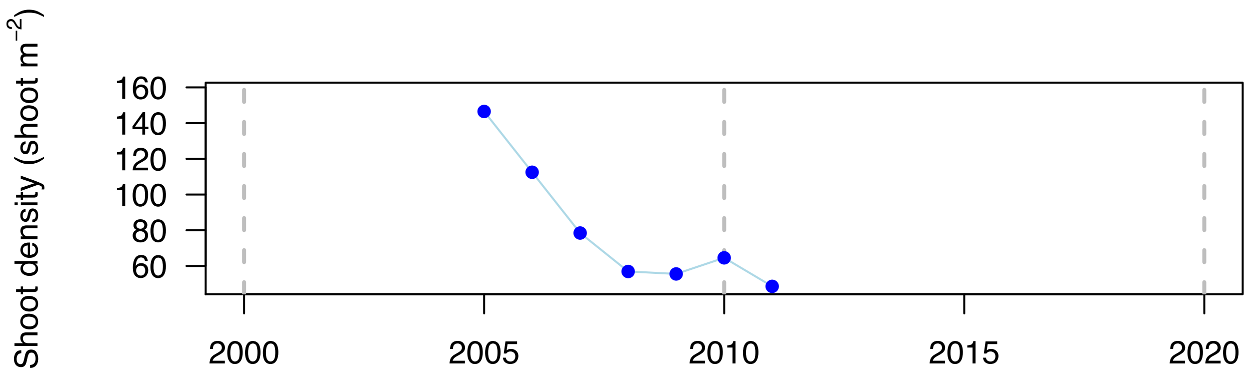

233_cover

Ramos–Esplá et al. 2006, 2007, 2008

SITE: Cabo Huertas (Spain – Mediterranean) – Po (–14 m)

OVERALL: Net = –9.39 %;   Rate = –3.9 % yr–1; Perc Final = 89 % > no change

DECADAL: NO (3 yr)

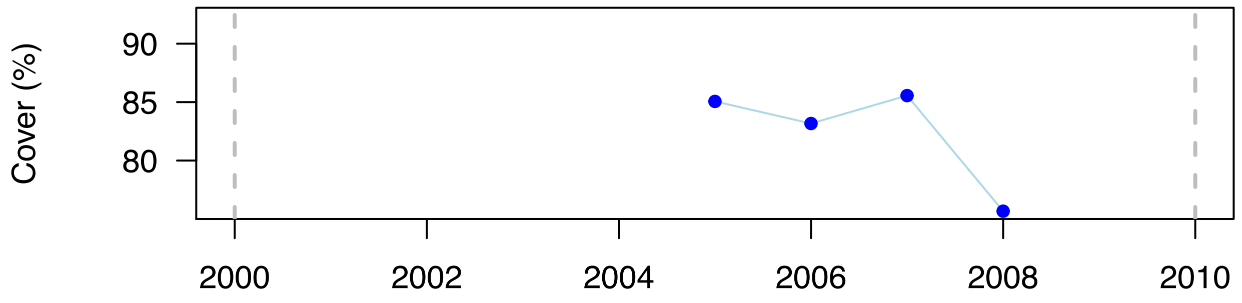

233_density

Ramos–Esplá et al. 2006, 2007, 2008

SITE: Cabo Huertas (Spain – Mediterranean) – Po (–14 m)

OVERALL: Net = –9.73 shoot m–2; Rate = –1.02 % yr–1; Perc Final = 97 % > no change

DECADAL: NO (3 yr)

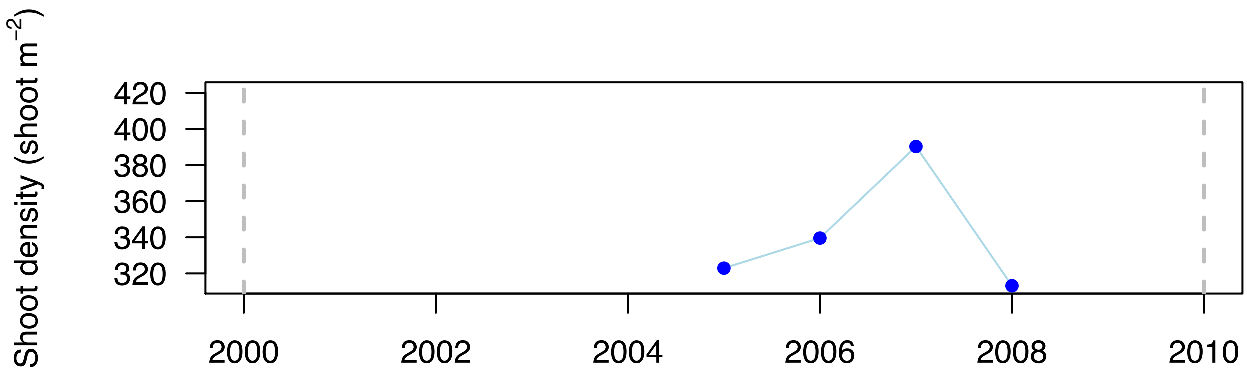

235_cover

Ramos–Esplá et al. 2006, 2007, 2008

SITE: Benidorm (Spain – Mediterranean) – Po (–14 m)

OVERALL: Net = –9.45 %; Rate = –3.35 % yr–1; Perc Final = 90 % > no change

DECADAL: NO (3 yr)

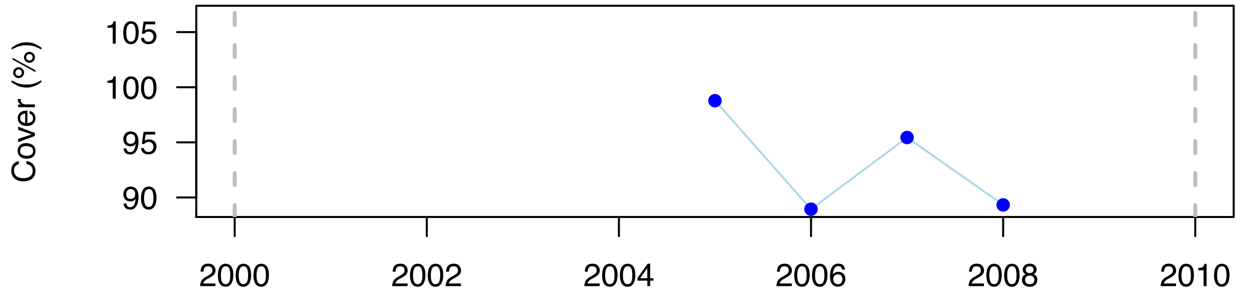

235_density

Ramos−Esplá et al. 2006, 2007, 2008

SITE: Benidorm (Spain − Mediterranean) − Po (−14 m)

OVERALL: Net = 13.19 shoot m−2;   Rate = 2.14 % yr−1; Perc Final = 107 % > no change

DECADAL: NO (3 yr)

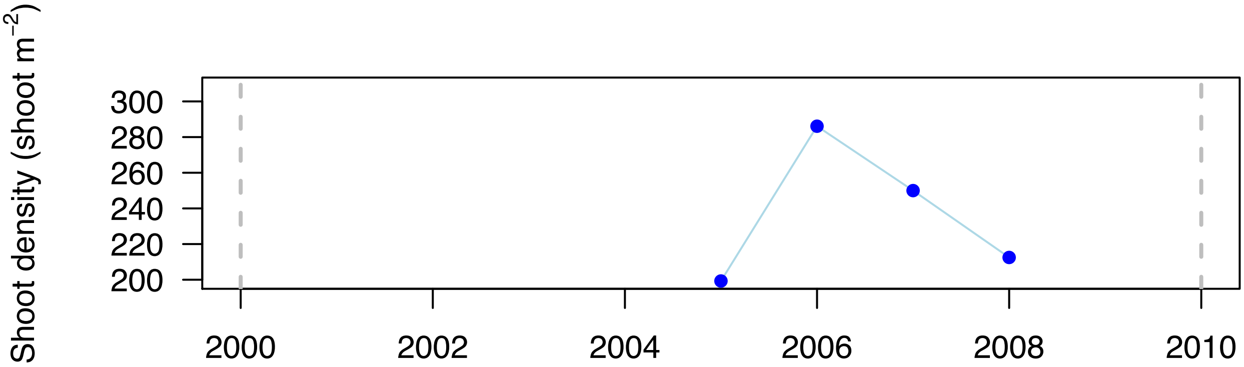

236_cover

Ramos−Esplá et al. 2006, 2007, 2008, 2009, 2010, 2011

SITE: Altea (Spain − Mediterranean) − Po (−15 m)

OVERALL: Net = −1.56 %;   Rate = −0.27 % yr−1; Perc Final = 98 % > no change

DECADAL: NO (6 yr)

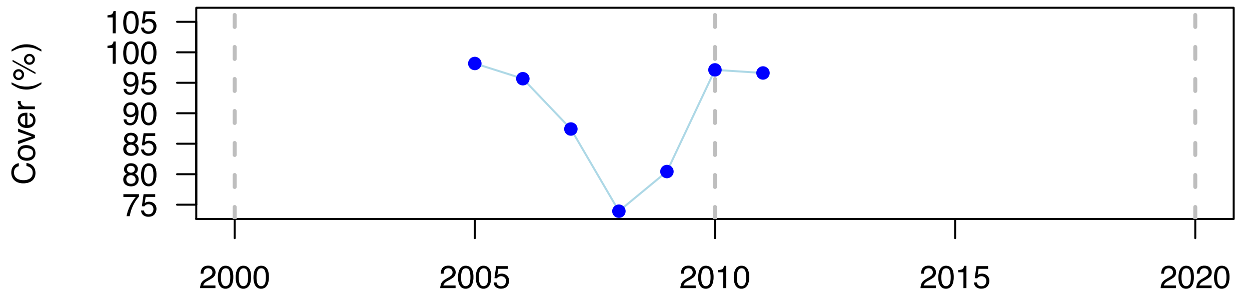

236_density

Ramos–Esplá et al. 2006, 2007, 2008, 2009, 2010, 2011

SITE: Altea (Spain – Mediterranean) – Po (–15 m)

OVERALL: Net = –102.08 shoot m–2;   Rate = –8.02 % yr–1; Perc Final = 62 % > decrease

DECADAL: NO (6 yr)

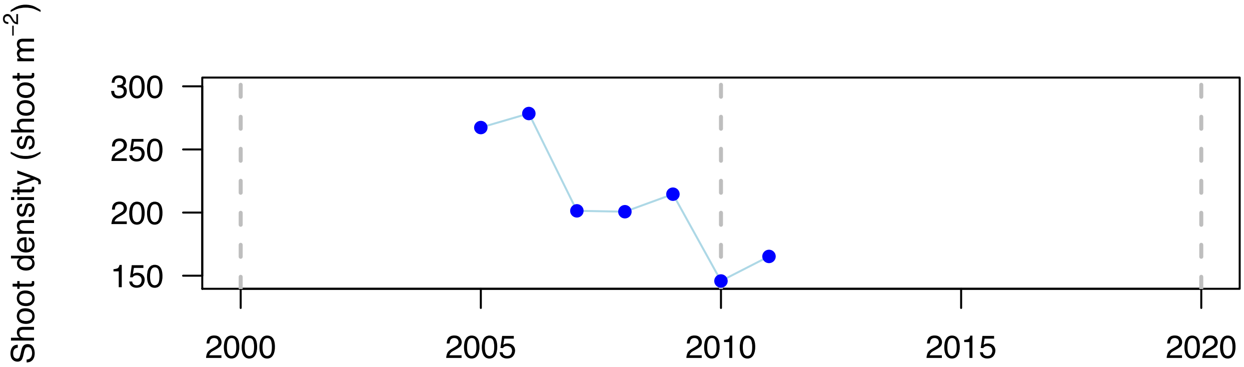

237_cover

Ramos–Esplá et al. 2006, 2007, 2008, 2009, 2010

SITE: Calpe (Spain – Mediterranean) – Po (–14 m)

OVERALL: Net = 10.28 %;   Rate = 2.23 % yr–1; Perc Final = 112 % > no change

DECADAL: NO (5 yr)

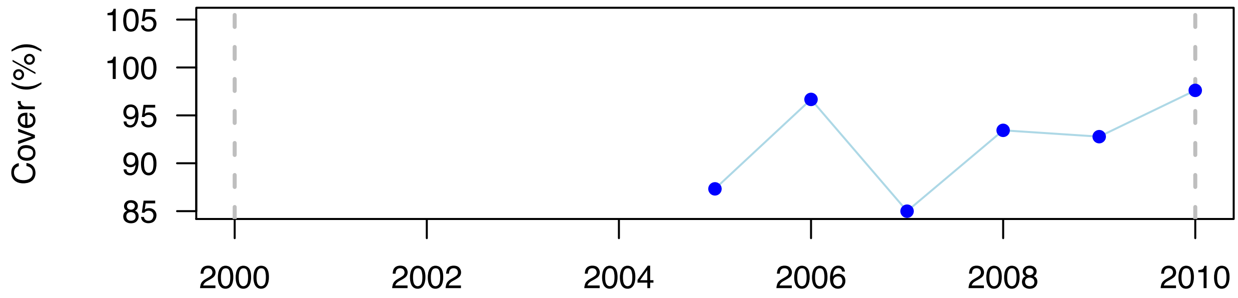

237_density

Ramos–Esplá et al. 2006, 2007, 2008, 2009, 2010

SITE: Calpe (Spain – Mediterranean) – Po (–14 m)

OVERALL: Net = –95.83 shoot m–2; Rate = –6.71 % yr–1; Perc Final = 71 % > decrease

DECADAL: NO (5 yr)

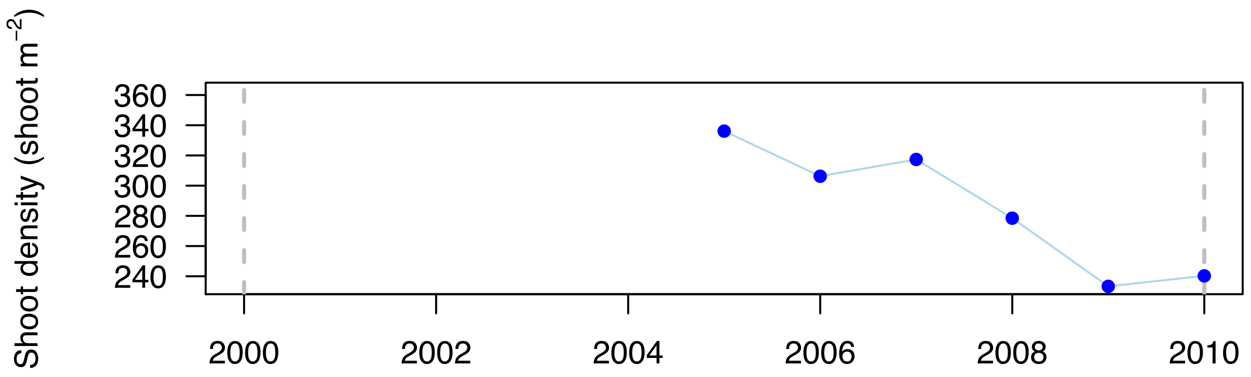

238_cover

Ramos–Esplá et al. 2006, 2007, 2008

SITE: Jávea (Spain – Mediterranean) – Po (–15 m)

OVERALL: Net = 23.6 %; Rate = 11.28 % yr–1; Perc Final = 140 % > increase

DECADAL: NO (3 yr)

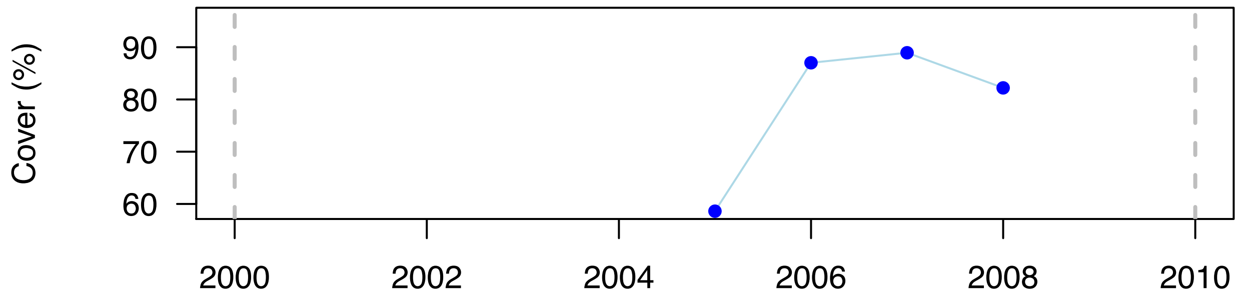

238_density

Ramos–Esplá et al. 2006, 2007, 2008

SITE: Jávea (Spain – Mediterranean) – Po (–15 m)

OVERALL: Net = 56.95 shoot m–2;   Rate = 6.91 % yr–1; Perc Final = 123 % > no change

DECADAL: NO (3 yr)

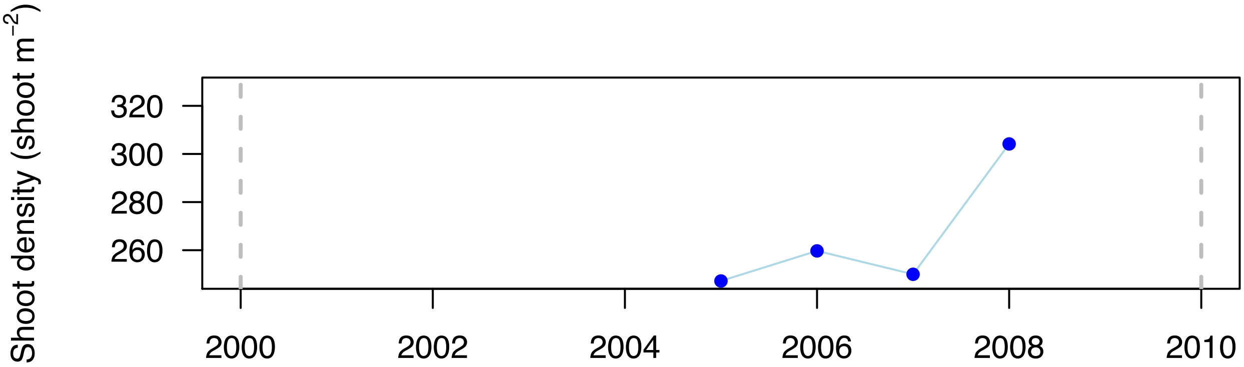

239_cover

Ramos–Esplá et al. 2006, 2007, 2008

SITE: Denia (Spain – Mediterranean) – Po (–15 m)

OVERALL: Net = –5.5 %;   Rate = –4.07 % yr–1; Perc Final = 89 % > no change

DECADAL: NO (3 yr)

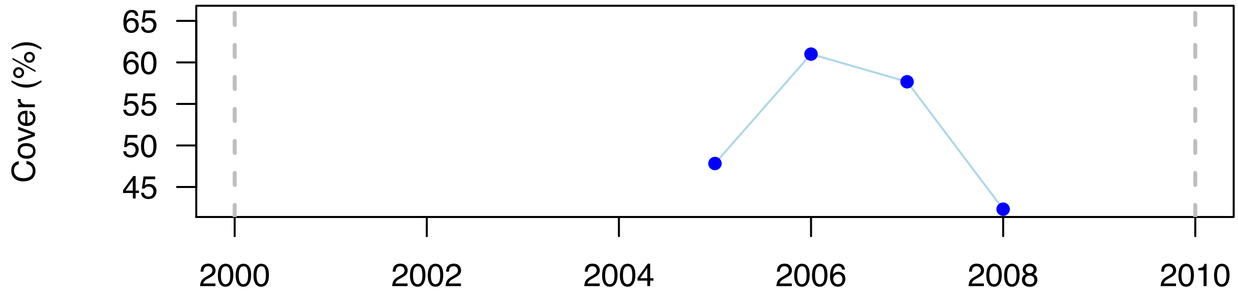

239_density
Ramos−Esplá et al. 2006, 2007, 2008
SITE: Denia (Spain − Mediterranean) − Po (−15 m)
OVERALL: Net = 85.42 shoot m−2;   Rate = 11.2 % yr−1; Perc Final = 140 % > increase
DECADAL: NO (3 yr)

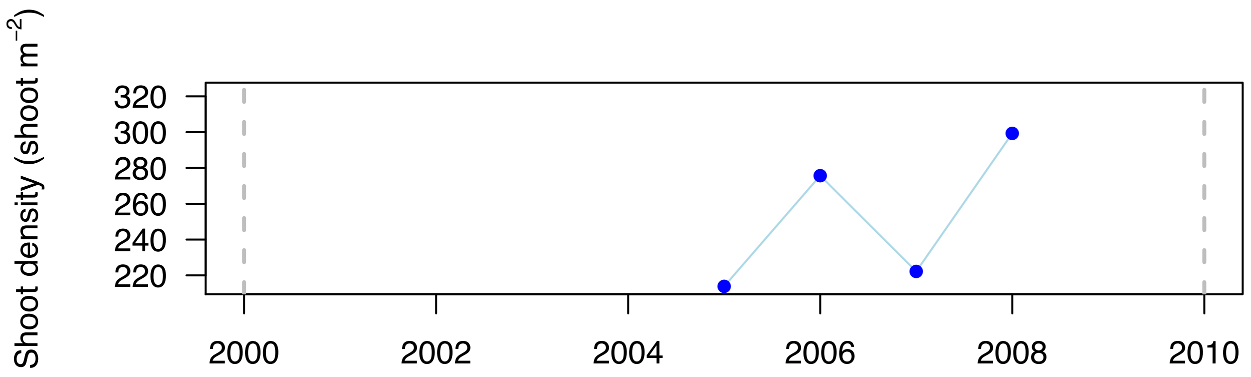

240_cover
Ramos−Esplá et al. 2006, 2007, 2008, 2009, 2010
SITE: Castellón (Spain − Mediterranean) − Po (−15.5 m)
OVERALL: Net = −1.61 %;   Rate = −9.76 % yr−1; Perc Final = 61 % > decrease
DECADAL: NO (5 yr)

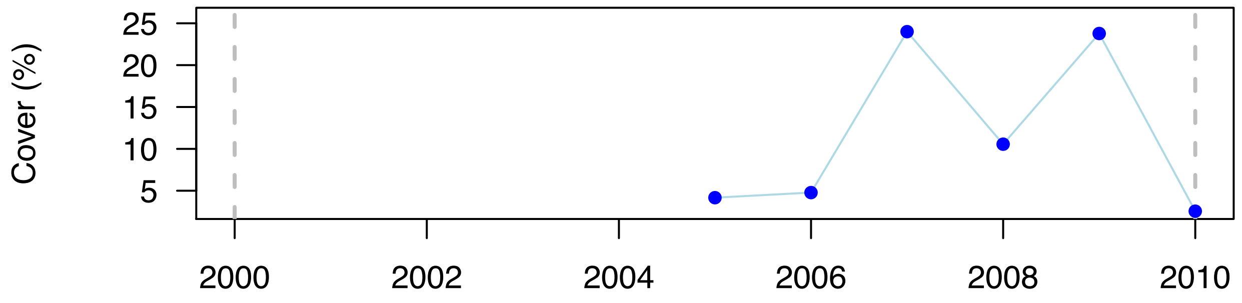

240_density

Ramos−Esplá et al. 2006, 2007, 2008, 2009, 2010

SITE: Castellón (Spain − Mediterranean) − Po (−15.5 m)

OVERALL: Net = −24.3 shoot m−2;   Rate = −6.9 % yr−1; Perc Final = 71 % > decrease

DECADAL: NO (5 yr)

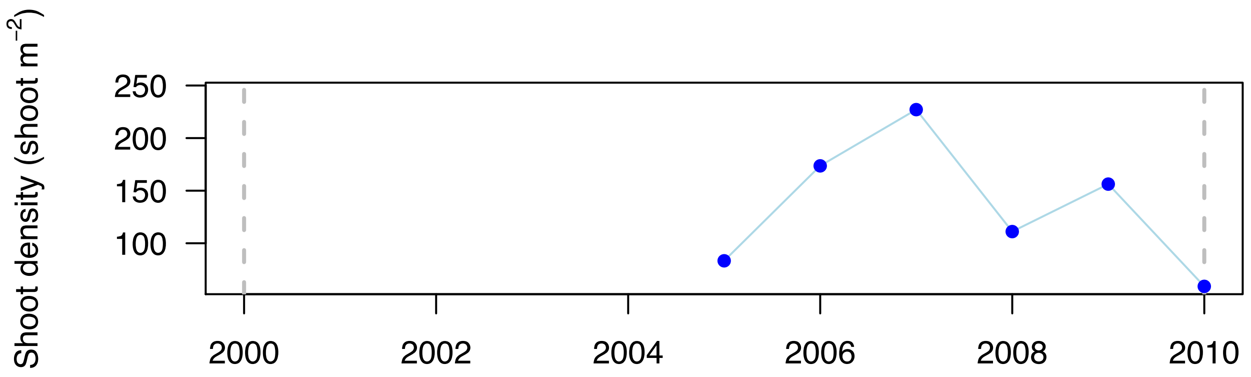

241_cover

Ramos−Esplá et al. 2006, 2007, 2008, 2009, 2010, 2011

SITE: Benicassim (Spain − Mediterranean) − Po (−15 m)

OVERALL: Net = 10 %;   Rate = 11.83 % yr−1; Perc Final = 203 % > increase

DECADAL: NO (6 yr)

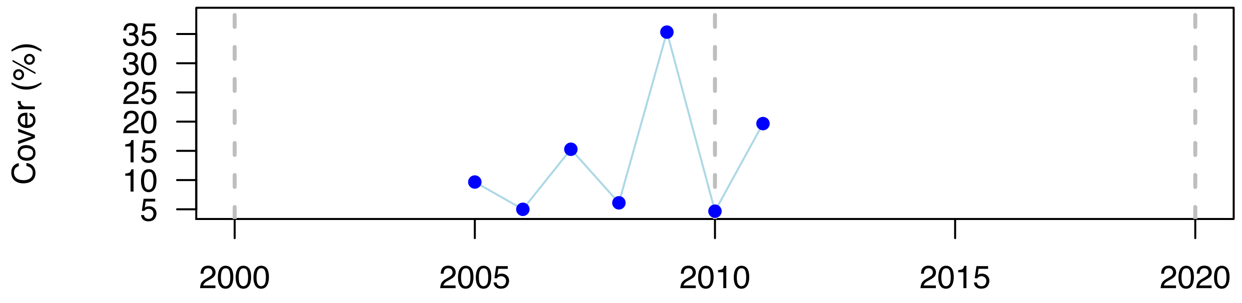

241_density

Ramos–Esplá et al. 2006, 2007, 2008, 2009, 2010, 2011

SITE: Benicassim (Spain – Mediterranean) – Po (–15 m)

OVERALL: Net = –43.75 shoot m–2;   Rate = –5.62 % yr–1; Perc Final = 71 % > decrease

DECADAL: NO (6 yr)

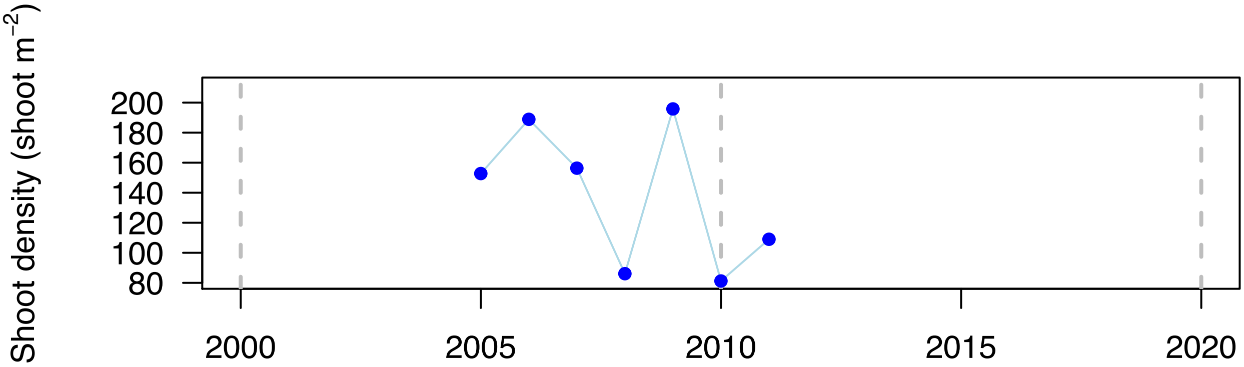

242_cover

Ramos–Esplá et al. 2006, 2007, 2008, 2009, 2010, 2011

SITE: Oropesa (Spain – Mediterranean) – Po (–15 m)

OVERALL: Net = –22.33 %;   Rate = –6.27 % yr–1; Perc Final = 73 % > decrease

DECADAL: NO (5 yr)

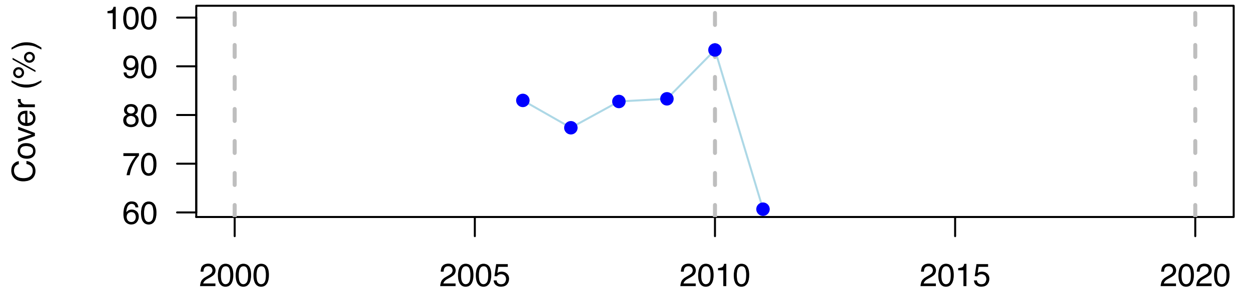

242_density
Ramos–Esplá et al. 2006, 2007, 2008, 2009, 2010, 2011

SITE: Oropesa (Spain – Mediterranean) – Po (–15 m)

OVERALL: Net = –89.58 shoot m–2; Rate = –8.11 % yr–1; Perc Final = 67 % > decrease

DECADAL: NO (5 yr)

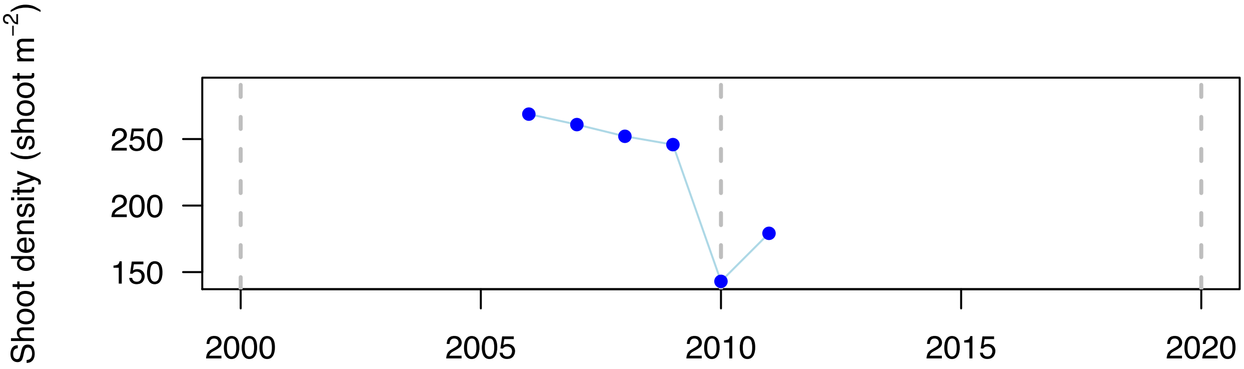

243_cover
Sánchez–Lizaso et al. 2013

SITE: San Pedro del Pinatar (Spain – Mediterranean) – Po (–28.5 m)

OVERALL: Net = 1.3 %; Rate = 0.34 % yr–1; Perc Final = 102 % > no change

DECADAL: NO (7 yr)

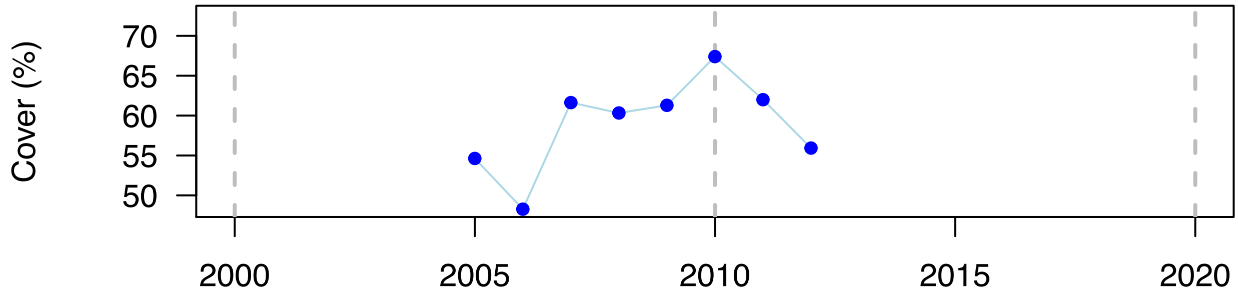

243_density

Sánchez–Lizaso et al. 2013

SITE: San Pedro del Pinatar (Spain – Mediterranean) – Po (–28.5 m)

OVERALL: Net = –75 shoot m–2;   Rate = –8.84 % yr–1; Perc Final = 54 % > decrease

DECADAL: NO (7 yr)

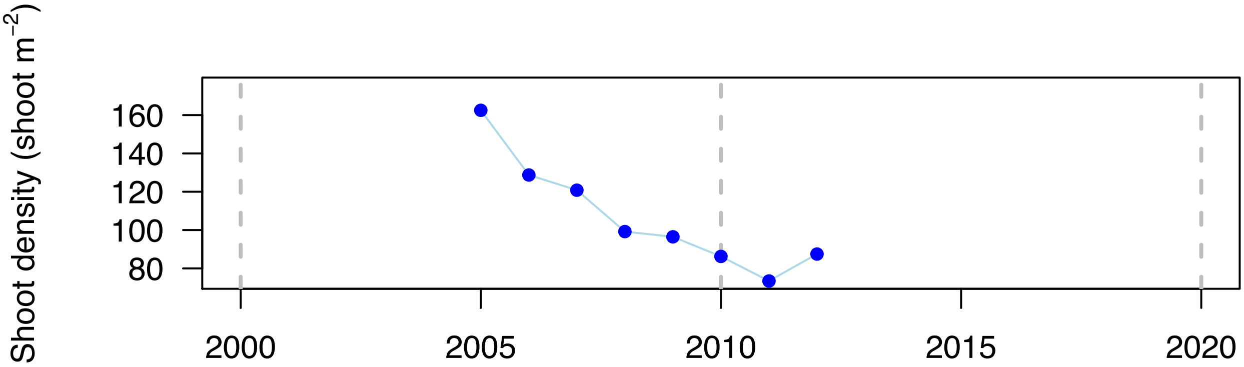

246_area

Ferre–Vera 2012

SITE: Cala Fornells – Caló de Ses Llises (Spain – Mediterranean) – Po (? m)

OVERALL: Net = –1.88 ha;   Rate = –0.2 % yr–1; Perc Final = 97 % > no change

DECADAL: YES (18 yr)

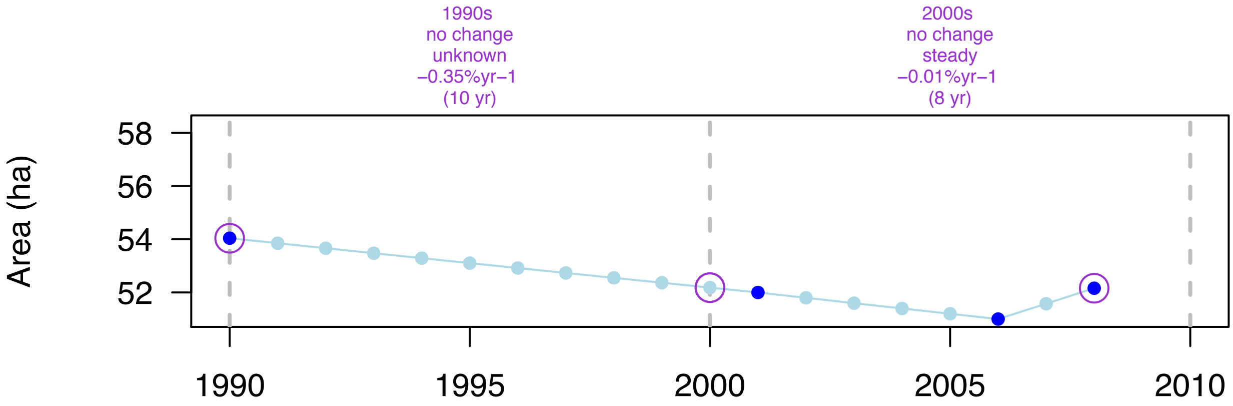

247_area

Sánchez–Camacho 2003

SITE: S'Illot – Cala Millor (Spain – Mediterranean) – Po (–10 m)

OVERALL: Net = –26.5 ha; Rate = –0.44 % yr–1; Perc Final = 82 % > decrease

DECADAL: YES (45 yr)

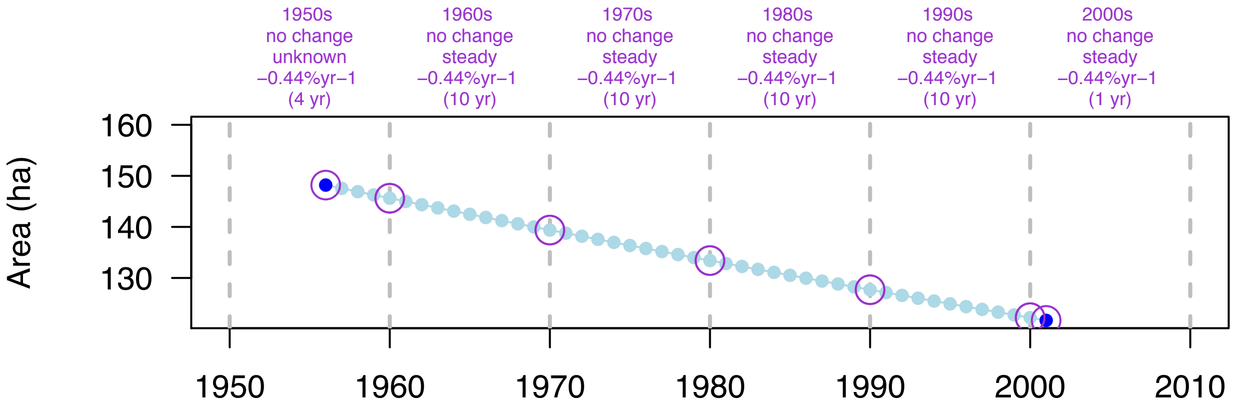

248_area

Sánchez–Camacho 2003

SITE: Magalluf – Portals Nous (Spain – Mediterranean) – Po (–10 m)

OVERALL: Net = –27.6 ha; Rate = –0.27 % yr–1; Perc Final = 89 % > decrease

DECADAL: YES (45 yr)

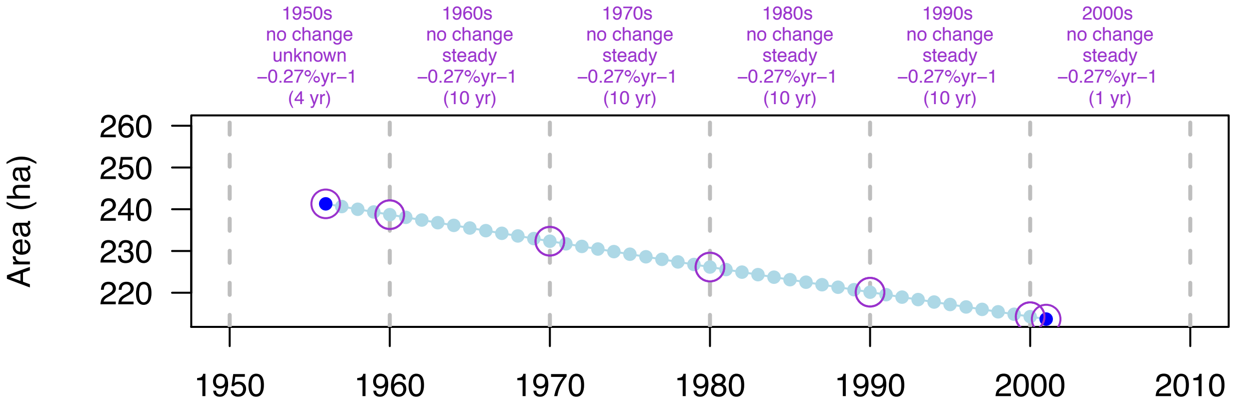

250_density

Terrados and Medina–Pons 2011

SITE: Magalluf Bay – Sa Porrassa (Spain – Mediterranean) – Po (–8 m)

OVERALL: Net = 361.2 shoot m–2;   Rate = 19.55 % yr–1; Perc Final = 219 % > increase

DECADAL: NO (4 yr)

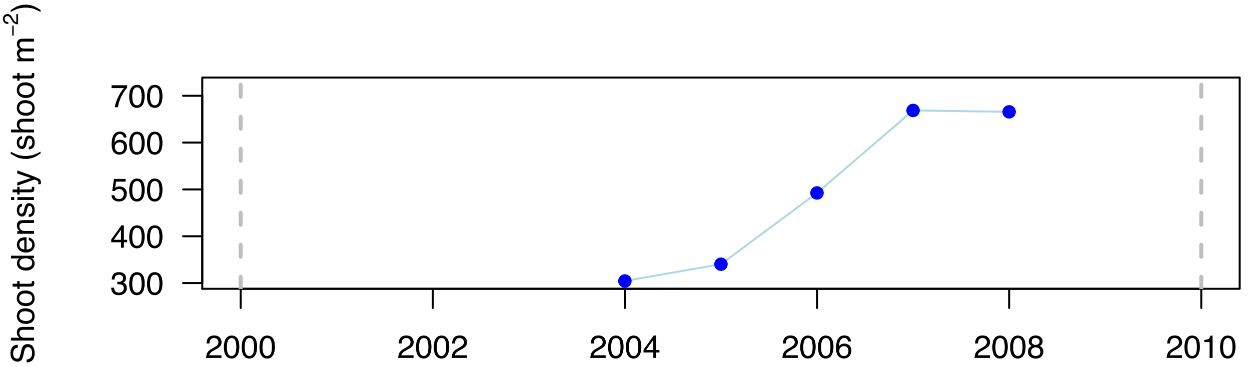

251_density

Terrados and Medina–Pons 2011

SITE: Ses Salines (Spain – Mediterranean) – Po (–8 m)

OVERALL: Net = 229.9 shoot m–2;   Rate = 7.14 % yr–1; Perc Final = 133 % > increase

DECADAL: NO (4 yr)

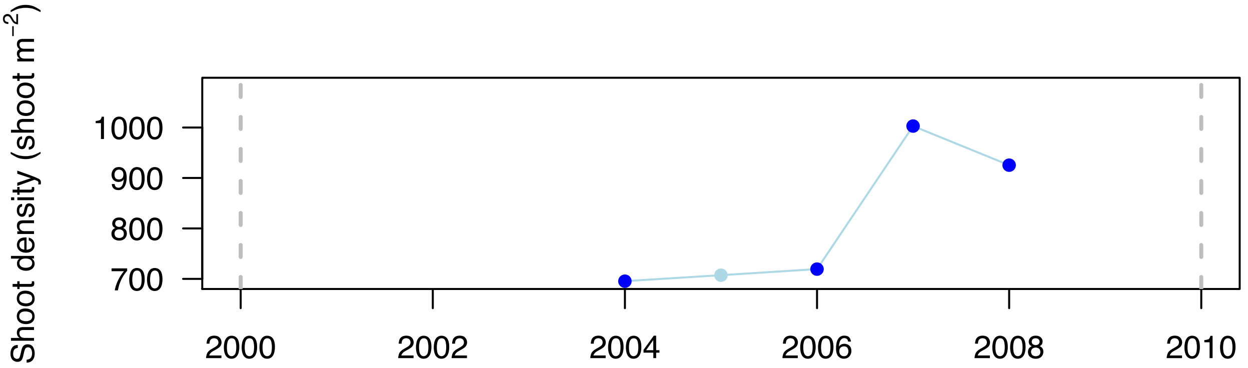

252_density

Díaz–Almela et al. 2009

SITE: Magalluf Bay – Sa Porrassa (Spain – Mediterranean) – Po (–7 m)

OVERALL: Net = –174.69 shoot m–2;   Rate = –9.29 % yr–1; Perc Final = 69 % > decrease

DECADAL: NO (4 yr)

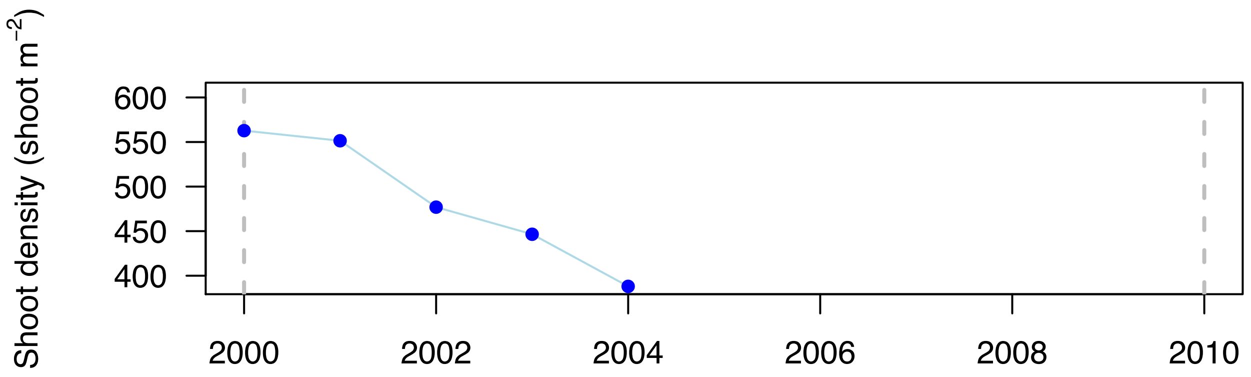

254_area

Ruiz et al. 2001

SITE: Hornillo Bay (Spain – Mediterranean) – Po (? m)

OVERALL: Net = –11.29 ha;   Rate = –3.32 % yr–1; Perc Final = 72 % > decrease

DECADAL: YES (10 yr)

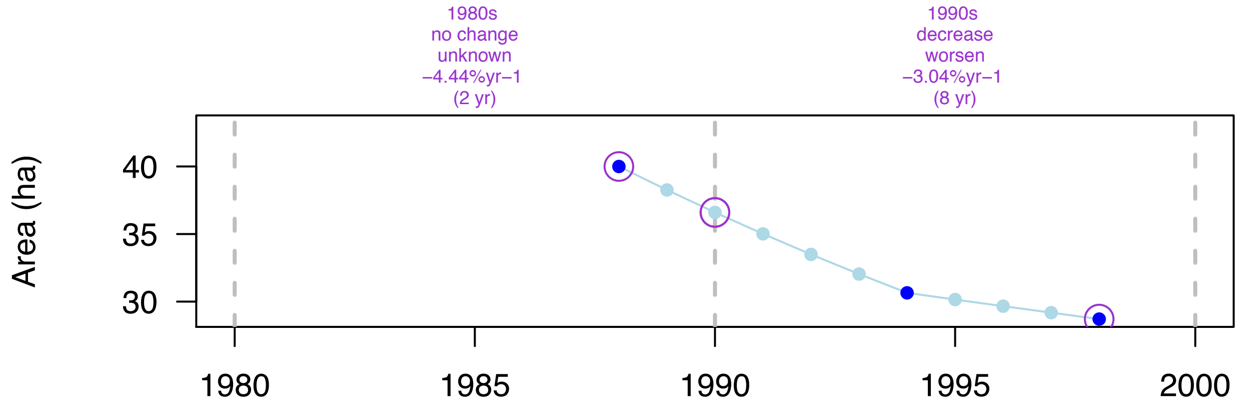

255_area

Garmendia et al. 2013

SITE: Bidasoa Estuary (Spain – Atlantic) – Zn (0 m)

OVERALL: Net = 0.13 ha;   Rate = 1.71 % yr–1; Perc Final = 107 % > no change

DECADAL: NO (4 yr)

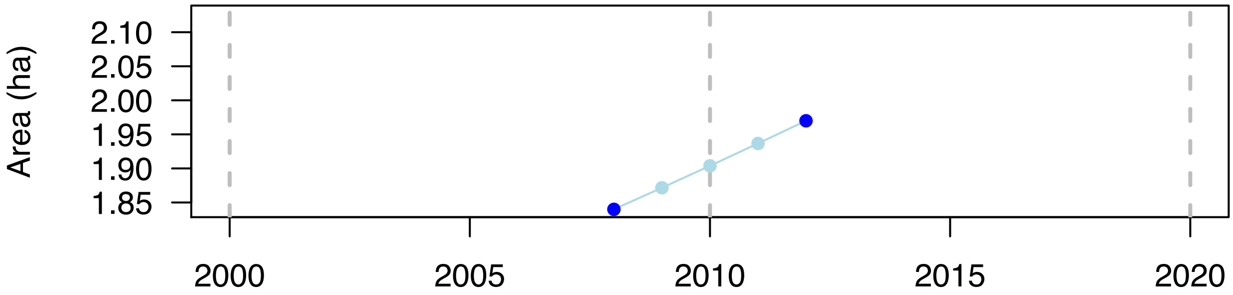

256_area

Garmendia et al. 2013

SITE: Lea Estuary (Spain – Atlantic) – Zn (0 m)

OVERALL: Net = 0.48 ha;   Rate = 37.2 % yr–1; Perc Final = 443 % > increase

DECADAL: NO (4 yr)

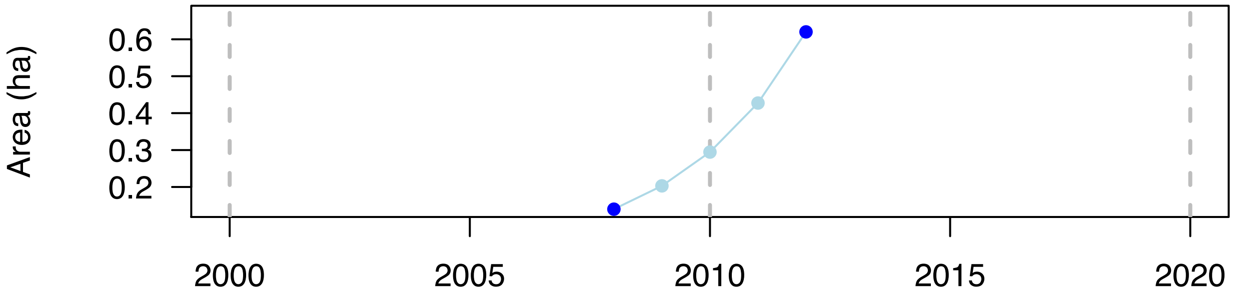

257_area

Garmendia et al. 2013

SITE: Oka Estuary (Kanala) (Spain – Atlantic) – Zn (0 m)

OVERALL: Net = 0.12 ha;   Rate = 3.07 % yr–1; Perc Final = 113 % > increase

DECADAL: NO (4 yr)

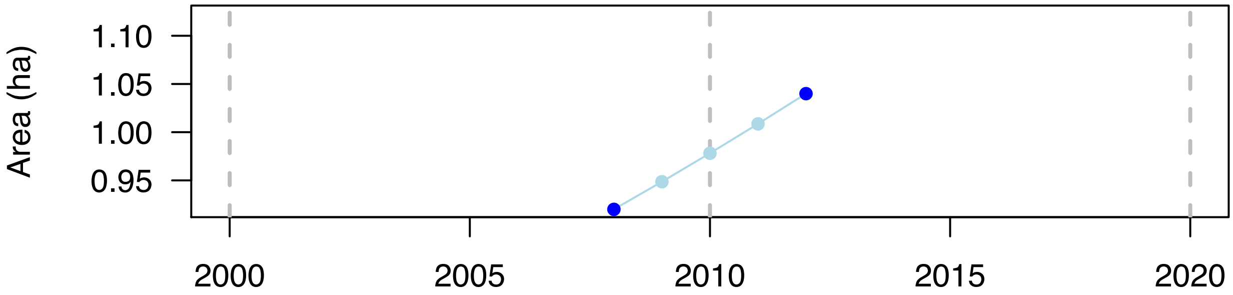

258_area

Garmendia et al. 2013

SITE: Oka Estuary (Arketas) (Spain – Atlantic) – Zn (0 m)

OVERALL: Net = –1.33 ha;   Rate = –30.73 % yr–1; Perc Final = 29 % > decrease

DECADAL: NO (4 yr)

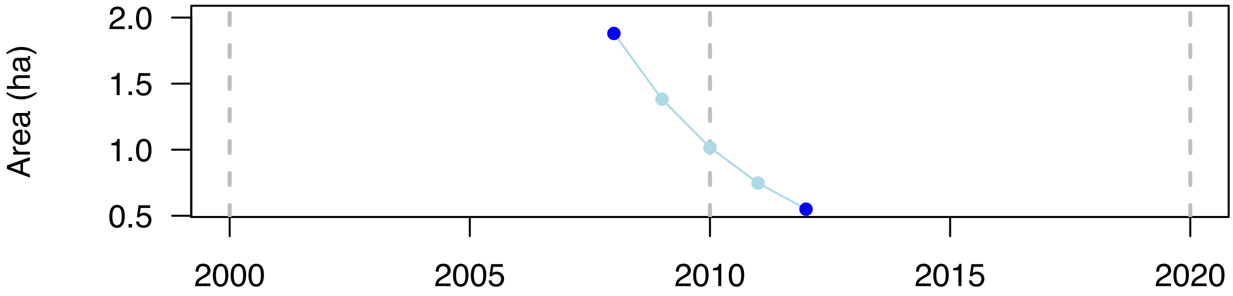

259_area

Garmendia et al. 2013

SITE: Oka Estuary (San Kristobal) (Spain – Atlantic) – Zn (0 m)

OVERALL: Net = −2.3 ha; Rate = −3.44 % yr−1; Perc Final = 87 % > decrease

DECADAL: NO (4 yr)

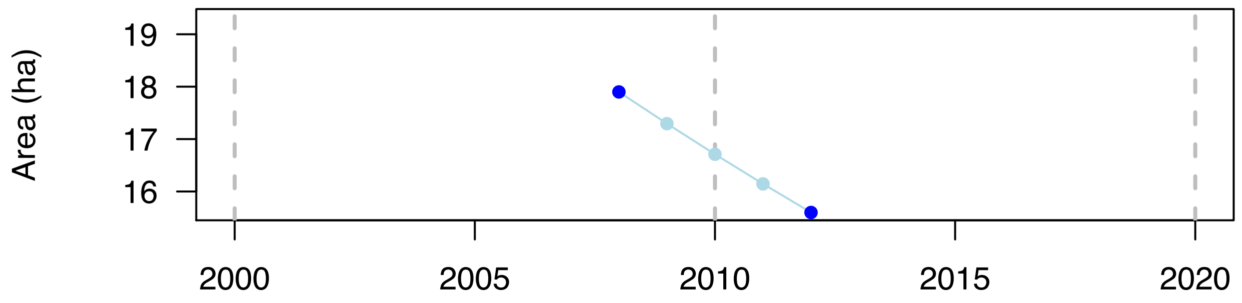

260_biomass

Tuya et al. 2013

SITE: Risco Verde (Spain – Atlantic) – Cn (−10 m)

OVERALL: Net = −413.9 g dw m−2; Rate = −17.89 % yr−1; Perc Final = 6 % > decrease

DECADAL: YES (16 yr)

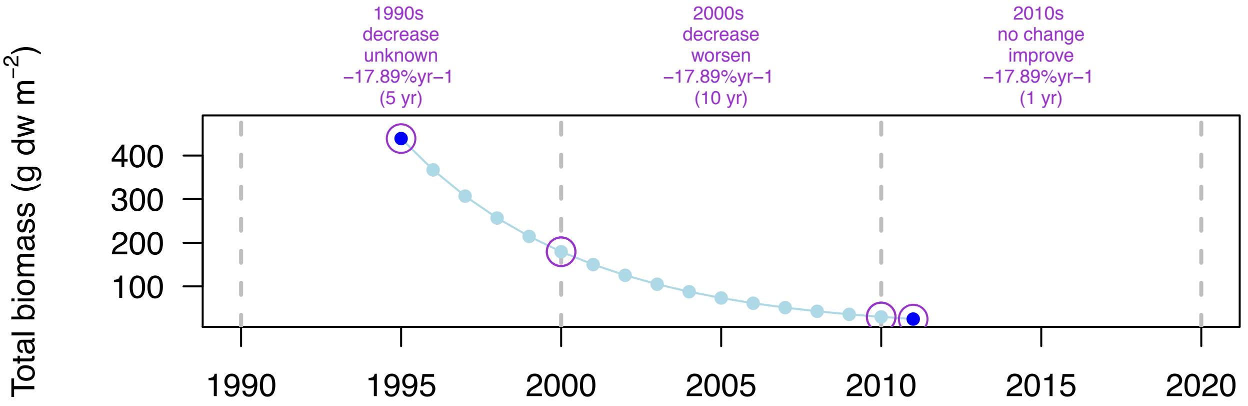

260_density	Tuya et al. 2013

SITE: Risco Verde (Spain – Atlantic) – Cn (–10 m)

OVERALL: Net = –1666 shoot m–2;   Rate = –20.42 % yr–1; Perc Final = 4 % > decrease

DECADAL: YES (16 yr)

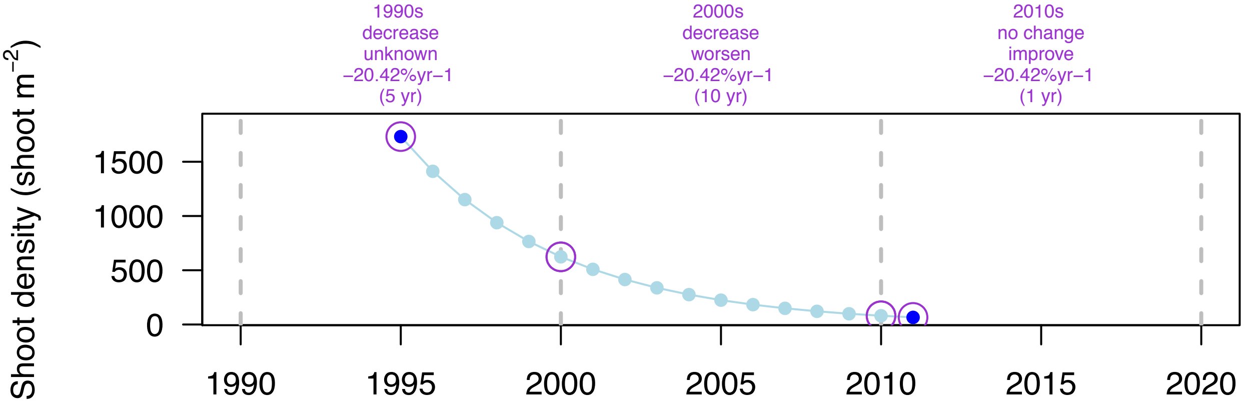

261_biomass	Tuya et al. 2013

SITE: Roque Arinaga (Spain – Atlantic) – Cn (–14 m)

OVERALL: Net = –879 g dw m–2;   Rate = –18.89 % yr–1; Perc Final = 5 % > decrease

DECADAL: YES (16 yr)

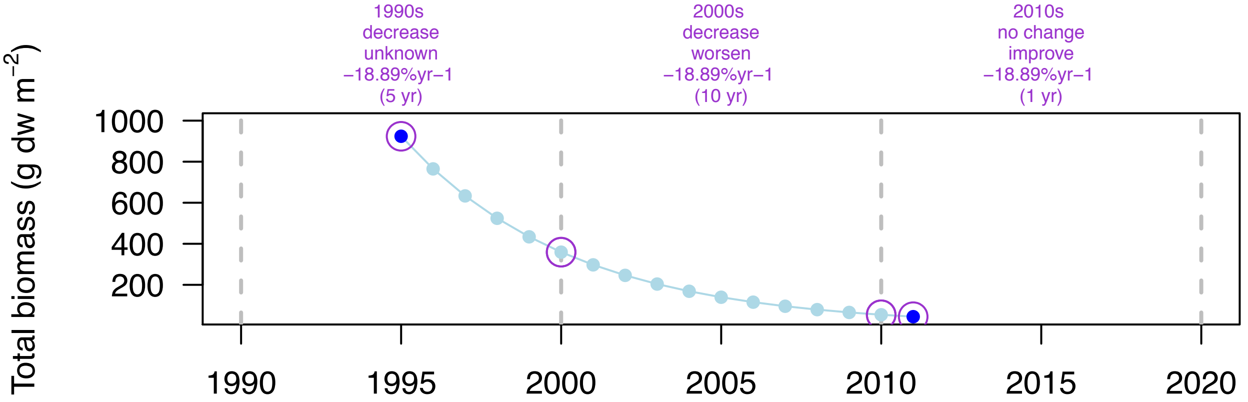

261_density

Tuya et al. 2013

SITE: Roque Arinaga (Spain – Atlantic) – Cn (–14 m)

OVERALL: Net = –1186 shoot m–2;   Rate = –15.52 % yr–1; Perc Final = 8 % > decrease

DECADAL: YES (16 yr)

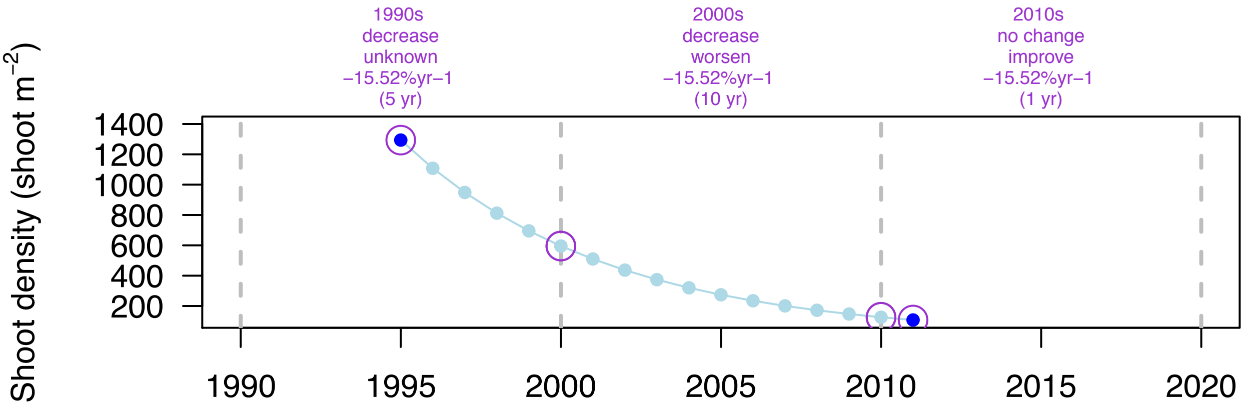

262_biomass

Tuya et al. 2013

SITE: Arinaga (Spain – Atlantic) – Cn (–5 m)

OVERALL: Net = –612.5 g dw m–2;   Rate = NA % yr–1; Perc Final = NA % > decrease

DECADAL: YES (16 yr)

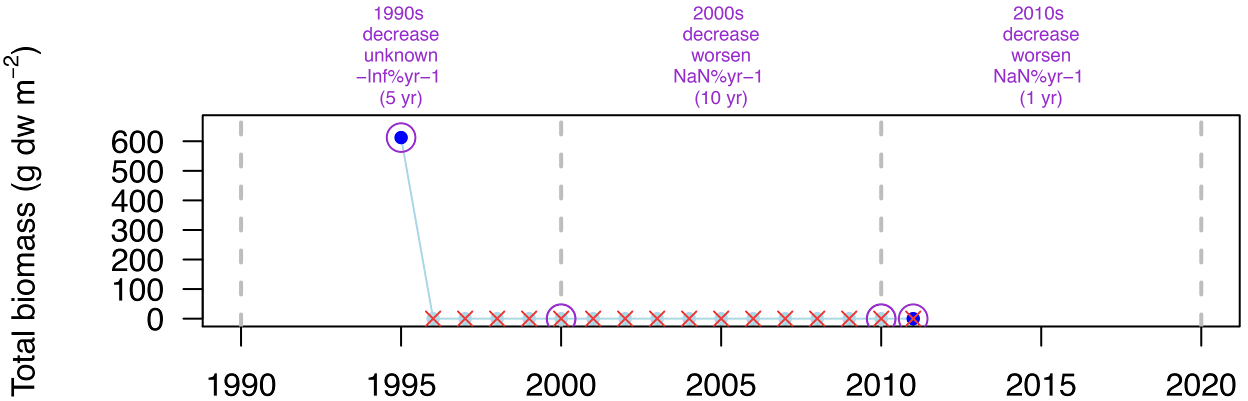

262_density

Tuya et al. 2013

SITE: Arinaga (Spain – Atlantic) – Cn (–5 m)

OVERALL: Net = –3078 shoot m–2; Rate = NA % yr–1; Perc Final = NA % > decrease

DECADAL: YES (16 yr)

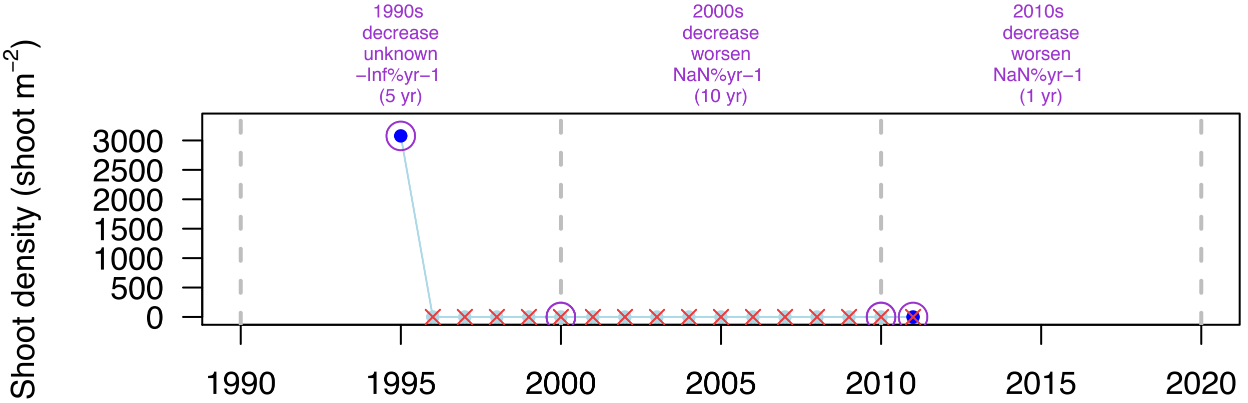

263_biomass

Tuya et al. 2013

SITE: Gando (Spain – Atlantic) – Cn (–4 m)

OVERALL: Net = –337.3 g dw m–2; Rate = –15.77 % yr–1; Perc Final = 8 % > decrease

DECADAL: YES (16 yr)

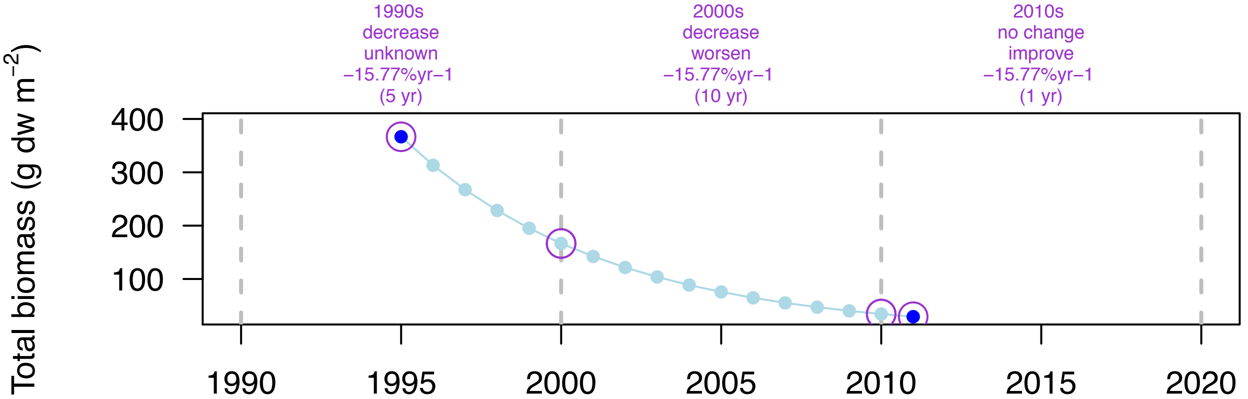

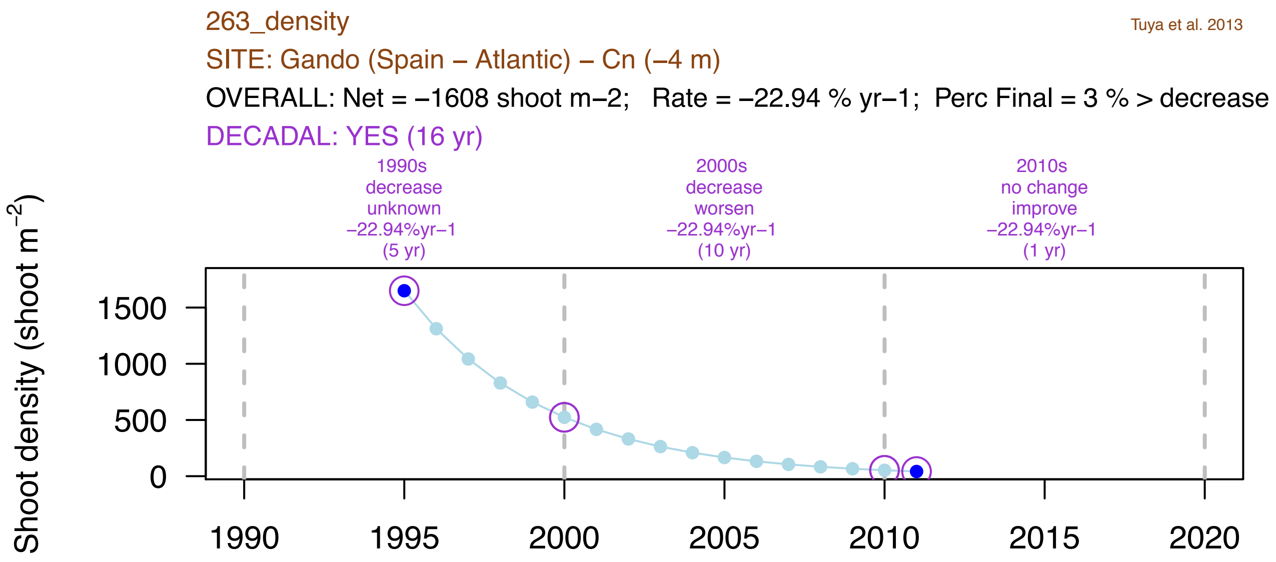
263_density
Tuya et al. 2013
SITE: Gando (Spain – Atlantic) – Cn (–4 m)
OVERALL: Net = –1608 shoot m–2; Rate = –22.94 % yr–1; Perc Final = 3 % > decrease
DECADAL: YES (16 yr)
1990s decrease unknown –22.94%yr–1 (5 yr)
2000s decrease worsen –22.94%yr–1 (10 yr)
2010s no change improve –22.94%yr–1 (1 yr)
Shoot density (shoot m^{-2})
1500
1000
500
0
1990 1995 2000 2005 2010 2015 2020

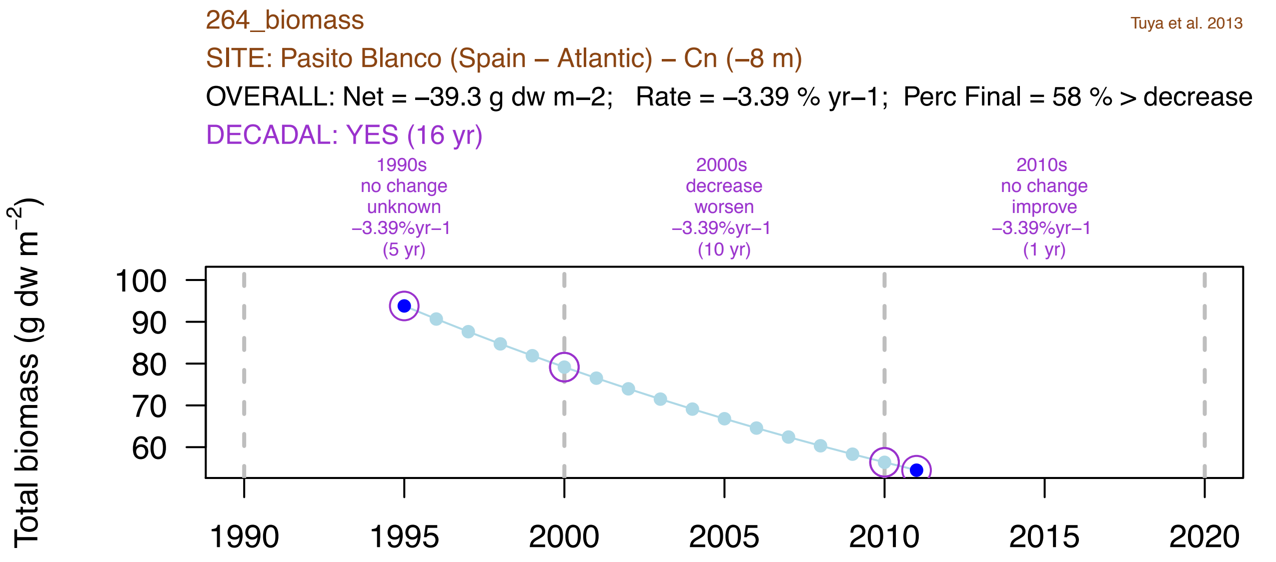
264_biomass
Tuya et al. 2013
SITE: Pasito Blanco (Spain – Atlantic) – Cn (–8 m)
OVERALL: Net = –39.3 g dw m–2; Rate = –3.39 % yr–1; Perc Final = 58 % > decrease
DECADAL: YES (16 yr)
1990s no change unknown –3.39%yr–1 (5 yr)
2000s decrease worsen –3.39%yr–1 (10 yr)
2010s no change improve –3.39%yr–1 (1 yr)
Total biomass (g dw m^{-2})
100
90
80
70
60
1990 1995 2000 2005 2010 2015 2020

264_density

Tuya et al. 2013

SITE: Pasito Blanco (Spain – Atlantic) – Cn (–8 m)

OVERALL: Net = –775 shoot m–2;   Rate = –14.74 % yr–1; Perc Final = 9 % > decrease

DECADAL: YES (16 yr)

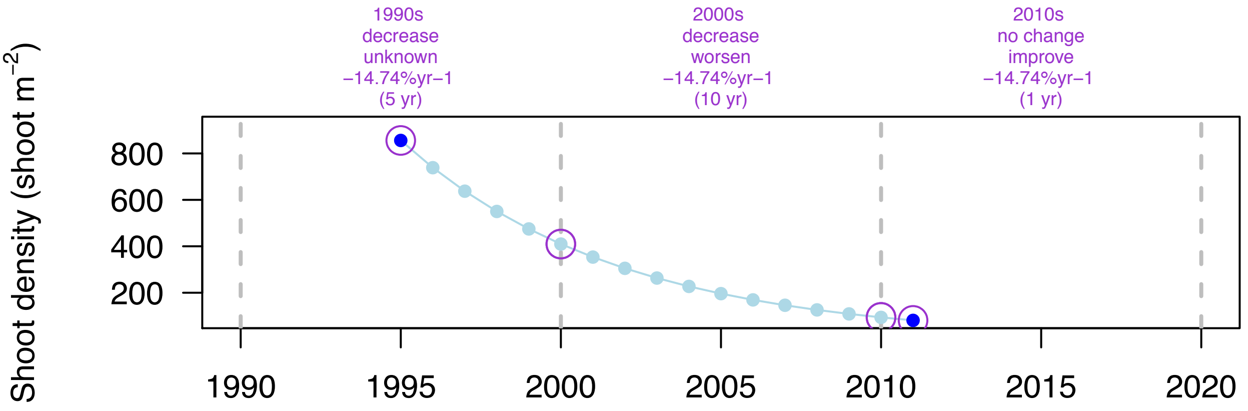

265_area

Gil–Rodríguez et al. 2012

SITE: Marina del Arrecife (Spain – Atlantic) – Zn (0 m)

OVERALL: Net = 0.01 ha;   Rate = 6.93 % yr–1; Perc Final = 200 % > increase

DECADAL: YES (10 yr)

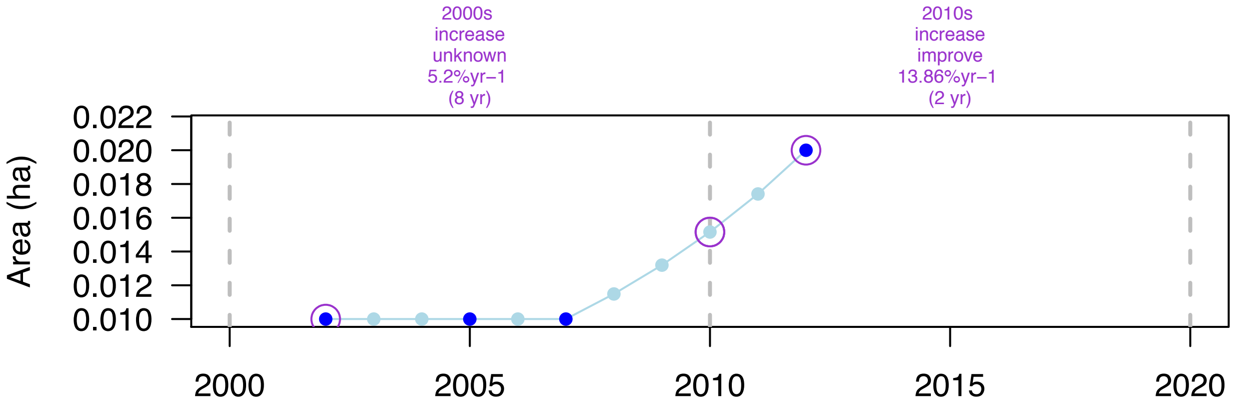

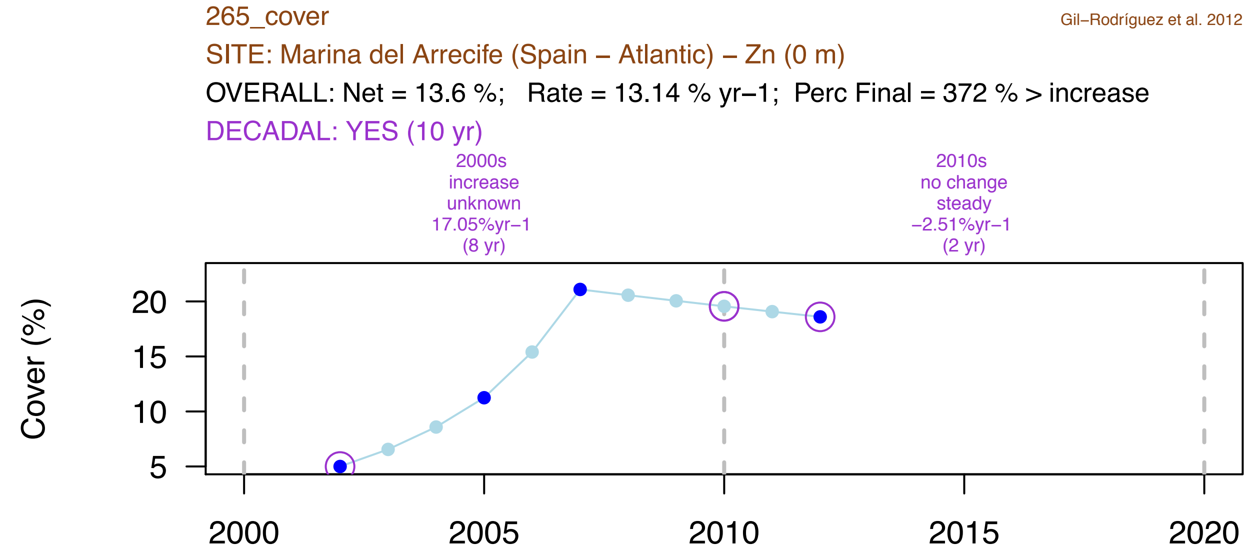
265_cover
Gil–Rodríguez et al. 2012
SITE: Marina del Arrecife (Spain – Atlantic) – Zn (0 m)
OVERALL: Net = 13.6 %; Rate = 13.14 % yr–1; Perc Final = 372 % > increase
DECADAL: YES (10 yr)
2000s
increase
unknown
17.05%yr–1
(8 yr)
2010s
no change
steady
–2.51%yr–1
(2 yr)
Cover (%)
20
15
10
5
2000
2005
2010
2015
2020

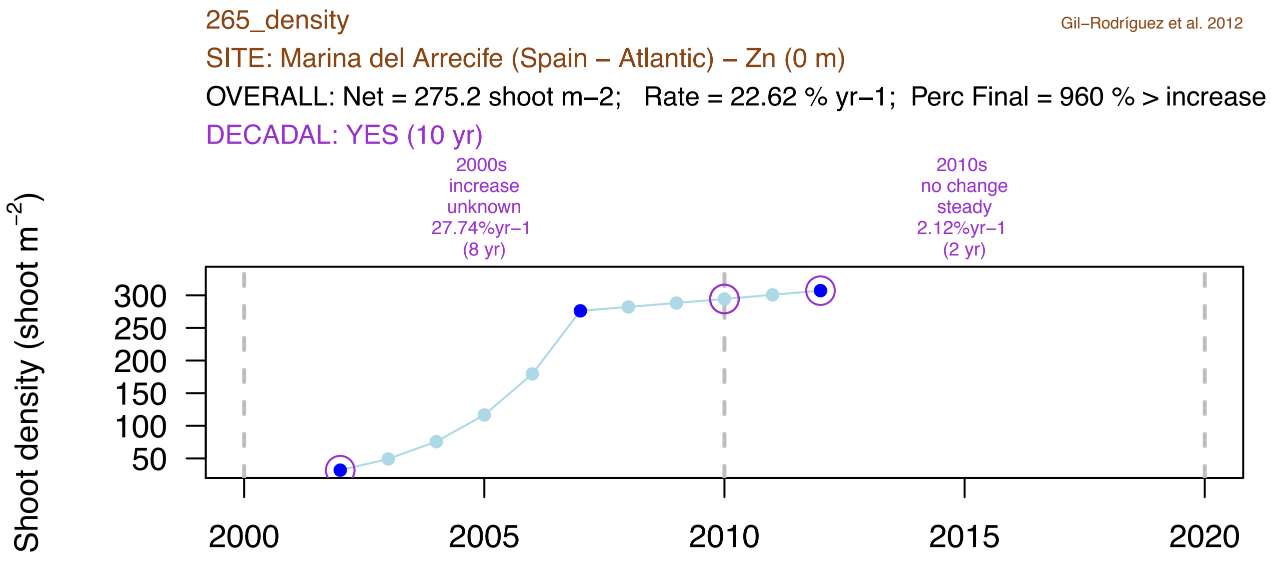
265_density
Gil–Rodríguez et al. 2012
SITE: Marina del Arrecife (Spain – Atlantic) – Zn (0 m)
OVERALL: Net = 275.2 shoot m–2; Rate = 22.62 % yr–1; Perc Final = 960 % > increase
DECADAL: YES (10 yr)
2000s
increase
unknown
27.74%yr–1
(8 yr)
2010s
no change
steady
2.12%yr–1
(2 yr)
Shoot density (shoot m^{-2})
300
250
200
150
100
50
2000
2005
2010
2015
2020

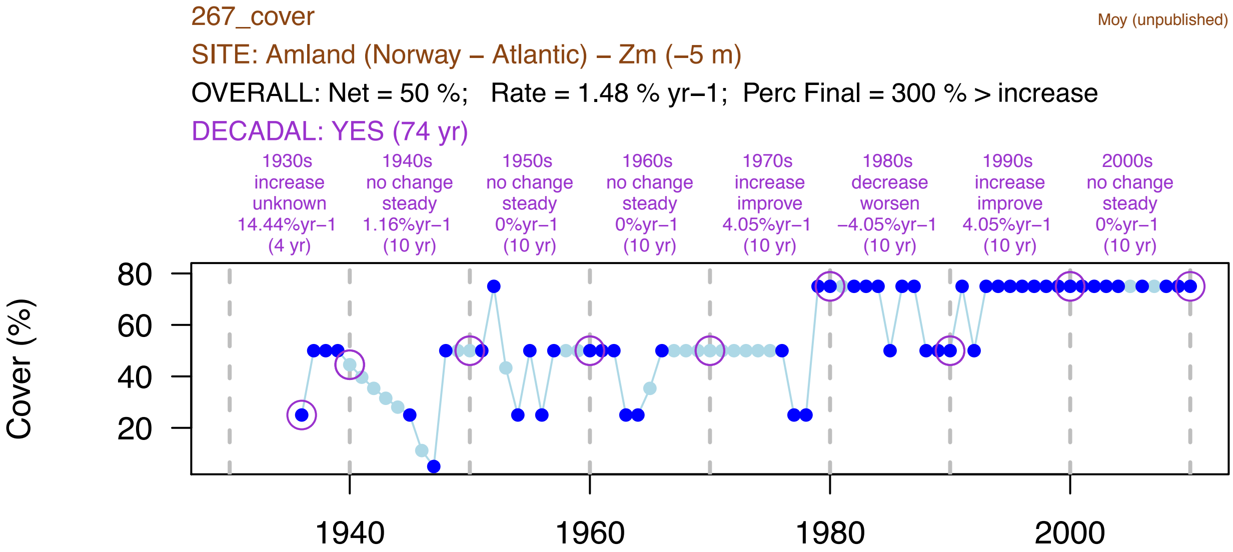
267_cover
Moy (unpublished)
SITE: Amland (Norway – Atlantic) – Zm (–5 m)
OVERALL: Net = 50 %; Rate = 1.48 % yr–1; Perc Final = 300 % > increase
DECADAL: YES (74 yr)
1930s increase unknown 14.44%yr–1 (4 yr)
1940s no change steady 1.16%yr–1 (10 yr)
1950s no change steady 0%yr–1 (10 yr)
1960s no change steady 0%yr–1 (10 yr)
1970s increase improve 4.05%yr–1 (10 yr)
1980s decrease worsen –4.05%yr–1 (10 yr)
1990s increase improve 4.05%yr–1 (10 yr)
2000s no change steady 0%yr–1 (10 yr)
Cover (%)
80
60
40
20
1940
1960
1980
2000

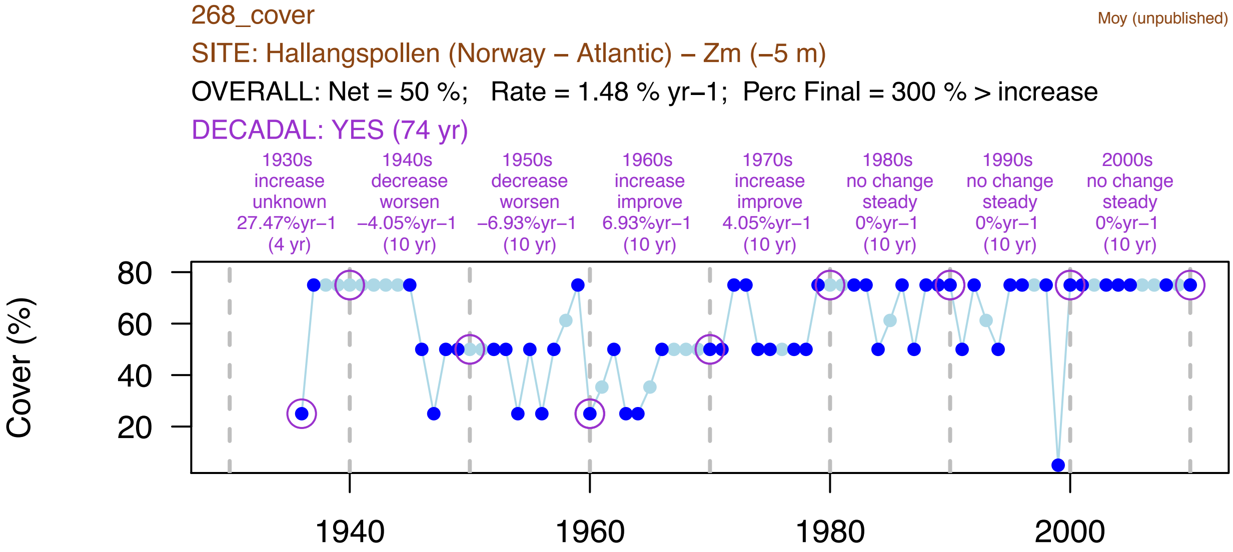
268_cover
Moy (unpublished)
SITE: Hallangspollen (Norway – Atlantic) – Zm (–5 m)
OVERALL: Net = 50 %; Rate = 1.48 % yr–1; Perc Final = 300 % > increase
DECADAL: YES (74 yr)
1930s increase unknown 27.47%yr–1 (4 yr)
1940s decrease worsen –4.05%yr–1 (10 yr)
1950s decrease worsen –6.93%yr–1 (10 yr)
1960s increase improve 6.93%yr–1 (10 yr)
1970s increase improve 4.05%yr–1 (10 yr)
1980s no change steady 0%yr–1 (10 yr)
1990s no change steady 0%yr–1 (10 yr)
2000s no change steady 0%yr–1 (10 yr)
Cover (%)
80
60
40
20
1940
1960
1980
2000

269_cover

Moy (unpublished)

SITE: Lusekilen (Norway – Atlantic) – Zm (–5 m)

OVERALL: Net = 25 %;   Rate = 0.94 % yr–1; Perc Final = 200 % > increase

DECADAL: YES (74 yr)

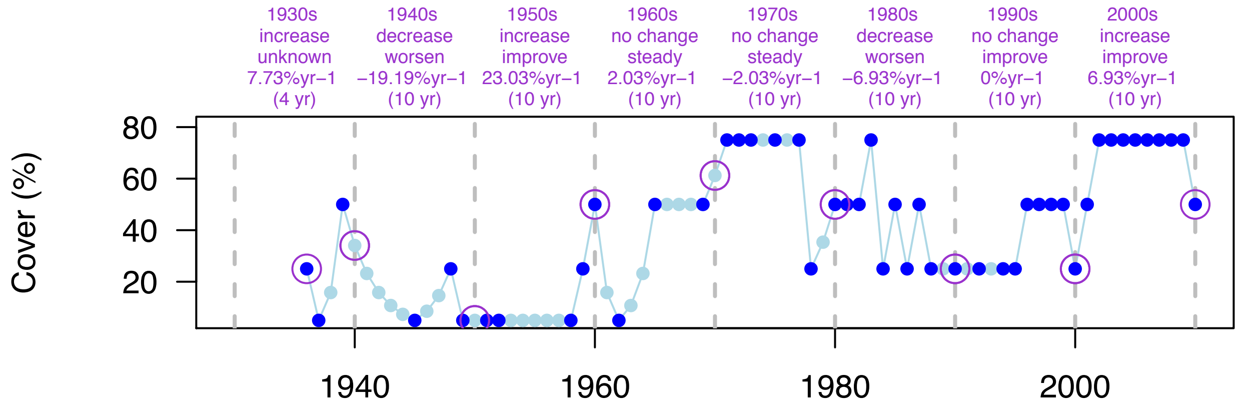

270_cover

Moy (unpublished)

SITE: Soppekilen (Norway – Atlantic) – Zm (–5 m)

OVERALL: Net = 70 %;   Rate = 3.66 % yr–1; Perc Final = 1500 % > increase

DECADAL: YES (74 yr)

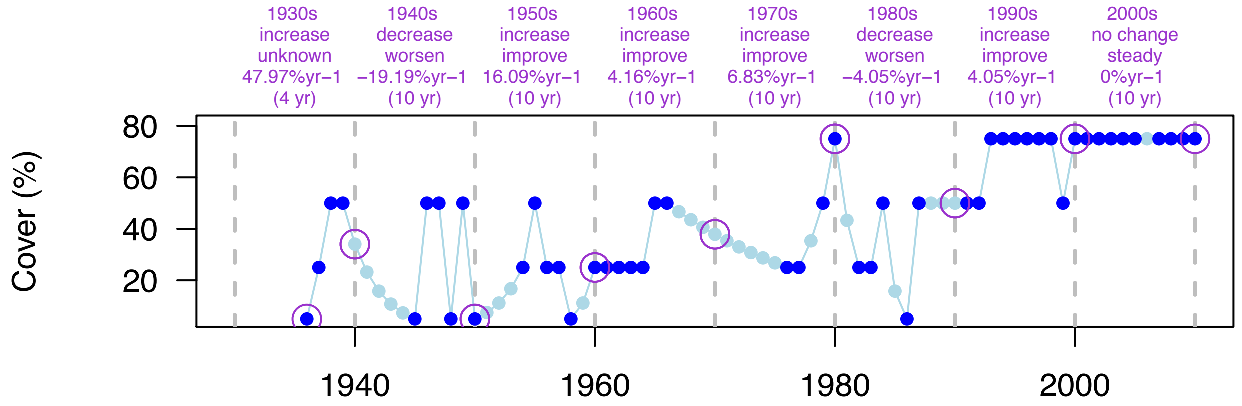

271_cover

Moy (unpublished)

SITE: Sundet (Norway – Atlantic) – Zm (–5 m)

OVERALL: Net = 45 %;   Rate = 3.11 % yr–1; Perc Final = 1000 % > increase

DECADAL: YES (74 yr)

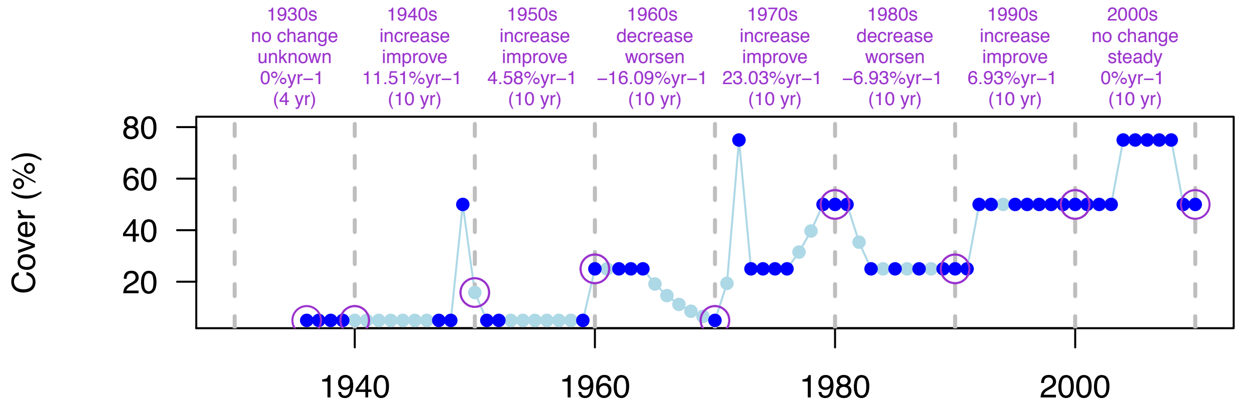

272_area

Wilkes et al. 2017

SITE: Ballysadare Estuary (Ireland – Atlantic) – Zn (? m)

OVERALL: Net = 5.91 ha;   Rate = 3.06 % yr–1; Perc Final = 117 % > increase

DECADAL: NO (5 yr)

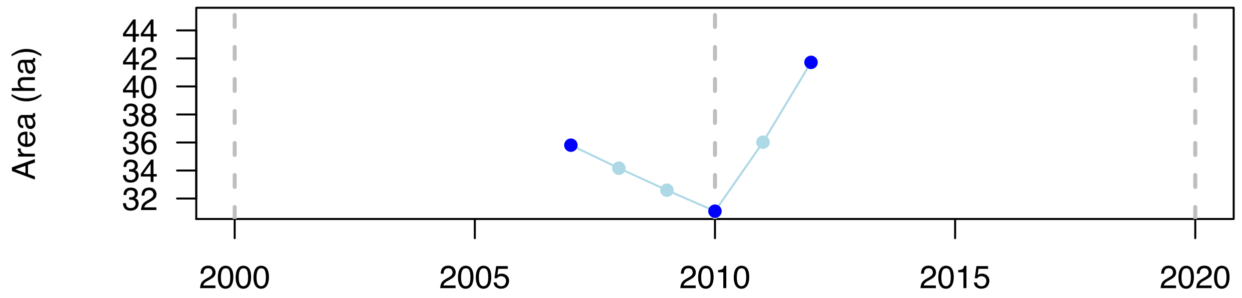

272_cover Wilkes et al. 2017

SITE: Ballysadare Estuary (Ireland – Atlantic) – Zn (? m)

OVERALL: Net = −7.53 %;   Rate = −2.78 % yr−1; Perc Final = 87 % > no change

DECADAL: NO (5 yr)

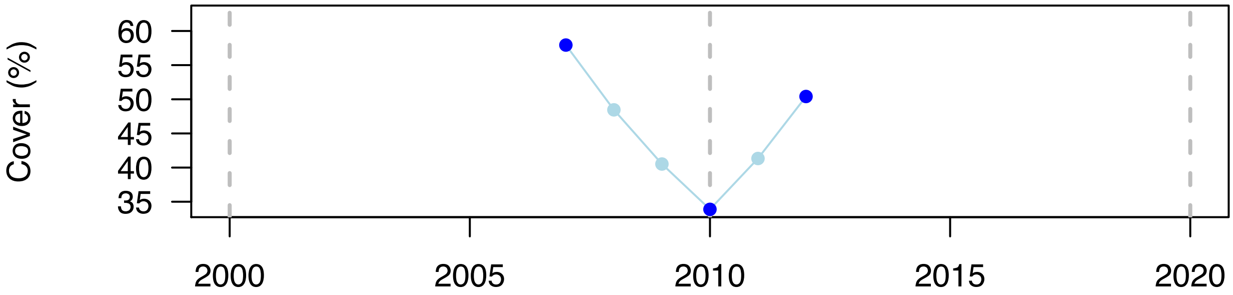

273_area Wilkes et al. 2017

SITE: Colligan Estuary (Ireland – Atlantic) – Zn (? m)

OVERALL: Net = 0.13 ha;   Rate = 2.84 % yr−1; Perc Final = 112 % > increase

DECADAL: NO (4 yr)

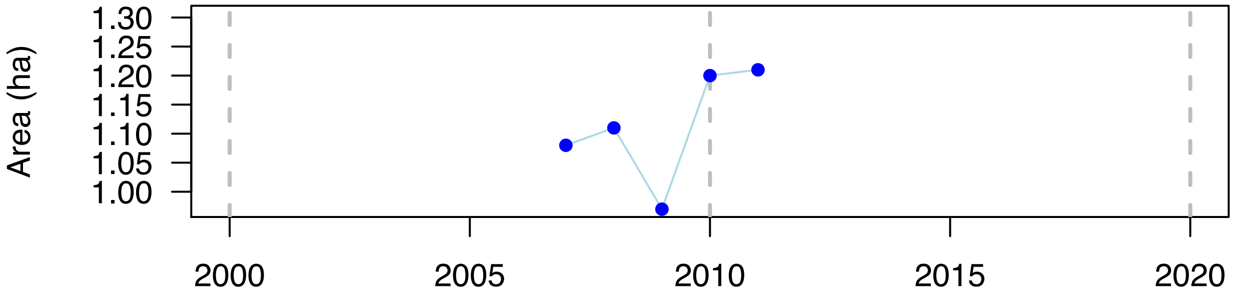

273_cover

Wilkes et al. 2017

SITE: Colligan Estuary (Ireland – Atlantic) – Zn (? m)

OVERALL: Net = 16.42 %;   Rate = 4.8 % yr–1; Perc Final = 121 % > no change

DECADAL: NO (4 yr)

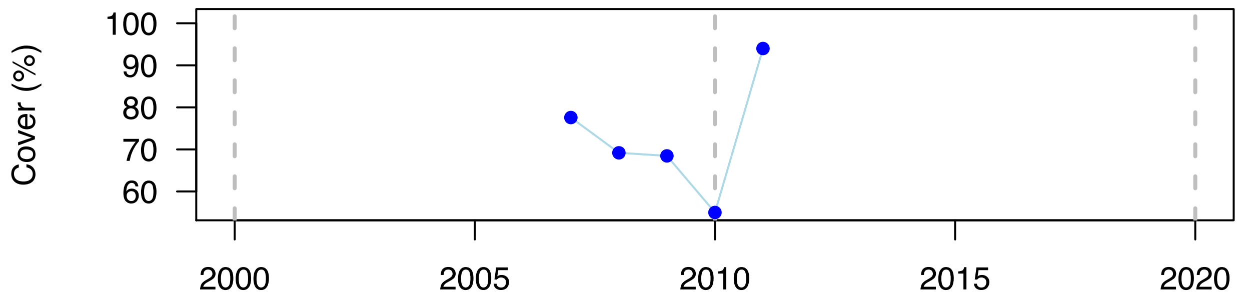

274_area

Wilkes et al. 2017

SITE: Cromane (Ireland – Atlantic) – Zn (? m)

OVERALL: Net = 7.16 ha;   Rate = 1.36 % yr–1; Perc Final = 104 % > no change

DECADAL: NO (3 yr)

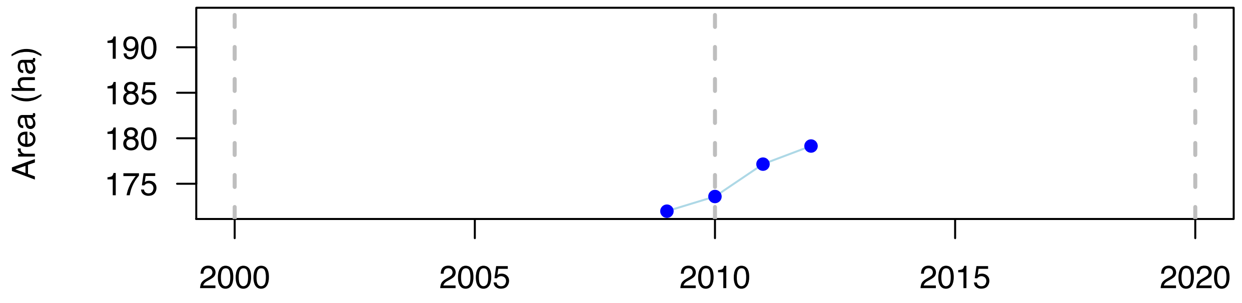

274_cover

Wilkes et al. 2017

SITE: Cromane (Ireland – Atlantic) – Zn (? m)

OVERALL: Net = 0.6 %;   Rate = 0.22 % yr–1; Perc Final = 101 % > no change

DECADAL: NO (3 yr)

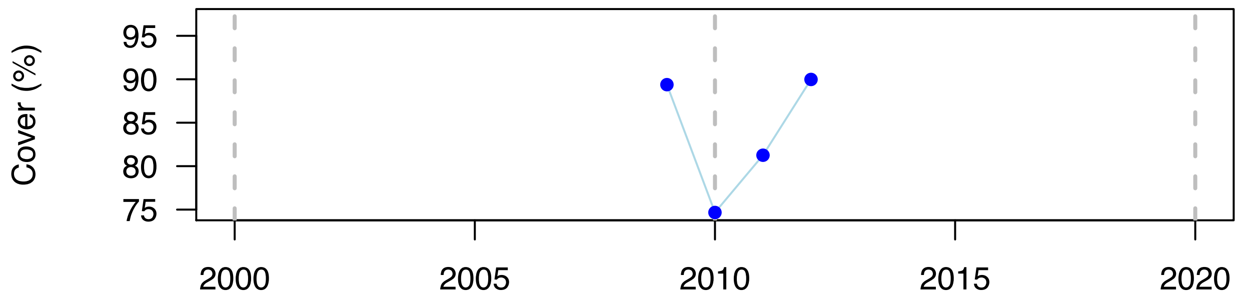

275_area

Wilkes et al. 2017

SITE: Dublin Bay (Ireland – Atlantic) – Zn (? m)

OVERALL: Net = 0.29 ha;   Rate = 3.45 % yr–1; Perc Final = 119 % > increase

DECADAL: NO (5 yr)

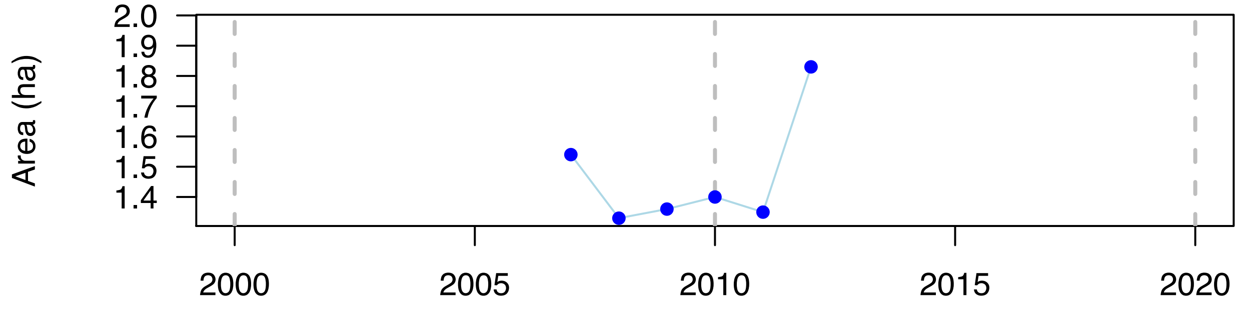

275_cover Wilkes et al. 2017

SITE: Dublin Bay (Ireland – Atlantic) – Zn (? m)

OVERALL: Net = −14.68 %; Rate = −4 % yr−1; Perc Final = 82 % > no change

DECADAL: NO (5 yr)

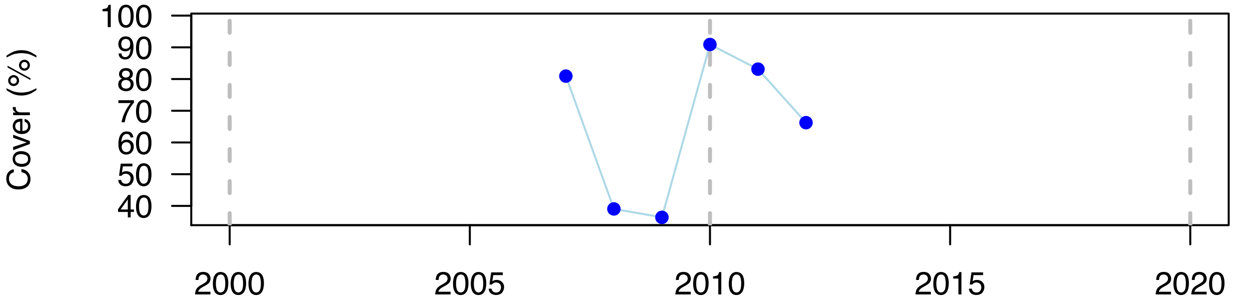

276_area Wilkes et al. 2017

SITE: Garavoge Estuary (Ireland – Atlantic) – Zn (? m)

OVERALL: Net = 1.08 ha; Rate = 3.69 % yr−1; Perc Final = 120 % > increase

DECADAL: NO (5 yr)

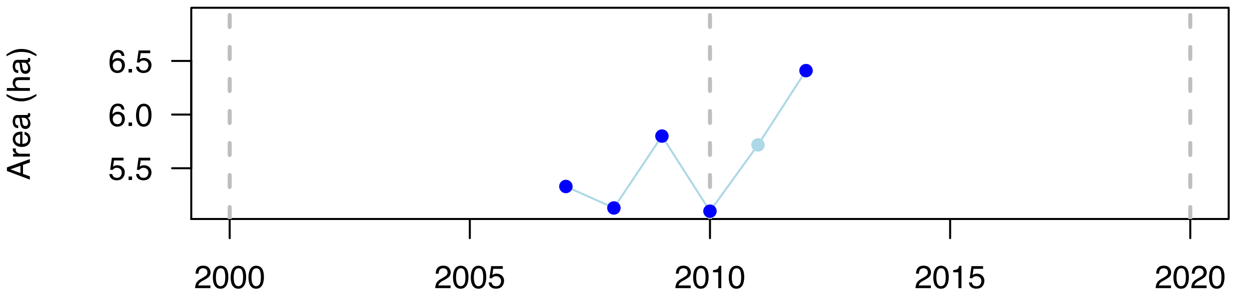

276_cover

Wilkes et al. 2017

SITE: Garavoge Estuary (Ireland – Atlantic) – Zn (? m)

OVERALL: Net = −16.76 %; Rate = −5.61 % yr−1; Perc Final = 76 % > no change

DECADAL: NO (5 yr)

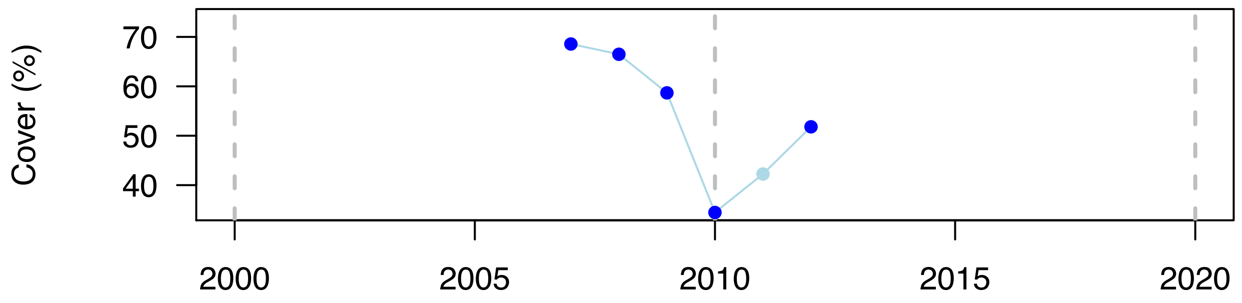

277_area

Wilkes et al. 2017

SITE: Malahide Bay (Ireland – Atlantic) – Zn (? m)

OVERALL: Net = 0.03 ha; Rate = 0.29 % yr−1; Perc Final = 101 % > no change

DECADAL: NO (3 yr)

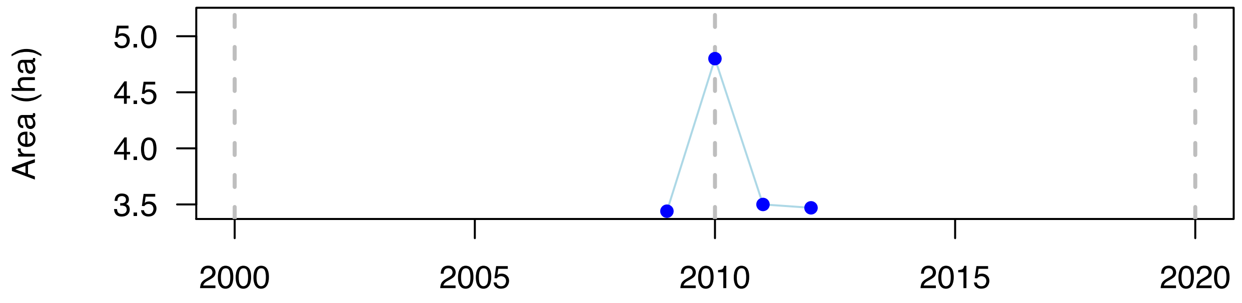

277_cover Wilkes et al. 2017

SITE: Malahide Bay (Ireland – Atlantic) – Zn (? m)

OVERALL: Net = −2.92 %; Rate = −1.58 % yr−1; Perc Final = 95 % > no change

DECADAL: NO (3 yr)

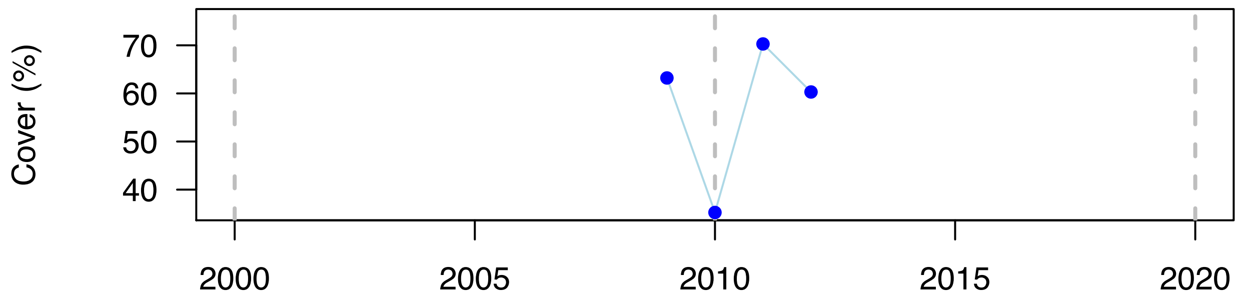

278_area Wilkes et al. 2017

SITE: Moy Estuary (Ireland – Atlantic) – Zn (? m)

OVERALL: Net = 1.67 ha; Rate = 2.41 % yr−1; Perc Final = 108 % > no change

DECADAL: NO (3 yr)

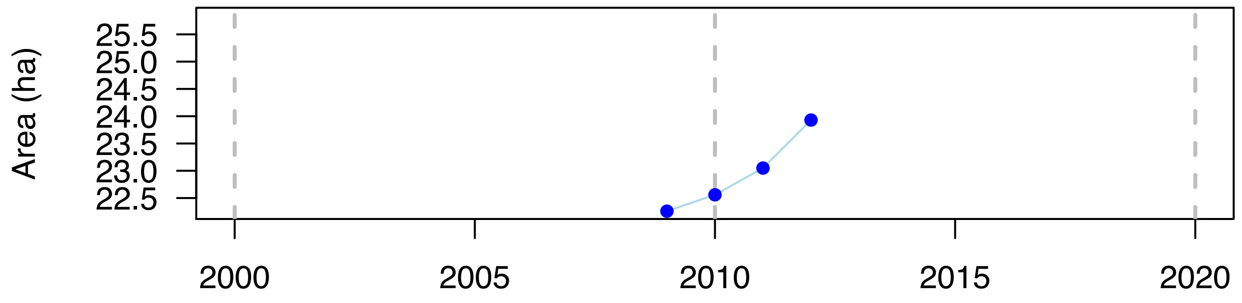

278_cover

Wilkes et al. 2017

SITE: Moy Estuary (Ireland – Atlantic) – Zn (? m)

OVERALL: Net = −21.86 %; Rate = −20.3 % yr−1; Perc Final = 54 % > decrease

DECADAL: NO (3 yr)

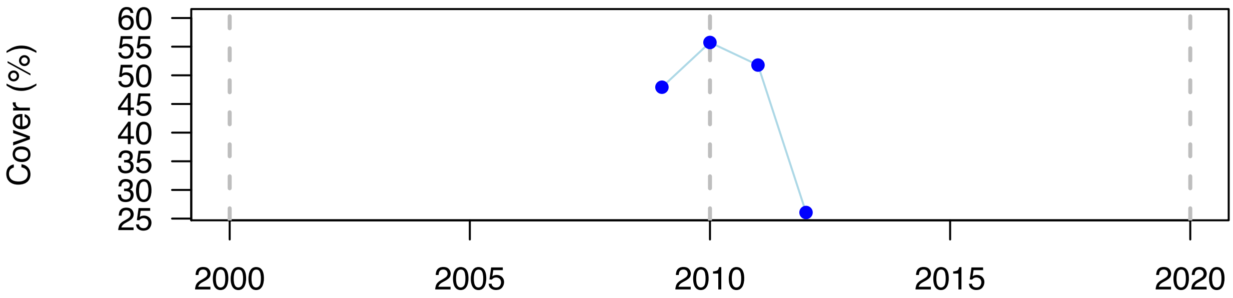

279_area

Wilkes et al. 2017

SITE: Tramore Back Strand (Ireland – Atlantic) – Zn (? m)

OVERALL: Net = 2.59 ha; Rate = 9.36 % yr−1; Perc Final = 145 % > increase

DECADAL: NO (4 yr)

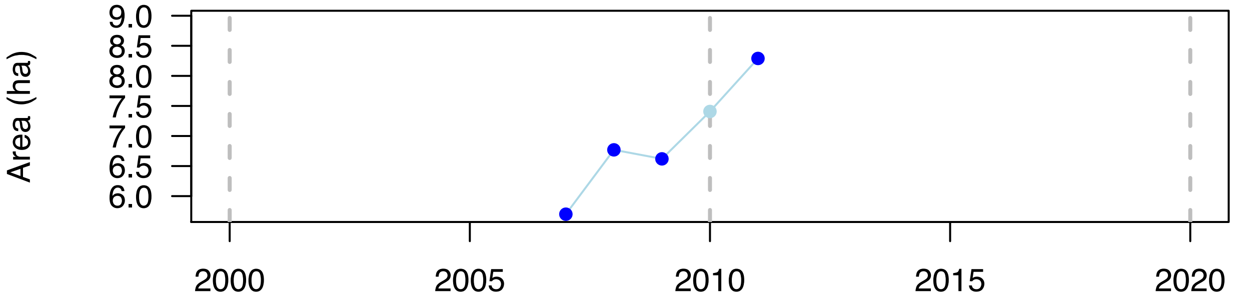

279_cover

Wilkes et al. 2017

SITE: Tramore Back Strand (Ireland – Atlantic) – Zn (? m)

OVERALL: Net = 28.73 %;   Rate = 12.2 % yr–1; Perc Final = 163 % > increase

DECADAL: NO (4 yr)

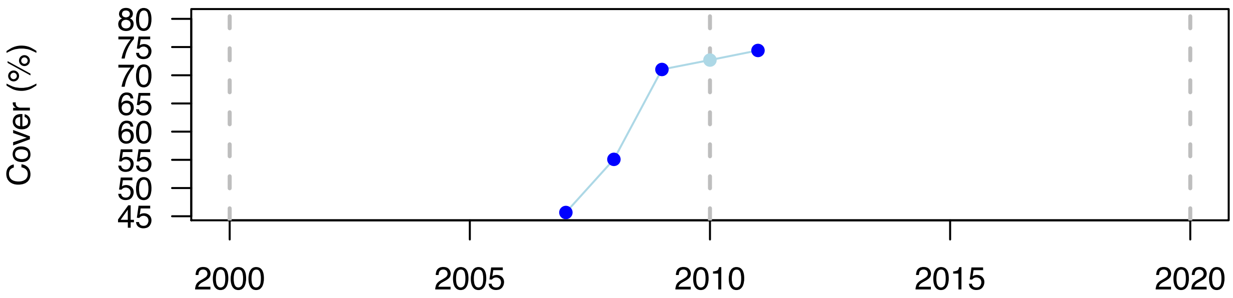

280_lowerlimit

Carstensen and Krause–Jensen 2012

SITE: Aabenraa Fjord (Denmark – Baltic) – Zm (? m)

OVERALL: Net = –0.71 m;   Rate = –0.78 % yr–1; Perc Final = 84 % > decrease

DECADAL: YES (22 yr)

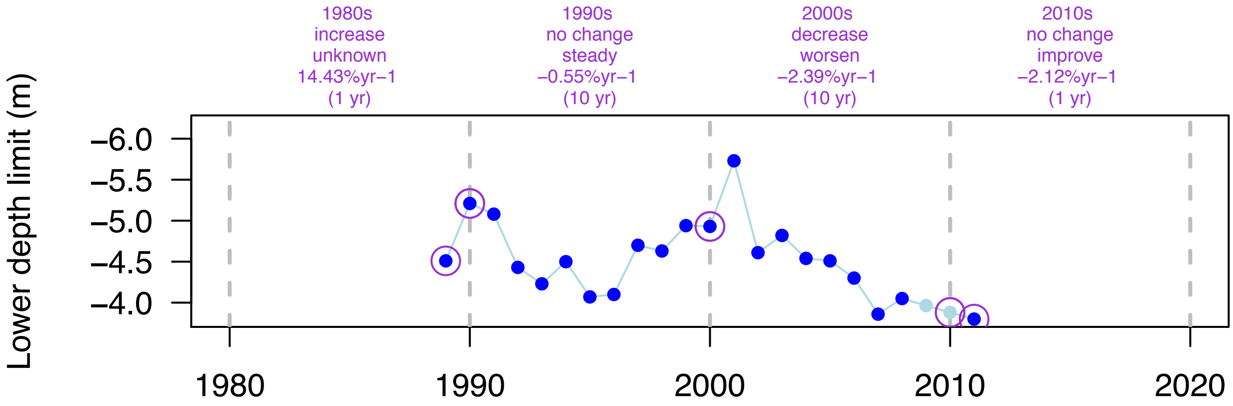

281_lowerlimit

Carstensen and Krause–Jensen 2012

SITE: Als Sund (Denmark – Baltic) – Zm (? m)

OVERALL: Net = –0.69 m;   Rate = –1.04 % yr–1; Perc Final = 80 % > decrease

DECADAL: YES (22 yr)

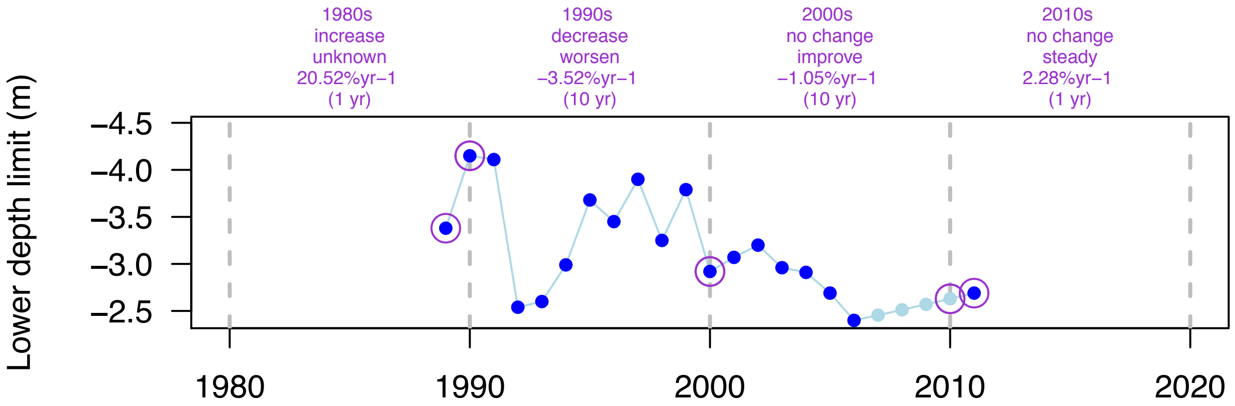

282_lowerlimit

Krause–Jensen and Rasmussen 2009, Carstensen and Krause–Jensen 2012

SITE: Århus Bugt (Denmark – Baltic) – Zm (? m)

OVERALL: Net = –4.87 m;   Rate = –0.6 % yr–1; Perc Final = 49 % > decrease

DECADAL: YES (120 yr)

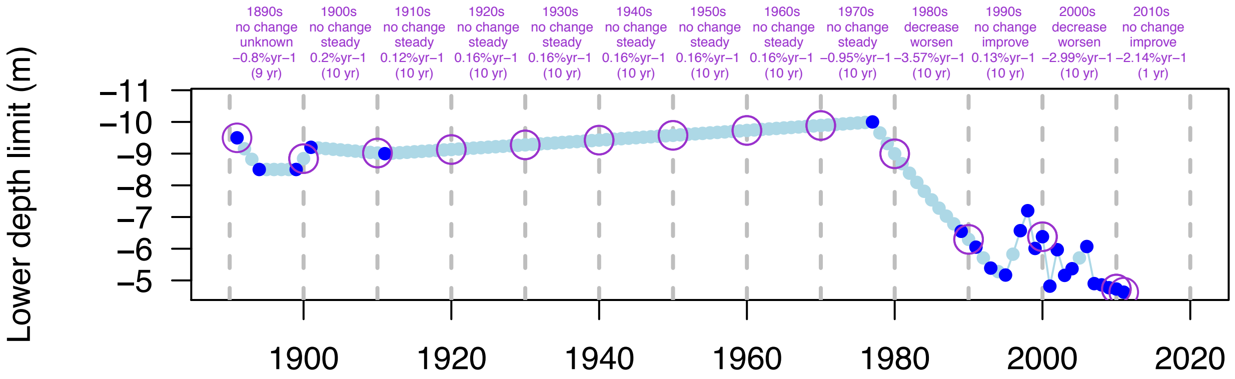

283_area

Vinther (unpublished)

SITE: Flensborg Fjord (Brunsnæs) (Denmark – Baltic) – Zm (? m)

OVERALL: Net = 21.77 ha; Rate = 3.33 % yr–1; Perc Final = 762 % > increase

DECADAL: YES (61 yr)

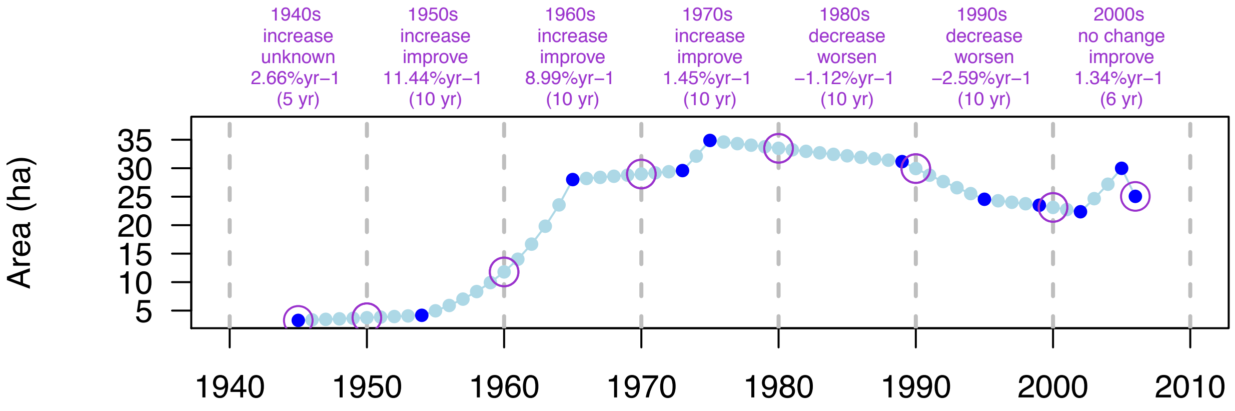

284_lowerlimit

Carstensen and Krause–Jensen 2012

SITE: Det Sydfynske Øhav (Denmark – Baltic) – Zm (? m)

OVERALL: Net = –1.5 m; Rate = –0.18 % yr–1; Perc Final = 80 % > decrease

DECADAL: YES (121 yr)

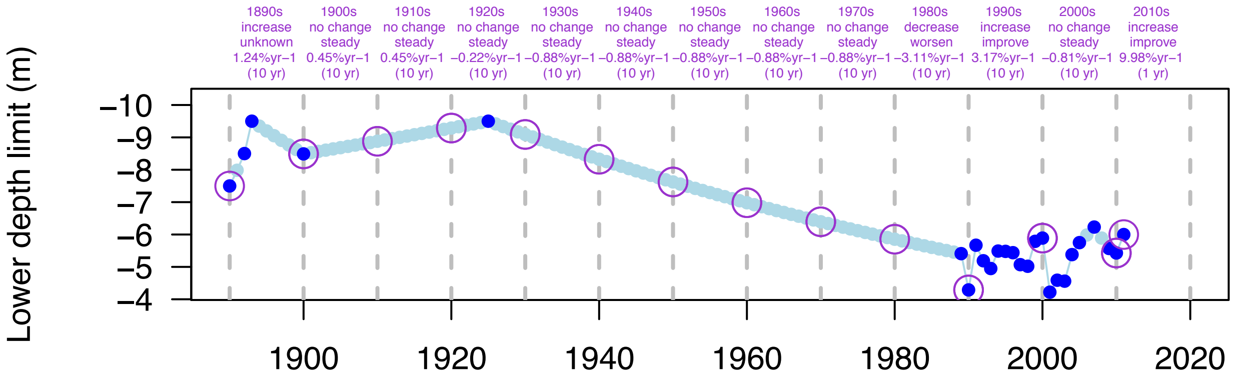

285_lowerlimit

Carstensen and Krause–Jensen 2012

SITE: Flensborg Fjord (Denmark – Baltic) – Zm (? m)

OVERALL: Net = –6.18 m;   Rate = –1.04 % yr–1; Perc Final = 39 % > decrease

DECADAL: YES (90 yr)

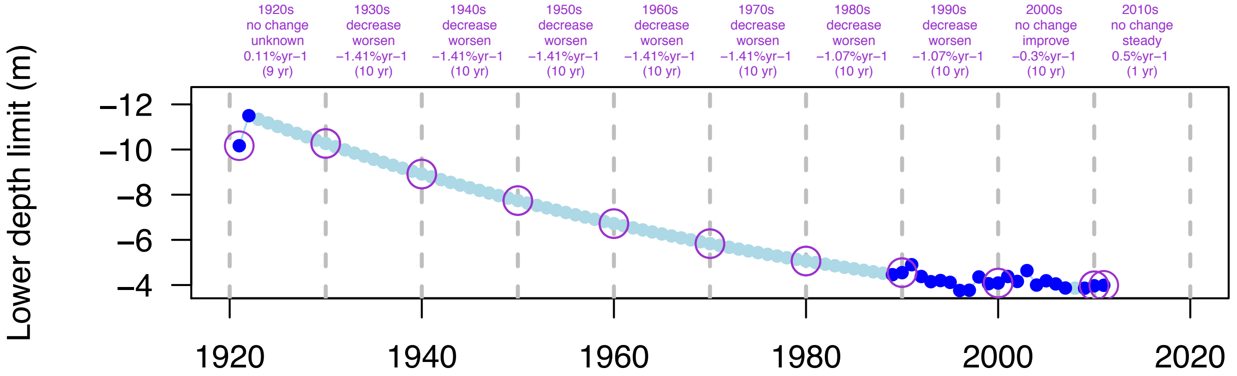

286_lowerlimit

Carstensen and Krause–Jensen 2012

SITE: Horsens Fjord (Denmark – Baltic) – Zm (? m)

OVERALL: Net = –7.51 m;   Rate = –1.25 % yr–1; Perc Final = 23 % > decrease

DECADAL: YES (118 yr)

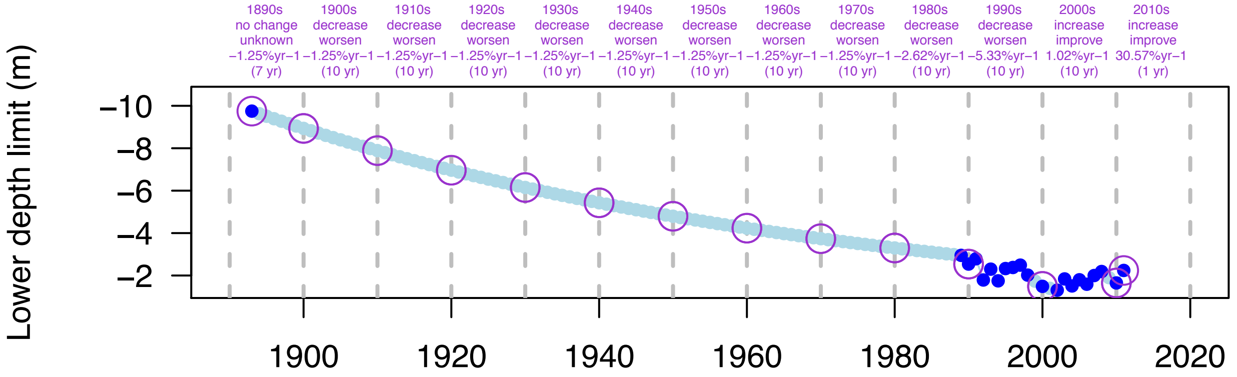

287_lowerlimit

Krause–Jensen and Rasmussen 2009, Carstensen and Krause–Jensen 2012

SITE: Isefjord (Inderbredning) (Denmark – Baltic) – Zm (? m)

OVERALL: Net = –1.38 m;   Rate = –0.37 % yr–1; Perc Final = 64 % > decrease

DECADAL: YES (121 yr)

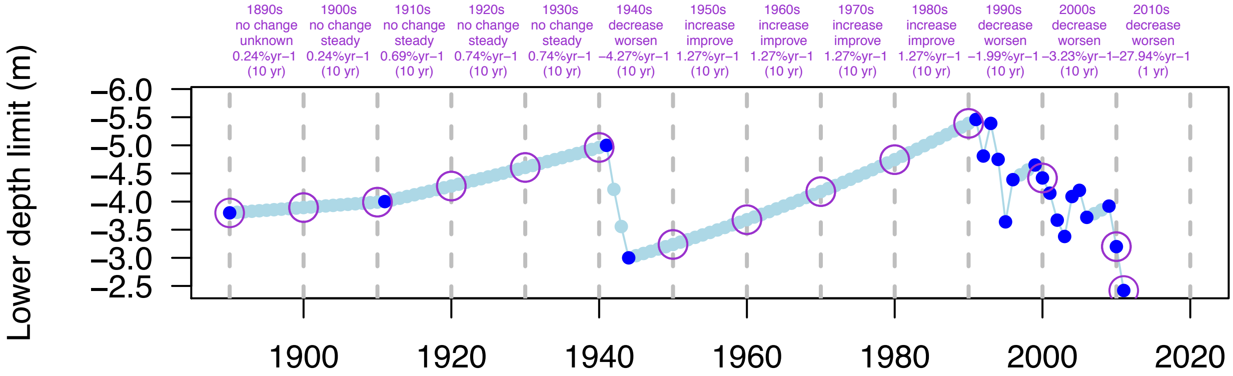

288_lowerlimit

Carstensen and Krause–Jensen 2012

SITE: Isefjord (Inderbredning) (Denmark – Baltic) – Zm (? m)

OVERALL: Net = –5.88 m;   Rate = –0.65 % yr–1; Perc Final = 47 % > decrease

DECADAL: YES (118 yr)

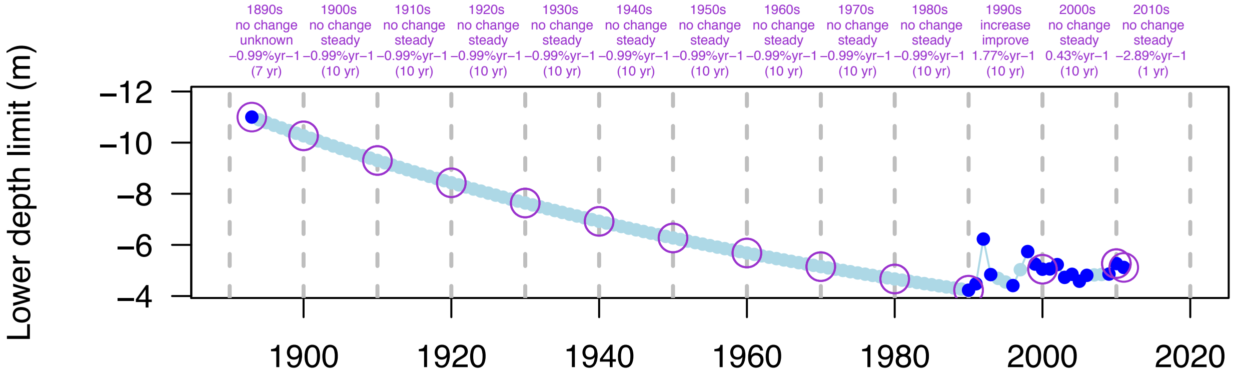

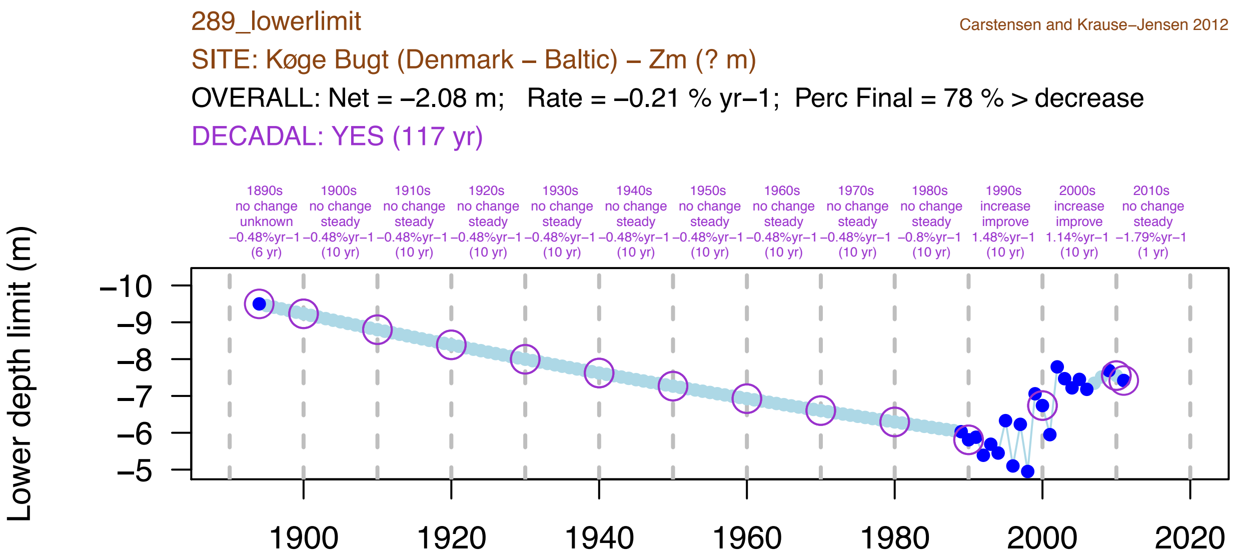
289_lowerlimit
Carstensen and Krause–Jensen 2012
SITE: Køge Bugt (Denmark – Baltic) – Zm (? m)
OVERALL: Net = –2.08 m; Rate = –0.21 % yr–1; Perc Final = 78 % > decrease
DECADAL: YES (117 yr)
1890s no change unknown –0.48%yr–1 (6 yr)
1900s no change steady –0.48%yr–1 (10 yr)
1910s no change steady –0.48%yr–1 (10 yr)
1920s no change steady –0.48%yr–1 (10 yr)
1930s no change steady –0.48%yr–1 (10 yr)
1940s no change steady –0.48%yr–1 (10 yr)
1950s no change steady –0.48%yr–1 (10 yr)
1960s no change steady –0.48%yr–1 (10 yr)
1970s no change steady –0.48%yr–1 (10 yr)
1980s no change steady –0.8%yr–1 (10 yr)
1990s increase improve 1.48%yr–1 (10 yr)
2000s increase improve 1.14%yr–1 (10 yr)
2010s no change steady –1.79%yr–1 (1 yr)
Lower depth limit (m)
–10
–9
–8
–7
–6
–5
1900
1920
1940
1960
1980
2000
2020

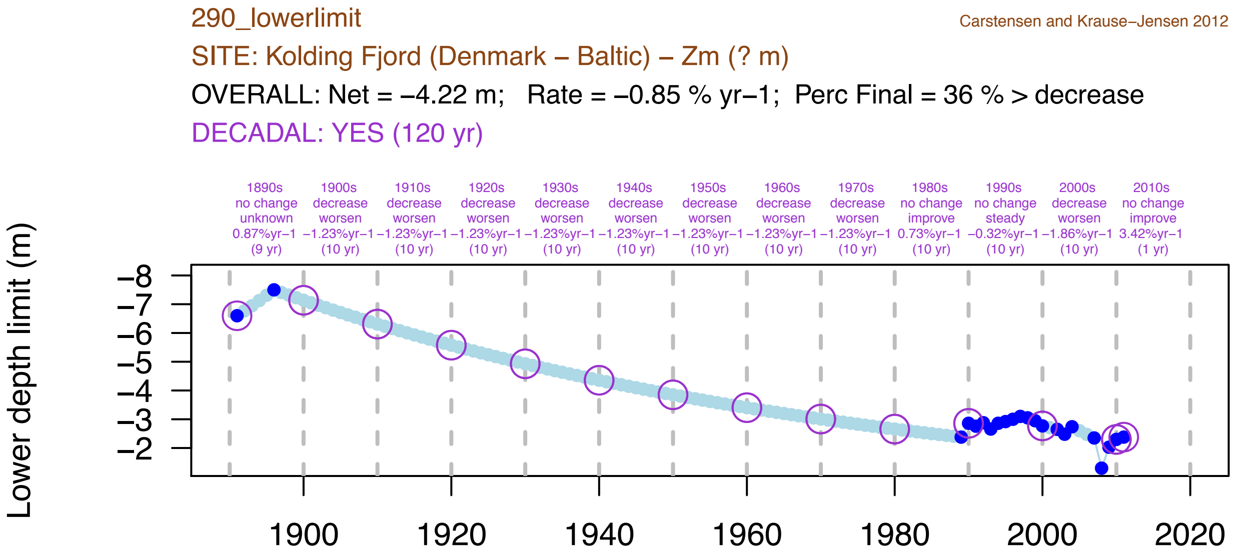
290_lowerlimit
Carstensen and Krause–Jensen 2012
SITE: Kolding Fjord (Denmark – Baltic) – Zm (? m)
OVERALL: Net = –4.22 m; Rate = –0.85 % yr–1; Perc Final = 36 % > decrease
DECADAL: YES (120 yr)
1890s no change unknown 0.87%yr–1 (9 yr)
1900s decrease worsen –1.23%yr–1 (10 yr)
1910s decrease worsen –1.23%yr–1 (10 yr)
1920s decrease worsen –1.23%yr–1 (10 yr)
1930s decrease worsen –1.23%yr–1 (10 yr)
1940s decrease worsen –1.23%yr–1 (10 yr)
1950s decrease worsen –1.23%yr–1 (10 yr)
1960s decrease worsen –1.23%yr–1 (10 yr)
1970s decrease worsen –1.23%yr–1 (10 yr)
1980s no change improve 0.73%yr–1 (10 yr)
1990s no change steady –0.32%yr–1 (10 yr)
2000s decrease worsen –1.86%yr–1 (10 yr)
2010s no change improve 3.42%yr–1 (1 yr)
Lower depth limit (m)
–8
–7
–6
–5
–4
–3
–2
1900
1920
1940
1960
1980
2000
2020

291_area

Frederiksen et al. 2004

SITE: Limfjorden (Boddum Vig) (Denmark – Baltic) – Zm (? m)

OVERALL: Net = 13.5 ha; Rate = 3.87 % yr–1; Perc Final = 550 % > increase

DECADAL: YES (44 yr)

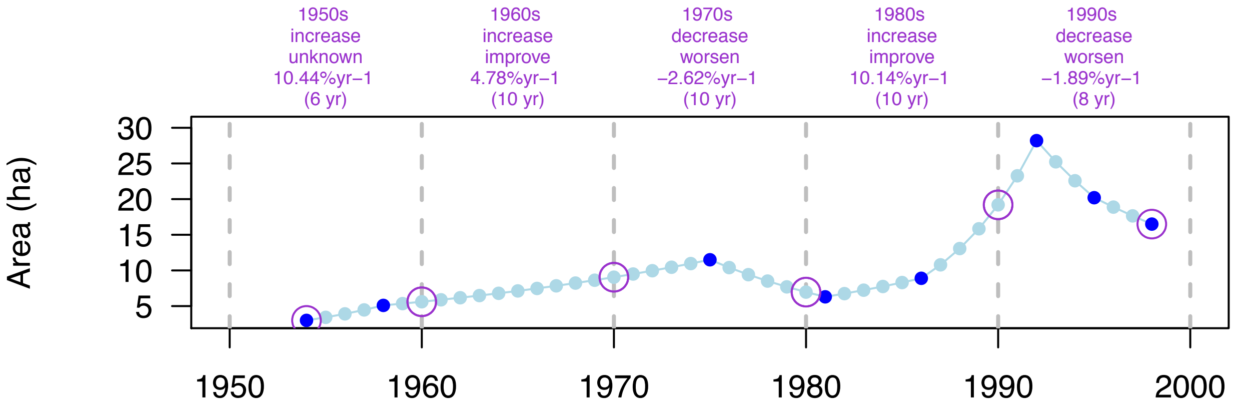

292_area

Frederiksen et al. 2004

SITE: Limfjorden (Holmstange) (Denmark – Baltic) – Zm (? m)

OVERALL: Net = 16 ha; Rate = 7.43 % yr–1; Perc Final = 4100 % > increase

DECADAL: YES (50 yr)

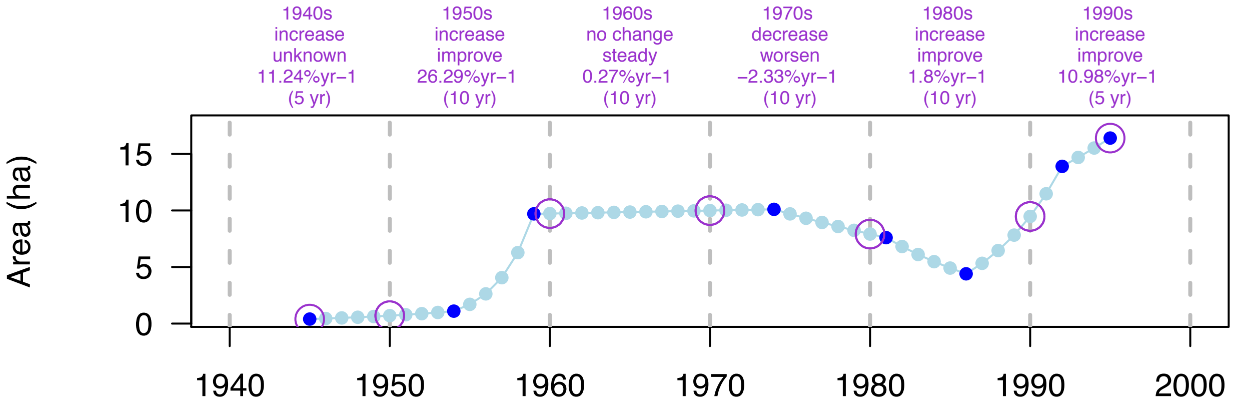

293_lowerlimit

Krause–Jensen and Rasmussen 2009, Carstensen and Krause–Jensen 2012

SITE: Løgstør Bredning (Denmark – Baltic) – Zm (? m)

OVERALL: Net = –2.55 m;   Rate = –0.54 % yr–1; Perc Final = 53 % > decrease

DECADAL: YES (118 yr)

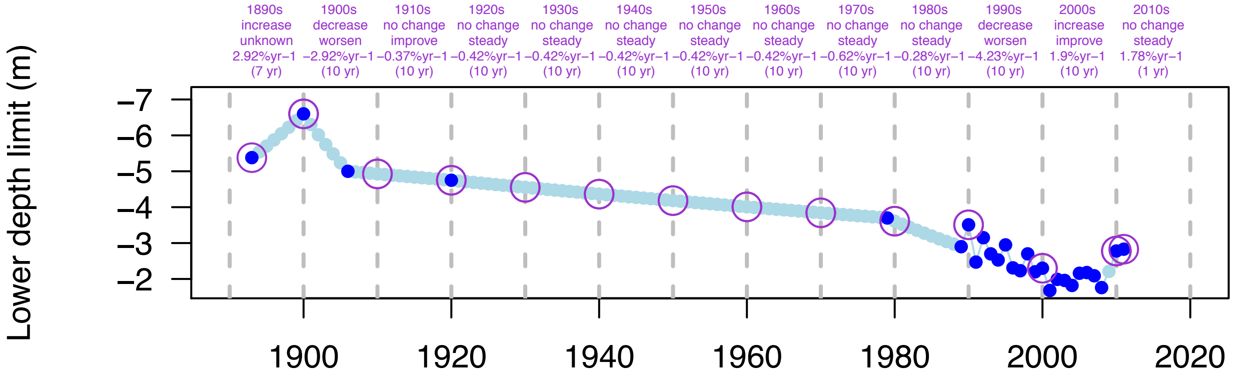

294_area

Laursen (unpublished)

SITE: Mariager Fjord (Denmark – Baltic) – Zm (? m)

OVERALL: Net = 198.7 ha;   Rate = 2.3 % yr–1; Perc Final = 186 % > increase

DECADAL: YES (27 yr)

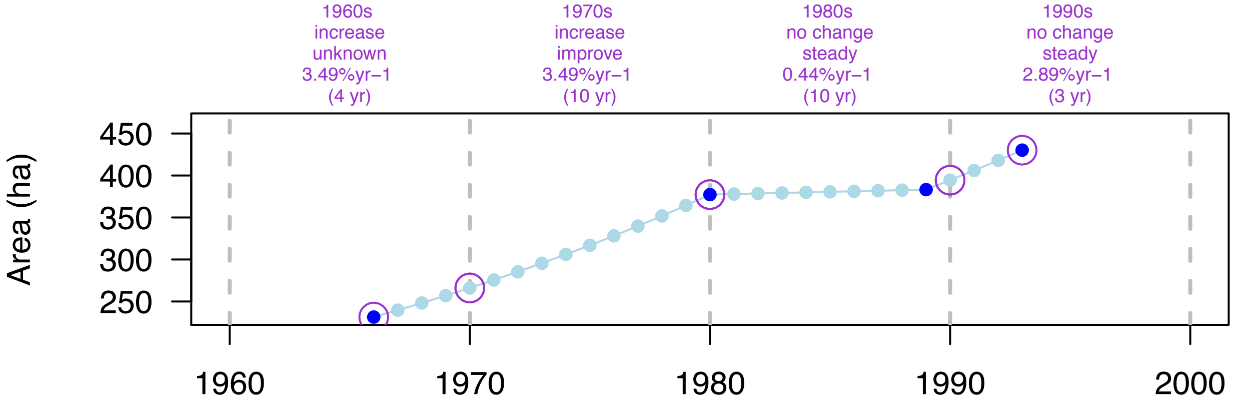

295_lowerlimit

Krause–Jensen and Rasmussen 2009, Carstensen and Krause–Jensen 2012

SITE: Nibe Gjøl Bredning (Denmark – Baltic) – Zm (? m)

OVERALL: Net = −2.06 m;   Rate = −0.52 % yr−1; Perc Final = 56 % > decrease

DECADAL: YES (111 yr)

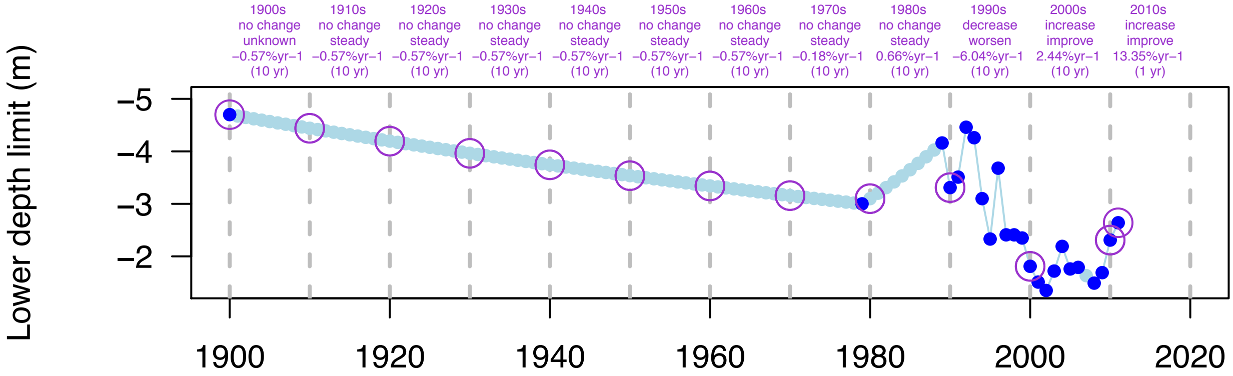

296_area

Josefson et al. 2009, Carstensen and Krause–Jensen 2012

SITE: Odense Fjord (Denmark – Baltic) – Zm (? m)

OVERALL: Net = −2490 ha;   Rate = −2.67 % yr−1; Perc Final = 6 % > decrease

DECADAL: YES (105 yr)

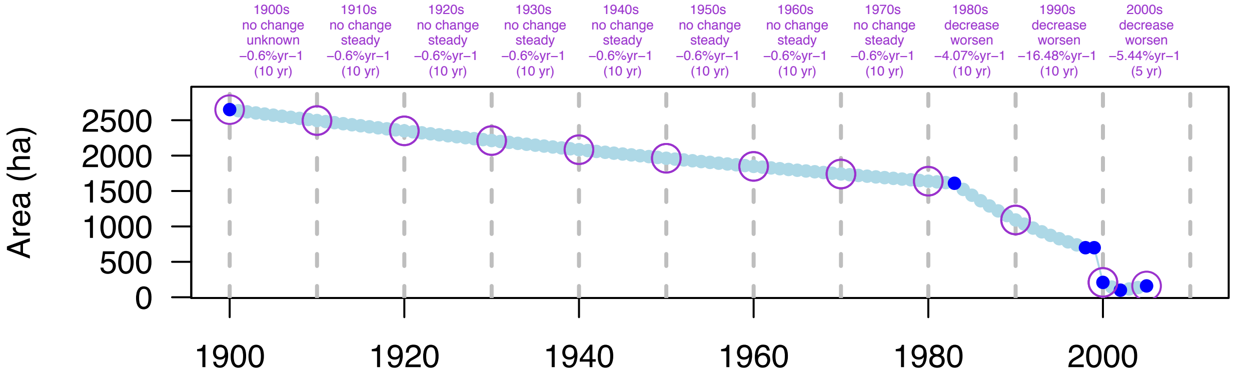

296_lowerlimit

Josefson et al. 2009, Carstensen and Krause–Jensen 2012

SITE: Odense Fjord (Denmark – Baltic) – Zm (? m)

OVERALL: Net = –3.51 m; Rate = –0.89 % yr–1; Perc Final = 37 % > decrease

DECADAL: YES (111 yr)

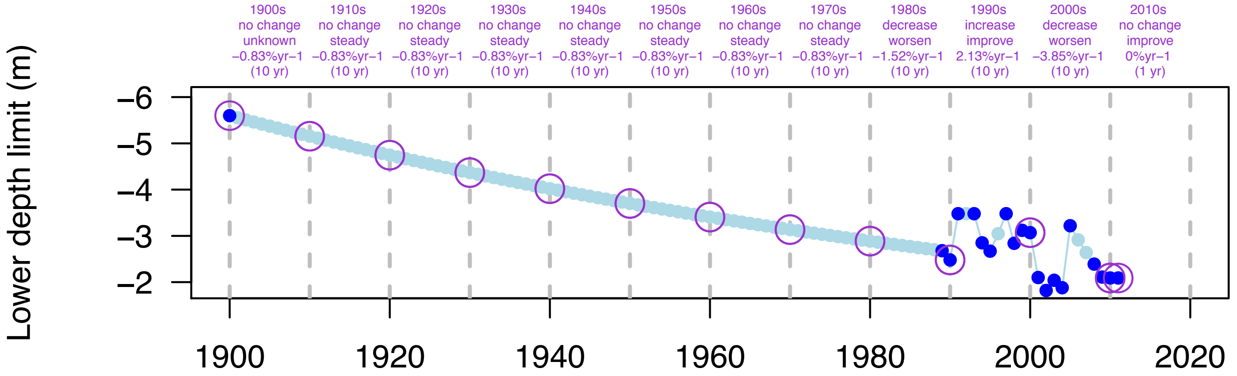

297_lowerlimit

Krause–Jensen and Rasmussen 2009, Carstensen and Krause–Jensen 2012

SITE: Øresund (entire) (Denmark – Baltic) – Zm (? m)

OVERALL: Net = –1.47 m; Rate = –0.17 % yr–1; Perc Final = 82 % > decrease

DECADAL: YES (117 yr)

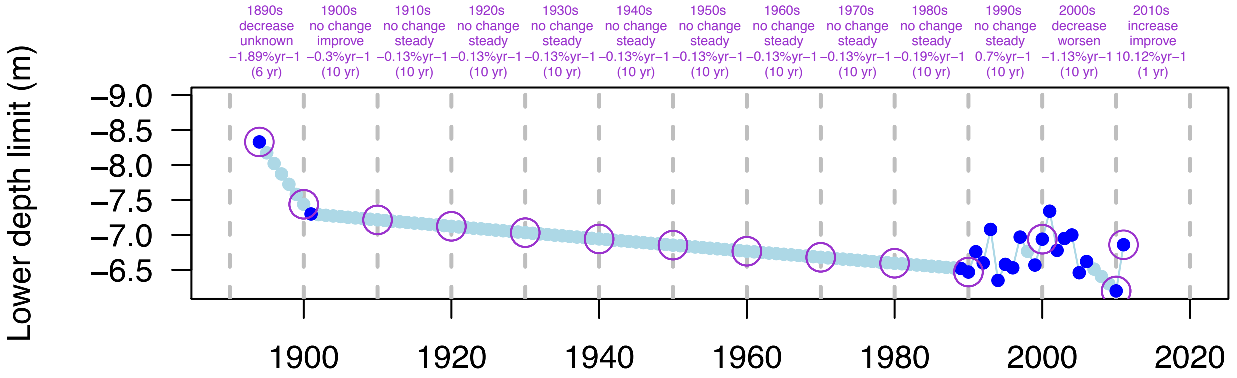

298_area

Frederiksen et al. 2004

SITE: Øresund (Amager) (Denmark – Baltic) – Zm (? m)

OVERALL: Net = –44.7 ha;   Rate = –0.95 % yr–1; Perc Final = 68 % > decrease

DECADAL: YES (41 yr)

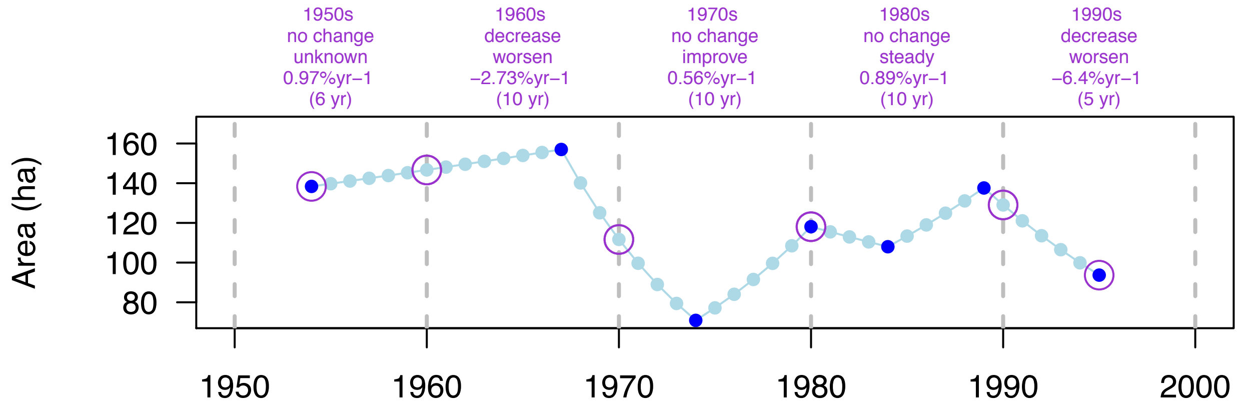

299_abiomass

Krause–Jensen et al. 2000

SITE: Saltholm (control) (Denmark – Baltic) – Zm (? m)

OVERALL: Net = 4 g dw m–2;   Rate = 0.78 % yr–1; Perc Final = 106 % > no change

DECADAL: NO (7 yr)

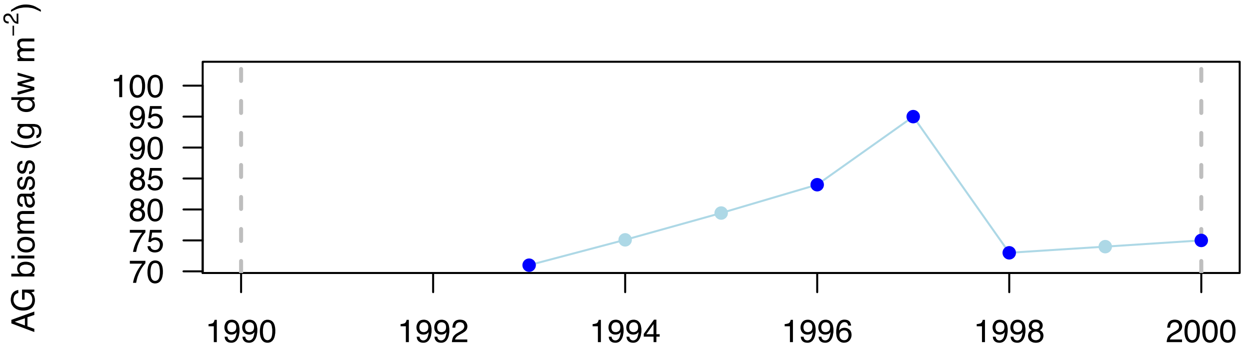

299_density

Krause−Jensen et al. 2000

SITE: Saltholm (control) (Denmark – Baltic) – Zm (? m)

OVERALL: Net = 119 shoot m−2;   Rate = 5.44 % yr−1; Perc Final = 146 % > increase

DECADAL: NO (7 yr)

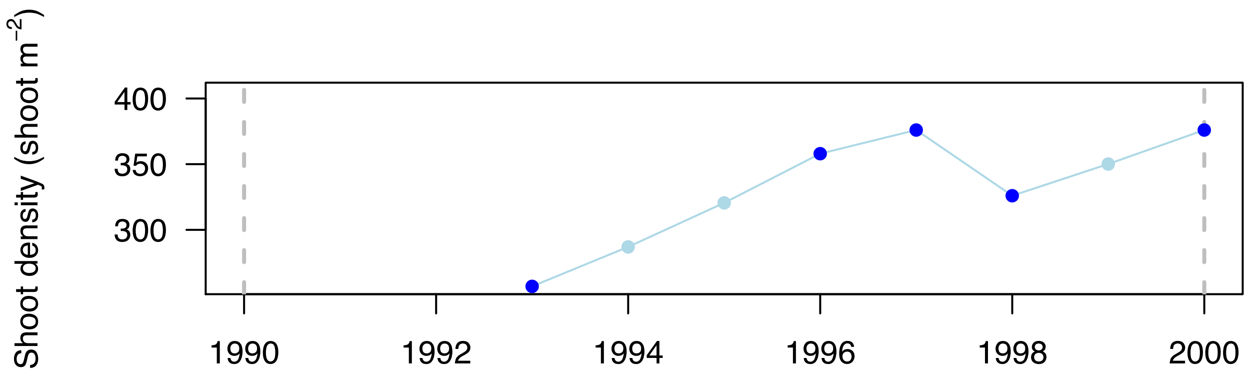

300_abiomass

Krause−Jensen et al. 2000

SITE: Saltholm (impacted) (Denmark – Baltic) – Zm (? m)

OVERALL: Net = 0 g dw m−2;   Rate = 0 % yr−1; Perc Final = 100 % > no change

DECADAL: NO (7 yr)

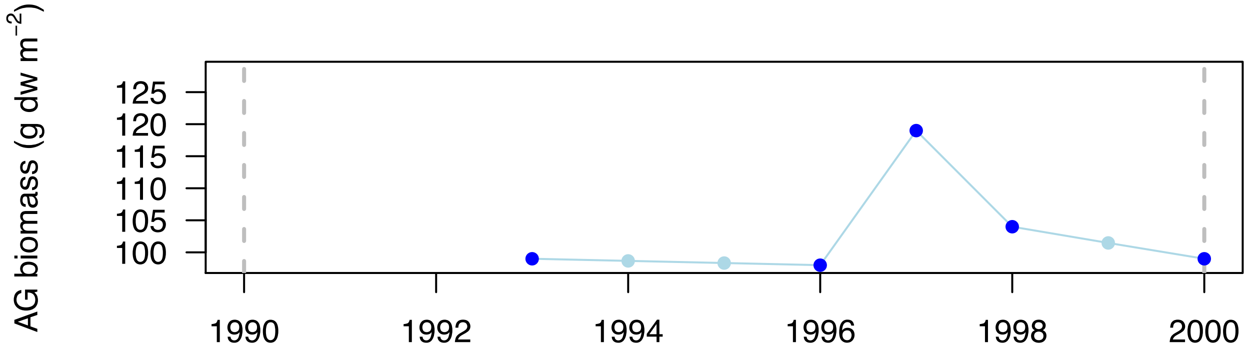

300_density

Krause–Jensen et al. 2000

SITE: Saltholm (impacted) (Denmark – Baltic) – Zm (? m)

OVERALL: Net = 224 shoot m–2;   Rate = 7.42 % yr–1; Perc Final = 168 % > increase

DECADAL: NO (7 yr)

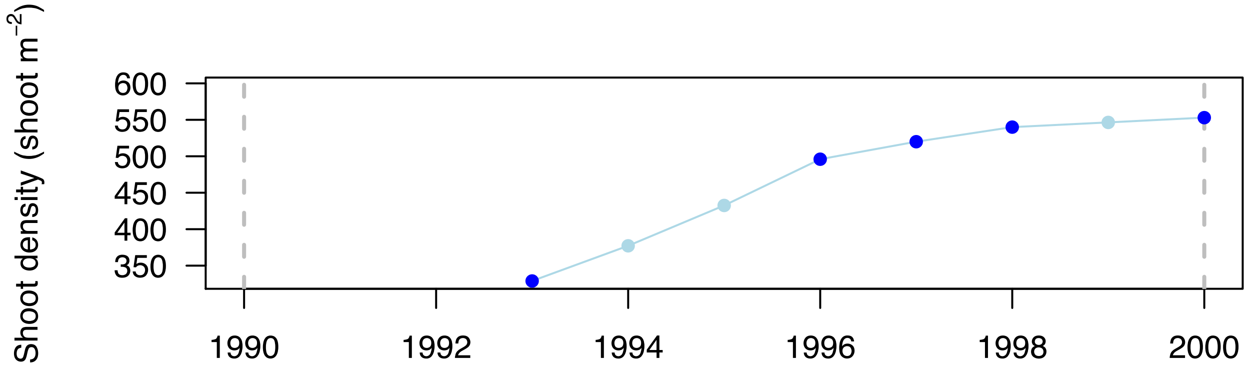

301_lowerlimit

Krause–Jensen and Rasmussen 2009, Carstensen and Krause–Jensen 2012

SITE: Præstø Fjord (Denmark – Baltic) – Zm (? m)

OVERALL: Net = –1.87 m;   Rate = –0.62 % yr–1; Perc Final = 66 % > decrease

DECADAL: YES (67 yr)

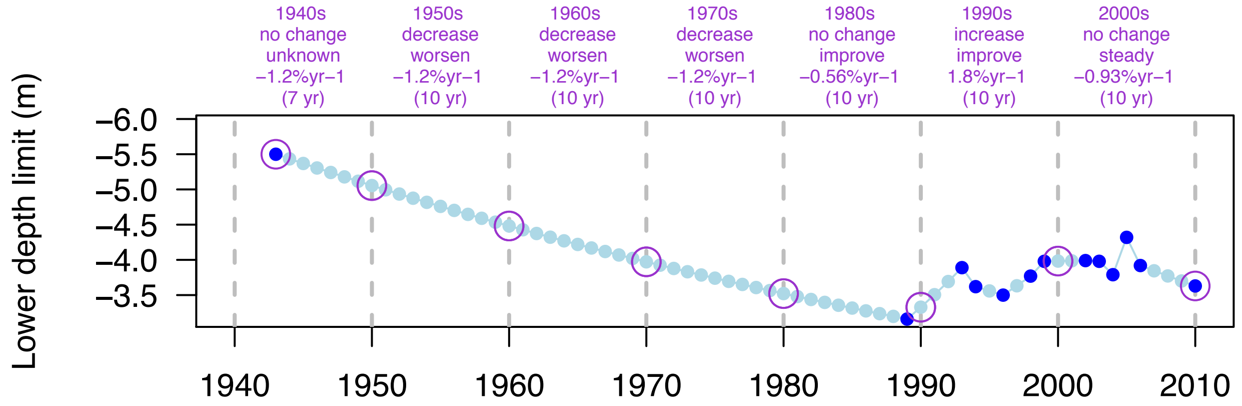

302_lowerlimit

Carstensen and Krause−Jensen 2012

SITE: Roskilde Fjord (Denmark − Baltic) − Zm (? m)

OVERALL: Net = −4.64 m;   Rate = −0.82 % yr−1; Perc Final = 38 % > decrease

DECADAL: YES (118 yr)

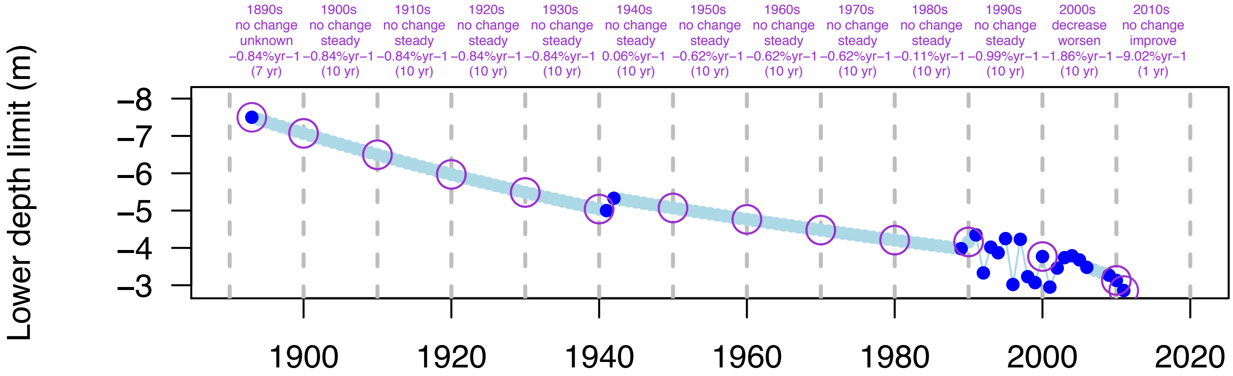

303_area

Frederiksen et al. 2004

SITE: Samsø (Stavns Fjord) (Denmark − Baltic) − Zm (? m)

OVERALL: Net = −60.5 ha;   Rate = −1.18 % yr−1; Perc Final = 59 % > decrease

DECADAL: YES (45 yr)

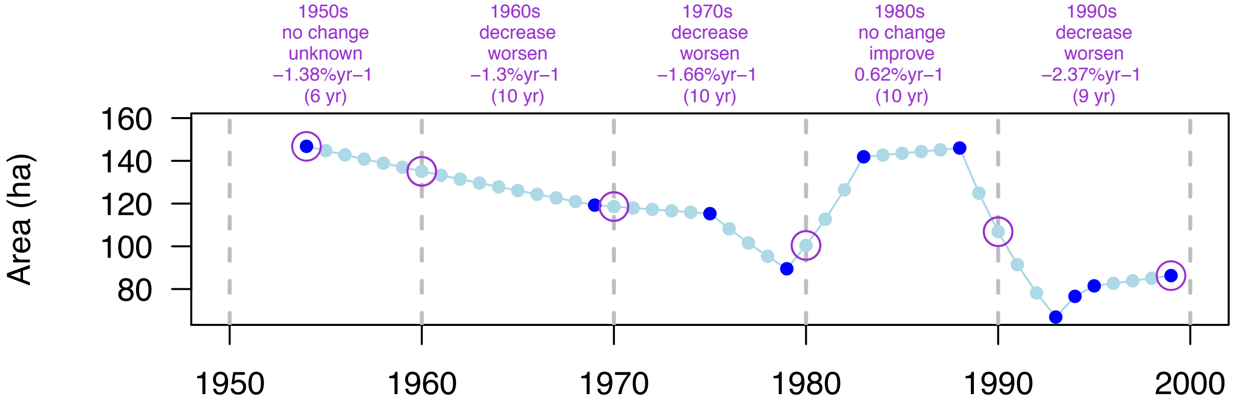

304_lowerlimit

Krause−Jensen and Rasmussen 2009, Carstensen and Krause−Jensen 2012

SITE: Sejerø Bugt (Denmark – Baltic) – Zm (? m)

OVERALL: Net = −7.52 m;   Rate = −0.73 % yr−1; Perc Final = 42 % > decrease

DECADAL: YES (119 yr)

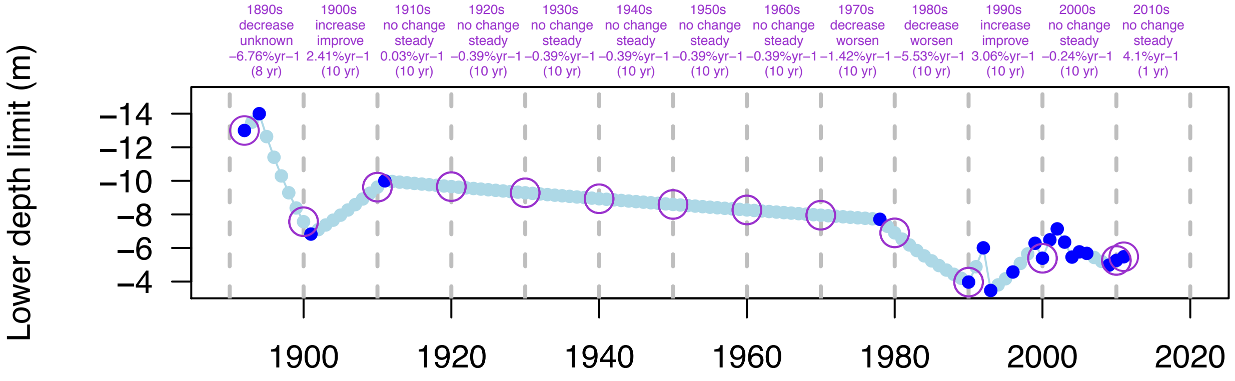

305_lowerlimit

Krause−Jensen and Rasmussen 2009, Carstensen and Krause−Jensen 2012, Carstensen et al. 2013

SITE: Skive Fjord (Denmark – Baltic) – Zm (? m)

OVERALL: Net = −3.43 m;   Rate = −0.81 % yr−1; Perc Final = 38 % > decrease

DECADAL: YES (121 yr)

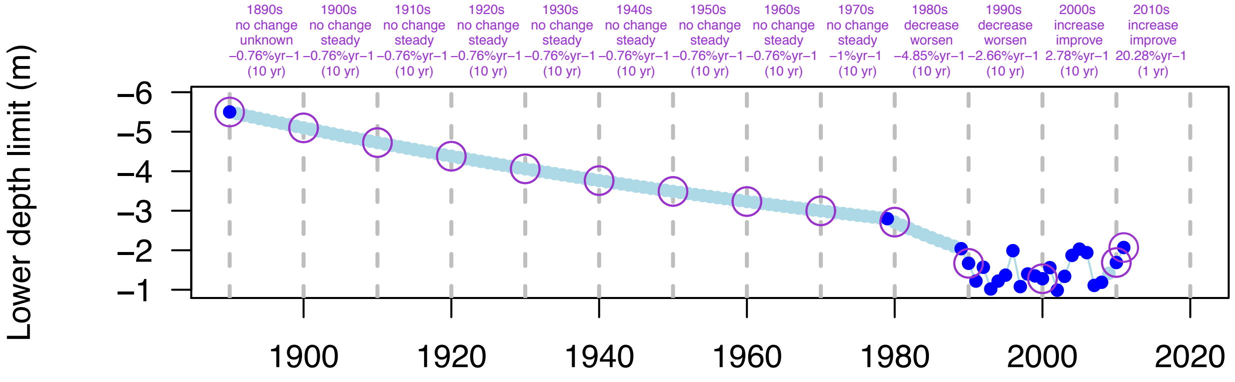

306_lowerlimit

Carstensen and Krause–Jensen 2012

SITE: Vejle Fjord (Denmark – Baltic) – Zm (? m)

OVERALL: Net = –3.62 m; Rate = –0.9 % yr–1; Perc Final = 34 % > decrease

DECADAL: YES (119 yr)

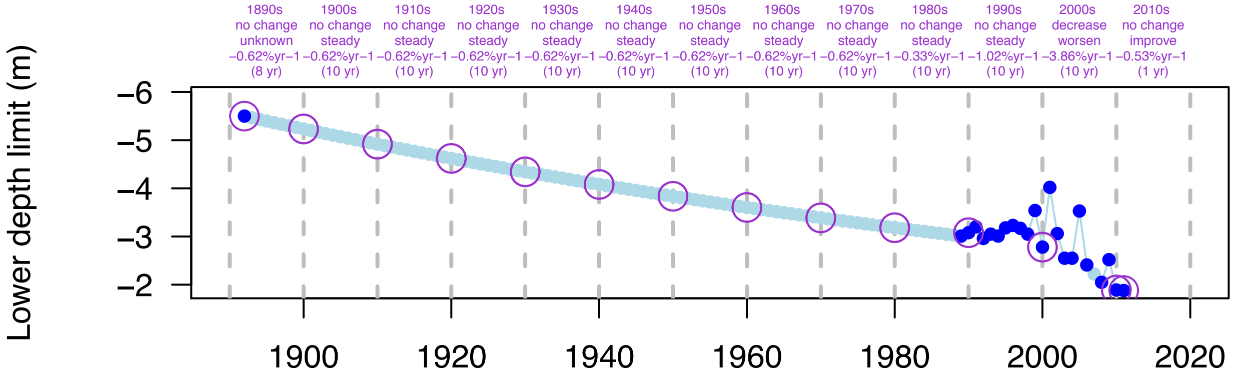

307_area

Frederiksen et al. 2004

SITE: Vejle Fjord (outer) (Denmark – Baltic) – Zm (? m)

OVERALL: Net = 3.6 ha; Rate = 2.95 % yr–1; Perc Final = 377 % > increase

DECADAL: YES (45 yr)

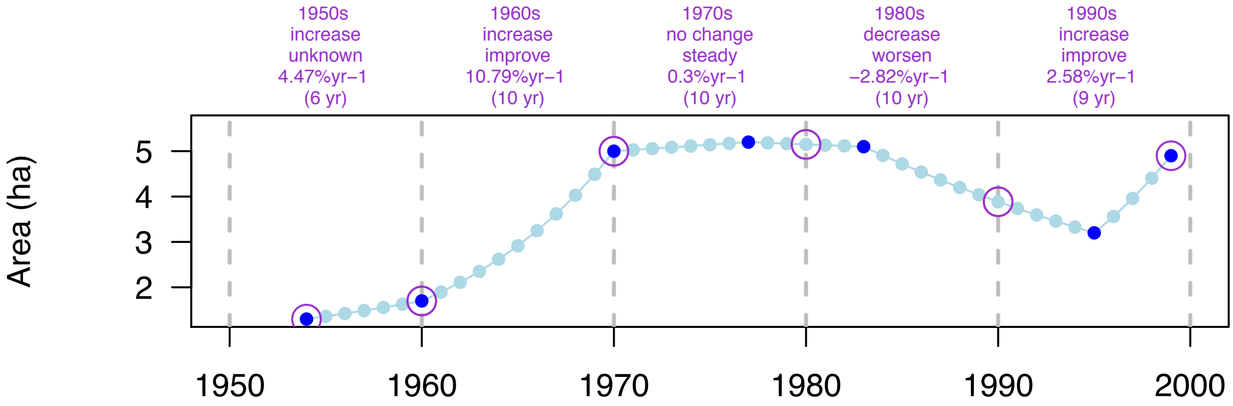

308_lowerlimit

Carstensen and Krause-Jensen 2012

SITE: Isefjord (Yderbredning) (Denmark – Baltic) – Zm (? m)

OVERALL: Net = 0.63 m; Rate = 0.12 % yr−1; Perc Final = 116 % > increase

DECADAL: YES (118 yr)

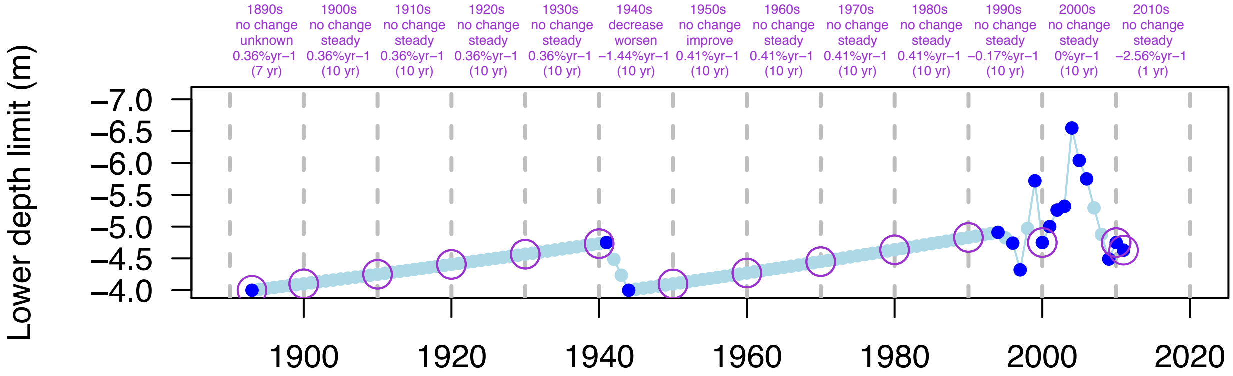

309_area

van der Graaf et al. 2009, Vinther (unpublished), Dolch et al. 2017

SITE: Grådyb (Denmark – Atlantic) – Zm (−0.2 m)

OVERALL: Net = −222.818 ha; Rate = −3.45 % yr−1; Perc Final = 17 % > decrease

DECADAL: YES (51 yr)

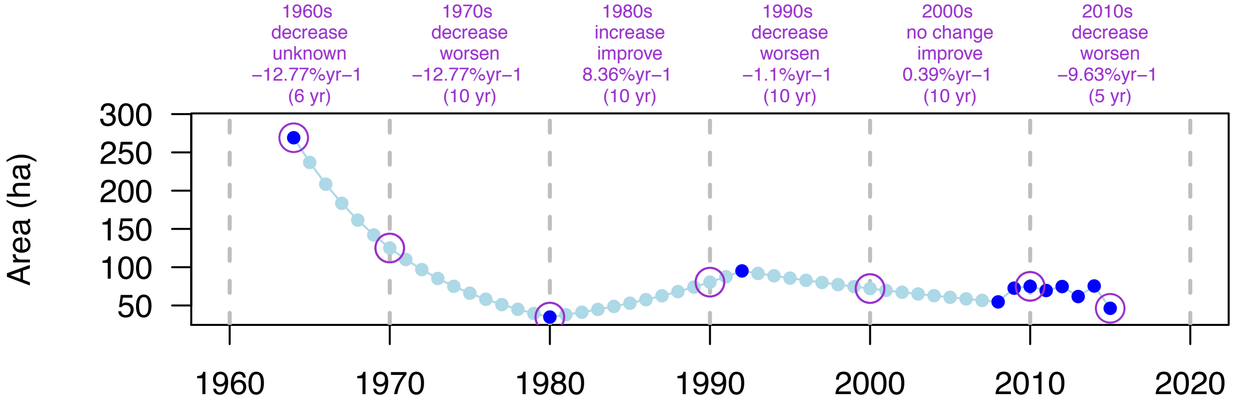

310_area

van der Graaf et al. 2009, Vinther (unpublished), Dolch et al. 2017

SITE: Juvre Dyb (Denmark – Atlantic) – Zm (0 m)

OVERALL: Net = −106.604 ha;   Rate = −0.4 % yr−1; Perc Final = 75 % > decrease

DECADAL: YES (70 yr)

1940s	1950s	1960s	1970s	1980s	1990s	2000s	2010s
increase	increase	increase	decrease	decrease	decrease	decrease	decrease
unknown	improve	improve	worsen	worsen	worsen	worsen	worsen
1.95%yr−1	1.95%yr−1	1.29%yr−1	−1.39%yr−1	−1.39%yr−1	−1.39%yr−1	−1.14%yr−1	−3.47%yr−1
(5 yr)	(10 yr)	(10 yr)	(10 yr)	(10 yr)	(10 yr)	(10 yr)	(5 yr)

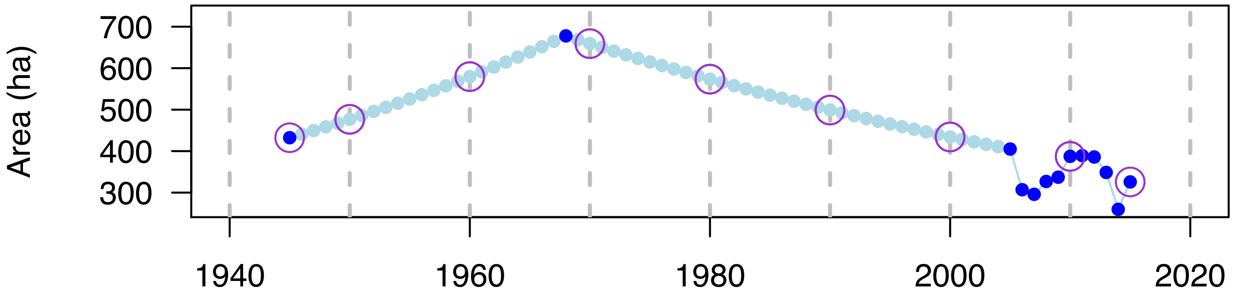

311_area

van der Graaf et al. 2009, Vinther (unpublished), Dolch et al. 2017

SITE: Knude Dyb (Denmark – Atlantic) – Zm (−0.2 m)

OVERALL: Net = −366.826 ha;   Rate = −9.13 % yr−1; Perc Final = 1 % > decrease

DECADAL: YES (51 yr)

1960s	1970s	1980s	1990s	2000s	2010s
increase	decrease	decrease	no change	decrease	decrease
unknown	worsen	worsen	improve	worsen	worsen
3.98%yr−1	−23.57%yr−1	−3.5%yr−1	−0.52%yr−1	−1.59%yr−1	−39.51%yr−1
(6 yr)	(10 yr)	(10 yr)	(10 yr)	(10 yr)	(5 yr)

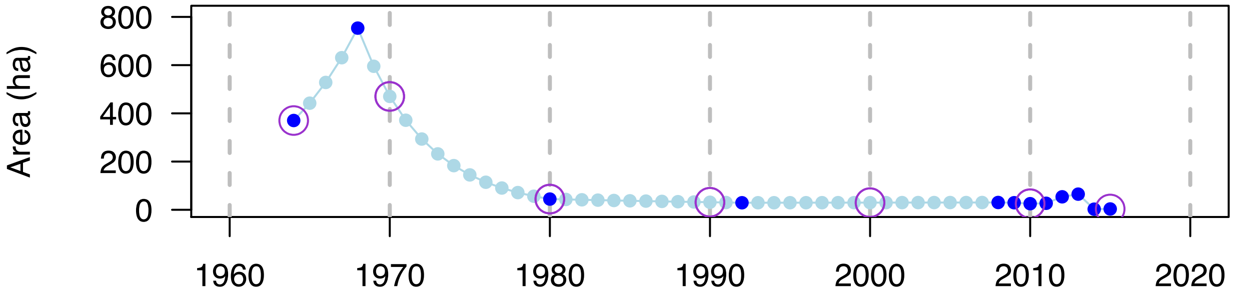

312_area

van der Graaf et al. 2009, Vinther (unpublished), Dolch et al. 2017

SITE: Lister Dyb (Denmark – Atlantic) – Zm (–0.2 m)

OVERALL: Net = –288.493 ha;   Rate = –0.92 % yr–1; Perc Final = 52 % > decrease

DECADAL: YES (70 yr)

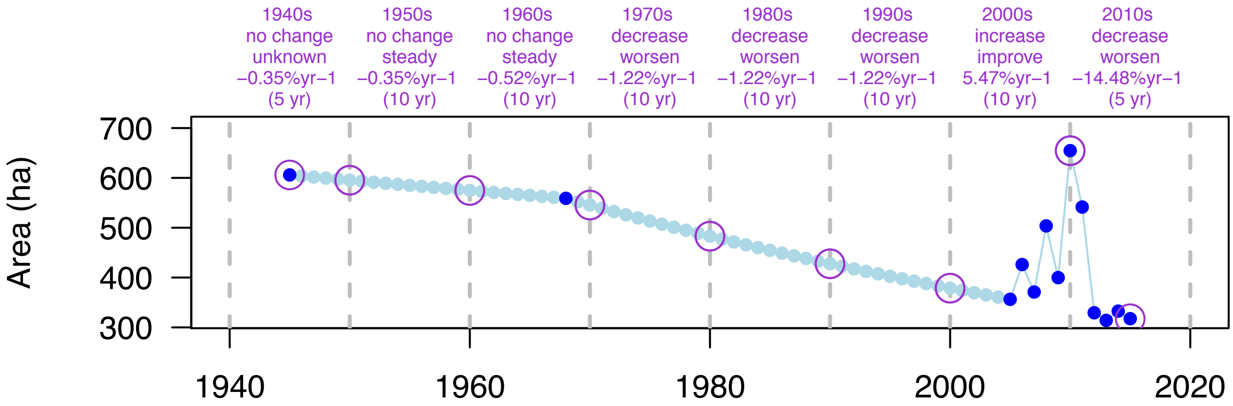

314_lowerlimit

Bernard et al. 2007

SITE: Berre Lagoon (France – Mediterranean) – Zm (? m)

OVERALL: Net = –5.5 m;   Rate = –2.54 % yr–1; Perc Final = 8 % > decrease

DECADAL: YES (98 yr)

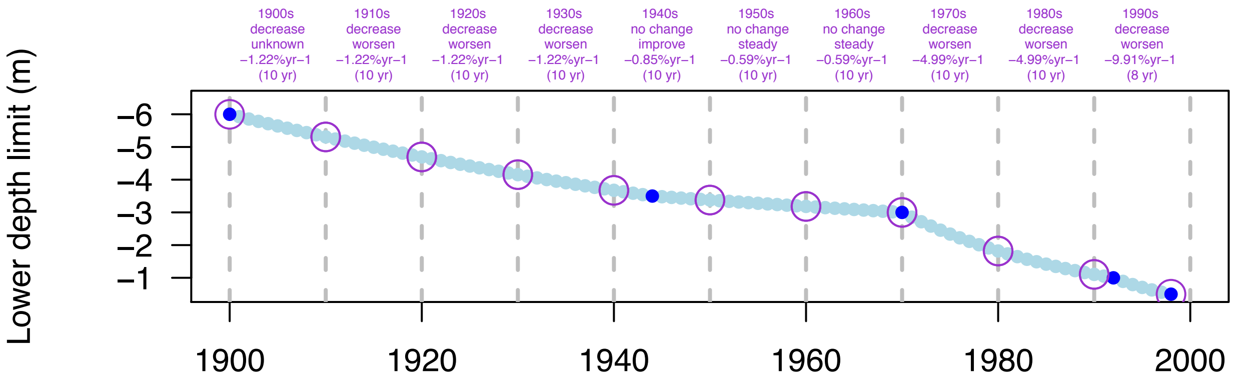

315_area

Bernard et al. 2007

SITE: Berre Lagoon (Figuerolles) (France – Mediterranean) – Zm (? m)

OVERALL: Net = –22.41 ha; Rate = –11.7 % yr–1; Perc Final = 0 % > decrease

DECADAL: YES (60 yr)

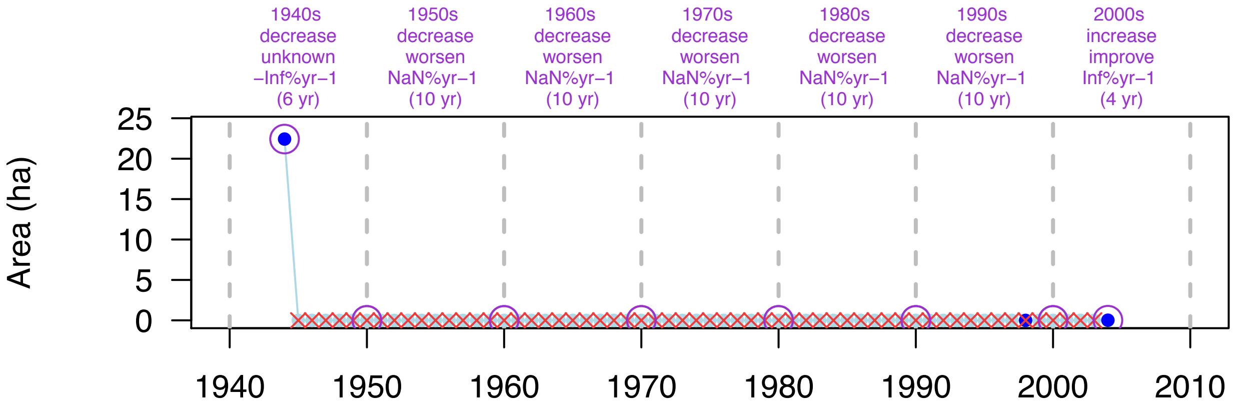

316_area

Bernard et al. 2007

SITE: Berre Lagoon (Martigues) (France – Mediterranean) – Zm (? m)

OVERALL: Net = –13.57 ha; Rate = –10.87 % yr–1; Perc Final = 0 % > decrease

DECADAL: YES (60 yr)

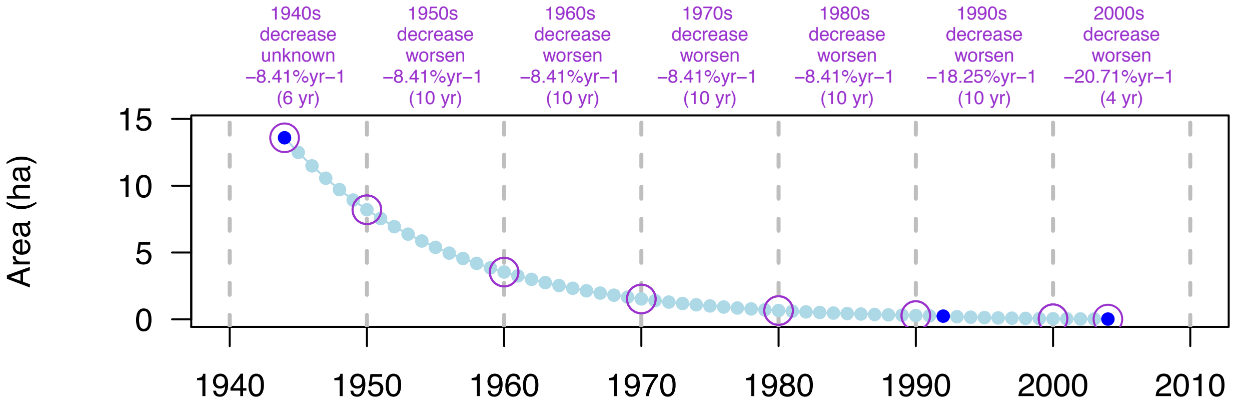

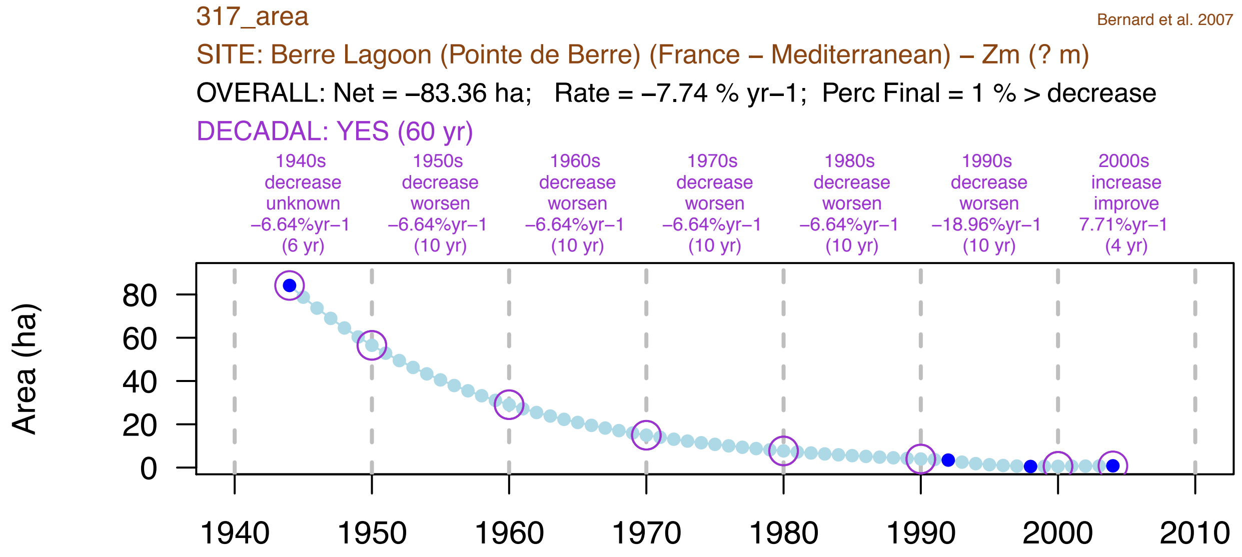

317_area
Bernard et al. 2007
SITE: Berre Lagoon (Pointe de Berre) (France – Mediterranean) – Zm (? m)
OVERALL: Net = –83.36 ha; Rate = –7.74 % yr–1; Perc Final = 1 % > decrease
DECADAL: YES (60 yr)
1940s decrease unknown –6.64%yr–1 (6 yr)
1950s decrease worsen –6.64%yr–1 (10 yr)
1960s decrease worsen –6.64%yr–1 (10 yr)
1970s decrease worsen –6.64%yr–1 (10 yr)
1980s decrease worsen –6.64%yr–1 (10 yr)
1990s decrease worsen –18.96%yr–1 (10 yr)
2000s increase improve 7.71%yr–1 (4 yr)
Area (ha)
80
60
40
20
0
1940
1950
1960
1970
1980
1990
2000
2010

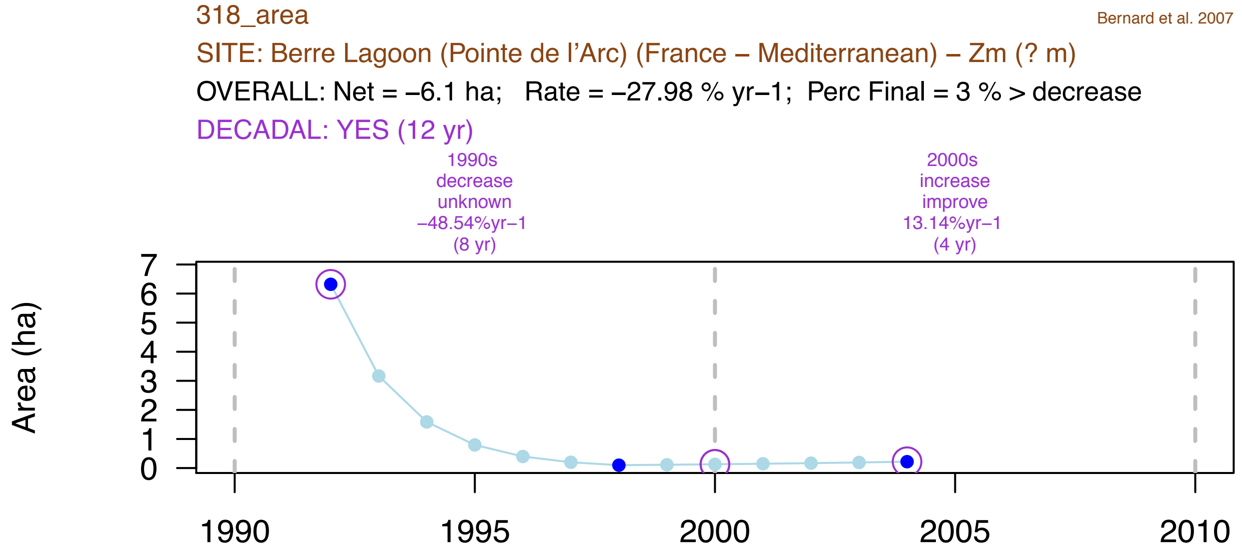

318_area
Bernard et al. 2007
SITE: Berre Lagoon (Pointe de l'Arc) (France – Mediterranean) – Zm (? m)
OVERALL: Net = –6.1 ha; Rate = –27.98 % yr–1; Perc Final = 3 % > decrease
DECADAL: YES (12 yr)
1990s decrease unknown –48.54%yr–1 (8 yr)
2000s increase improve 13.14%yr–1 (4 yr)
Area (ha)
7
6
5
4
3
2
1
0
1990
1995
2000
2005
2010

319_area

Astier 1984, Bourcier 1996, 1989, Picard and Bourcier 1976, Picard 1978

SITE: La Ciotat – Les Leques (France – Mediterranean) – Po (? m)

OVERALL: Net = –139.56 ha; Rate = –0.71 % yr–1; Perc Final = 84 % > decrease

DECADAL: YES (24 yr)

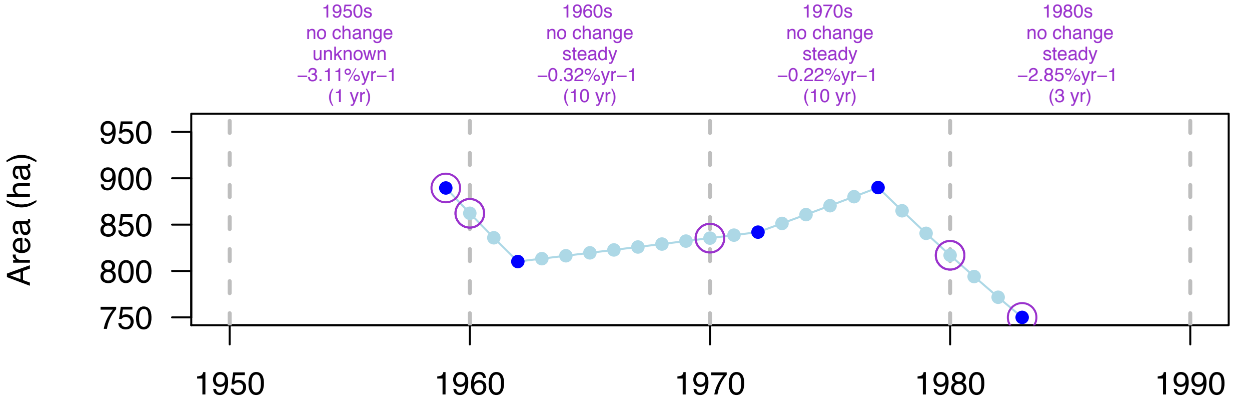

319_lowerlimit

Astier 1984, Bourcier 1996, 1989, Picard and Bourcier 1976, Picard 1978

SITE: La Ciotat – Les Leques (France – Mediterranean) – Po (? m)

OVERALL: Net = –8 m; Rate = –5.19 % yr–1; Perc Final = 77 % > decrease

DECADAL: NO (5 yr)

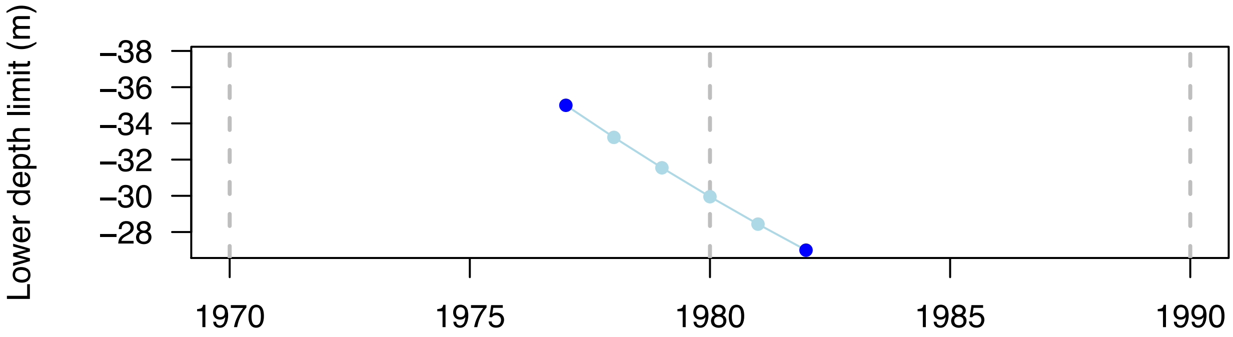

321_cover

Bonhomme et al. 2010

SITE: La Palud Cove (France – Mediterranean) – Po (–34 m)

OVERALL: Net = –7.8 %;   Rate = –6.36 % yr–1; Perc Final = 68 % > decrease

DECADAL: NO (6 yr)

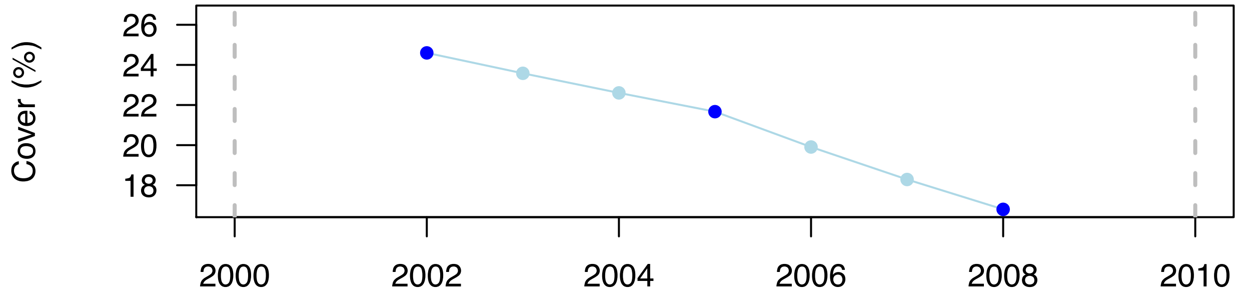

321_density

Bonhomme et al. 2010

SITE: La Palud Cove (France – Mediterranean) – Po (–34 m)

OVERALL: Net = –73.4 shoot m–2;   Rate = –15.42 % yr–1; Perc Final = 40 % > decrease

DECADAL: NO (6 yr)

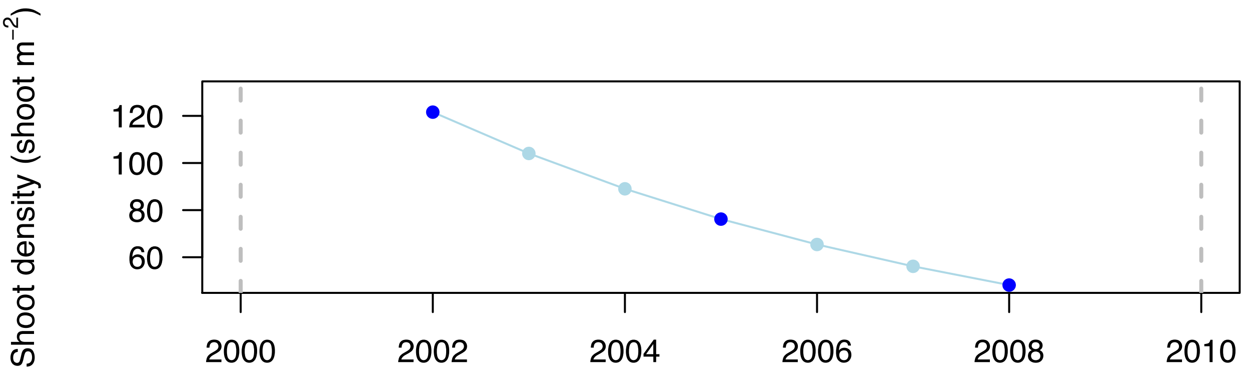

322_cover

Bonhomme et al. 2010

SITE: Vaisseau – Tuf (France – Mediterranean) – Po (–34 m)

OVERALL: Net = –18.4 %;   Rate = –21.63 % yr–1; Perc Final = 65 % > decrease

DECADAL: NO (2 yr)

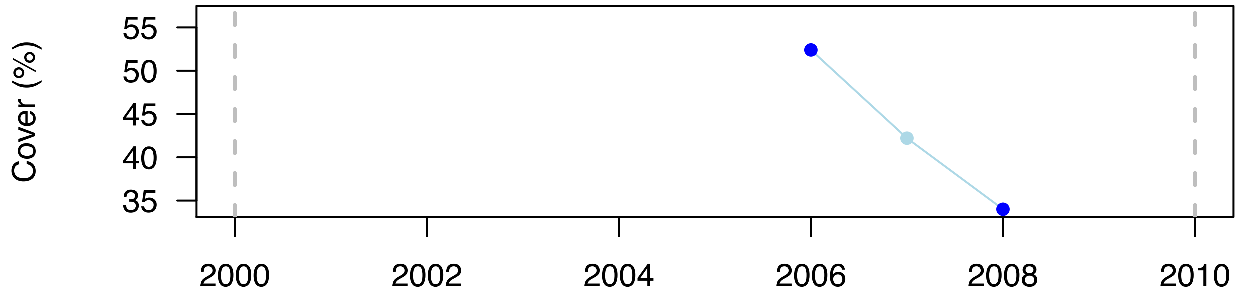

322_density

Bonhomme et al. 2010

SITE: Vaisseau – Tuf (France – Mediterranean) – Po (–34 m)

OVERALL: Net = –3.2 shoot m–2;   Rate = –1.54 % yr–1; Perc Final = 97 % > no change

DECADAL: NO (2 yr)

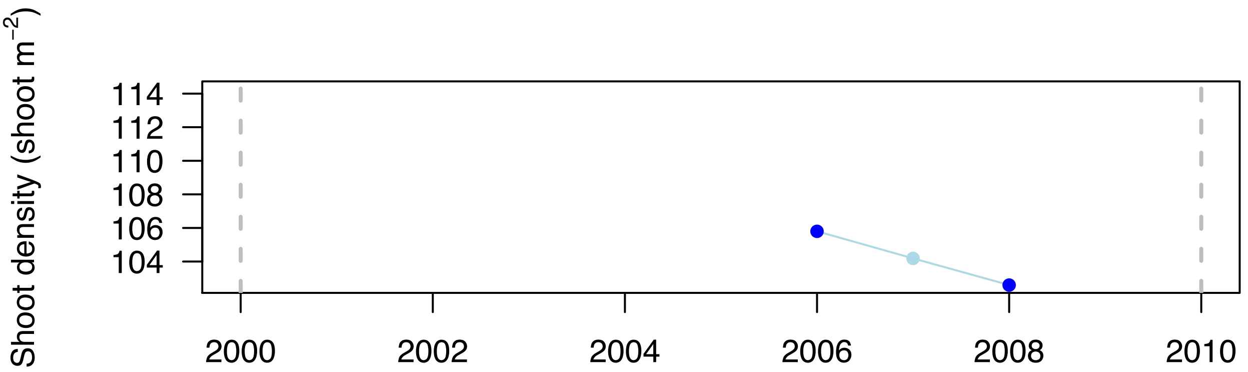

323_area

Astruch et al. 2012

SITE: Bay de Port–Cros (barrier reef) (France – Mediterranean) – Po (–20 m)

OVERALL: Net = –0.31 ha; Rate = –0.66 % yr–1; Perc Final = 48 % > decrease

DECADAL: YES (110 yr)

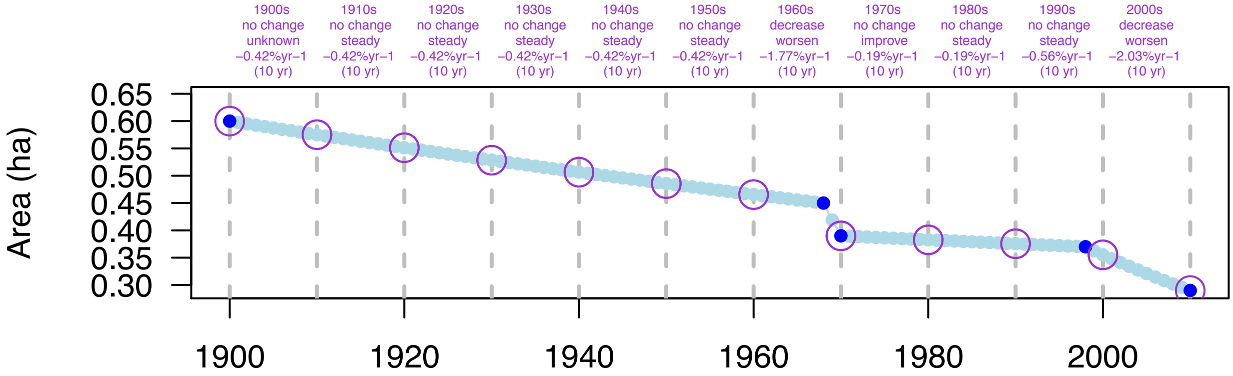

324_area

Astier 1972, 1975, 1984, Nodot et al. 1978

SITE: Plages du Mourillon (France – Mediterranean) – Po (? m)

OVERALL: Net = –236 ha; Rate = –5.58 % yr–1; Perc Final = 35 % > decrease

DECADAL: YES (19 yr)

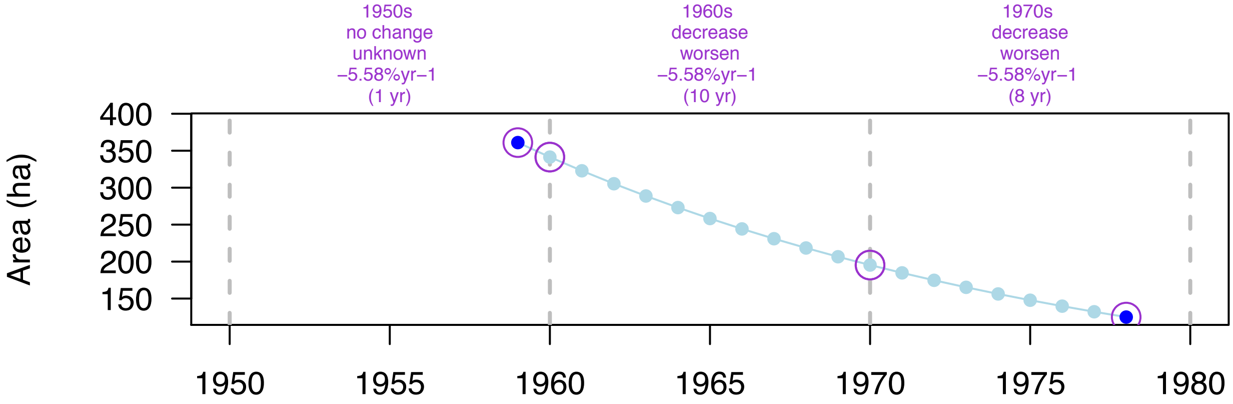

325_area

Glemarec et al. 1997, Hily et al. 2003

SITE: Glenan Archipelago (France – Atlantic) – Zm (–5 m)

OVERALL: Net = –35.2 ha;   Rate = –0.12 % yr–1; Perc Final = 92 % > no change

DECADAL: YES (68 yr)

1930s	1940s	1950s	1960s	1970s	1980s	1990s
decrease	decrease	increase	no change	no change	decrease	increase
unknown	worsen	improve	steady	steady	worsen	improve
–6.38%yr–1	–6.38%yr–1	3.17%yr–1	–0.42%yr–1	–0.5%yr–1	–1.19%yr–1	9.58%yr–1
(8 yr)	(10 yr)	(10 yr)	(10 yr)	(10 yr)	(10 yr)	(10 yr)

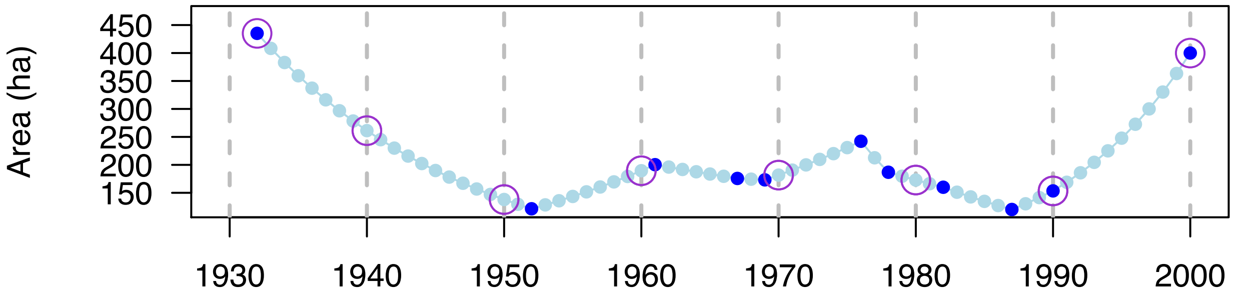

326_area

Plus et al. 2010

SITE: Arcachon Bay (France – Atlantic) – Zn (0 m)

OVERALL: Net = –2280 ha;   Rate = –2.25 % yr–1; Perc Final = 67 % > decrease

DECADAL: YES (18 yr)

1980s	1990s	2000s
no change	no change	decrease
unknown	steady	worsen
–0.75%yr–1	–0.75%yr–1	–4.61%yr–1
(1 yr)	(10 yr)	(7 yr)

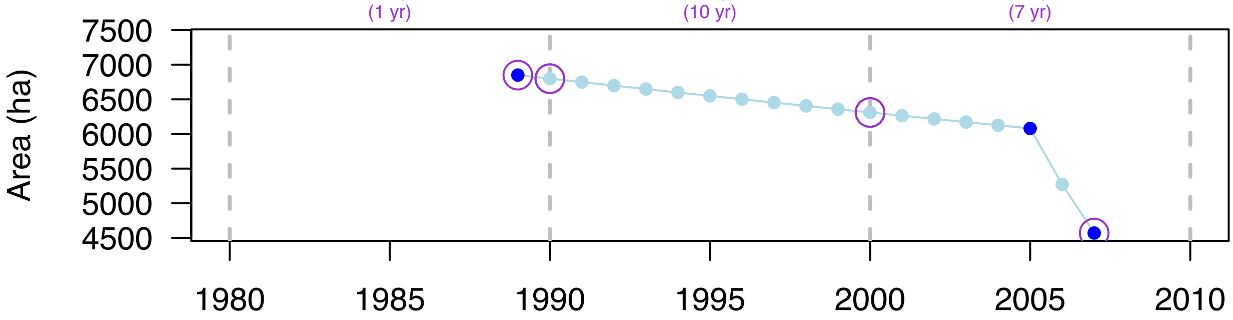

327_area

Plus et al. 2010

SITE: Arcachon Bay (France – Atlantic) – Zm (–5 m)

OVERALL: Net = –270 ha; Rate = –6.54 % yr–1; Perc Final = 27 % > decrease

DECADAL: YES (20 yr)

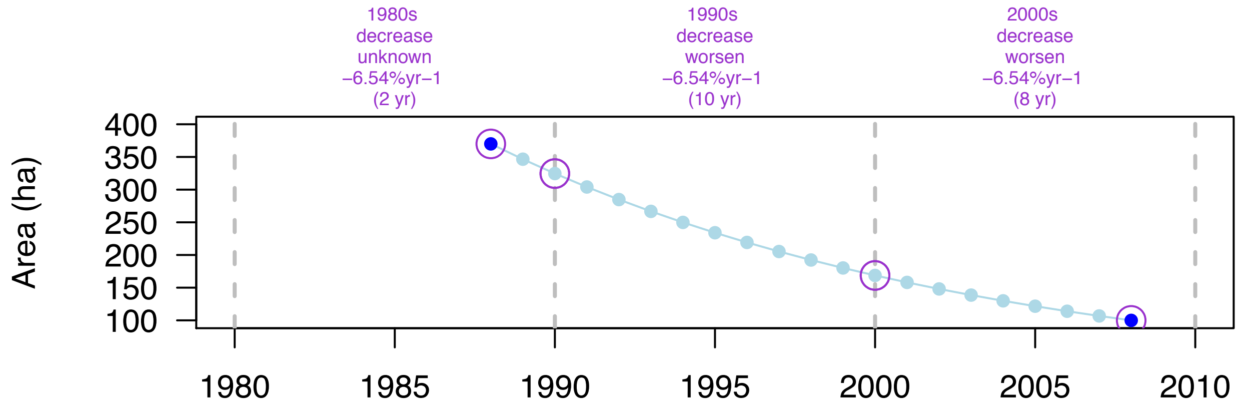

328_area

Plus et al. 2010

SITE: Arguin Bank (France – Atlantic) – Zm (–5 m)

OVERALL: Net = –7 ha; Rate = –7.52 % yr–1; Perc Final = 22 % > decrease

DECADAL: YES (20 yr)

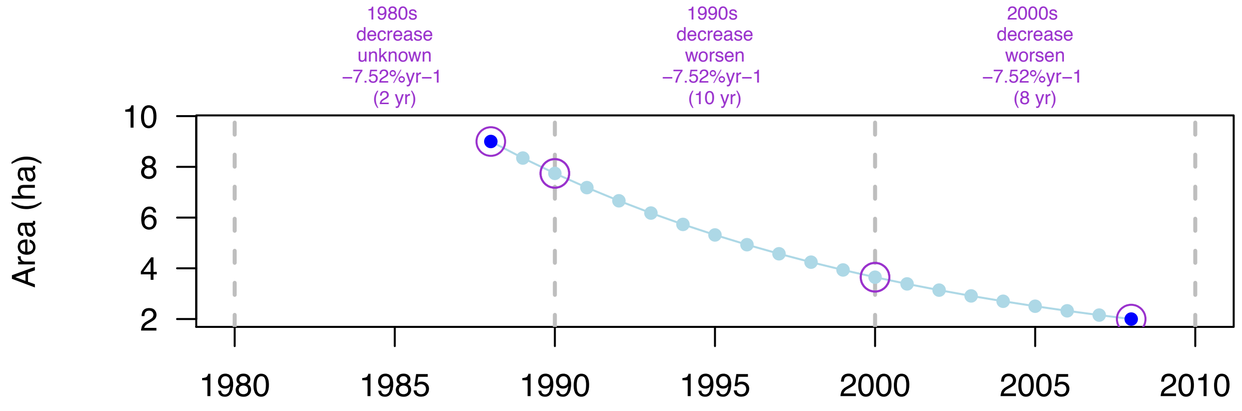

329_area Leriche et al. 2006

SITE: Bay of Saint–Cyr (France – Mediterranean) – Po (? m)

OVERALL: Net = −10.08 ha; Rate = −0.76 % yr−1; Perc Final = 85 % > decrease

DECADAL: YES (21 yr)

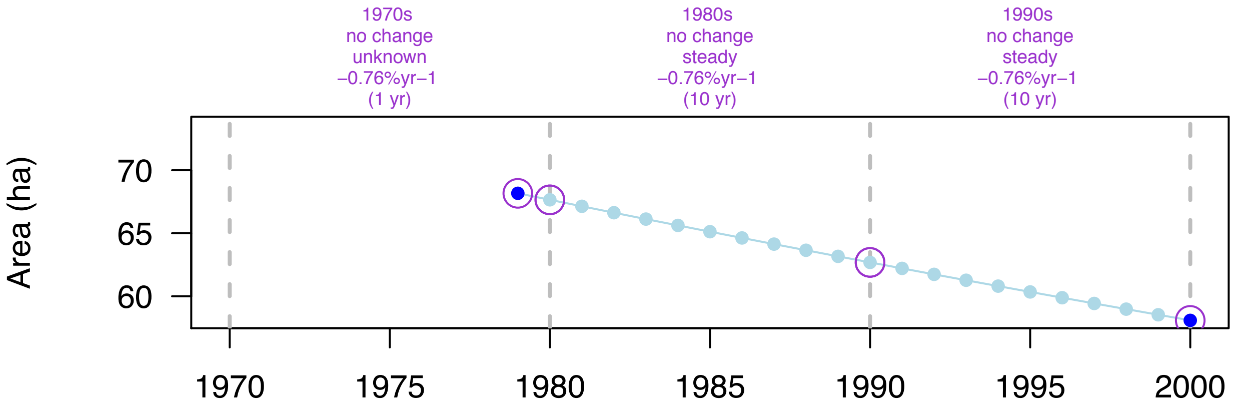

330_area Pergent–Martini et al. 2002

SITE: Marseilles (test area) (France – Mediterranean) – Po (−15 m)

OVERALL: Net = 0.14 ha; Rate = 1.27 % yr−1; Perc Final = 121 % > increase

DECADAL: YES (15 yr)

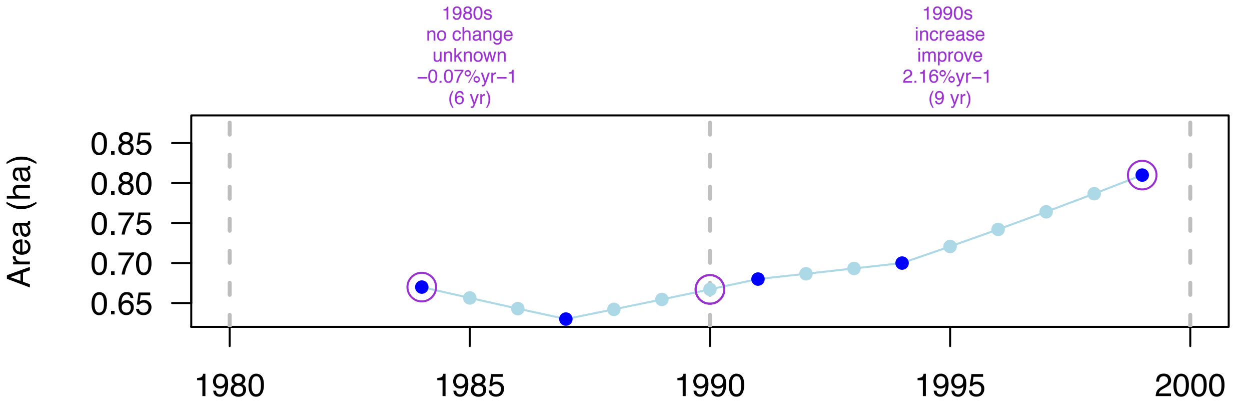

331_density

Pergent–Martini et al. 2002

SITE: Marseilles (Cortiou – anthropissed) (France – Mediterranean) – Po (? m)

OVERALL: Net = –176 shoot m–2; Rate = –3.21 % yr–1; Perc Final = 68 % > decrease

DECADAL: YES (12 yr)

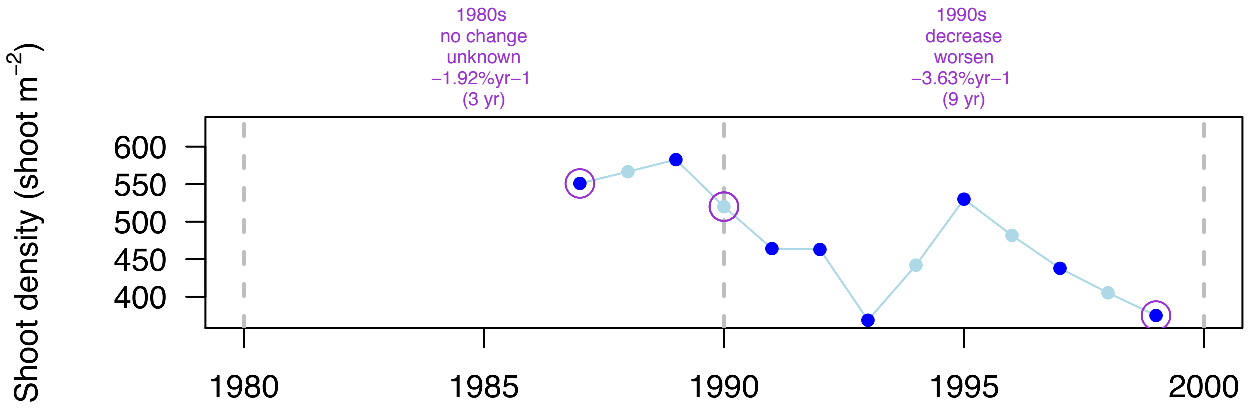

331_lowerlimit

Pergent–Martini et al. 2002

SITE: Marseilles (Cortiou – anthropissed) (France – Mediterranean) – Po (? m)

OVERALL: Net = 0 m; Rate = 0 % yr–1; Perc Final = 100 % > no change

DECADAL: YES (10 yr)

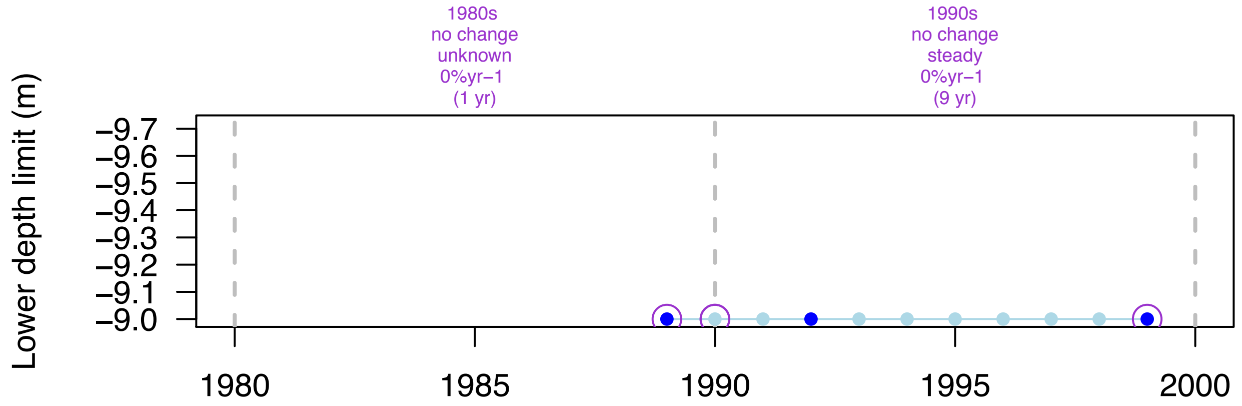

332_density

Pergent–Martini et al. 2002

SITE: Marseilles (Riou – reference) (France – Mediterranean) – Po (? m)

OVERALL: Net = 2 shoot m–2;   Rate = 0.07 % yr–1; Perc Final = 101 % > no change

DECADAL: YES (12 yr)

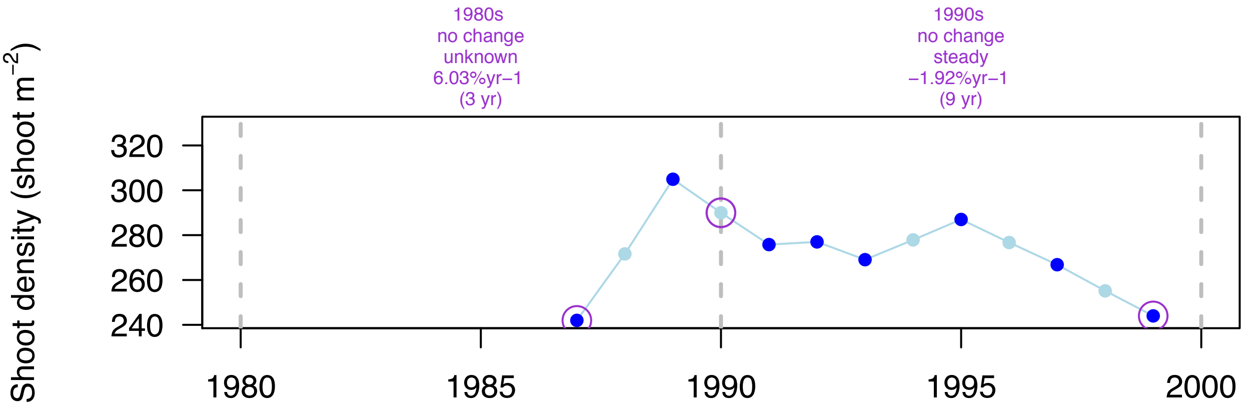

332_lowerlimit

Pergent–Martini et al. 2002

SITE: Marseilles (Riou – reference) (France – Mediterranean) – Po (? m)

OVERALL: Net = 0 m;   Rate = 0 % yr–1; Perc Final = 100 % > no change

DECADAL: YES (10 yr)

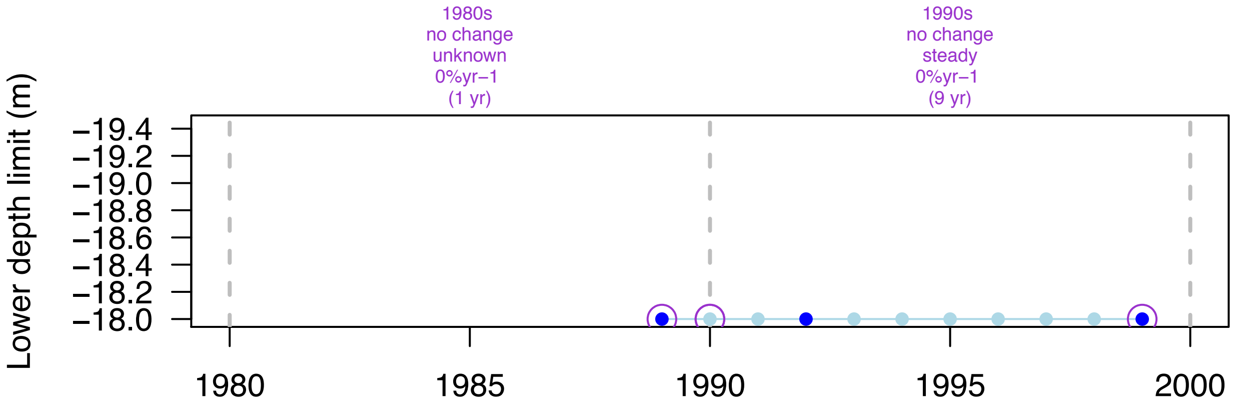

333_area

Pergent–Martini and Pergent 1996

SITE: Plateau des Chèvres (France – Mediterranean) – Po (? m)

OVERALL: Net = −186 ha;   Rate = −2.82 % yr−1; Perc Final = 59 % > decrease

DECADAL: YES (19 yr)

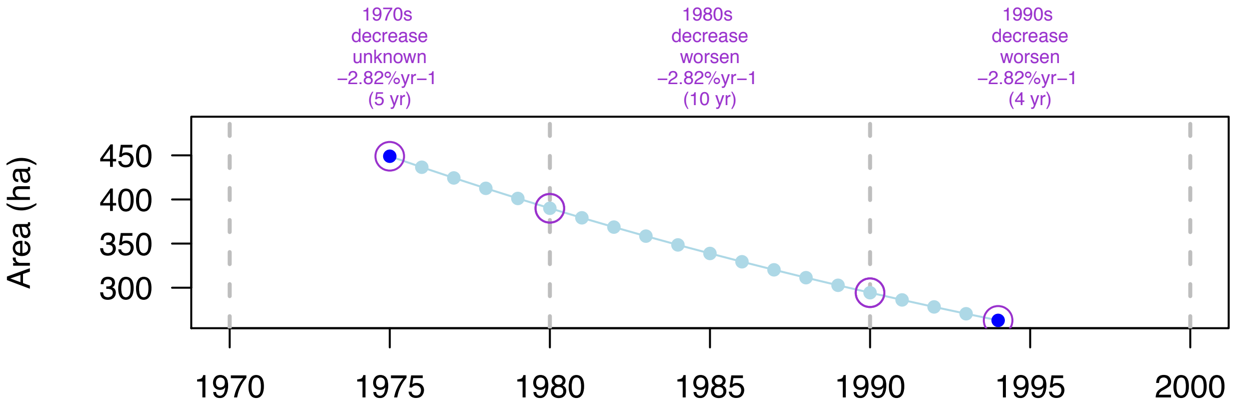

333_lowerlimit

Pergent–Martini and Pergent 1996

SITE: Plateau des Chèvres (France – Mediterranean) – Po (? m)

OVERALL: Net = 0 m;   Rate = 0 % yr−1; Perc Final = 100 % > no change

DECADAL: NO (7 yr)

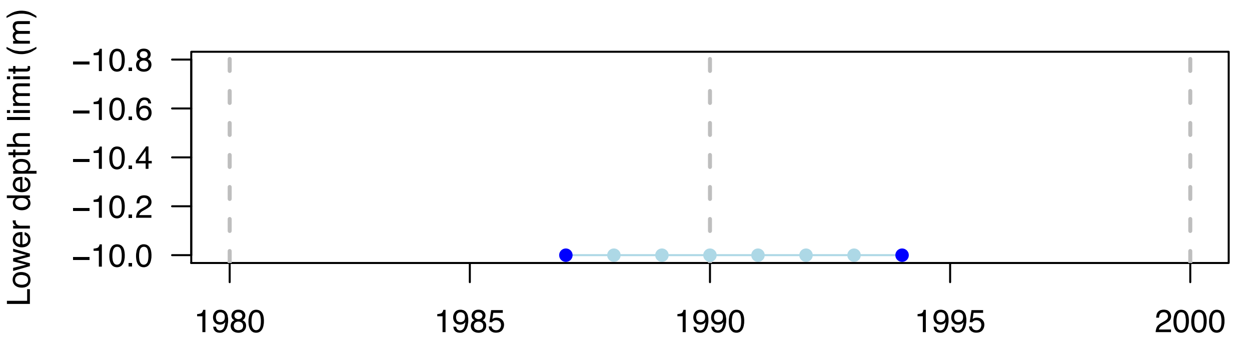

334_area

Bonacorsi et al. 2013

SITE: Cap Corse (entire) (France – Mediterranean) – Po (? m)

OVERALL: Net = −231 ha;   Rate = −0.14 % yr−1; Perc Final = 98 % > no change

DECADAL: YES (16 yr)

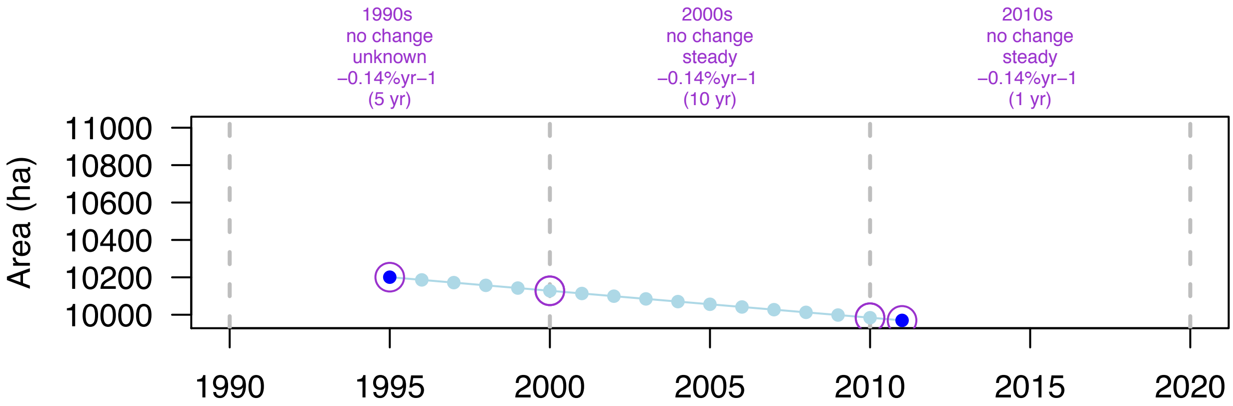

335_area

Bonacorsi et al. 2013

SITE: Cap Corse (Macinaggio) (France – Mediterranean) – Po (? m)

OVERALL: Net = −43 ha;   Rate = −0.8 % yr−1; Perc Final = 88 % > decrease

DECADAL: YES (16 yr)

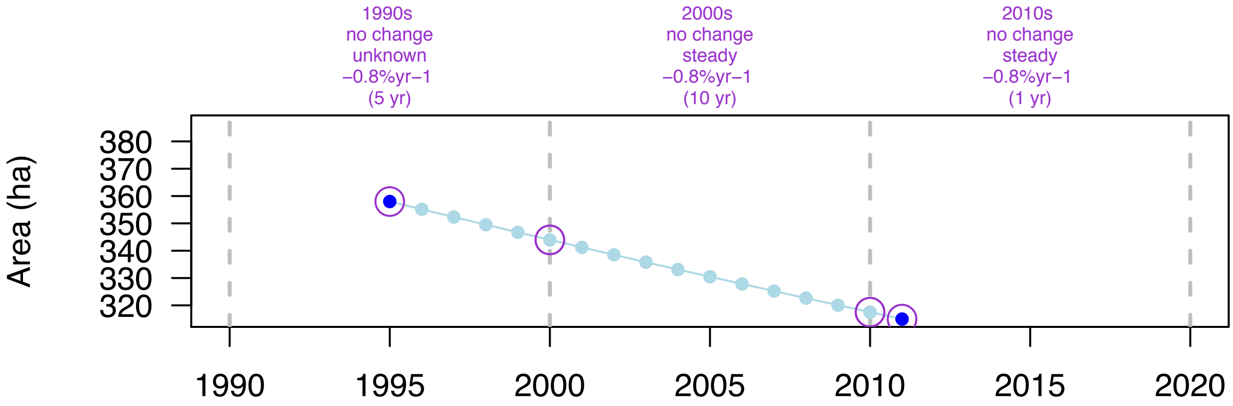

336_area

Bonacorsi et al. 2013

SITE: Cap Corse (Saint Florent) (France – Mediterranean) – Po (? m)

OVERALL: Net = −1.08 ha;   Rate = −0.22 % yr−1; Perc Final = 90 % > no change

DECADAL: YES (46 yr)

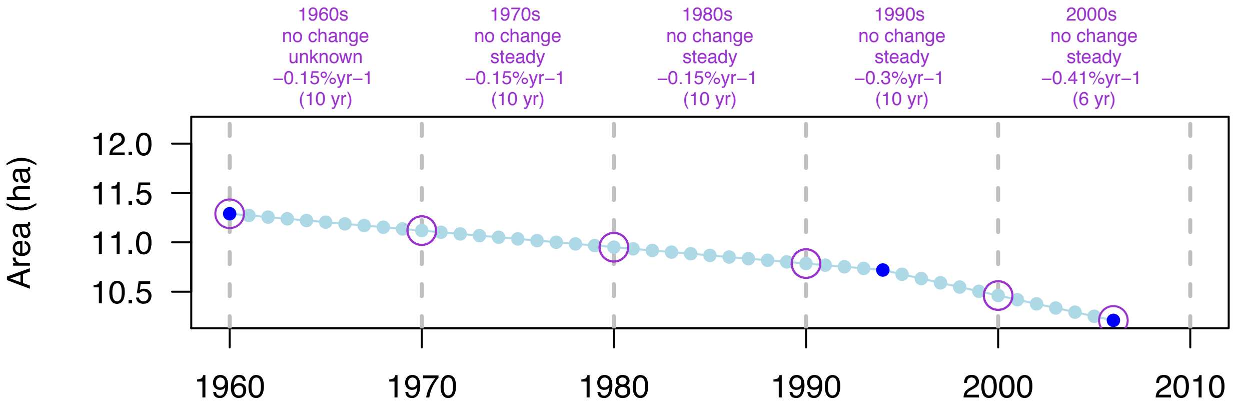

337_area

Bonacorsi et al. 2013

SITE: Cap Corse (Saint Florent) (France – Mediterranean) – Cn (? m)

OVERALL: Net = −7 ha;   Rate = −1.98 % yr−1; Perc Final = 40 % > decrease

DECADAL: YES (46 yr)

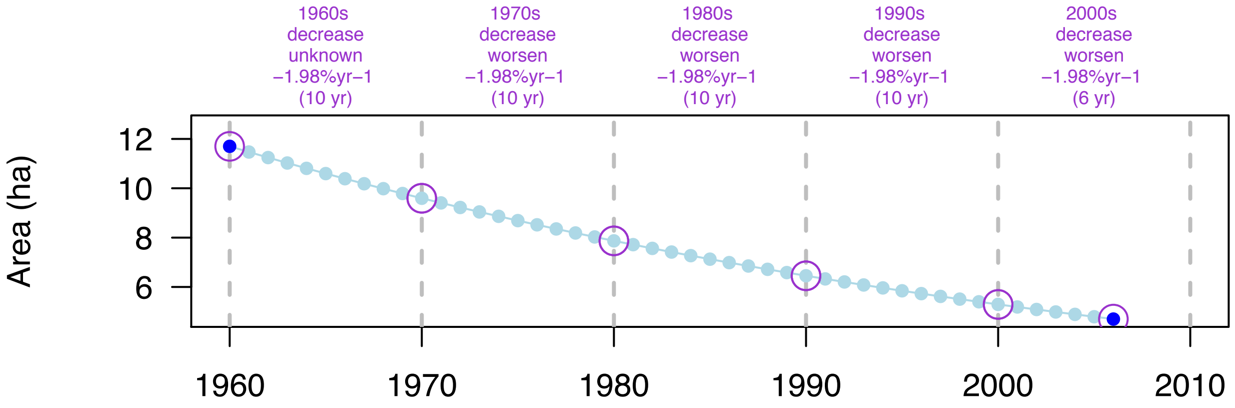

338_area

Fournier et al. 2006, Auby et al. 2010

SITE: Plage de les Haas (France – Atlantic) – Zn (0 m)

OVERALL: Net = 21 ha;   Rate = NA % yr–1; Perc Final = NA % > increase

DECADAL: YES (80 yr)

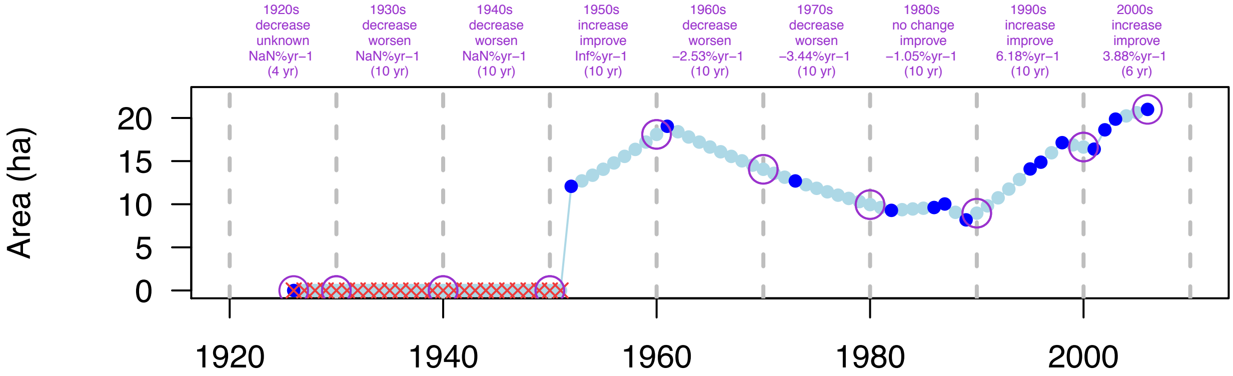

339_area

Dolch et al. 2013, Dolch et al. 2017

SITE: Northfrisian Wadden Sea (Germany – Atlantic) – Zn (0 m)

OVERALL: Net = 2434.56 ha;   Rate = 0.22 % yr–1; Perc Final = 119 % > increase

DECADAL: YES (80 yr)

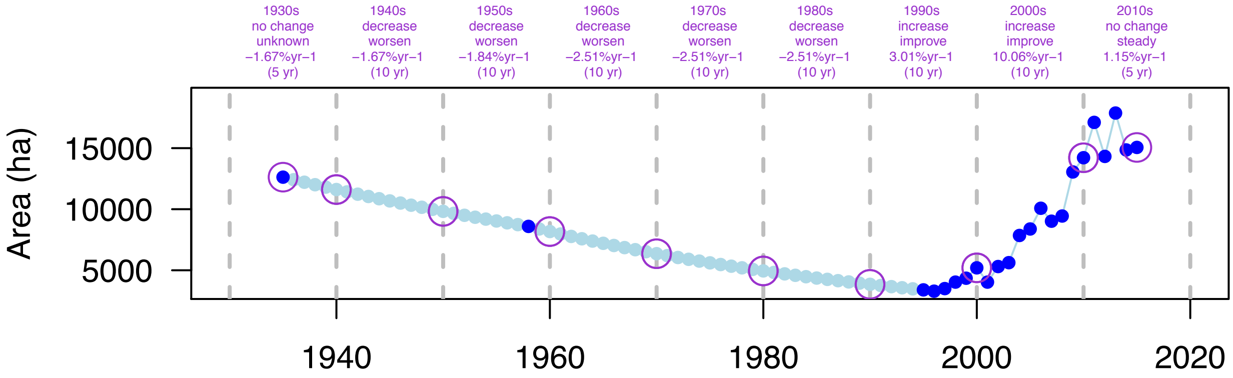

342_area

Gambi et al. 2005

SITE: Maronti Bay (Italy – Mediterranean) – Po (? m)

OVERALL: Net = –2 ha;   Rate = –34.66 % yr–1; Perc Final = 50 % > decrease

DECADAL: NO (2 yr)

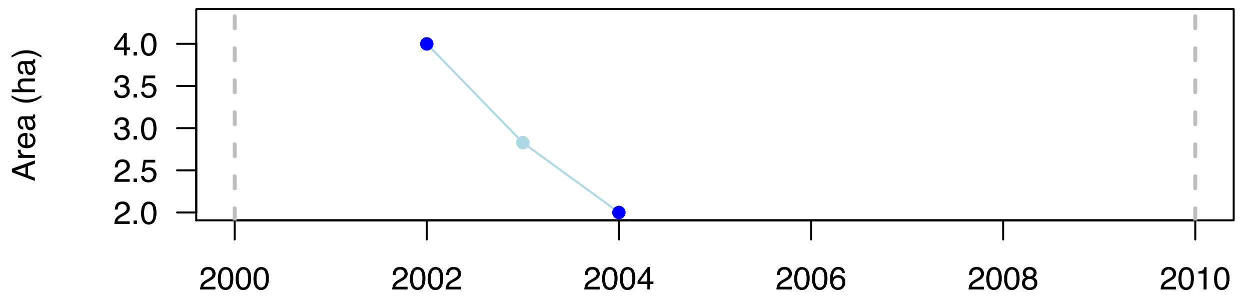

342_lowerlimit

Gambi et al. 2005

SITE: Maronti Bay (Italy – Mediterranean) – Po (? m)

OVERALL: Net = –7 m;   Rate = –16.43 % yr–1; Perc Final = 72 % > decrease

DECADAL: NO (2 yr)

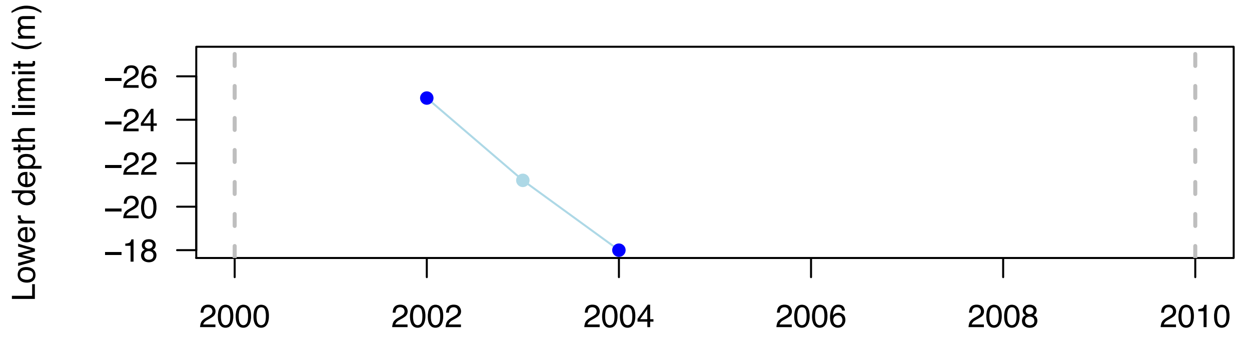

342_upperlimit

Gambi et al. 2005

SITE: Maronti Bay (Italy – Mediterranean) – Po (? m)

OVERALL: Net = 0 m;   Rate = 0 % yr–1; Perc Final = 100 % > no change

DECADAL: NO (2 yr)

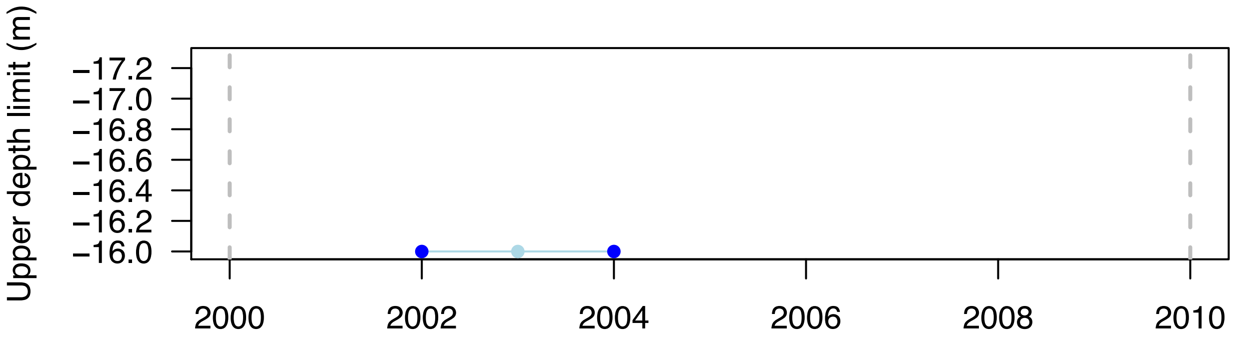

343_area

Barsanti et al. 2007

SITE: Tigullio Gulf (all sectors) (Italy – Mediterranean) – Cn (? m)

OVERALL: Net = –127.7 ha;   Rate = –3.76 % yr–1; Perc Final = 69 % > decrease

DECADAL: YES (10 yr)

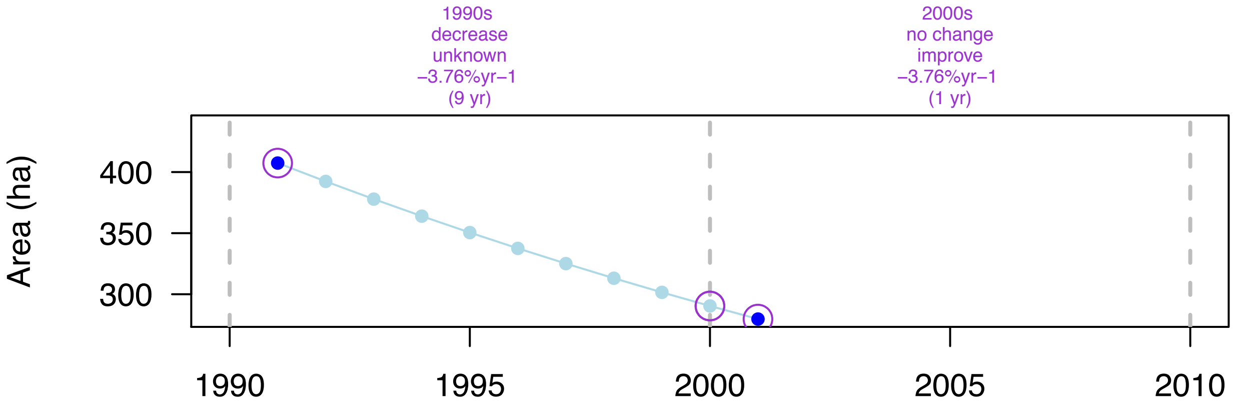

344_area

Badalamenti et al. 2006, 2011

SITE: Cabo Feto (Italy – Mediterranean) – Po (? m)

OVERALL: Net = −54.98 ha; Rate = −8.98 % yr−1; Perc Final = 24 % > decrease

DECADAL: YES (16 yr)

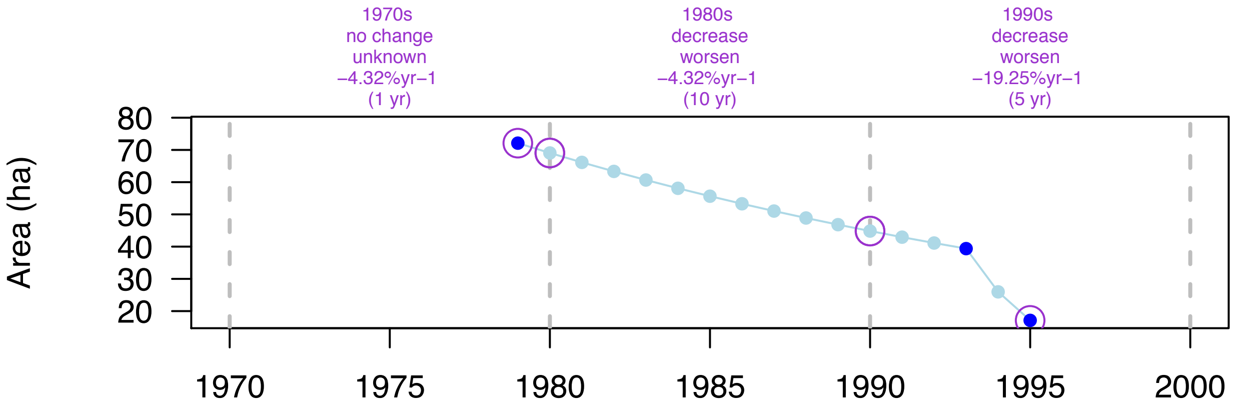

345_area

Badalamenti et al. 2006, 2011

SITE: Cabo Feto (Italy – Mediterranean) – Cn (? m)

OVERALL: Net = 4.86 ha; Rate = NA % yr−1; Perc Final = NA % > increase

DECADAL: YES (16 yr)

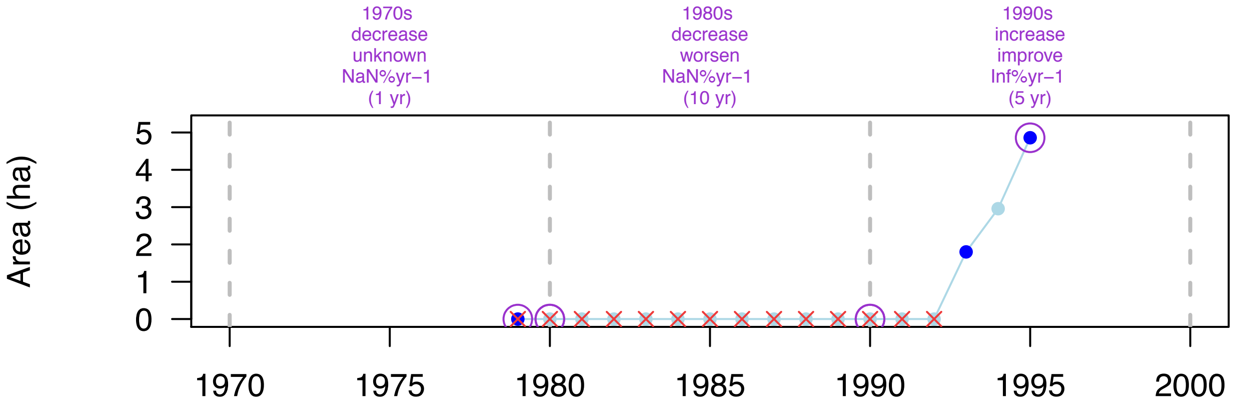

346_area

Badalamenti et al. 2006, 2011

SITE: Cabo Feto (Italy – Mediterranean) – Po (? m)

OVERALL: Net = −26.21 ha; Rate = −2.89 % yr−1; Perc Final = 63 % > decrease

DECADAL: YES (16 yr)

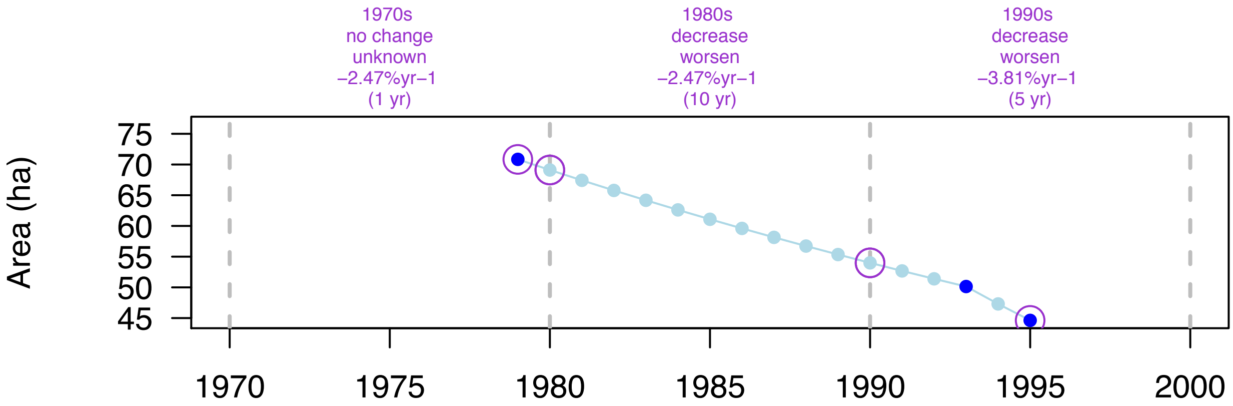

347_cover

Peirano et al. 2011

SITE: Monterosso al Mare (Italy – Mediterranean) – Po (−10 m)

OVERALL: Net = 22.4 %; Rate = 2.46 % yr−1; Perc Final = 141 % > increase

DECADAL: YES (14 yr)

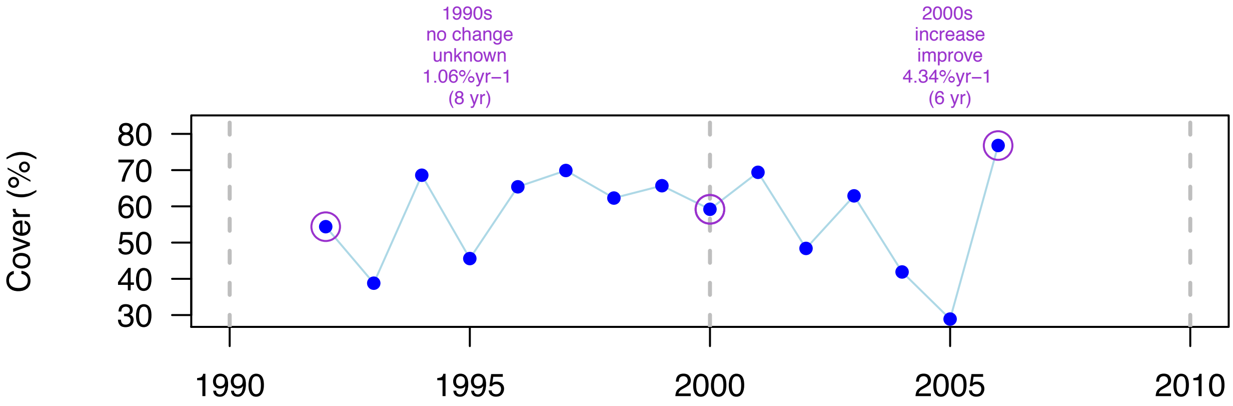

347_density

Peirano et al. 2011

SITE: Monterosso al Mare (Italy – Mediterranean) – Po (–10 m)

OVERALL: Net = −129.5 shoot m−2;   Rate = −3.4 % yr−1; Perc Final = 62 % > decrease

DECADAL: YES (14 yr)

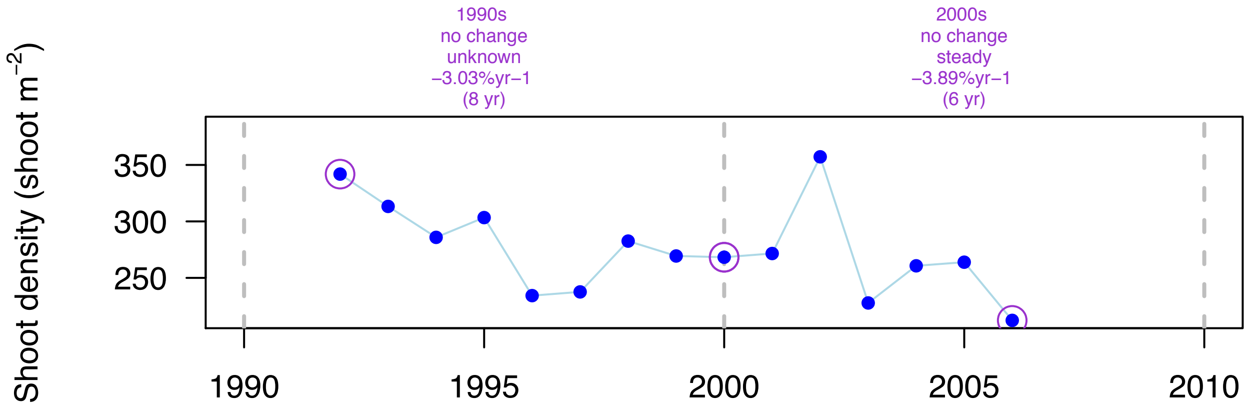

348_cover

Peirano et al. 2011

SITE: Monterosso al Mare (Italy – Mediterranean) – Po (–17 m)

OVERALL: Net = 30 %;   Rate = 5.56 % yr−1; Perc Final = 218 % > increase

DECADAL: YES (14 yr)

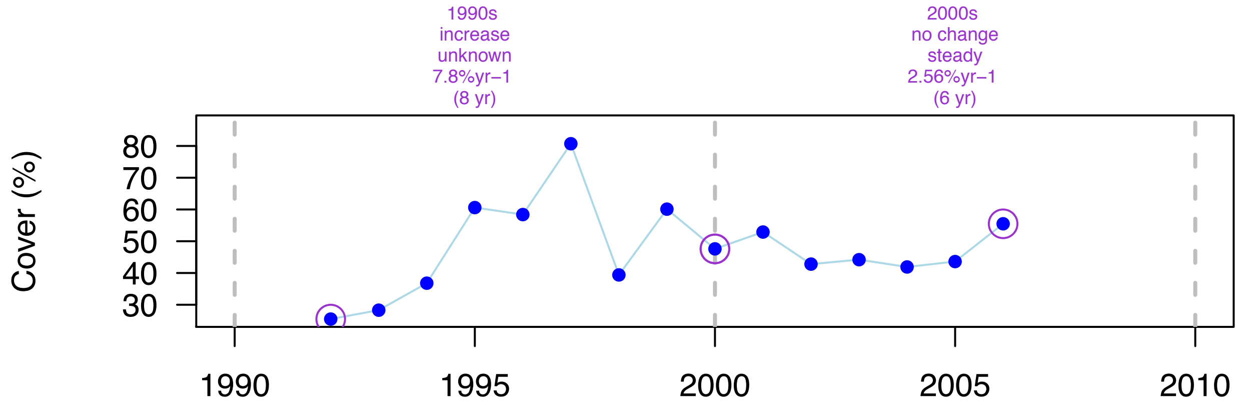

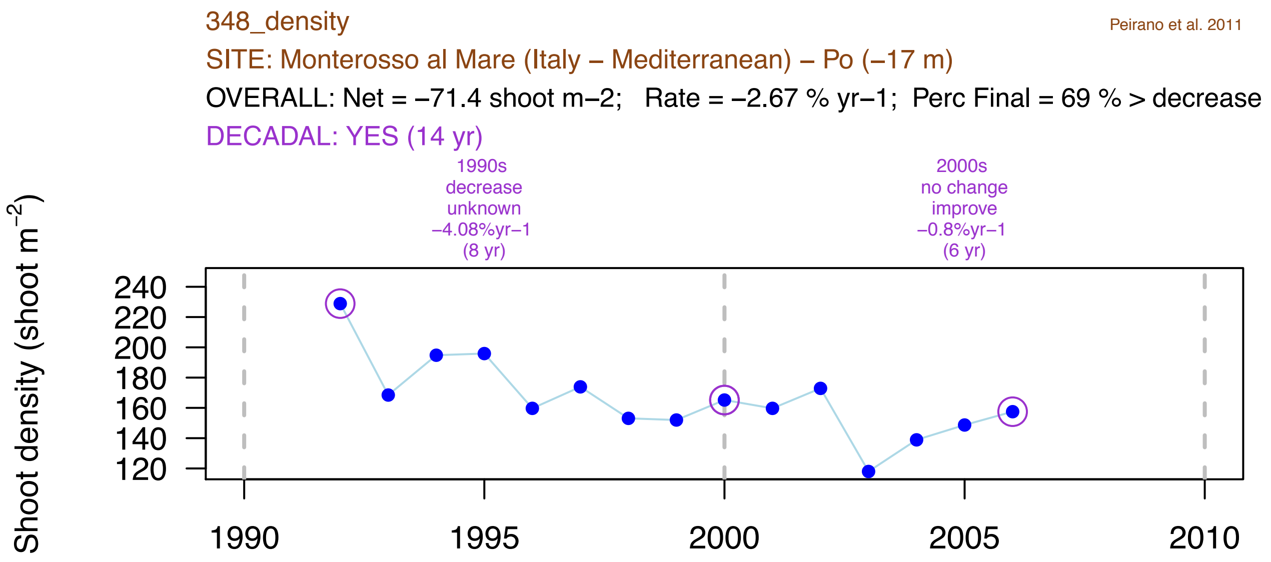
348_density
Peirano et al. 2011
SITE: Monterosso al Mare (Italy – Mediterranean) – Po (–17 m)
OVERALL: Net = –71.4 shoot m–2; Rate = –2.67 % yr–1; Perc Final = 69 % > decrease
DECADAL: YES (14 yr)
1990s
decrease
unknown
–4.08%yr–1
(8 yr)
2000s
no change
improve
–0.8%yr–1
(6 yr)
Shoot density (shoot m-2)
240
220
200
180
160
140
120
1990
1995
2000
2005
2010

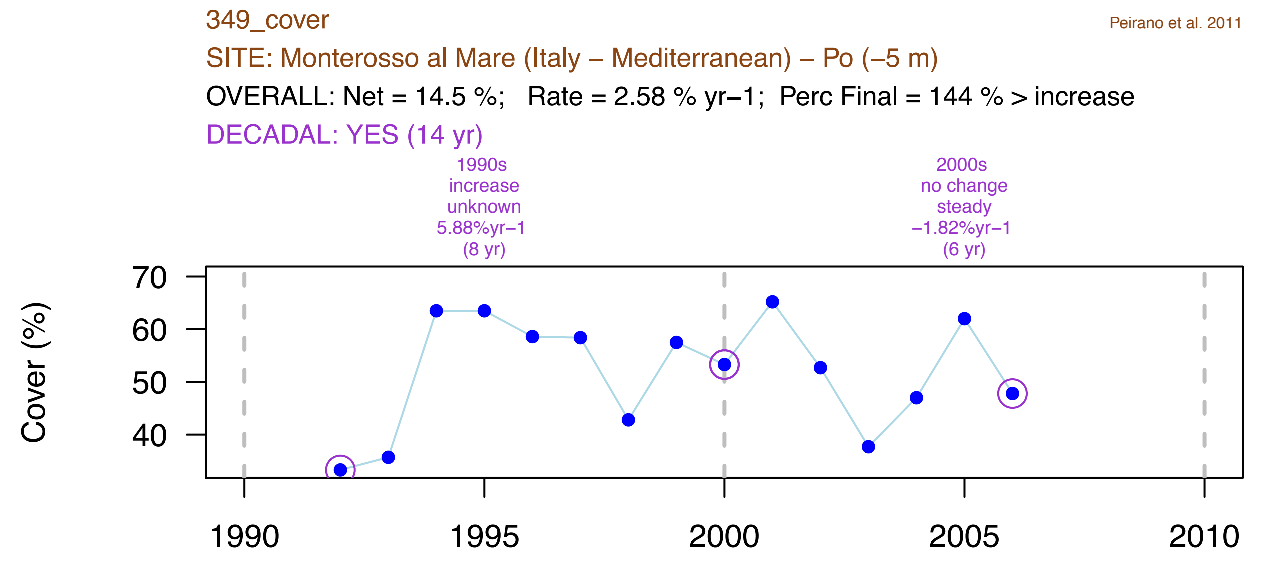
349_cover
Peirano et al. 2011
SITE: Monterosso al Mare (Italy – Mediterranean) – Po (–5 m)
OVERALL: Net = 14.5 %; Rate = 2.58 % yr–1; Perc Final = 144 % > increase
DECADAL: YES (14 yr)
1990s
increase
unknown
5.88%yr–1
(8 yr)
2000s
no change
steady
–1.82%yr–1
(6 yr)
Cover (%)
70
60
50
40
1990
1995
2000
2005
2010

349_density

Peirano et al. 2011

SITE: Monterosso al Mare (Italy – Mediterranean) – Po (–5 m)

OVERALL: Net = –63.7 shoot m–2;   Rate = –1.15 % yr–1; Perc Final = 85 % > no change

DECADAL: YES (14 yr)

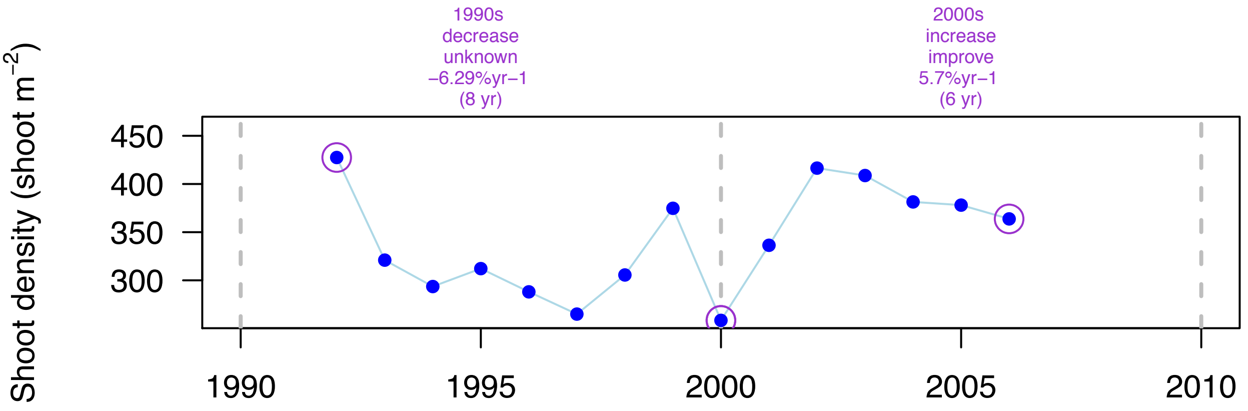

350_area

Giovani et al. 2010

SITE: Orbetello lagoon (Italy – Mediterranean) – Zn (–1.2 m)

OVERALL: Net = 0.36 ha;   Rate = 7.63 % yr–1; Perc Final = 158 % > increase

DECADAL: NO (6 yr)

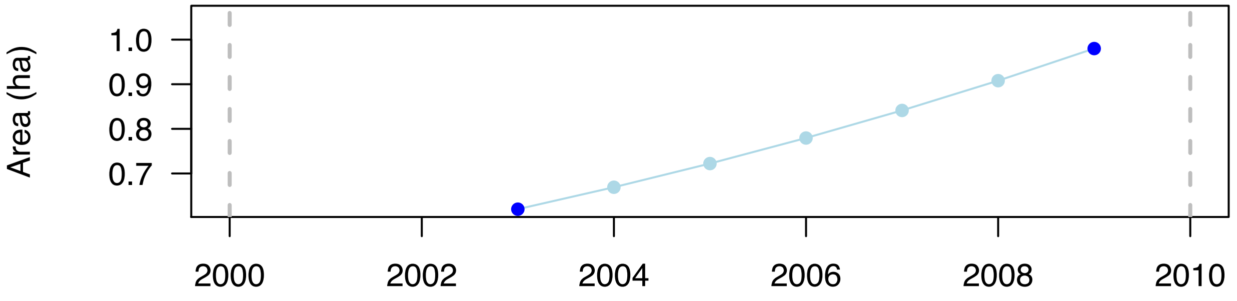

351_area

Giovani et al. 2010

SITE: Orbetello lagoon (Italy – Mediterranean) – Cn (–1.2 m)

OVERALL: Net = –0.29 ha;   Rate = –12.16 % yr–1; Perc Final = 48 % > decrease

DECADAL: NO (6 yr)

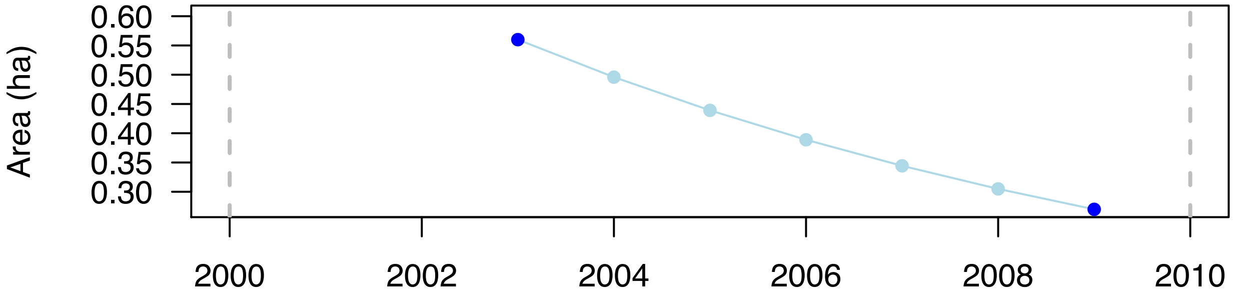

352_lowerlimit

Falconetti and Meinesz 1989

SITE: Larvotto Beach (Monaco – Mediterranean) – Po (? m)

OVERALL: Net = –9 m;   Rate = –4.85 % yr–1; Perc Final = 68 % > decrease

DECADAL: YES (8 yr)

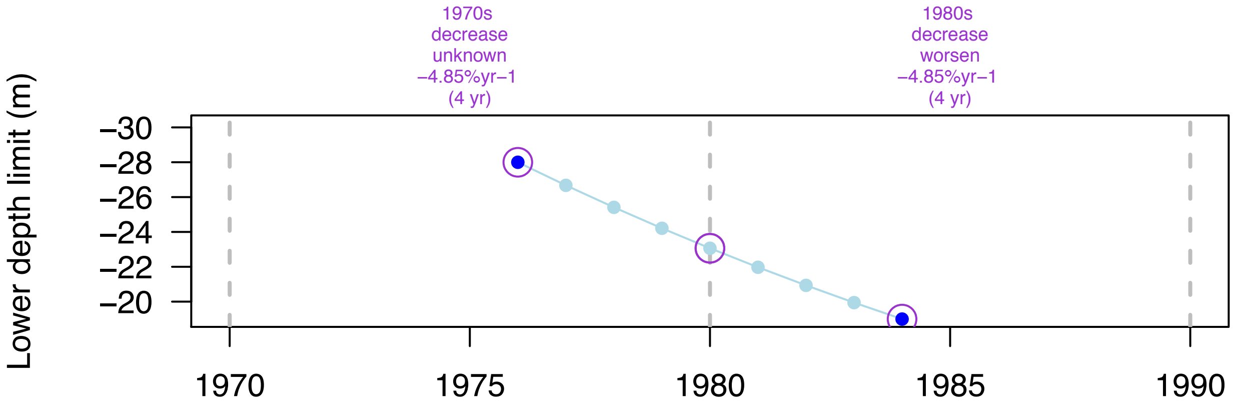

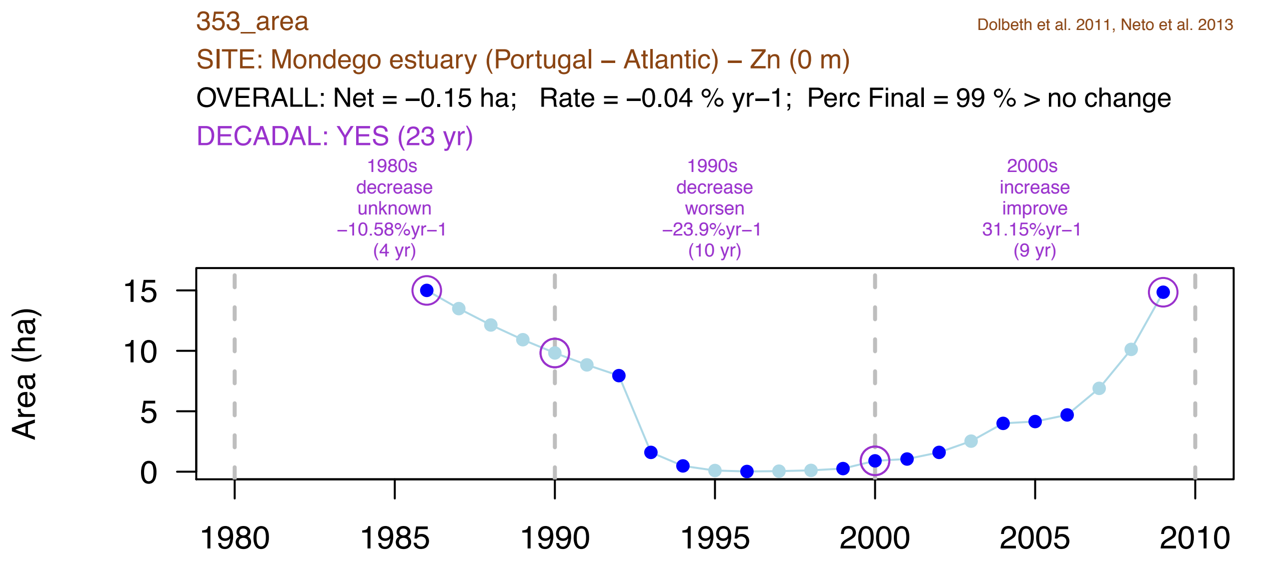
353_area
Dolbeth et al. 2011, Neto et al. 2013
SITE: Mondego estuary (Portugal – Atlantic) – Zn (0 m)
OVERALL: Net = –0.15 ha; Rate = –0.04 % yr–1; Perc Final = 99 % > no change
DECADAL: YES (23 yr)
1980s
decrease
unknown
–10.58%yr–1
(4 yr)
1990s
decrease
worsen
–23.9%yr–1
(10 yr)
2000s
increase
improve
31.15%yr–1
(9 yr)
Area (ha)
15
10
5
0
1980
1985
1990
1995
2000
2005
2010

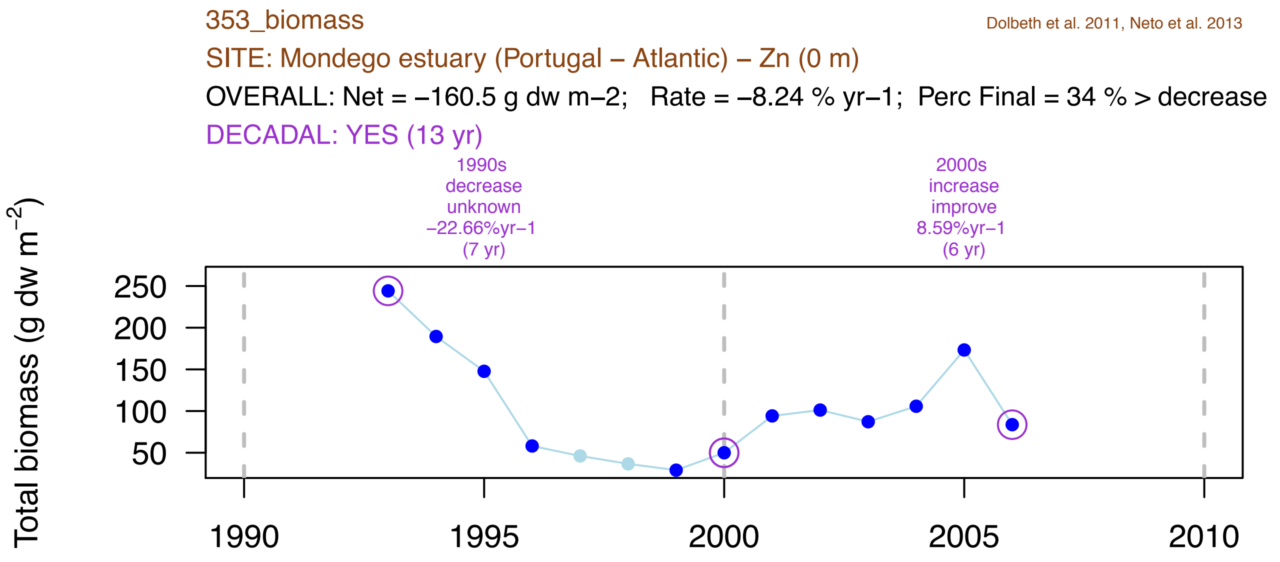
353_biomass
Dolbeth et al. 2011, Neto et al. 2013
SITE: Mondego estuary (Portugal – Atlantic) – Zn (0 m)
OVERALL: Net = –160.5 g dw m–2; Rate = –8.24 % yr–1; Perc Final = 34 % > decrease
DECADAL: YES (13 yr)
1990s
decrease
unknown
–22.66%yr–1
(7 yr)
2000s
increase
improve
8.59%yr–1
(6 yr)
Total biomass (g dw m–2)
250
200
150
100
50
1990
1995
2000
2005
2010

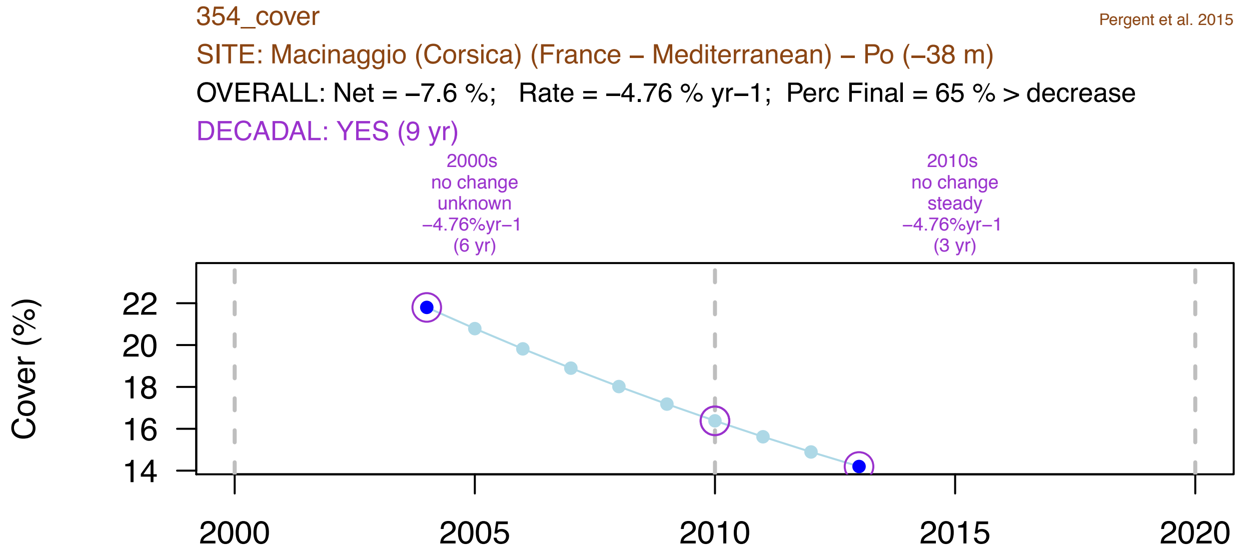
354_cover
Pergent et al. 2015
SITE: Macinaggio (Corsica) (France – Mediterranean) – Po (–38 m)
OVERALL: Net = –7.6 %; Rate = –4.76 % yr–1; Perc Final = 65 % > decrease
DECADAL: YES (9 yr)
2000s
no change
unknown
–4.76%yr–1
(6 yr)
2010s
no change
steady
–4.76%yr–1
(3 yr)
Cover (%)
22
20
18
16
14
2000
2005
2010
2015
2020

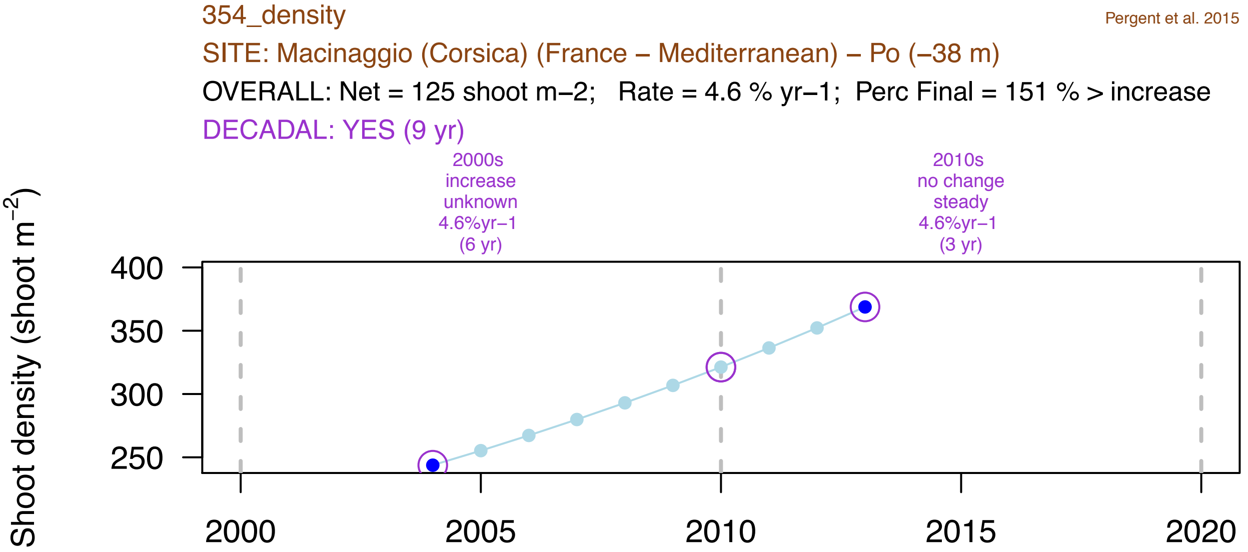
354_density
Pergent et al. 2015
SITE: Macinaggio (Corsica) (France – Mediterranean) – Po (–38 m)
OVERALL: Net = 125 shoot m–2; Rate = 4.6 % yr–1; Perc Final = 151 % > increase
DECADAL: YES (9 yr)
2000s
increase
unknown
4.6%yr–1
(6 yr)
2010s
no change
steady
4.6%yr–1
(3 yr)
Shoot density (shoot m^{-2})
400
350
300
250
2000
2005
2010
2015
2020

354_lowerlimit

Pergent et al. 2015

SITE: Macinaggio (Corsica) (France – Mediterranean) – Po (–38 m)

OVERALL: Net = 0.1 m;   Rate = 0.03 % yr–1; Perc Final = 100 % > no change

DECADAL: YES (9 yr)

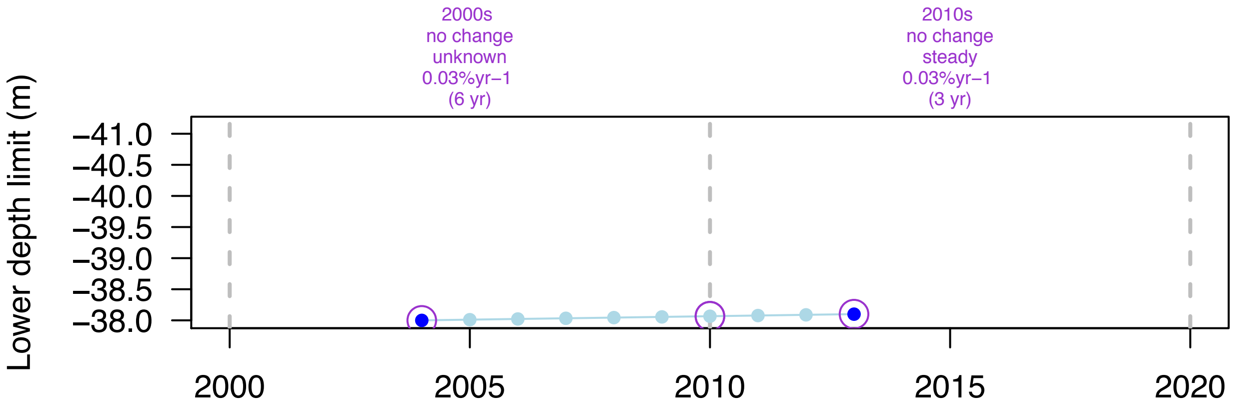

355_cover

Pergent et al. 2015

SITE: Cap Sagro (Corsica) (France – Mediterranean) – Po (–33 m)

OVERALL: Net = –7.5 %;   Rate = –9.33 % yr–1; Perc Final = 43 % > decrease

DECADAL: YES (9 yr)

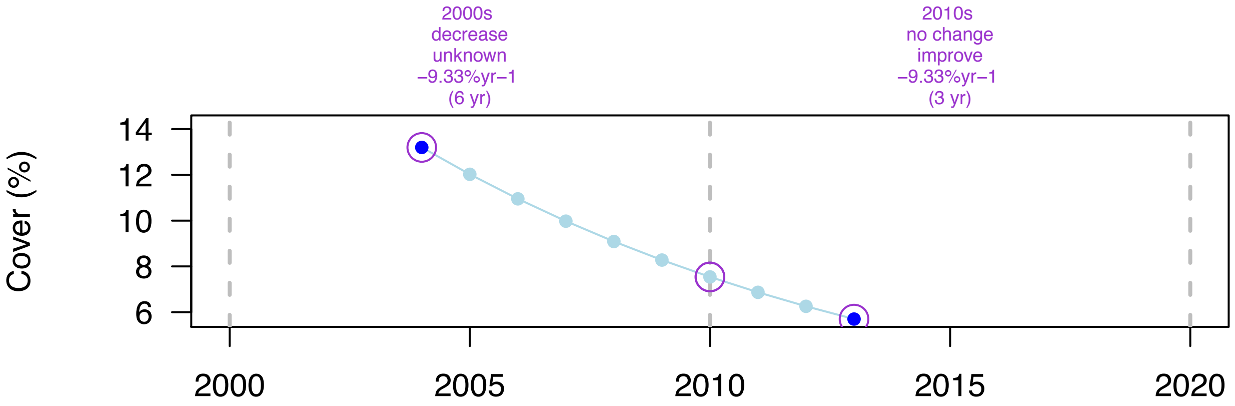

355_density

Pergent et al. 2015

SITE: Cap Sagro (Corsica) (France – Mediterranean) – Po (–33 m)

OVERALL: Net = 36.8 shoot m–2;   Rate = 1.42 % yr–1; Perc Final = 114 % > no change

DECADAL: YES (9 yr)

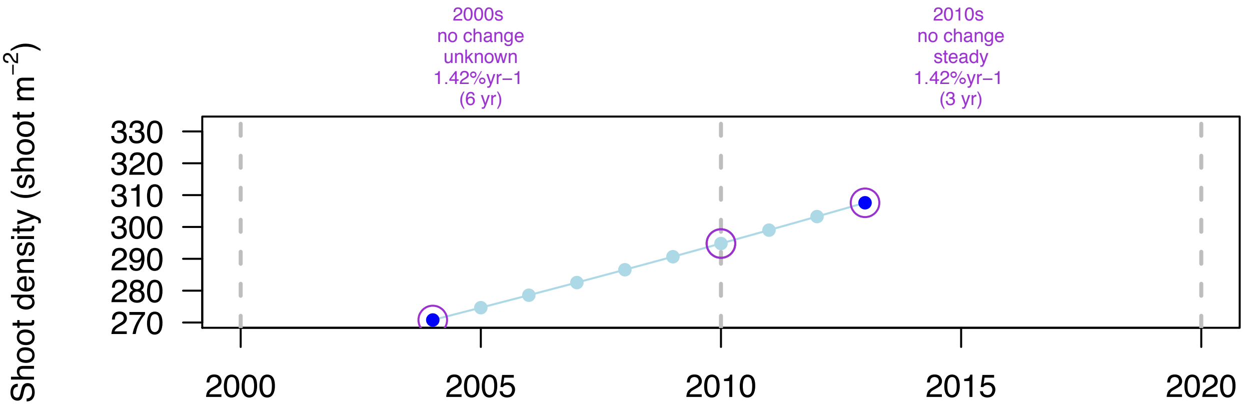

355_lowerlimit

Pergent et al. 2015

SITE: Cap Sagro (Corsica) (France – Mediterranean) – Po (–33 m)

OVERALL: Net = 0 m;   Rate = 0 % yr–1; Perc Final = 100 % > no change

DECADAL: YES (9 yr)

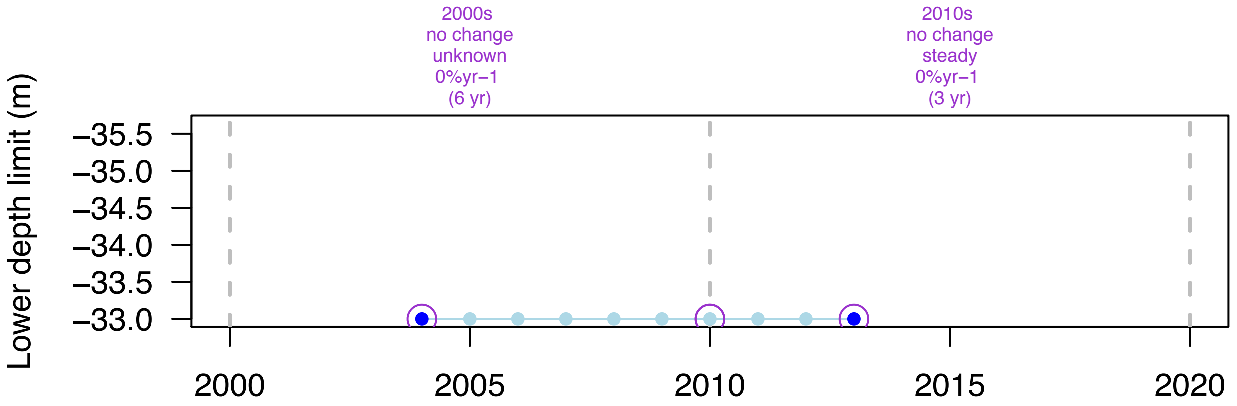

356_cover

Pergent et al. 2015

SITE: Toga (Corsica) (France – Mediterranean) – Po (–24 m)

OVERALL: Net = 22.1 %;   Rate = 8.54 % yr–1; Perc Final = 216 % > increase

DECADAL: YES (9 yr)

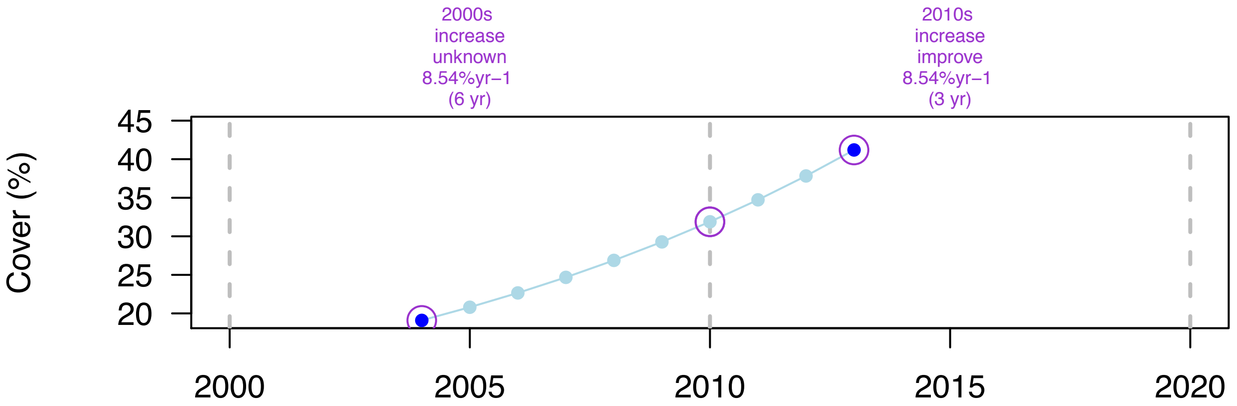

356_density

Pergent et al. 2015

SITE: Toga (Corsica) (France – Mediterranean) – Po (–24 m)

OVERALL: Net = 37.7 shoot m–2;   Rate = 1.68 % yr–1; Perc Final = 116 % > no change

DECADAL: YES (9 yr)

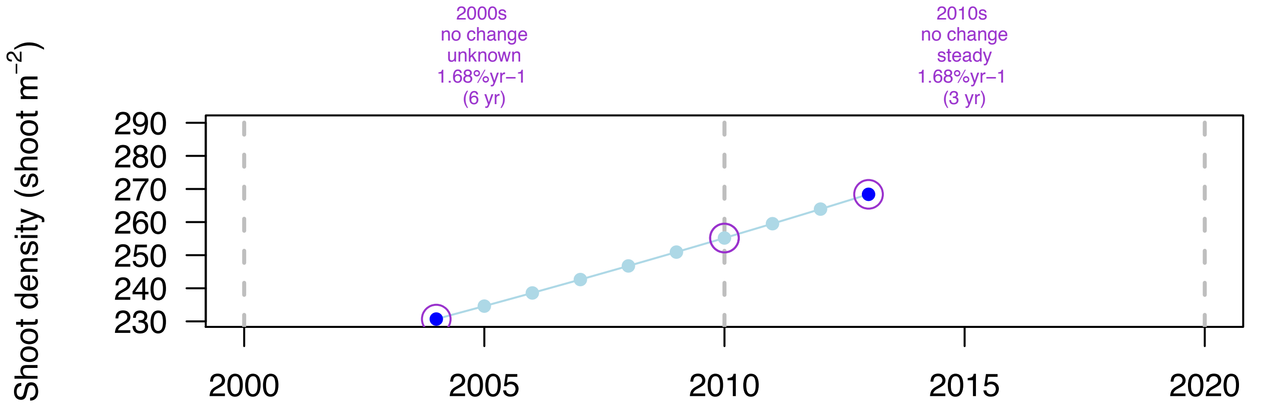

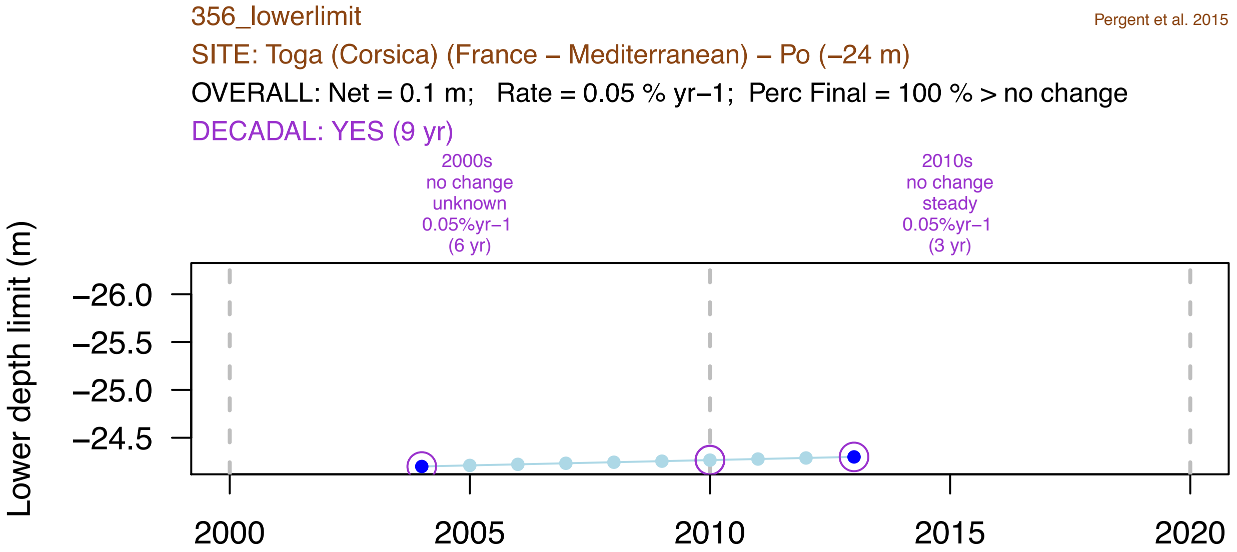
356_lowerlimit
Pergent et al. 2015
SITE: Toga (Corsica) (France – Mediterranean) – Po (–24 m)
OVERALL: Net = 0.1 m; Rate = 0.05 % yr–1; Perc Final = 100 % > no change
DECADAL: YES (9 yr)
2000s
no change
unknown
0.05%yr–1
(6 yr)
2010s
no change
steady
0.05%yr–1
(3 yr)
Lower depth limit (m)
–26.0
–25.5
–25.0
–24.5
2000
2005
2010
2015
2020

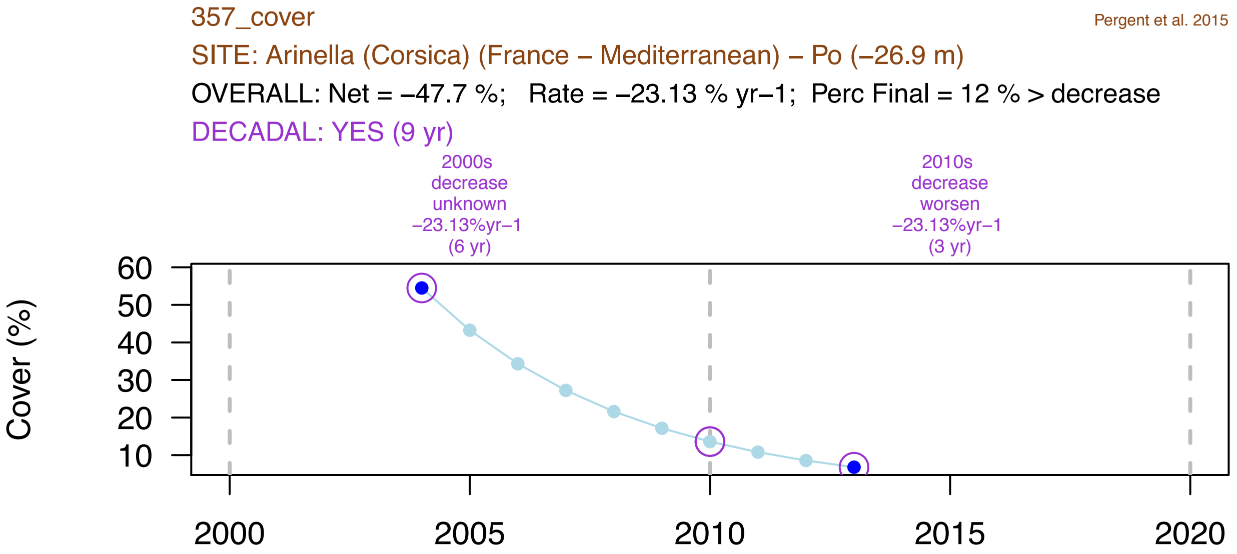
357_cover
Pergent et al. 2015
SITE: Arinella (Corsica) (France – Mediterranean) – Po (–26.9 m)
OVERALL: Net = –47.7 %; Rate = –23.13 % yr–1; Perc Final = 12 % > decrease
DECADAL: YES (9 yr)
2000s
decrease
unknown
–23.13%yr–1
(6 yr)
2010s
decrease
worsen
–23.13%yr–1
(3 yr)
Cover (%)
60
50
40
30
20
10
2000
2005
2010
2015
2020

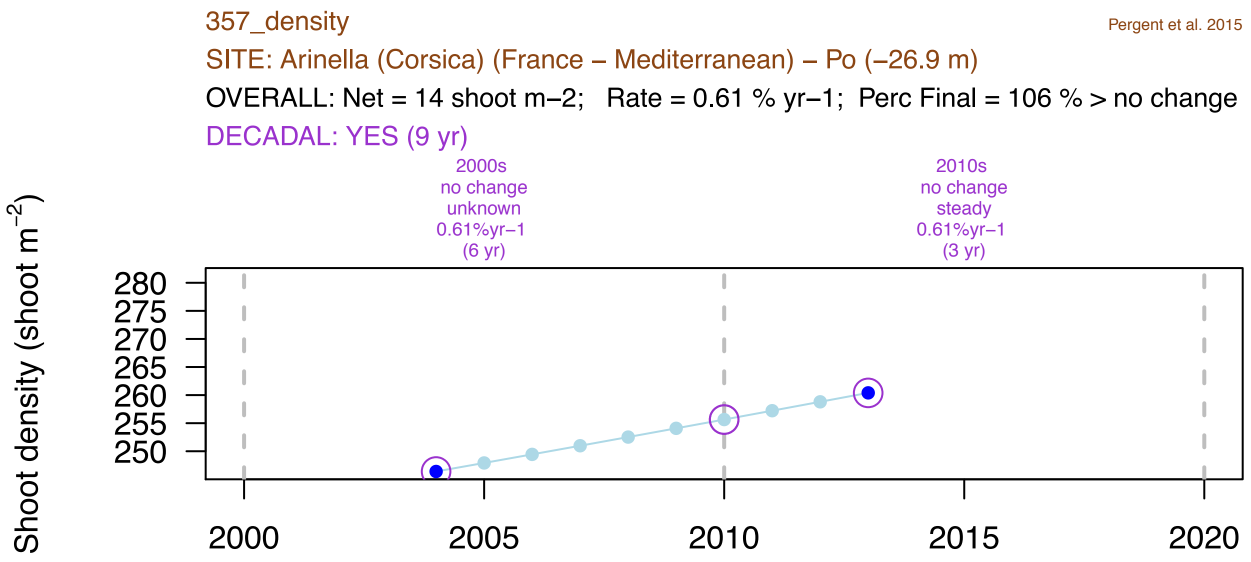
357_density
Pergent et al. 2015
SITE: Arinella (Corsica) (France – Mediterranean) – Po (–26.9 m)
OVERALL: Net = 14 shoot m–2; Rate = 0.61 % yr–1; Perc Final = 106 % > no change
DECADAL: YES (9 yr)
2000s
no change
unknown
0.61%yr–1
(6 yr)
2010s
no change
steady
0.61%yr–1
(3 yr)
Shoot density (shoot m^{-2})
280
275
270
265
260
255
250
2000
2005
2010
2015
2020

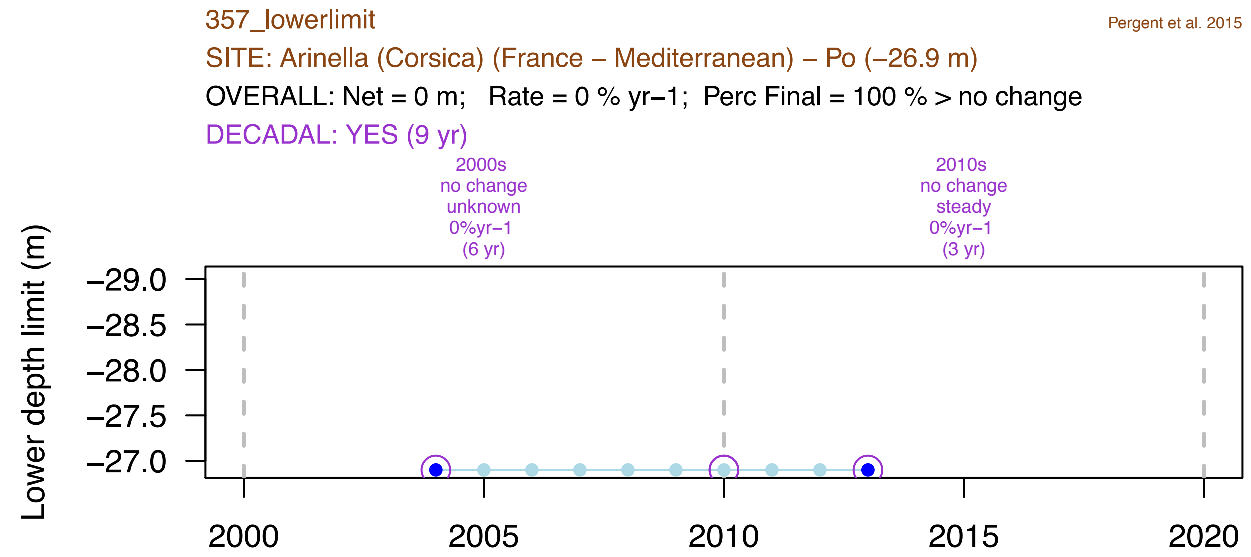
357_lowerlimit
Pergent et al. 2015
SITE: Arinella (Corsica) (France – Mediterranean) – Po (–26.9 m)
OVERALL: Net = 0 m; Rate = 0 % yr–1; Perc Final = 100 % > no change
DECADAL: YES (9 yr)
2000s
no change
unknown
0%yr–1
(6 yr)
2010s
no change
steady
0%yr–1
(3 yr)
Lower depth limit (m)
−29.0
−28.5
−28.0
−27.5
−27.0
2000
2005
2010
2015
2020

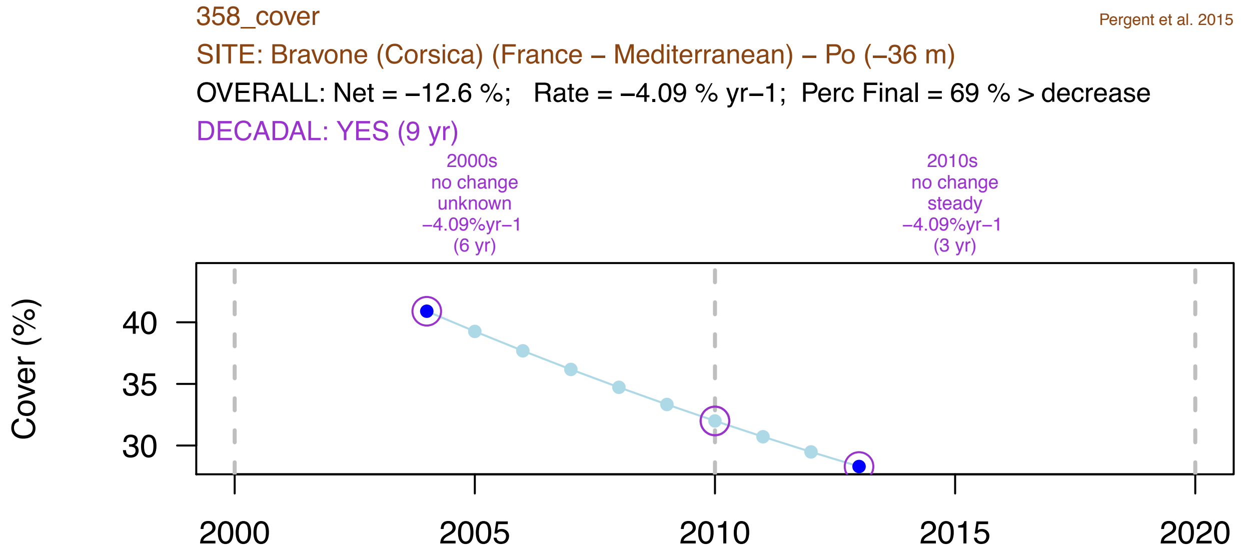
358_cover
Pergent et al. 2015
SITE: Bravone (Corsica) (France – Mediterranean) – Po (–36 m)
OVERALL: Net = –12.6 %; Rate = –4.09 % yr–1; Perc Final = 69 % > decrease
DECADAL: YES (9 yr)
2000s
no change
unknown
–4.09%yr–1
(6 yr)
2010s
no change
steady
–4.09%yr–1
(3 yr)
Cover (%)
40
35
30
2000
2005
2010
2015
2020

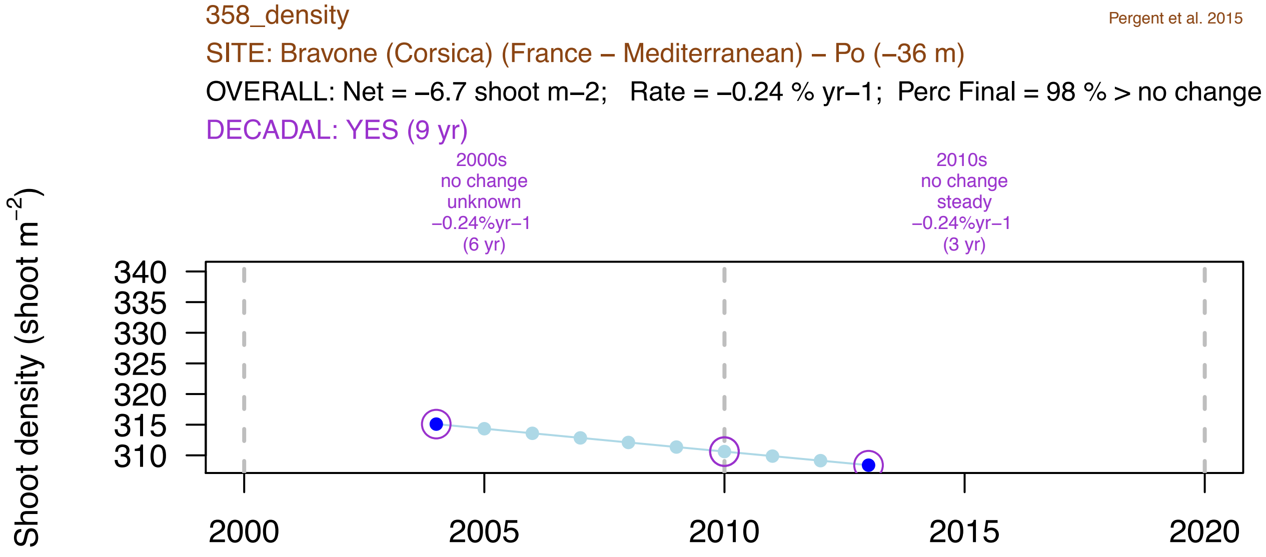
358_density
Pergent et al. 2015
SITE: Bravone (Corsica) (France – Mediterranean) – Po (–36 m)
OVERALL: Net = –6.7 shoot m–2; Rate = –0.24 % yr–1; Perc Final = 98 % > no change
DECADAL: YES (9 yr)
2000s
no change
unknown
–0.24%yr–1
(6 yr)
2010s
no change
steady
–0.24%yr–1
(3 yr)
Shoot density (shoot m⁻²)
340
335
330
325
320
315
310
2000
2005
2010
2015
2020

358_lowerlimit

Pergent et al. 2015

SITE: Bravone (Corsica) (France – Mediterranean) – Po (–36 m)

OVERALL: Net = –0.09 m;   Rate = –0.03 % yr–1; Perc Final = 100 % > no change

DECADAL: YES (9 yr)

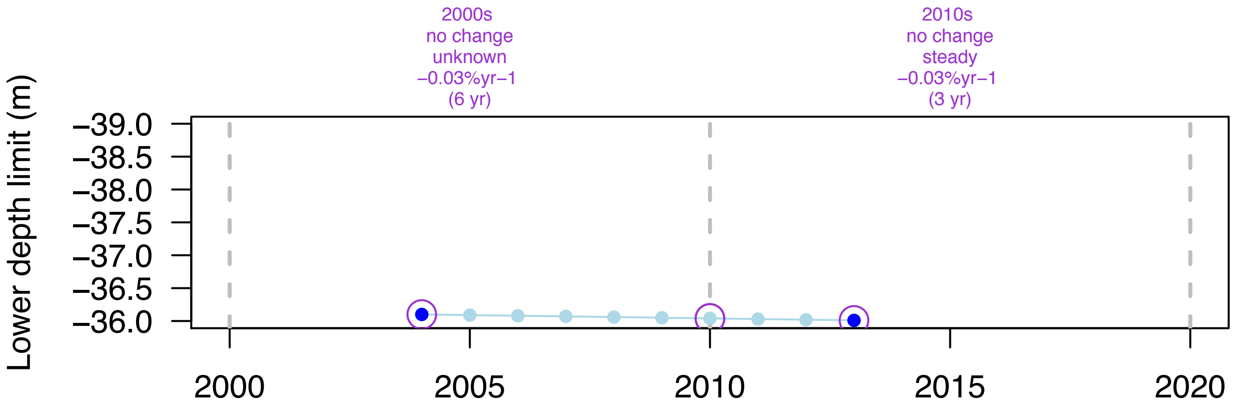

359_cover

Pergent et al. 2015

SITE: Favone (Corsica) (France – Mediterranean) – Po (–36.9 m)

OVERALL: Net = –0.1 %;   Rate = –0.16 % yr–1; Perc Final = 99 % > no change

DECADAL: YES (8 yr)

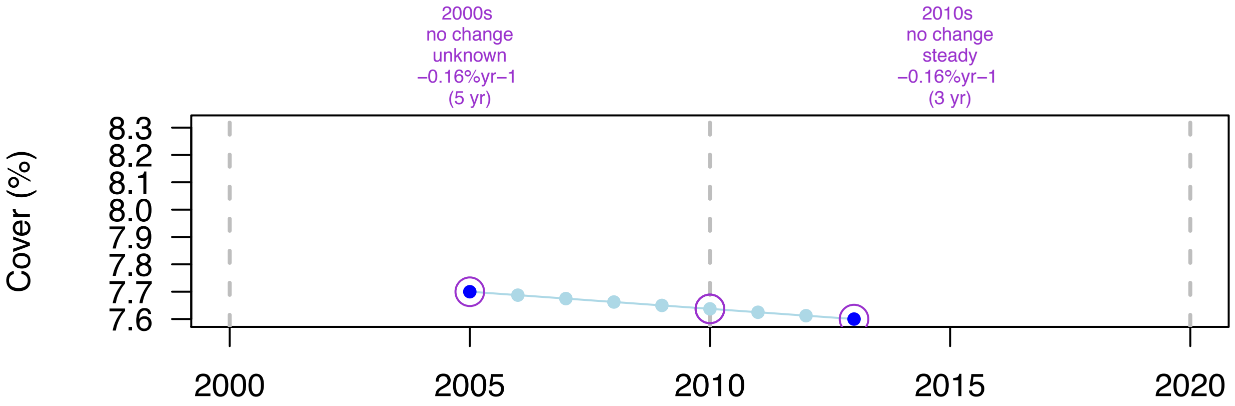

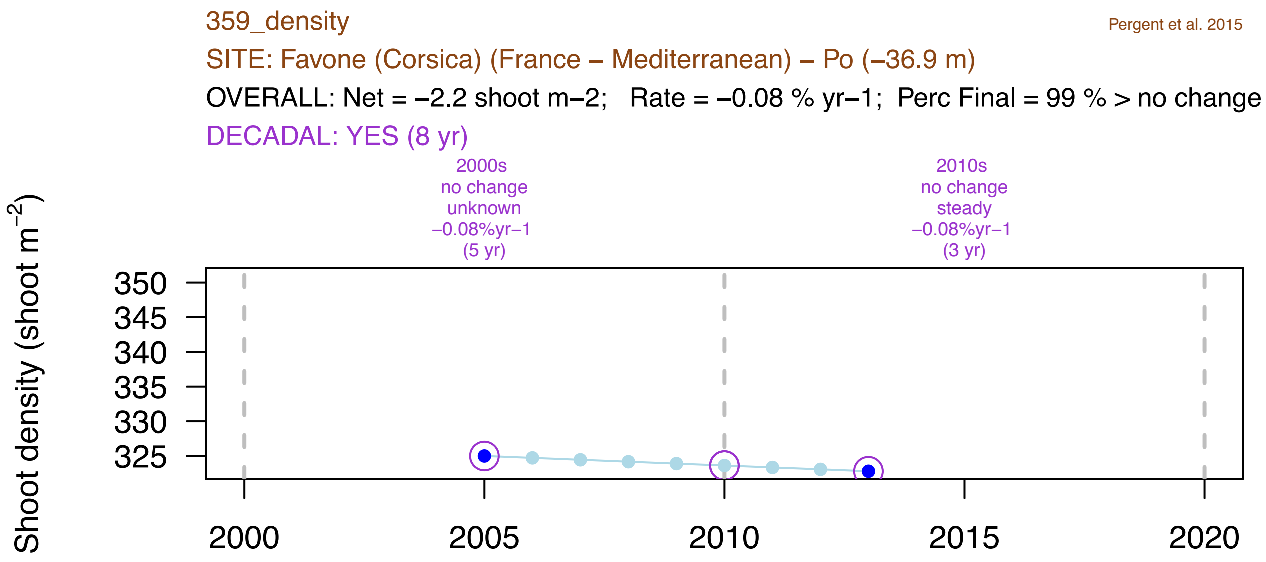
359_density
Pergent et al. 2015
SITE: Favone (Corsica) (France – Mediterranean) – Po (–36.9 m)
OVERALL: Net = –2.2 shoot m–2; Rate = –0.08 % yr–1; Perc Final = 99 % > no change
DECADAL: YES (8 yr)
2000s
no change
unknown
–0.08%yr–1
(5 yr)
2010s
no change
steady
–0.08%yr–1
(3 yr)
Shoot density (shoot m–2)
350
345
340
335
330
325
2000
2005
2010
2015
2020

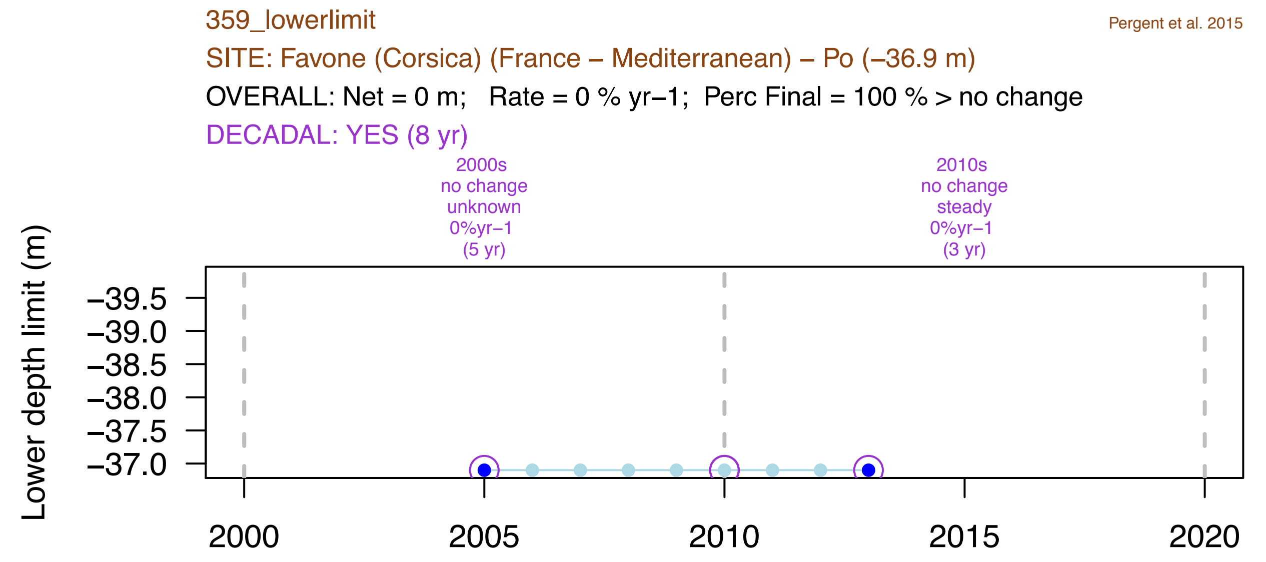
359_lowerlimit
Pergent et al. 2015
SITE: Favone (Corsica) (France – Mediterranean) – Po (–36.9 m)
OVERALL: Net = 0 m; Rate = 0 % yr–1; Perc Final = 100 % > no change
DECADAL: YES (8 yr)
2000s
no change
unknown
0%yr–1
(5 yr)
2010s
no change
steady
0%yr–1
(3 yr)
Lower depth limit (m)
–39.5
–39.0
–38.5
–38.0
–37.5
–37.0
2000
2005
2010
2015
2020

360_cover

Pergent et al. 2015

SITE: La Chiappa (Corsica) (France – Mediterranean) – Po (–35.3 m)

OVERALL: Net = –5.2 %; Rate = –4.35 % yr–1; Perc Final = 71 % > decrease

DECADAL: YES (8 yr)

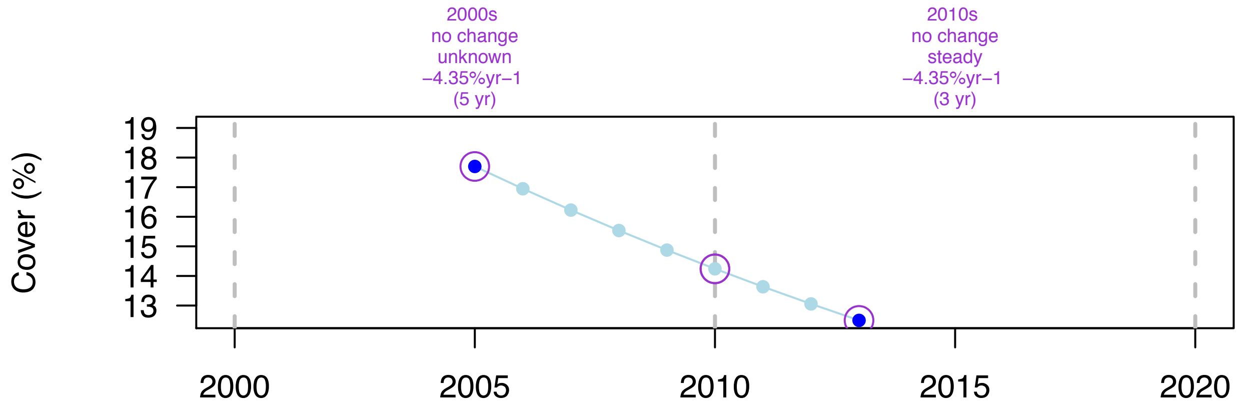

360_density

Pergent et al. 2015

SITE: La Chiappa (Corsica) (France – Mediterranean) – Po (–35.3 m)

OVERALL: Net = 14 shoot m–2; Rate = 0.56 % yr–1; Perc Final = 105 % > no change

DECADAL: YES (8 yr)

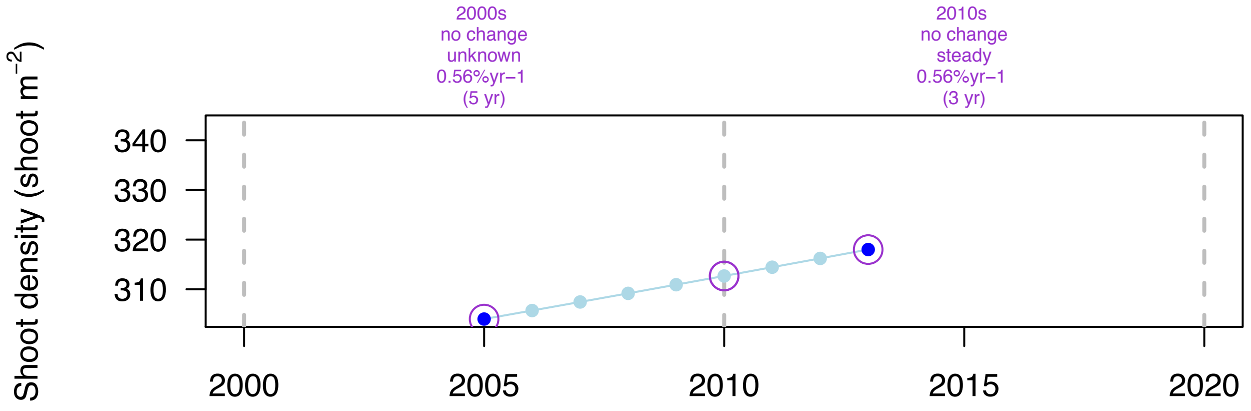

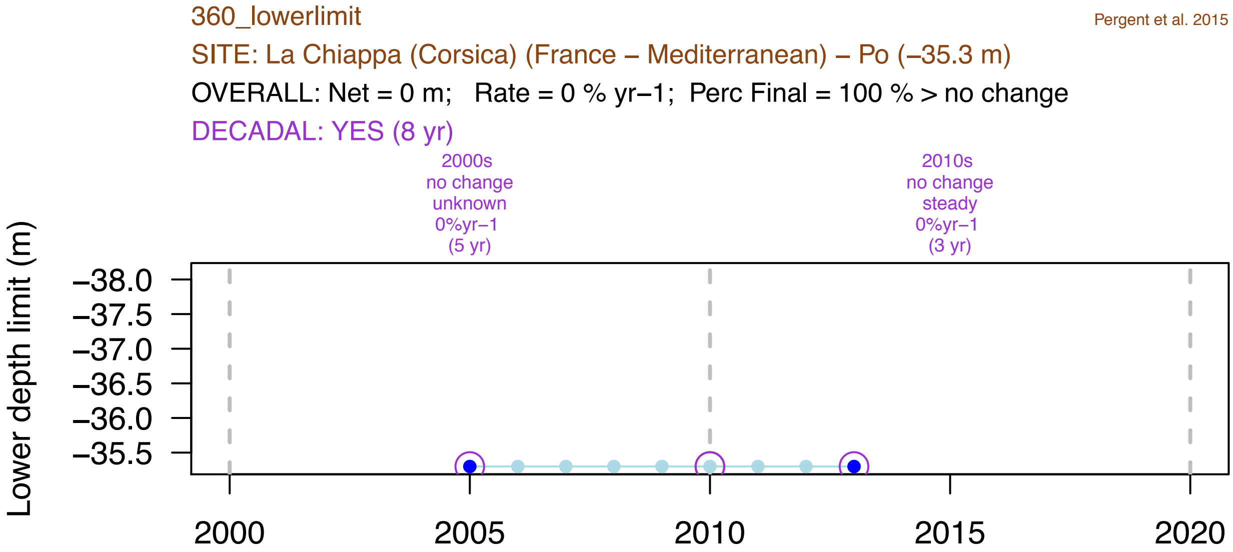
360_lowerlimit
Pergent et al. 2015
SITE: La Chiappa (Corsica) (France – Mediterranean) – Po (–35.3 m)
OVERALL: Net = 0 m; Rate = 0 % yr–1; Perc Final = 100 % > no change
DECADAL: YES (8 yr)
2000s
no change
unknown
0%yr–1
(5 yr)
2010s
no change
steady
0%yr–1
(3 yr)
Lower depth limit (m)
–38.0
–37.5
–37.0
–36.5
–36.0
–35.5
2000
2005
2010
2015
2020

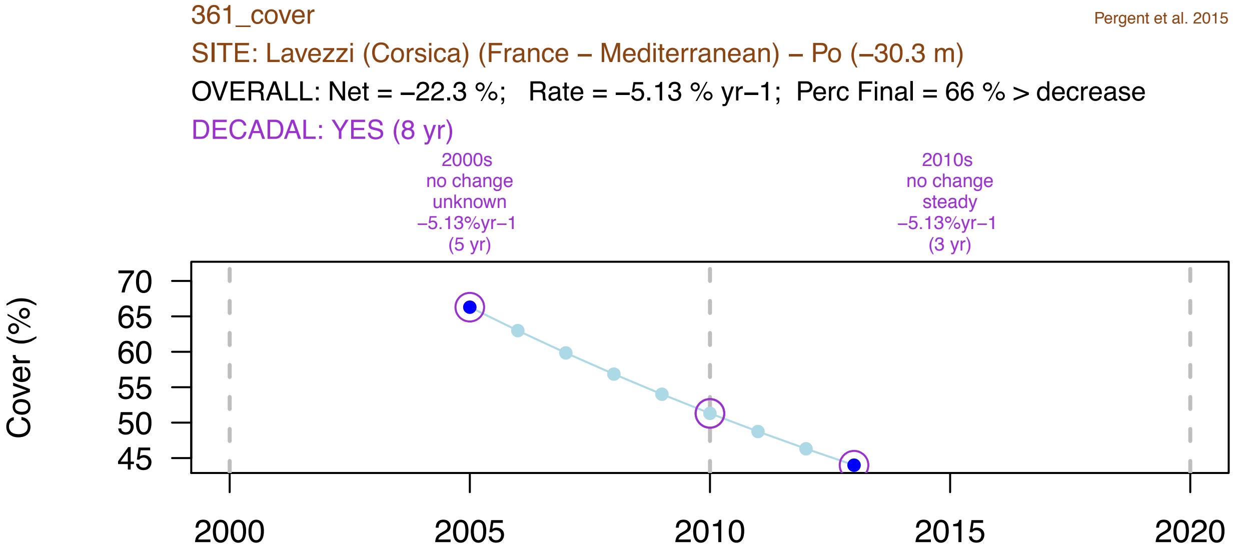
361_cover
Pergent et al. 2015
SITE: Lavezzi (Corsica) (France – Mediterranean) – Po (–30.3 m)
OVERALL: Net = –22.3 %; Rate = –5.13 % yr–1; Perc Final = 66 % > decrease
DECADAL: YES (8 yr)
2000s
no change
unknown
–5.13%yr–1
(5 yr)
2010s
no change
steady
–5.13%yr–1
(3 yr)
Cover (%)
70
65
60
55
50
45
2000
2005
2010
2015
2020

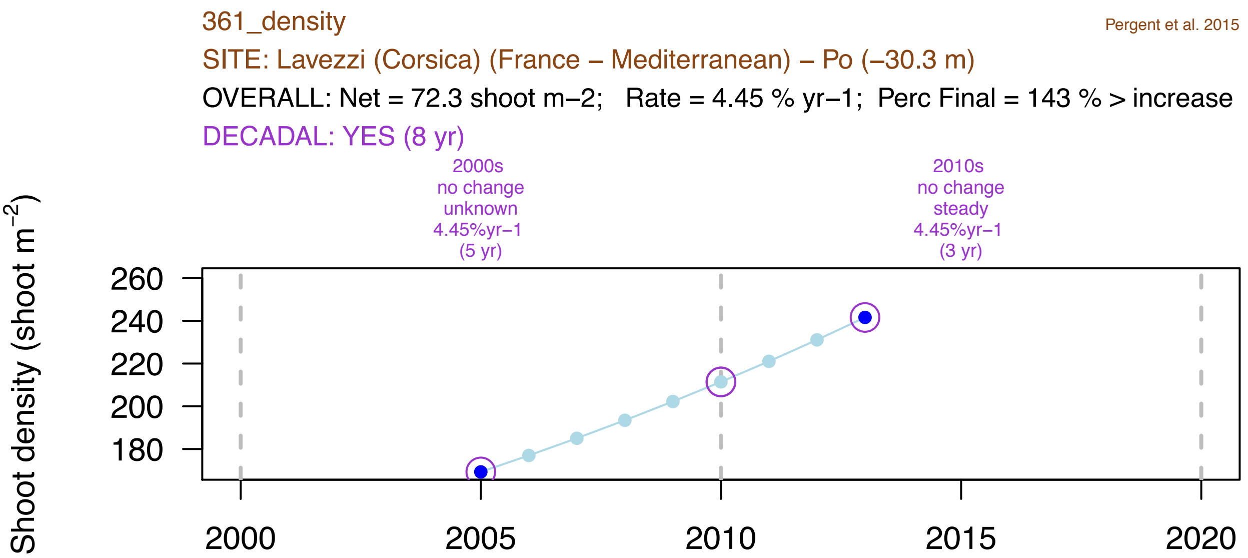
361_density
Pergent et al. 2015
SITE: Lavezzi (Corsica) (France – Mediterranean) – Po (–30.3 m)
OVERALL: Net = 72.3 shoot m–2; Rate = 4.45 % yr–1; Perc Final = 143 % > increase
DECADAL: YES (8 yr)
2000s
no change
unknown
4.45%yr–1
(5 yr)
2010s
no change
steady
4.45%yr–1
(3 yr)
Shoot density (shoot m–2)
260
240
220
200
180
2000
2005
2010
2015
2020

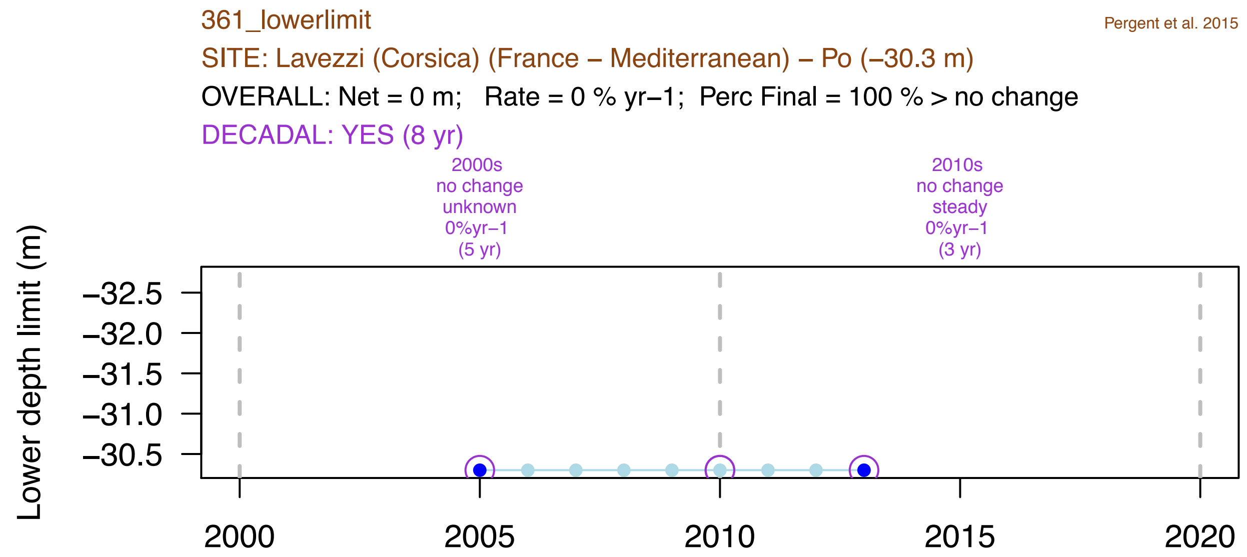
361_lowerlimit
Pergent et al. 2015
SITE: Lavezzi (Corsica) (France – Mediterranean) – Po (–30.3 m)
OVERALL: Net = 0 m; Rate = 0 % yr–1; Perc Final = 100 % > no change
DECADAL: YES (8 yr)
2000s
no change
unknown
0%yr–1
(5 yr)
2010s
no change
steady
0%yr–1
(3 yr)
Lower depth limit (m)
–32.5
–32.0
–31.5
–31.0
–30.5
2000
2005
2010
2015
2020

362_cover

Pergent et al. 2015

SITE: Porto Polo (Corsica) (France – Mediterranean) – Po (–36.5 m)

OVERALL: Net = –27.2 %; Rate = –23.71 % yr–1; Perc Final = 15 % > decrease

DECADAL: YES (8 yr)

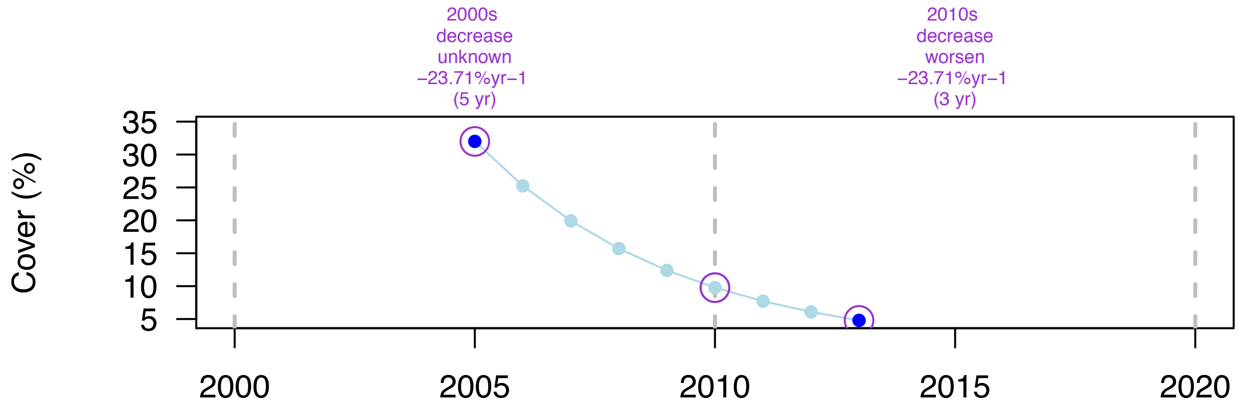

362_density

Pergent et al. 2015

SITE: Porto Polo (Corsica) (France – Mediterranean) – Po (–36.5 m)

OVERALL: Net = 94.9 shoot m–2; Rate = 3.94 % yr–1; Perc Final = 137 % > increase

DECADAL: YES (8 yr)

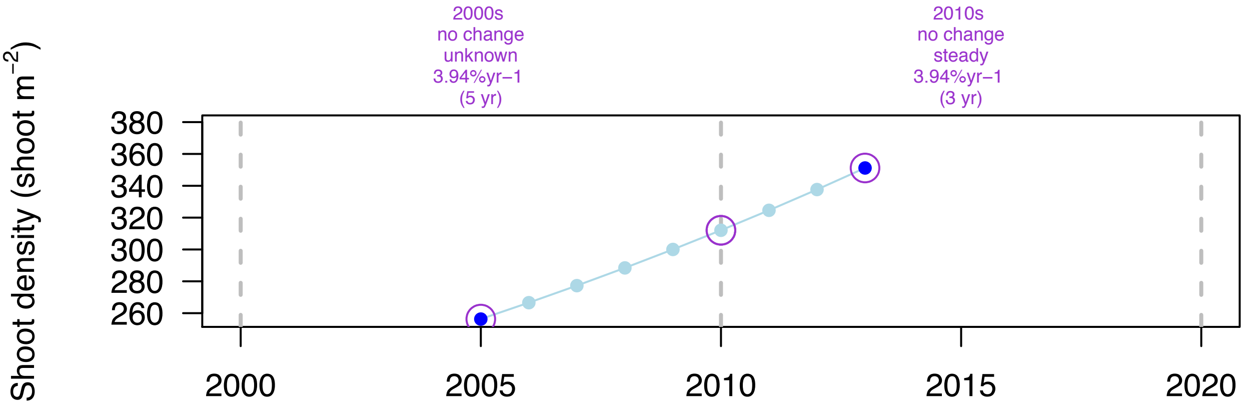

362_lowerlimit

Pergent et al. 2015

SITE: Porto Polo (Corsica) (France – Mediterranean) – Po (–36.5 m)

OVERALL: Net = –4.3 m; Rate = –1.57 % yr–1; Perc Final = 88 % > decrease

DECADAL: YES (8 yr)

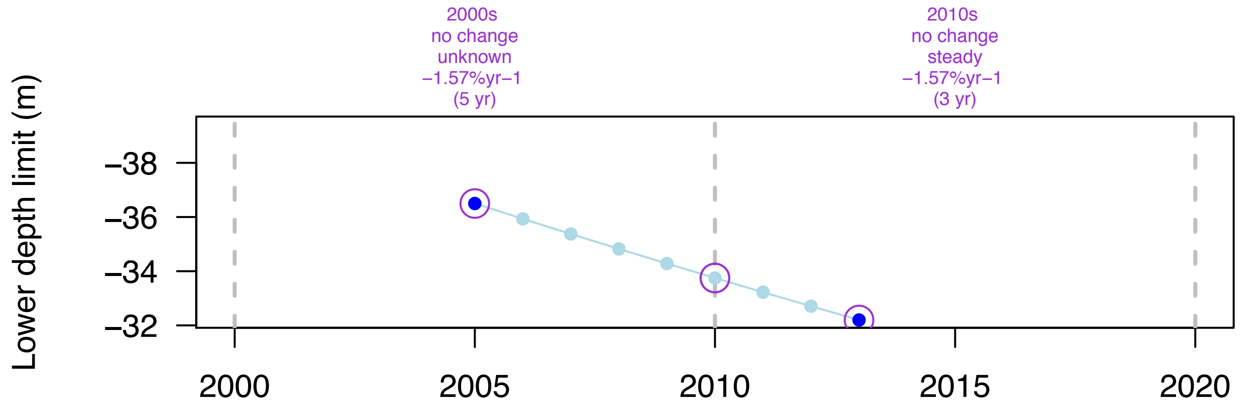

363_cover

Pergent et al. 2015

SITE: La Parata (Corsica) (France – Mediterranean) – Po (–35.3 m)

OVERALL: Net = –9.1 %; Rate = –28.91 % yr–1; Perc Final = 10 % > decrease

DECADAL: YES (8 yr)

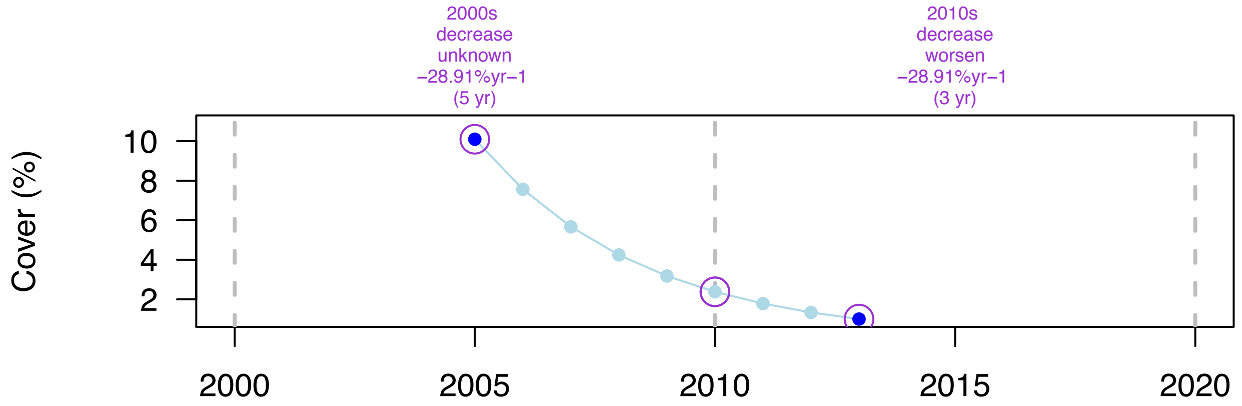

363_density

Pergent et al. 2015

SITE: La Parata (Corsica) (France – Mediterranean) – Po (–35.3 m)

OVERALL: Net = 35.5 shoot m–2;   Rate = 2.3 % yr–1; Perc Final = 120 % > no change

DECADAL: YES (8 yr)

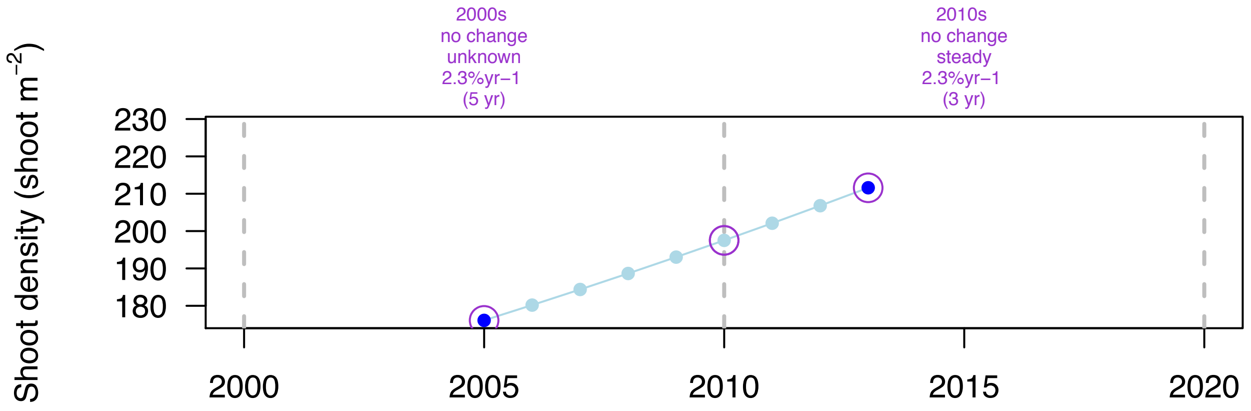

363_lowerlimit

Pergent et al. 2015

SITE: La Parata (Corsica) (France – Mediterranean) – Po (–35.3 m)

OVERALL: Net = 0 m;   Rate = 0 % yr–1; Perc Final = 100 % > no change

DECADAL: YES (8 yr)

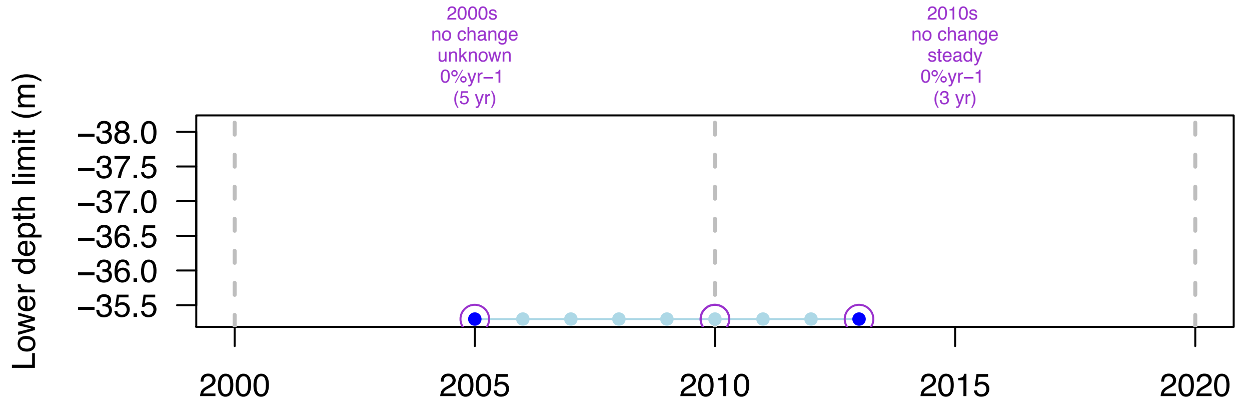

364_cover

Pergent et al. 2015

SITE: Sagone (Corsica) (France – Mediterranean) – Po (–33.2 m)

OVERALL: Net = 8.7 %; Rate = 4.73 % yr–1; Perc Final = 133 % > increase

DECADAL: NO (6 yr)

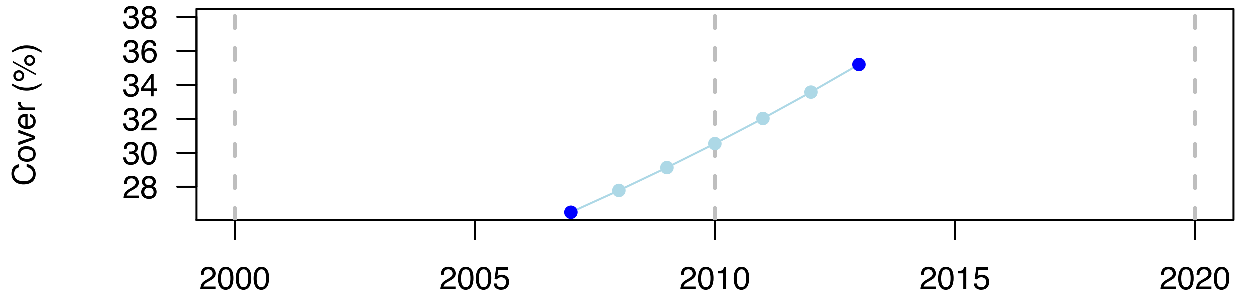

364_density

Pergent et al. 2015

SITE: Sagone (Corsica) (France – Mediterranean) – Po (–33.2 m)

OVERALL: Net = –24.4 shoot m–2; Rate = –0.88 % yr–1; Perc Final = 95 % > no change

DECADAL: NO (6 yr)

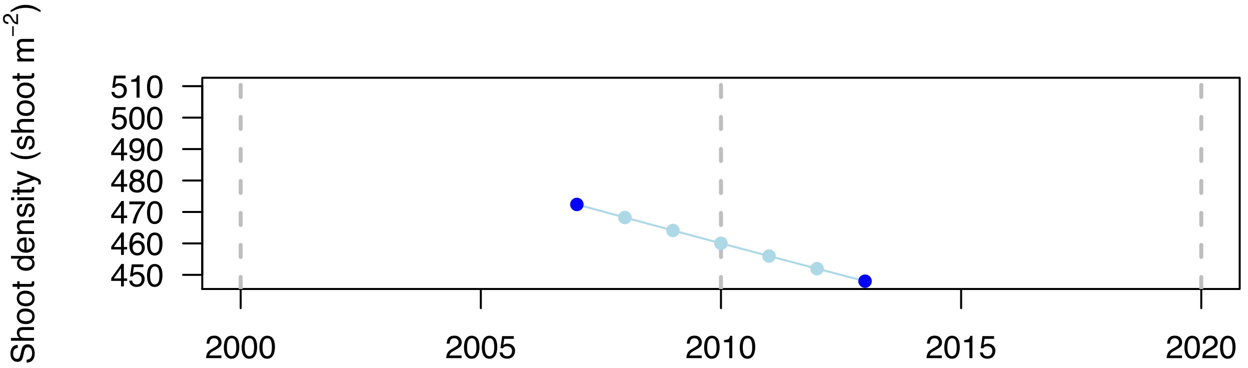

364_lowerlimit

Pergent et al. 2015

SITE: Sagone (Corsica) (France – Mediterranean) – Po (–33.2 m)

OVERALL: Net = 0 m;   Rate = 0 % yr–1; Perc Final = 100 % > no change

DECADAL: NO (6 yr)

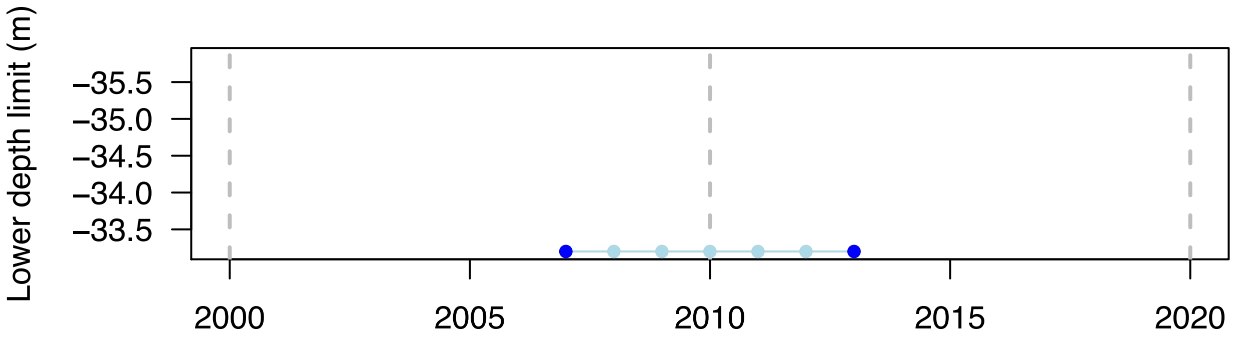

365_cover

Pergent et al. 2015

SITE: Porto (Corsica) (France – Mediterranean) – Po (–36.5 m)

OVERALL: Net = 3.4 %;   Rate = 2.6 % yr–1; Perc Final = 117 % > no change

DECADAL: NO (6 yr)

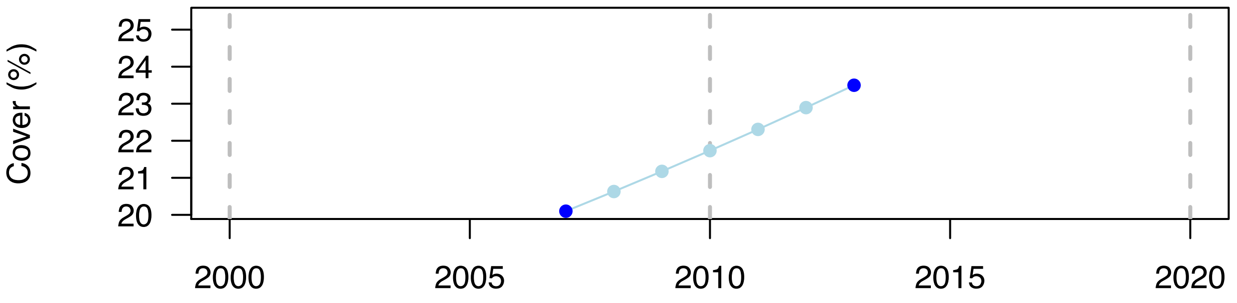

365_density

Pergent et al. 2015

SITE: Porto (Corsica) (France – Mediterranean) – Po (–36.5 m)

OVERALL: Net = –40 shoot m–2; Rate = –2.1 % yr–1; Perc Final = 88 % > no change

DECADAL: NO (6 yr)

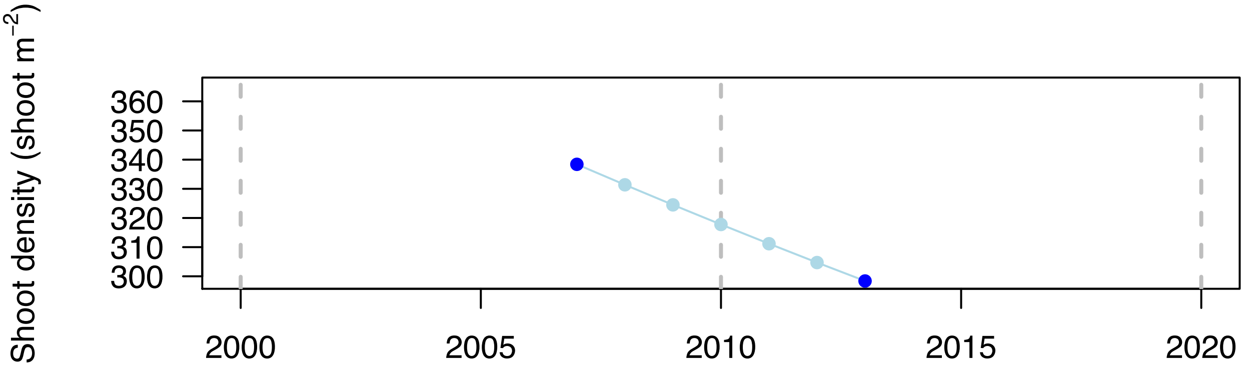

365_lowerlimit

Pergent et al. 2015

SITE: Porto (Corsica) (France – Mediterranean) – Po (–36.5 m)

OVERALL: Net = 0 m; Rate = 0 % yr–1; Perc Final = 100 % > no change

DECADAL: NO (6 yr)

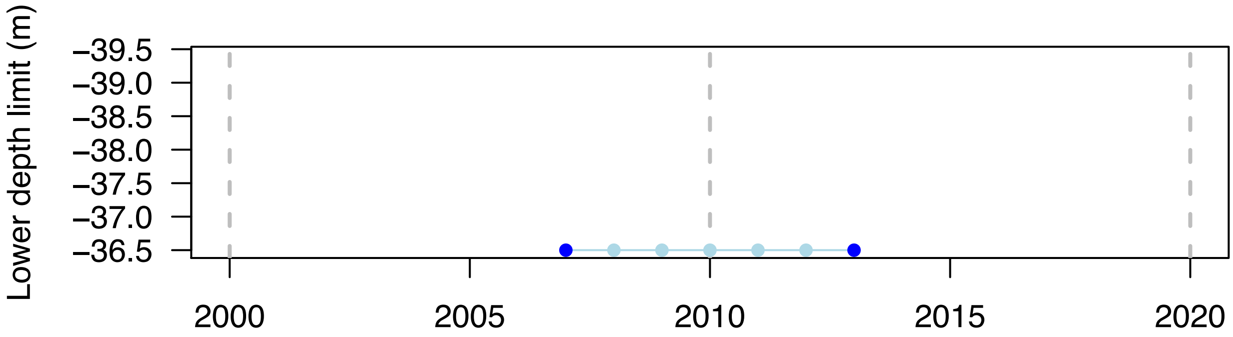

366_cover Pergent et al. 2015

SITE: Stareso (Corsica) (France – Mediterranean) – Po (–38.6 m)

OVERALL: Net = –8.4 %;   Rate = –5.18 % yr–1; Perc Final = 70 % > decrease

DECADAL: NO (7 yr)

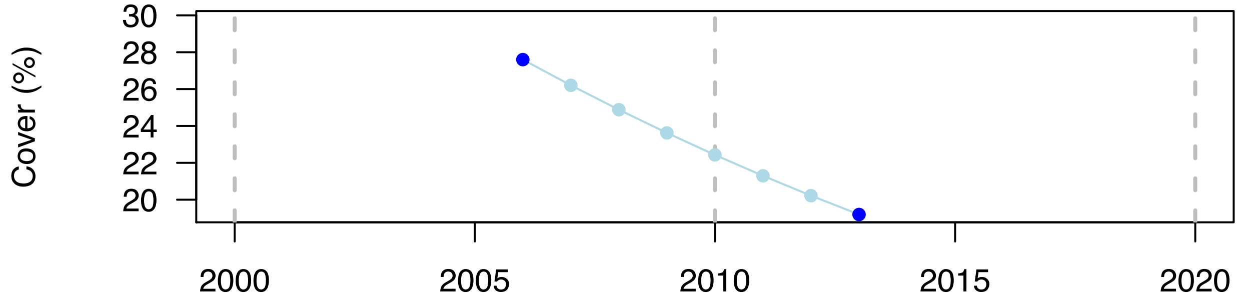

366_density Pergent et al. 2015

SITE: Stareso (Corsica) (France – Mediterranean) – Po (–38.6 m)

OVERALL: Net = –69.6 shoot m–2;   Rate = –3.63 % yr–1; Perc Final = 78 % > no change

DECADAL: NO (7 yr)

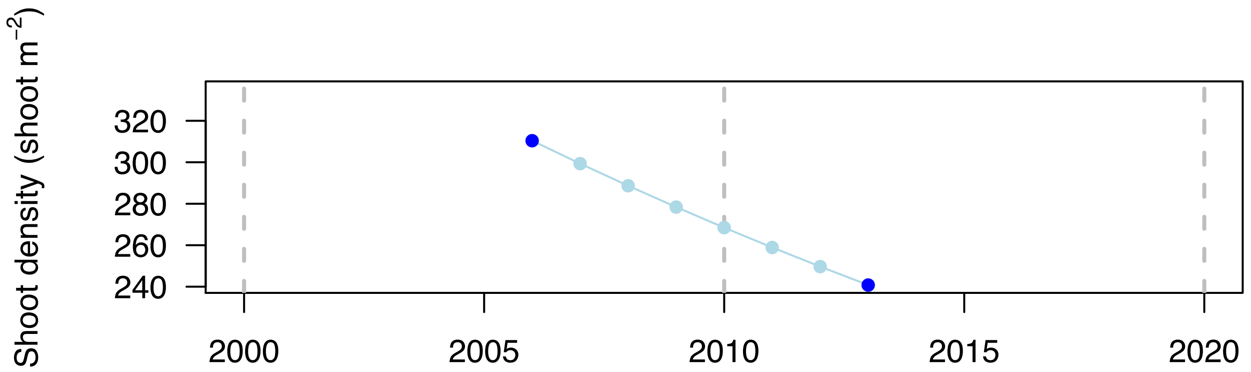

366_lowerlimit

Pergent et al. 2015

SITE: Stareso (Corsica) (France – Mediterranean) – Po (–38.6 m)

OVERALL: Net = 0 m;   Rate = 0 % yr–1; Perc Final = 100 % > no change

DECADAL: NO (7 yr)

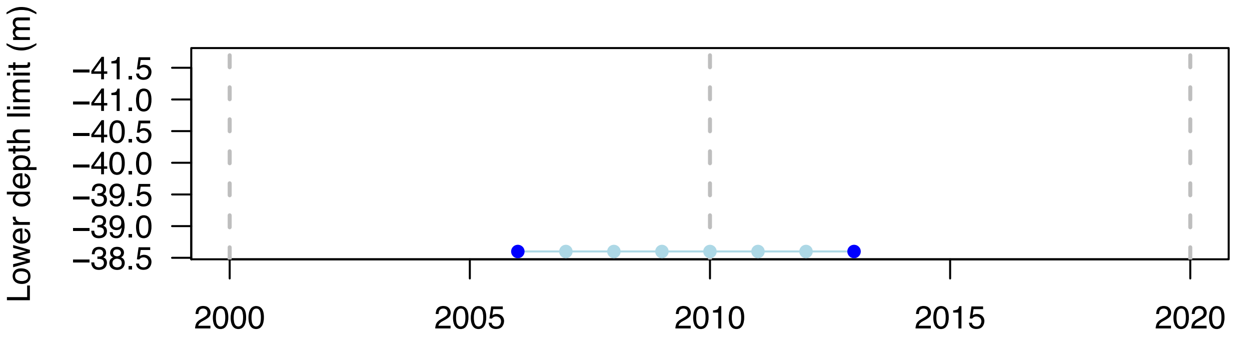

367_cover

Pergent et al. 2015

SITE: L'Île Rousse (Corsica) (France – Mediterranean) – Po (–35.8 m)

OVERALL: Net = –11 %;   Rate = –6.61 % yr–1; Perc Final = 67 % > decrease

DECADAL: NO (6 yr)

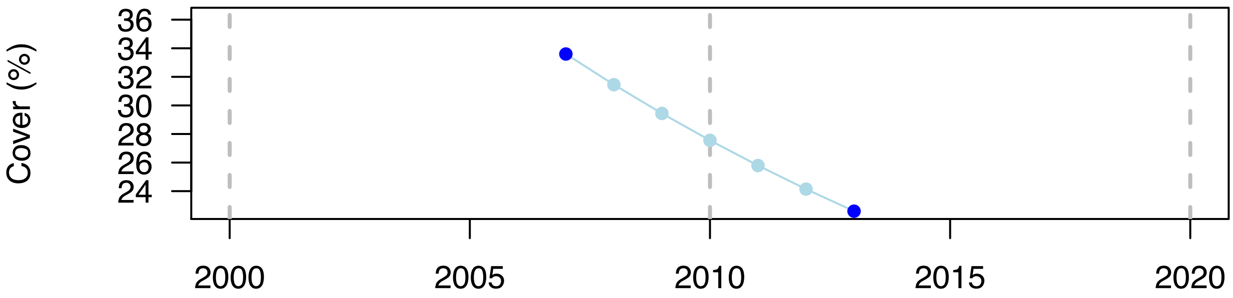

367_density

Pergent et al. 2015

SITE: L’Île Rousse (Corsica) (France – Mediterranean) – Po (–35.8 m)

OVERALL: Net = 50.1 shoot m–2;   Rate = 3.59 % yr–1; Perc Final = 124 % > no change

DECADAL: NO (6 yr)

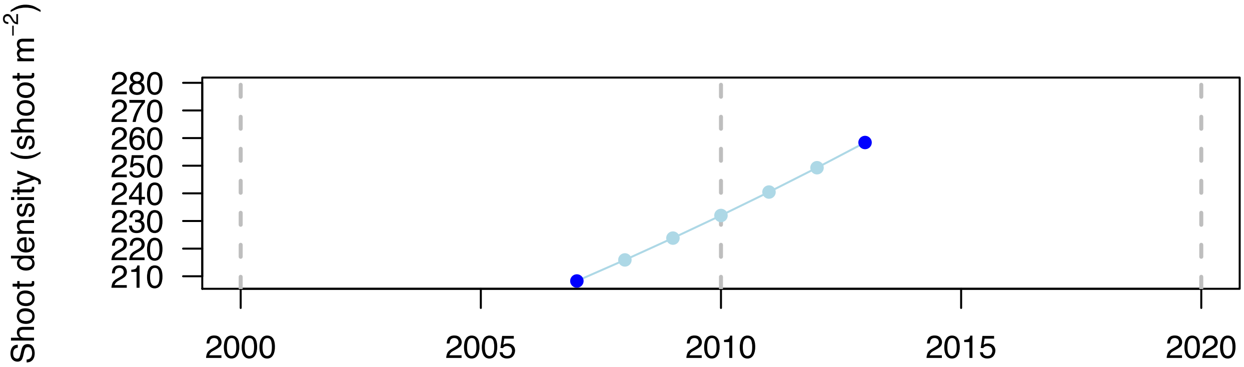

367_lowerlimit

Pergent et al. 2015

SITE: L’Île Rousse (Corsica) (France – Mediterranean) – Po (–35.8 m)

OVERALL: Net = 0 m;   Rate = 0 % yr–1; Perc Final = 100 % > no change

DECADAL: NO (6 yr)

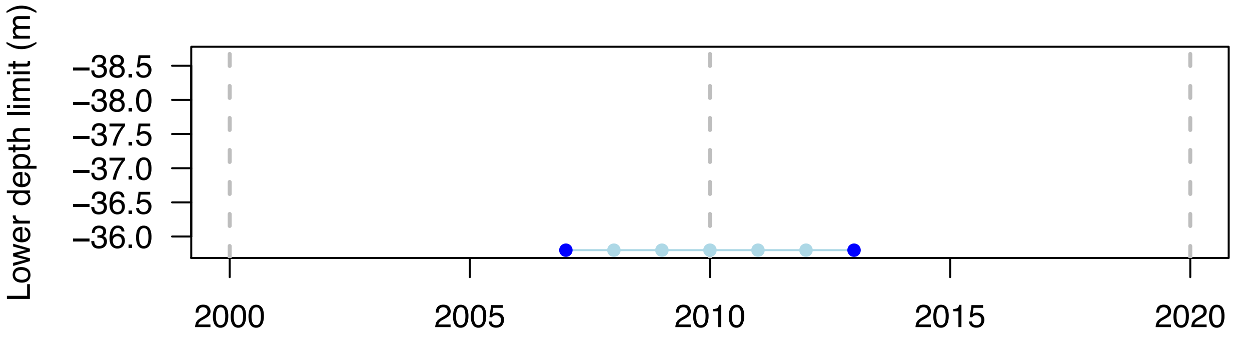

368_cover

Pergent et al. 2015

SITE: Canari (Corsica) (France – Mediterranean) – Po (–27.4 m)

OVERALL: Net = 10.5 %;   Rate = 1.9 % yr–1; Perc Final = 112 % > no change

DECADAL: NO (6 yr)

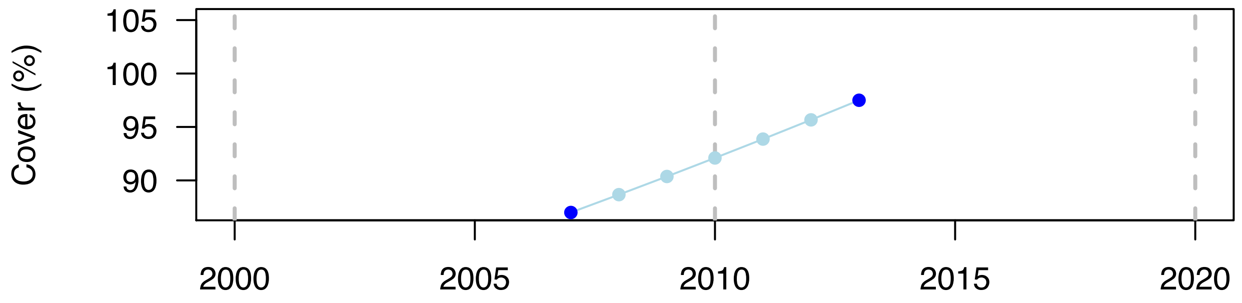

368_density

Pergent et al. 2015

SITE: Canari (Corsica) (France – Mediterranean) – Po (–27.4 m)

OVERALL: Net = 96.6 shoot m–2;   Rate = 4.94 % yr–1; Perc Final = 134 % > increase

DECADAL: NO (6 yr)

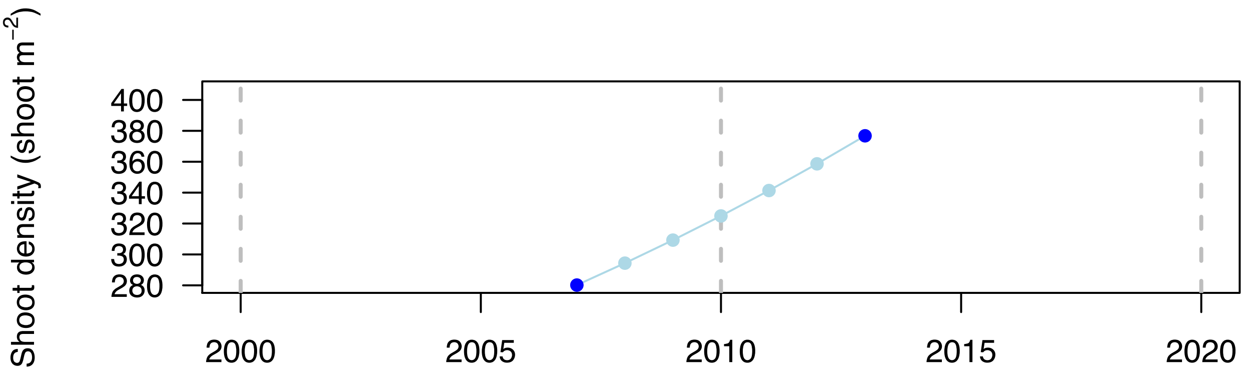

368_lowerlimit

Pergent et al. 2015

SITE: Canari (Corsica) (France – Mediterranean) – Po (–27.4 m)

OVERALL: Net = 0 m;   Rate = 0 % yr–1; Perc Final = 100 % > no change

DECADAL: NO (6 yr)

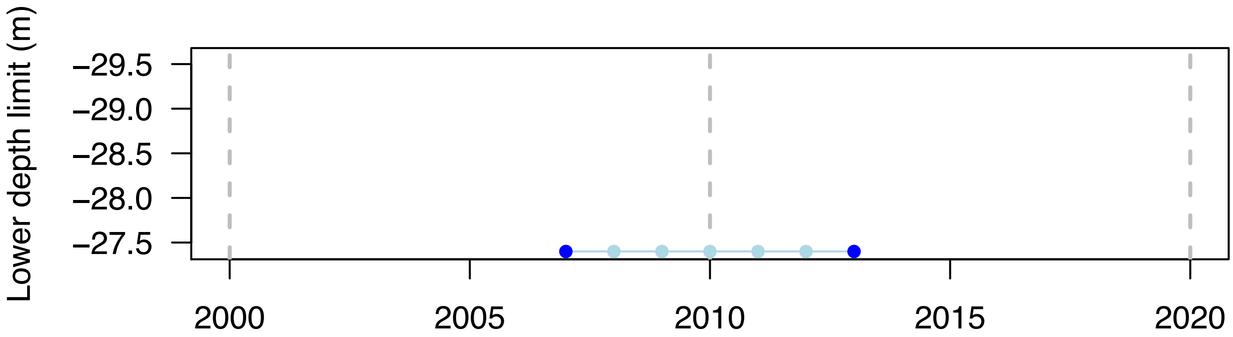

370_area

Cunha et al. 2005

SITE: Ancão Inlet (Portugal – Atlantic) – Zn (0 m)

OVERALL: Net = 13.6 ha;   Rate = 1.84 % yr–1; Perc Final = 292 % > increase

DECADAL: YES (58 yr)

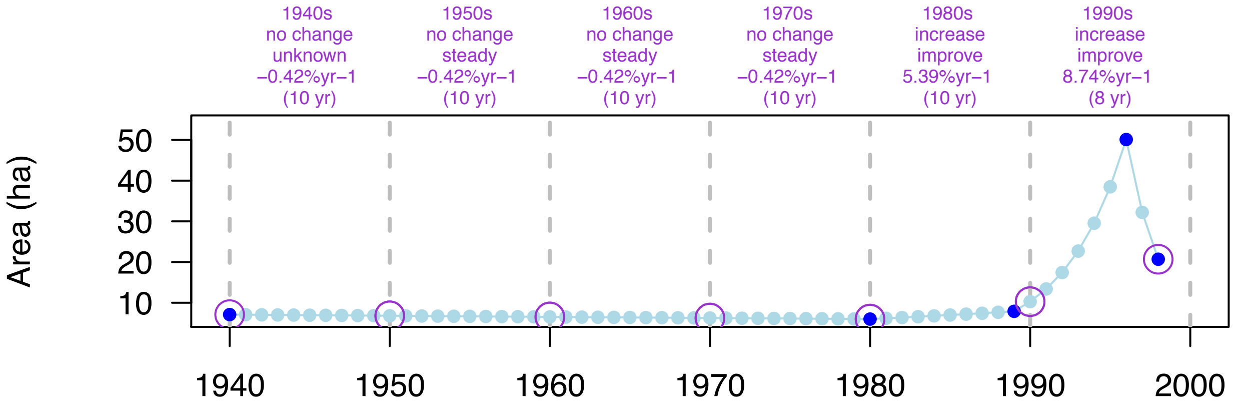

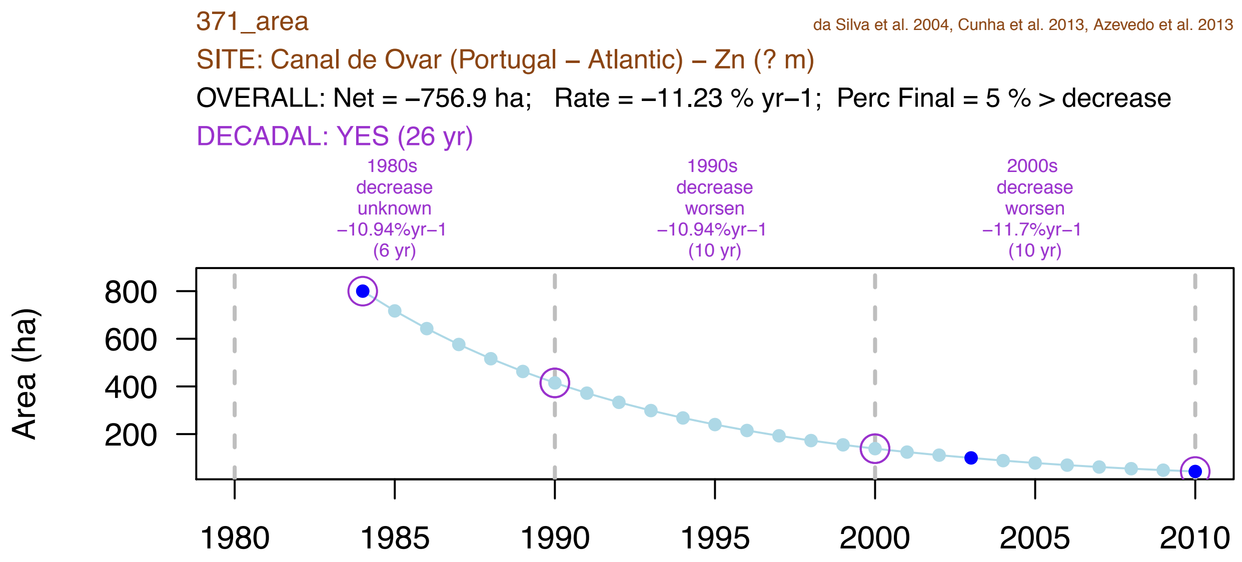

371_area
da Silva et al. 2004, Cunha et al. 2013, Azevedo et al. 2013
SITE: Canal de Ovar (Portugal – Atlantic) – Zn (? m)
OVERALL: Net = –756.9 ha; Rate = –11.23 % yr–1; Perc Final = 5 % > decrease
DECADAL: YES (26 yr)
1980s decrease unknown –10.94%yr–1 (6 yr)
1990s decrease worsen –10.94%yr–1 (10 yr)
2000s decrease worsen –11.7%yr–1 (10 yr)
Area (ha)
800
600
400
200
1980
1985
1990
1995
2000
2005
2010

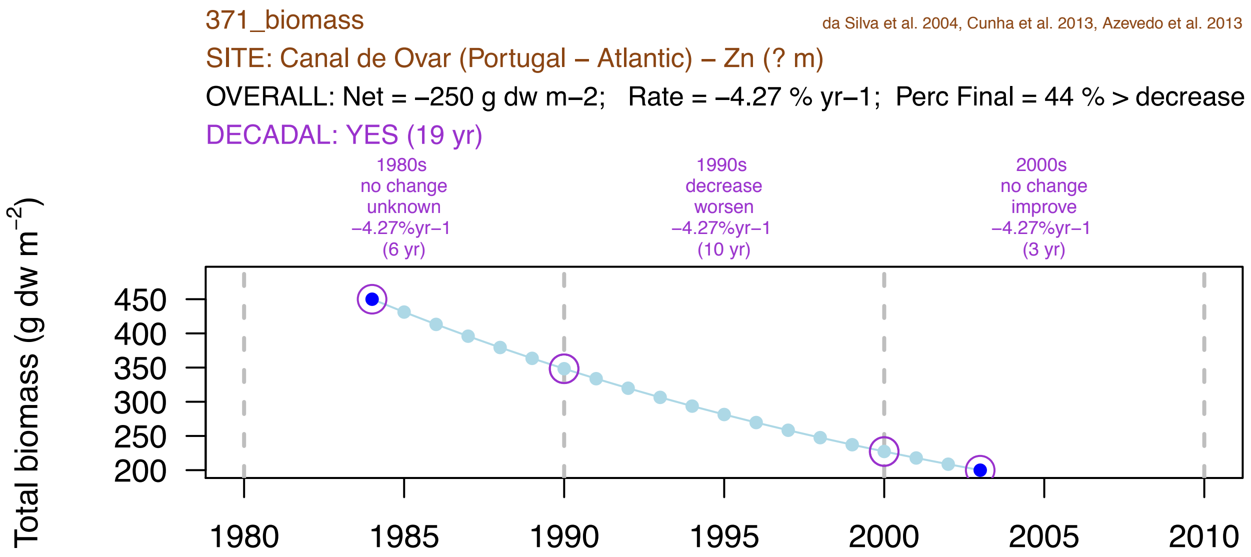

371_biomass
da Silva et al. 2004, Cunha et al. 2013, Azevedo et al. 2013
SITE: Canal de Ovar (Portugal – Atlantic) – Zn (? m)
OVERALL: Net = –250 g dw m–2; Rate = –4.27 % yr–1; Perc Final = 44 % > decrease
DECADAL: YES (19 yr)
1980s no change unknown –4.27%yr–1 (6 yr)
1990s decrease worsen –4.27%yr–1 (10 yr)
2000s no change improve –4.27%yr–1 (3 yr)
Total biomass (g dw m^{-2})
450
400
350
300
250
200
1980
1985
1990
1995
2000
2005
2010

372_area

Cunha et al. 2013

SITE: Mira River (Portugal – Atlantic) – Zn (? m)

OVERALL: Net = −72.5 ha;   Rate = −9.47 % yr−1; Perc Final = 9 % > decrease

DECADAL: YES (25 yr)

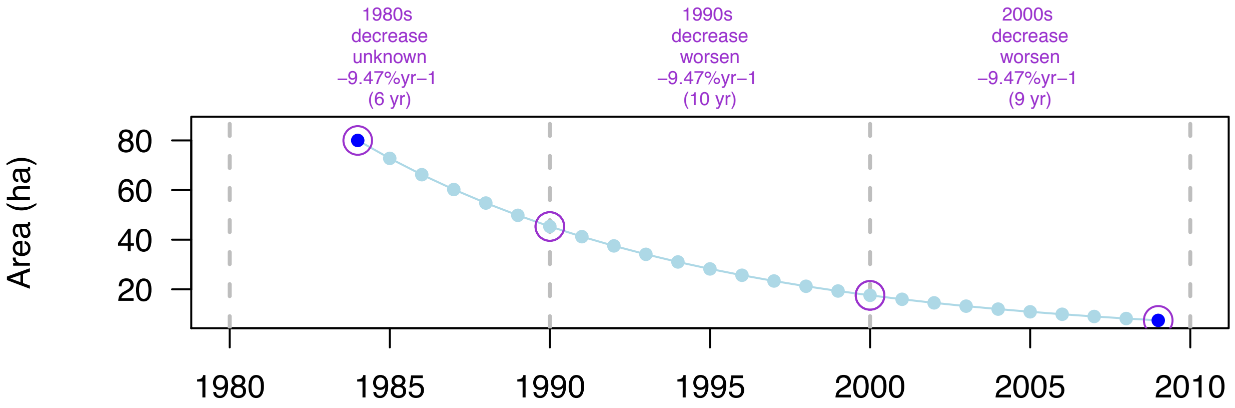

373_area

Cunha et al. 2013

SITE: Ria de Alvor (Portugal – Atlantic) – Zn (? m)

OVERALL: Net = −1.96 ha;   Rate = NA % yr−1; Perc Final = NA % > decrease

DECADAL: YES (37 yr)

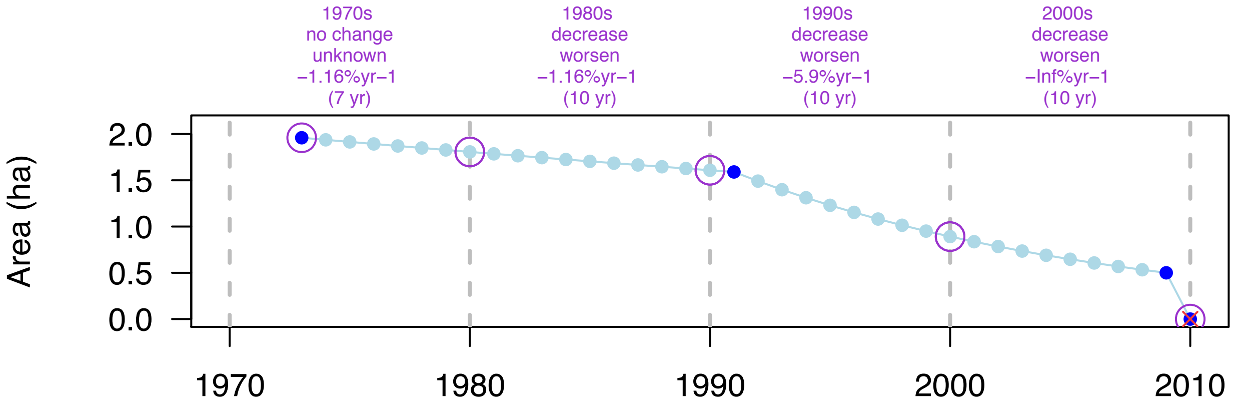

374_area

Cunha et al. 2013

SITE: Arade River (Portugal – Atlantic) – Zn (? m)

OVERALL: Net = −1.4 ha; Rate = −30.08 % yr−1; Perc Final = 22 % > decrease

DECADAL: NO (5 yr)

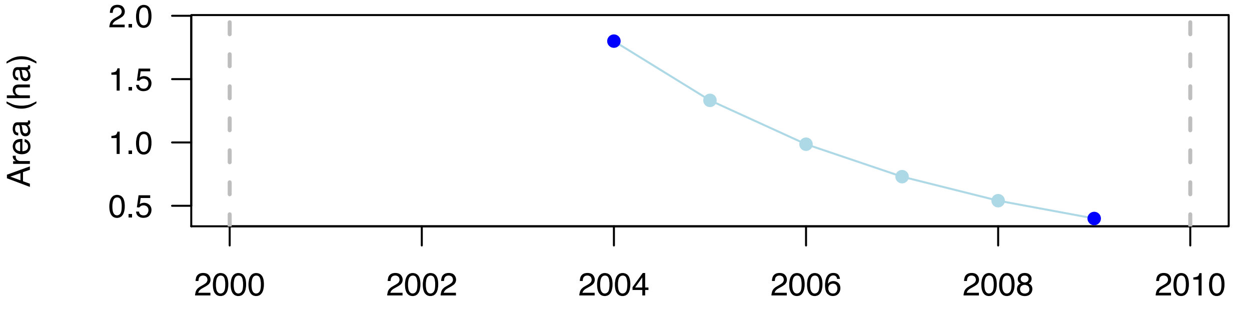

375_area

Cunha et al. 2013

SITE: Óbidos Lagoon (Portugal – Atlantic) – Zm (? m)

OVERALL: Net = −148.95 ha; Rate = −9.92 % yr−1; Perc Final = 1 % > decrease

DECADAL: YES (50 yr)

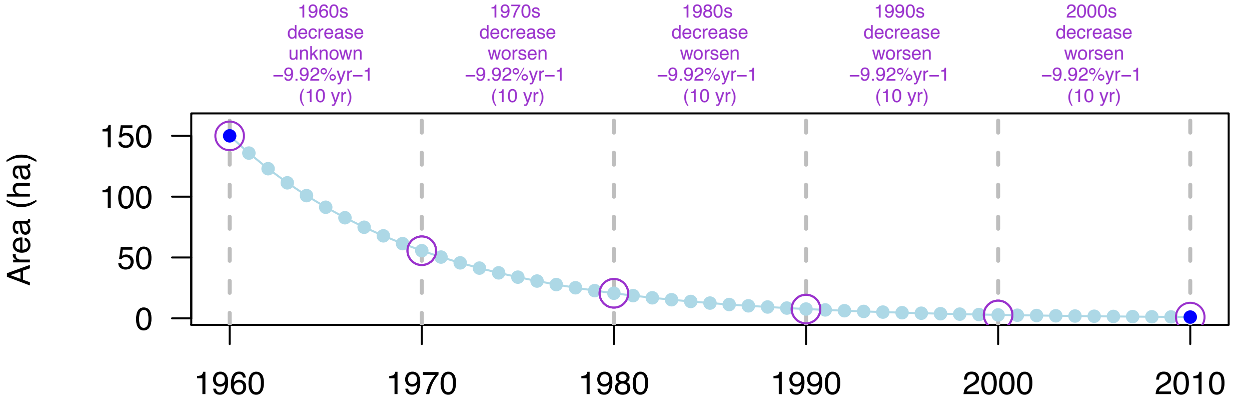

376_area
Cunha et al. 2013
SITE: Costa da Galé (Portugal – Atlantic) – Zm (? m)
OVERALL: Net = 0 ha;   Rate = NA % yr–1; Perc Final = NA % > decrease
DECADAL: NO (4 yr)

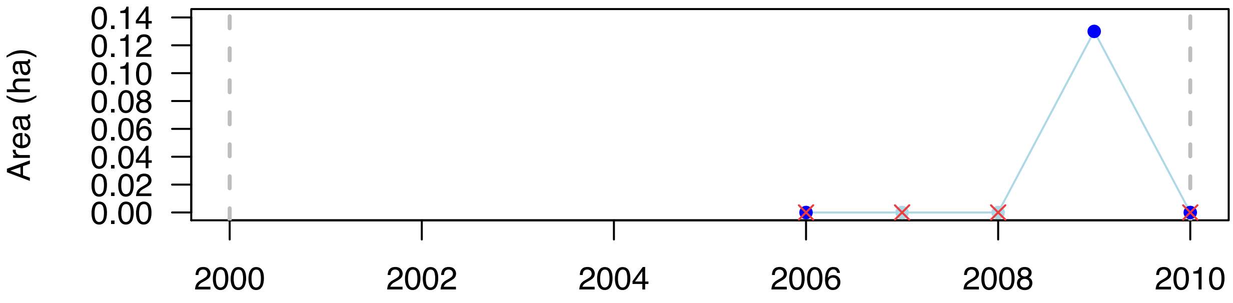

377_area
Cunha et al. 2013
SITE: Mira River (Portugal – Atlantic) – Zm (? m)
OVERALL: Net = –0.4 ha;   Rate = NA % yr–1; Perc Final = NA % > decrease
DECADAL: YES (25 yr)

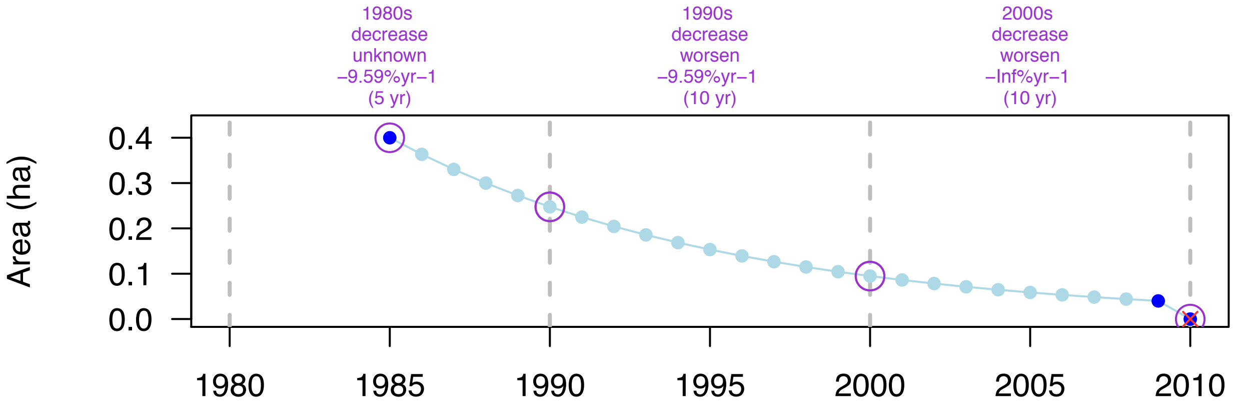

378_area

Cunha et al. 2013

SITE: Portinho da Arrábida (Portugal – Atlantic) – Zm (? m)

OVERALL: Net = −10 ha;   Rate = NA % yr−1; Perc Final = NA % > decrease

DECADAL: YES (61 yr)

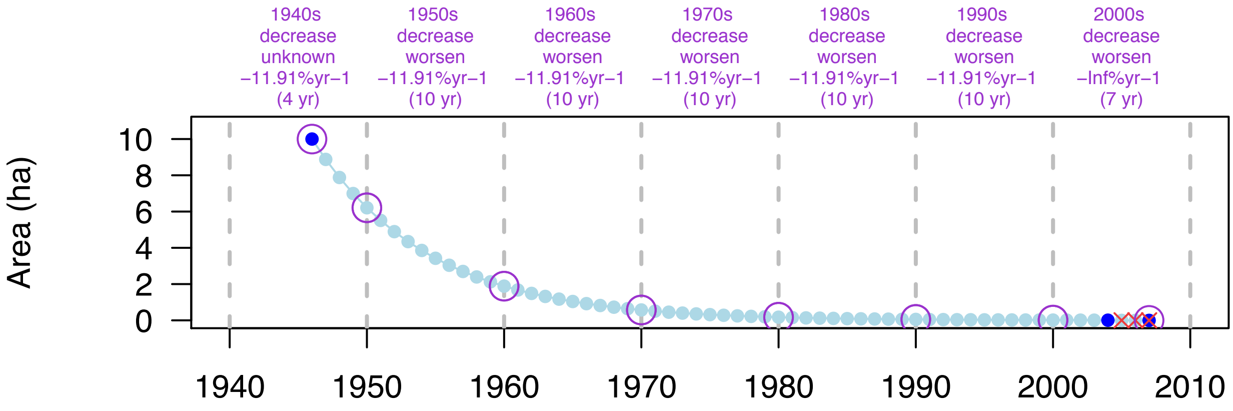

379_area

Cunha et al. 2013

SITE: Ponta do Adoche (Portugal – Atlantic) – Zm (? m)

OVERALL: Net = −1.21 ha;   Rate = NA % yr−1; Perc Final = NA % > decrease

DECADAL: NO (1 yr)

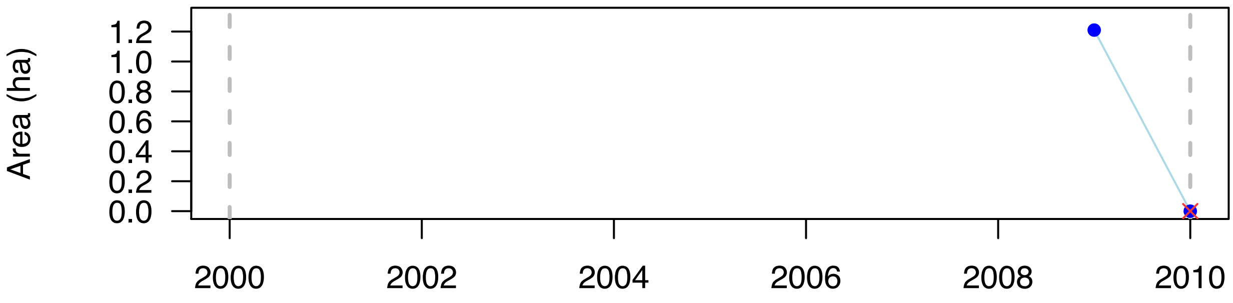

381_biomass

Cabaço et al. 2010, Cabaço and Santos 2014, Cabaço and Santos (unpublished)

SITE: Ancão Peninsula (meadow A) (Portugal – Atlantic) – Cn (? m)

OVERALL: Net = −399.52 g dw m−2; Rate = NA % yr−1; Perc Final = NA % > decrease

DECADAL: YES (10 yr)

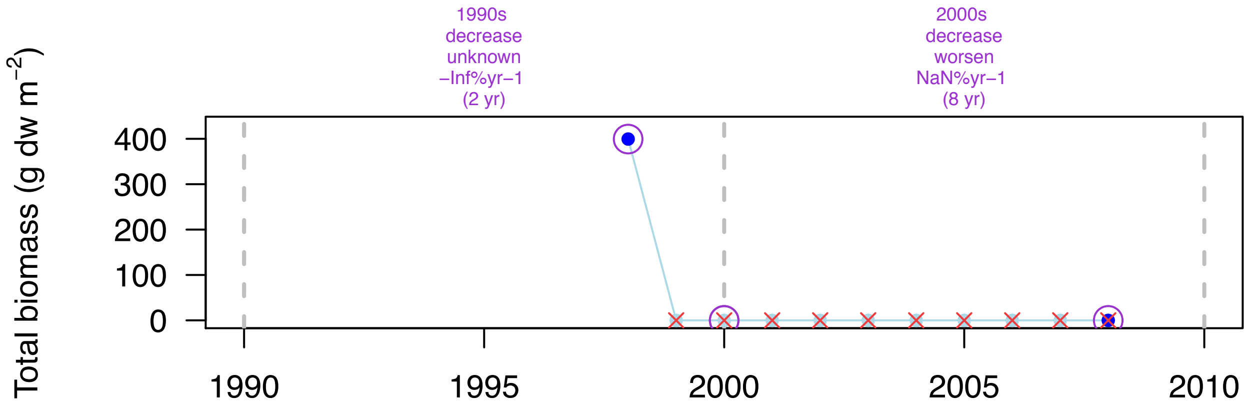

381_density

Cabaço et al. 2010, Cabaço and Santos 2014, Cabaço and Santos (unpublished)

SITE: Ancão Peninsula (meadow A) (Portugal – Atlantic) – Cn (? m)

OVERALL: Net = −716.56 shoot m−2; Rate = NA % yr−1; Perc Final = NA % > decrease

DECADAL: YES (10 yr)

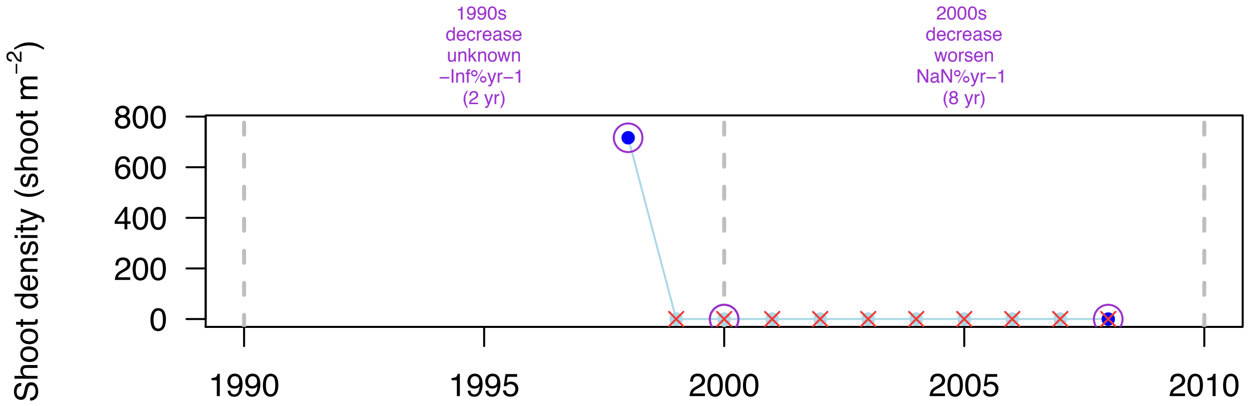

382_biomass

Cabaço et al. 2010, Cabaço and Santos 2014, Cabaço and Santos (unpublished)

SITE: Ancão Peninsula (meadow B) (Portugal – Atlantic) – Cn (? m)

OVERALL: Net = –186.35 g dw m–2; Rate = NA % yr–1; Perc Final = NA % > decrease

DECADAL: YES (10 yr)

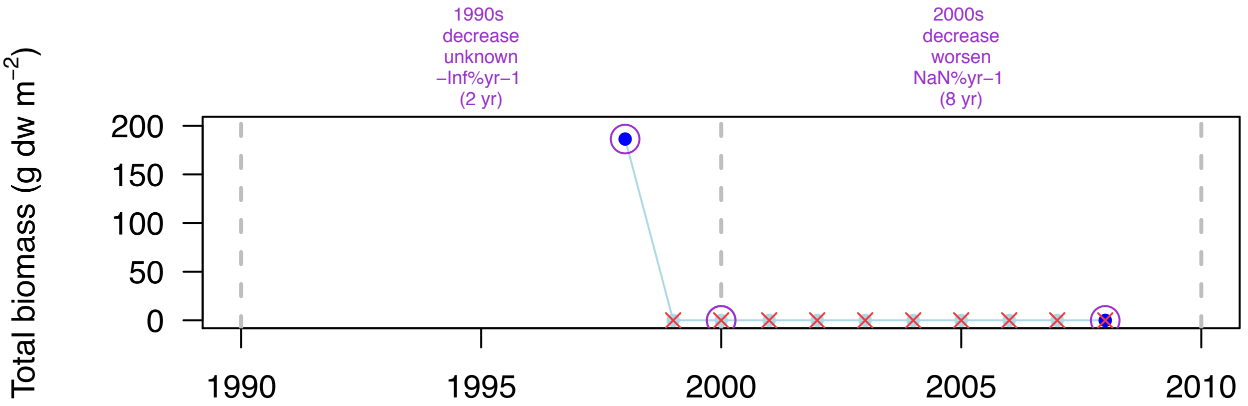

382_density

Cabaço et al. 2010, Cabaço and Santos 2014, Cabaço and Santos (unpublished)

SITE: Ancão Peninsula (meadow B) (Portugal – Atlantic) – Cn (? m)

OVERALL: Net = –573.25 shoot m–2; Rate = NA % yr–1; Perc Final = NA % > decrease

DECADAL: YES (10 yr)

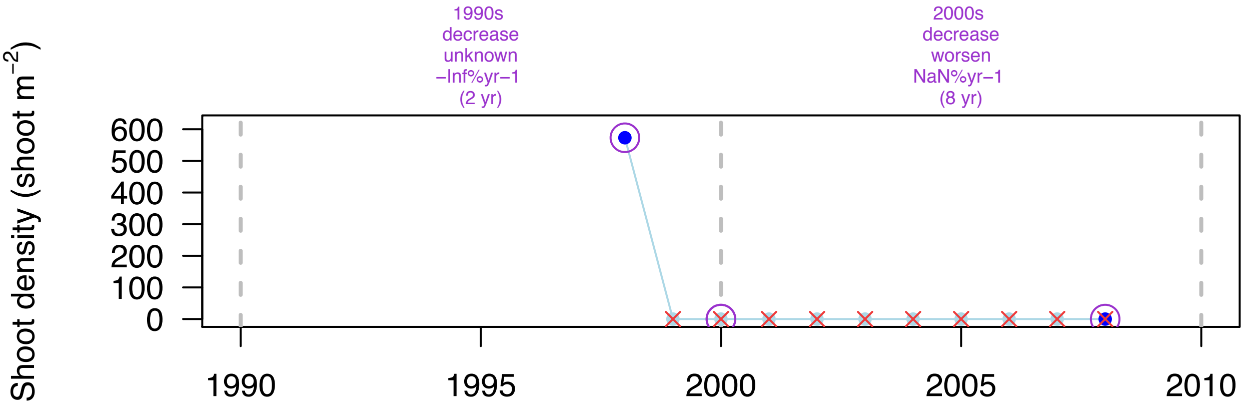

383_biomass

Cabaço et al. 2010, Cabaço and Santos 2014, Cabaço and Santos (unpublished)

SITE: Ancão Peninsula (meadow C) (Portugal – Atlantic) – Cn (? m)

OVERALL: Net = 494.65 g dw m−2;   Rate = 10.24 % yr−1; Perc Final = 278 % > increase

DECADAL: YES (10 yr)

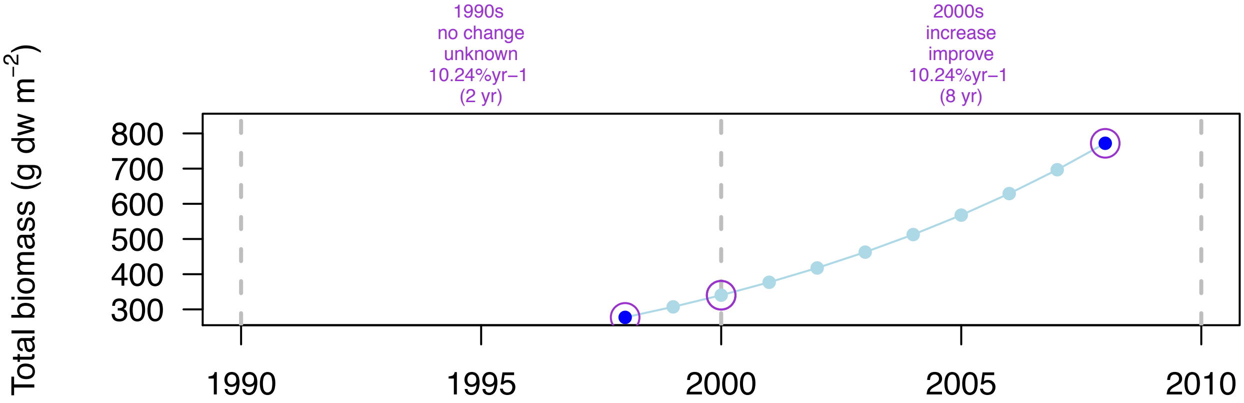

383_density

Cabaço et al. 2010, Cabaço and Santos 2014, Cabaço and Santos (unpublished)

SITE: Ancão Peninsula (meadow C) (Portugal – Atlantic) – Cn (? m)

OVERALL: Net = 2712.31 shoot m−2;   Rate = 16.73 % yr−1; Perc Final = 533 % > increase

DECADAL: YES (10 yr)

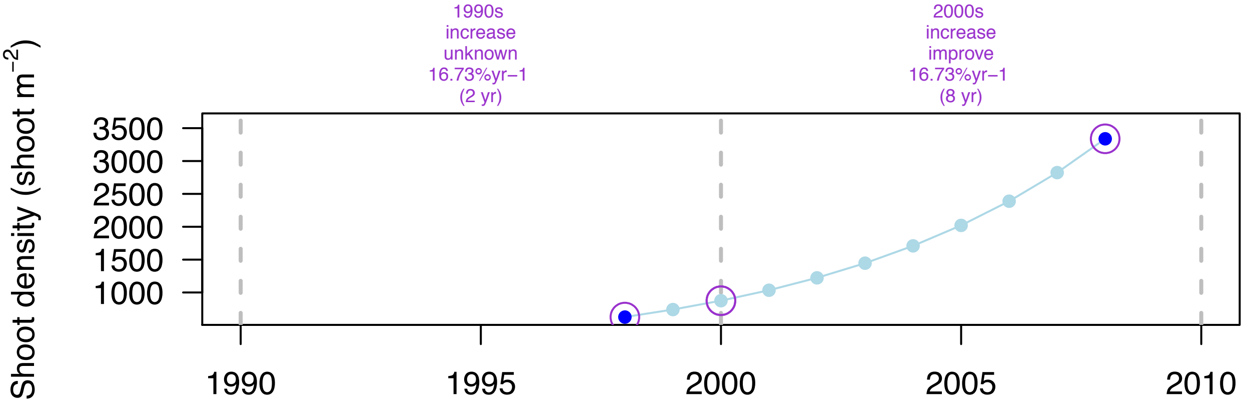

384_biomass

Cabaço et al. 2010, Cabaço and Santos 2014, Cabaço and Santos (unpublished)

SITE: Ramalhete (meadow D) (Portugal – Atlantic) – Cn (? m)

OVERALL: Net = −109.11 g dw m−2;   Rate = −2.06 % yr−1; Perc Final = 75 % > decrease

DECADAL: YES (14 yr)

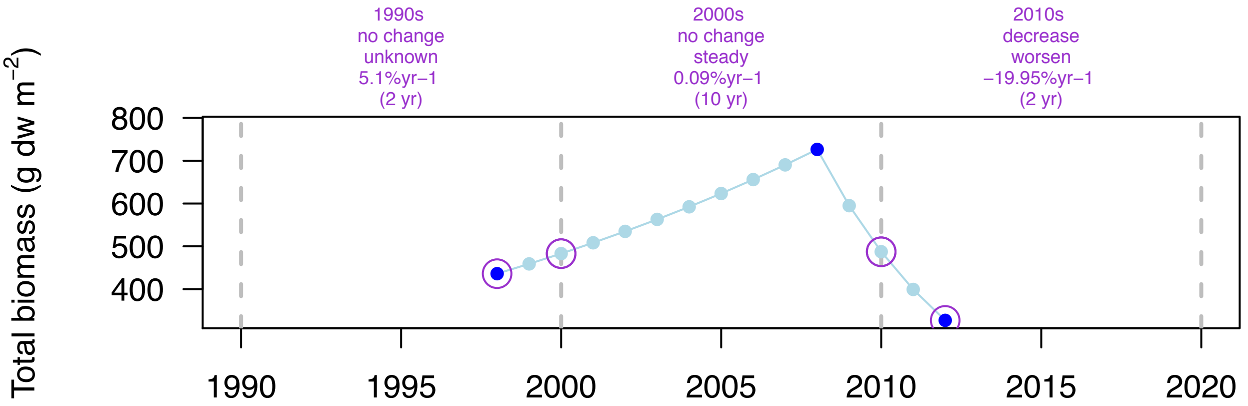

384_density

Cabaço et al. 2010, Cabaço and Santos 2014, Cabaço and Santos (unpublished)

SITE: Ramalhete (meadow D) (Portugal – Atlantic) – Cn (? m)

OVERALL: Net = 88.9 shoot m−2;   Rate = 0.73 % yr−1; Perc Final = 111 % > no change

DECADAL: YES (14 yr)

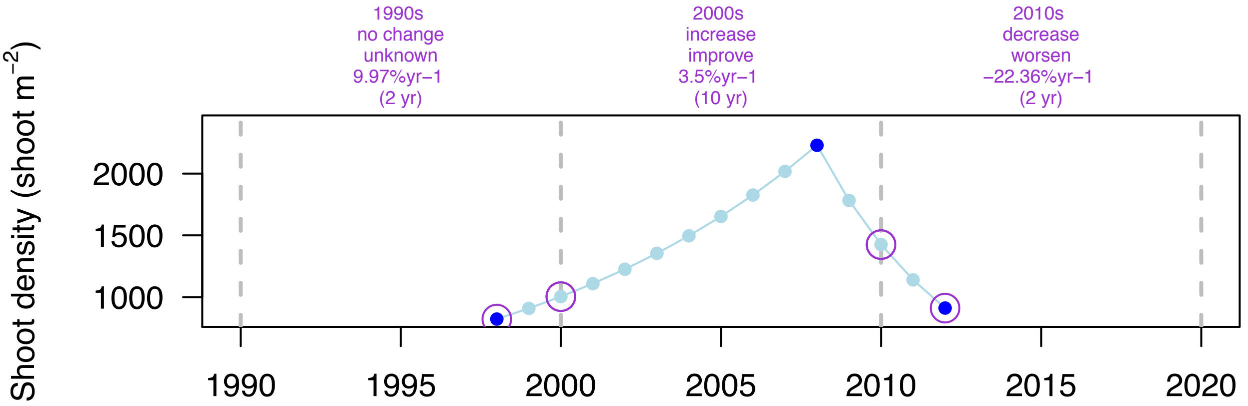

385_biomass

Cabaço and Santos (unpublished)

SITE: Ramalhete (pond outfall) (Portugal – Atlantic) – Cn (? m)

OVERALL: Net = 404.56 g dw m−2;   Rate = 47.5 % yr−1; Perc Final = 259 % > increase

DECADAL: NO (2 yr)

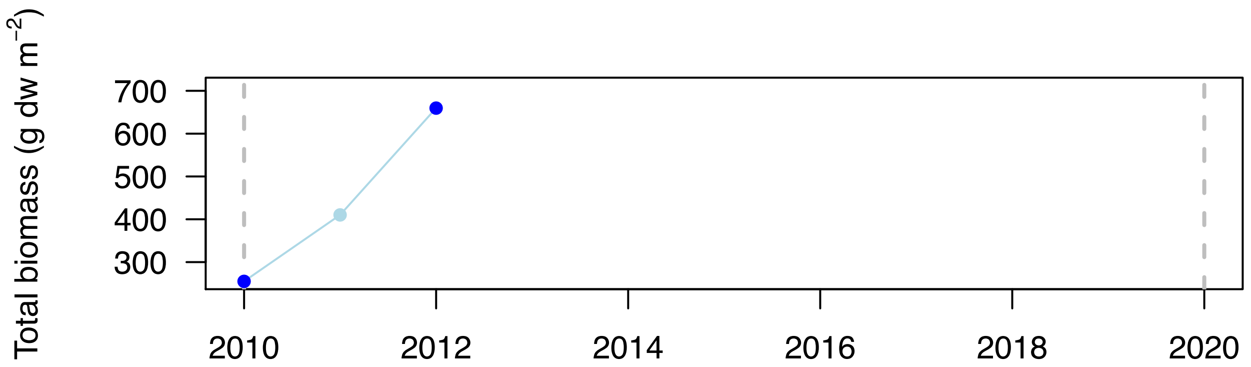

385_density

Cabaço and Santos (unpublished)

SITE: Ramalhete (pond outfall) (Portugal – Atlantic) – Cn (? m)

OVERALL: Net = −277.52 shoot m−2;   Rate = −22.56 % yr−1; Perc Final = 64 % > decrease

DECADAL: NO (2 yr)

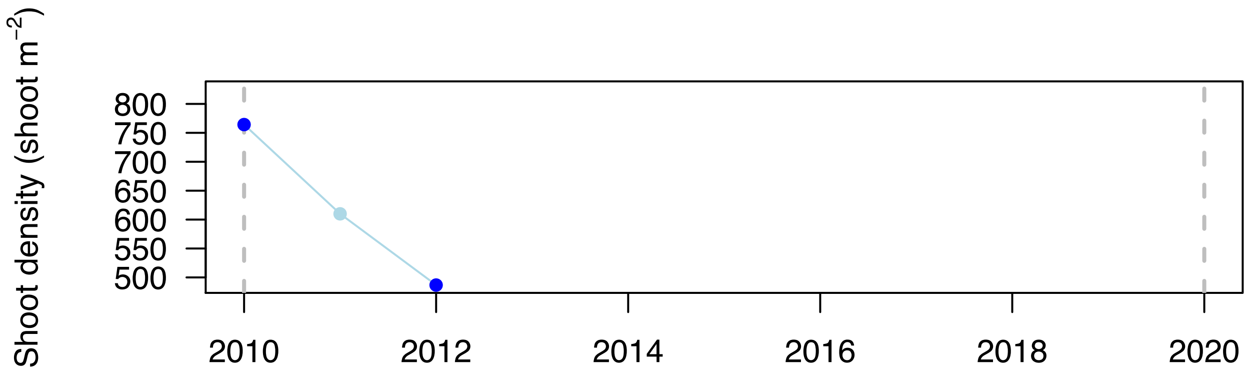

386_biomass

Cabaço et al. 2007, 2008, Cabaço and Santos (unpublished)

SITE: Ramalhete (Portugal – Atlantic) – Zn (? m)

OVERALL: Net = 79.51 g dw m–2;   Rate = 2.89 % yr–1; Perc Final = 134 % > increase

DECADAL: YES (10 yr)

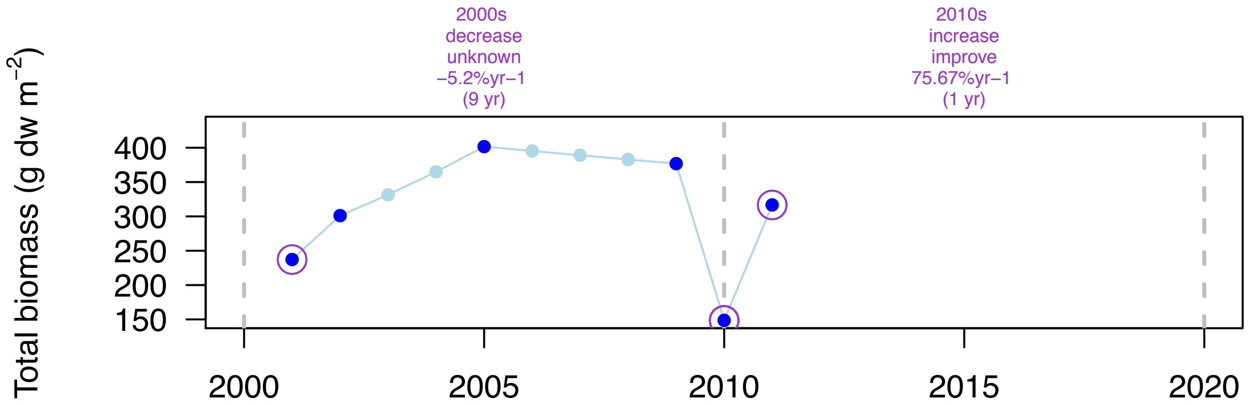

386_density

Cabaço et al. 2007, 2008, Cabaço and Santos (unpublished)

SITE: Ramalhete (Portugal – Atlantic) – Zn (? m)

OVERALL: Net = –3385.12 shoot m–2;   Rate = –3.02 % yr–1; Perc Final = 74 % > decrease

DECADAL: YES (10 yr)

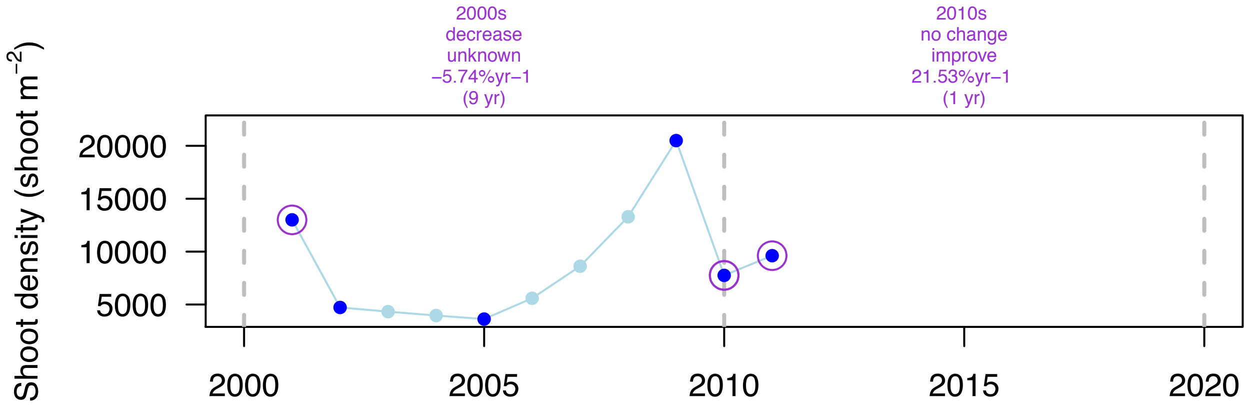

387_biomass

Cabaço et al. 2007, 2008

SITE: ETAR Faro (Portugal – Atlantic) – Zn (? m)

OVERALL: Net = −85.31 g dw m−2;   Rate = −13 % yr−1; Perc Final = 59 % > decrease

DECADAL: NO (4 yr)

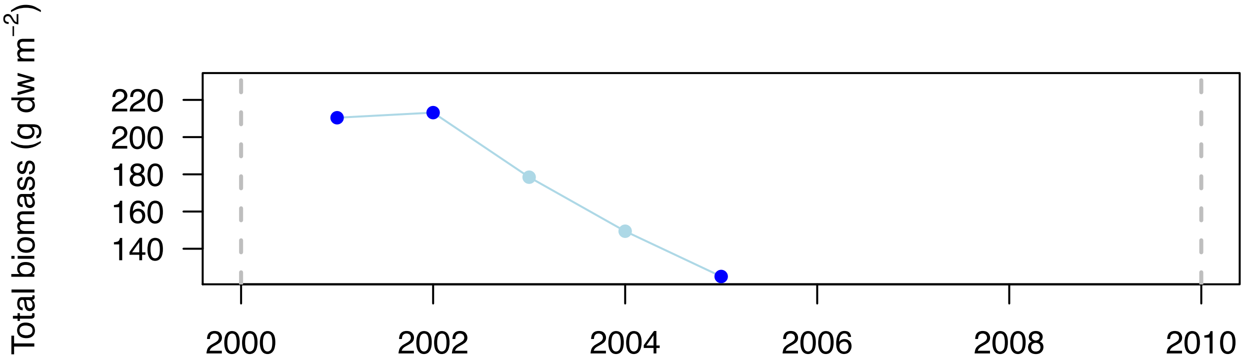

387_density

Cabaço et al. 2007, 2008

SITE: ETAR Faro (Portugal – Atlantic) – Zn (? m)

OVERALL: Net = −3666.84 shoot m−2;   Rate = −28.27 % yr−1; Perc Final = 32 % > decrease

DECADAL: NO (4 yr)

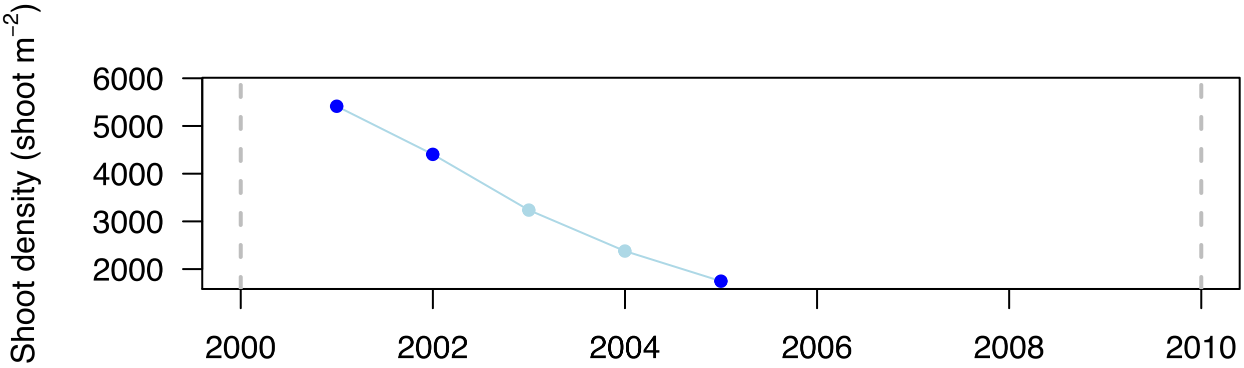

388_biomass

Cabaço et al. 2007, 2008, Cabaço and Santos (unpublished)

SITE: Praia de Faro (Portugal – Atlantic) – Zn (? m)

OVERALL: Net = −389.81 g dw m−2;   Rate = −32.58 % yr−1; Perc Final = 4 % > decrease

DECADAL: YES (10 yr)

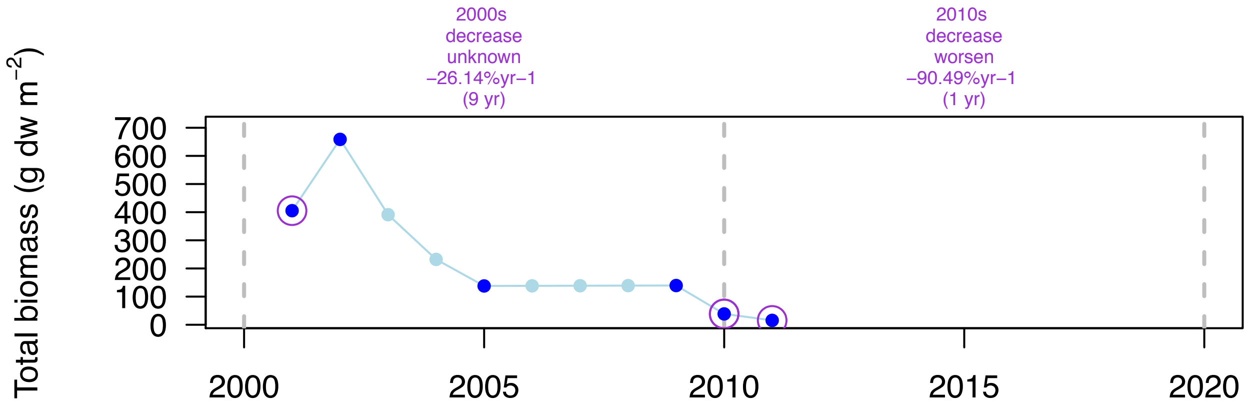

388_density

Cabaço et al. 2007, 2008, Cabaço and Santos (unpublished)

SITE: Praia de Faro (Portugal – Atlantic) – Zn (? m)

OVERALL: Net = −4888.28 shoot m−2;   Rate = −14.41 % yr−1; Perc Final = 24 % > decrease

DECADAL: YES (10 yr)

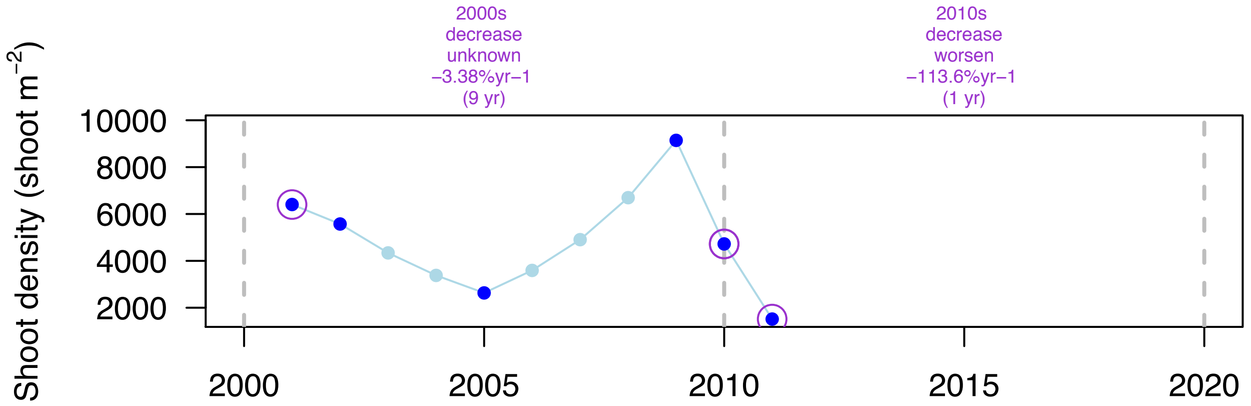

389_biomass

Cabaço et al. 2007, 2008

SITE: Quatro Águas Faro (Portugal – Atlantic) – Zn (? m)

OVERALL: Net = –272.01 g dw m–2;   Rate = –32.93 % yr–1; Perc Final = 27 % > decrease

DECADAL: NO (4 yr)

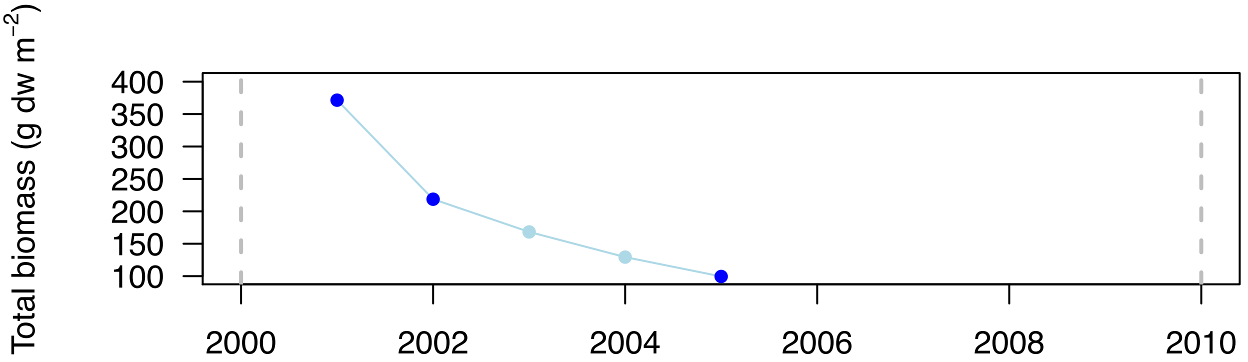

389_density

Cabaço et al. 2007, 2008

SITE: Quatro Águas Faro (Portugal – Atlantic) – Zn (? m)

OVERALL: Net = –3852.62 shoot m–2;   Rate = –24.45 % yr–1; Perc Final = 38 % > decrease

DECADAL: NO (4 yr)

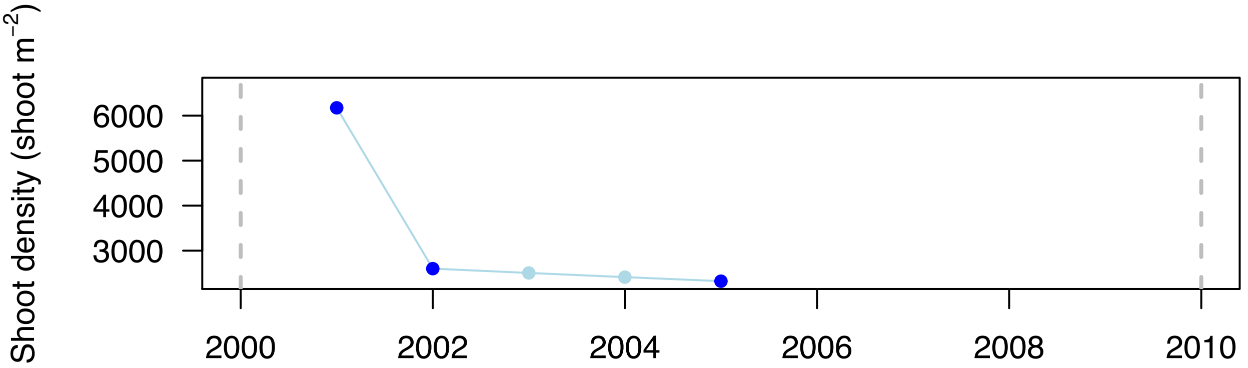

390_biomass

Cabaço and Santos (unpublished)

SITE: Barra Faro (Portugal – Atlantic) – Zn (? m)

OVERALL: Net = –656.26 g dw m–2;   Rate = NA % yr–1; Perc Final = NA % > decrease

DECADAL: NO (2 yr)

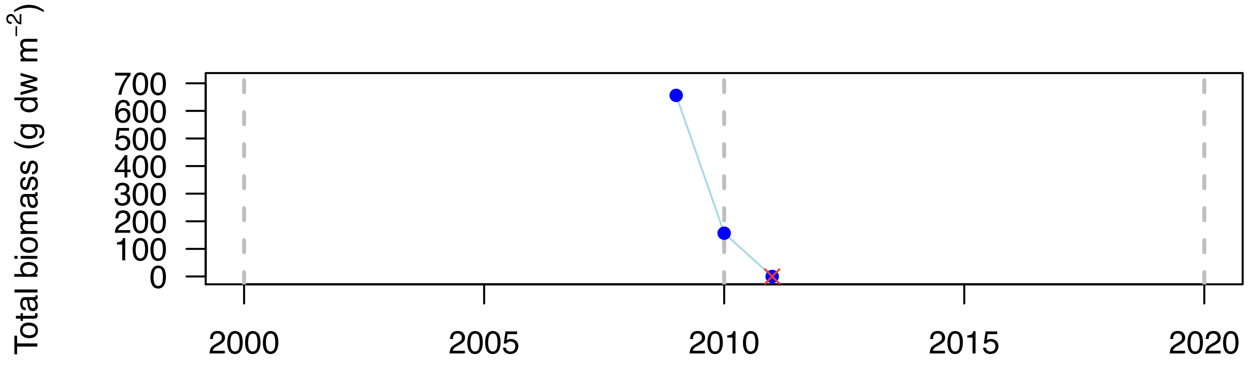

390_cover

Cabaço and Santos (unpublished)

SITE: Barra Faro (Portugal – Atlantic) – Zn (? m)

OVERALL: Net = –99.67 %;   Rate = NA % yr–1; Perc Final = NA % > decrease

DECADAL: NO (2 yr)

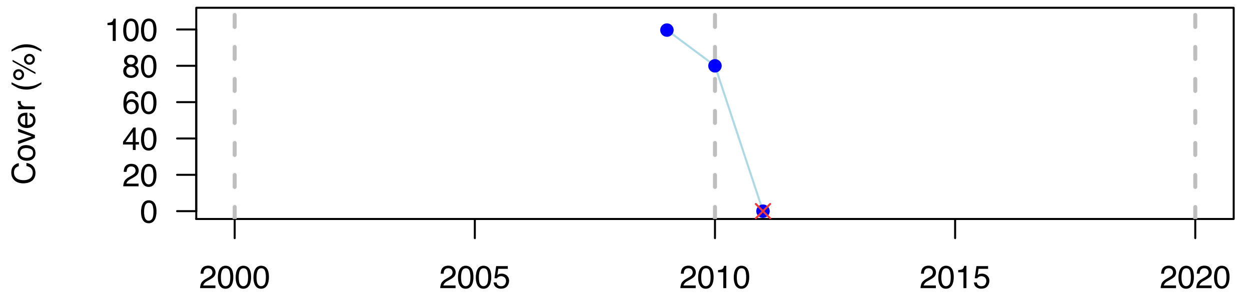

390_density
Cabaço and Santos (unpublished)
SITE: Barra Faro (Portugal – Atlantic) – Zn (? m)
OVERALL: Net = −17158.46 shoot m−2;   Rate = NA % yr−1; Perc Final = NA % > decrease
DECADAL: NO (2 yr)

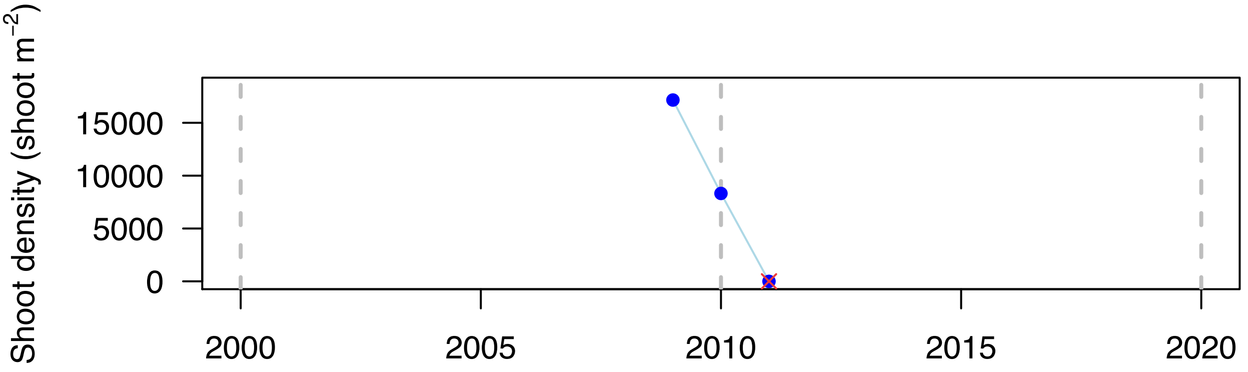

391_biomass
Cabaço and Santos (unpublished)
SITE: ETAR Tavira (Portugal – Atlantic) – Zn (? m)
OVERALL: Net = −206.87 g dw m−2;   Rate = NA % yr−1; Perc Final = NA % > decrease
DECADAL: NO (5 yr)

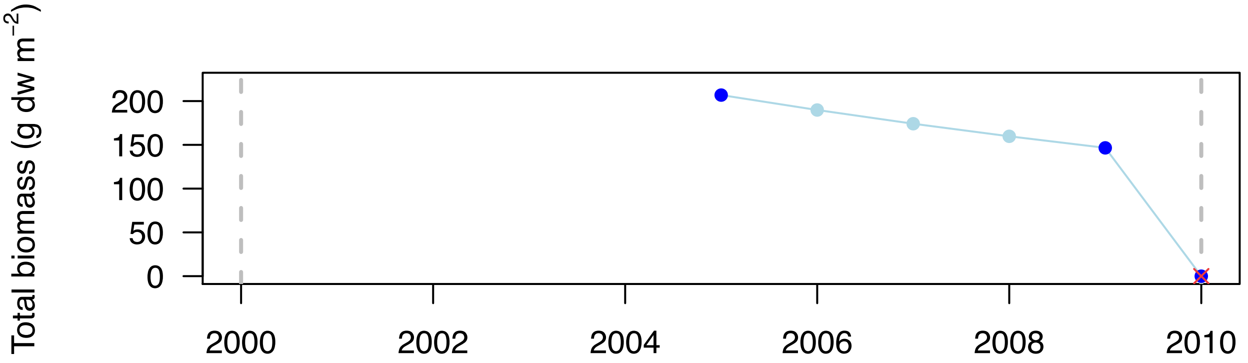

391_density

Cabaço and Santos (unpublished)

SITE: ETAR Tavira (Portugal – Atlantic) – Zn (? m)

OVERALL: Net = –3295.29 shoot m–2; Rate = NA % yr–1; Perc Final = NA % > decrease

DECADAL: NO (5 yr)

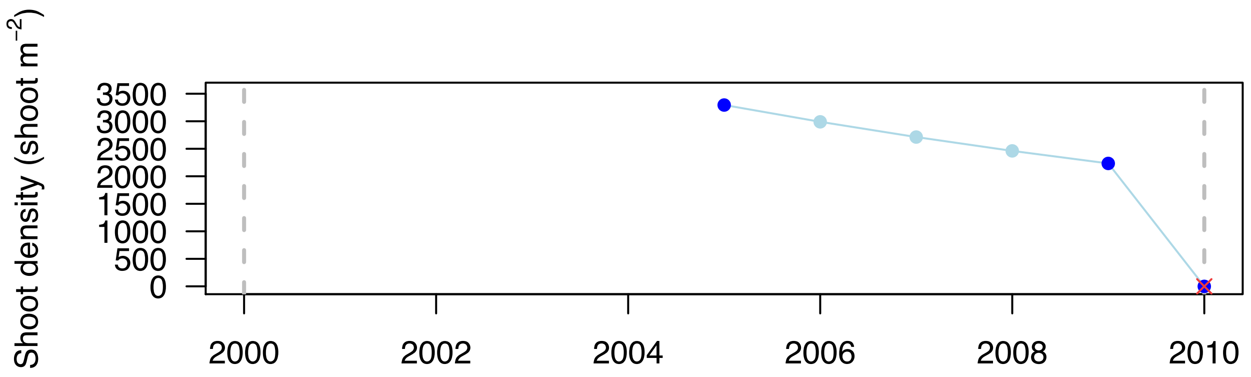

392_biomass

Cabaço and Santos (unpublished)

SITE: Albacora Tavira (Portugal – Atlantic) – Zn (? m)

OVERALL: Net = –411.23 g dw m–2; Rate = NA % yr–1; Perc Final = NA % > decrease

DECADAL: NO (5 yr)

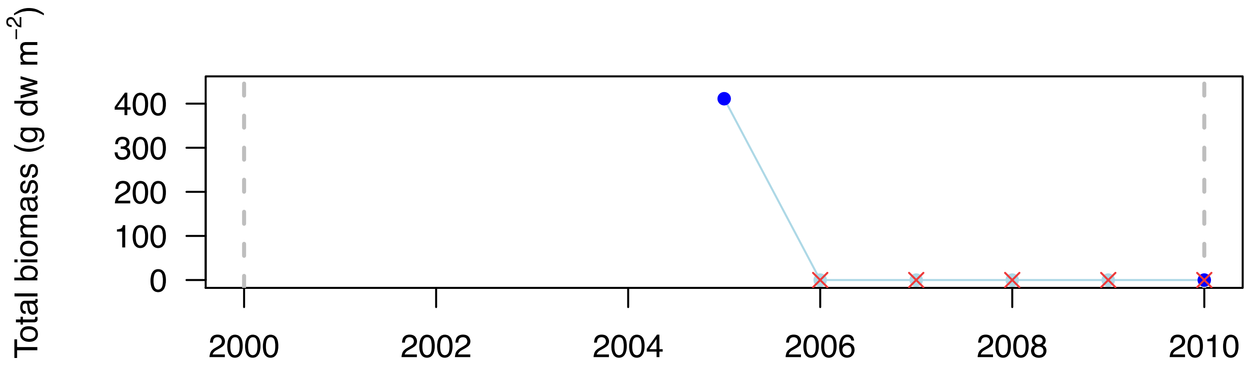

392_density

Cabaço and Santos (unpublished)

SITE: Albacora Tavira (Portugal – Atlantic) – Zn (? m)

OVERALL: Net = −5949.22 shoot m−2;   Rate = NA % yr−1; Perc Final = NA % > decrease

DECADAL: NO (5 yr)

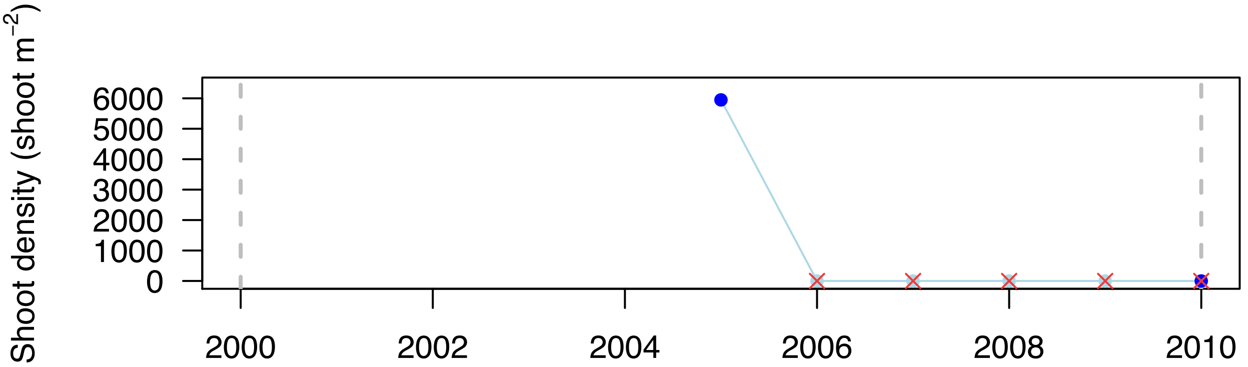

393_biomass

Cabaço et al. 2007, Cabaço and Santos (unpublished)

SITE: ETAR Arade Estuary (Portugal – Atlantic) – Zn (? m)

OVERALL: Net = −95.92 g dw m−2;   Rate = NA % yr−1; Perc Final = NA % > decrease

DECADAL: NO (4 yr)

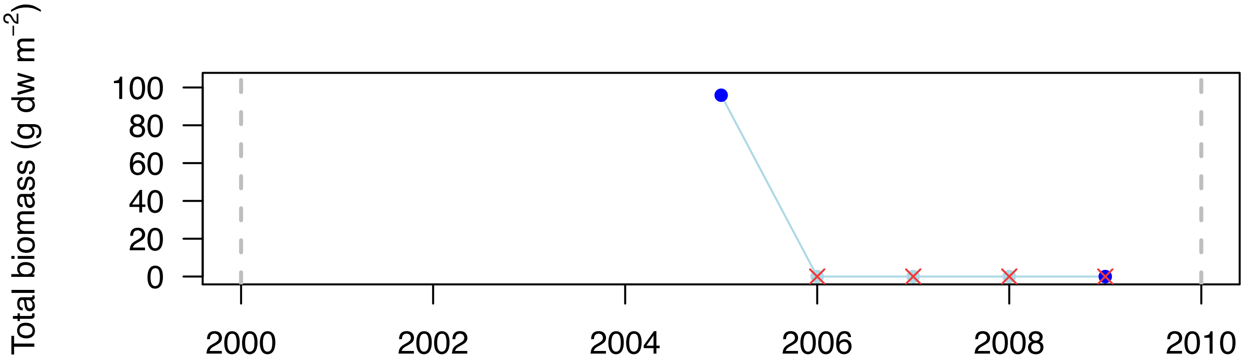

393_density

Cabaço et al. 2007, Cabaço and Santos (unpublished)

SITE: ETAR Arade Estuary (Portugal – Atlantic) – Zn (? m)

OVERALL: Net = −3450.11 shoot m−2;   Rate = NA % yr−1; Perc Final = NA % > decrease

DECADAL: NO (4 yr)

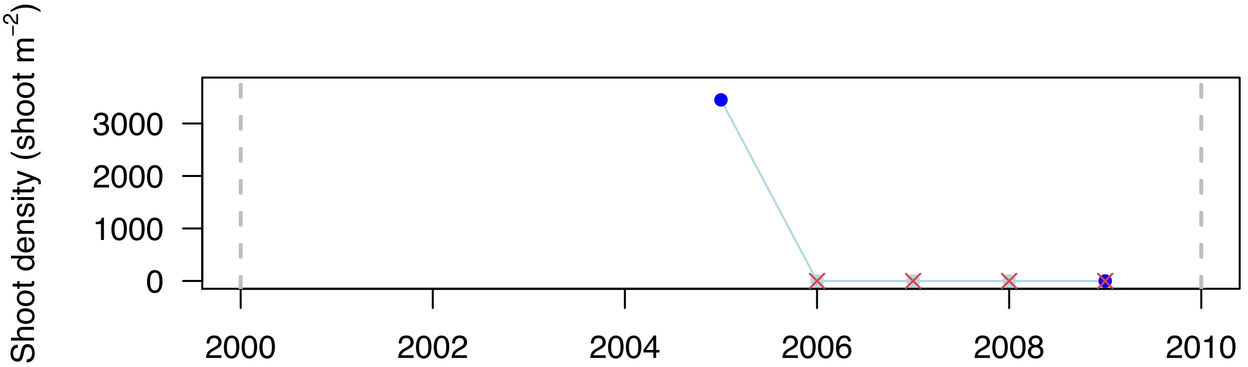

394_biomass

Cabaço et al. 2007, Cabaço and Santos (unpublished)

SITE: Arade Estuary 2 (Portugal – Atlantic) – Zn (? m)

OVERALL: Net = −190.76 g dw m−2;   Rate = NA % yr−1; Perc Final = NA % > decrease

DECADAL: NO (4 yr)

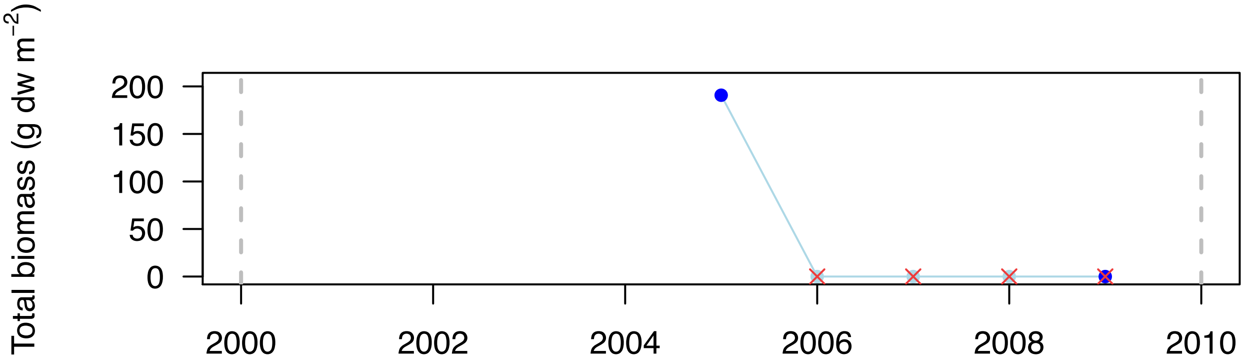

394_density
Cabaço et al. 2007, Cabaço and Santos (unpublished)
SITE: Arade Estuary 2 (Portugal – Atlantic) – Zn (? m)
OVERALL: Net = –3162.6 shoot m–2;   Rate = NA % yr–1; Perc Final = NA % > decrease
DECADAL: NO (4 yr)

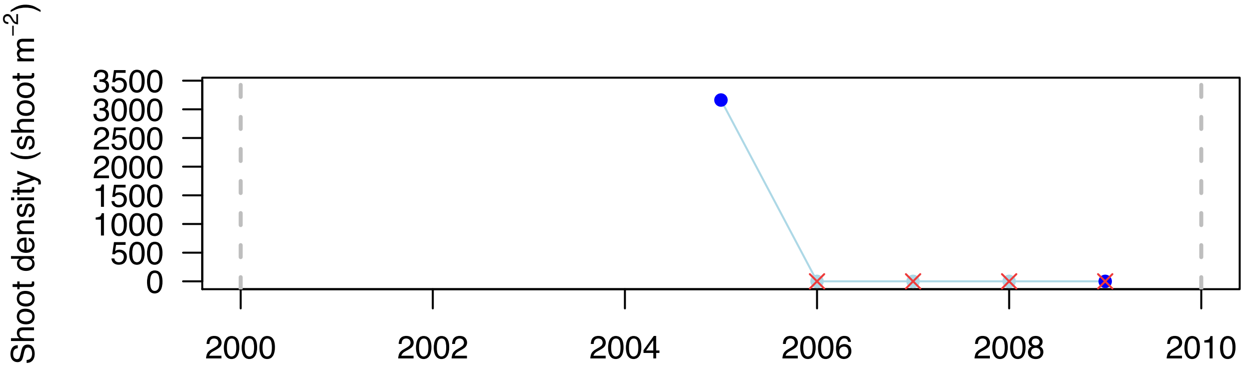

395_biomass
Cabaço et al. 2007, Cabaço and Santos (unpublished)
SITE: Arade Estuary 3 (Portugal – Atlantic) – Zn (? m)
OVERALL: Net = –177.51 g dw m–2;   Rate = NA % yr–1; Perc Final = NA % > decrease
DECADAL: NO (4 yr)

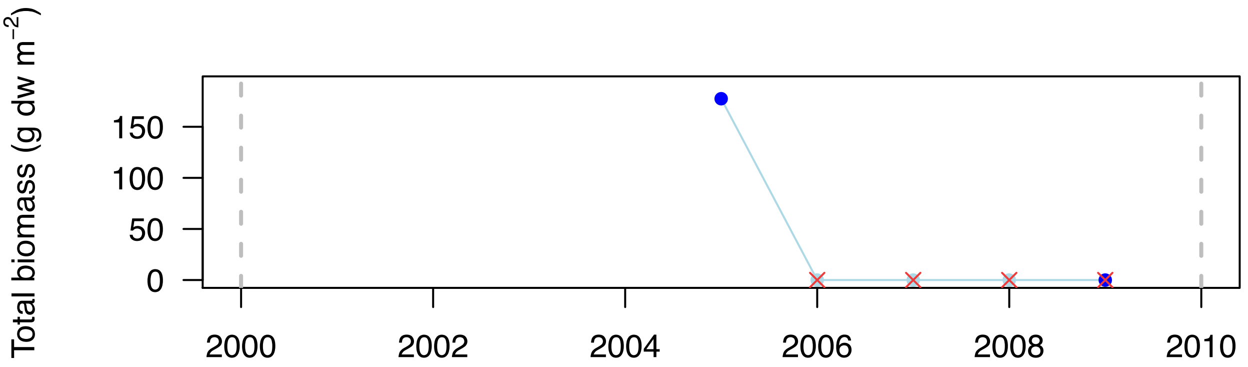

395_density   Cabaço et al. 2007, Cabaço and Santos (unpublished)

SITE: Arade Estuary 3 (Portugal – Atlantic) – Zn (? m)

OVERALL: Net = −1636.59 shoot m−2;   Rate = NA % yr−1; Perc Final = NA % > decrease

DECADAL: NO (4 yr)

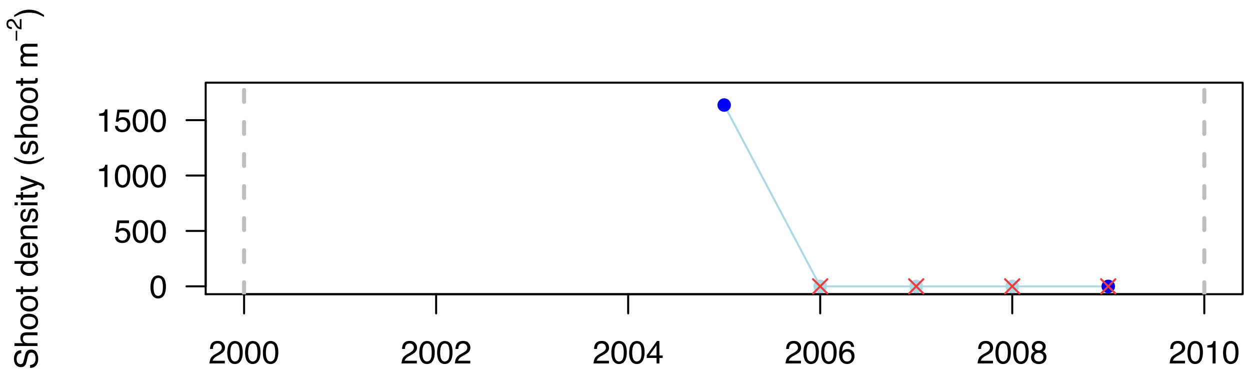

396_biomass   Cabaço et al. 2007, Cabaço and Santos (unpublished)

SITE: Arade Moinhos (Portugal – Atlantic) – Zn (? m)

OVERALL: Net = −272.64 g dw m−2;   Rate = NA % yr−1; Perc Final = NA % > decrease

DECADAL: NO (4 yr)

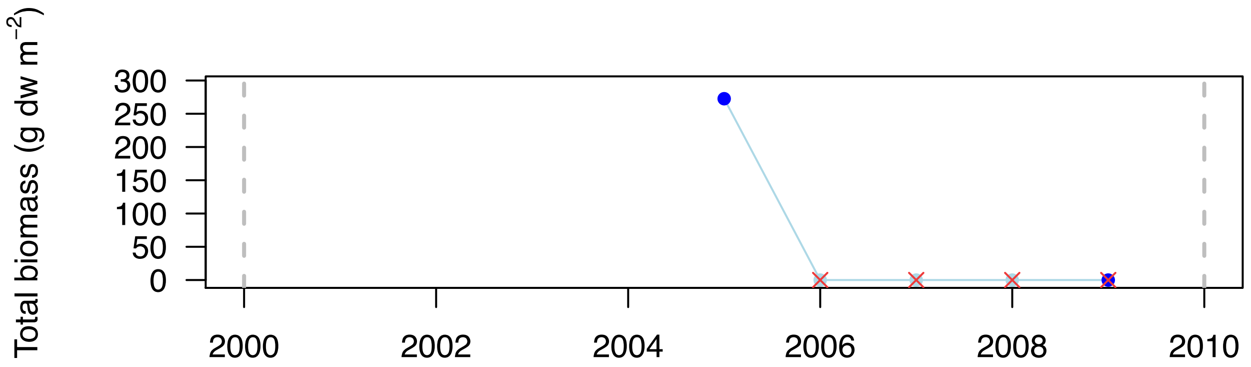

396_density
Cabaço et al. 2007, Cabaço and Santos (unpublished)
SITE: Arade Moinhos (Portugal – Atlantic) – Zn (? m)
OVERALL: Net = −3715.5 shoot m−2;   Rate = NA % yr−1; Perc Final = NA % > decrease
DECADAL: NO (4 yr)

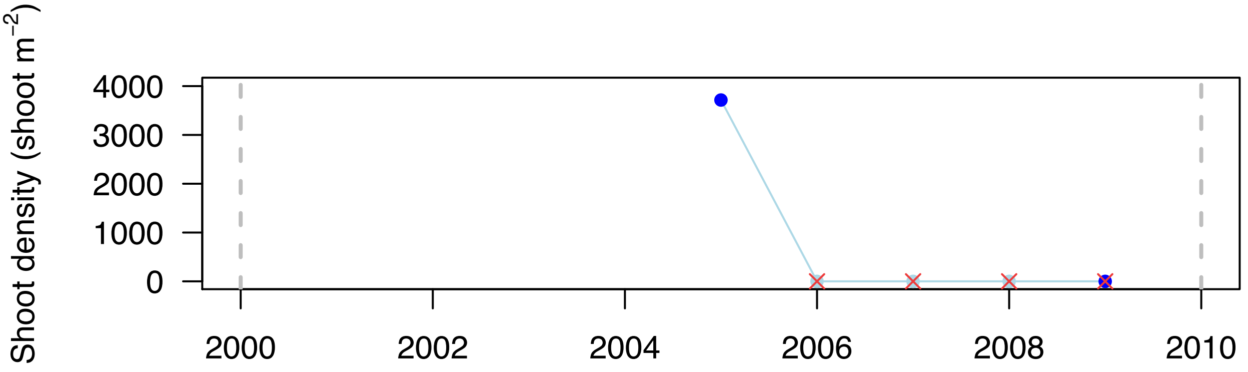

397_biomass
Cabaço et al. 2007, Cabaço and Santos (unpublished)
SITE: Guadiana Estuary (Portugal – Atlantic) – Zn (? m)
OVERALL: Net = −97.89 g dw m−2;   Rate = −27.5 % yr−1; Perc Final = 33 % > decrease
DECADAL: NO (4 yr)

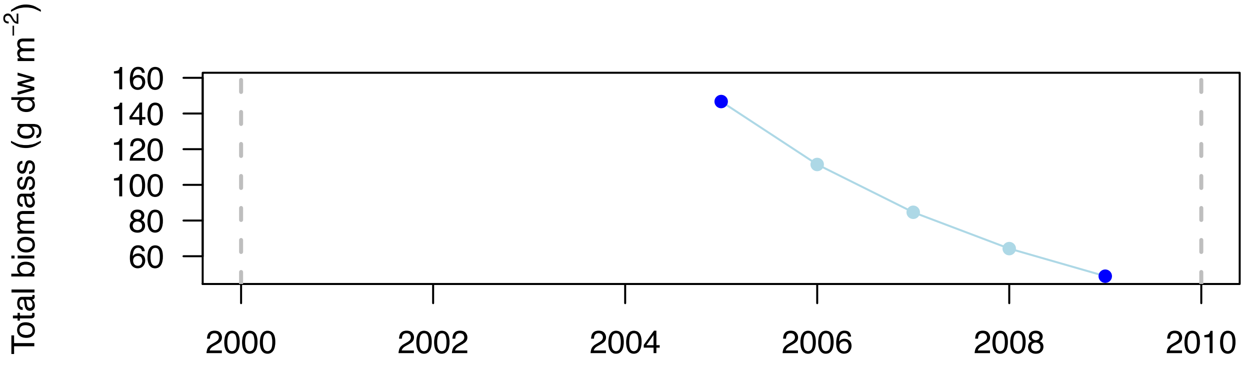

397_density

Cabaço et al. 2007, Cabaço and Santos (unpublished)

SITE: Guadiana Estuary (Portugal – Atlantic) – Zn (? m)

OVERALL: Net = 2441.79 shoot m–2;   Rate = 13.25 % yr–1; Perc Final = 170 % > increase

DECADAL: NO (4 yr)

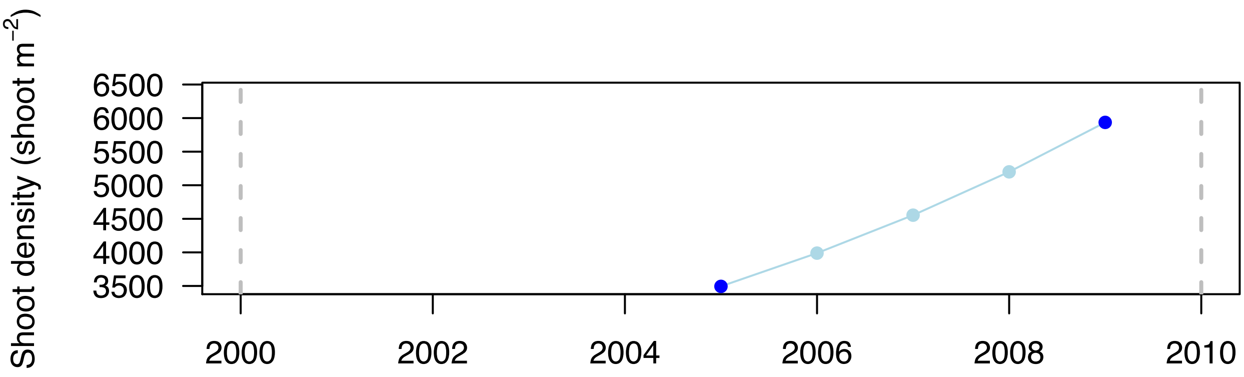

399_area

Pergent (unpublished)

SITE: Calvi (France – Mediterranean) – Po (–25 m)

OVERALL: Net = 0 ha;   Rate = 0 % yr–1; Perc Final = 100 % > no change

DECADAL: NO (6 yr)

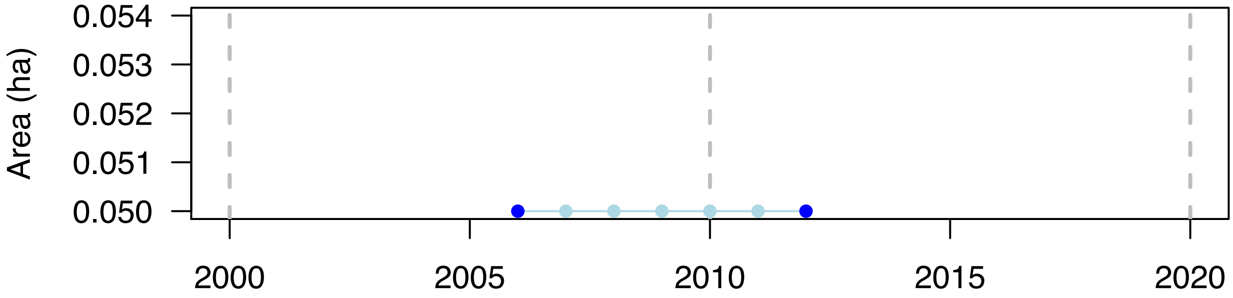

399_biomass

Pergent (unpublished)

SITE: Calvi (France – Mediterranean) – Po (–25 m)

OVERALL: Net = –52 g dw m–2;   Rate = –1.99 % yr–1; Perc Final = 89 % > no change

DECADAL: NO (6 yr)

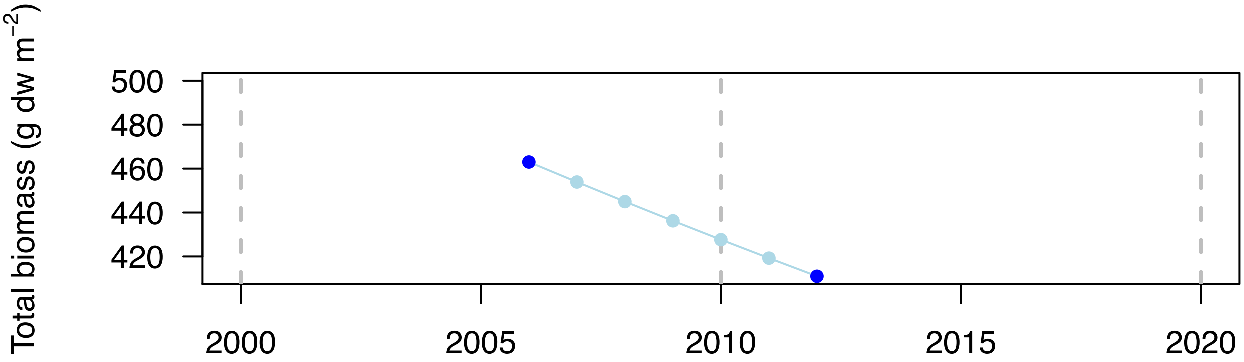

399_cover

Pergent (unpublished)

SITE: Calvi (France – Mediterranean) – Po (–25 m)

OVERALL: Net = –4 %;   Rate = –0.68 % yr–1; Perc Final = 96 % > no change

DECADAL: NO (6 yr)

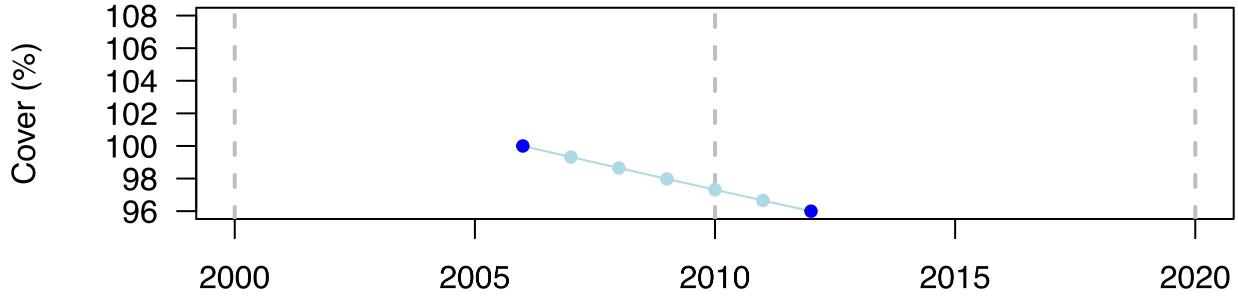

399_density

Pergent (unpublished)

SITE: Calvi (France – Mediterranean) – Po (–25 m)

OVERALL: Net = 0 shoot m–2; Rate = 0 % yr–1; Perc Final = 100 % > no change

DECADAL: NO (6 yr)

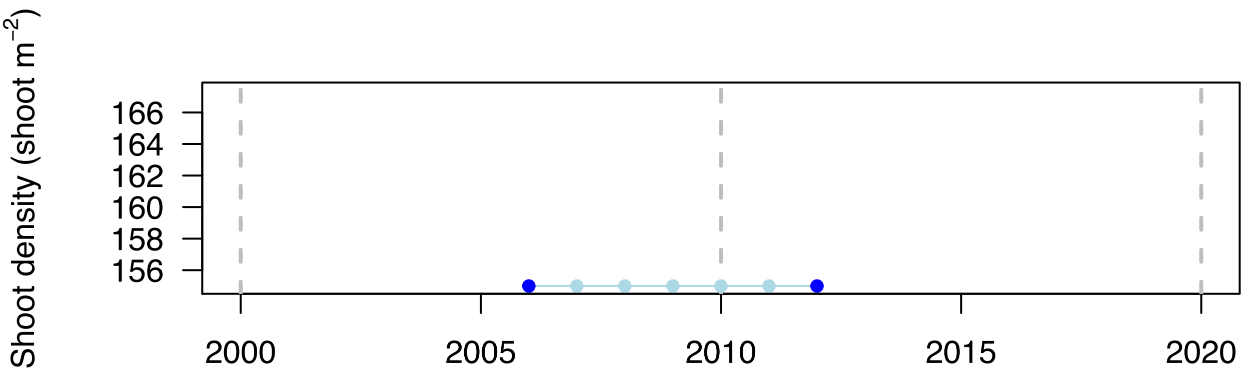

401_area

Pergent (unpublished)

SITE: Calvi (France – Mediterranean) – Po (–9 m)

OVERALL: Net = 0 ha; Rate = 0 % yr–1; Perc Final = 100 % > no change

DECADAL: NO (6 yr)

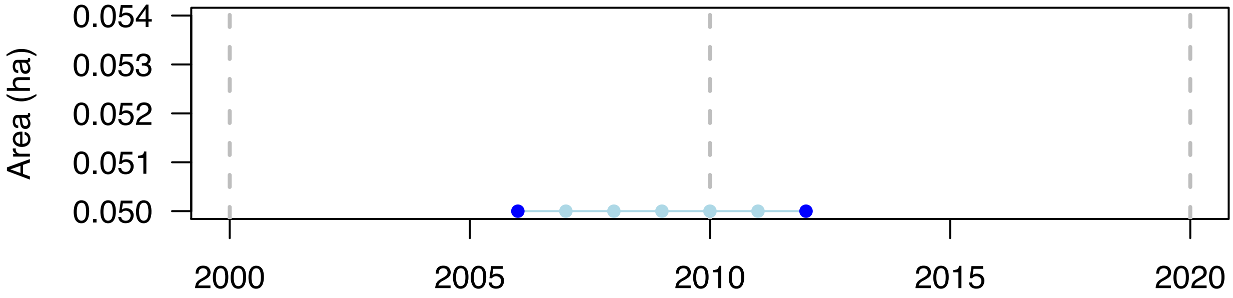

401_biomass

Pergent (unpublished)

SITE: Calvi (France – Mediterranean) – Po (–9 m)

OVERALL: Net = –64 g dw m–2;   Rate = –1.73 % yr–1; Perc Final = 90 % > no change

DECADAL: NO (6 yr)

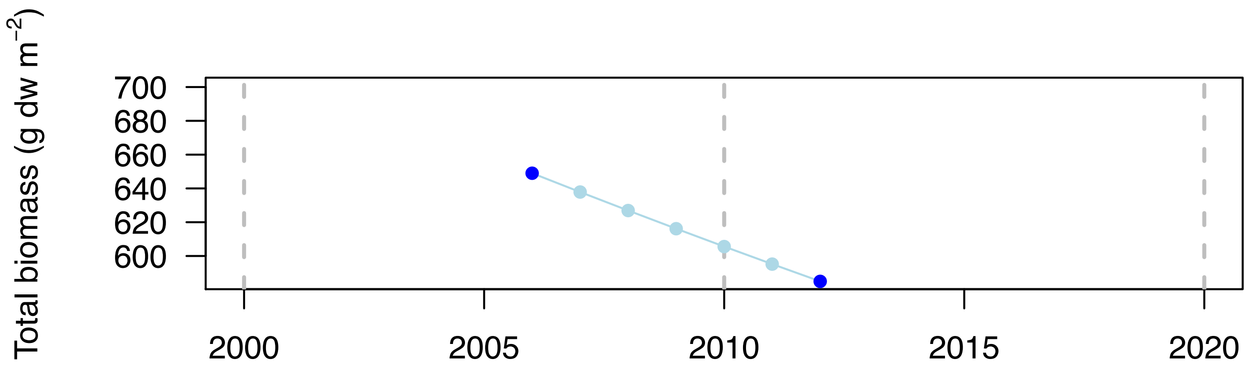

401_cover

Pergent (unpublished)

SITE: Calvi (France – Mediterranean) – Po (–9 m)

OVERALL: Net = 28 %;   Rate = 5.68 % yr–1; Perc Final = 141 % > increase

DECADAL: NO (6 yr)

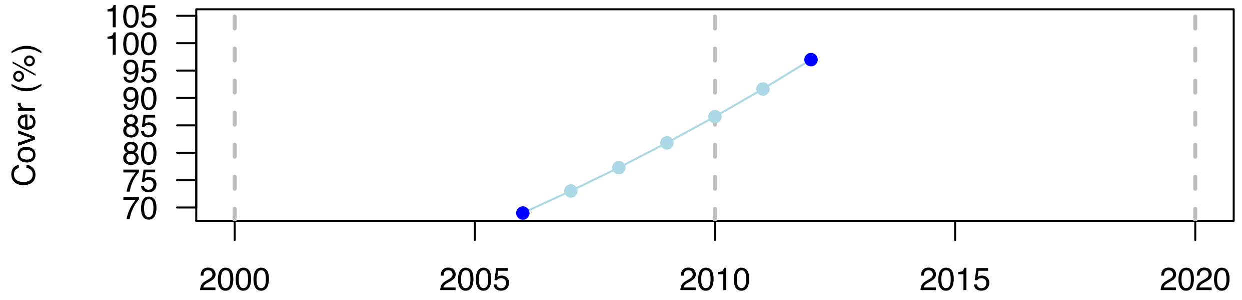

401_density

Pergent (unpublished)

SITE: Calvi (France – Mediterranean) – Po (–9 m)

OVERALL: Net = 164 shoot m–2;   Rate = 9 % yr–1; Perc Final = 172 % > increase

DECADAL: NO (6 yr)

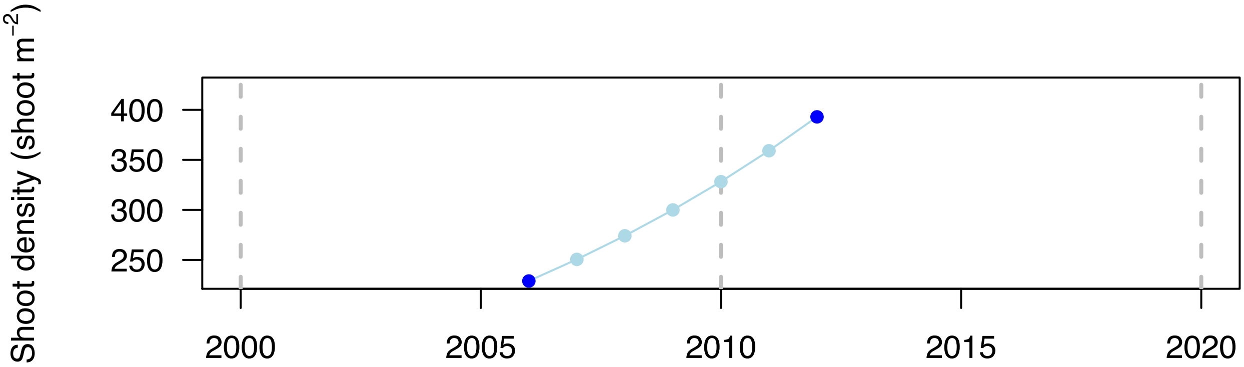

402_area

Bourcier 1989

SITE: Oest de Sanary (France – Mediterranean) – Po (? m)

OVERALL: Net = –220 ha;   Rate = –12.24 % yr–1; Perc Final = 48 % > decrease

DECADAL: NO (6 yr)

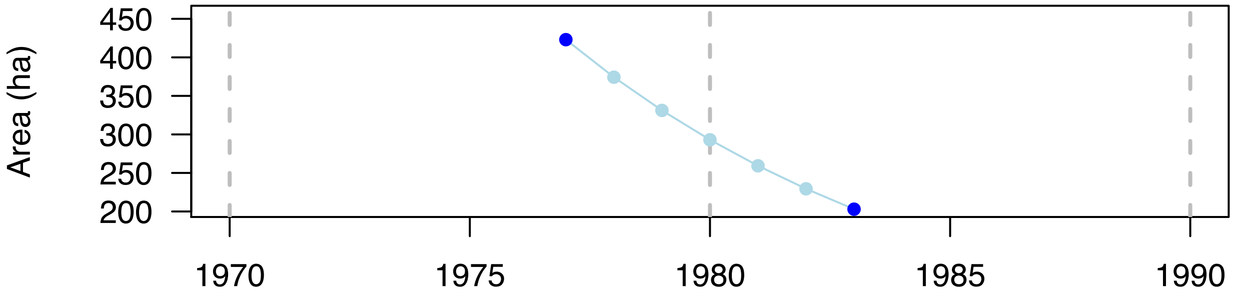

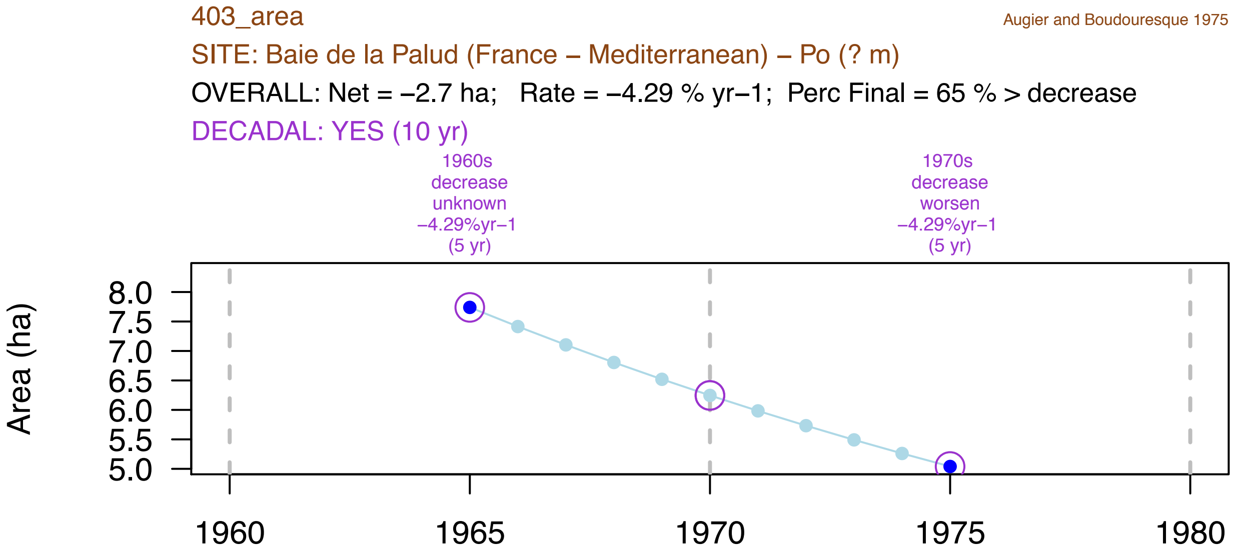
403_area
Augier and Boudouresque 1975
SITE: Baie de la Palud (France – Mediterranean) – Po (? m)
OVERALL: Net = −2.7 ha; Rate = −4.29 % yr−1; Perc Final = 65 % > decrease
DECADAL: YES (10 yr)
1960s
decrease
unknown
−4.29%yr−1
(5 yr)
1970s
decrease
worsen
−4.29%yr−1
(5 yr)
Area (ha)
8.0
7.5
7.0
6.5
6.0
5.5
5.0
1960
1965
1970
1975
1980

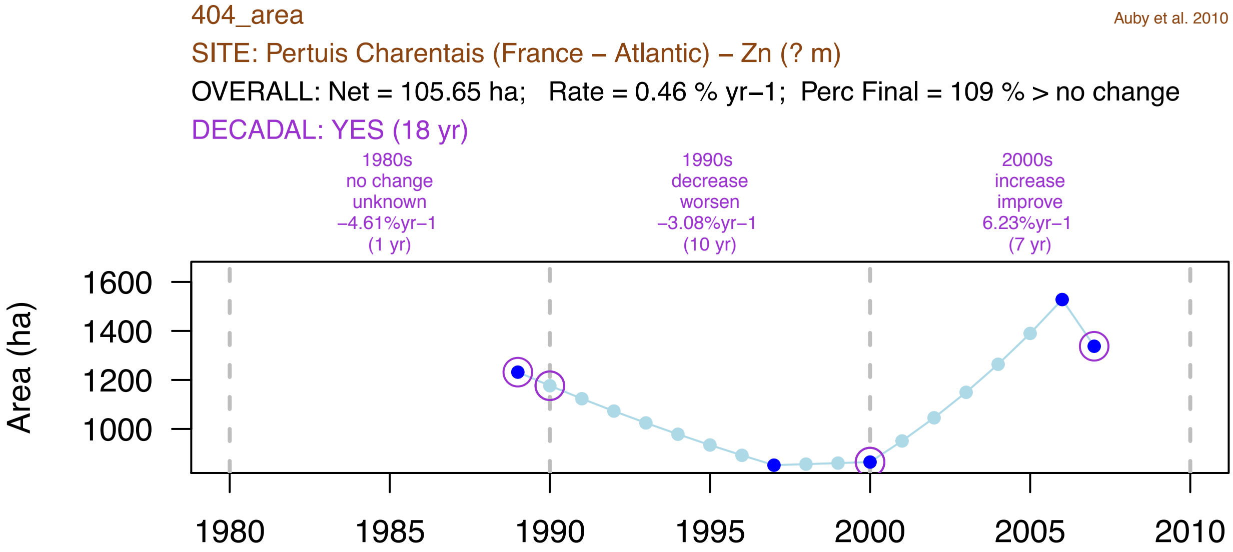
404_area
Auby et al. 2010
SITE: Pertuis Charentais (France – Atlantic) – Zn (? m)
OVERALL: Net = 105.65 ha; Rate = 0.46 % yr−1; Perc Final = 109 % > no change
DECADAL: YES (18 yr)
1980s
no change
unknown
−4.61%yr−1
(1 yr)
1990s
decrease
worsen
−3.08%yr−1
(10 yr)
2000s
increase
improve
6.23%yr−1
(7 yr)
Area (ha)
1600
1400
1200
1000
1980
1985
1990
1995
2000
2005
2010

405_density

Zupo et al. 2006

SITE: Lacco Ameno (Ischia) (Italy – Mediterranean) – Po (? m)

OVERALL: Net = −28.57 shoot m−2;   Rate = −1.05 % yr−1; Perc Final = 92 % > no change

DECADAL: YES (8 yr)

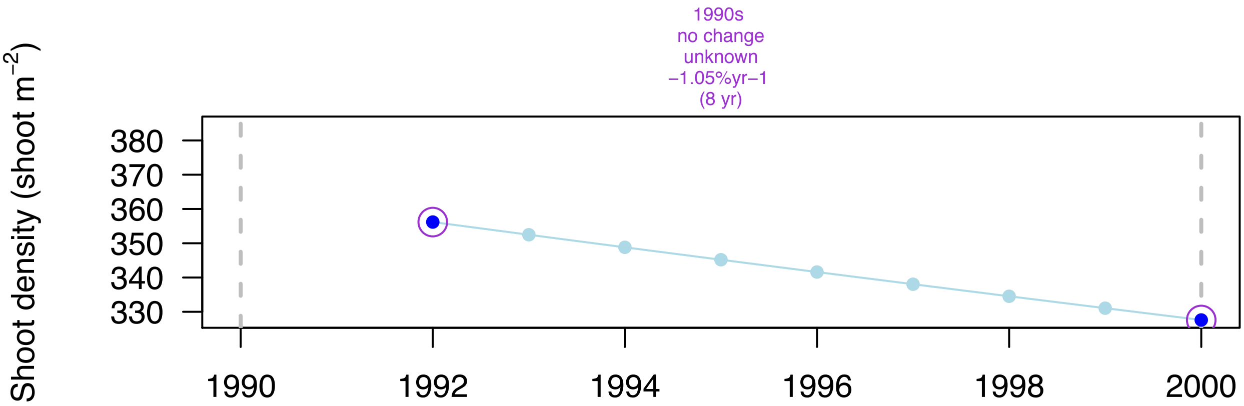

406_cover

Coll et al. 1999

SITE: Port de Cabrera (Spain – Mediterranean) – Po (? m)

OVERALL: Net = 14.6 %;   Rate = 2.64 % yr−1; Perc Final = 124 % > no change

DECADAL: YES (8 yr)

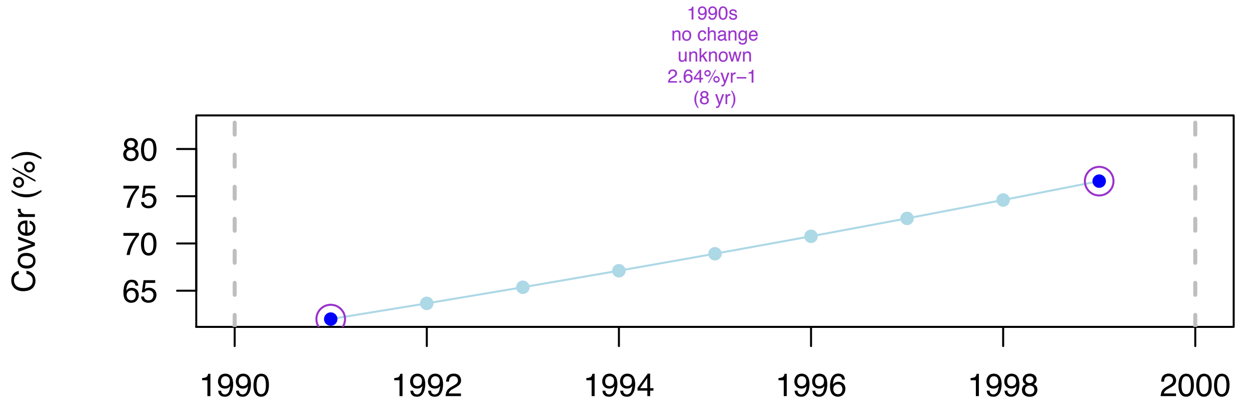

407_density

Guillén–Nieto and Ramos–Esplá 1994

SITE: Isla de Tabarca (Spain – Mediterranean) – Po (–19.3 m)

OVERALL: Net = 50 shoot m–2;   Rate = 59.73 % yr–1; Perc Final = 600 % > increase

DECADAL: NO (3 yr)

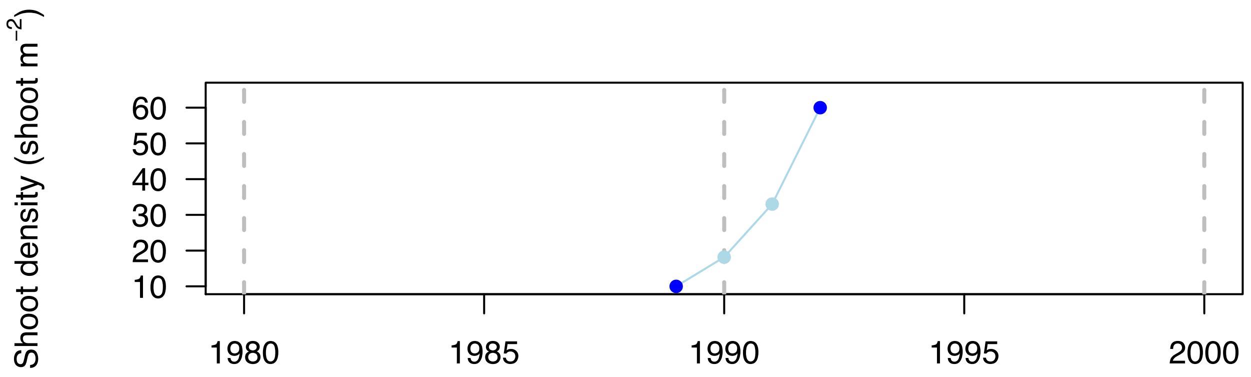

408_area

Ruiz et al. 1993, Ruiz and Romero 2003

SITE: Levante Bay (Spain – Mediterranean) – Po (? m)

OVERALL: Net = –3.65 ha;   Rate = –1.98 % yr–1; Perc Final = 79 % > decrease

DECADAL: YES (12 yr)

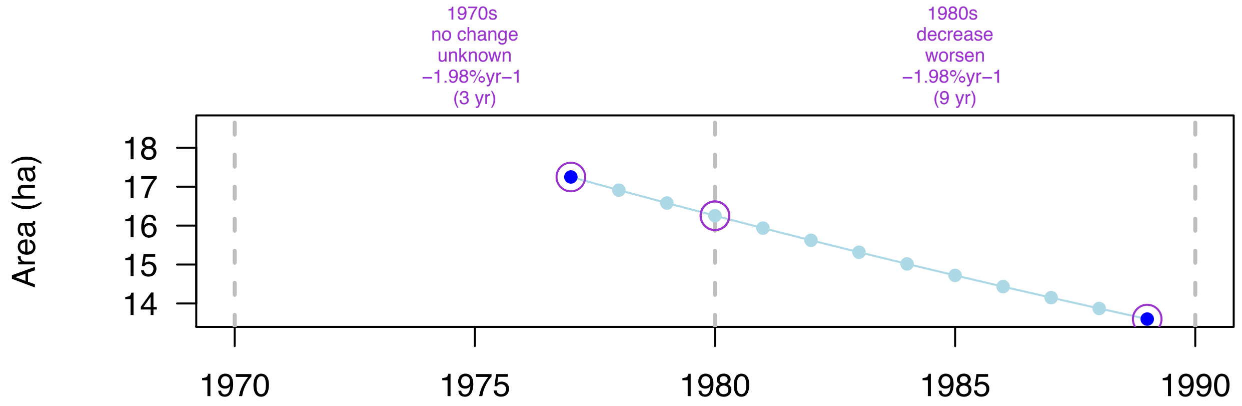

410_area

Montefalcone et al. 2013

SITE: Genova Vesima (Italy – Mediterranean) – Po (–3.3 m)

OVERALL: Net = –3.2 ha;   Rate = NA % yr–1; Perc Final = NA % > decrease

DECADAL: YES (15 yr)

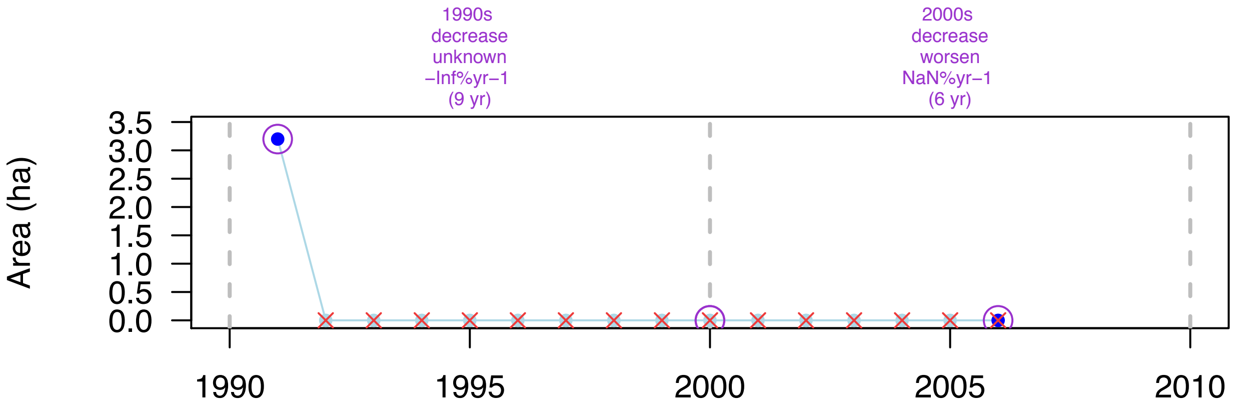

416_area

Montefalcone et al. 2013

SITE: Ventimiglia (Italy – Mediterranean) – Po (–12.5 m)

OVERALL: Net = 84.098 ha;   Rate = 2.72 % yr–1; Perc Final = 155 % > increase

DECADAL: YES (16 yr)

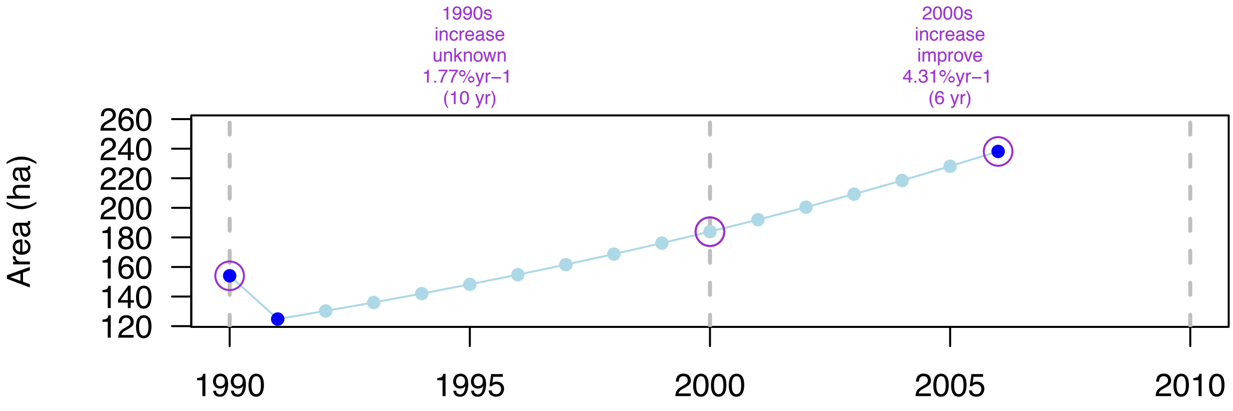

417_area

Montefalcone et al. 2013

SITE: Sanremo (Italy – Mediterranean) – Po (–16.4 m)

OVERALL: Net = 32.683 ha;   Rate = 0.26 % yr–1; Perc Final = 104 % > no change

DECADAL: YES (16 yr)

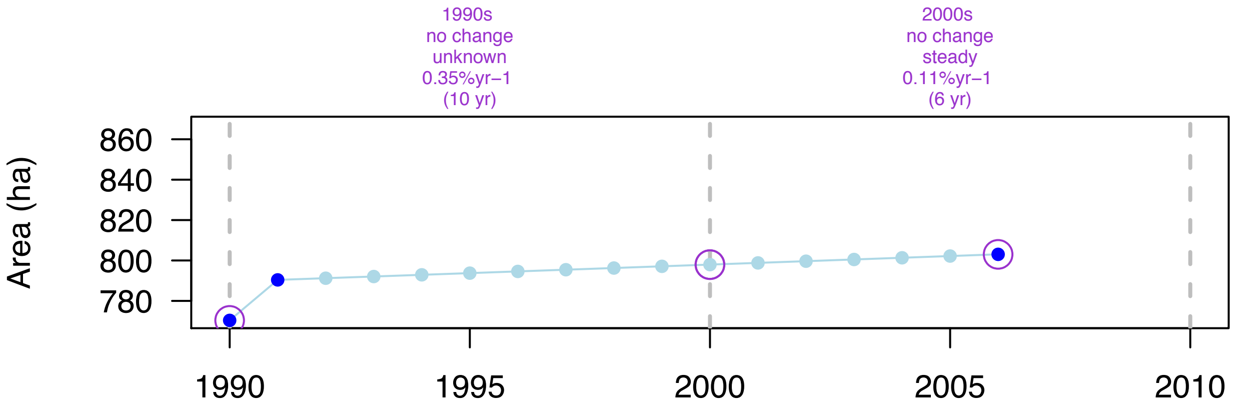

418_area

Montefalcone et al. 2013

SITE: Santo Stefano al Mare (Italy – Mediterranean) – Po (–18.2 m)

OVERALL: Net = 40.399 ha;   Rate = 0.74 % yr–1; Perc Final = 113 % > increase

DECADAL: YES (16 yr)

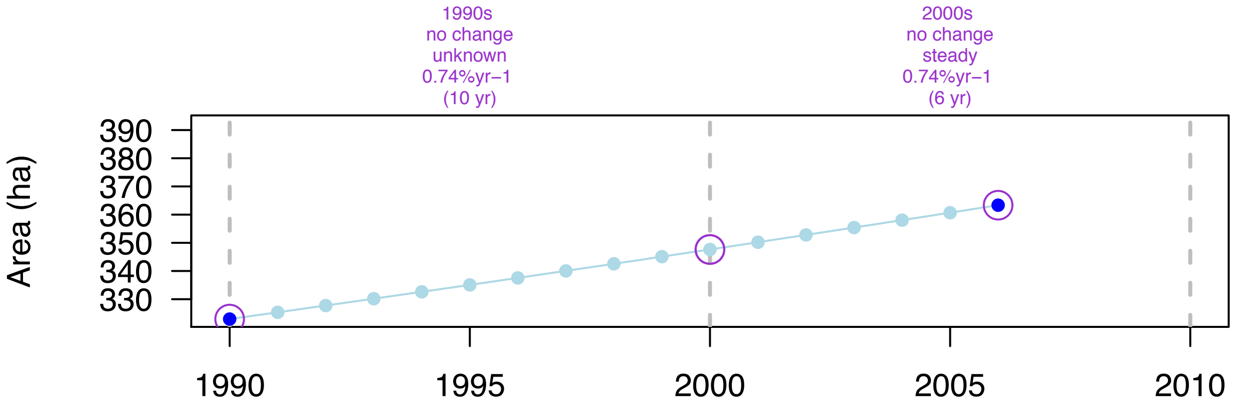

419_area

Montefalcone et al. 2013

SITE: Porto Maurizio (Italy – Mediterranean) – Po (–17.5 m)

OVERALL: Net = 31.575 ha;   Rate = 0.3 % yr–1; Perc Final = 105 % > no change

DECADAL: YES (16 yr)

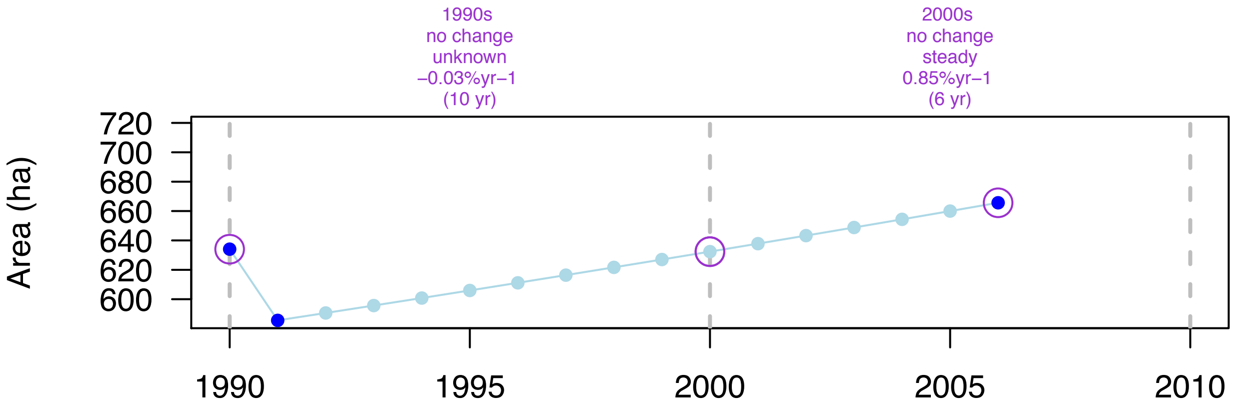

420_area

Montefalcone et al. 2013

SITE: Diano Marina (Italy – Mediterranean) – Po (–16.4 m)

OVERALL: Net = –26.938 ha;   Rate = –0.42 % yr–1; Perc Final = 94 % > no change

DECADAL: YES (16 yr)

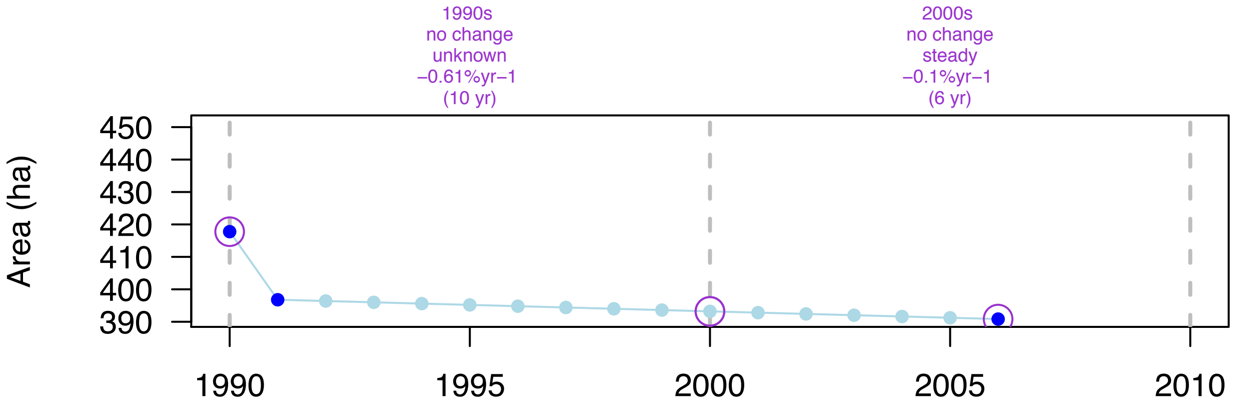

421_area

Montefalcone et al. 2013

SITE: Laigueglia (Italy – Mediterranean) – Po (–15 m)

OVERALL: Net = –141.205 ha;   Rate = –4.49 % yr–1; Perc Final = 49 % > decrease

DECADAL: YES (16 yr)

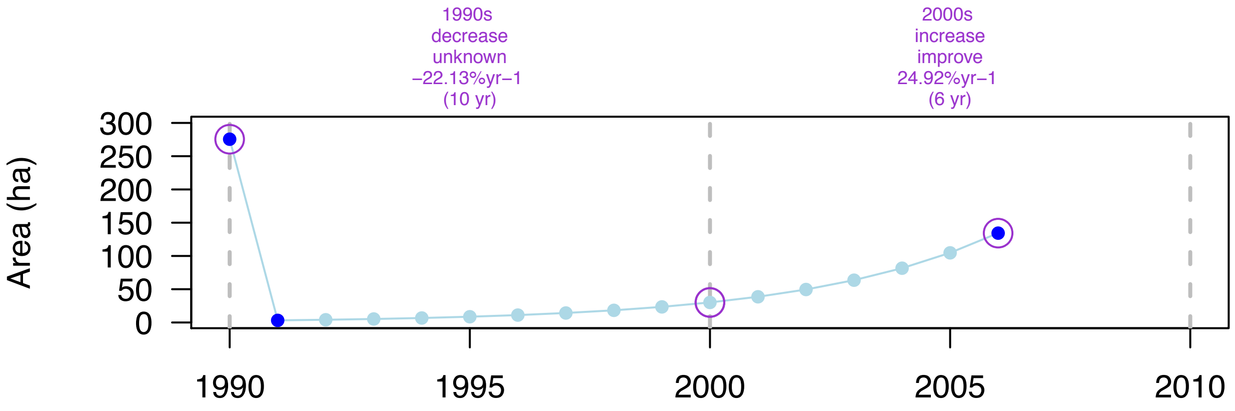

422_area

Montefalcone et al. 2013

SITE: Isola Gallinara (Italy – Mediterranean) – Po (–10.6 m)

OVERALL: Net = –65.434 ha;   Rate = –3.37 % yr–1; Perc Final = 58 % > decrease

DECADAL: YES (16 yr)

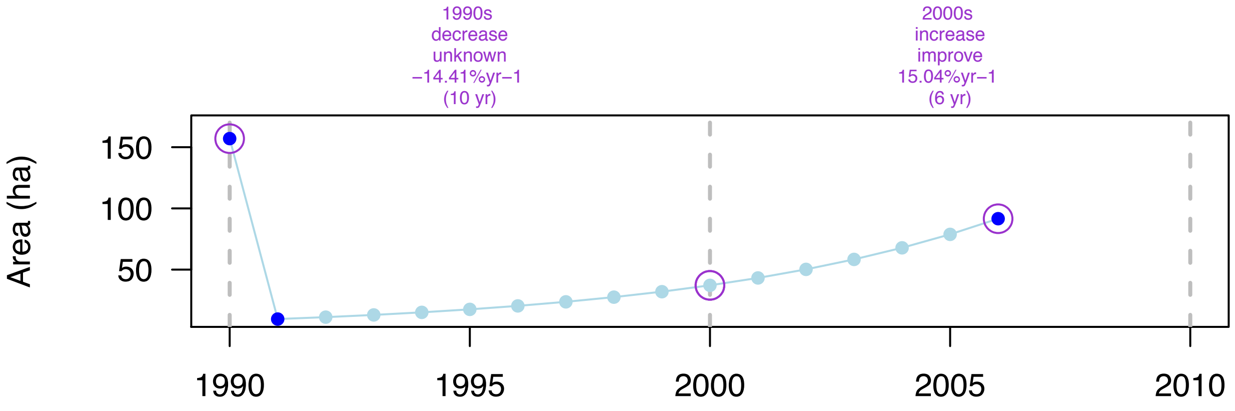

423_area

Montefalcone et al. 2013

SITE: Albenga – Loano (Italy – Mediterranean) – Po (–14.3 m)

OVERALL: Net = –30.896 ha; Rate = –0.52 % yr–1; Perc Final = 92 % > no change

DECADAL: YES (16 yr)

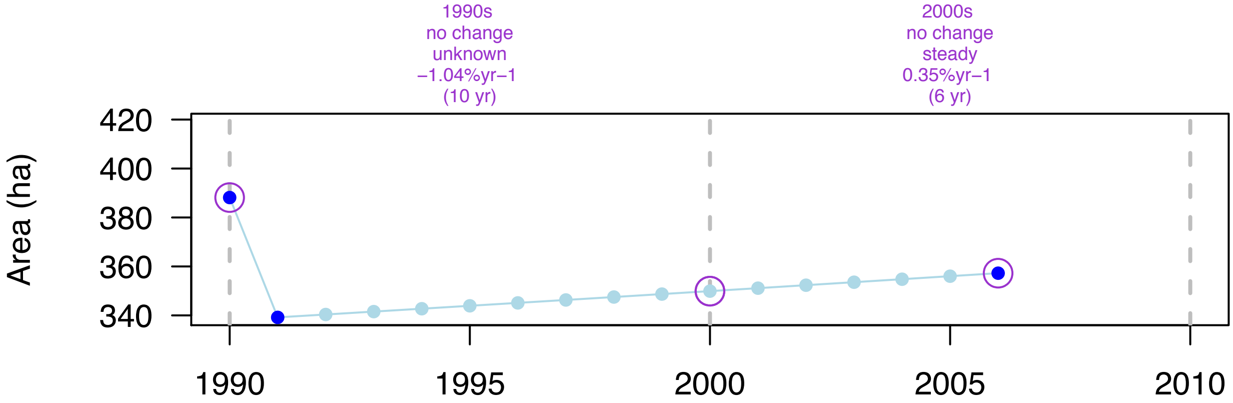

424_area

Montefalcone et al. 2013

SITE: Pietra Ligure (Italy – Mediterranean) – Po (–9 m)

OVERALL: Net = –64.962 ha; Rate = –13.07 % yr–1; Perc Final = 12 % > decrease

DECADAL: YES (16 yr)

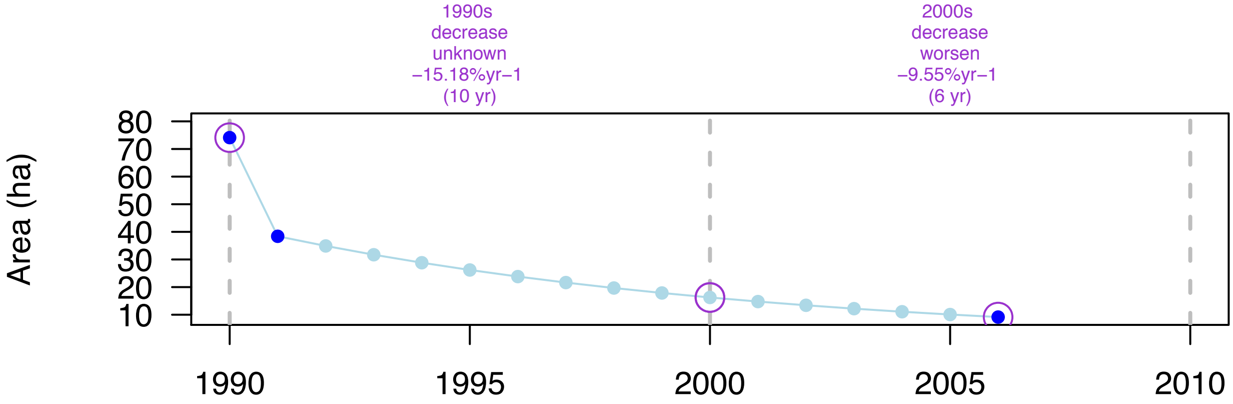

425_area

Montefalcone et al. 2013

SITE: Noli – Spotorno (Italy – Mediterranean) – Po (−14.2 m)

OVERALL: Net = −23.252 ha; Rate = −5.78 % yr−1; Perc Final = 40 % > decrease

DECADAL: YES (16 yr)

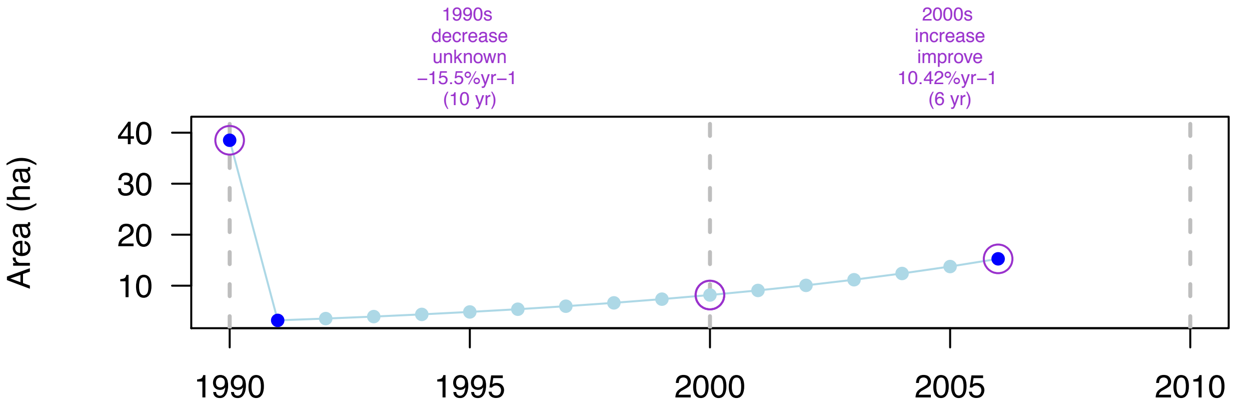

426_area

Montefalcone et al. 2013

SITE: Isola di Bergeggi (Italy – Mediterranean) – Po (−15.7 m)

OVERALL: Net = −43.359 ha; Rate = −4.88 % yr−1; Perc Final = 46 % > decrease

DECADAL: YES (16 yr)

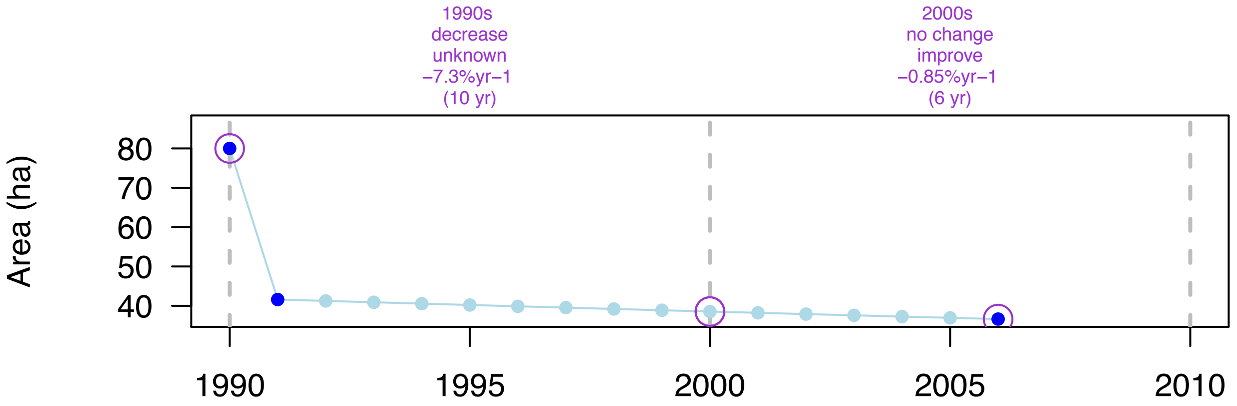

427_area

Montefalcone et al. 2013

SITE: Vado Ligure (Italy – Mediterranean) – Po (–18.8 m)

OVERALL: Net = –41.3 ha;   Rate = –12.81 % yr–1; Perc Final = 13 % > decrease

DECADAL: YES (16 yr)

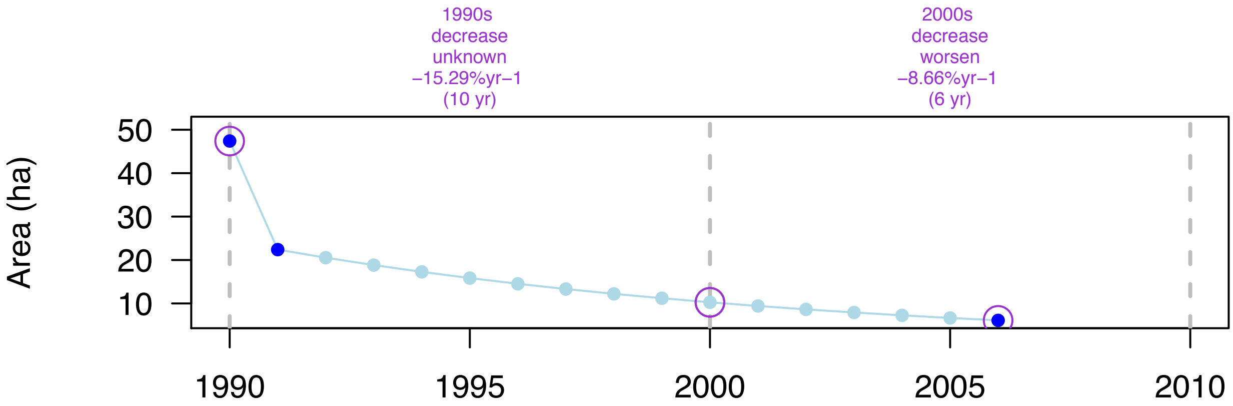

428_area

Montefalcone et al. 2013

SITE: Savona (Italy – Mediterranean) – Po (? m)

OVERALL: Net = 5.374 ha;   Rate = 1.93 % yr–1; Perc Final = 134 % > increase

DECADAL: YES (15 yr)

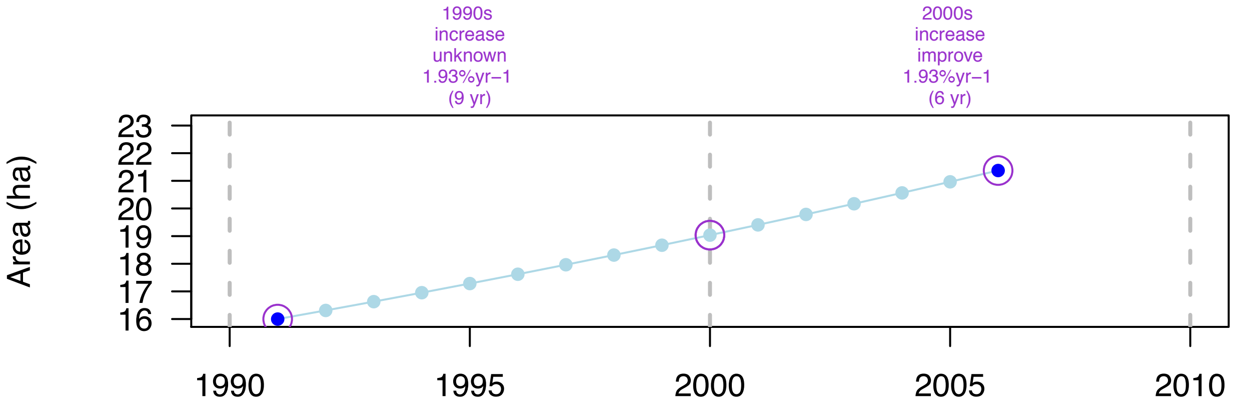

429_area

Montefalcone et al. 2013

SITE: Albissola Marina – Celle Ligure (Italy – Mediterranean) – Po (? m)

OVERALL: Net = 34.583 ha; Rate = 4.52 % yr–1; Perc Final = 206 % > increase

DECADAL: YES (16 yr)

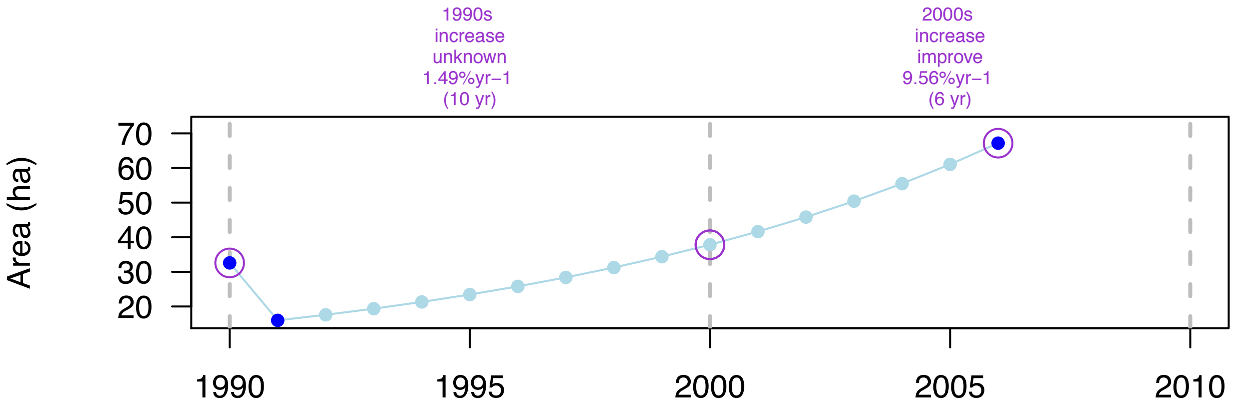

430_area

Montefalcone et al. 2013

SITE: Arenzano (Italy – Mediterranean) – Po (? m)

OVERALL: Net = 40.082 ha; Rate = 1.9 % yr–1; Perc Final = 136 % > increase

DECADAL: YES (16 yr)

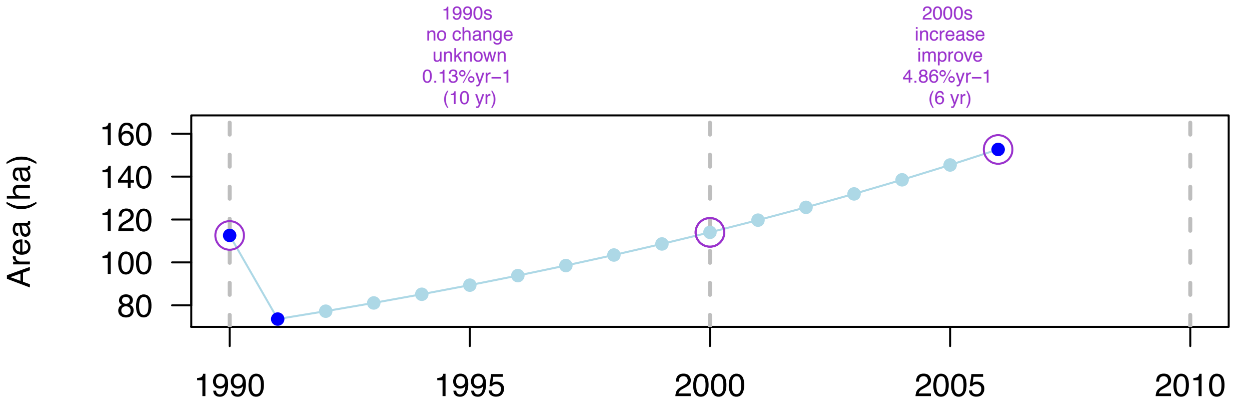

431_cover

Sandulli et al. 1994, Bianchi et al. 2009, Montefalcone et al. 2007 (a)

SITE: Bergeggi (Italy – Mediterranean) – Cn (–8 m)

OVERALL: Net = –90 %; Rate = NA % yr–1; Perc Final = NA % > decrease

DECADAL: YES (17 yr)

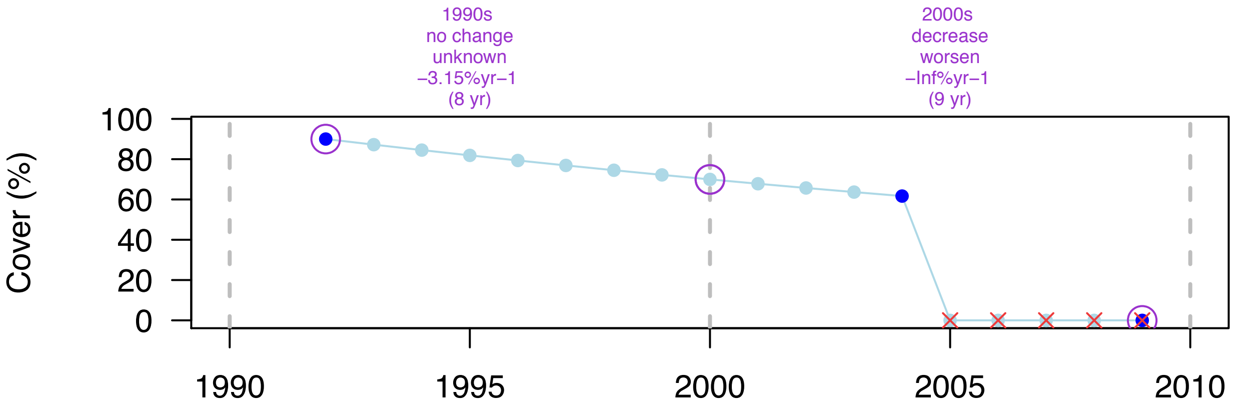

432_cover

Vetere and Pessani 1989, Sandulli et al. 1994, Montefalcone et al 2007a, Bianchi et al. 2009, Oprandi et al. 2014

SITE: Bergeggi (Italy – Mediterranean) – Po (? m)

OVERALL: Net = 1.4 %; Rate = 0.08 % yr–1; Perc Final = 102 % > no change

DECADAL: YES (20 yr)

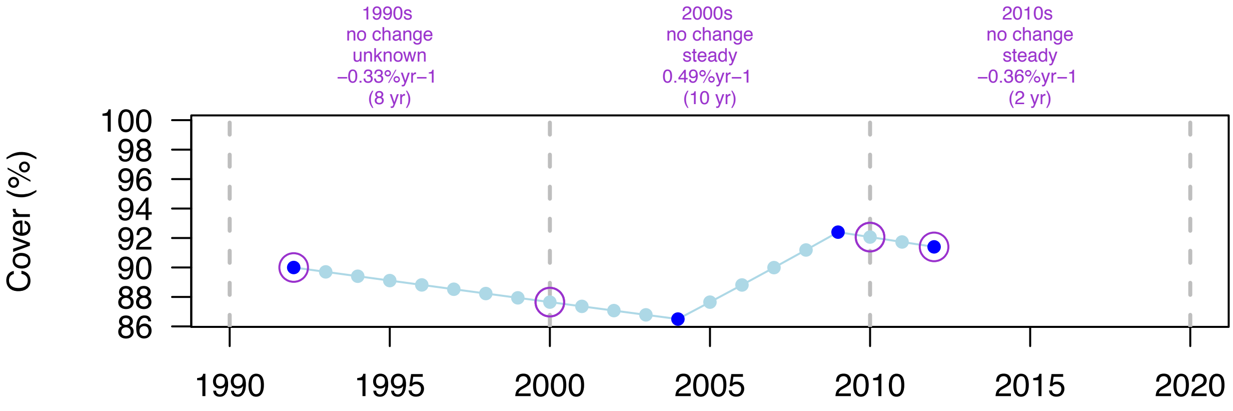

432_lowerlimit   Vetere and Pessani 1989, Sandulli et al. 1994, Montefalcone et al 2007a, Bianchi et al. 2009, Oprandi et al. 2014

SITE: Bergeggi (Italy – Mediterranean) – Po (? m)

OVERALL: Net = 0.3 m;   Rate = 0.06 % yr–1; Perc Final = 102 % > no change

DECADAL: YES (25 yr)

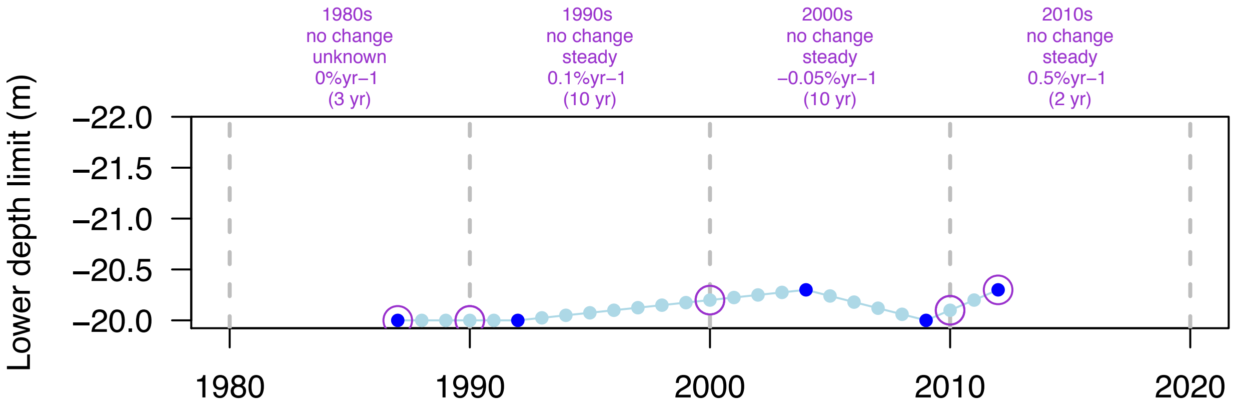

432_upperlimit   Vetere and Pessani 1989, Sandulli et al. 1994, Montefalcone et al 2007a, Bianchi et al. 2009, Oprandi et al. 2014

SITE: Bergeggi (Italy – Mediterranean) – Po (? m)

OVERALL: Net = 1.6 m;   Rate = 0.89 % yr–1; Perc Final = 125 % > increase

DECADAL: YES (25 yr)

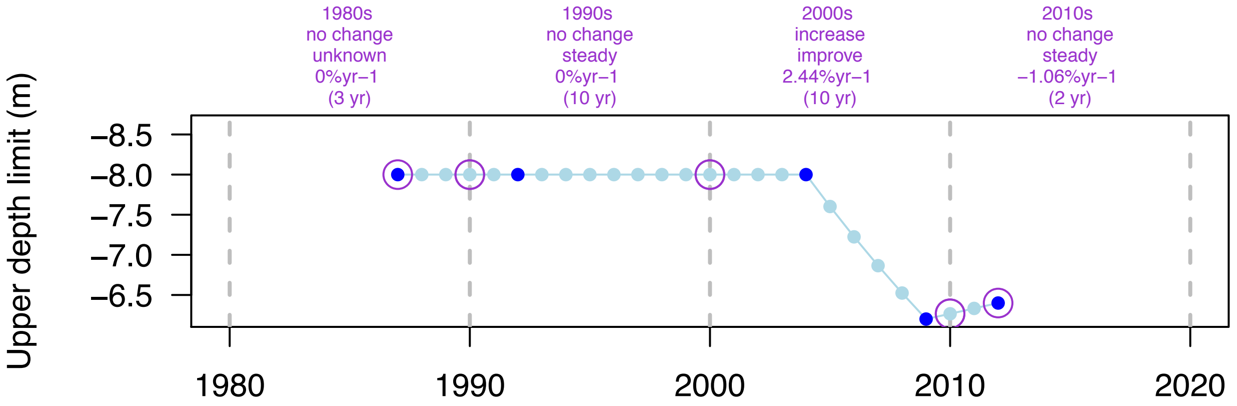

433_cover

Bianchi and Sandulli 1992, Oprandi et al. 2014

SITE: Noli (Italy – Mediterranean) – Cn (–4 m)

OVERALL: Net = –100 %;   Rate = NA % yr–1; Perc Final = NA % > decrease

DECADAL: YES (20 yr)

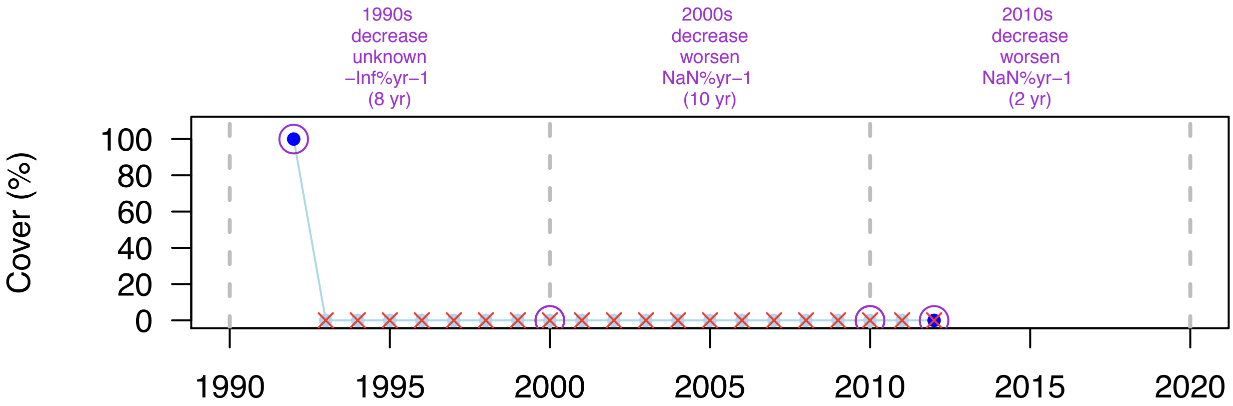

434_cover

Bianchi and Sandulli 1992, Oprandi et al. 2014

SITE: Noli (Italy – Mediterranean) – Po (? m)

OVERALL: Net = 11 %;   Rate = 0.69 % yr–1; Perc Final = 115 % > no change

DECADAL: YES (20 yr)

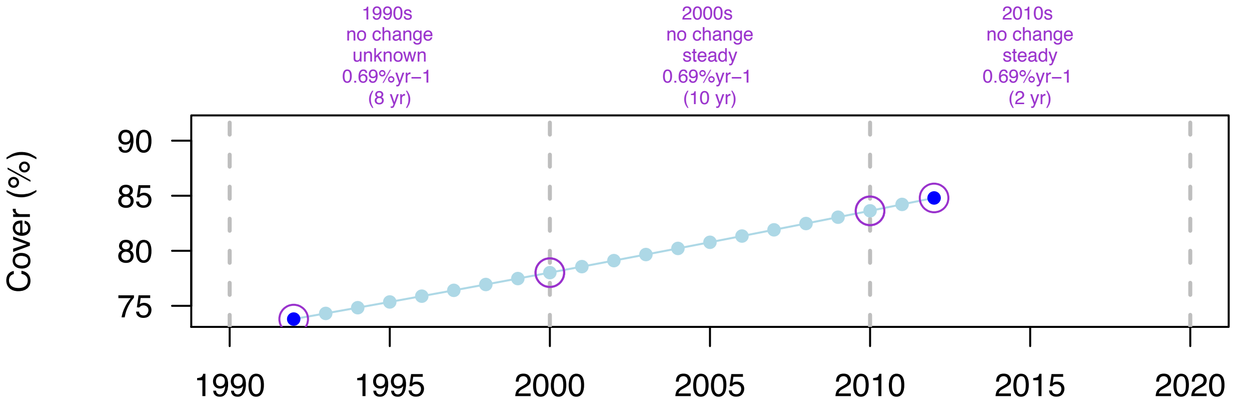

434_lowerlimit

Bianchi and Sandulli 1992, Oprandi et al. 2014

SITE: Noli (Italy – Mediterranean) – Po (? m)

OVERALL: Net = −1.3 m;   Rate = −0.31 % yr−1; Perc Final = 94 % > no change

DECADAL: YES (20 yr)

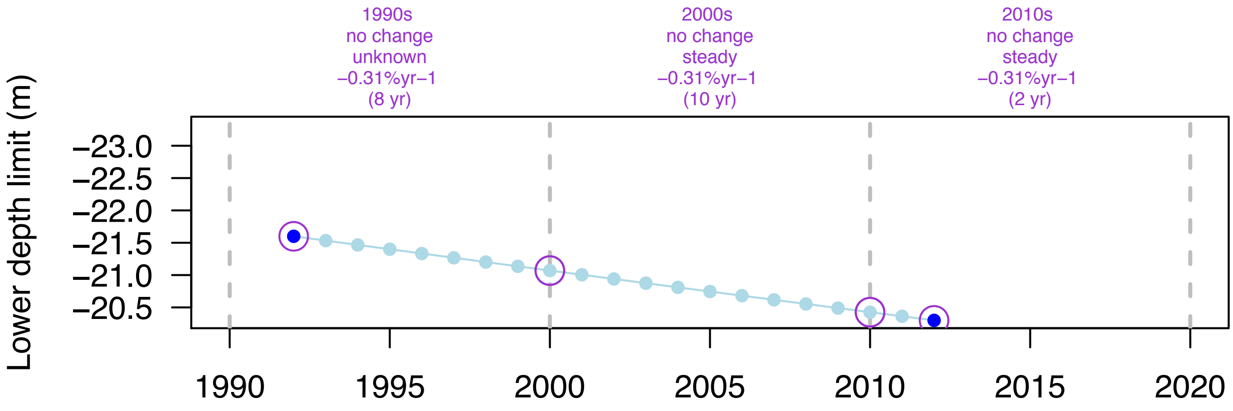

434_upperlimit

Bianchi and Sandulli 1992, Oprandi et al. 2014

SITE: Noli (Italy – Mediterranean) – Po (? m)

OVERALL: Net = 0 m;   Rate = 0 % yr−1; Perc Final = 100 % > no change

DECADAL: YES (20 yr)

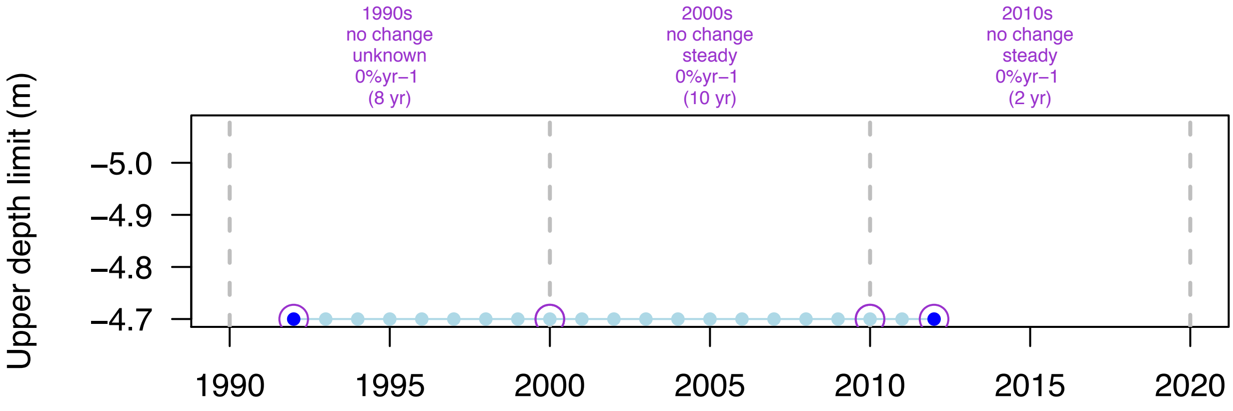

435_cover

Diviacco 2000, Oprandi et al. 2014, Montefalcone (unpublished)

SITE: Spotorno (Italy – Mediterranean) – Po (? m)

OVERALL: Net = –9.5 %;   Rate = –1.22 % yr–1; Perc Final = 86 % > no change

DECADAL: YES (12 yr)

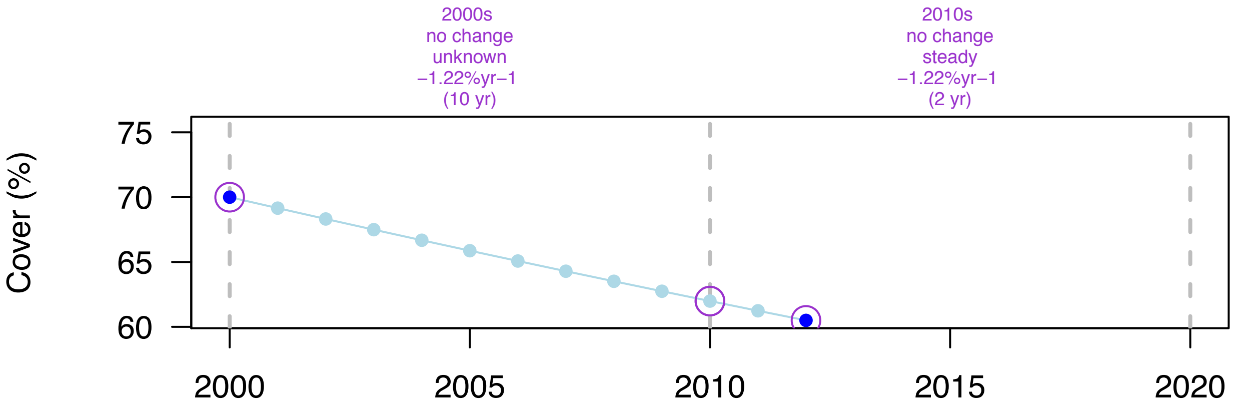

435_upperlimit

Diviacco 2000, Oprandi et al. 2014, Montefalcone (unpublished)

SITE: Spotorno (Italy – Mediterranean) – Po (? m)

OVERALL: Net = 0.2 m;   Rate = 0.67 % yr–1; Perc Final = 108 % > no change

DECADAL: YES (12 yr)

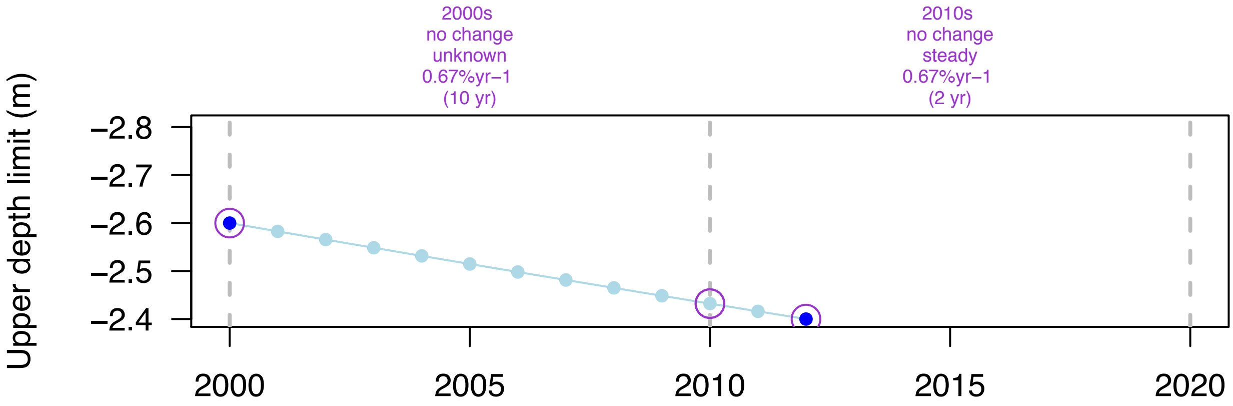

437_cover

Montefalcone et al. 2007b, Montefalcone (unpublished)

SITE: Prelo (Italy – Mediterranean) – Po (? m)

OVERALL: Net = 17.3 %;   Rate = 2.84 % yr–1; Perc Final = 133 % > increase

DECADAL: YES (10 yr)

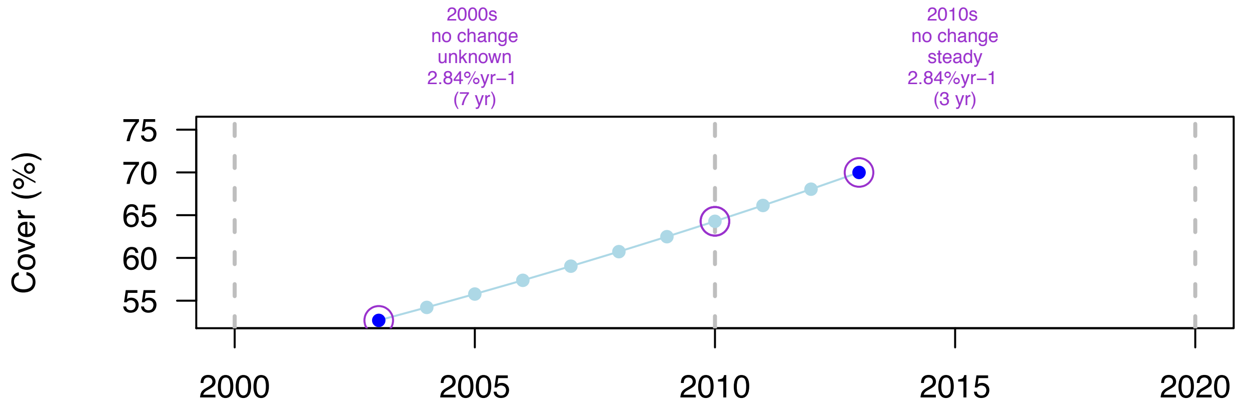

437_density

Montefalcone et al. 2007b, Montefalcone (unpublished)

SITE: Prelo (Italy – Mediterranean) – Po (? m)

OVERALL: Net = –1 shoot m–2;   Rate = –0.02 % yr–1; Perc Final = 100 % > no change

DECADAL: YES (10 yr)

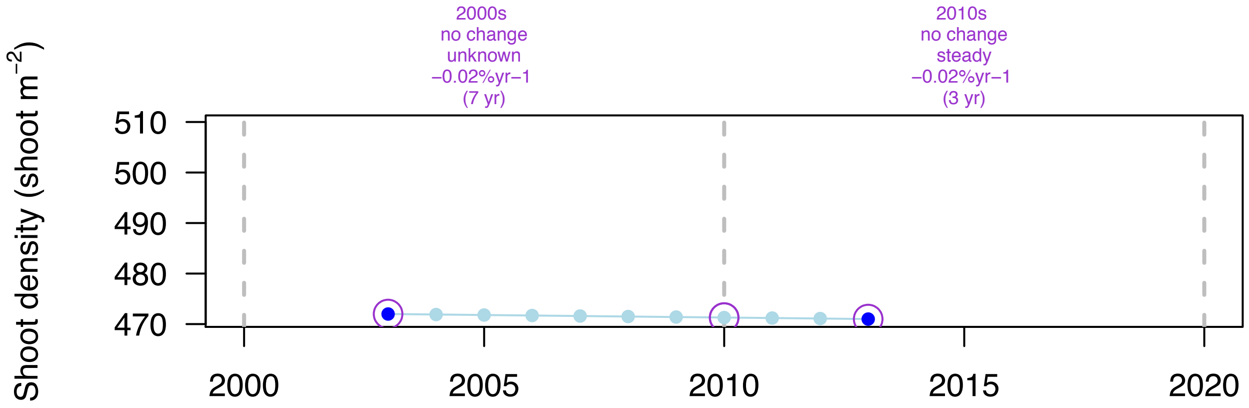

437_lowerlimit

Montefalcone et al. 2007b, Montefalcone (unpublished)

SITE: Prelo (Italy – Mediterranean) – Po (? m)

OVERALL: Net = –3.7 m;   Rate = –3.44 % yr–1; Perc Final = 71 % > decrease

DECADAL: YES (10 yr)

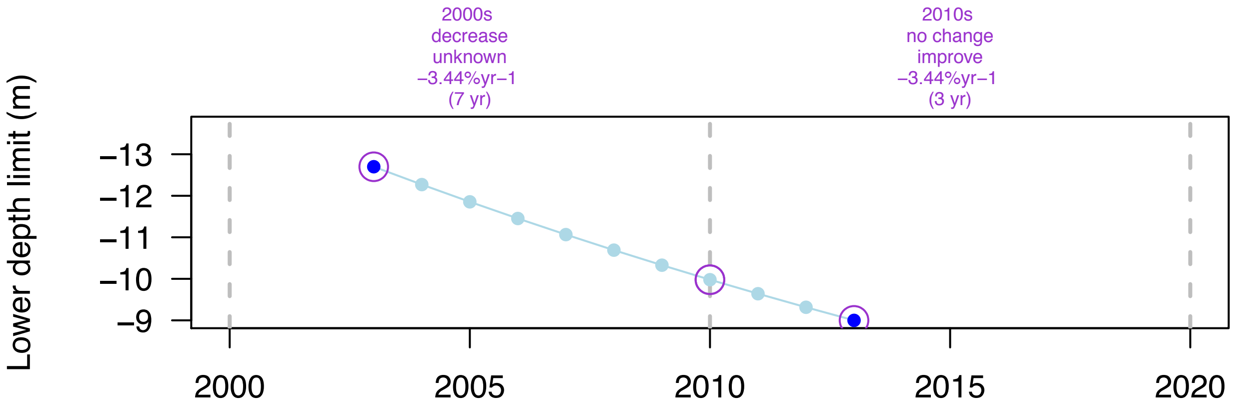

437_upperlimit

Montefalcone et al. 2007b, Montefalcone (unpublished)

SITE: Prelo (Italy – Mediterranean) – Po (? m)

OVERALL: Net = –0.2 m;   Rate = –1.25 % yr–1; Perc Final = 88 % > decrease

DECADAL: YES (10 yr)

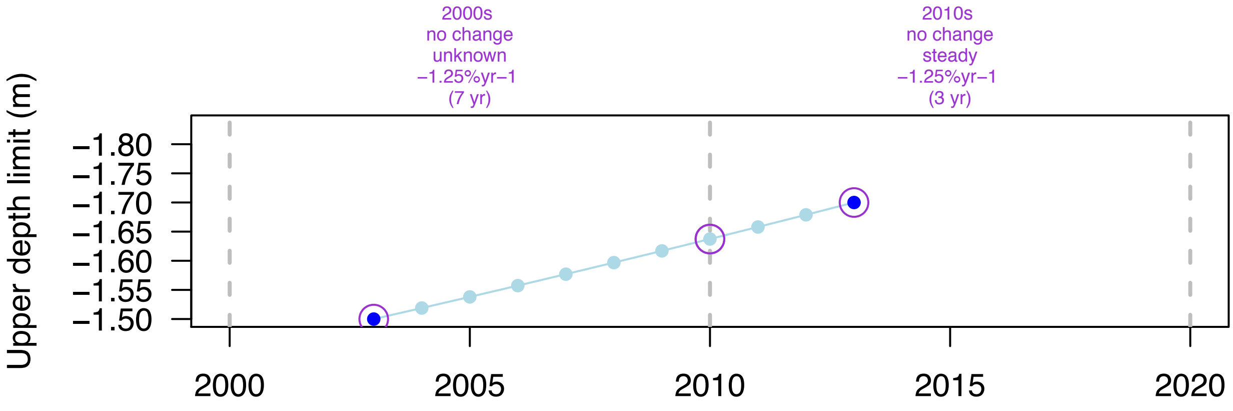

441_area

Fournier 2002

SITE: Baie de Locquirec (France – Atlantic) – Zm (–4 m)

OVERALL: Net = –1.7 ha;   Rate = –2.99 % yr–1; Perc Final = 76 % > decrease

DECADAL: YES (9 yr)

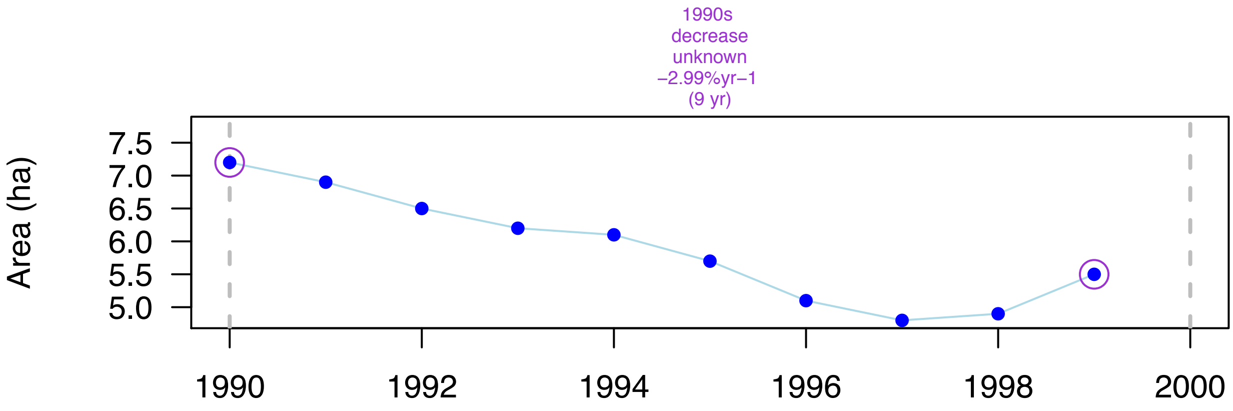

442_area

Godet et al. 2008, Auby et al. 2010

SITE: Chausey Archipelago (intertidal) (France – Atlantic) – Zm (? m)

OVERALL: Net = –165.5 ha;   Rate = –1.18 % yr–1; Perc Final = 40 % > decrease

DECADAL: YES (78 yr)

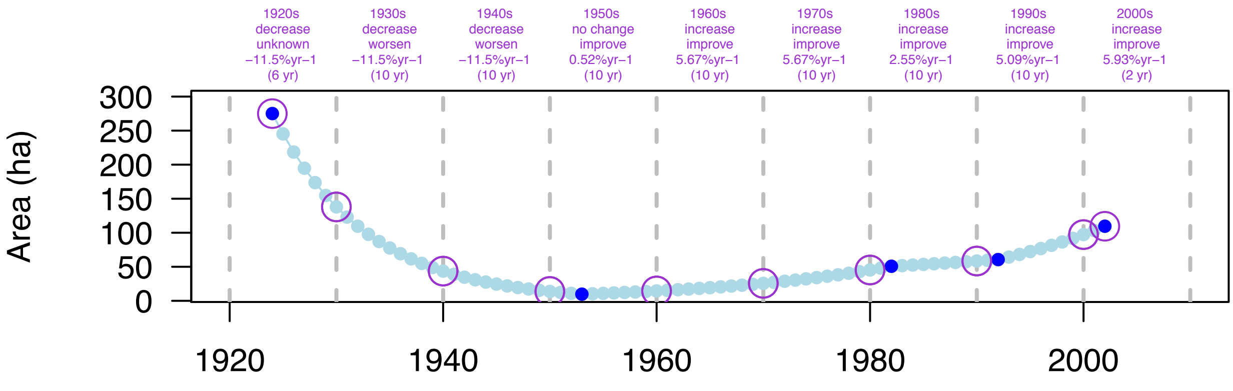

443_area

Godet et al. 2008, Auby et al. 2010

SITE: Chausey Archipelago (subtidal) (France – Atlantic) – Zm (–4 m)

OVERALL: Net = –256.4 ha; Rate = –0.95 % yr–1; Perc Final = 48 % > decrease

DECADAL: YES (78 yr)

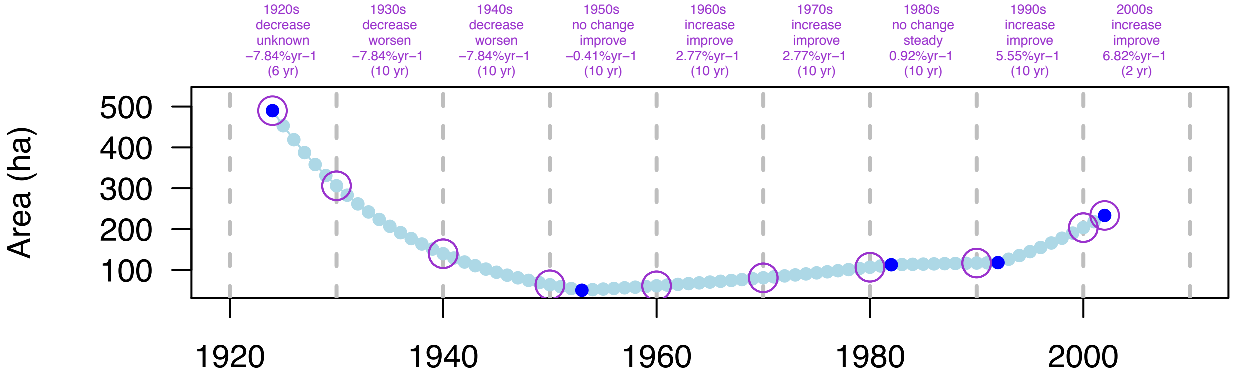

444_area

Barillé et al. 2010

SITE: Bourgneuf Bay (France – Atlantic) – Zn (? m)

OVERALL: Net = 378 ha; Rate = 7.4 % yr–1; Perc Final = 282 % > increase

DECADAL: YES (14 yr)

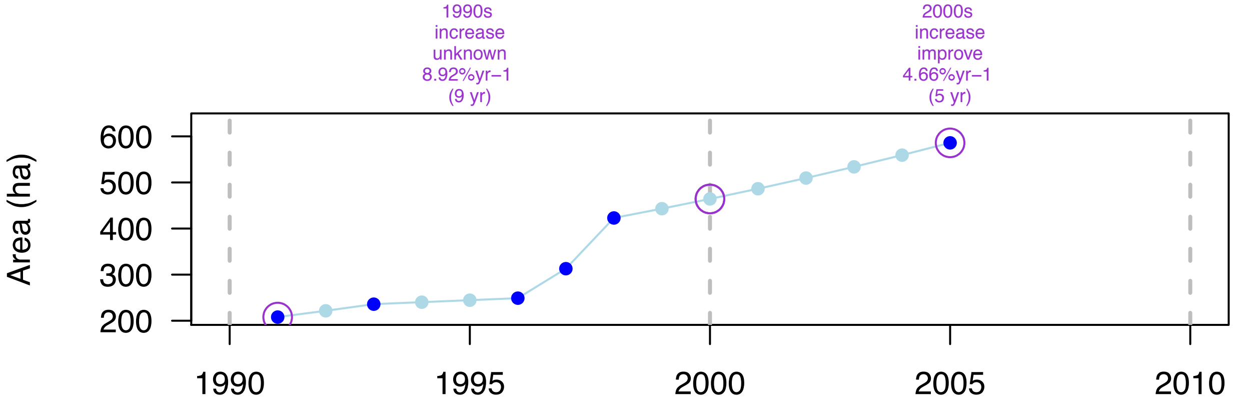

446_area

Fournier 2003, Nebout et al. 2008

SITE: La Canue (France – Atlantic) – Zn (? m)

OVERALL: Net = –1.15 ha; Rate = –19.9 % yr–1; Perc Final = 30 % > decrease

DECADAL: NO (6 yr)

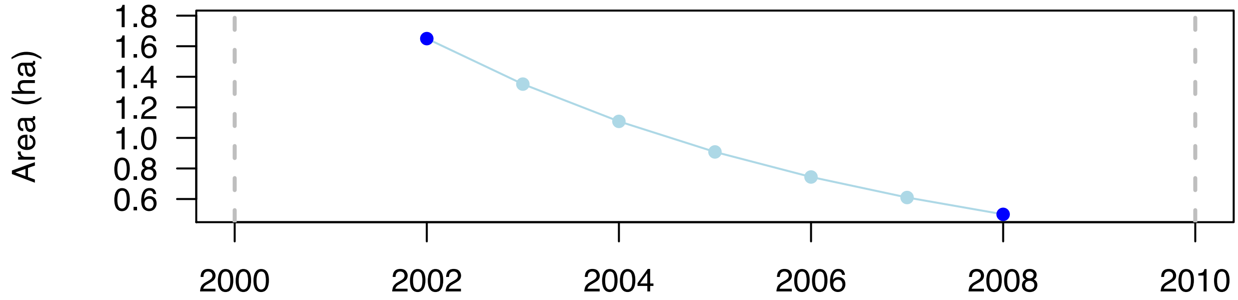

448_abiomass

Auby et al. 2010

SITE: Arcachon Bay (France – Atlantic) – Zn (0 m)

OVERALL: Net = –27.6 g dw m–2; Rate = –1.08 % yr–1; Perc Final = 77 % > no change

DECADAL: YES (24 yr)

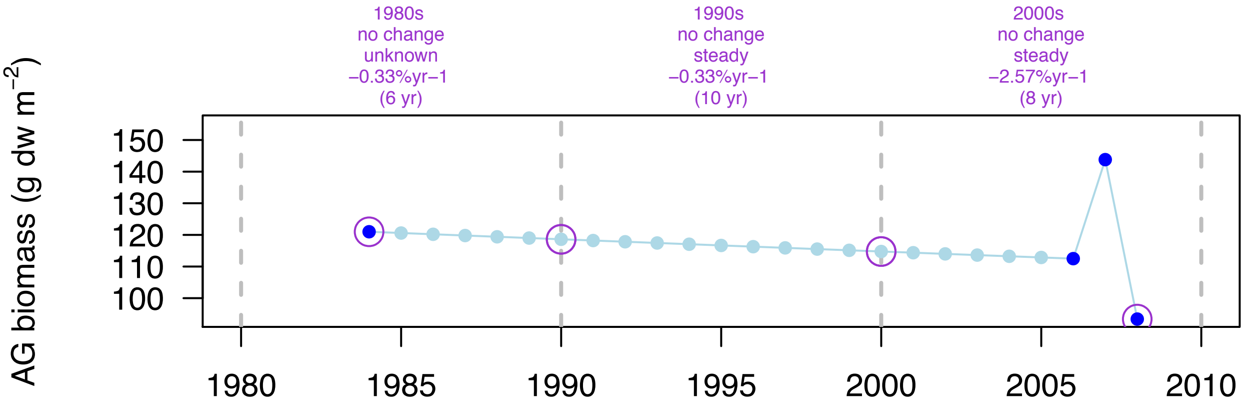

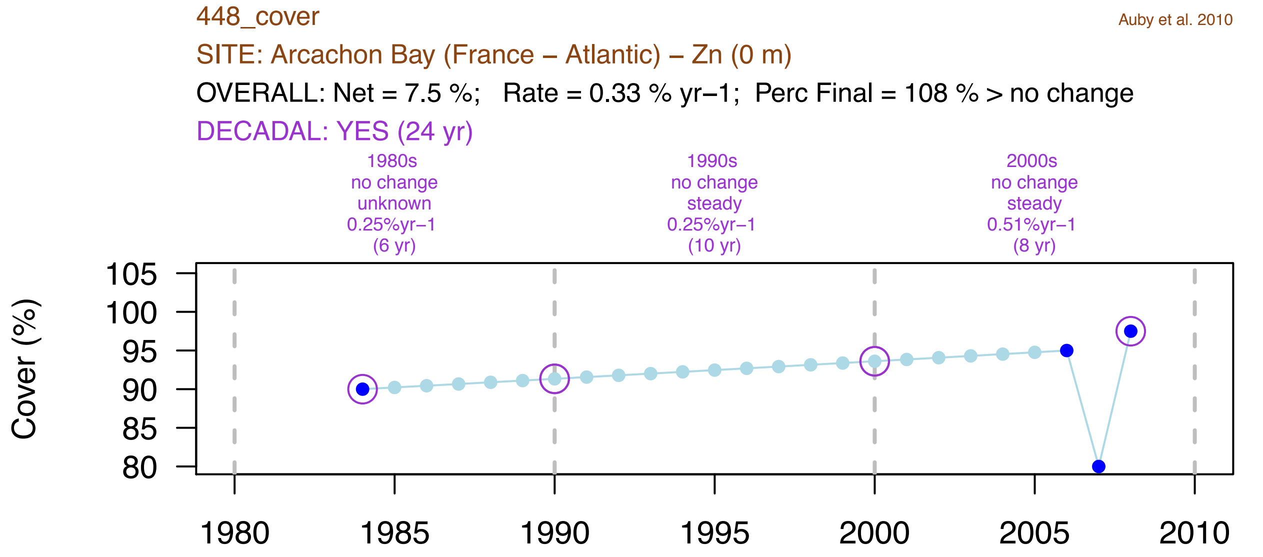
448_cover
Auby et al. 2010
SITE: Arcachon Bay (France – Atlantic) – Zn (0 m)
OVERALL: Net = 7.5 %; Rate = 0.33 % yr–1; Perc Final = 108 % > no change
DECADAL: YES (24 yr)
1980s
no change
unknown
0.25%yr–1
(6 yr)
1990s
no change
steady
0.25%yr–1
(10 yr)
2000s
no change
steady
0.51%yr–1
(8 yr)
Cover (%)
105
100
95
90
85
80
1980
1985
1990
1995
2000
2005
2010

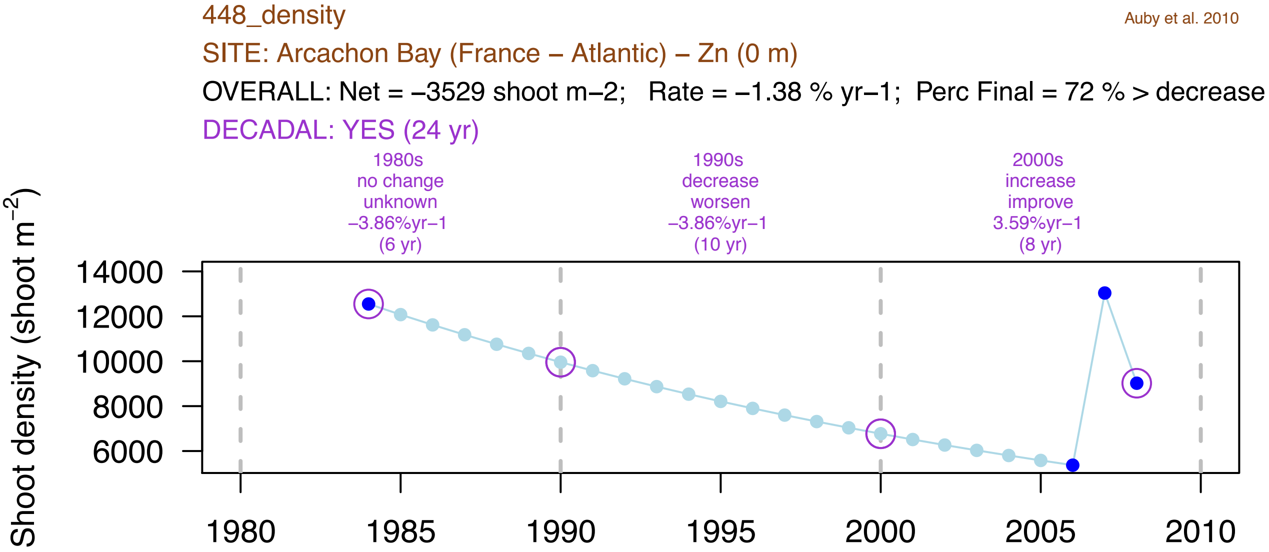
448_density
Auby et al. 2010
SITE: Arcachon Bay (France – Atlantic) – Zn (0 m)
OVERALL: Net = –3529 shoot m–2; Rate = –1.38 % yr–1; Perc Final = 72 % > decrease
DECADAL: YES (24 yr)
1980s
no change
unknown
–3.86%yr–1
(6 yr)
1990s
decrease
worsen
–3.86%yr–1
(10 yr)
2000s
increase
improve
3.59%yr–1
(8 yr)
Shoot density (shoot m^{-2})
14000
12000
10000
8000
6000
1980
1985
1990
1995
2000
2005
2010

449_abiomass

Plus et al. 2010

SITE: Arcachon Bay (France – Atlantic) – Zm (–5 m)

OVERALL: Net = 60.3 g dw m–2;   Rate = 37.2 % yr–1; Perc Final = 210 % > increase

DECADAL: NO (2 yr)

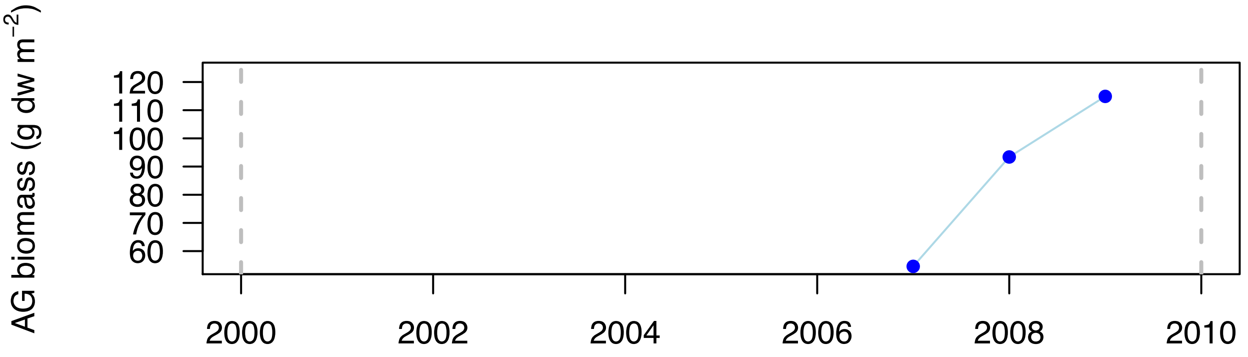

449_density

Plus et al. 2010

SITE: Arcachon Bay (France – Atlantic) – Zm (–5 m)

OVERALL: Net = 48 shoot m–2;   Rate = 18.39 % yr–1; Perc Final = 144 % > increase

DECADAL: NO (2 yr)

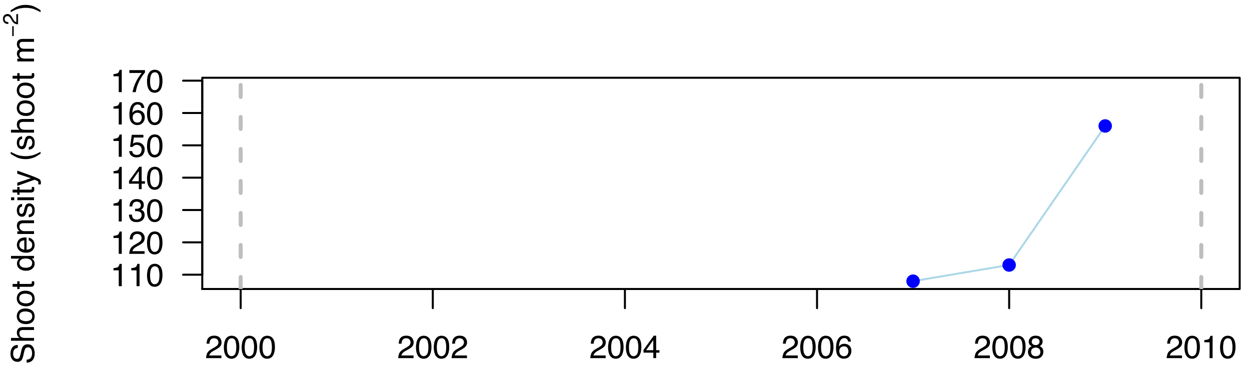

454_abiomass

Auby et al. 2010

SITE: Callot (Baie de Morlaix) (France – Atlantic) – Zm (? m)

OVERALL: Net = −56.6 g dw m−2;   Rate = −11.93 % yr−1; Perc Final = 55 % > decrease

DECADAL: NO (5 yr)

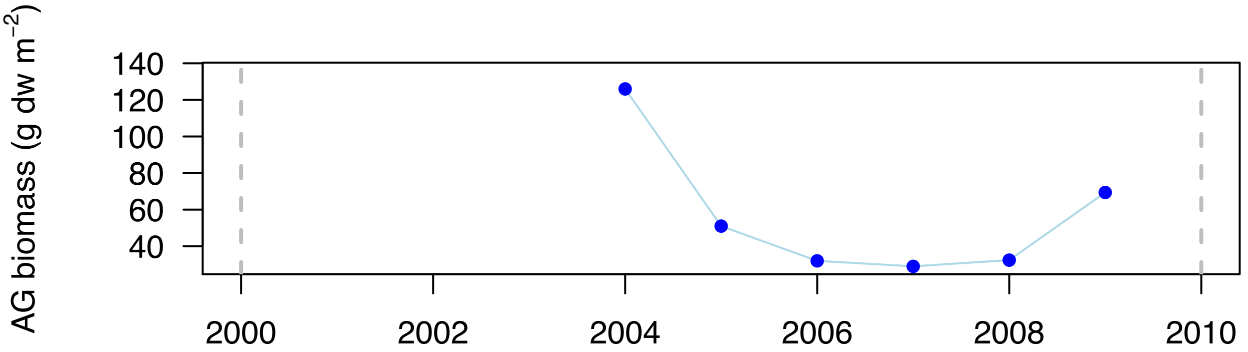

454_density

Auby et al. 2010

SITE: Callot (Baie de Morlaix) (France – Atlantic) – Zm (? m)

OVERALL: Net = 1 shoot m−2;   Rate = 0.07 % yr−1; Perc Final = 100 % > no change

DECADAL: NO (5 yr)

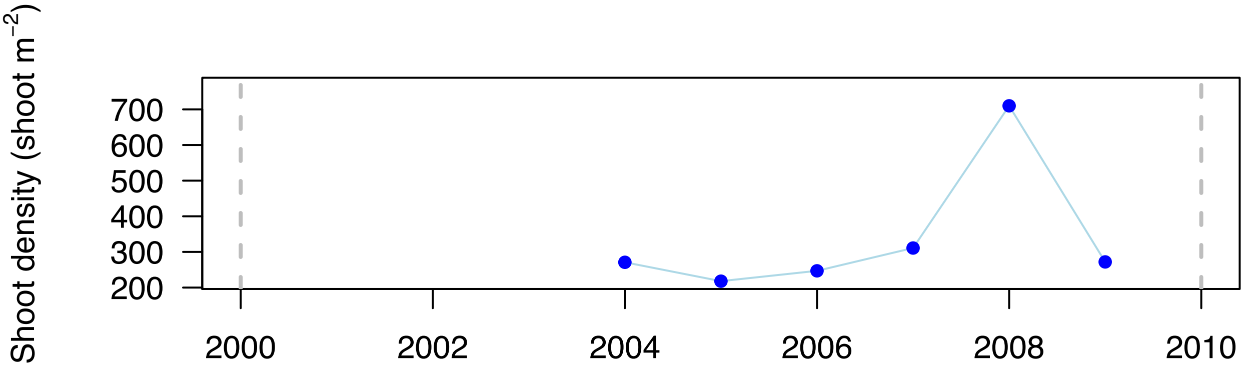

469_abiomass

Auby et al. 2010

SITE: Concarneau (Glenan Archipelago) (France – Atlantic) – Zm (? m)

OVERALL: Net = 37.2 g dw m–2;   Rate = 23.29 % yr–1; Perc Final = 159 % > increase

DECADAL: NO (2 yr)

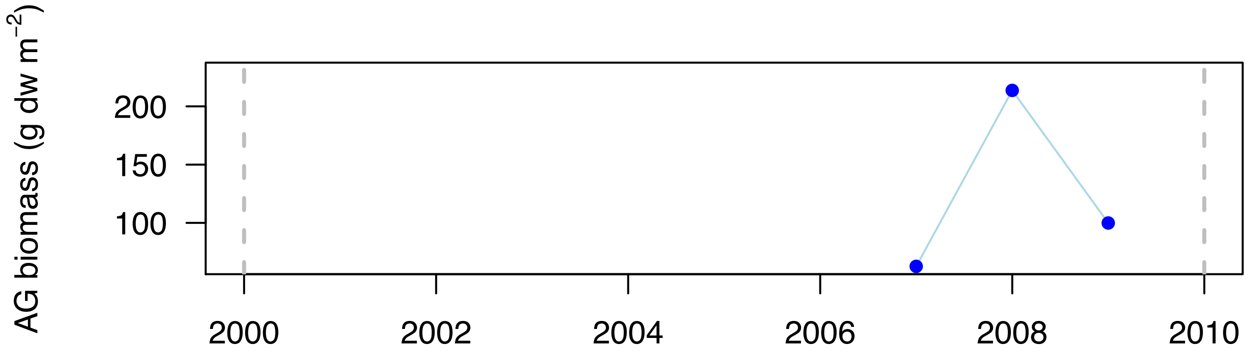

469_density

Auby et al. 2010

SITE: Concarneau (Glenan Archipelago) (France – Atlantic) – Zm (? m)

OVERALL: Net = –128 shoot m–2;   Rate = –11.6 % yr–1; Perc Final = 79 % > no change

DECADAL: NO (2 yr)

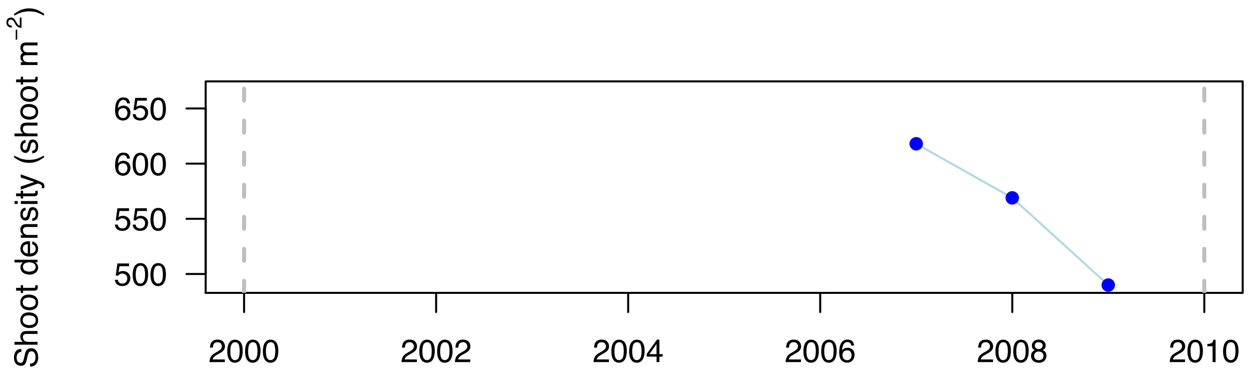

473_area

Nebout et al. 2008, Auby et al. 2010

SITE: Plage de L'Ecluse (France – Atlantic) – Zm (? m)

OVERALL: Net = 10.2 ha;   Rate = 4.17 % yr–1; Perc Final = 803 % > increase

DECADAL: YES (50 yr)

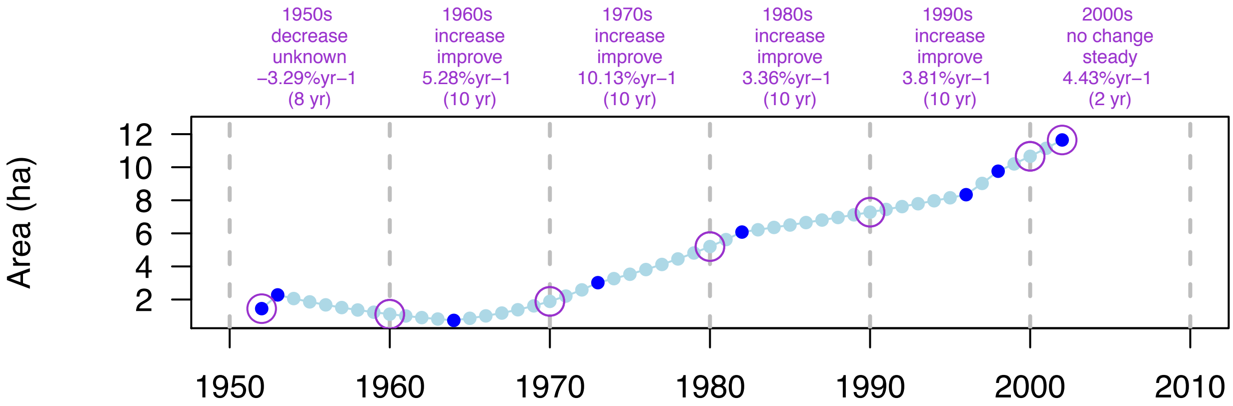

473_cover

Nebout et al. 2008, Auby et al. 2010

SITE: Plage de L'Ecluse (France – Atlantic) – Zm (? m)

OVERALL: Net = 0 %;   Rate = 0 % yr–1; Perc Final = 100 % > no change

DECADAL: YES (50 yr)

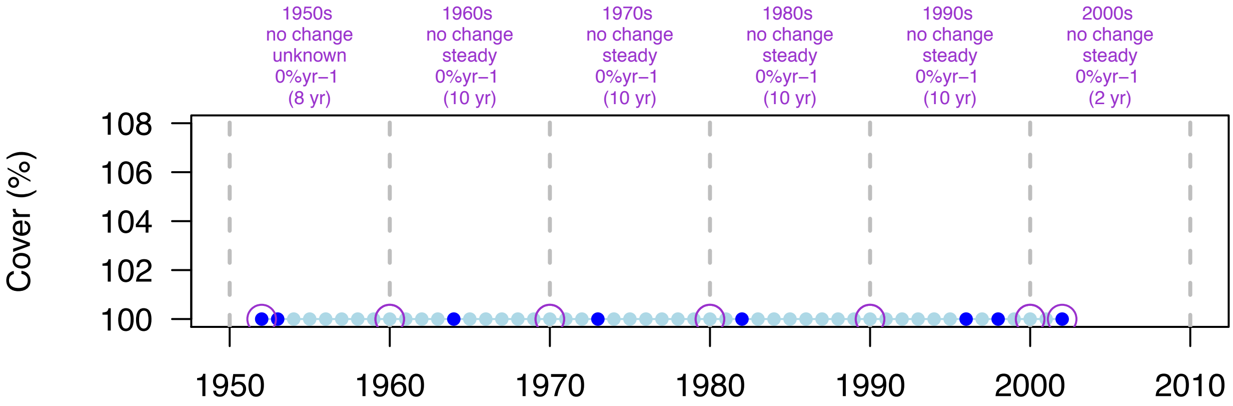

474_area

Nebout et al. 2008, Auby et al. 2010

SITE: Baie du Prieuré (France – Atlantic) – Zm (? m)

OVERALL: Net = 0.13 ha;   Rate = 0.03 % yr–1; Perc Final = 101 % > no change

DECADAL: YES (50 yr)

1950s	1960s	1970s	1980s	1990s	2000s
increase	no change	decrease	no change	increase	increase
unknown	steady	worsen	improve	improve	improve
8.01%yr–1	–0.48%yr–1	–11.31%yr–1	–0.56%yr–1	4.75%yr–1	6.67%yr–1
(8 yr)	(10 yr)	(10 yr)	(10 yr)	(10 yr)	(2 yr)

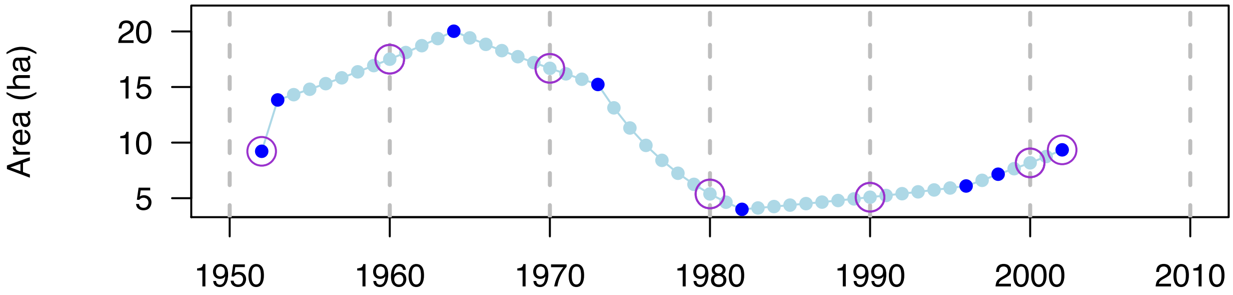

474_cover

Nebout et al. 2008, Auby et al. 2010

SITE: Baie du Prieuré (France – Atlantic) – Zm (? m)

OVERALL: Net = 0 %;   Rate = 0 % yr–1; Perc Final = 100 % > no change

DECADAL: YES (50 yr)

1950s	1960s	1970s	1980s	1990s	2000s
no change	no change	no change	no change	no change	no change
unknown	steady	steady	steady	steady	steady
0%yr–1	0%yr–1	0%yr–1	0%yr–1	0%yr–1	0%yr–1
(8 yr)	(10 yr)	(10 yr)	(10 yr)	(10 yr)	(2 yr)

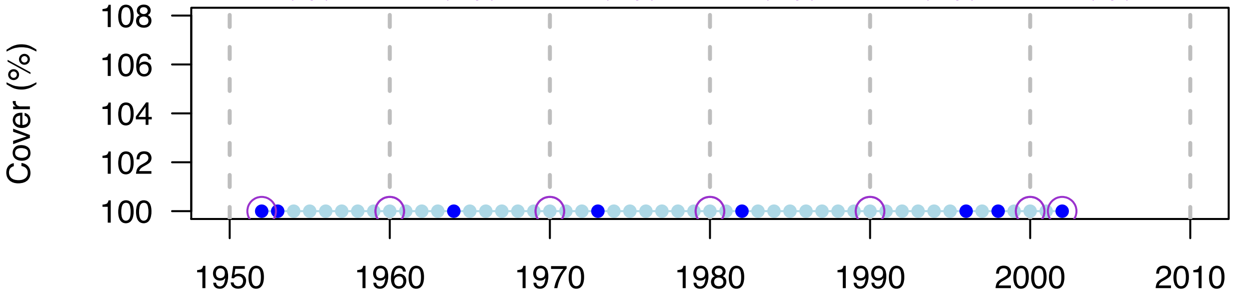

475_abiomass

Auby et al. 2010

SITE: Estuaire Bidassoa (France – Atlantic) – Zn (? m)

OVERALL: Net = 15.42 g dw m–2;   Rate = 19.94 % yr–1; Perc Final = 149 % > increase

DECADAL: NO (2 yr)

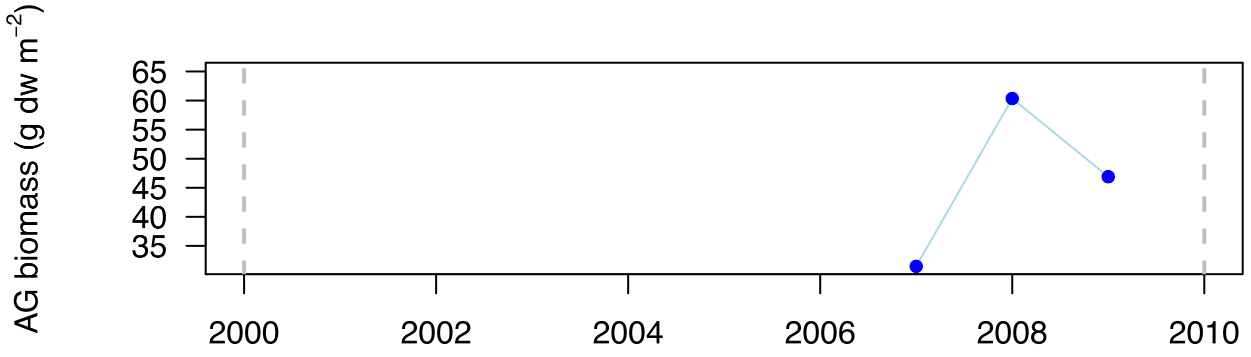

475_cover

Auby et al. 2010

SITE: Estuaire Bidassoa (France – Atlantic) – Zn (? m)

OVERALL: Net = 0 %;   Rate = 0 % yr–1; Perc Final = 100 % > no change

DECADAL: NO (2 yr)

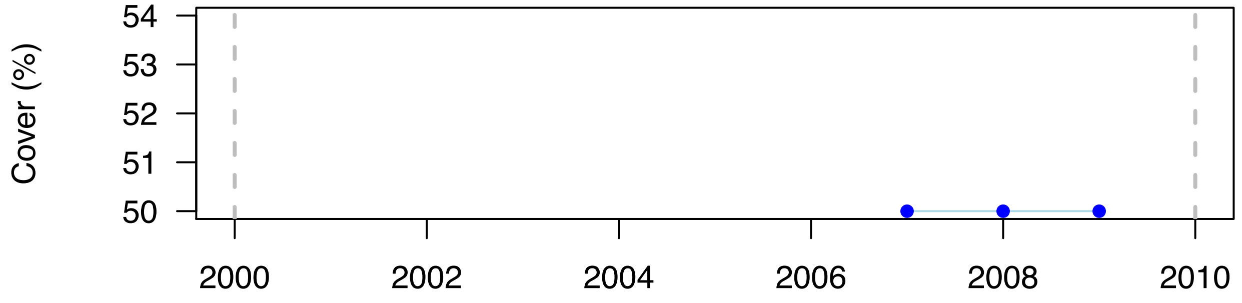

475_density

Auby et al. 2010

SITE: Estuaire Bidassoa (France – Atlantic) – Zn (? m)

OVERALL: Net = 2375 shoot m–2;   Rate = 23.14 % yr–1; Perc Final = 159 % > increase

DECADAL: NO (2 yr)

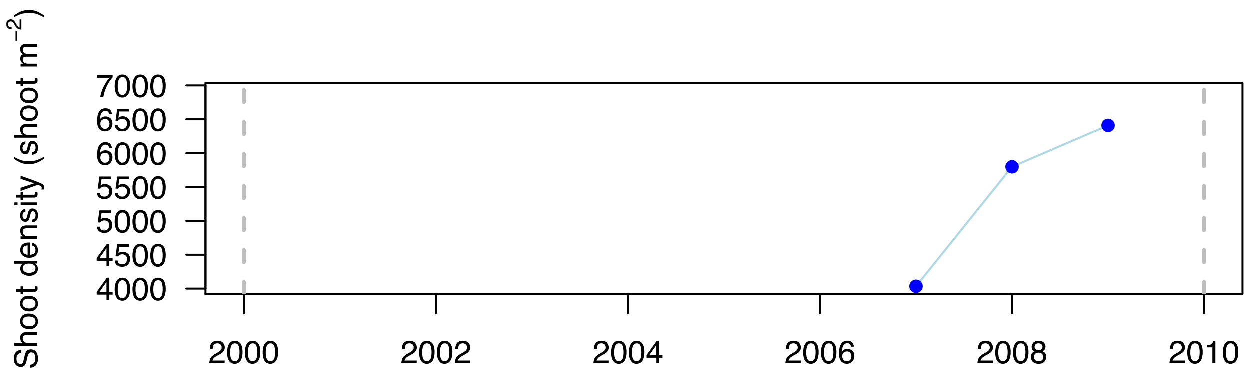

476_area

Auby et al. 2010

SITE: Estuaire Bidassoa (France – Atlantic) – Zn (? m)

OVERALL: Net = –7.04 ha;   Rate = –5.34 % yr–1; Perc Final = 19 % > decrease

DECADAL: YES (31 yr)

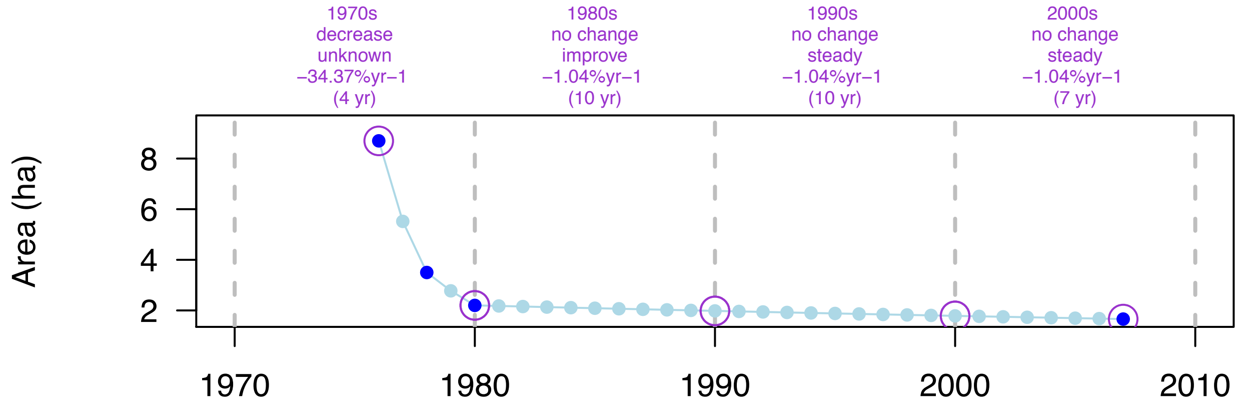

480_area

Auby et al. 2010

SITE: Golfe du Morbihan (France – Atlantic) – Zn (0 m)

OVERALL: Net = –517 ha; Rate = –1.15 % yr–1; Perc Final = 58 % > decrease

DECADAL: YES (47 yr)

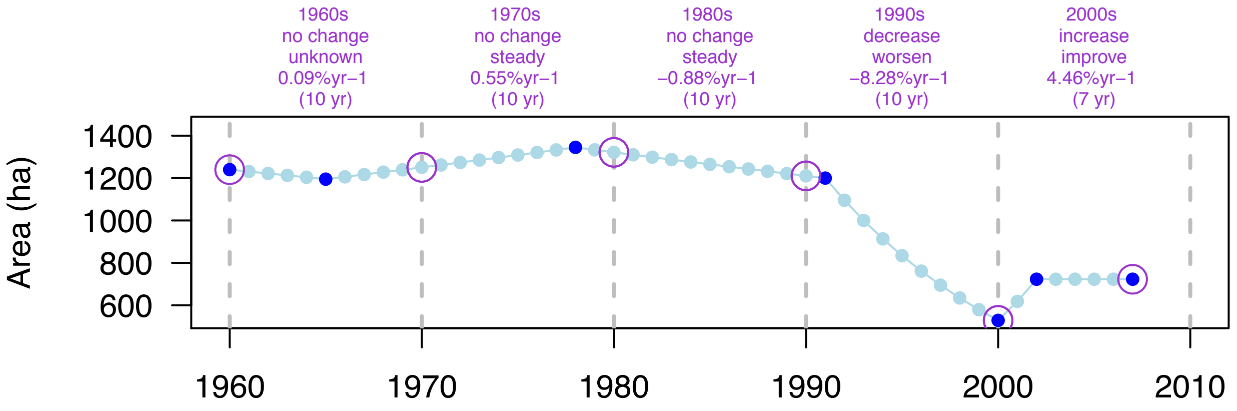

481_area

Auby et al. 2010

SITE: Golfe du Morbihan (France – Atlantic) – Zm (0 m)

OVERALL: Net = –2422 ha; Rate = –1.64 % yr–1; Perc Final = 31 % > decrease

DECADAL: YES (72 yr)

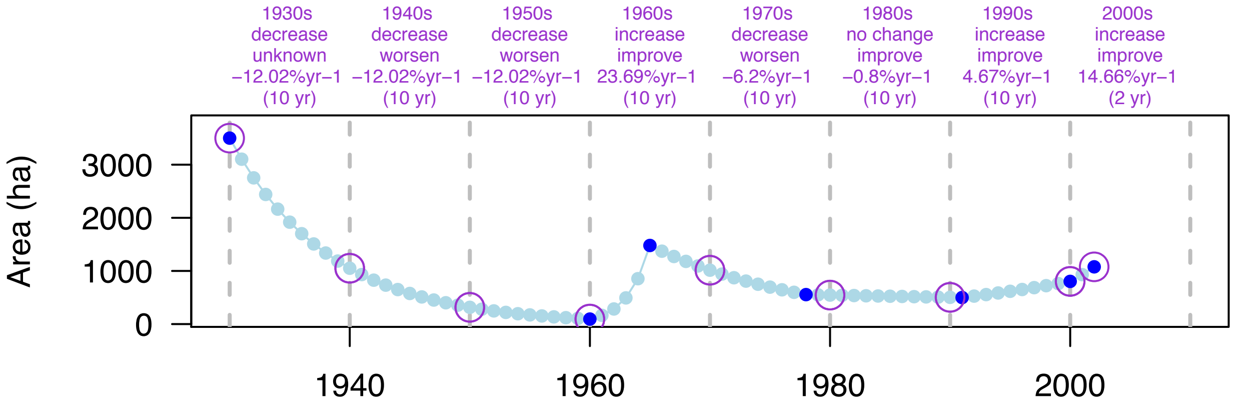

482_area

Auby et al. 2010

SITE: Ouest Cotentin (France – Atlantic) – Zm (? m)

OVERALL: Net = –728 ha; Rate = –6.94 % yr–1; Perc Final = 18 % > decrease

DECADAL: YES (25 yr)

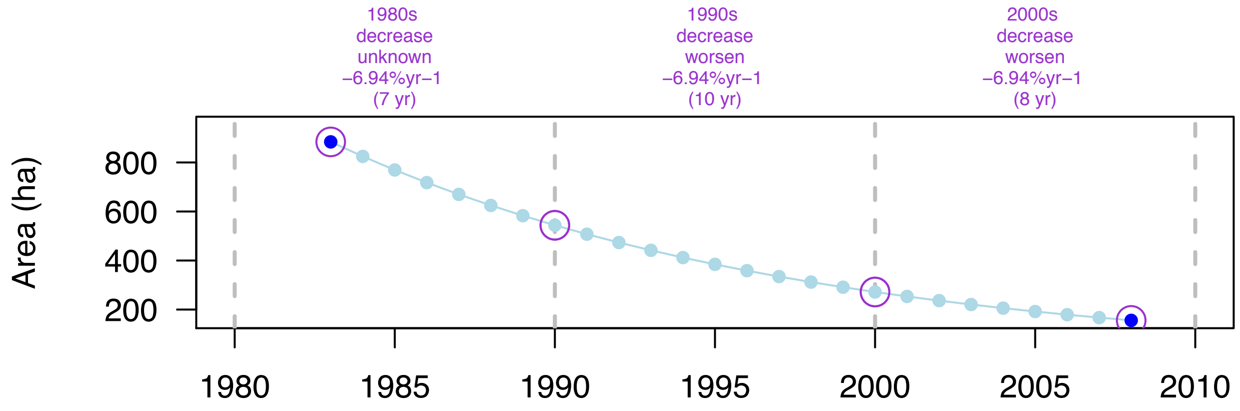

487_abiomass

Auby et al. 2010

SITE: Molène (France – Atlantic) – Zm (? m)

OVERALL: Net = –83.2 g dw m–2; Rate = –12.41 % yr–1; Perc Final = 54 % > decrease

DECADAL: NO (5 yr)

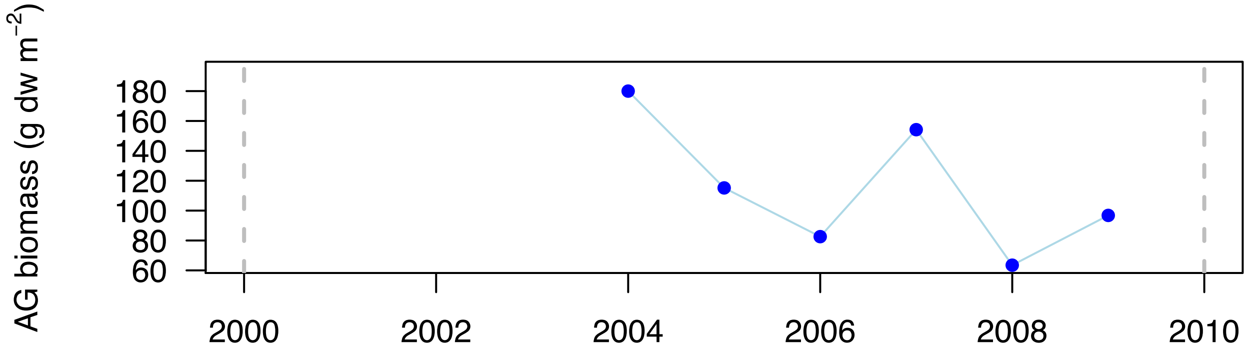

487_density

Auby et al. 2010

SITE: Molène (France – Atlantic) – Zm (? m)

OVERALL: Net = –48 shoot m–2;   Rate = –5.05 % yr–1; Perc Final = 78 % > no change

DECADAL: NO (5 yr)

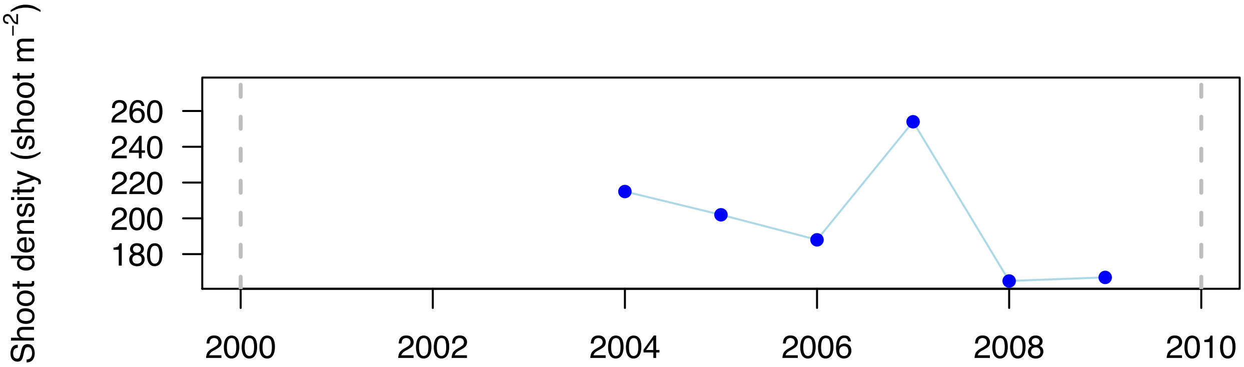

496_area

Auby et al. 2010

SITE: Lac d'Hossegor (France – Atlantic) – Zn (? m)

OVERALL: Net = 0.46 ha;   Rate = NA % yr–1; Perc Final = NA % > increase

DECADAL: YES (41 yr)

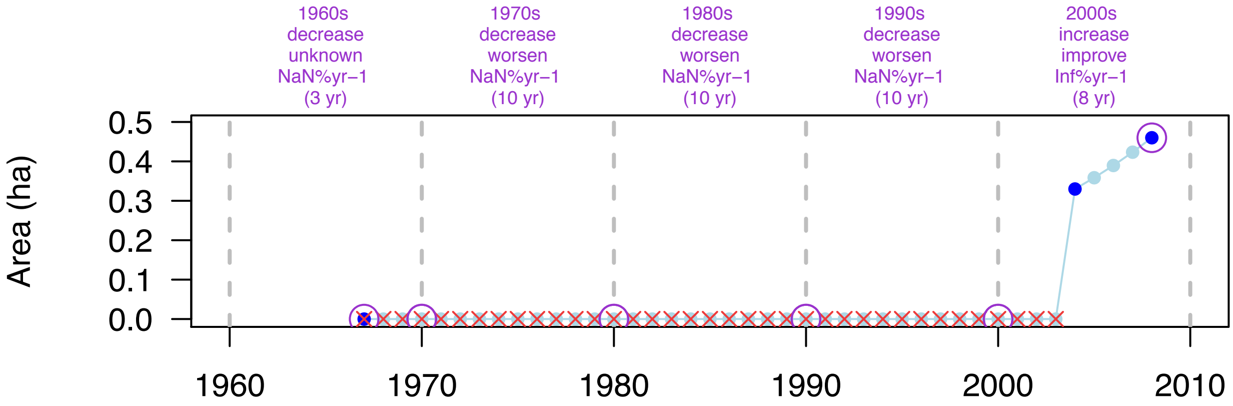

497_area

Auby et al. 2010

SITE: Lac d'Hossegor (France – Atlantic) – Zm (? m)

OVERALL: Net = 0.47 ha;   Rate = NA % yr–1; Perc Final = NA % > increase

DECADAL: YES (41 yr)

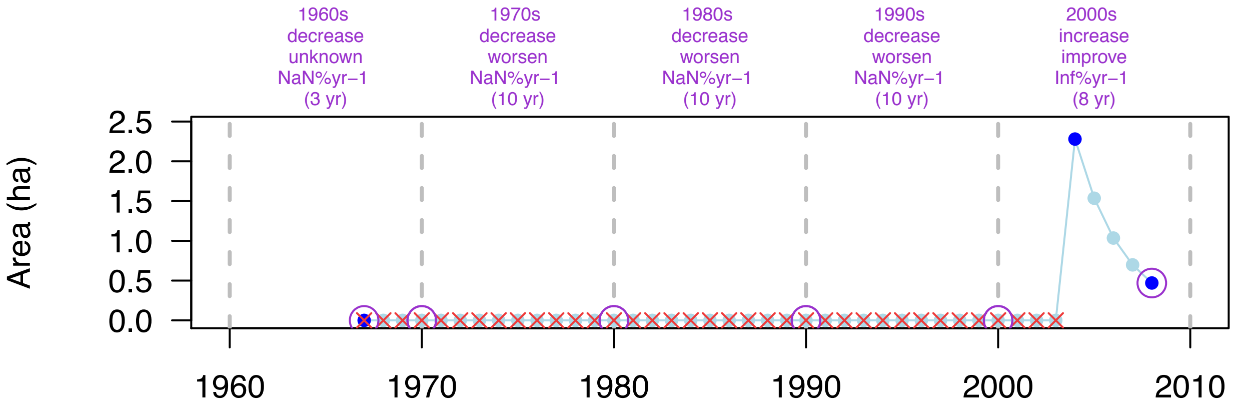

506_area

Auby et al. 2010

SITE: L'estuaire du Lay (France – Atlantic) – Zn (? m)

OVERALL: Net = 17.6 ha;   Rate = NA % yr–1; Perc Final = NA % > increase

DECADAL: NO (4 yr)

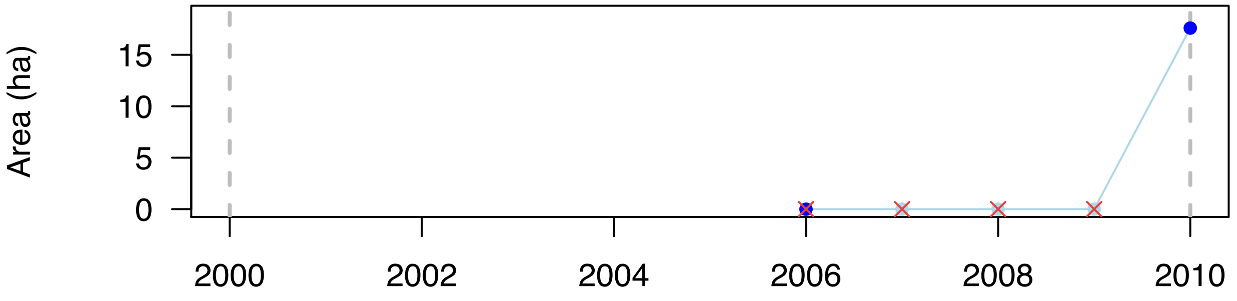

507_abiomass

Auby et al. 2010

SITE: Sainte−Marquerite (France − Atlantic) − Zm (? m)

OVERALL: Net = −19 g dw m−2;   Rate = −4.86 % yr−1; Perc Final = 78 % > no change

DECADAL: NO (5 yr)

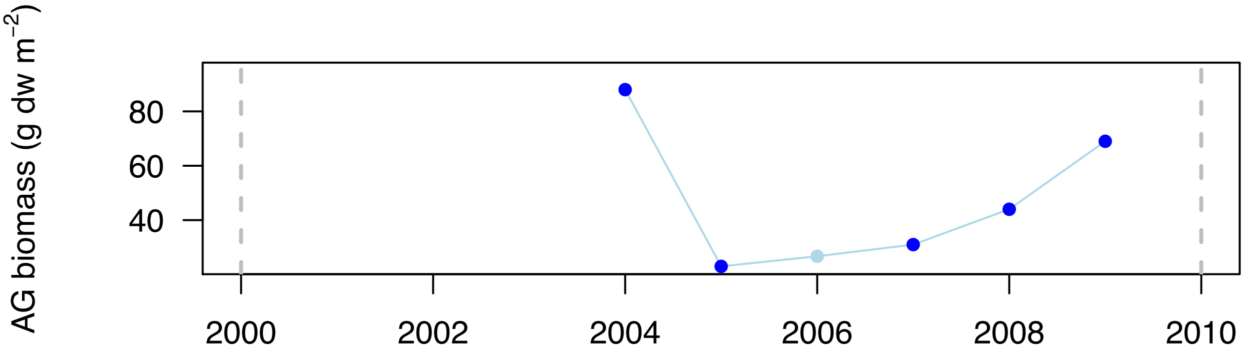

507_density

Auby et al. 2010

SITE: Sainte−Marquerite (France − Atlantic) − Zm (? m)

OVERALL: Net = −288 shoot m−2;   Rate = −19.18 % yr−1; Perc Final = 38 % > decrease

DECADAL: NO (5 yr)

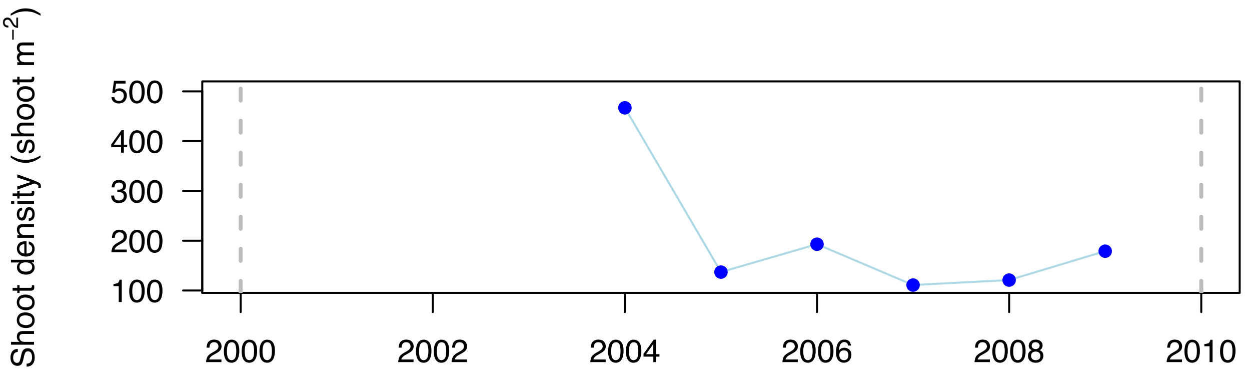

519_abiomass

Auby et al. 2010

SITE: Paimpol (France – Atlantic) – Zm (? m)

OVERALL: Net = 11.2 g dw m–2; Rate = 2.74 % yr–1; Perc Final = 115 % > no change

DECADAL: NO (5 yr)

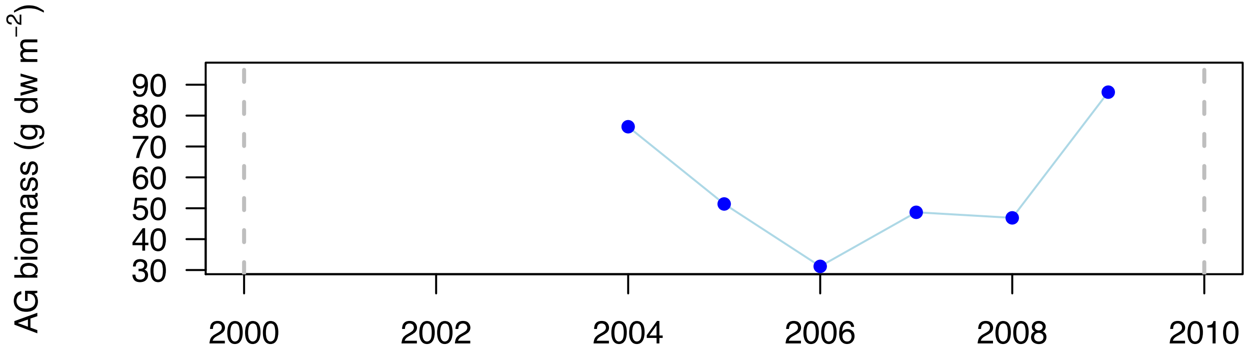

519_density

Auby et al. 2010

SITE: Paimpol (France – Atlantic) – Zm (? m)

OVERALL: Net = –330 shoot m–2; Rate = –17.11 % yr–1; Perc Final = 43 % > decrease

DECADAL: NO (5 yr)

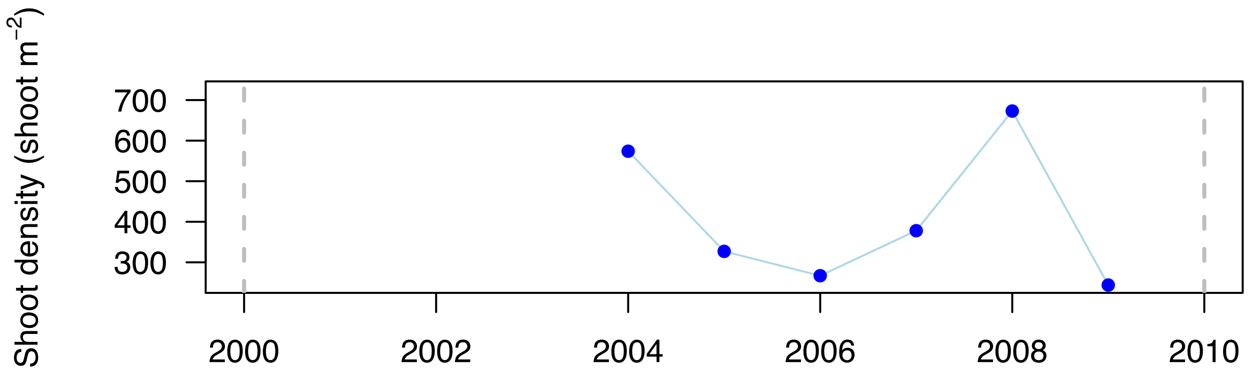

520_abiomass

Auby et al. 2010

SITE: Les Sept Îles (France – Atlantic) – Zm (? m)

OVERALL: Net = –36.9 g dw m–2;   Rate = –15.54 % yr–1; Perc Final = 73 % > decrease

DECADAL: NO (2 yr)

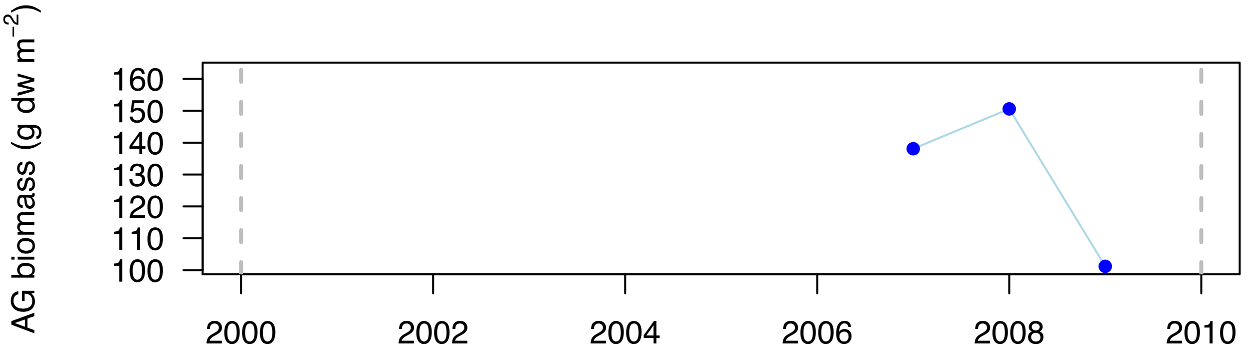

520_density

Auby et al. 2010

SITE: Les Sept Îles (France – Atlantic) – Zm (? m)

OVERALL: Net = –56 shoot m–2;   Rate = –4.03 % yr–1; Perc Final = 92 % > no change

DECADAL: NO (2 yr)

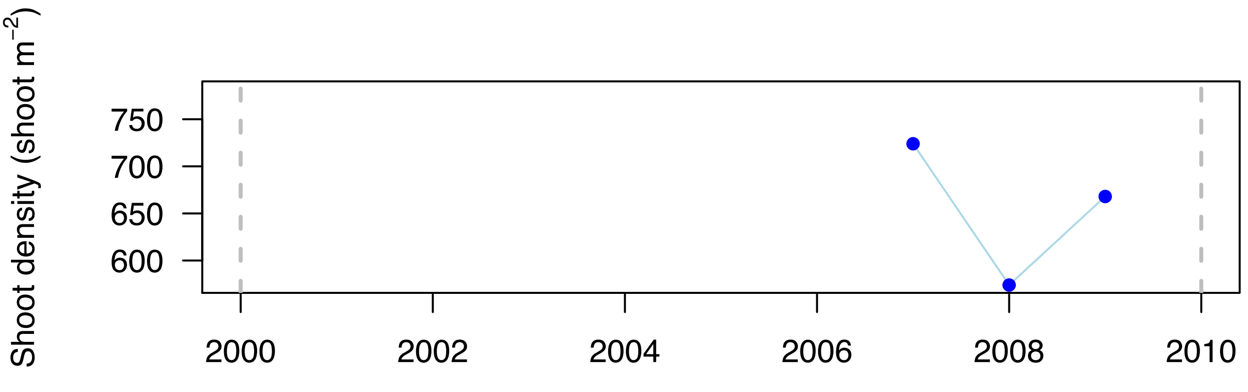

521_area Auby et al. 2010

SITE: Pertuis Breton (France – Atlantic) – Zn (? m)

OVERALL: Net = −504.6 ha; Rate = −2.73 % yr−1; Perc Final = 9 % > decrease

DECADAL: YES (90 yr)

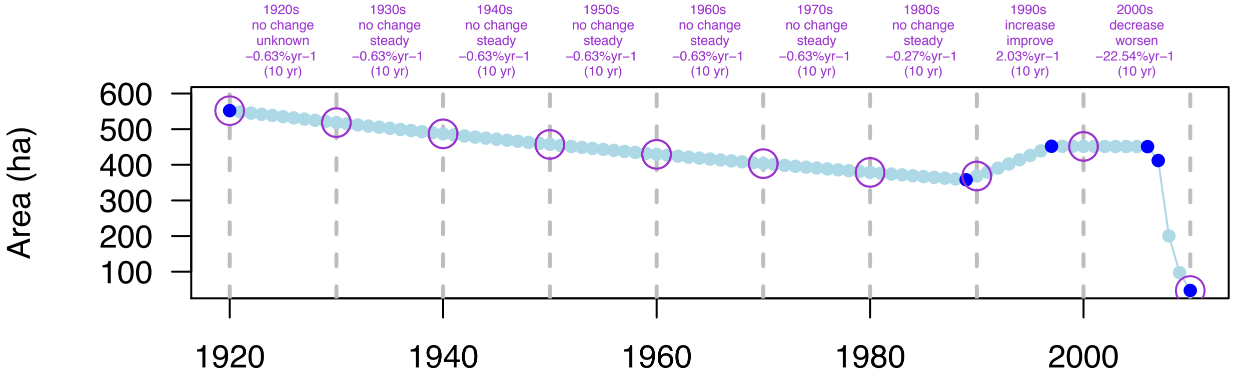

522_area Auby et al. 2010

SITE: Pertuis Breton (France – Atlantic) – Zm (? m)

OVERALL: Net = −591.45 ha; Rate = NA % yr−1; Perc Final = NA % > decrease

DECADAL: YES (74 yr)

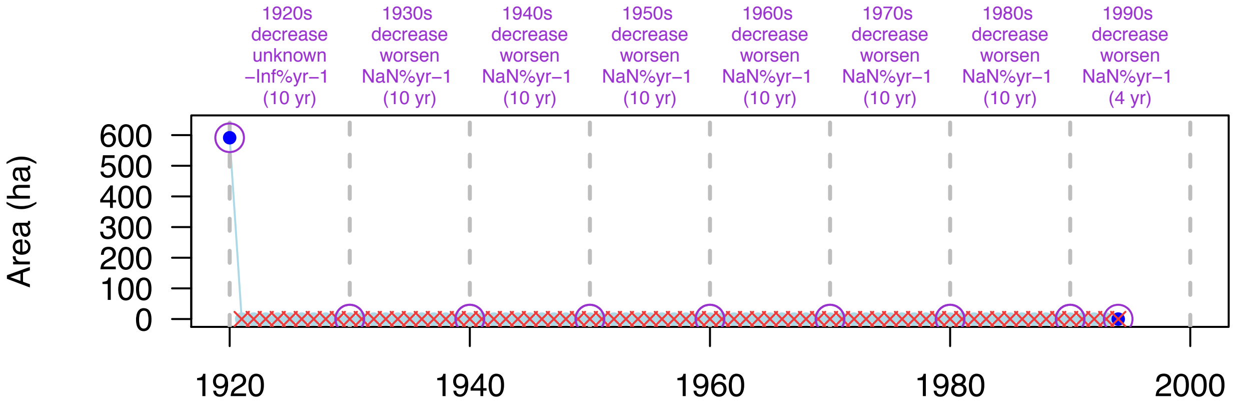

523_abiomass

Auby et al. 2010

SITE: Les Doux (Pertuis Charentais) (France – Atlantic) – Zn (? m)

OVERALL: Net = 17.1 g dw m–2;   Rate = 3.27 % yr–1; Perc Final = 134 % > increase

DECADAL: YES (9 yr)

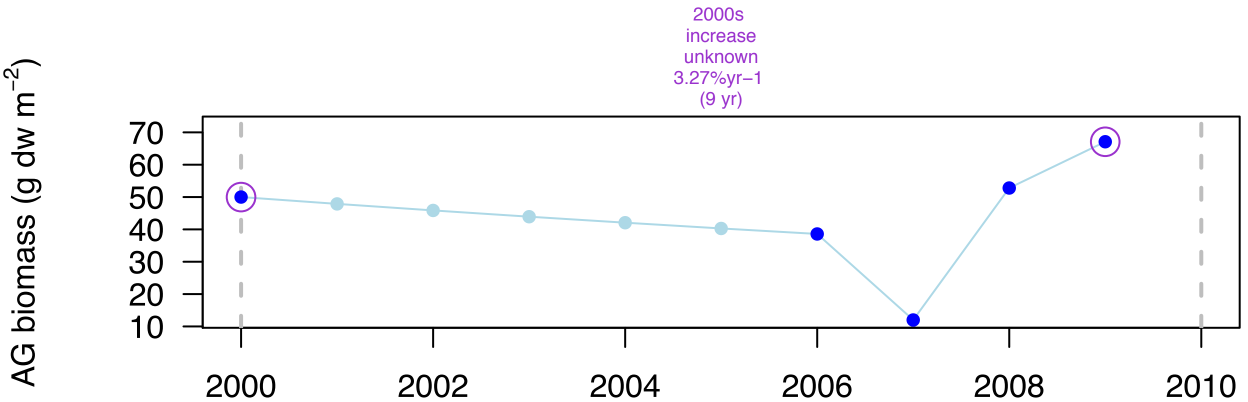

523_density

Auby et al. 2010

SITE: Les Doux (Pertuis Charentais) (France – Atlantic) – Zn (? m)

OVERALL: Net = 2934 shoot m–2;   Rate = 24.48 % yr–1; Perc Final = 163 % > increase

DECADAL: NO (2 yr)

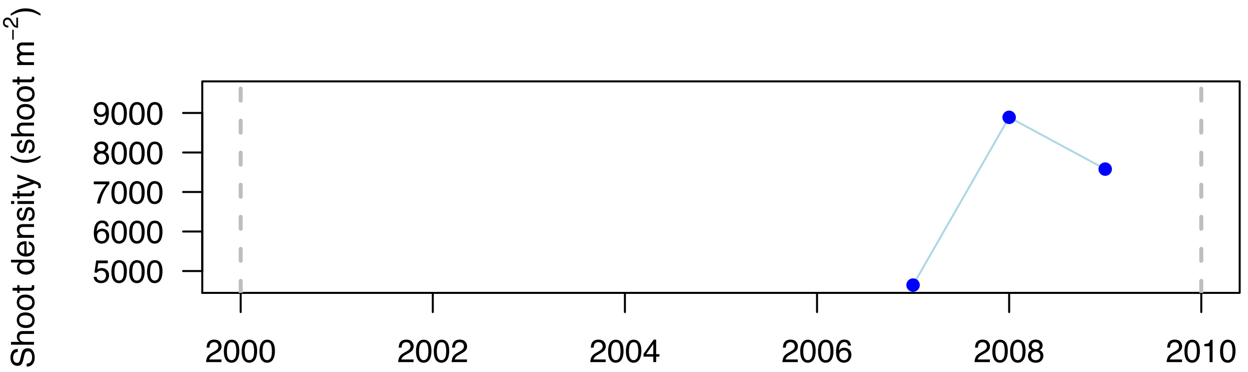

531_area

Auby et al. 2010

SITE: Rade de Brest (France – Atlantic) – Zm (? m)

OVERALL: Net = 60.3 ha; Rate = 20.36 % yr–1; Perc Final = 1730 % > increase

DECADAL: YES (14 yr)

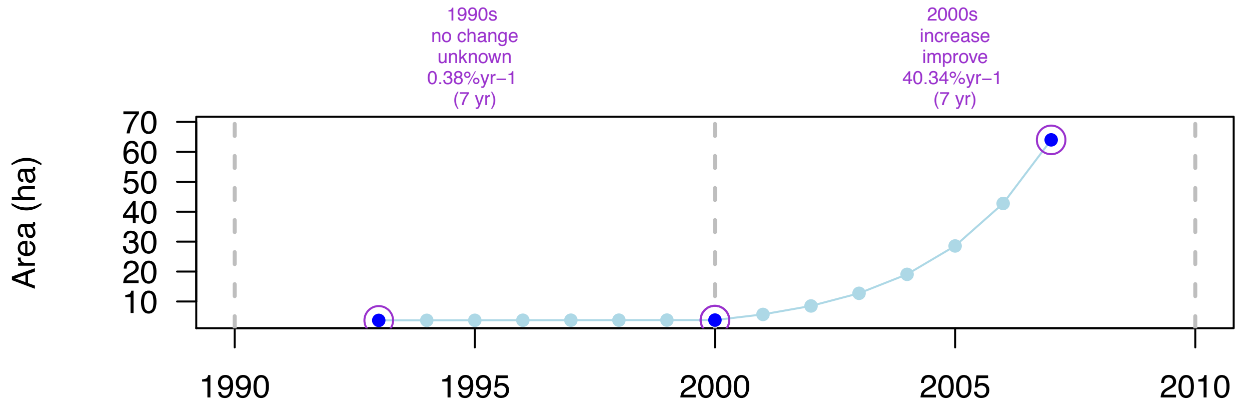

556_density

Marbà and Duarte 2010, Duarte and Marbà (unpublished)

SITE: El Castell (Spain – Mediterranean) – Po (–25 m)

OVERALL: Net = –84.38 shoot m–2; Rate = –6.85 % yr–1; Perc Final = 47 % > decrease

DECADAL: YES (11 yr)

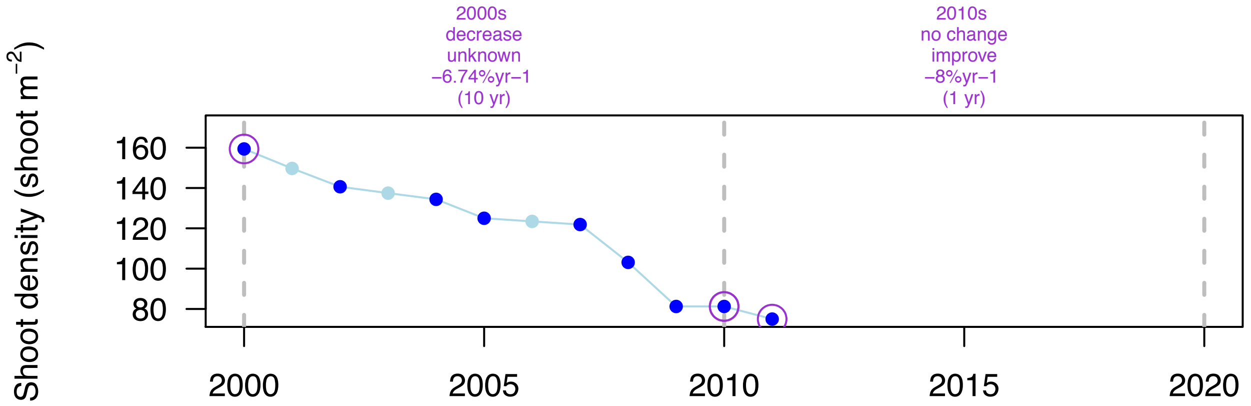

557_density

Marbà and Duarte 2010, Duarte and Marbà (unpublished)

SITE: Cala Santa María (Spain – Mediterranean) – Po (–17 m)

OVERALL: Net = –181.33 shoot m–2;   Rate = –4.78 % yr–1; Perc Final = 62 % > decrease

DECADAL: YES (10 yr)

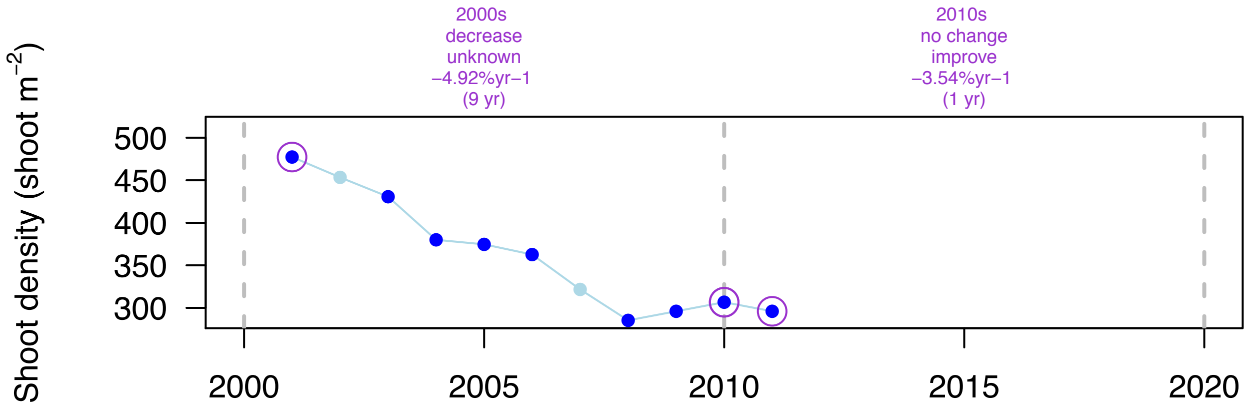

558_density

Marbà and Duarte 2010, Duarte and Marbà (unpublished)

SITE: Cala Santa María (Spain – Mediterranean) – Po (–7 m)

OVERALL: Net = –337.04 shoot m–2;   Rate = –3.74 % yr–1; Perc Final = 66 % > decrease

DECADAL: YES (11 yr)

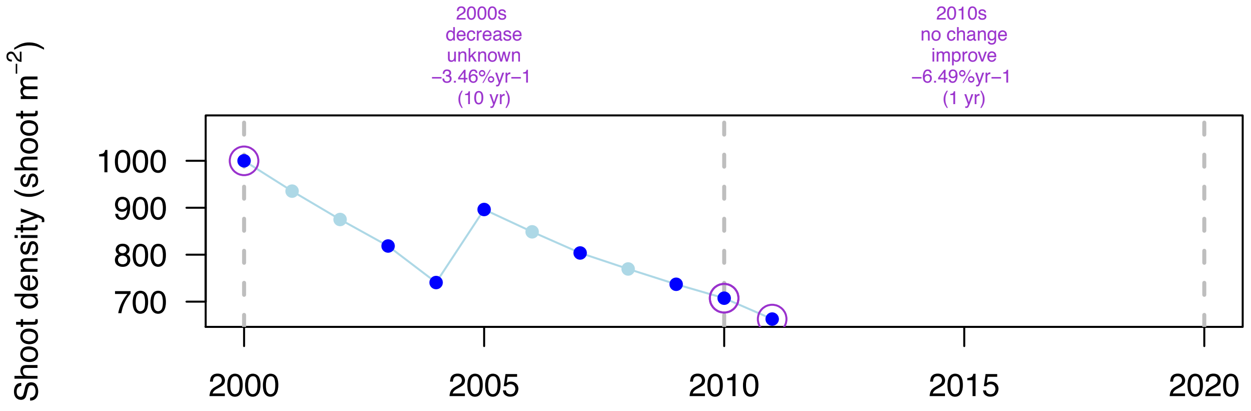

559_density

Marbà and Duarte 2010, Duarte and Marbà (unpublished)

SITE: El Castell (Spain – Mediterranean) – Po (–20 m)

OVERALL: Net = –93.75 shoot m–2;   Rate = –4.51 % yr–1; Perc Final = 61 % > decrease

DECADAL: YES (11 yr)

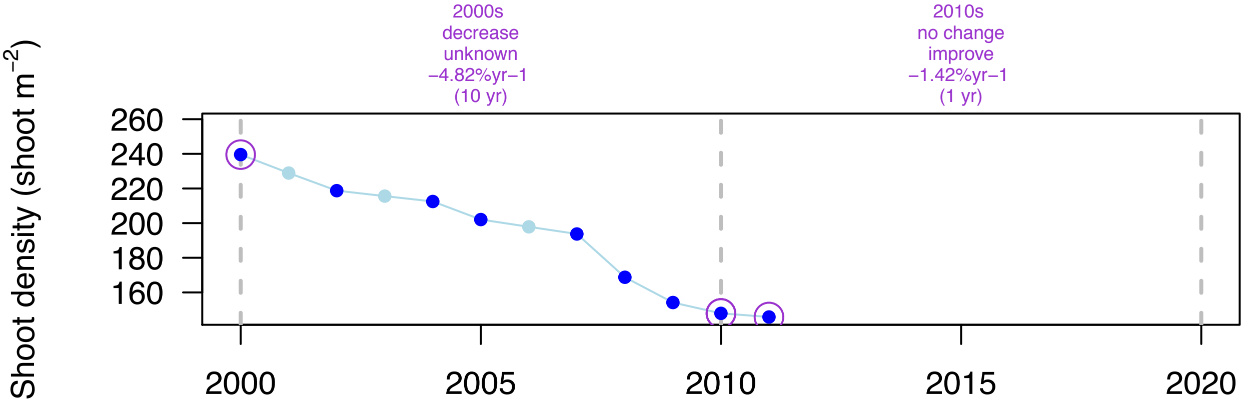

560_lowerlimit

Aliani et al. 1998, Dando et al. 1995

SITE: Paleochori Bay (Greece – Mediterranean) – Po (? m)

OVERALL: Net = 0 m;   Rate = 0 % yr–1; Perc Final = 100 % > no change

DECADAL: NO (4 yr)

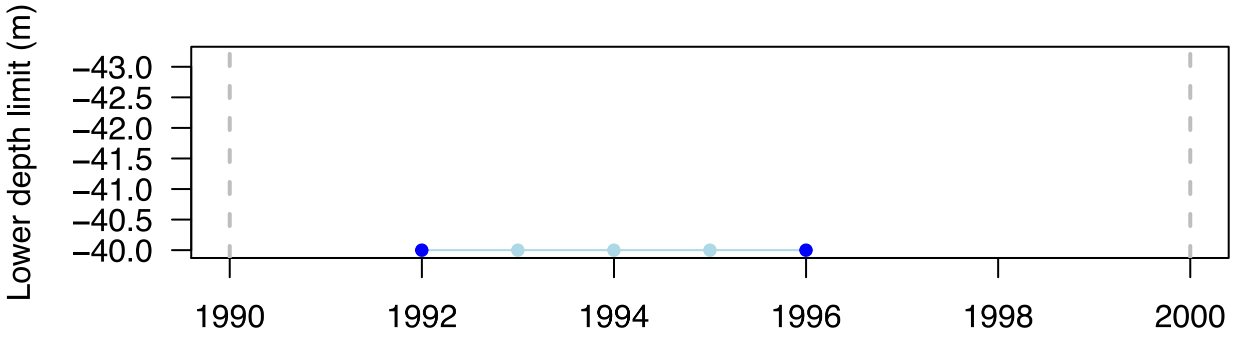

560_upperlimit

Aliani et al. 1998, Dando et al. 1995

SITE: Paleochori Bay (Greece – Mediterranean) – Po (? m)

OVERALL: Net = 0 m;   Rate = 0 % yr–1; Perc Final = 100 % > no change

DECADAL: NO (4 yr)

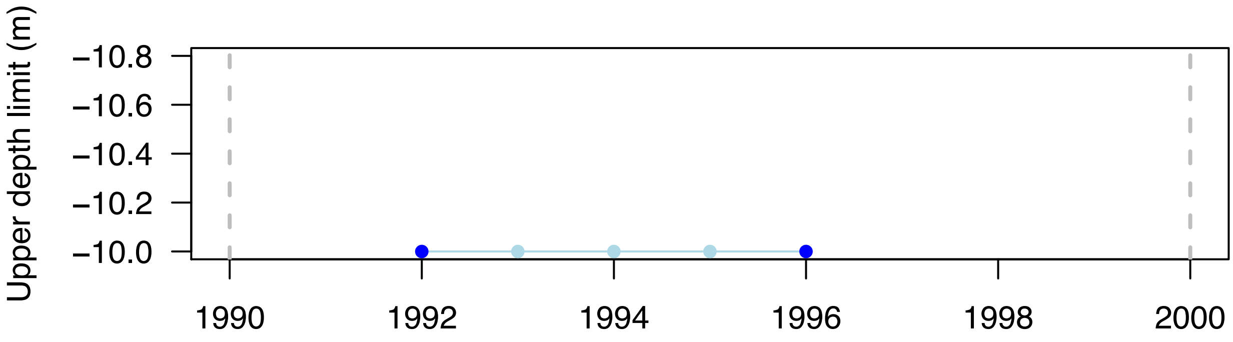

561_area

Auby et al. 2010

SITE: Baie de Morlaix (France – Atlantic) – Zm (? m)

OVERALL: Net = 180 ha;   Rate = 10.47 % yr–1; Perc Final = 433 % > increase

DECADAL: YES (14 yr)

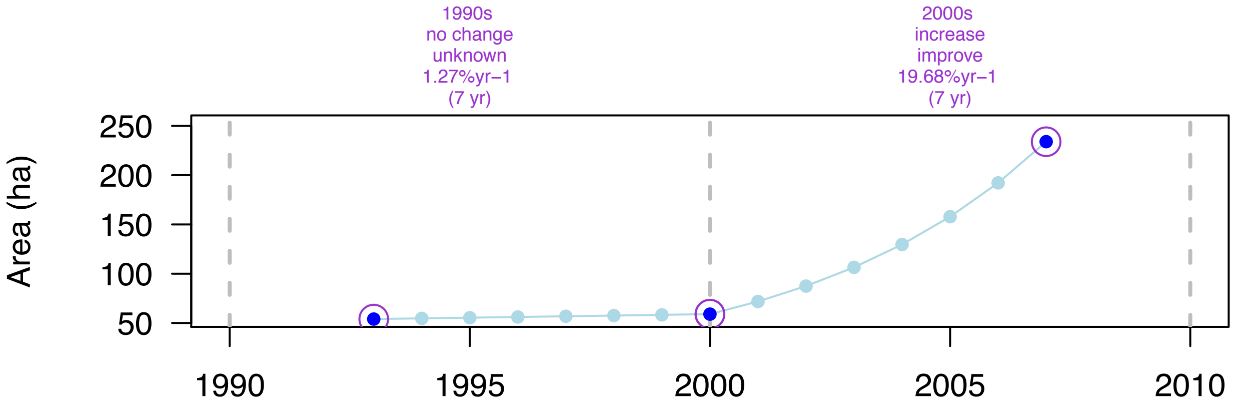

563_abiomass

Auby et al. 2010

SITE: Golfe du Morbihan (France – Atlantic) – Zn (0 m)

OVERALL: Net = –56.5 g dw m–2;   Rate = –3.33 % yr–1; Perc Final = 44 % > decrease

DECADAL: YES (25 yr)

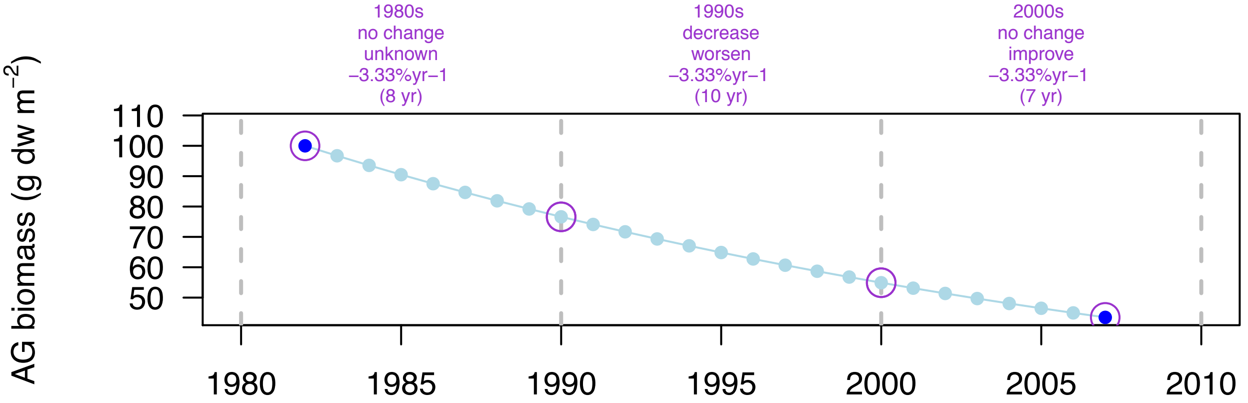

563_density

Auby et al. 2010

SITE: Golfe du Morbihan (France – Atlantic) – Zn (0 m)

OVERALL: Net = 8543 shoot m–2;   Rate = 5.67 % yr–1; Perc Final = 413 % > increase

DECADAL: YES (25 yr)

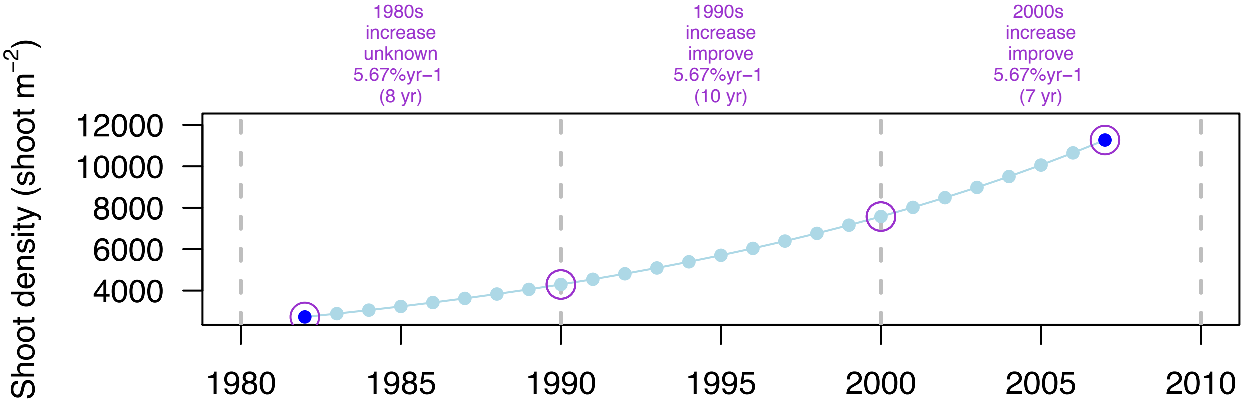

564_abiomass

Auby et al. 2010

SITE: Golfe du Morbihan (France – Atlantic) – Zm (0 m)

OVERALL: Net = –96.9 g dw m–2;   Rate = –5.15 % yr–1; Perc Final = 22 % > decrease

DECADAL: YES (29 yr)

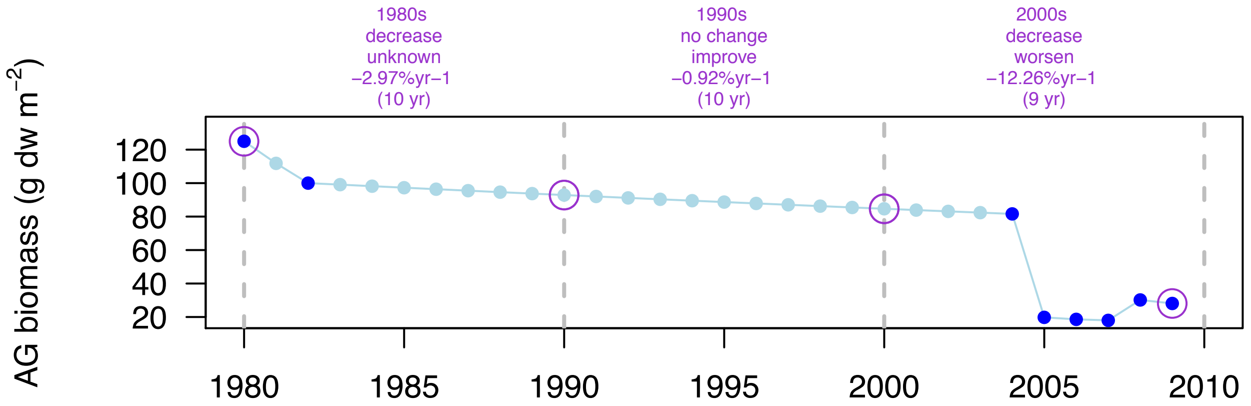

564_density

Auby et al. 2010

SITE: Golfe du Morbihan (France – Atlantic) – Zm (0 m)

OVERALL: Net = –1982 shoot m–2;   Rate = –10.71 % yr–1; Perc Final = 4 % > decrease

DECADAL: YES (29 yr)

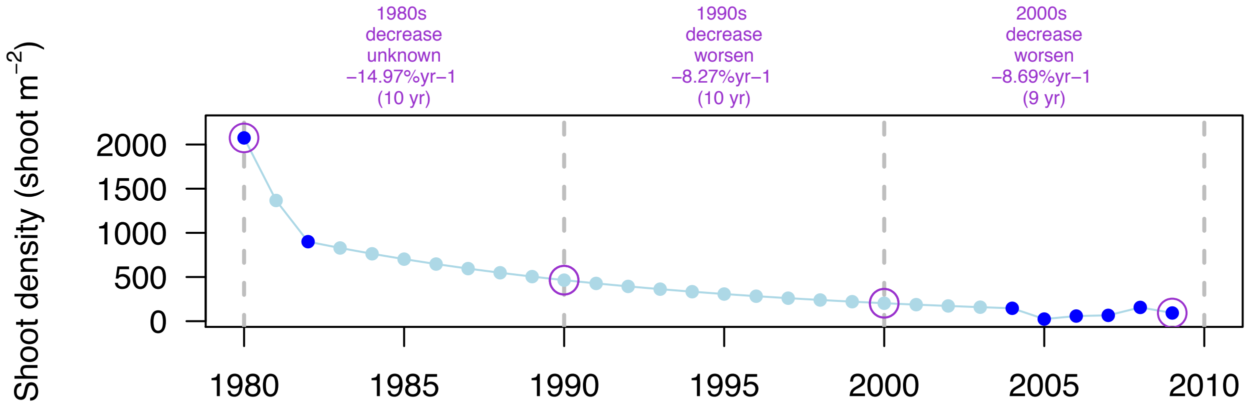

567_area

Arroyo et al. 2015

SITE: Bahía de Algeciras (Spain – Mediterranean) – Cn (? m)

OVERALL: Net = –400 ha;   Rate = NA % yr–1; Perc Final = NA % > decrease

DECADAL: YES (10 yr)

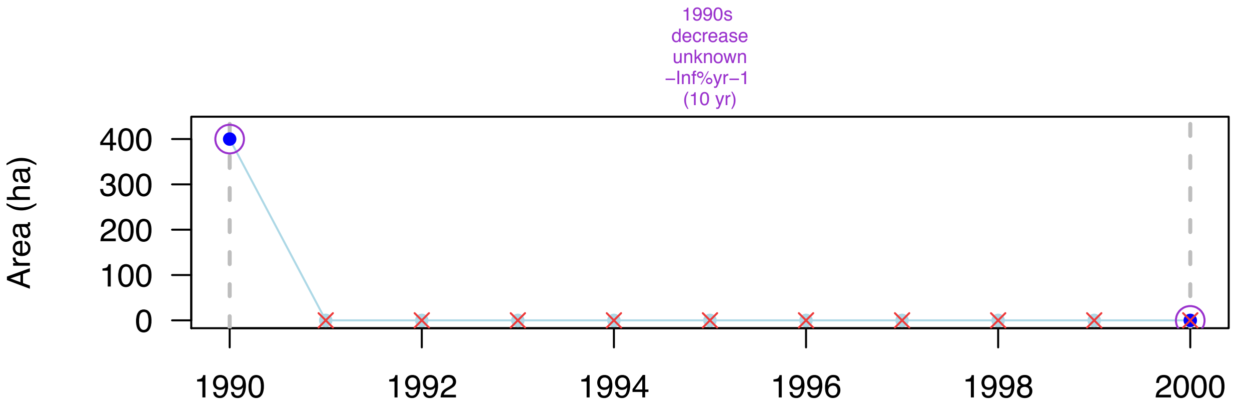

568_cover

Arroyo et al. 2015

SITE: Cala Chinches (Spain – Mediterranean) – Po (? m)

OVERALL: Net = –30 %;   Rate = –16.18 % yr–1; Perc Final = 62 % > decrease

DECADAL: NO (3 yr)

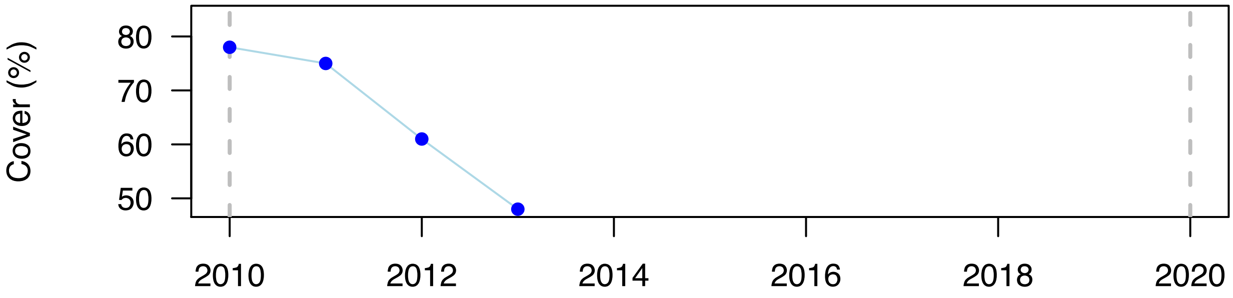

568_density

Arroyo et al. 2015

SITE: Cala Chinches (Spain – Mediterranean) – Po (? m)

OVERALL: Net = 90 shoot m–2;   Rate = 4.59 % yr–1; Perc Final = 115 % > no change

DECADAL: NO (3 yr)

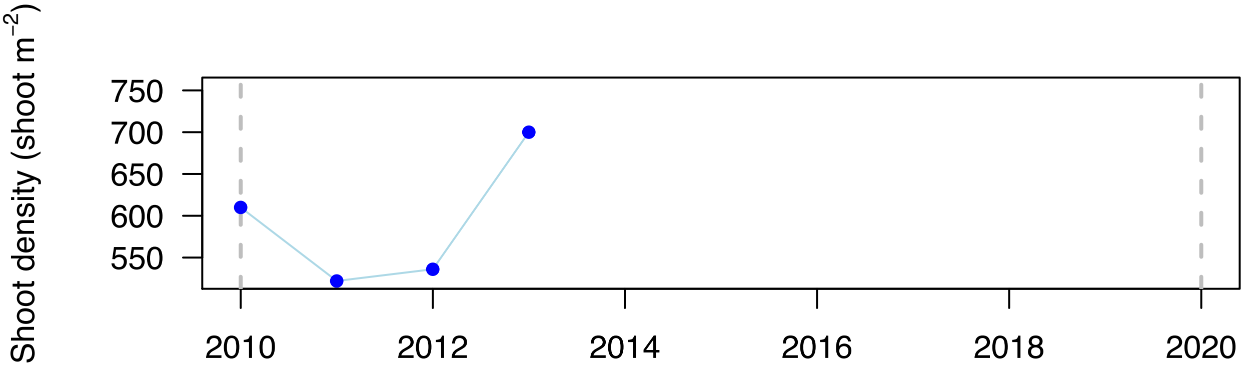

569_density

Arroyo et al. 2015

SITE: Calaburras (Peñón del Fraile) (Spain – Mediterranean) – Po (? m)

OVERALL: Net = –30 shoot m–2;   Rate = –0.54 % yr–1; Perc Final = 96 % > no change

DECADAL: NO (7 yr)

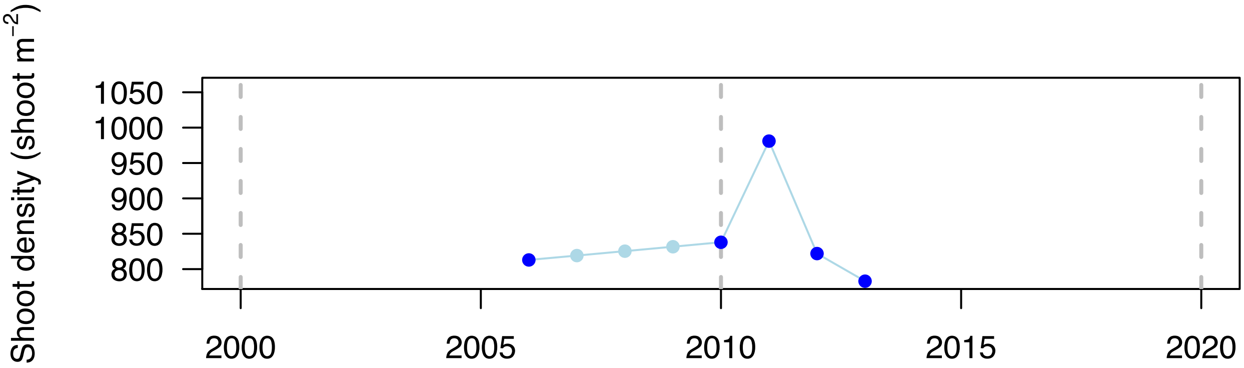

570_cover

Arroyo et al. 2015

SITE: Cambriles (Spain – Mediterranean) – Po (? m)

OVERALL: Net = −33 %;   Rate = −12.29 % yr−1; Perc Final = 61 % > decrease

DECADAL: NO (4 yr)

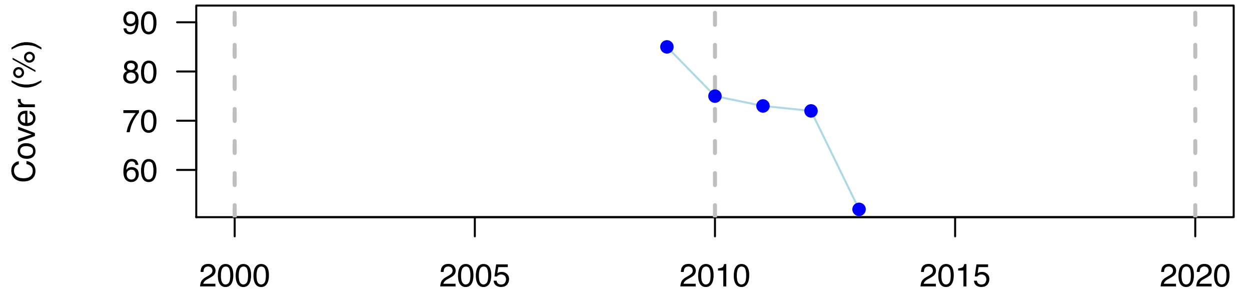

570_density

Arroyo et al. 2015

SITE: Cambriles (Spain – Mediterranean) – Po (? m)

OVERALL: Net = 76 shoot m−2;   Rate = 5.13 % yr−1; Perc Final = 123 % > no change

DECADAL: NO (4 yr)

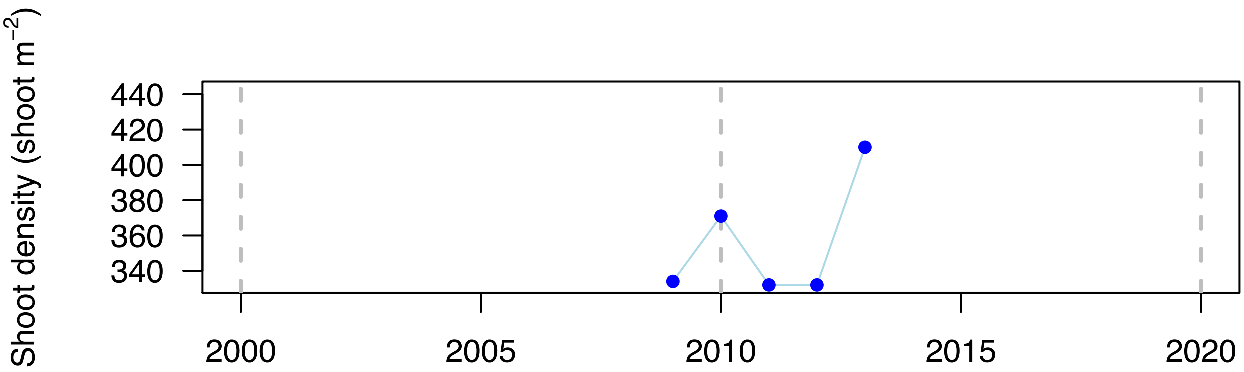

571_cover Arroyo et al. 2015

SITE: El Lance (Spain – Mediterranean) – Po (? m)

OVERALL: Net = –10 %;   Rate = –5.34 % yr–1; Perc Final = 81 % > no change

DECADAL: NO (4 yr)

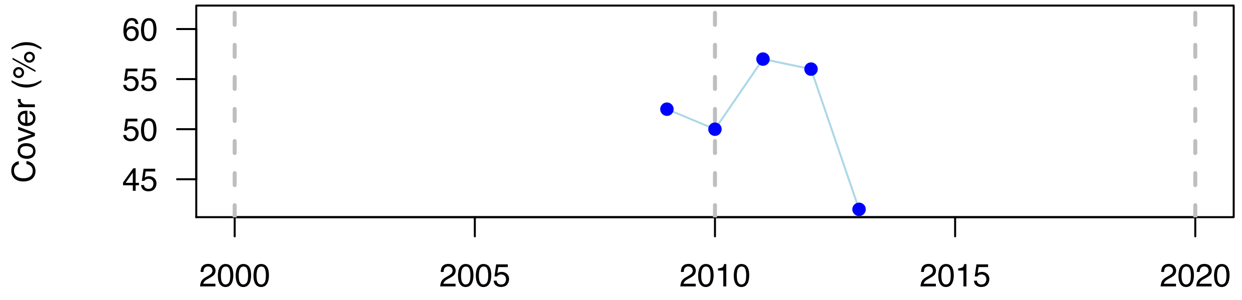

571_density Arroyo et al. 2015

SITE: El Lance (Spain – Mediterranean) – Po (? m)

OVERALL: Net = 198 shoot m–2;   Rate = 12.25 % yr–1; Perc Final = 163 % > increase

DECADAL: NO (4 yr)

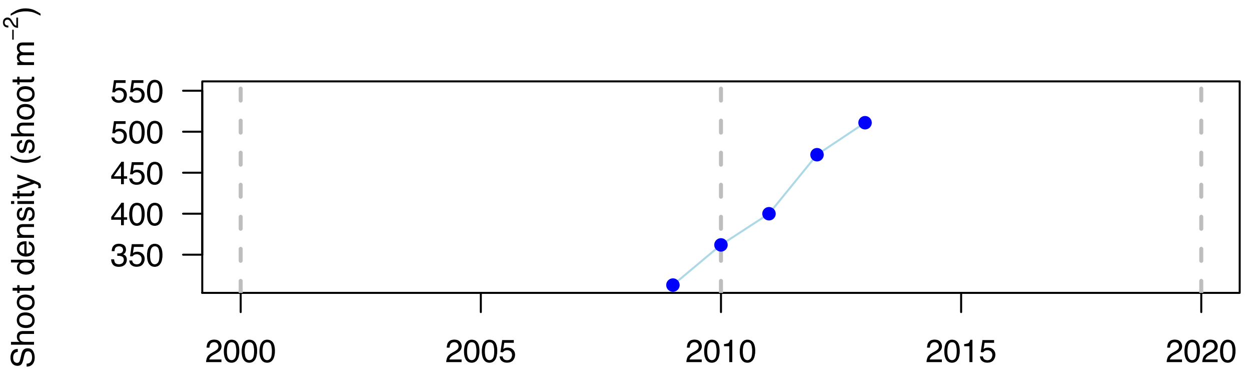

572_cover

Arroyo et al. 2015

SITE: Melicena (Spain – Mediterranean) – Po (–7 m)

OVERALL: Net = –6 %; Rate = –2.74 % yr–1; Perc Final = 92 % > no change

DECADAL: NO (3 yr)

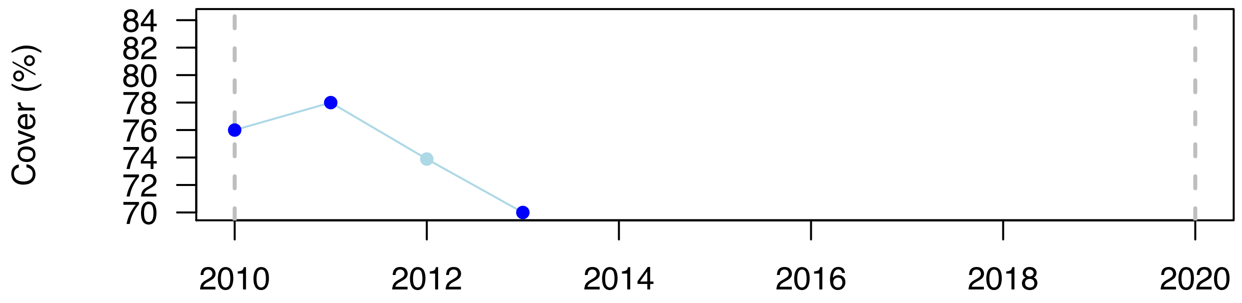

572_density

Arroyo et al. 2015

SITE: Melicena (Spain – Mediterranean) – Po (–7 m)

OVERALL: Net = 114 shoot m–2; Rate = 6.38 % yr–1; Perc Final = 121 % > no change

DECADAL: NO (3 yr)

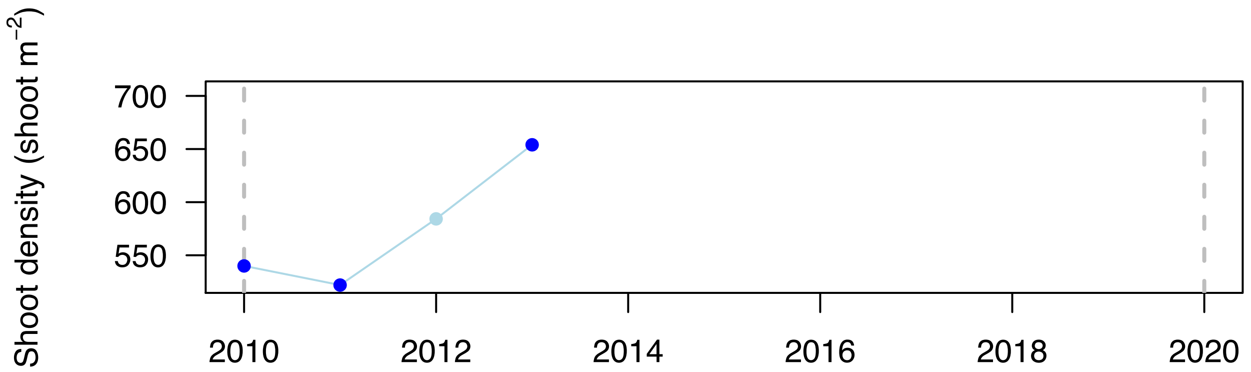

573_density

Arroyo et al. 2015

SITE: Nerja (Spain – Mediterranean) – Po (? m)

OVERALL: Net = 436 shoot m–2;   Rate = 8.47 % yr–1; Perc Final = 181 % > increase

DECADAL: NO (7 yr)

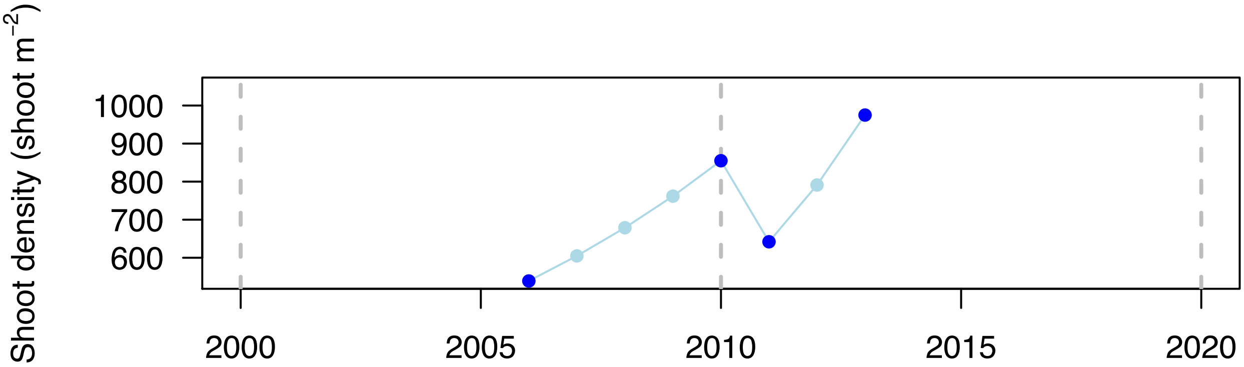

574_density

Arroyo et al. 2015

SITE: Molino de Papel (Spain – Mediterranean) – Po (? m)

OVERALL: Net = 113 shoot m–2;   Rate = 4.18 % yr–1; Perc Final = 134 % > increase

DECADAL: NO (7 yr)

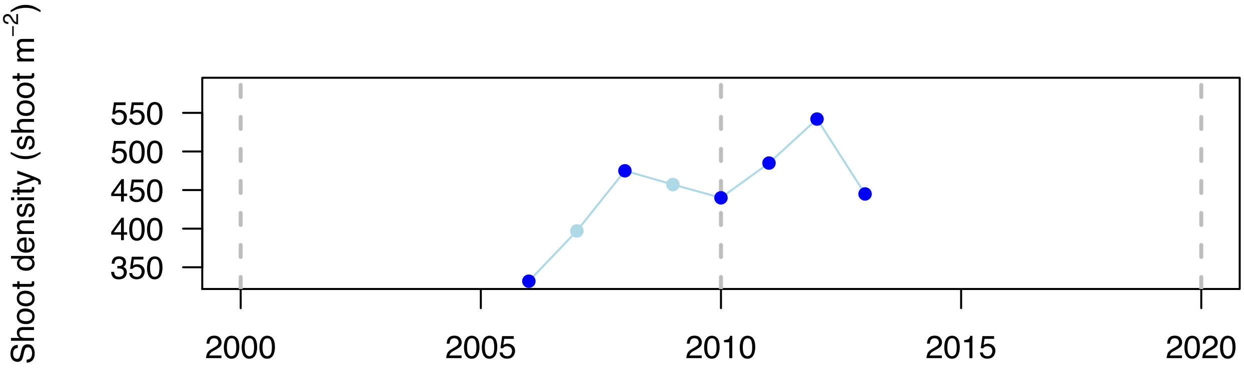

578_area

Rueda et al. 2009

SITE: Playa del Cañuelo (Spain – Mediterranean) – Zm (? m)

OVERALL: Net = –38.8 ha;   Rate = NA % yr–1; Perc Final = NA % > decrease

DECADAL: NO (6 yr)

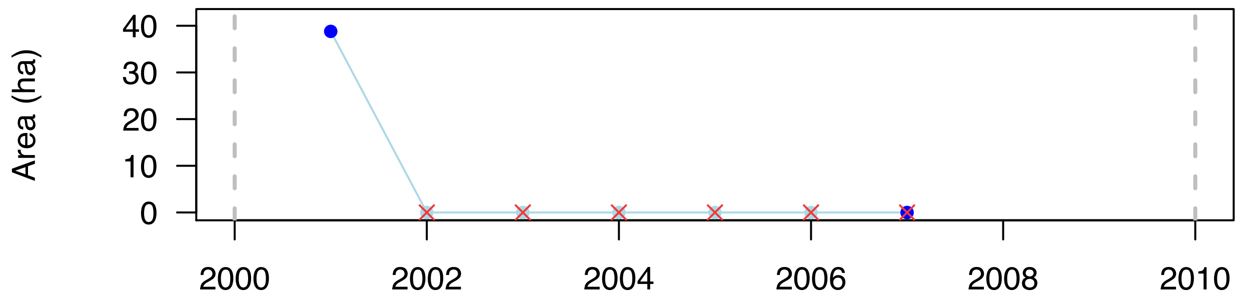

578_biomass

Rueda et al. 2009

SITE: Playa del Cañuelo (Spain – Mediterranean) – Zm (? m)

OVERALL: Net = –187.97 g dw m–2;   Rate = NA % yr–1; Perc Final = NA % > decrease

DECADAL: NO (3 yr)

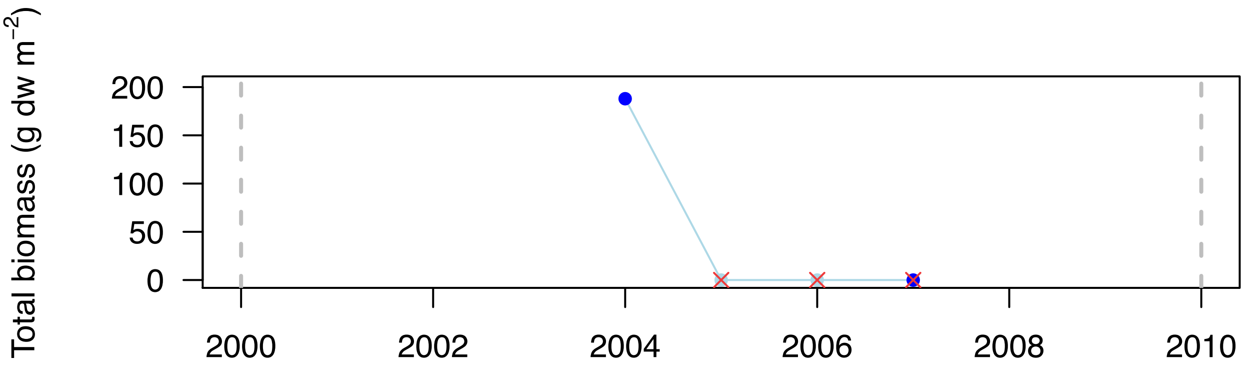

578_density

Rueda et al. 2009

SITE: Playa del Cañuelo (Spain – Mediterranean) – Zm (? m)

OVERALL: Net = −404 shoot m−2;   Rate = NA % yr−1; Perc Final = NA % > decrease

DECADAL: NO (3 yr)

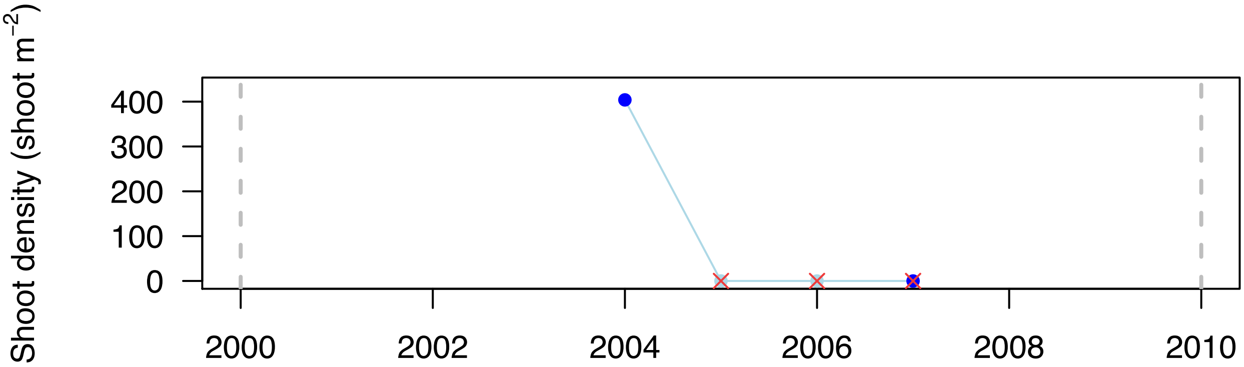

586_upperlimit

Aragonés et al. 2015

SITE: Poniente Beach Benidorm (Spain – Mediterranean) – Po (? m)

OVERALL: Net = −3.5 m;   Rate = −4.2 % yr−1; Perc Final = 53 % > decrease

DECADAL: YES (15 yr)

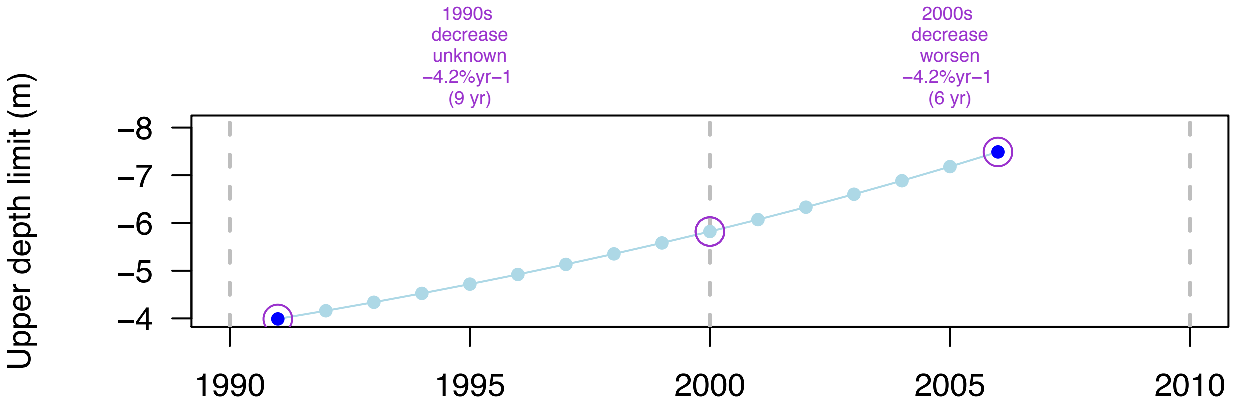

596_area

Auby et al. 2010

SITE: Les Abers Large (France – Atlantic) – Zm (? m)

OVERALL: Net = 128 ha;   Rate = 7.7 % yr–1; Perc Final = 294 % > increase

DECADAL: YES (14 yr)

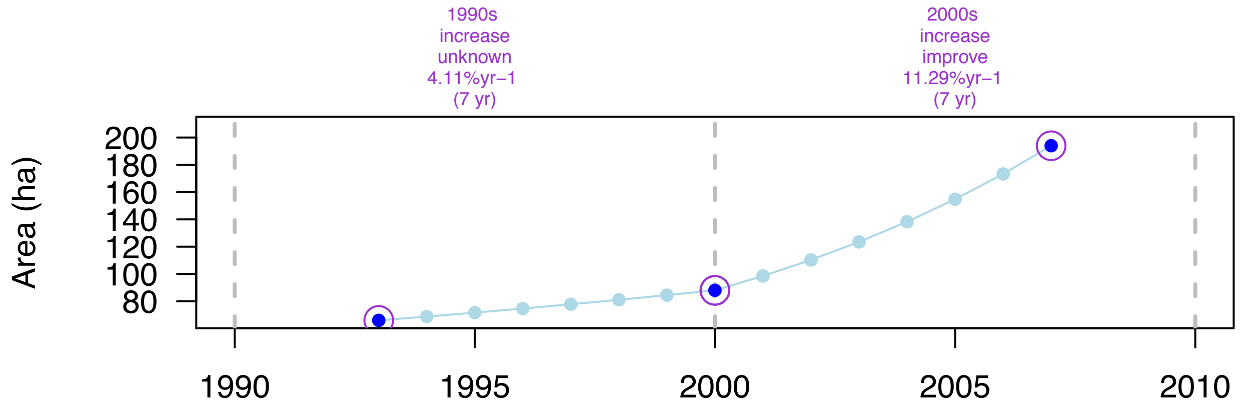

597_abiomass

Auby et al. 2010

SITE: Roscanavel (France – Atlantic) – Zm (? m)

OVERALL: Net = –29.1 g dw m–2;   Rate = –4.45 % yr–1; Perc Final = 80 % > no change

DECADAL: NO (5 yr)

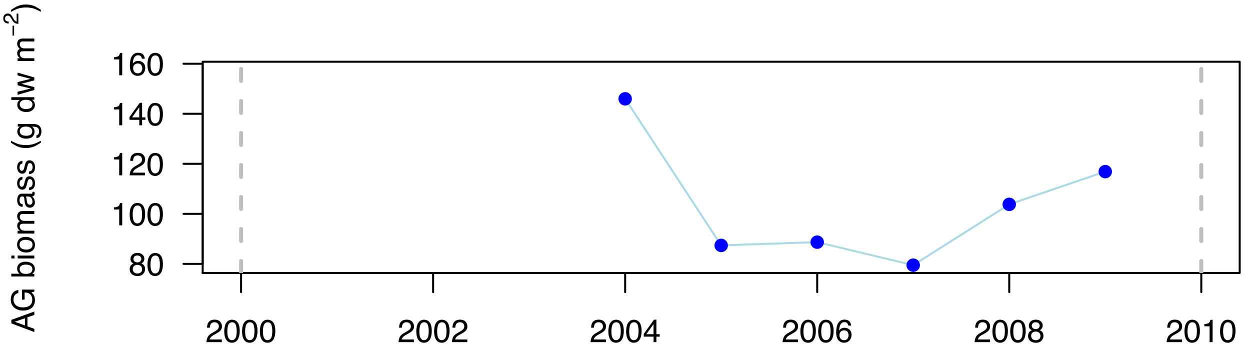

597_density

Auby et al. 2010

SITE: Roscanavel (France – Atlantic) – Zm (? m)

OVERALL: Net = –176 shoot m–2;   Rate = –12.91 % yr–1; Perc Final = 52 % > decrease

DECADAL: NO (5 yr)

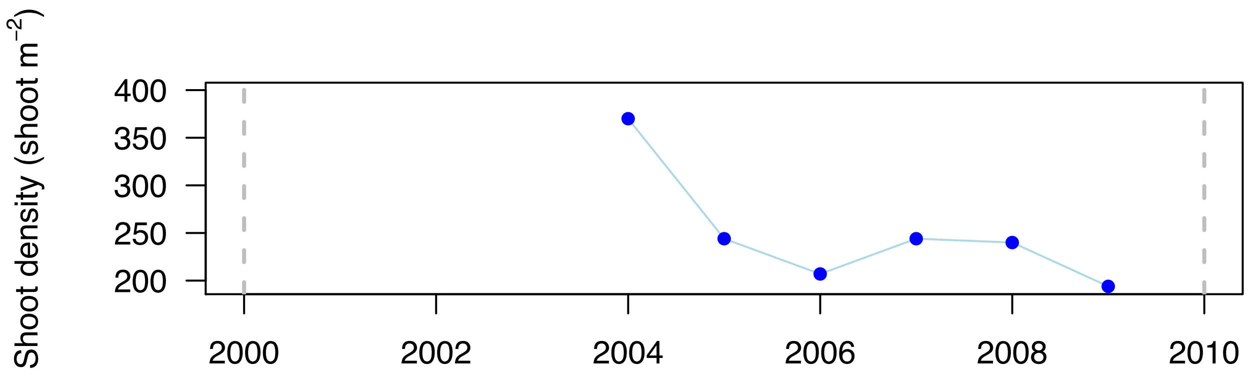

598_abiomass

Auby et al. 2010

SITE: Plage de la Charge Neuve (France – Atlantic) – Zn (? m)

OVERALL: Net = 6.4 g dw m–2;   Rate = 4.55 % yr–1; Perc Final = 120 % > no change

DECADAL: NO (4 yr)

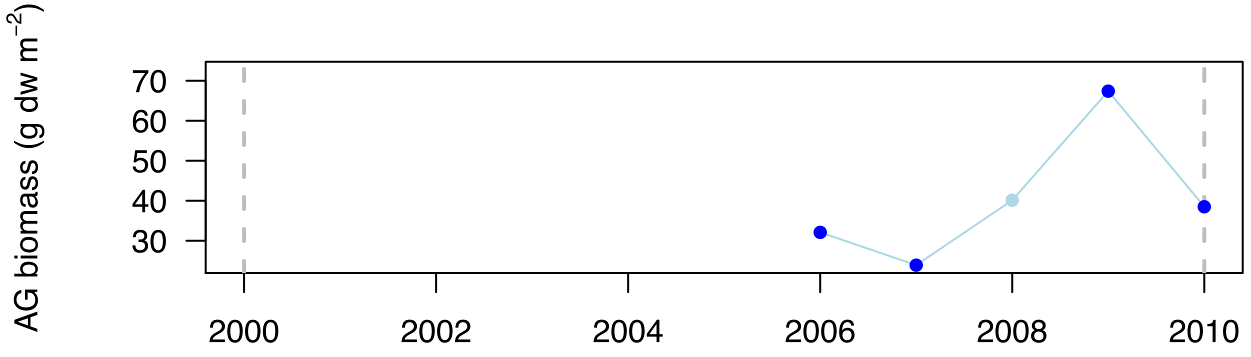

598_density

Auby et al. 2010

SITE: Plage de la Charge Neuve (France – Atlantic) – Zn (? m)

OVERALL: Net = −4506 shoot m−2;   Rate = −13.25 % yr−1; Perc Final = 59 % > decrease

DECADAL: NO (4 yr)

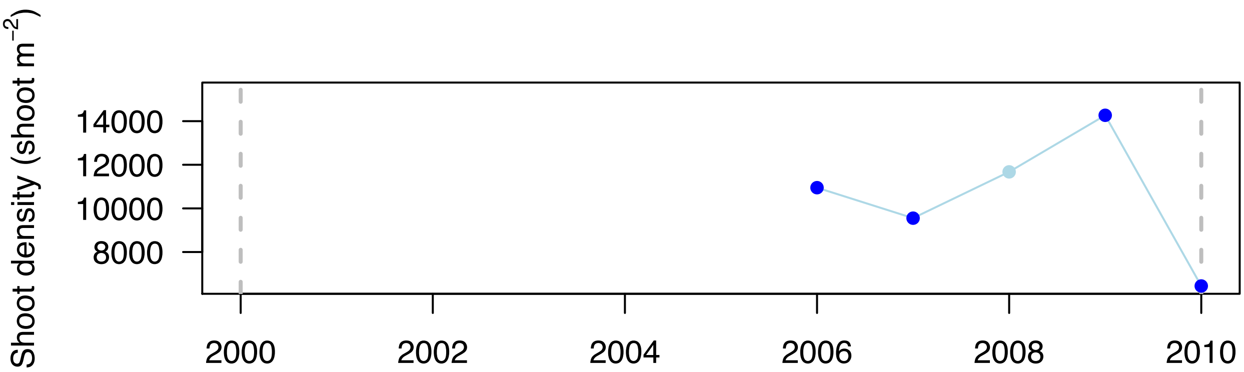

620_area

Calleja et al. 2017

SITE: Santander Bay (Spain – Atlantic) – Zn (0.9 m)

OVERALL: Net = 12.15 ha;   Rate = 5 % yr−1; Perc Final = 472 % > increase

DECADAL: YES (31 yr)

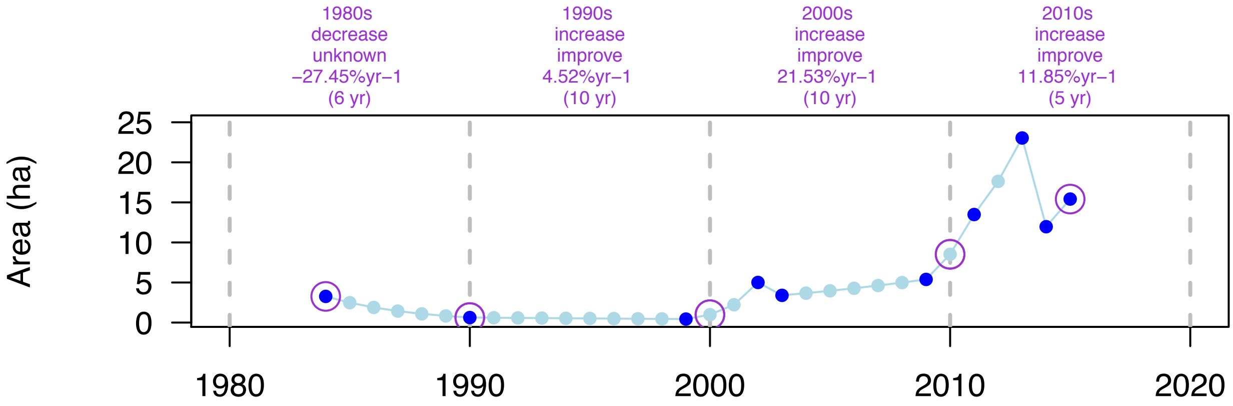

621_area

Calleja et al. 2017

SITE: Santander Bay (Spain – Atlantic) – Zn (0.3 m)

OVERALL: Net = 7.48 ha; Rate = 2.15 % yr–1; Perc Final = 194 % > increase

DECADAL: YES (31 yr)

1980s	1990s	2000s	2010s
decrease	no change	increase	increase
unknown	improve	improve	improve
–4.79%yr–1	0.83%yr–1	7.58%yr–1	2.23%yr–1
(6 yr)	(10 yr)	(10 yr)	(5 yr)

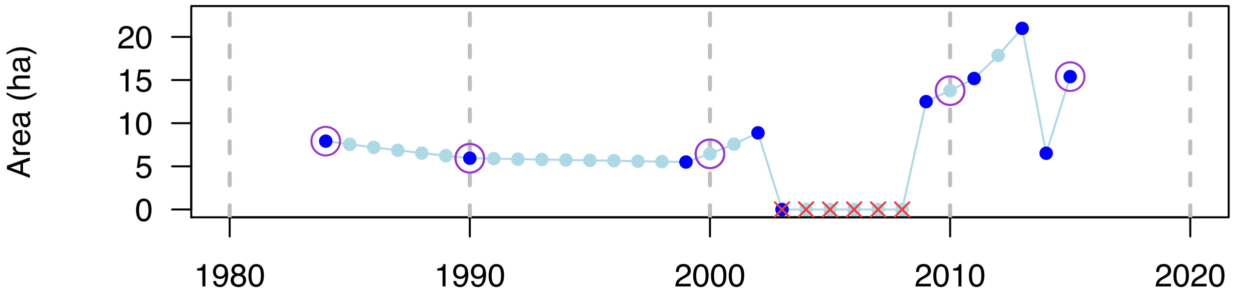

622_area

Calleja et al. 2017

SITE: Santander Bay (Spain – Atlantic) – Zn (0.4 m)

OVERALL: Net = 18.23 ha; Rate = 2.57 % yr–1; Perc Final = 222 % > increase

DECADAL: YES (31 yr)

1980s	1990s	2000s	2010s
increase	decrease	increase	decrease
unknown	worsen	improve	worsen
8.04%yr–1	–8.7%yr–1	17.4%yr–1	–11.09%yr–1
(6 yr)	(10 yr)	(10 yr)	(5 yr)

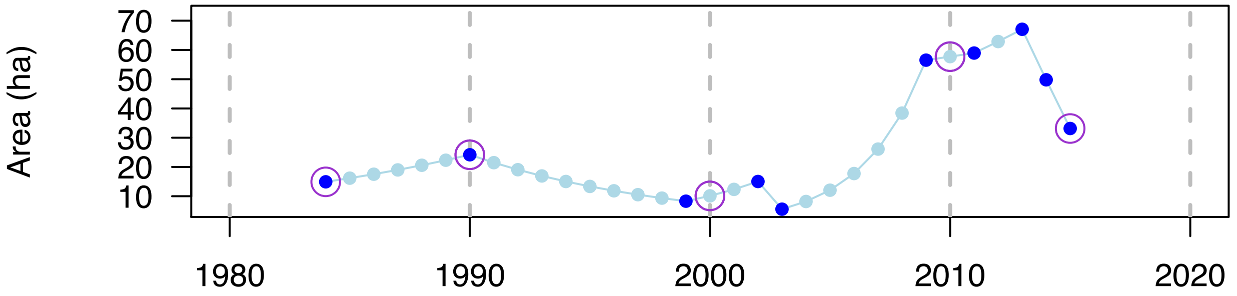

623_area

Calleja et al. 2017

SITE: Santander Bay (Spain – Atlantic) – Zn (–0.7 m)

OVERALL: Net = 10.89 ha;   Rate = 3.48 % yr–1; Perc Final = 239 % > increase

DECADAL: YES (25 yr)

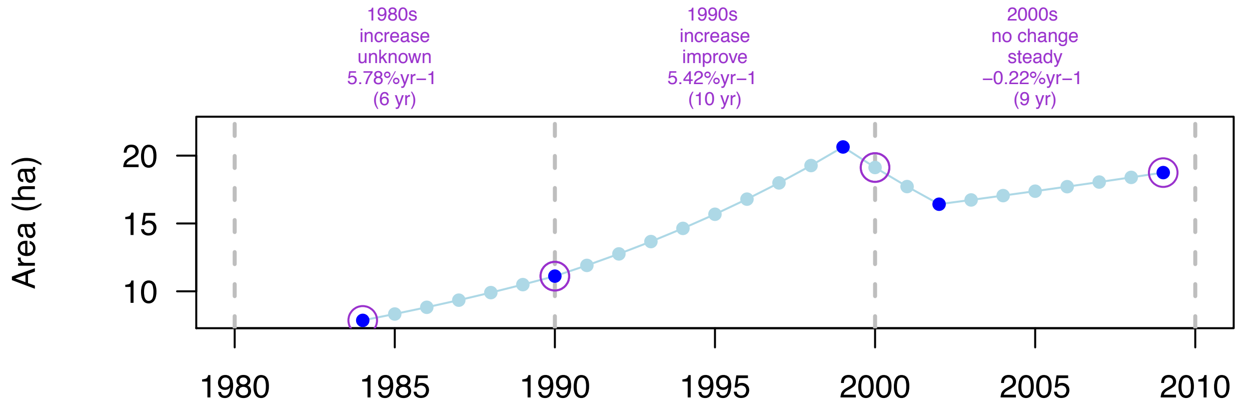

624_density

Bernard et al. 2005

SITE: Berre Lagoon (La Mède oil terminal) (France – Mediterranean) – Zm (–2 m)

OVERALL: Net = 66.5 shoot m–2;   Rate = NA % yr–1; Perc Final = NA % > increase

DECADAL: NO (2 yr)

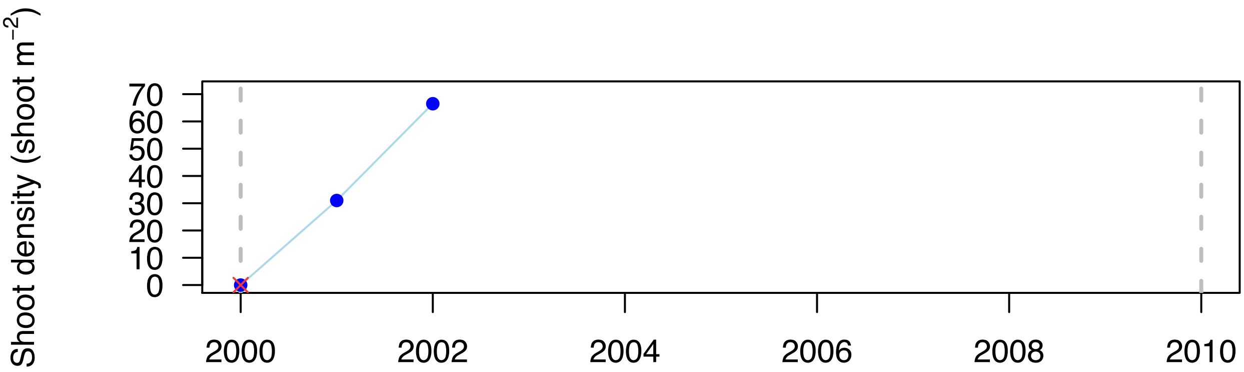

641_area

de Paz et al. 2008

SITE: Linera inlet (Spain – Atlantic) – Zn (? m)

OVERALL: Net = –38 ha; Rate = –2.11 % yr–1; Perc Final = 73 % > decrease

DECADAL: YES (15 yr)

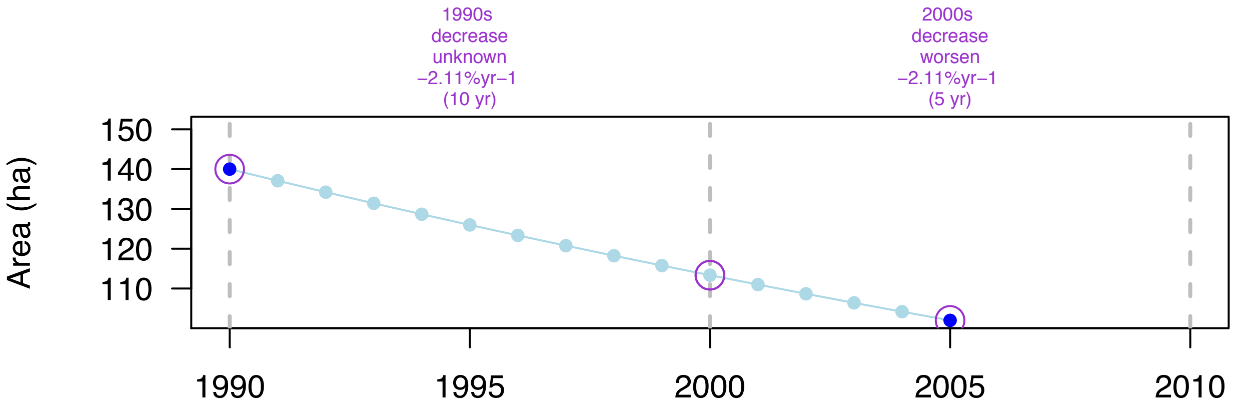

658_biomass

Kruk–Dowgiallo 1991, Gic–Grusza et al. 2009, Kruk–Dowgiallo and Szaniawska 2008

SITE: Puck Bay (Poland – Baltic) – Zm (–3.1 m)

OVERALL: Net = –16.2 g dw m–2; Rate = –1.44 % yr–1; Perc Final = 56 % > decrease

DECADAL: YES (40 yr)

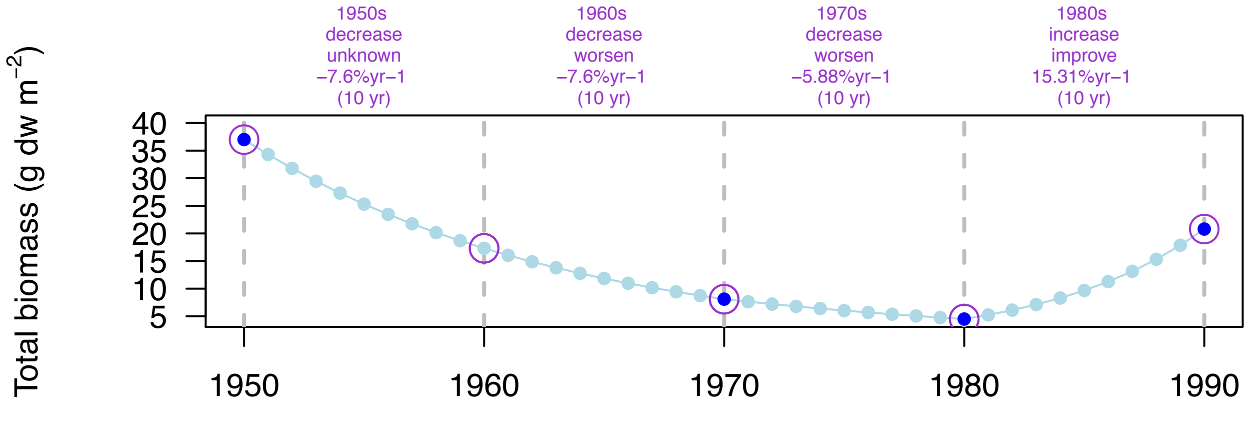

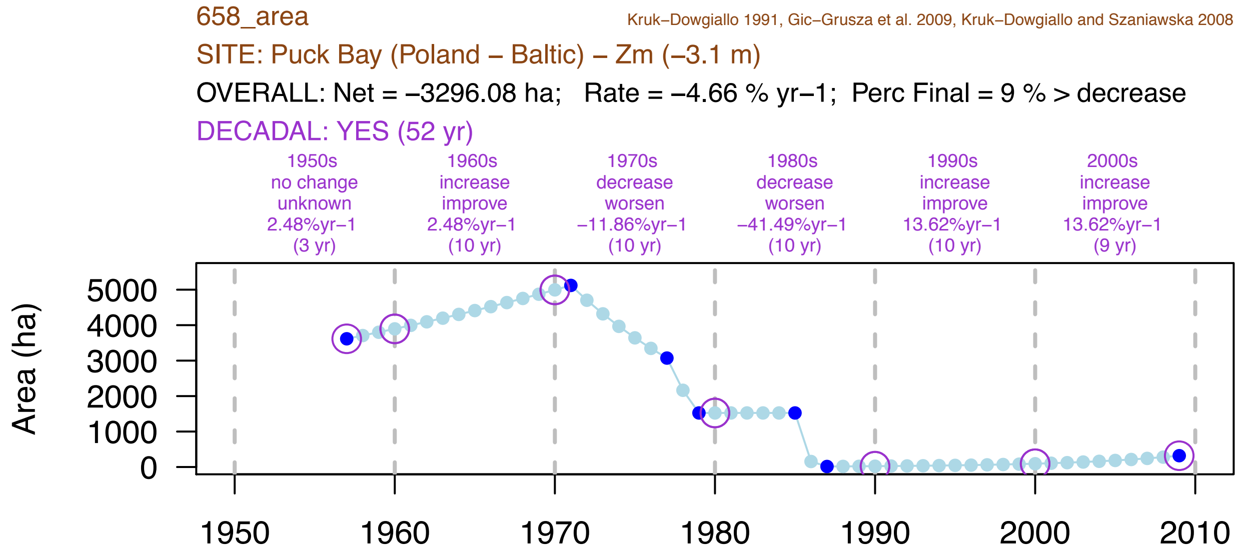
658_area
Kruk–Dowgiallo 1991, Gic–Grusza et al. 2009, Kruk–Dowgiallo and Szaniawska 2008
SITE: Puck Bay (Poland – Baltic) – Zm (–3.1 m)
OVERALL: Net = –3296.08 ha; Rate = –4.66 % yr–1; Perc Final = 9 % > decrease
DECADAL: YES (52 yr)
1950s no change unknown 2.48%yr–1 (3 yr)
1960s increase improve 2.48%yr–1 (10 yr)
1970s decrease worsen –11.86%yr–1 (10 yr)
1980s decrease worsen –41.49%yr–1 (10 yr)
1990s increase improve 13.62%yr–1 (10 yr)
2000s increase improve 13.62%yr–1 (9 yr)
Area (ha)
5000
4000
3000
2000
1000
0
1950
1960
1970
1980
1990
2000
2010

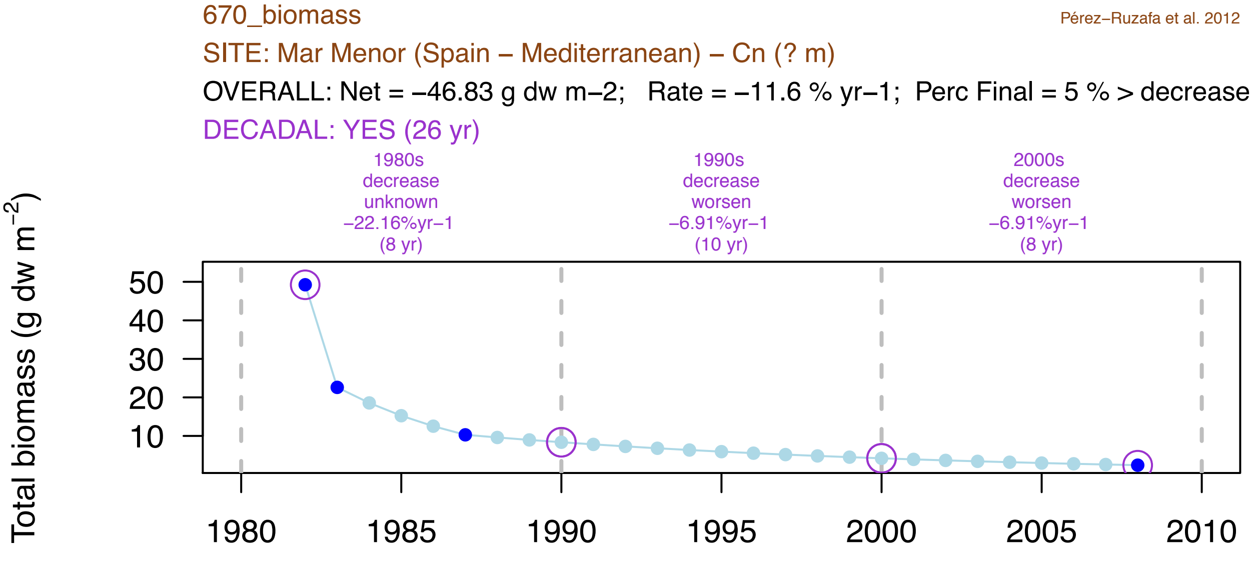
670_biomass
Pérez–Ruzafa et al. 2012
SITE: Mar Menor (Spain – Mediterranean) – Cn (? m)
OVERALL: Net = –46.83 g dw m–2; Rate = –11.6 % yr–1; Perc Final = 5 % > decrease
DECADAL: YES (26 yr)
1980s decrease unknown –22.16%yr–1 (8 yr)
1990s decrease worsen –6.91%yr–1 (10 yr)
2000s decrease worsen –6.91%yr–1 (8 yr)
Total biomass (g dw m^{-2})
50
40
30
20
10
1980
1985
1990
1995
2000
2005
2010

684_lowerlimit

Meinesz et al. 2002

SITE: Elbu Bay (France – Mediterranean) – Po (–35.5 m)

OVERALL: Net = –3 m; Rate = –0.4 % yr–1; Perc Final = 92 % > no change

DECADAL: YES (22 yr)

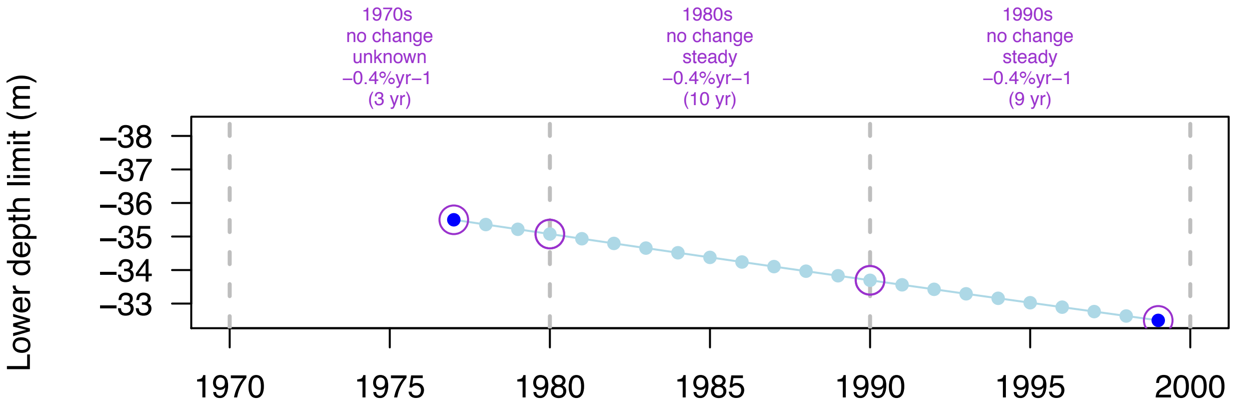

691_area

Arroyo et al. 2015, Consejería de Medio Ambiente 2016

SITE: Palmones (Spain – Mediterranean) – Zn (? m)

OVERALL: Net = 1.43 ha; Rate = NA % yr–1; Perc Final = NA % > increase

DECADAL: YES (26 yr)

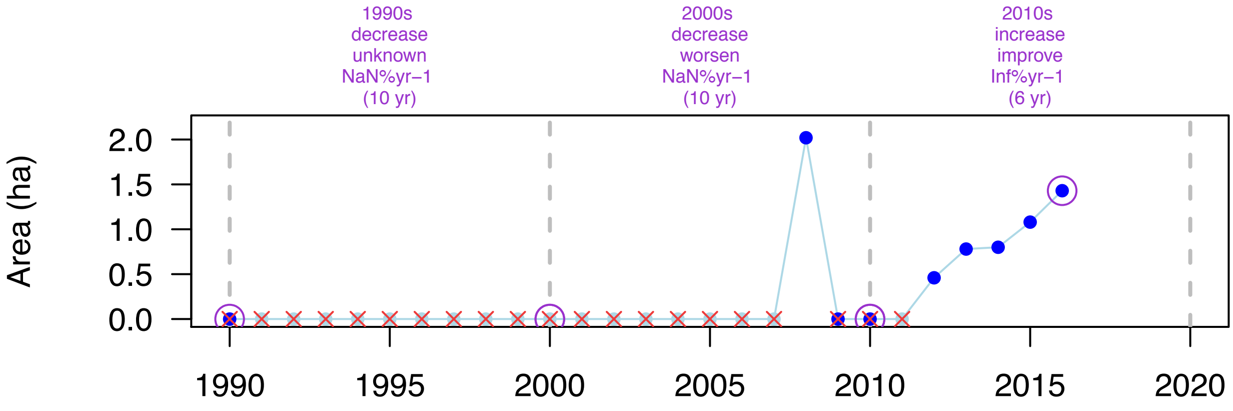

691_cover

Arroyo et al. 2015, Consejería de Medio Ambiente 2016

SITE: Palmones (Spain – Mediterranean) – Zn (? m)

OVERALL: Net = 23.34 %; Rate = 8.9 % yr–1; Perc Final = 186 % > increase

DECADAL: NO (7 yr)

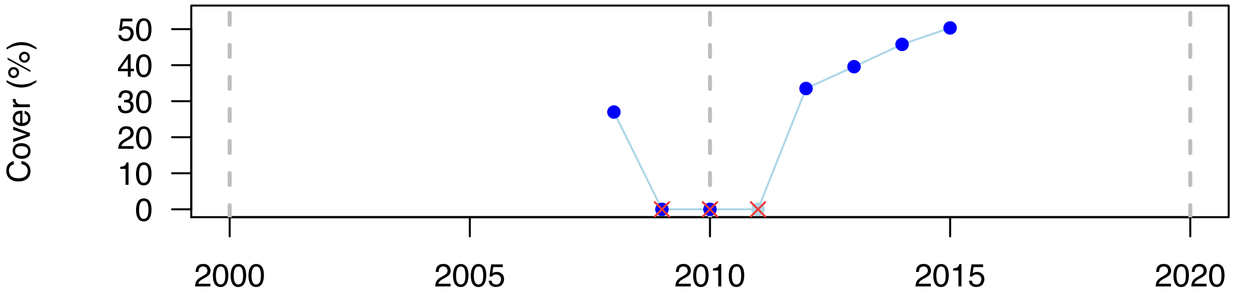

730_area

Mancusi et al. 2011

SITE: Piombino (Italy – Mediterranean) – Po (? m)

OVERALL: Net = –68.6 ha; Rate = –0.43 % yr–1; Perc Final = 91 % > no change

DECADAL: YES (21 yr)

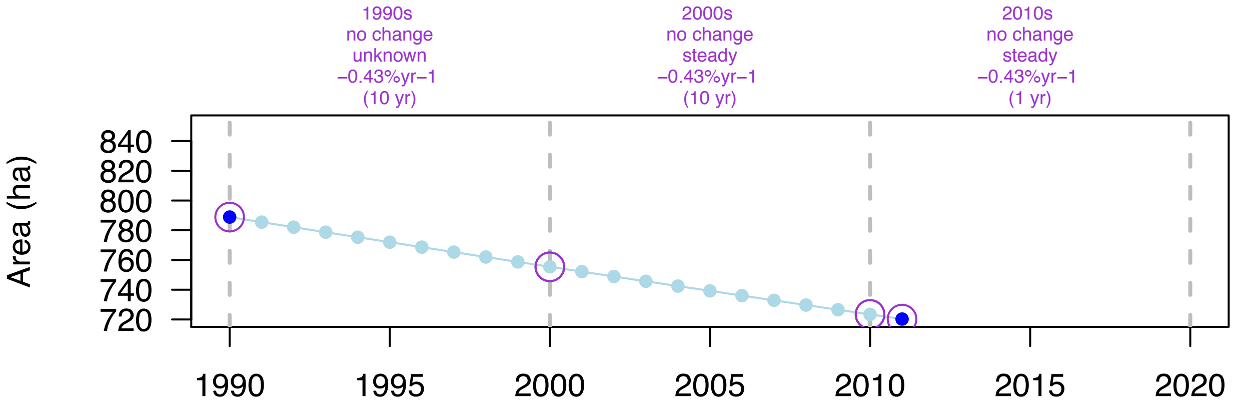

773_abiomass

Auby et al. 2010

SITE: Saint Malo (Rance – Fresnaye) (France – Atlantic) – Zm (? m)

OVERALL: Net = –4.5 g dw m–2;   Rate = –2.93 % yr–1; Perc Final = 86 % > no change

DECADAL: NO (5 yr)

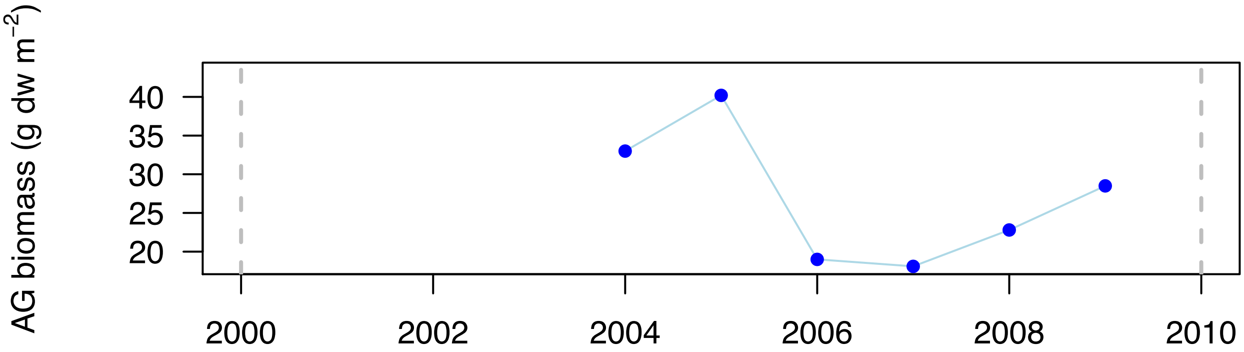

773_density

Auby et al. 2010

SITE: Saint Malo (Rance – Fresnaye) (France – Atlantic) – Zm (? m)

OVERALL: Net = –341 shoot m–2;   Rate = –12.64 % yr–1; Perc Final = 53 % > decrease

DECADAL: NO (5 yr)

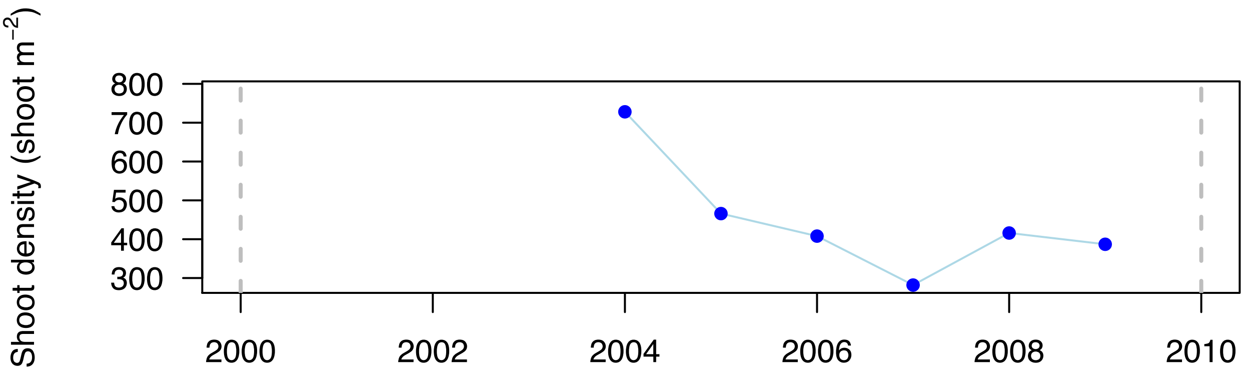

774_area
Auby et al. 2010
SITE: Rance – Fresnaye (France – Atlantic) – Zm (? m)
OVERALL: Net = 210 ha;   Rate = 9.61 % yr–1; Perc Final = 384 % > increase
DECADAL: YES (14 yr)

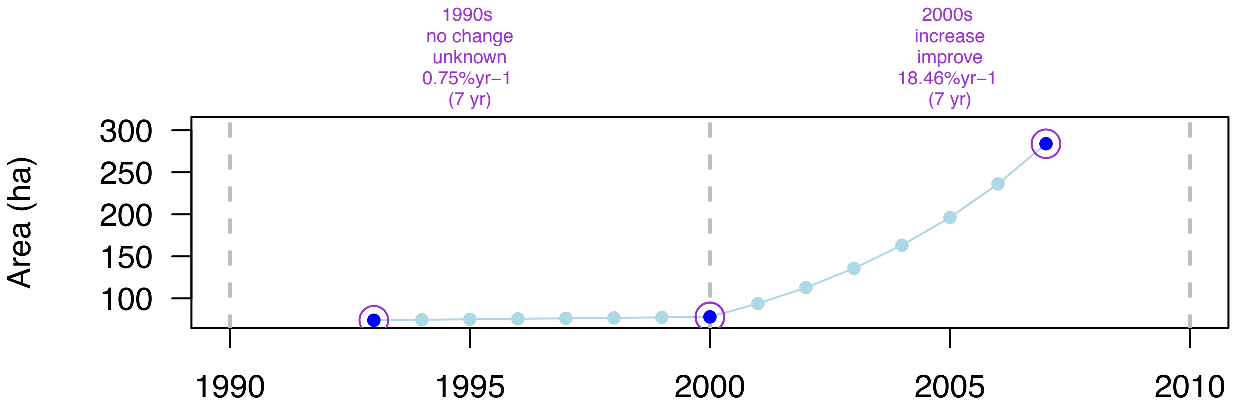

789_cover
Consejería de Medio Ambiente 2016
SITE: Río Piedras (Spain – Atlantic) – Zn (? m)
OVERALL: Net = –34.1 %;   Rate = –26.97 % yr–1; Perc Final = 58 % > decrease
DECADAL: NO (2 yr)

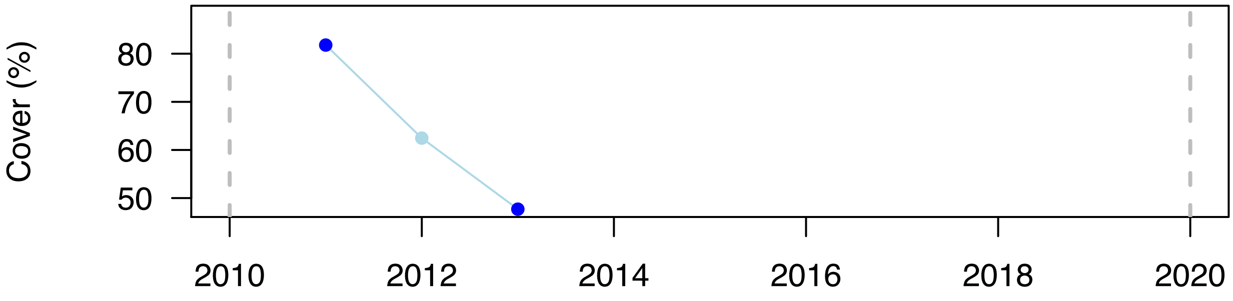

789_density

Consejería de Medio Ambiente 2016

SITE: Río Piedras (Spain – Atlantic) – Zn (? m)

OVERALL: Net = −1883 shoot m−2;   Rate = −20.57 % yr−1; Perc Final = 66 % > decrease

DECADAL: NO (2 yr)

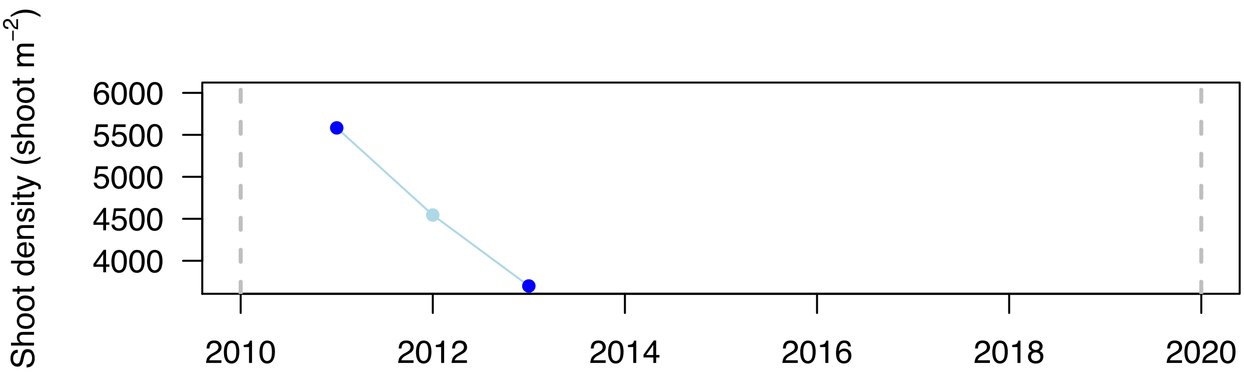

798_area

Cole 2016

SITE: Fishcombe Cove (United Kingdom – Atlantic) – Zm (? m)

OVERALL: Net = −0.07 ha;   Rate = −7.66 % yr−1; Perc Final = 68 % > decrease

DECADAL: NO (5 yr)

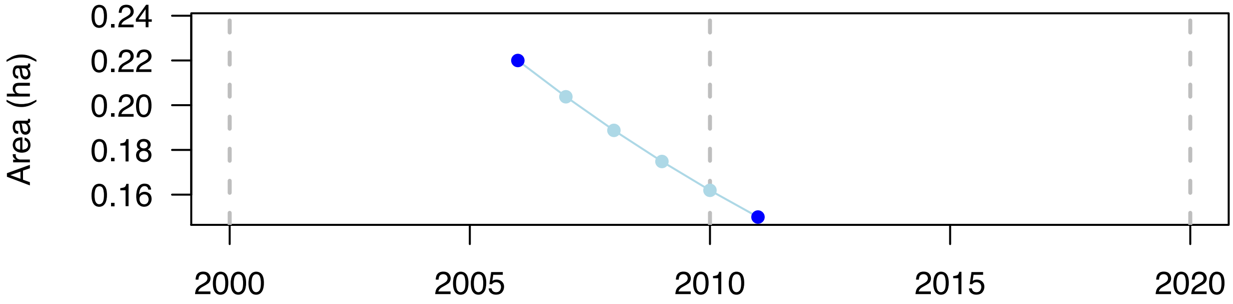

799_area

Wirtz 1995, Kaufmann and Maranhão 2017

SITE: Machico Bay (Portugal – Atlantic) – Cn (? m)

OVERALL: Net = –8 ha;   Rate = NA % yr–1; Perc Final = NA % > decrease

DECADAL: YES (17 yr)

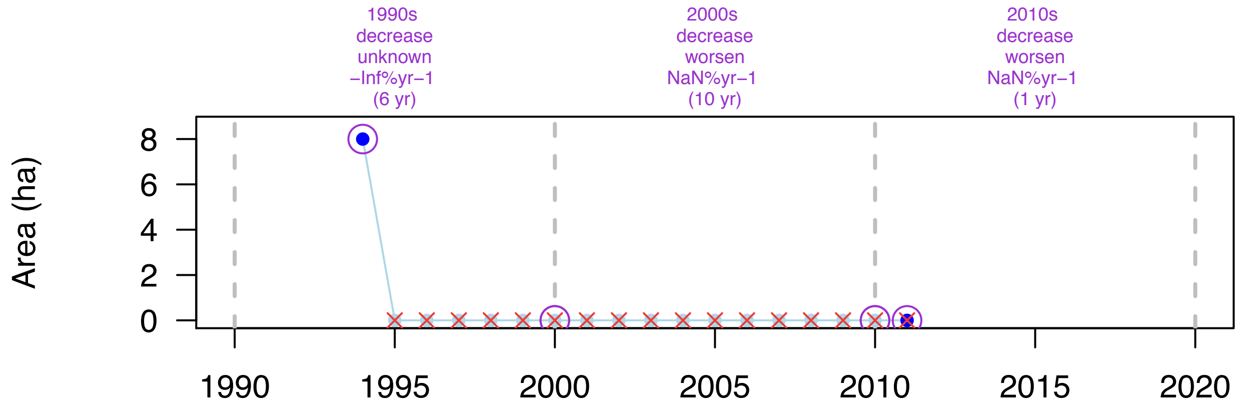

800_cover

Cook (unpublished)

SITE: Portsmouth Harbour (coastal) (United Kingdom – Atlantic) – Zm (? m)

OVERALL: Net = –15.88 %;   Rate = –20.03 % yr–1; Perc Final = 67 % > decrease

DECADAL: NO (2 yr)

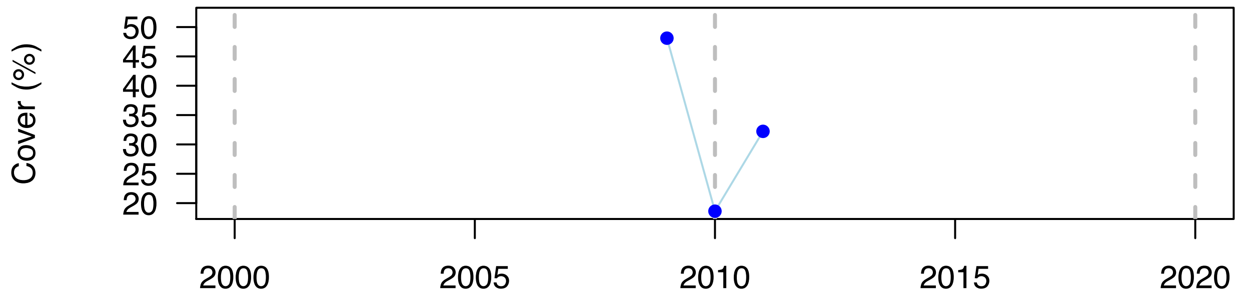

803_area

Polderman and Den Hartog 1975, de Jong (unpublished)

SITE: Balgzand (The Netherlands – Atlantic) – Zn (–0.6 m)

OVERALL: Net = –47.4 ha; Rate = NA % yr–1; Perc Final = NA % > decrease

DECADAL: YES (17 yr)

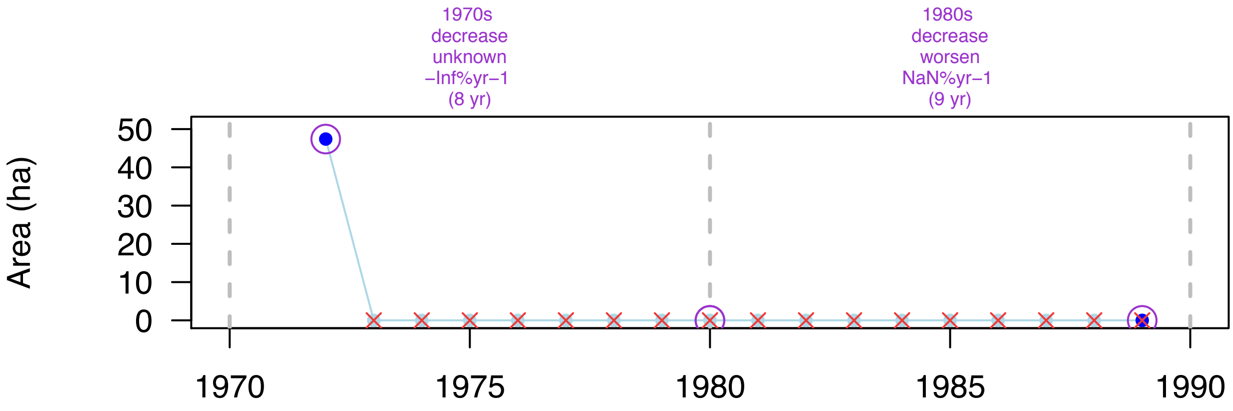

803_lowerlimit

Polderman and Den Hartog 1975, de Jong (unpublished)

SITE: Balgzand (The Netherlands – Atlantic) – Zn (–0.6 m)

OVERALL: Net = NA m; Rate = NA % yr–1; Perc Final = NA % > decrease

DECADAL: YES (17 yr)

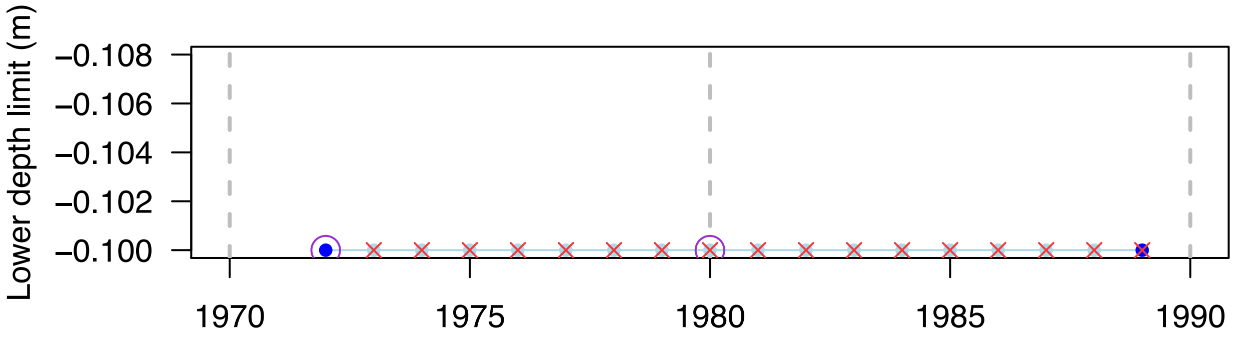

803_upperlimit

Polderman and Den Hartog 1975, de Jong (unpublished)

SITE: Balgzand (The Netherlands – Atlantic) – Zn (–0.6 m)

OVERALL: Net = NA m; Rate = NA % yr–1; Perc Final = NA % > decrease

DECADAL: YES (17 yr)

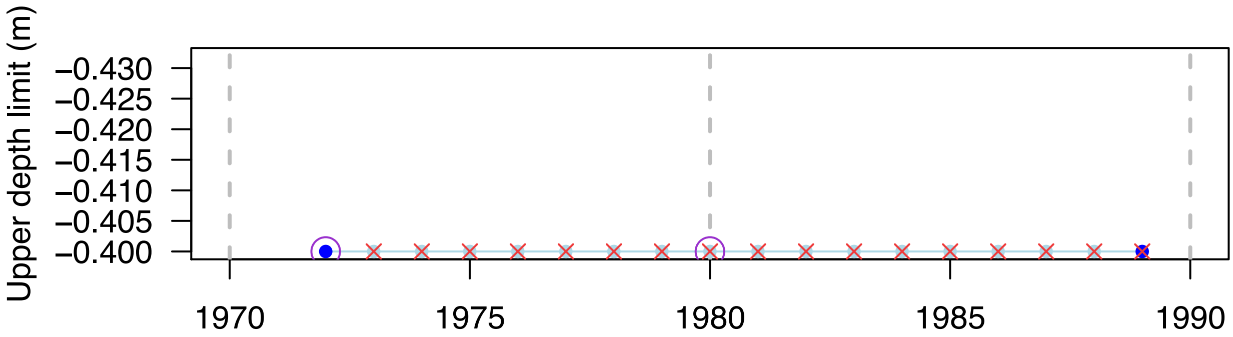

806_area

Kastler and Michaelis 1999

SITE: Borkum (Germany – Atlantic) – Zn (? m)

OVERALL: Net = 22.9 ha; Rate = 1.11 % yr–1; Perc Final = 131 % > increase

DECADAL: YES (24 yr)

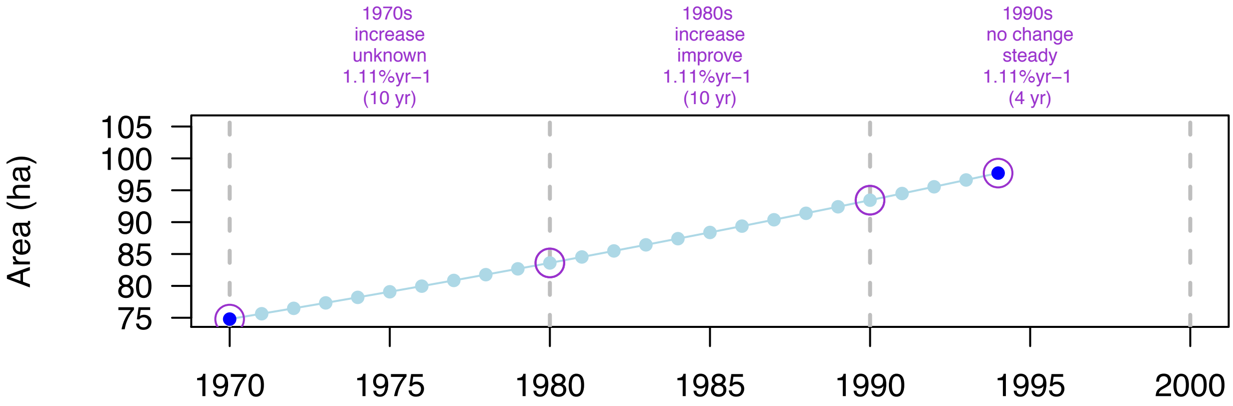

807_area
Kastler and Michaelis 1999
SITE: Juist (Germany – Atlantic) – Zn (? m)
OVERALL: Net = –728.1 ha;   Rate = –16.34 % yr–1; Perc Final = 2 % > decrease
DECADAL: YES (24 yr)

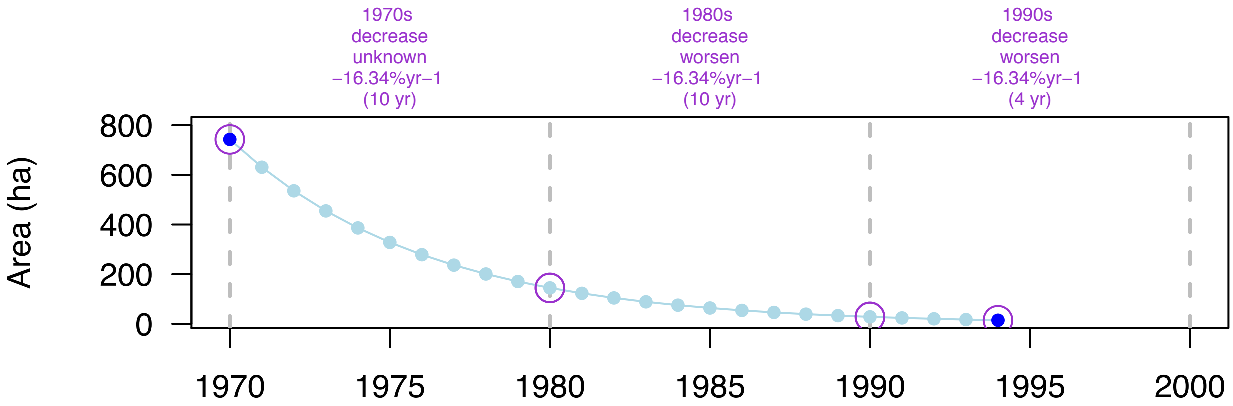

808_area
Kastler and Michaelis 1999
SITE: Norderney (Germany – Atlantic) – Zn (? m)
OVERALL: Net = –395.5 ha;   Rate = –14.59 % yr–1; Perc Final = 3 % > decrease
DECADAL: YES (24 yr)

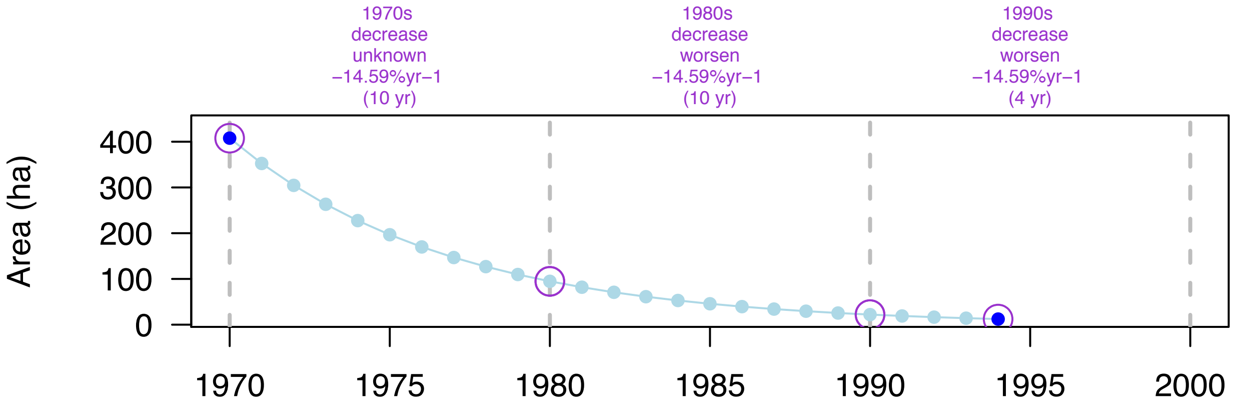

809_area

Kastler and Michaelis 1999

SITE: Baltrum (Germany – Atlantic) – Zn (? m)

OVERALL: Net = –178.6 ha; Rate = NA % yr–1; Perc Final = NA % > decrease

DECADAL: YES (24 yr)

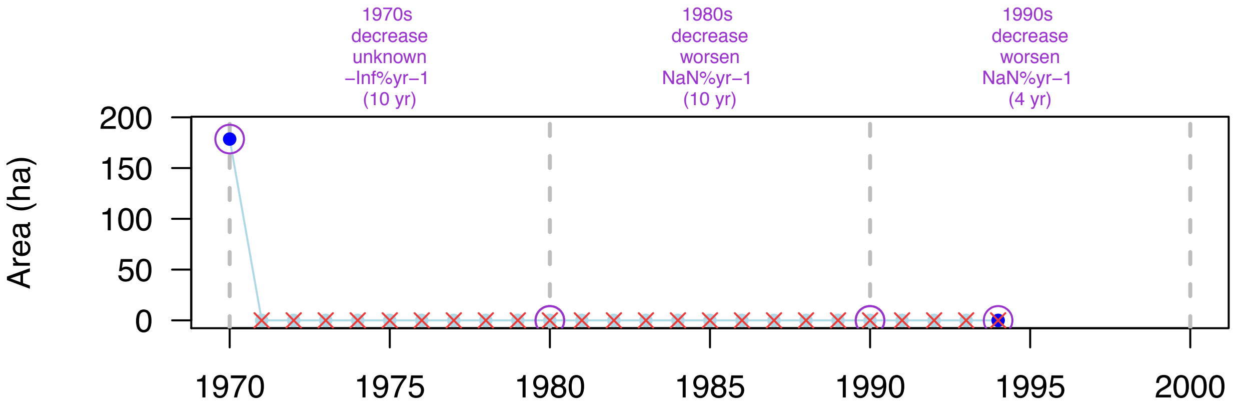

810_area

Kastler and Michaelis 1999

SITE: Langeoog (Germany – Atlantic) – Zn (? m)

OVERALL: Net = –332.9 ha; Rate = –14.2 % yr–1; Perc Final = 3 % > decrease

DECADAL: YES (24 yr)

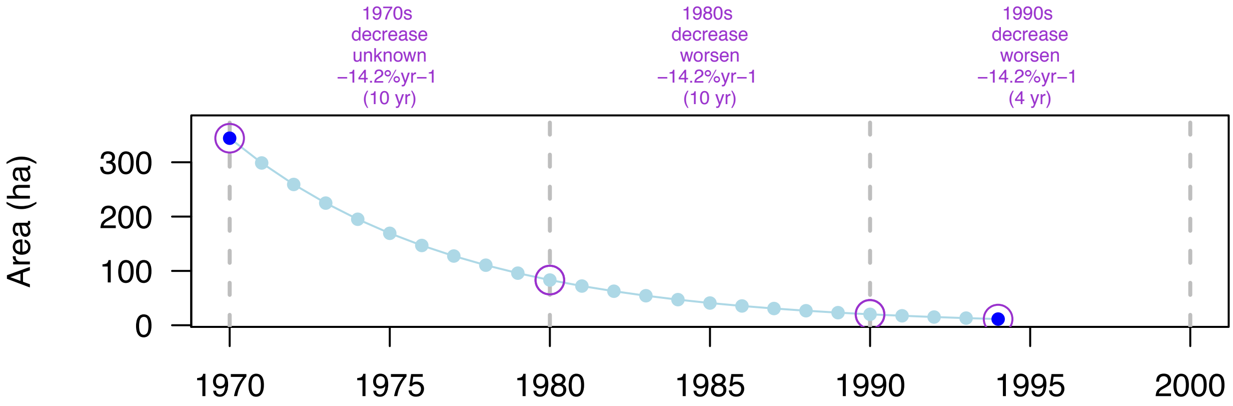

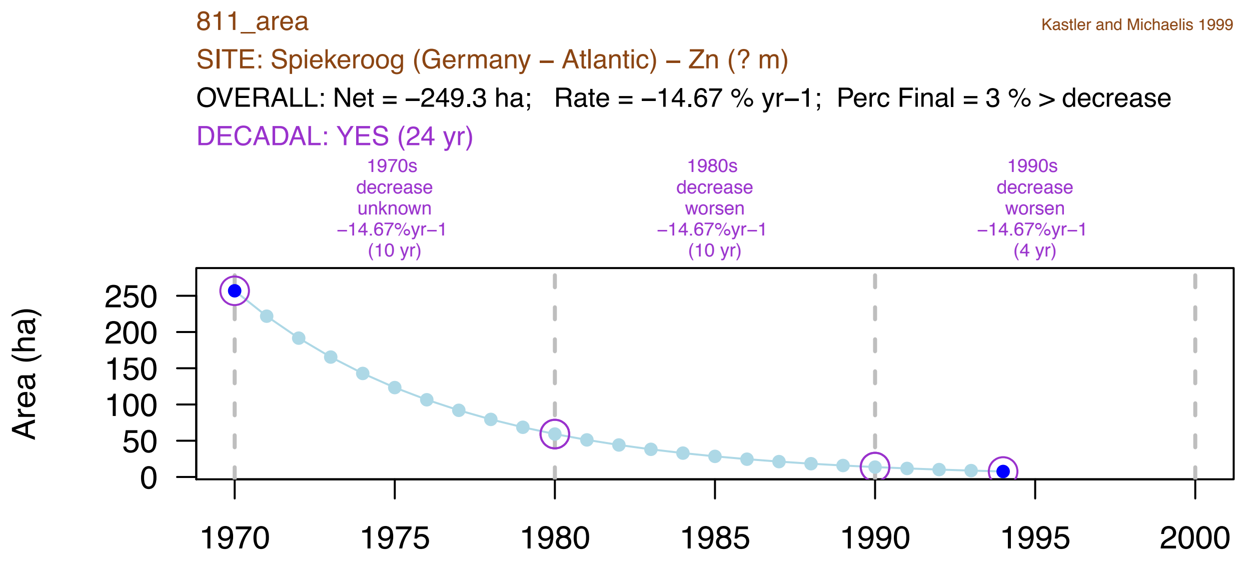
811_area
Kastler and Michaelis 1999
SITE: Spiekeroog (Germany – Atlantic) – Zn (? m)
OVERALL: Net = –249.3 ha; Rate = –14.67 % yr–1; Perc Final = 3 % > decrease
DECADAL: YES (24 yr)
1970s decrease unknown –14.67%yr–1 (10 yr)
1980s decrease worsen –14.67%yr–1 (10 yr)
1990s decrease worsen –14.67%yr–1 (4 yr)
Area (ha)
250
200
150
100
50
0
1970
1975
1980
1985
1990
1995
2000

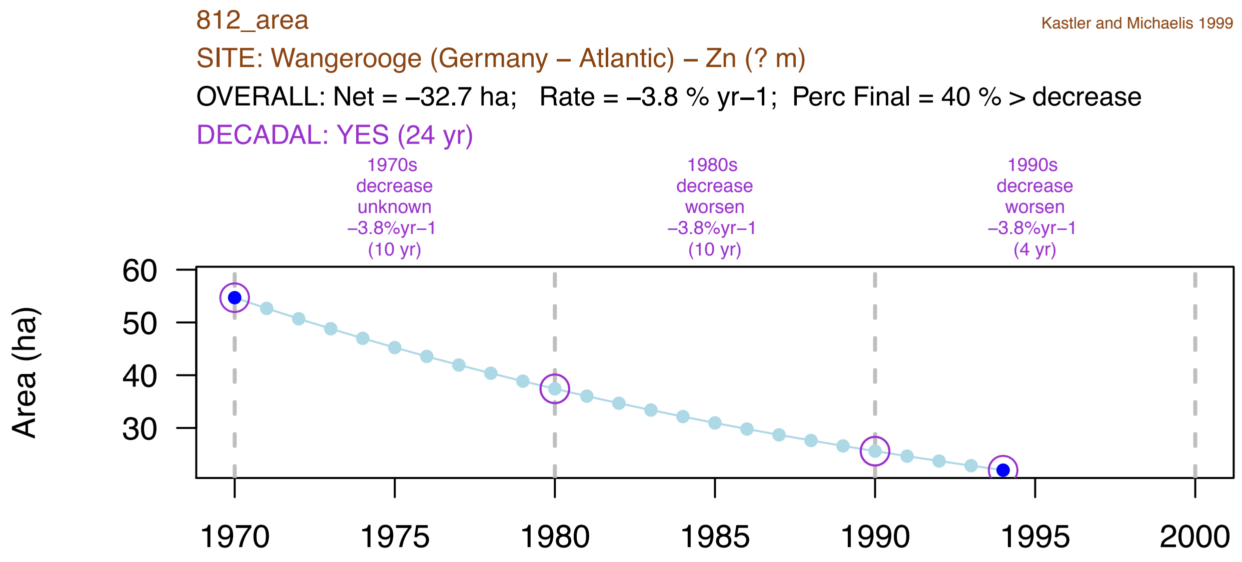
812_area
Kastler and Michaelis 1999
SITE: Wangerooge (Germany – Atlantic) – Zn (? m)
OVERALL: Net = –32.7 ha; Rate = –3.8 % yr–1; Perc Final = 40 % > decrease
DECADAL: YES (24 yr)
1970s decrease unknown –3.8%yr–1 (10 yr)
1980s decrease worsen –3.8%yr–1 (10 yr)
1990s decrease worsen –3.8%yr–1 (4 yr)
Area (ha)
60
50
40
30
1970
1975
1980
1985
1990
1995
2000

813_area

Kastler and Michaelis 1999

SITE: Wilhelmshaven (Germany – Atlantic) – Zn (? m)

OVERALL: Net = −175.1 ha;   Rate = −1.66 % yr−1; Perc Final = 67 % > decrease

DECADAL: YES (24 yr)

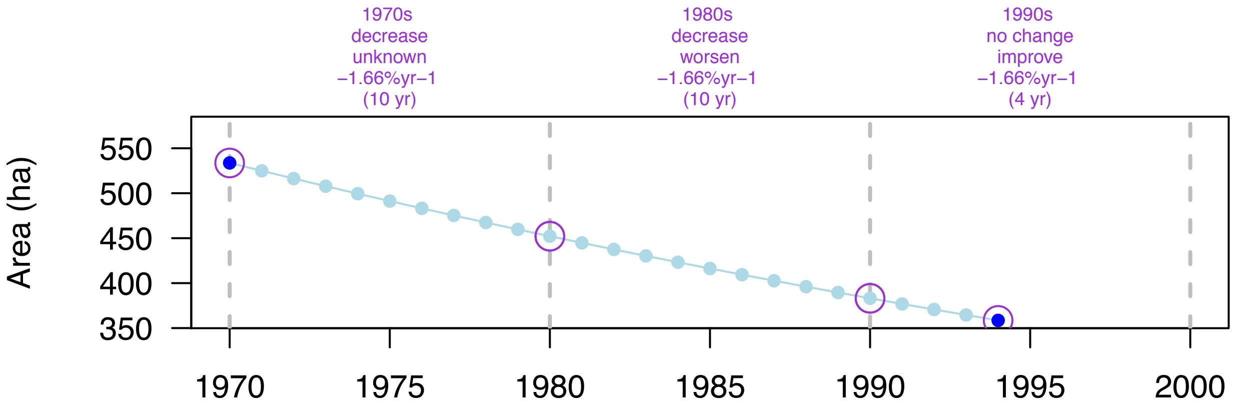

814_area

Kastler and Michaelis 1999

SITE: Jade Bright (Germany – Atlantic) – Zn (? m)

OVERALL: Net = −257 ha;   Rate = NA % yr−1; Perc Final = NA % > decrease

DECADAL: YES (24 yr)

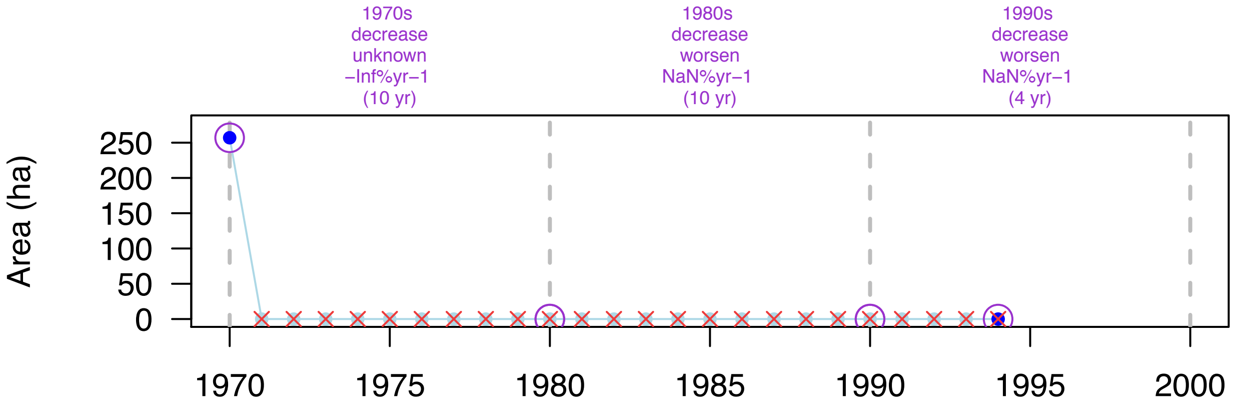

815_area
Kastler and Michaelis 1999
SITE: Weser Estuary (Germany – Atlantic) – Zn (? m)
OVERALL: Net = –503.5 ha;   Rate = –13.98 % yr–1; Perc Final = 3 % > decrease
DECADAL: YES (24 yr)

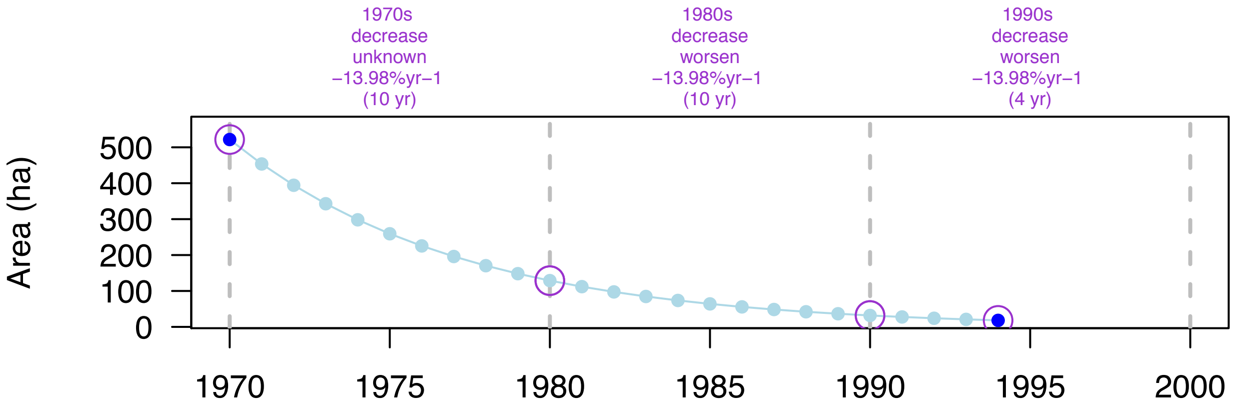

816_area
Kastler and Michaelis 1999
SITE: Bremerhaven (Germany – Atlantic) – Zn (? m)
OVERALL: Net = –2.8 ha;   Rate = –0.06 % yr–1; Perc Final = 99 % > no change
DECADAL: YES (24 yr)

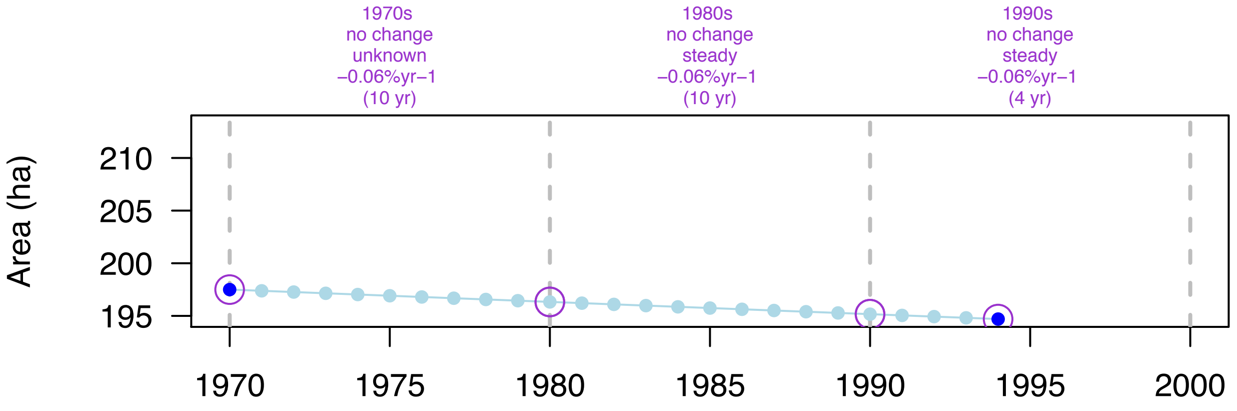

817_density
Jakl et al. 2015
SITE: Cuska Dumboka (Croatia – Mediterranean) – Po (? m)
OVERALL: Net = 4.31 shoot m–2;   Rate = 0.82 % yr–1; Perc Final = 102 % > no change
DECADAL: NO (3 yr)

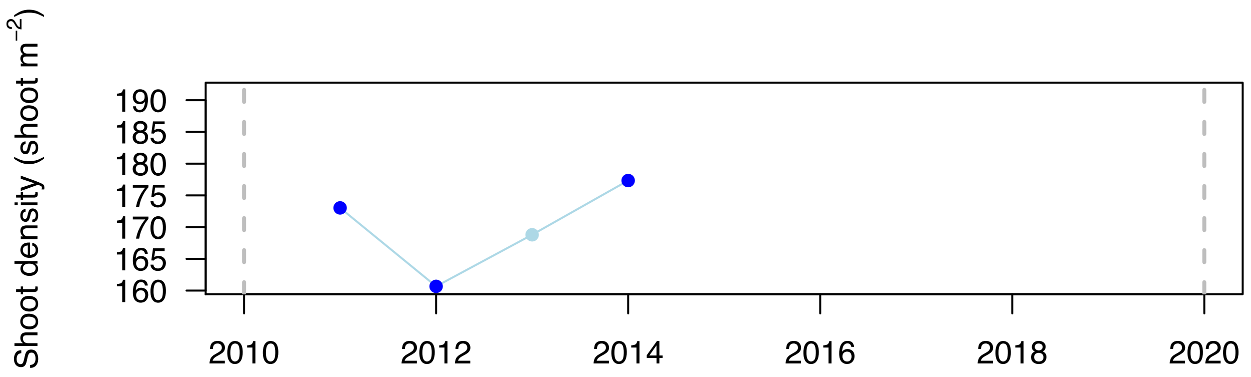

818_density
Jakl et al. 2015
SITE: Kobiljak (Croatia – Mediterranean) – Po (? m)
OVERALL: Net = 14.62 shoot m–2;   Rate = 2.51 % yr–1; Perc Final = 108 % > no change
DECADAL: NO (3 yr)

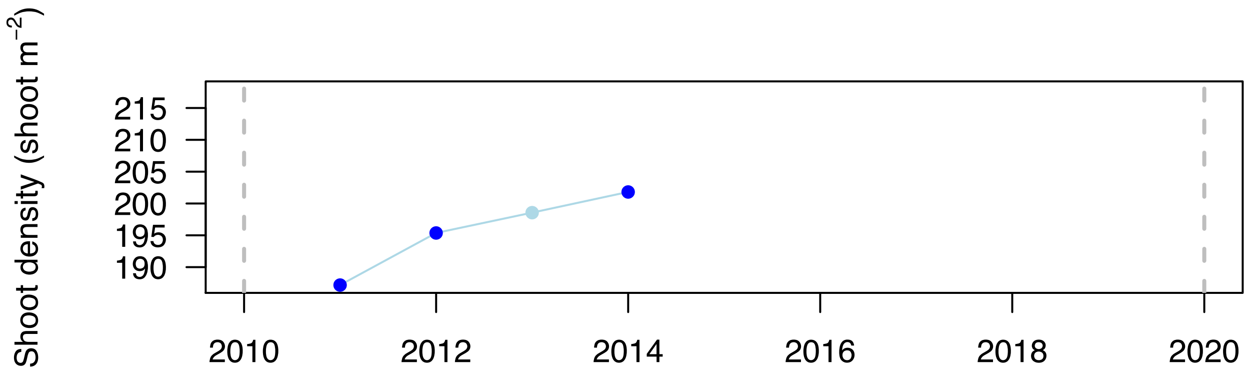

819_density

Jakl et al. 2015

SITE: Lucica (Croatia – Mediterranean) – Po (? m)

OVERALL: Net = 65 shoot m–2; Rate = 9.15 % yr–1; Perc Final = 132 % > increase

DECADAL: NO (3 yr)

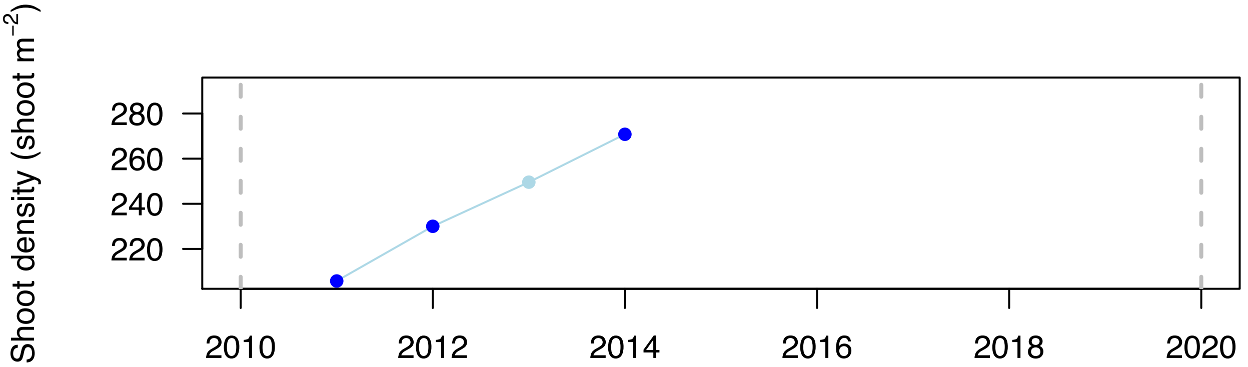

820_density

Jakl et al. 2015

SITE: Sestrica (Croatia – Mediterranean) – Po (? m)

OVERALL: Net = –4.99 shoot m–2; Rate = –0.72 % yr–1; Perc Final = 98 % > no change

DECADAL: NO (3 yr)

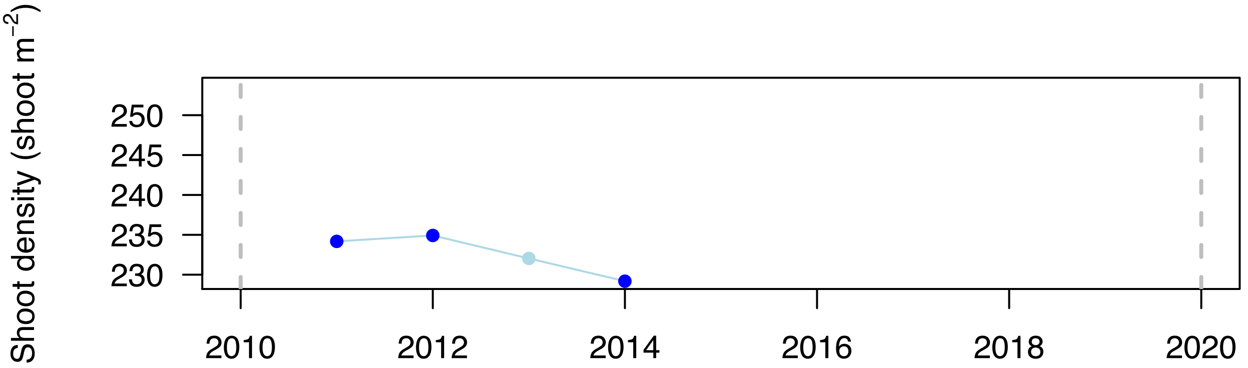

821_density

Jakl et al. 2015

SITE: Garmenjak (Croatia – Mediterranean) – Po (? m)

OVERALL: Net = −2.32 shoot m−2; Rate = −0.22 % yr−1; Perc Final = 99 % > no change

DECADAL: NO (3 yr)

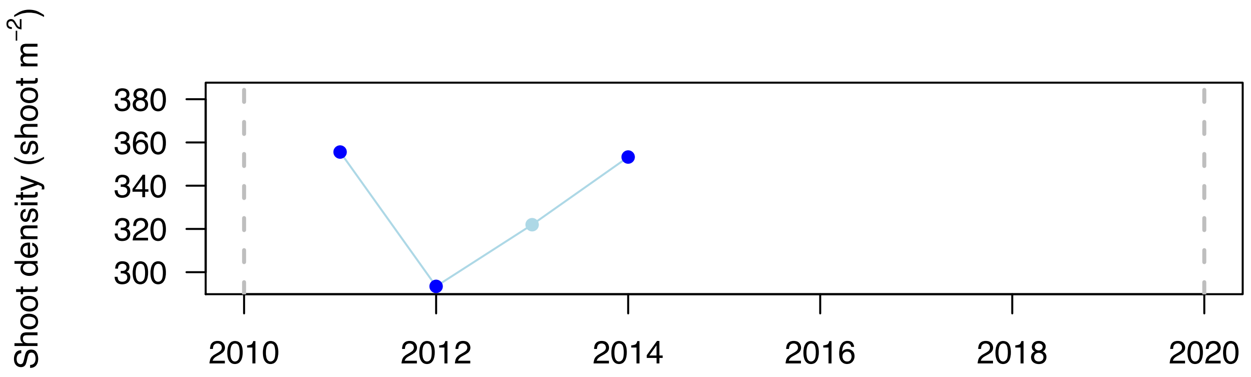

830_area

Bertelli et al. 2017

SITE: Angle Bay (United Kingdom – Atlantic) – Zn (? m)

OVERALL: Net = 35.46 ha; Rate = 10.27 % yr−1; Perc Final = 779 % > increase

DECADAL: YES (20 yr)

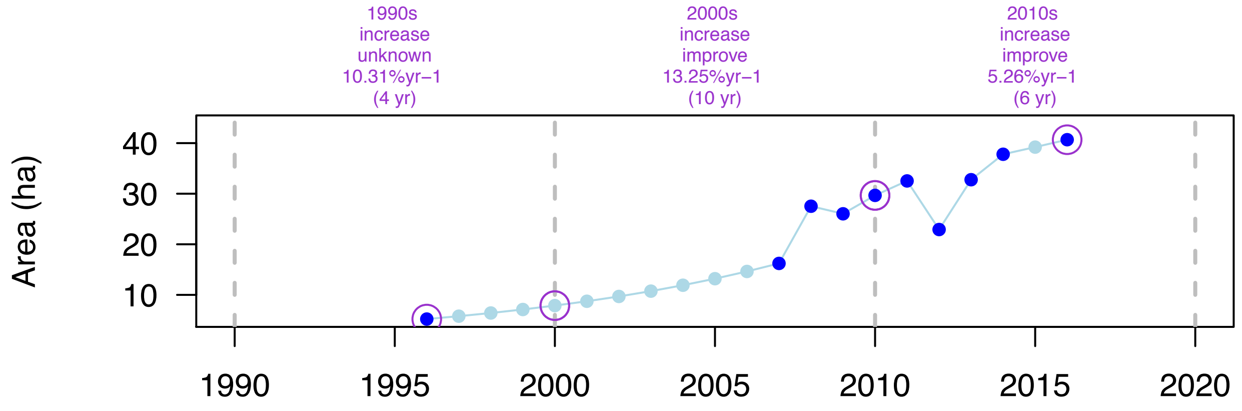

830_cover

Bertelli et al. 2017

SITE: Angle Bay (United Kingdom – Atlantic) – Zn (? m)

OVERALL: Net = −0.28 %;   Rate = −0.04 % yr−1; Perc Final = 99 % > no change

DECADAL: YES (20 yr)

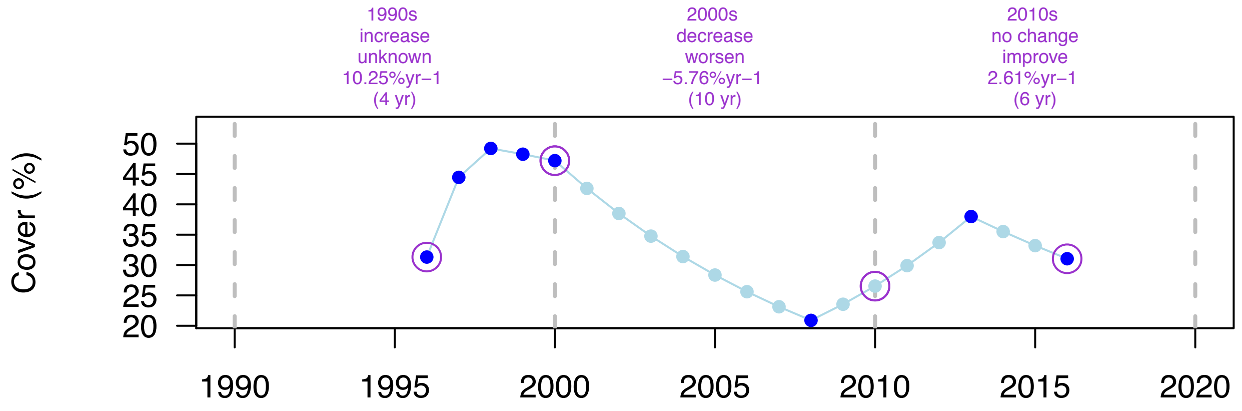

831_area

Bertelli et al. 2017

SITE: Carew (United Kingdom – Atlantic) – Zn (? m)

OVERALL: Net = 3.74 ha;   Rate = 9.72 % yr−1; Perc Final = 218 % > increase

DECADAL: YES (8 yr)

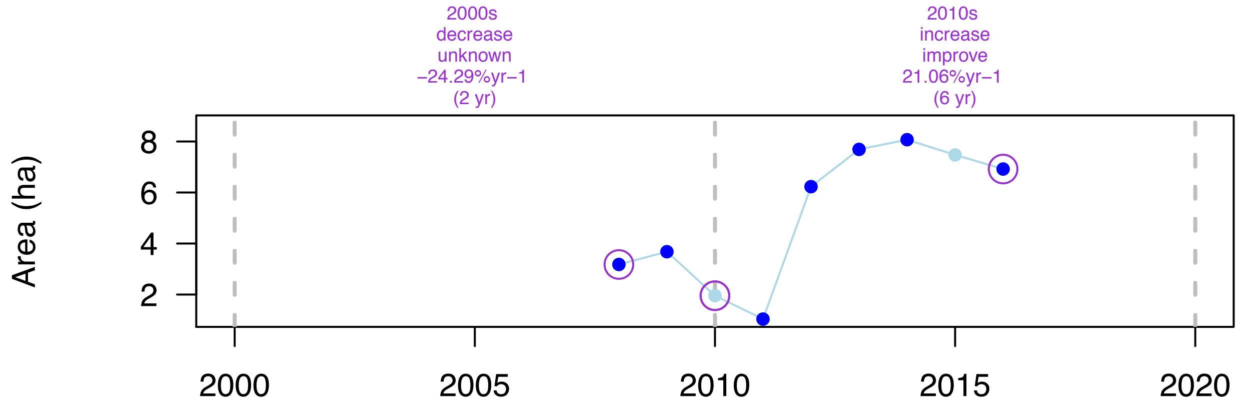

832_area
Bertelli et al. 2017
SITE: Cosheston (United Kingdom – Atlantic) – Zn (? m)
OVERALL: Net = 0.45 ha; Rate = 17.6 % yr–1; Perc Final = 288 % > increase
DECADAL: NO (6 yr)

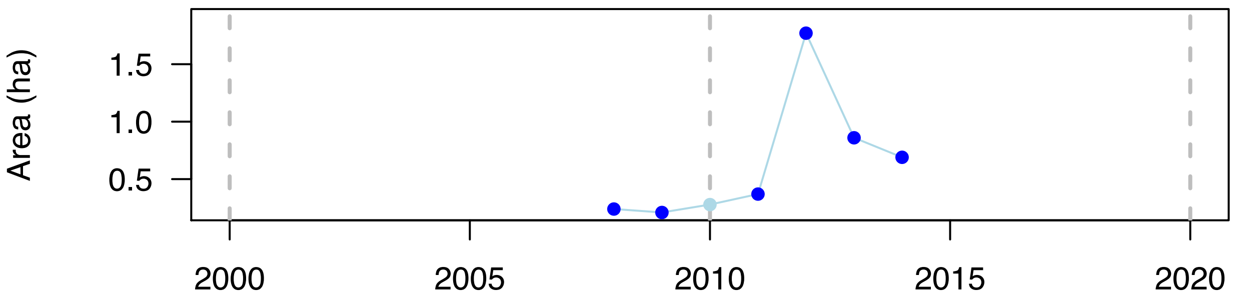

833_area
Bertelli et al. 2017
SITE: Garron pill (United Kingdom – Atlantic) – Zn (? m)
OVERALL: Net = 0.13 ha; Rate = 0.35 % yr–1; Perc Final = 103 % > no change
DECADAL: YES (8 yr)

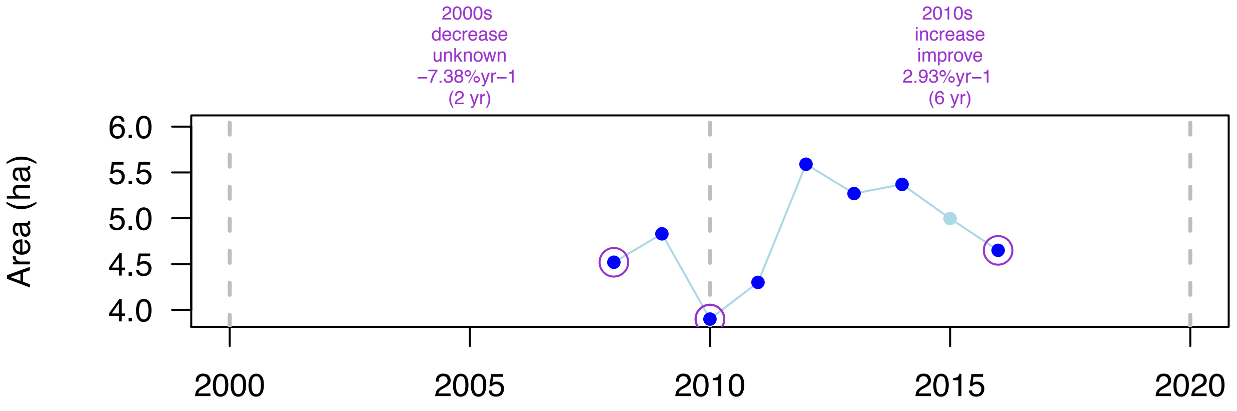

834_area

Bertelli et al. 2017

SITE: Hobbs point (United Kingdom – Atlantic) – Zn (? m)

OVERALL: Net = 1.4 ha; Rate = 6.41 % yr–1; Perc Final = 167 % > increase

DECADAL: YES (8 yr)

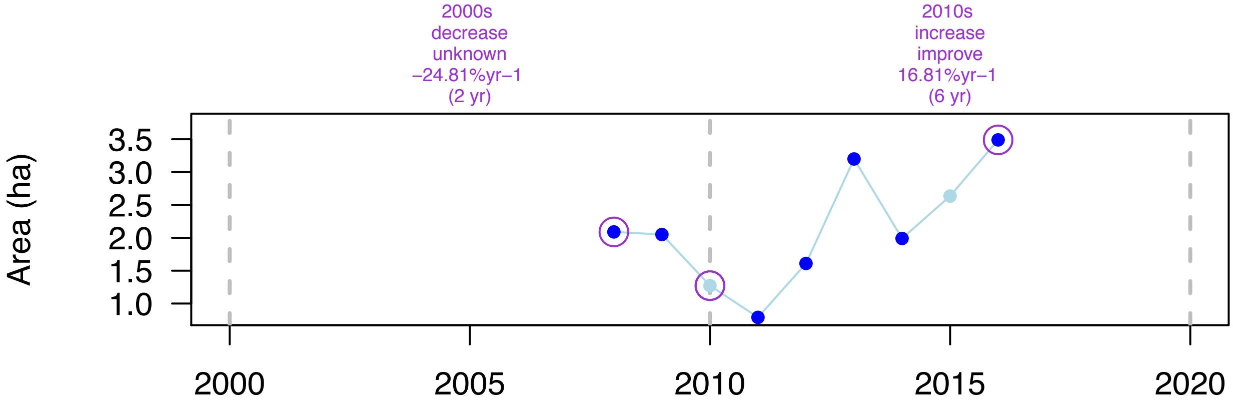

835_area

Bertelli et al. 2017

SITE: Pembroke river (United Kingdom – Atlantic) – Zn (? m)

OVERALL: Net = 42.42 ha; Rate = 8.17 % yr–1; Perc Final = 177 % > increase

DECADAL: NO (7 yr)

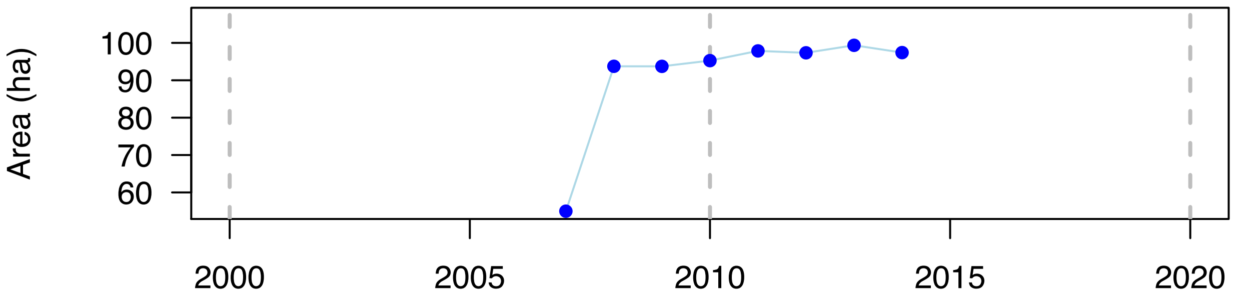

835_cover

Bertelli et al. 2017

SITE: Pembroke river (United Kingdom – Atlantic) – Zn (? m)

OVERALL: Net = 5.99 %; Rate = 1.31 % yr–1; Perc Final = 108 % > no change

DECADAL: NO (6 yr)

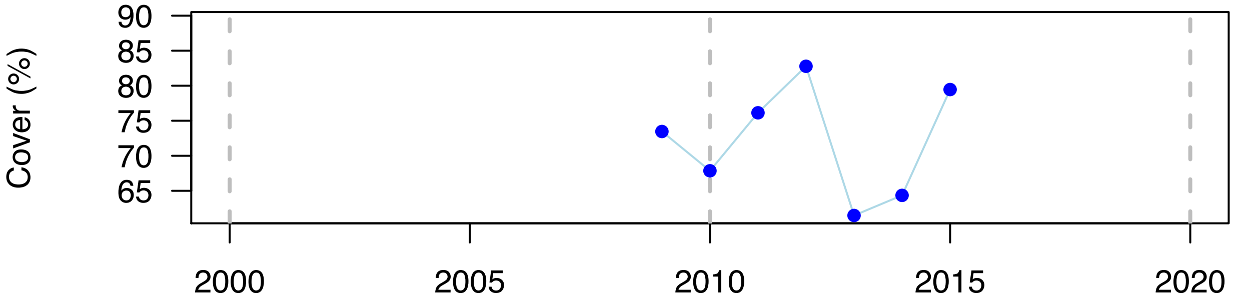

836_area

Bertelli et al. 2017

SITE: Pwllcrochan flats (United Kingdom – Atlantic) – Zn (? m)

OVERALL: Net = 3.68 ha; Rate = 39 % yr–1; Perc Final = 2265 % > increase

DECADAL: YES (8 yr)

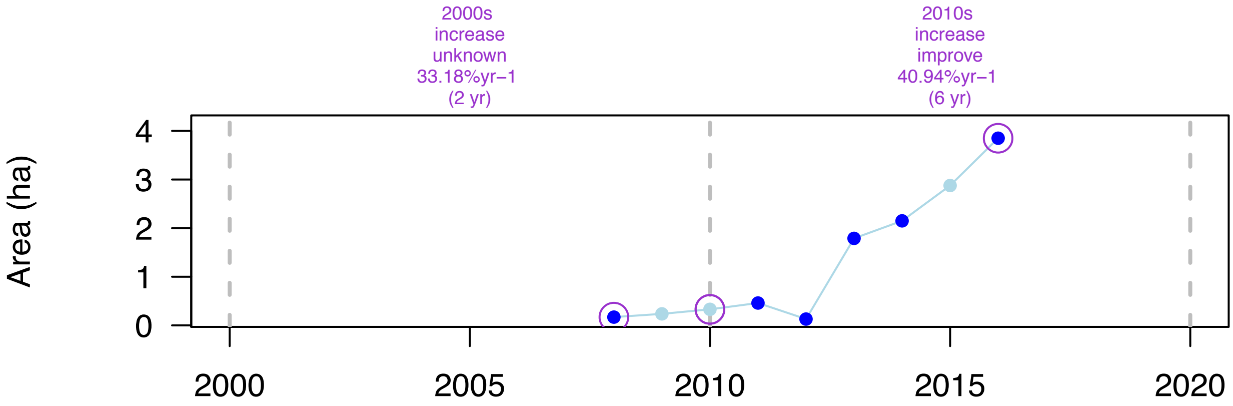

837_area

Bertelli et al. 2017

SITE: Sprinkle pill (United Kingdom – Atlantic) – Zn (? m)

OVERALL: Net = 0.16 ha;   Rate = 8.66 % yr–1; Perc Final = 200 % > increase

DECADAL: YES (8 yr)

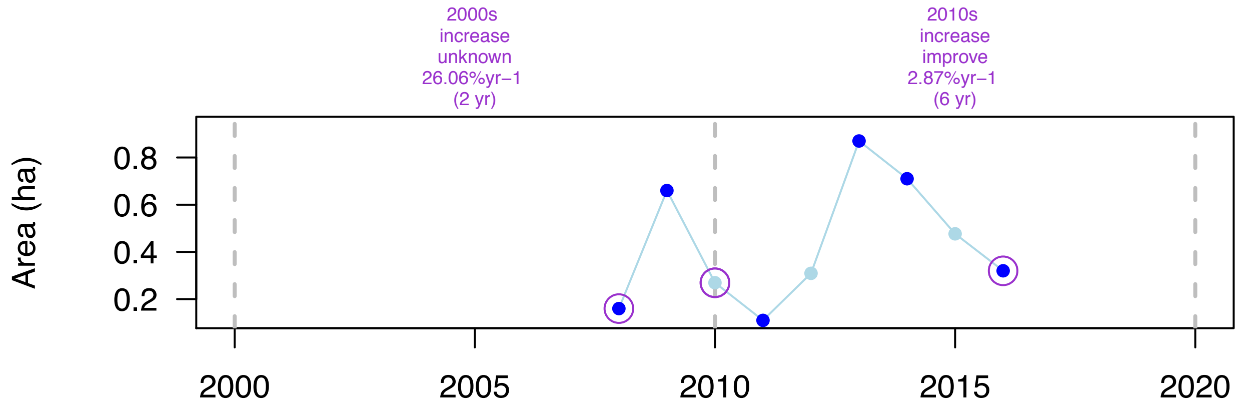

856_density

Lorenti et al. 2005

SITE: Lacco Ameno (Italy – Mediterranean) – Po (–5 m)

OVERALL: Net = –106.06 shoot m–2;   Rate = –13.98 % yr–1; Perc Final = 76 % > no change

DECADAL: NO (2 yr)

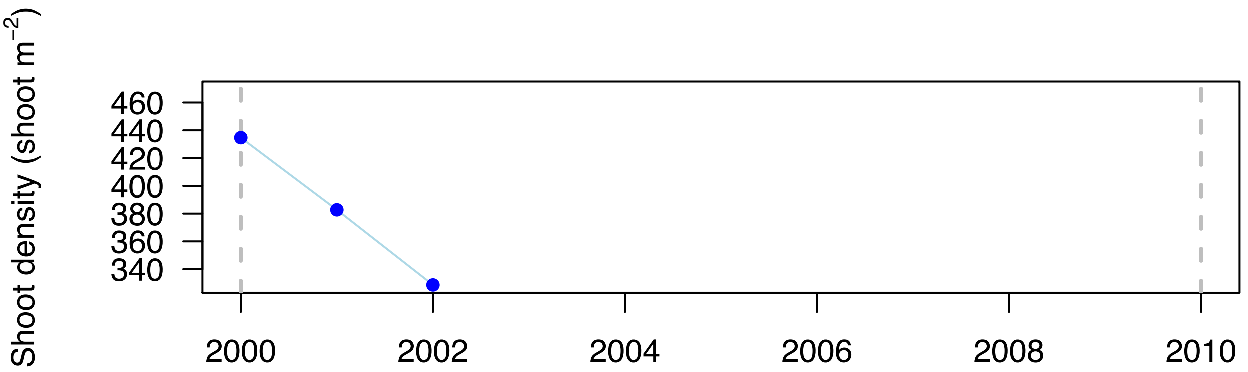

857_density

Lorenti et al. 2005

SITE: Lacco Ameno (Italy – Mediterranean) – Po (–10 m)

OVERALL: Net = 5.38 shoot m–2;   Rate = 0.86 % yr–1; Perc Final = 102 % > no change

DECADAL: NO (2 yr)

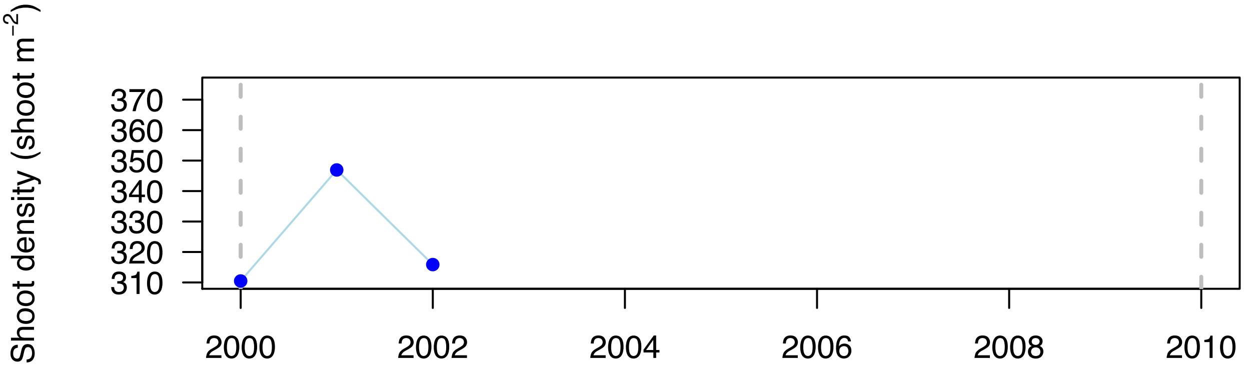

858_density

Lorenti et al. 2005

SITE: Lacco Ameno (Italy – Mediterranean) – Po (–30 m)

OVERALL: Net = 14.24 shoot m–2;   Rate = 7.41 % yr–1; Perc Final = 116 % > no change

DECADAL: NO (2 yr)

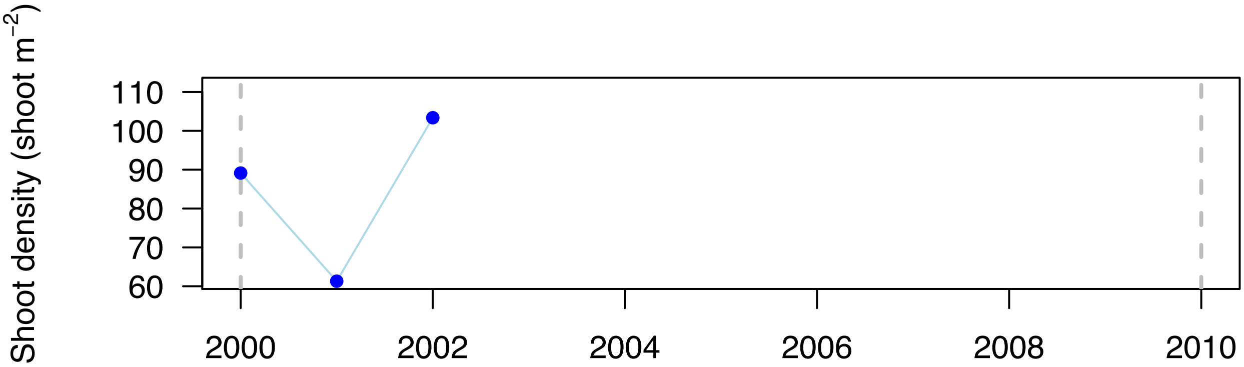

859_density

Lorenti et al. 2005

SITE: Off Scarrupata (Italy – Mediterranean) – Po (–10 m)

OVERALL: Net = 28.79 shoot m–2;   Rate = 2.88 % yr–1; Perc Final = 106 % > no change

DECADAL: NO (2 yr)

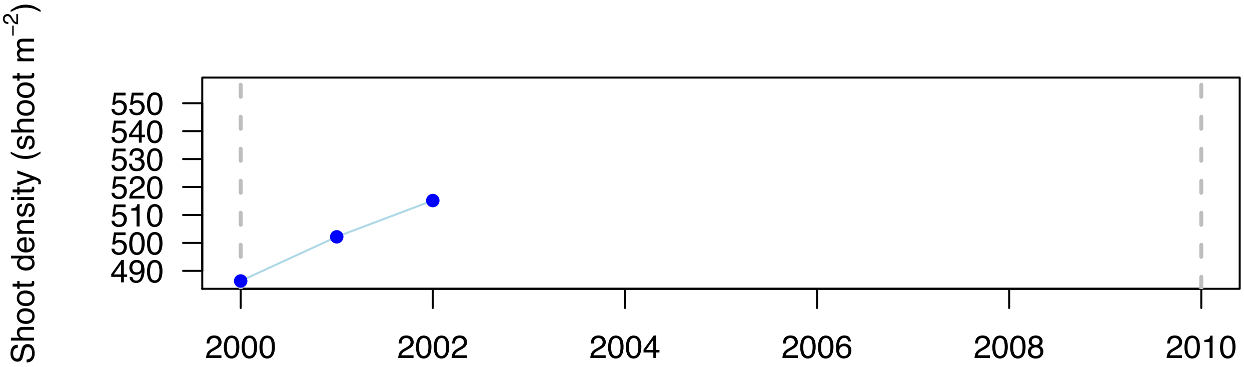

860_density

Lorenti et al. 2005

SITE: Off Scarrupata (Italy – Mediterranean) – Po (–30 m)

OVERALL: Net = 4.89 shoot m–2;   Rate = 1.49 % yr–1; Perc Final = 103 % > no change

DECADAL: NO (2 yr)

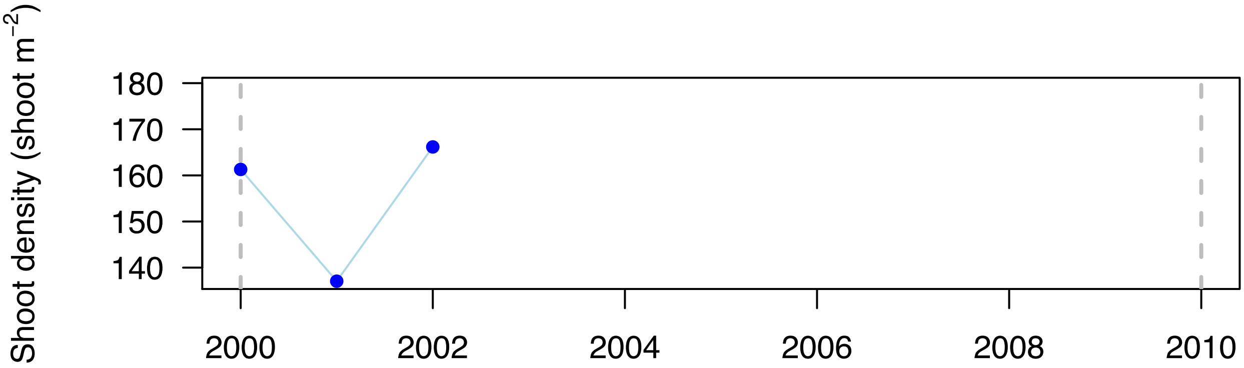

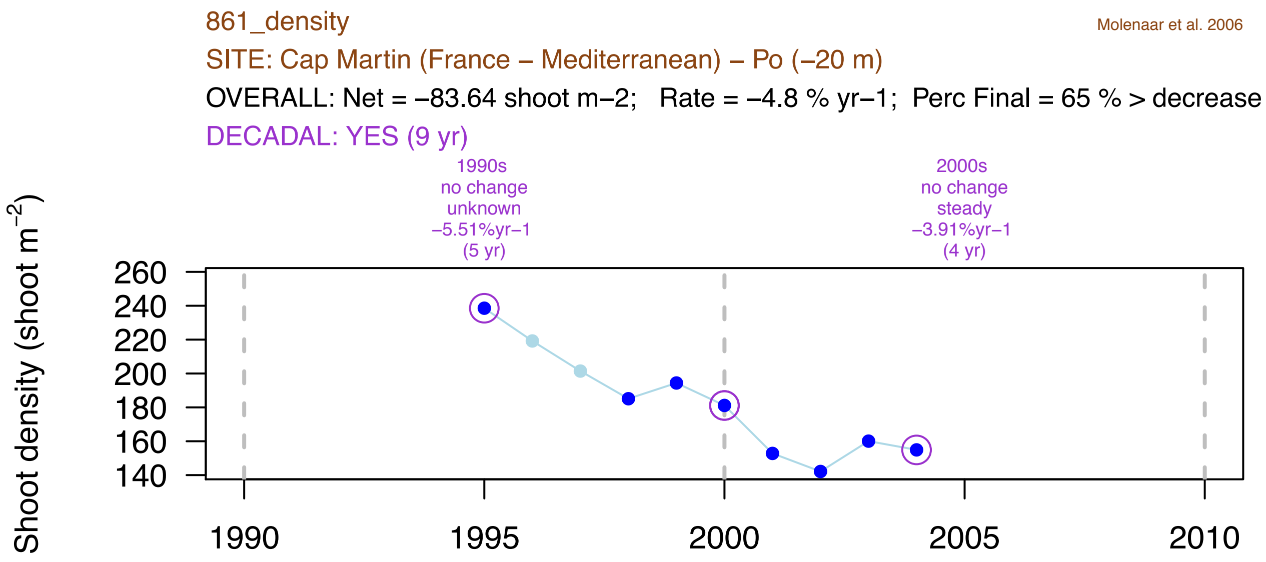
861_density
Molenaar et al. 2006
SITE: Cap Martin (France – Mediterranean) – Po (–20 m)
OVERALL: Net = –83.64 shoot m–2; Rate = –4.8 % yr–1; Perc Final = 65 % > decrease
DECADAL: YES (9 yr)
1990s
no change
unknown
–5.51%yr–1
(5 yr)
2000s
no change
steady
–3.91%yr–1
(4 yr)
Shoot density (shoot m–2)
260
240
220
200
180
160
140
1990
1995
2000
2005
2010

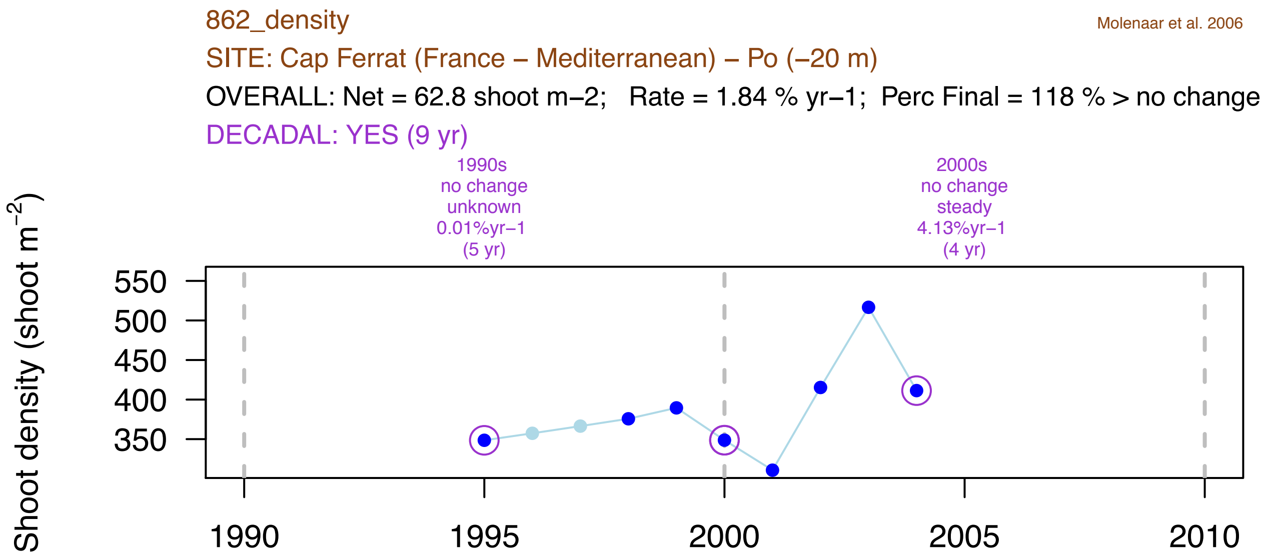
862_density
Molenaar et al. 2006
SITE: Cap Ferrat (France – Mediterranean) – Po (–20 m)
OVERALL: Net = 62.8 shoot m–2; Rate = 1.84 % yr–1; Perc Final = 118 % > no change
DECADAL: YES (9 yr)
1990s
no change
unknown
0.01%yr–1
(5 yr)
2000s
no change
steady
4.13%yr–1
(4 yr)
Shoot density (shoot m–2)
550
500
450
400
350
1990
1995
2000
2005
2010

863_density

Molenaar et al. 2009

SITE: Cap d’Antibes (France – Mediterranean) – Po (–6 m)

OVERALL: Net = –50.7 shoot m–2;   Rate = –1.32 % yr–1; Perc Final = 88 % > no change

DECADAL: YES (10 yr)

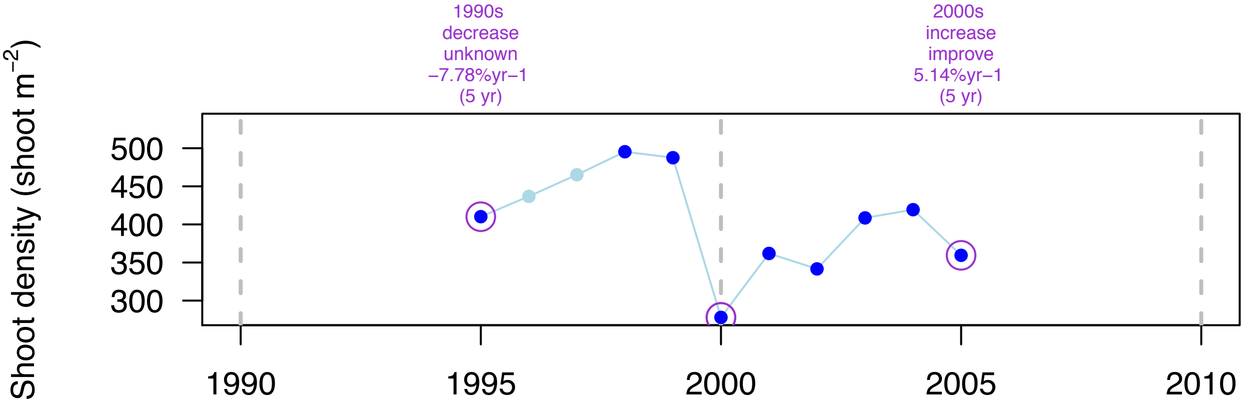

864_density

Molenaar et al. 2009

SITE: Cap Martin (France – Mediterranean) – Po (–6 m)

OVERALL: Net = –551.47 shoot m–2;   Rate = –16.28 % yr–1; Perc Final = 20 % > decrease

DECADAL: YES (10 yr)

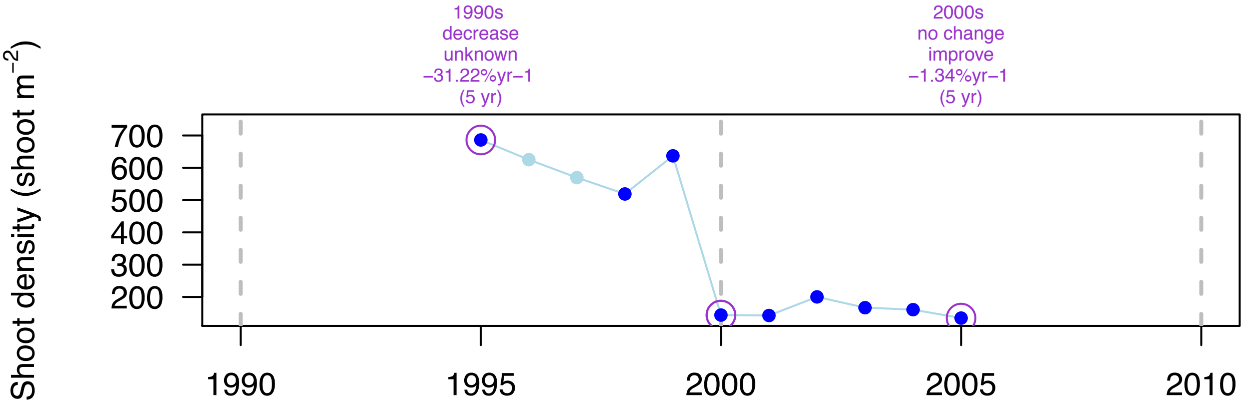

875_area

Procaccini et al. 2003

SITE: Islas Medes (entire) (Spain – Mediterranean) – Po (? m)

OVERALL: Net = 0 ha; Rate = 0 % yr–1; Perc Final = 100 % > no change

DECADAL: YES (17 yr)

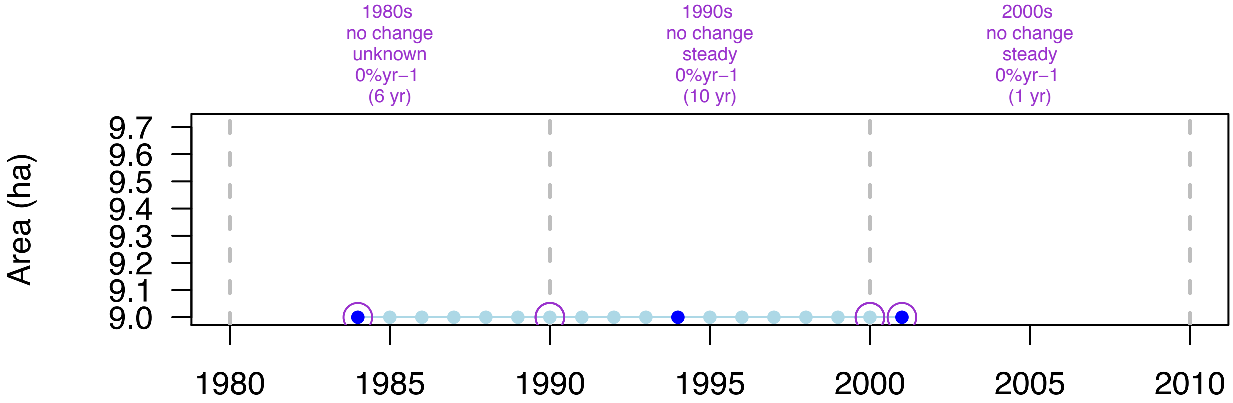

Supplement: Supplementary file 6 — Supplementary Data 2 [file 41467_2019_11340_MOESM6_ESM.pdf]
